# α-Lipoic acid induces Endoplasmic Reticulum stress-mediated apoptosis in hepatoma cells

**DOI:** 10.1038/s41598-020-64004-5

**Published:** 2020-04-28

**Authors:** Monica Pibiri, Pia Sulas, Tania Camboni, Vera Piera Leoni, Gabriella Simbula

**Affiliations:** 1 Department of Biomedical Sciences, Unit of Oncology and Molecular Pathology, University of Cagliari, Cagliari, Italy; 2 National Research Council, Institute of Biomedical Technologies, Segrate, Milano Italy

**Keywords:** Apoptosis, Apoptosis, Targeted therapies, Targeted therapies

## Abstract

Hepatocellular carcinoma (HCC) is the most common liver cancer and a major cause of adult death. The current treatments for HCC suffer from drug resistance and poor prognosis; therefore, novel therapeutic agents are urgently needed. Phytochemicals have been proposed to treat a range of cancers. Among them, α-lipoic acid (α-LA), a naturally synthesized antioxidant found in various dietary animal and plant sources, prevents oxidant-mediated cell death in normal cells while inducing apoptosis in several cancer cell lines. Previously, we demonstrated that the treatment of hepatoma cells with α-LA induced apoptosis, which was preceded by the generation of reactive oxygen species (ROS) and activation of the p53 protein, a known inducer of mitochondria-mediated apoptosis. Several studies have shown that ROS-induced apoptosis is associated with endoplasmic reticulum (ER) stress and Unfolded Protein Response (UPR) activation. Herein, we investigated if α-LA-induced apoptosis in hepatoma cell lines was ER stress- and UPR-mediated by gene expression profiling analyses. UPR and ER stress pathways were the most up-regulated after treatment with α-LA. This finding, which has been confirmed by expression analyses of ER- and UPR-associated proteins, provides a better understanding of the molecular mechanisms behind the anti-tumoral action of α-LA on hepatoma cells.

## Introduction

Hepatocellular carcinoma (HCC) is the most frequent and deadliest primary hepatic carcinoma worldwide^1,2^. Since it is commonly diagnosed at an intermediate or advanced stage, surgery supported by adjuvant chemotherapies currently represent the elective therapeutic strategy^3,4^. Unfortunately, given the poor selectivity of anticancer drugs between highly proliferating non-tumor and tumor cells, which could favor malignant progression and resistance to treatment, HCC is widely regarded as a chemotherapy-resistant disease^5^. Therefore, novel approaches are required to successfully treat this tumor type. Recently, phytochemicals have been regarded as anticancer compounds alternative to classical chemotherapy^6^. Indeed, in virtue of their anticancer potential coupled with fewer side effects compared to classic chemotherapy agents, the use of phytochemicals has opened-up new research avenues^6–8^. Among them, α-Lipoic acid (α-LA), also known as thioctic acid, is a naturally occurring antioxidant compound either found in a various dietary sources or endogenously synthetized. In addition to its action as cofactor for several mitochondrial enzyme complexes^9,10^ and to its well-established anti-oxidative and anti-inflammatory properties^11–13^, a growing number of studies have provided evidence that α-LA prevents cell death in normal cells, while causing apoptosis in several cancer cell lines^14–17^. Accordingly, we have previously shown that treatment of hepatoma cells with α-LA induces mitochondria-mediated apoptosis that is preceded by increased Reactive Oxygen Species (ROS) generation and p53 activation^14^. However, the precise molecular mechanism(s) underlying the pro-apoptotic action of α-LA on cancer cells is not yet completely understood. Accumulating evidences suggest that, in addition to mitochondria, ROS play a central role in the regulation of an important apoptotic point of control, the Endoplasmic reticulum (ER)^18^. ER is the cell site for Ca^2+^ storage and for synthesis, folding, and maturation of most secreted and transmembrane proteins^19^. Physiological and pathological processes, such as toxic insults, failure of protein synthesis, folding and transport or degradation and calcium overload, can result in ER stress and activation of a set of signaling pathways collectively termed as Unfolded Protein Response (UPR)^20,21^. Although the UPR program is primarily activated to restore the ER homeostasis, thus preventing loss of cell viability, sustained and/or prolonged stress may result in cell death induction by apoptosis^22^. Therefore, understanding the mechanism(s) regulating the cell survival/death decision under ER stress condition may be crucial in order to identify specific tumor cell targets and, possibly, overcomes the HCC resistance to therapy. Furthermore, recently a straight connection between mitochondria- and ER stress-induced apoptosis has been observed^23,24^. On these bases, in the present study we aimed to understand if ER stress and UPR activation could be involved in α-LA-induced apoptosis. To this end, the effect of α-LA has been investigated in both rat and human hepatoma cells, FaO and HepG2, respectively. Data obtained have shown that α-LA was able to induce modifications in the gene and protein expression of the principal regulators of UPR and ER stress response^25–28^, such as Protein Disulfide Isomerase (PDI)^29^ and the ER stress sensors PKR-like ER Kinase (PERK)^30,31^, Inositol-requiring enzyme 1 alpha (IRE1)^30,32^ and Activation transcription factor 6 (ATF6)^30,33^. In particular, in both cell lines the loss of viability was preceded by activation of the ER-related pro-apoptotic gene C/EBP Homologues Protein/DNA Damage Inducible Transcription 3 (GADD153/CHOP)^30^. In summary, our results have provided evidence that α-LA induces apoptosis in hepatoma cells by triggering chronic ER stress and UPR response.

## Results

### α-LA treatment induces apoptosis of FaO cells

**Figure 1:**
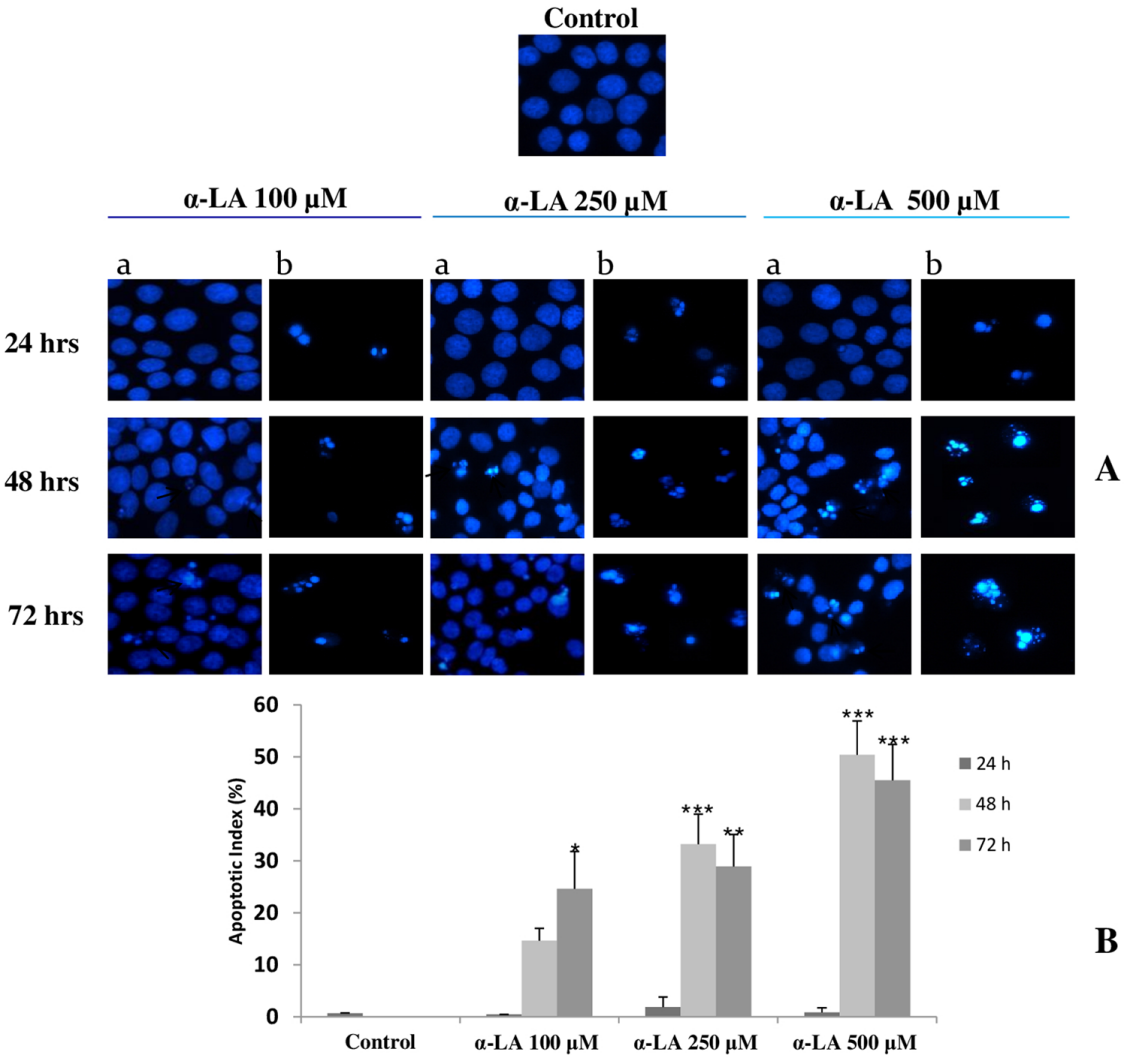
Induction of apoptosis after exposure to increasing concentrations of α-LA **(A)** FaO cells were exposed to 100, 250 and 500 µM α-LA for 24, 48 and 72 hours and subjected to Hoechst 33258 nuclear staining; with **a** are indicated stained attached cells and with **b** apoptotic detached cells. (**B)** Percentage of apoptotic cells (Apoptotic Index). Data are shown as means ± S.E. Assay was performed in quadruplicate. Significantly different from control cells for *p* < 0.05*; *p* < 0.01 **; *p* < 0.001***.

As previously described^14^, treatment of FaO cells with α-LA results in mitochondria mediated-apoptosis that is preceded by ROS production. Accordingly, nuclear staining with Hoechst 33258, here performed on FaO cells treated with increasing doses of α-LA, has shown nuclear condensation typical of apoptosis (Fig. 1A). This was particularly evident at 48 and 72 hours after α-LA treatment (Fig. 1B), being most effective at 500 μM dose (Fig. 1A,B).

### α-LA-treated FaO cells show dysregulation of genes involved in ER stress and UPR pathways

**Figure 2:**
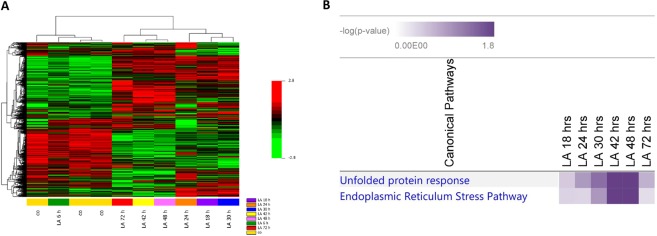
Gene expression profiling in α-LA-treated FaO cells. (**A**) Hierarchical clustering of genes expression profile in α-LA-treated cells. Each row represents the expression profile of a gene and each column represents a sample. Controls and treated cells at different time points, from 6 up to 72 hours, are indicated by colored bars. Red and green colors represent higher and low relative gene expression, respectively. (**B**) Functional analysis of differentially expressed genes in α-LA-treated cells.

**Figure 3:**
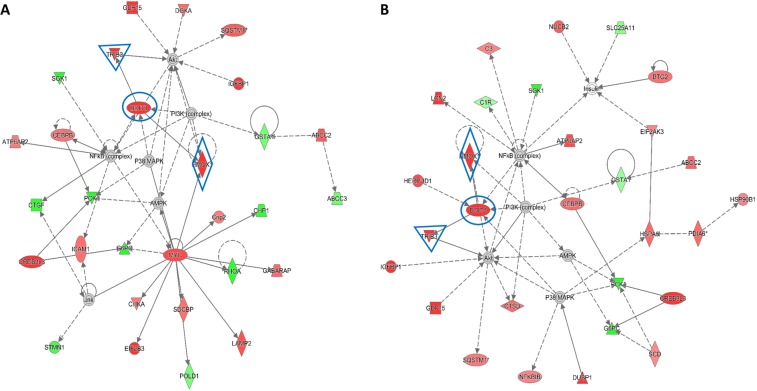
Activated network in α-LA-treated FaO cells. Network visualization of the transcription factor GADD153/CHOP (DDIT3) after 24 (**A**) and 48 (**B**) hours of treatment. Genes were colored based on their expression (Red for up-regulated genes; Green for down-regulated genes). Interested genes (*Ddit3*, *Hmox1* and *Trib3*) were highlighted in blue.

**Figure 4:**
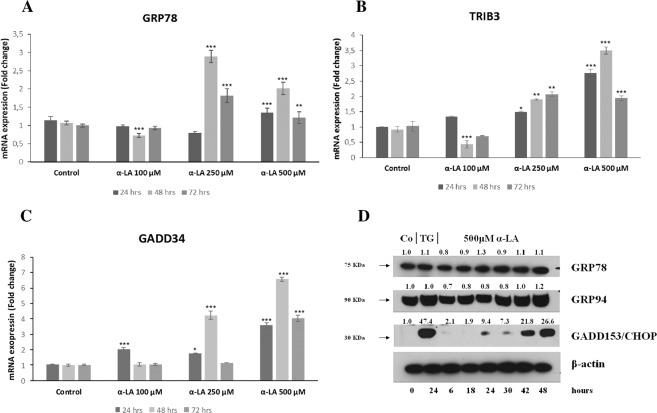
Involvement of ER stress in the onset of apoptosis α-LA-mediated in FaO hepatoma cells. **(A–C)** Expression profiles of ER stress representative genes *Bip/Grp78*, *Trib3* and *Gadd34* were validated with Real Time PCR analysis using RNA isolated from three biological replicate. FaO cells were exposed to 100, 250 and 500 µM α-LA for 24, 48 and 72 hours. Results are expressed as means ± SE. Significantly different from control cells for *p* < 0.05*; *p* < 0.01 **; *p* < 0.001***. (**D)** Western blot analysis of GRP78, GRP94 and GADD153/CHOP in rat FaO hepatoma cells treated with 500 µM α-LA from 6 up to 48 hours. Cells treated with 0,5 μM Thapsigargin (TG) were used as positive control. β-actin was used as loading control. For densitometric analysis specific protein expression was normalized to β-actin expression and values (reported over each band) have been expressed as fold change respect to control. Each lane represents a pool of three individual samples. Western Blot images (**D**) have been cropped for clarity with full blot presented in Supplementary Fig. 6.

Increased intracellular ROS levels has been associated to the activation of ER stress and UPR pathways^34,35^. In order to investigate the possible involvement of these pathways in α-LA-induced apoptosis, gene expression profiling was performed on FaO cells treated from 6 up to 72 hours with 500 μM α-LA, by Illumina microarray. A total of 7022 (Table I, Supplementary data), out of 21,791 genes included in the array were selected, as described in Methods section. A heat map was generated by comparing gene expression profiles of α-LA-treated cells with those of control cells over time (Fig. 2A). Unsupervised hierarchical cluster analysis has revealed the existence of two major clusters: i) control and 6 hours α-LA group and ii) from 18 up to 72 hours α-LA group. This last cluster was formed by two distinct sub clusters: early time point group (18-24-30 hours α-LA), and late time point group (42-48-72 hours α-LA) which showed evident differences in the gene expression regulation. To investigate the differentially expressed genes, the random-variance model (F-test) and the multivariate permutation test were applied. As indicated in Table II (Supplementary data), no differences were evident between control and 6 hours α-LA group while an average of 166 upregulated and 287 downregulated (FC ≥ 2.0) genes in the early time point group and 255 upregulated and 365 downregulated (FC ≥ 2.0) genes in late time point group were identified compared to control group. The genes upregulated by α-LA included the marker of ER stress-induced apoptosis, *Gadd153/Chop*, and other three genes associated to ER stress: *Grp78*^27,28^, Myeloid differentiation primary response gene 116 (*Gadd34*)^27^ and Tribbles Homolog 3 (*Trib3*)^36^. All these are involved in sensing and respond to the accumulation of unfolded and misfolded proteins during the ER stress. More in detail, while *Gadd153/Chop* showed a very early induction (6 hours), with an eleven folds increase at 42 hours (Table I, Supplementary data), of *Grp78, Gadd34* and *Trib3* showed an increased expression from two up to five folds all throughout the experimental time points. In order to identify a possible correlation among the genes found dysregulated in Fao cells after treatment with 500 μM α-LA, the Ingenuity Pathway Analysis (IPA) was accomplished. These data have provided insight for a key role of ER stress and UPR in the induction of α-LA-mediated apoptosis in hepatoma cells. Accordingly, among the canonical pathways activated by chronic exposure to α-LA, the UPR and the ER stress have resulted significantly regulated (Fig. 2B). The highest number of modified genes involved in both pathways was found at 42 and 48 hours after treatment. In particular, as shown in Supplementary Fig. 1A,B, *Perk* and the downstream effector *Gadd153/Chop (Ddit3)*, and the two ER stress chaperones *Grp78/Bip* and *Grp94* resulted overexpressed at 48 hours. Moreover, UPR pathway has resulted characterized by the overexpression of PDI, an enzymatic chaperone which generally mediates pro-survival response against different types of cell insults. Then, the Network analysis was performed and that associated to the transcription factor GADD153/CHOP (DDIT3) (Fig. 3A,B) emerged as the most important network (Network 1). In particular, *Gadd153/Chop (Ddit3)*, which the highest expression was observed between 24 and 48 hours (Table I, Supplementary data), was found associated to the casual network together with three regulators connected to each other: *Trib3*^37,38^, Alpha Serine/Threonine Kinase (*Akt*)^37,38^, Heme Oxygenase 1 (*Hmox*1)^39^ (Fig. 3A,B). Next, quantitative RT-PCR allowed to validate the modification of several ER stress-induced genes, such as *Bip/Grp78* and the targets of *Gadd153/Chop, Trib3* and Growth Arrest and DNA-Damage inducible protein 34 (*Gadd34*) (Fig. 4A–C).

### α-LA induces a time-dependent increase in ER stress protein content

**Figure 5:**
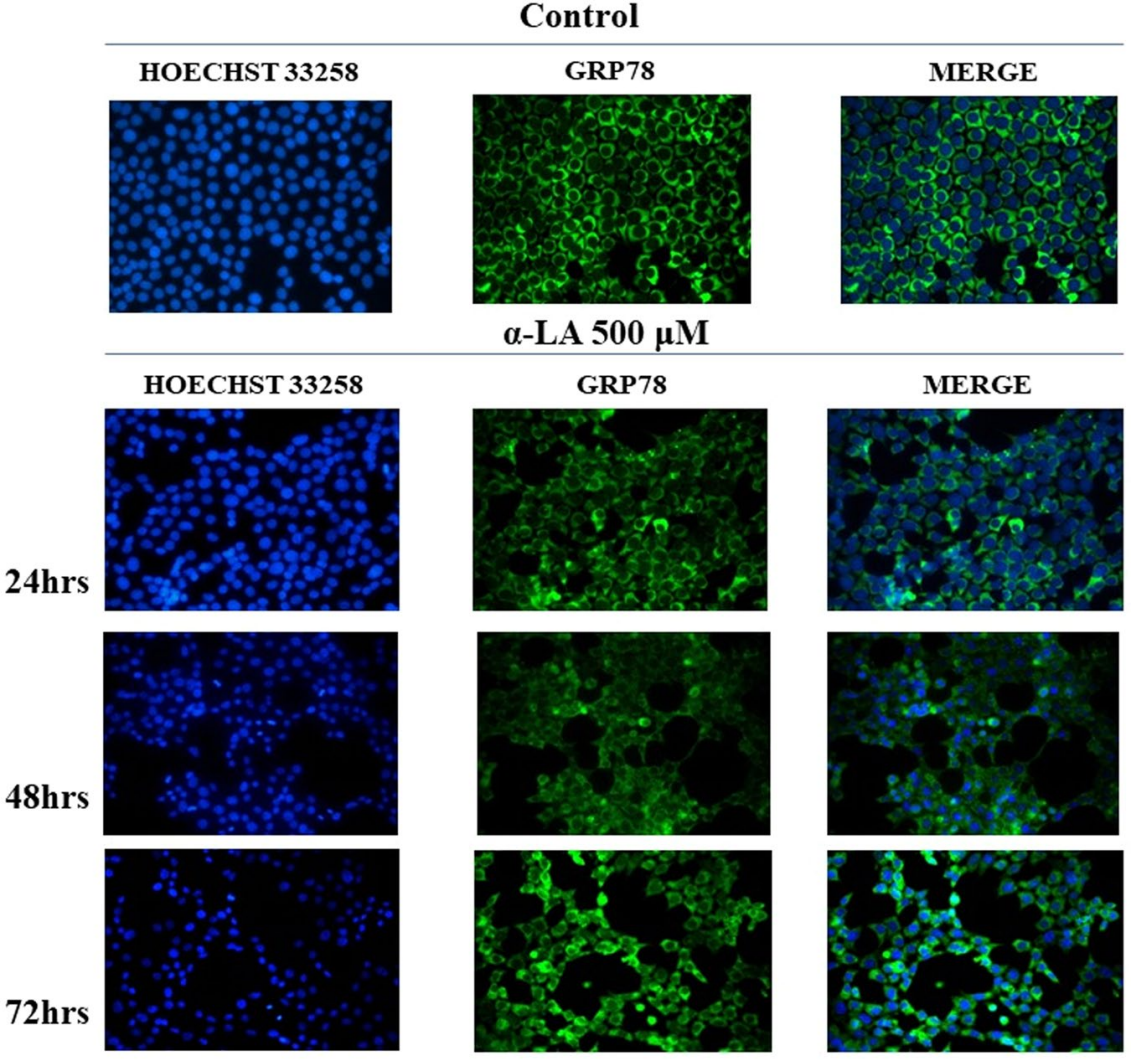
Analysis of GRP78 in rat FaO hepatoma cells after exposure to 500 uM α-LA by immunofluorescence. FaO cells were subjected to Hoechst 33258 nuclear staining or GRP78 staining after exposure to 500 μM α-LA for 24, 48 and 72 hours. Assay was performed in quadruplicate.

**Figure 6:**
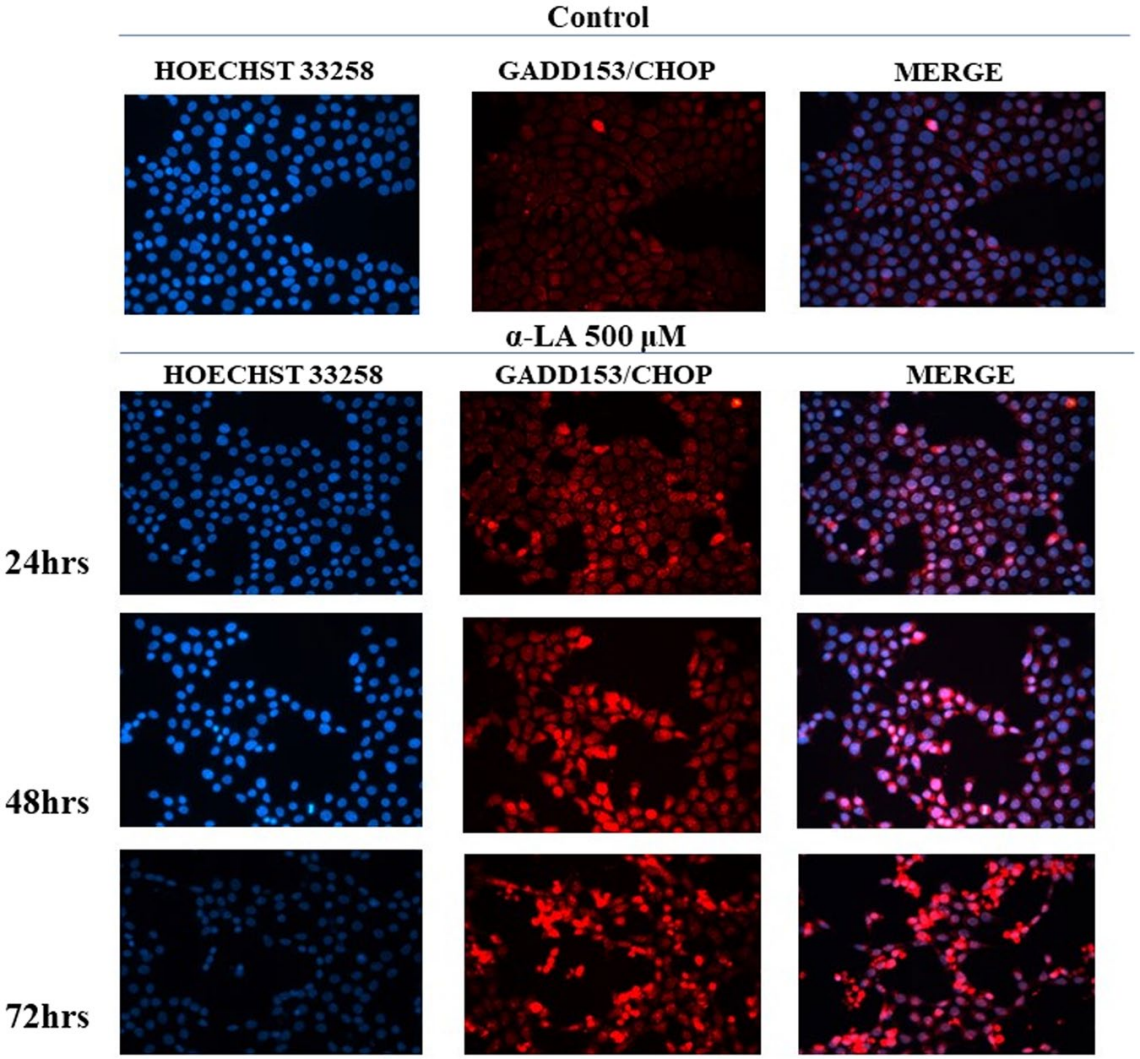
Analysis of GADD153/CHOP in rat FaO hepatoma cells after exposure to 500 uM α-LA by immunofluorescence. FaO cells were subjected to Hoechst 33258 nuclear staining or GADD153/CHOP staining after exposure to 500 uM α-LA for 24, 48 and 72 hours. Assay was performed in quadruplicate.

ER stress involvement in α-LA-induced apoptosis has been also confirmed by protein analyses through Western blot and immunofluorescence. More in details, treatment of FaO cells with 500 µM α-LA determined a progressive increase in the protein content of GADD153/CHOP while GRP78 and GRP94 essentially maintain a constant and sustained expression overtime (Fig. 4D). Thapsigargin (TG)-treated cells were used as positive control, being TG a known inducer of ER stress-mediated apoptosis^40^. The effect on GRP78 and the induction of GADD153/CHOP were also evaluated by immunofluorescence analysis. Data obtained showed a diffused cytoplasmic localization for GRP78 in most of the untreated control cells. Treatment with 500 µM α-LA, despite an increased gene expression, did not induce an appreciable increase in GRP78 protein content, and did not modify its cellular localization (Fig. 5). A constant expression of GRP78 was also observed in the presence of 250 µM and 100 µM α-LA (Supplementary Fig. 2,3). On the other hand, GADD153/CHOP protein has resulted barely present in untreated control cells while being induced in cells treated with increasing concentrations of α-LA, from 24 up to 72 hours (Fig. 6 and Supplementary Figs. 4,5). Notably, clusters of GADD153/CHOP positivity were observed in the nucleus of cells treated with 250 (Supplementary Fig. 5) and 500 µM (Fig. 6) α-LA, being this nuclear translocation time-dependent and indicative of GADD153/CHOP activation.

### α-LA activates UPR in FaO cells

**Figure 7:**
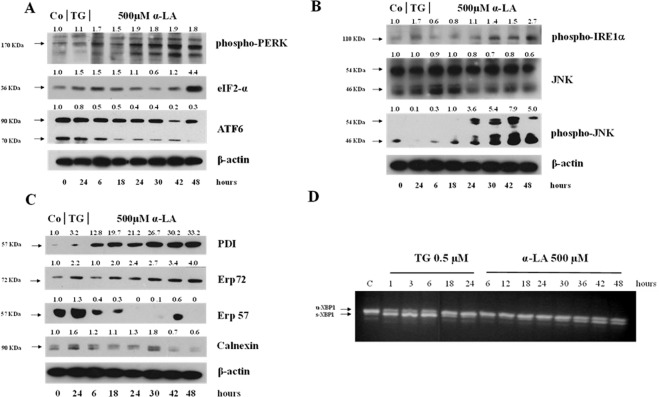
Analysis of PERK, IRE1 and ATF6 pathways and PDI proteins involved in UPR activation. Western blot analysis of (**A)** phospho-PERK, eIF2-α and ATF6, **(B)** phospho-IRE1α, total JNK and phospho-JNK (**C**) PDI, Erp72, Erp57 and calnexin in rat FaO hepatoma cells treated with 500 µM α-LA from 6 up to 48 hours. β-actin was used as loading control. For densitometric analysis specific protein expression was normalized to β-actin expression and values (reported over each band) have been expressed as fold change respect to control. Each lane represents a pool of three individual samples. TG: Thapsigargin. **(D)** Splicing of XBP-1 in rat FaO hepatoma cells after exposure to 0,5 uM TG from 1 up to 24 hours or 500 μM α-LA from 3 up to 48 hours. The data are representative of three different individual experiments.   Images (**A**–**D**) have been cropped for clarity with full blot presented in Supplementary Fig. 7.

The UPR is controlled by three distinct sensors, PERK, ATF6 and IRE1, associated to BIP/GRP78 in their inactive form. PERK is a transmembrane protein, which, once activated by oligomerization and auto-phosphorylation, mediates phosphorylation of eIF2α and induction of GADD153/CHOP, which, in turn, upregulate the transcription of GADD34. As shown in Fig. 7A, 500 µM α-LA induced an increase in phospho-PERK and eIF2α proteins, which peaked at 42 and 48 hours, respectively. As to ATF6, it is known that once it dissociated from GRP78, it is transported to the Golgi apparatus to be proteolytically cleaved. Then, the cleaved ATF6 (p50ATF6) translocates to the nucleus, where transactivates genes encoding for ER chaperones, such as GRP78, and proteins involved in ER-Associated Degradation (ERAD)^26,27^. Hereby, ATF6 activation helps the cells to cope with accumulating unfolded proteins, thus preserving their viability during ER stress. As shown in Fig. 7A, treatment with 500 µM α-LA caused a progressive decline in cleaved ATF6 protein levels. Finally, we evaluated whether also the IRE1 pathway could contribute to α-LA-induced apoptosis. It is well known that among the activities related to IRE1 activation there are the processing of unspliced XBP1 mRNA (u-XBP1), resulting in an active form (s-XBP1) encoding the bZIP transcription factor XBP1^41^, and phosphorylation of JNK^42^. Thus, the processing of XBP1 and the phospho-JNK protein levels were analyzed. XBP1 processing was evaluated by RT-PCR using oligonucleotides that flanked the splice site in the XBP1 mRNA. As shown in Fig. 7D, α-LA treatment was associated to an increasing processing of XBP1 mRNA, from 30 up to 48 hours, while it was evident as early as 1 hour after administration of TG, a known inducer of XBP1 splicing (Fig. 7D). Furthermore, Western blotting analysis showed that activated phosphorylated IRE1 protein increased starting from 30 hours after treatment while phospho-JNK protein was detected as early as 24 hours (Fig. 7B), possibly due to its involvement in other different pathways which contributed to its activation. α-LA-mediated cell death of hepatoma cells has been associated to ROS production (14) which determines alteration of the intracellular redox state. This is responsible of UPR induction due to dysregulation in protein folding. Accordingly, as shown in Fig. 7C, alteration of intracellular redox state in FaO cells resulted associated to a progressive and persistent increased expression of PDI family involved in UPR triggering. Among PDI proteins, we found a progressive increase in PDI and ERp72 protein expression, and a decrease of ERp57 and calnexin^43^ protein expression except for an unexpected increase at 42 and 30 hours, respectively.

### α-LA induces ER stress in HepG2 cells through upregulation of UPR

**Figure 8:**
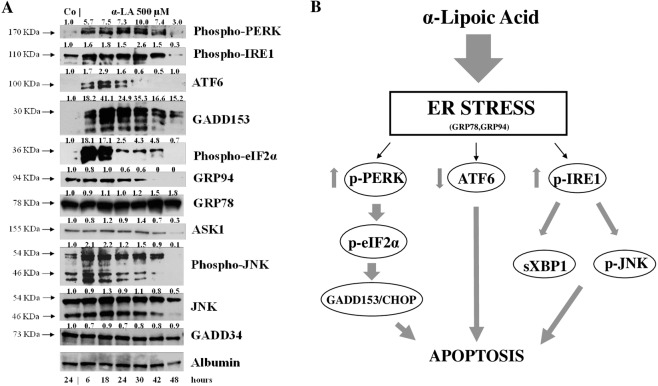
α-LA induces ER stress in hepatoma cells. (**A**) Western analysis of key players in UPR after treatment of HepG2 with 500 µM α-LA from 6 up to 48 hours. Albumin was used as loading control. For densitometric analysis protein expression was normalized to Albumin expression and values (reported over each band) have been expressed as fold change respect to control. Each lane represents a pool of three individual samples. (**B**) Schematic representation of α-LA-mediated apoptosis in hepatoma cells. Western blot images (**A**) have been cropped for clarity with full blot presented in Supplementary Fig. 8.

To confirm the involvement of ER stress in apoptosis α-LA-mediated in hepatoma cells, Western Blot analysis was performed on a human cell line, HepG2, upon treatment with 500 µM α-LA from 6 to 48 hours. As already observed in FaO cells, PERK, IRE1 and ATF6 exhibited a general upregulation trend in HepG2 cells (Fig. 8A). Downstream, GADD153/CHOP and phospho-eIF2α were upregulated up to 48 and 42 hours, respectively, while GADD34 showed a persistent and stable expression over the time (Fig. 8A). In addition, while GRP78 expression tended to be constant and sustained over the time, GRP94 levels showed a slightly decrease from 24 up to 30 hours and a roughly decreased thereafter. Finally, the IRE1-dependent slightly increased expression of Apoptotic Signaling Kinase1 (ASK1), observed at 18 and 30 hours, and of its downstream target^25^ phospho-JNK, from 6 up 30 hours, were detected, implicating that the persistent induction of IRE 1 is responsible of a proapoptotic response (Fig. 8A).

## Discussion

The natural occurring antioxidant α-LA has been found able to selectively induce cell death in several cancer cell lines^44–46^. Accordingly, we have previously shown that 500 µM α-LA inhibits cell proliferation and induces mitochondria-mediated apoptosis in hepatoma cells, preceded by ROS production and activation of p53^14^. Increased intracellular ROS levels have been associated to activation of ER stress and UPR pathways which, due to the cell death inducing effect associated to their prolonged activation, have been regarded as potential anticancer targets^47,48^. In the present study, we have provided evidence that ER is involved in α-LA-induced apoptosis in hepatoma cells. We have utilized a systems biology approach in order to analyze α-LA action not only on single gene/protein, but also at pathways and network levels. Gene expression analysis on FaO cells treated with 500 µM α-LA have revealed that although no major differences were observed between untreated and 6 hours treated cells, the total number of significantly modified genes increased progressively from 18 up to 72 hours after α-LA treatment (Table II Supplementary data). Among those genes, there were those related to ER stress and UPR response, most of which were found progressively upregulated, reaching a peak between 42 and 48 hours (Fig. 2B). It is known that under persistent ER stress, shifting from the pro-survival to the pro-apoptotic role of UPR is usually accompanied by increased expression of GADD153/CHOP, the major regulator of ER stress-related apoptosis^26^. Thus, the activation of *Perk* and *Gadd153/Chop* genes (Supplementary Fig. 1A,B), with the latter being detected as early as 6 hrs (Table I, Supplementary data), observed in FaO cells aids our hypothesis that ER stress and UPR response are involved in α-LA-mediated apoptosis. Indeed, analysis of GADD153/CHOP network (Fig. 3A,B) showing activation of its target genes *Trib3*^37,38^ and *Hmox1*^39^, further supported our hypothesis. Despite increased *Grp78* mRNA levels, immunofluorescence analysis of protein expression showed no appreciable modifications of GRP78 and GRP94 chaperones between controls and α-LA treated cells (Fig. 4D). On the opposite, an increasing expression of GADD153/CHOP (Fig. 4D) and its consequent nuclear translocation (Fig. 4C) to activate the transcription of target genes, such as *Gadd34*, was observed. From these data it emerges that, after α-LA treatment, modification of *Gadd153/Chop* gene expression is an early event (starting from 6 hours) (Table I Supplementary data) which is preceded by modification of intracellular ROS levels, as previously demonstrated^14^. Then, starting from 24 hours, increased levels of GADD153/CHOP protein, which preceded the loss of FaO cell viability (Fig. 1A,B), were observed. Moreover, a persistent overexpression of PDI family proteins, such as PDI and Erp72 (Fig. 7C), was found, further supporting the role of ER in the induction of α-LA-mediated apoptosis. Indeed, while initially upregulation of PDI proteins has a protective function aimed to restore normal cell homeostasis, persistent ER stress causes their release from ER followed by their interaction with pro-apoptotic proteins, such as Bak, and induction of Mitochondrial Outer Membrane Permeabilization^29^. Next, we have examined the regulation of the three UPR branches which have also been reported to be involved in the adaptability to ER stress and evasion from apoptosis of cancer cells, thus allowing them to maintain malignancy. Opposite, chronic ER stress causes UPR-mediated apoptosis of cancer cells^22^. To analyze the role of the three branches of UPR in α-LA-induced apoptosis, protein expression analysis was performed in FaO and HepG2 hepatoma cells. In both cell lines, the chaperone GRP78 showed a constant sustained expression in both treated and untreated cells overtime. Under ER stress, GRP78, which originally binds to the luminal domain of the three ER transducers sensors, IRE1, PERK and ATF6, dissociates from them allowing IRE1- PERK- and ATF6-mediated UPR in order to maintain and restore ER homeostasis^22^. However, if ER stress is prolonged so that UPR is unable to cope with unfolded proteins, UPR is promoted to trigger an apoptotic response through the same signaling pathways (ATF6-, PERK- and IRE1-dependent)^22^. Accordingly, our data have shown that induction α-LA treatment led to PERK and IRE1 pathways activation which contribute to ER stress-mediated apoptosis through: i) the induction of GADD153/CHOP, ii) the activation of XBP-1 and iii) the phosphorylation of JNK. Opposite, the anti-apoptotic ATF6 pathway, which was shown to enhance tumor cell survival through activation of the Rheb-mTOR signaling pathway^49^ or by induction of GRP78 expression^25^, has resulted inhibited. Summarizing, together with our previous results^14^, the present data suggest that α-LA-induced apoptosis of hepatoma cells occurs, at least in part, by ER stress activation and is regulated via distinct UPR signaling pathways. The outline of our current hypothesis is illustrated in Fig. 8B. Induction of ER stress by α-LA results in the activation of PERK pathway which, in turn, triggers GADD153/CHOP leading to apoptosis. In addition, ER stress induces apoptosis through activation of IRE1 pathway, which acts via JNK phosphorylation and activation of XBP-1, and by inhibition of the ATF6-mediated pro-survival pathway. Our data are in agreement with those obtained from previous studies suggesting that under prolonged ER stress conditions, PERK and IRE1 signaling can trigger cell death^50^. High levels of GRP78 are usually considered to be a marker of cell survival so that this ER stress chaperone usually resulted upregulated in cancer cells compared to normal ones^51,52^. One of the major functions of GRP78 is the protection against stress-induced apoptosis by suppression of CHOP induction, which mediates the apoptotic arm of the UPR^53^. Accordingly, in our experimental condition GRP78 showed a sustained and constant expression in both treated and untreated hepatoma cells, whereas, after α-LA treatment, increasing expression levels of CHOP, overwhelm the GRP78 protective activity, ultimately leading to apoptosis. In this scenario, pro-survival GRP78 and pro-apoptotic GADD153/CHOP are key opposing actors involved in ER stress response α-LA-mediated in hepatoma cells. These findings suggests that α-LA is able to induce ER stress-mediated apoptosis in hepatoma cells as a consequence of the aggravation of a pre-existing ER stress condition related to redox imbalance, while it does not exert any toxic effect on normal hepatocytes^54^, which are likely expected to be exposed to very low levels of ER-inducing agents. On these premises, α-LA might represent a useful tool to exceed tumor cells adaptive capacity to ER stress, by inhibiting the ER stress-dependent pro-survival pathway. Nevertheless, it should be emphasized that while in some contexts the use of α-LA can be beneficial, in others it can be detrimental. Indeed, also normal cells exposed to different conditions leading to UPR, could suffer of ER stress aggravation under α-LA exposure and undergo cell death. In agreement with the latter hypothesis, it was previously shown^54^ that α-LA aggravates liver injury and promotes the growth of preneoplastic lesions in a rat model of hepatocarcinogenesis that, similarly to human HCC development, is characterized by fatty change and hepatocyte injury^55,56^. In conclusion, to the best of our knowledge, this study shows for the first time the involvement of ER stress response in α-LA-induced apoptosis in hepatoma cells. Data here reported suggest that in the presence of ER stress, α-LA acts as ER stress aggravator (ERSA)^57^. These compelling evidences suggest that α-LA might represent a useful tool to counteract chemoresistance of liver tumor cells even in association with other chemotherapeutic agents. Anyway, it is important to underline that, since α-LA is widely used in clinic as antioxidant additional studies *in vivo and in vitro* are needed to verify the potential role of α-LA as ER stress aggravator in pathological conditions characterized by ER stress, such as, fatty liver disease, insulin resistance, hepato-steatosis, nonalcoholic steatohepatitis (NASH), all disorders that increase risk of hepatocarcinogenesis^58^.

## Methods

### Cell lines

The rat hepatoma cell line, FaO, and the hepatocarcinoma cell line, HepG2, were supplied by Interlab Cell Line Collection (Servizio Biotecnologie, IST, Genova, Italy), and maintained, respectively, in Dulbecco’s medium (DMEM plus Glutamax I) (Invitrogen) and supplemented with penicillin, streptomycin and 10% heat-inactivated fetal calf-serum (FCS) (Invitrogen) in a humidified atmosphere of 5% CO_2_/95% air, at 37 °C. α-Lipoic Acid (α-LA) and Thapsigargin (TG) were purchased from Sigma (Sigma-Aldrich, Milano, Italy). α-LA, dissolved in sodium hydroxide NaOH 1 N and neutralized in medium, and TG dissolved in DMSO, were added to the culture media to the final concentrations specified in the text.

### Morphological assessment of apoptosis

Morphological assessment and detection of apoptotic cells was performed using Hoechst 33258 (Invitrogen) staining. FaO cells (2 × 10^5^ cells/well) were plated in chamber-slides (Lab-Tek Chamber, NUNC, NewYork, USA) and cultured in the presence or absence of α-LA. After treatment, the cells were fixed with 2% paraformaldehyde and stained with Hoescht 33258. Stained cells were visualized under a Leica DM2000 fluorescence microscope and images were acquired with a digital camera Leica DCF420C.

### Immunofluorescence Assay for GADD153/CHOP and GRP78

Treated FaO cells were grown on chamber slides, as described above, and were fixed with methanol and incubated with GRP78 antibody (abcam, Cambridge, UK) (1:900 in PBS-5%BSA) or with GADD153/CHOP (Santa Cruz, Biotechnology Inc., CA, USA) (1:400 in PBS-5%BSA). Following incubation with appropriate secondary antibody, the nuclei cells were stained with Hoechst 33258. Stained cells were observed using a Leica DM2000 fluorescence microscope and images were acquired with a digital camera Leica DCF420C.

### mRNA expression profile

RNA was isolated from FaO cells with TRIzol reagent (Invitrogen). For gene expression profile, 500 ng of RNA was amplified (Illumina TotalPrep Amplification Kit), labelled and hybridized on Illumina microarray (RatRef-12V1 BeadsChips, Illumina Inc. San Diego, CA, USA) including 21.791 gene specific oligonucleotide probes. The intensity files were loaded into the Illumina BeadStudio 3.0.19.0 software (Illumina Inc, San Diego, CA, USA) and BRB Assay Tools (Version 4.2.0) for quality control and gene expression analysis. The quantile normalization algorithm was applied on the dataset. Only genes, with a detection p-value <0.05 whose expression differed by at least 1.5 folds from the median in at least 20% of the arrays and characterized by 50^th^ percentile intensities greater than 300, were retained. According to these criteria, 7022 expressed transcripts out of 21791 were considered as detected. Raw Microarray data have been deposited in the Gene Expression Omnibus (GEO) website (http://www.ncbi.nlm.nih.gov/geo/info/linking.html) with Accession Number GSE59164.

### Pathway analysis

Genes were submitted to the Ingenuity Pathway analysis (IPA) analysis pipeline, to examine the bio logical network associated with the LA treatment from 6 up to 72 hours. IPA software (http://www.ingenuity.com) used manually curated database which contain information from several sources including published journal papers and gene annotation databases. Analysis of “Pathways and Function” was based on the number of genes significantly dysregulated (fold difference cut off ±2.0).

### Gene expression analysis

RNA (2 µg) was reverse transcribed using High Capacity cDNA Reverse Transcription Kit (Life Technologies). Real time PCR was performed in a 7300 Real-Time PCR System (Life Technologies). The amplification mixture contained 20 ng of cDNA, 5 µl 2X TaqMan Gene Expression PCR Master mix (Life Technologies) and 0,5 µl of specific 20X TaqMan Gene Expression Assay (Rn00565250_m1 for Hspa5/GRP78, Rn00891854-m1Pirir15/GADD34 and Rn00595314_m1 Trib3, Life Technologies). All samples were analyzed in triplicate and β-actin gene expression (Life Technologies) was used as housekeeping control for possible differences in cDNA amount. The relative gene expression was calculated according to the 2^−ΔΔCT^ method using the untreated cells as control.

### Splicing analysis of XBP1

cDNA was PCR amplified using the following primers: 5′AAACAGAGTAGCAGCACAGACTGC-3′ AND 5′CCTTCTGGGTAGACCTCTGGGAG-3′ as forward and revers primers respectively. The PCR reaction mix contained 50 ng of cDNA, 100 µM deoxynucleeosidetriphosphatases dNTPs, 1.5 mmol/L MgCl_2_, 2U of Platinum Taq DNA Polymerase (Life Technologies) and 5 pmol of each primer; the final volume was adjusted to 25 µl. PCR was carried out at 95 °C for 30 seconds, at 58 °C for 30 seconds, and 72 °C for 30 seconds in a GeneAmp PCR System 9700 (Applied Byosystems) for a total of 40 cycles: Quality analysis of PCR amplifications were assessed by loading the reaction products on ethidium bromide stained 2.4% agarose gels.

### Western Blot analysis

Both FaO and HepG2 cells were lysed in RIPA lysis buffer (1X PBS, 1% IGEPAL, 0.5% sodium deoxycholate, 0.1% SDS) plus the protease inhibitors 300 µg/ml aprotinin, 100 µg/ml sodium orthovanadate and 100 µg/ml phenylmethylsulfonylfluoride (PMSF) (Sigma) for 30 min at 4 °C. Protein Hercules, CA). Protein concentration was determined according to Bradford^59^. The densitometric analysis was performed by using ImageJ, normalized relative to their respective loading control band (actin for FaO cell proteins and Albumin for HepG2 cell proteins). Value are expressed as fold change respect to control value (1.00). For more details see Supp. Methods.

### Statistical analysis

Instant software (GraphPad Prism4, San Diego, CA) was used to analyze the data. One-way analysis of variance (ANOVA) with post hoc analysis using Tukey’s multiple comparison test was used for parametric data. The results of multiple observations were presented as the means ± S.D. of at least 3 separate experiments. A *p* value of <0.05 was considered statistically significant.

## Supplementary information

Supplementary information.

## Data Availability

All data generated and analysed during this study are included in the published article and its Supplementary information file.
