# Supplementary material for: α-Lipoic acid induces Endoplasmic Reticulum stress-mediated apoptosis in hepatoma cells
Source: Sci Rep. 2020 Apr 28;10:7139. doi: 10.1038/s41598-020-64004-5 (PMC7189383; doi:10.1038/s41598-020-64004-5)
Supplement: Supplementary file 1 — Supplementary information. [file 41598_2020_64004_MOESM1_ESM.pdf]

Supplementary Information

α-Lipoic acid induces Endoplasmic Reticulum stress-mediated apoptosis in hepatoma cells

Pibiri M[1], Sulas P[1], Camboni T[2], Leoni VP[1], Simbula G[1]*

[1]Biomedical Sciences Department, Oncology and Molecular Pathology Unit, University of Cagliari, Italy

[2]National Research Council, Institute of Biomedical Technologies, Segrate (MI), Italy

*Corresponding to: gsimbula@unica.it Biomedical Sciences Department, University of Cagliari, Cittadella Universitaria di Monserrato, S.P. 8 Monserrato-Sestu km 0.700, Blocco A 09042 Monserrato (Cagliari), Italy, Phone: +39 070-6756826

Supplementary Figure 1

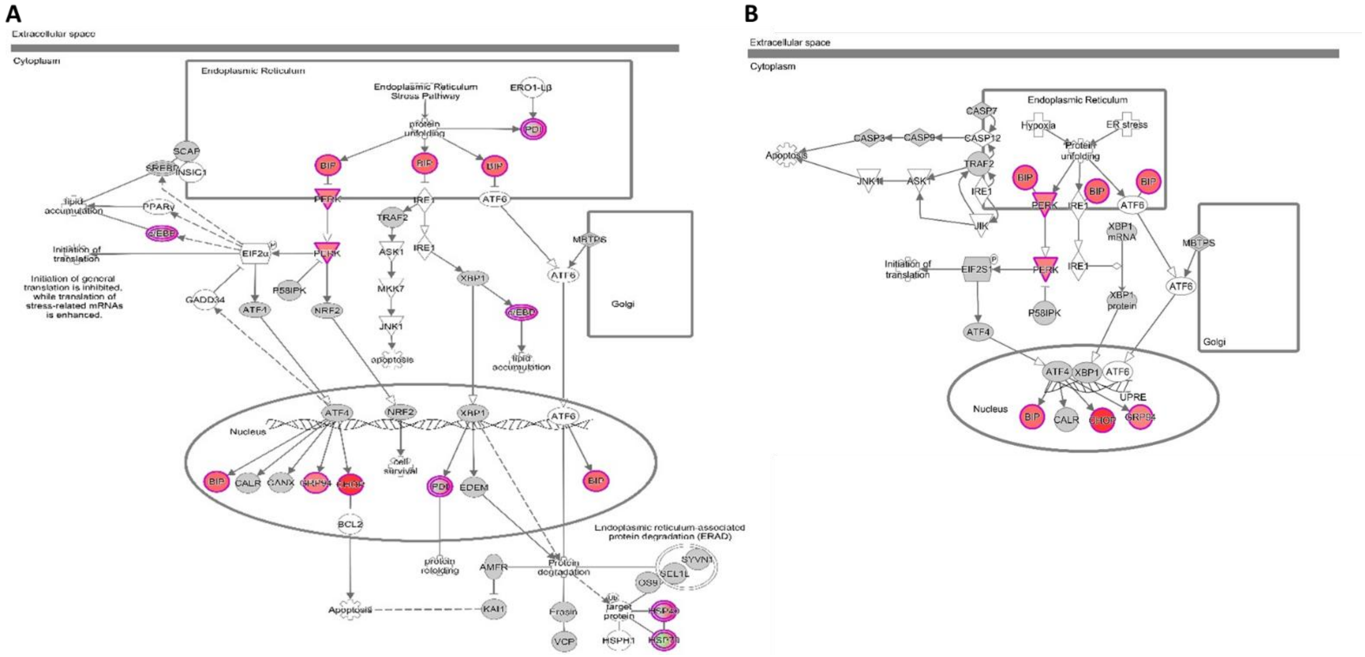

Supplementary Figure 2

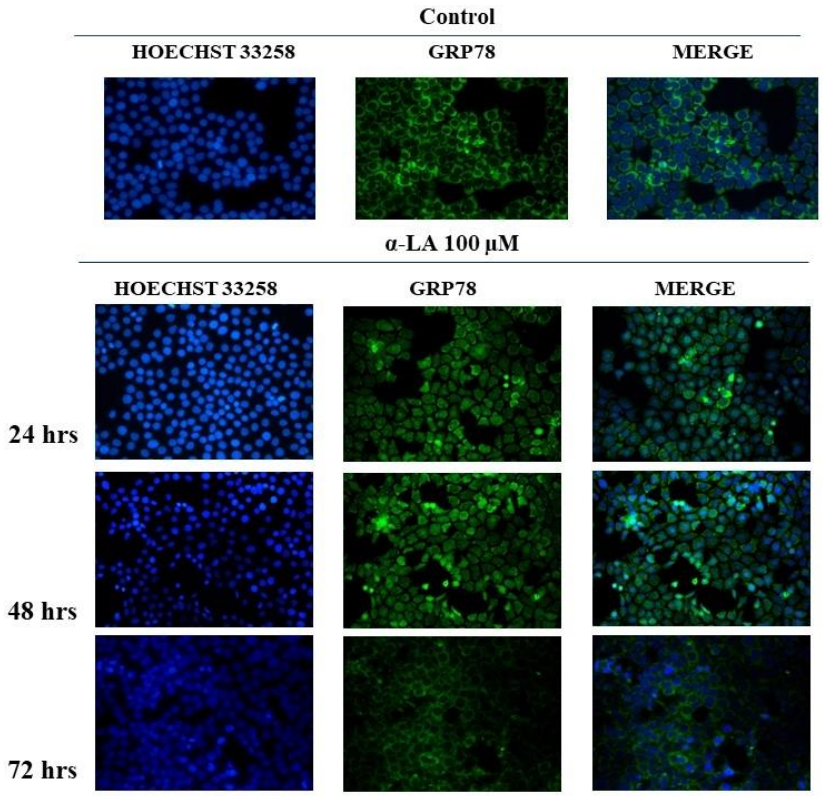

Supplementary Figure 3

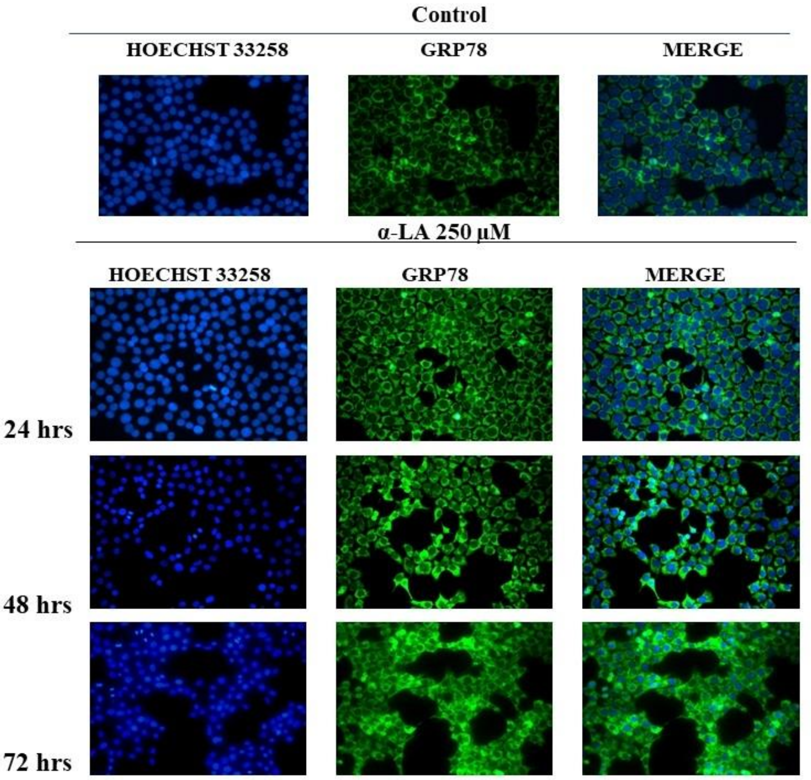

Supplementary Figure 4

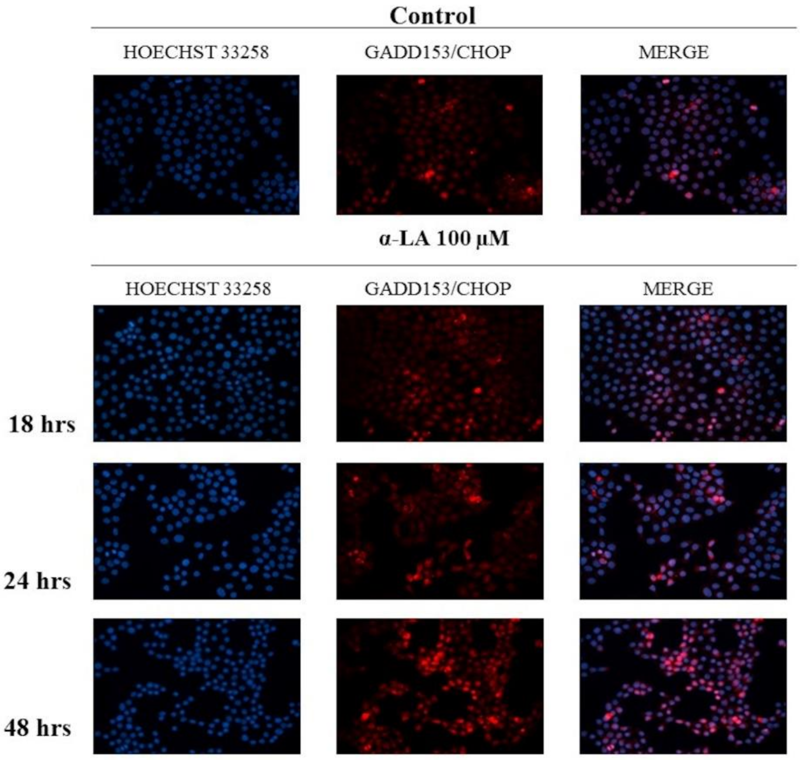

Supplementary Figure 5

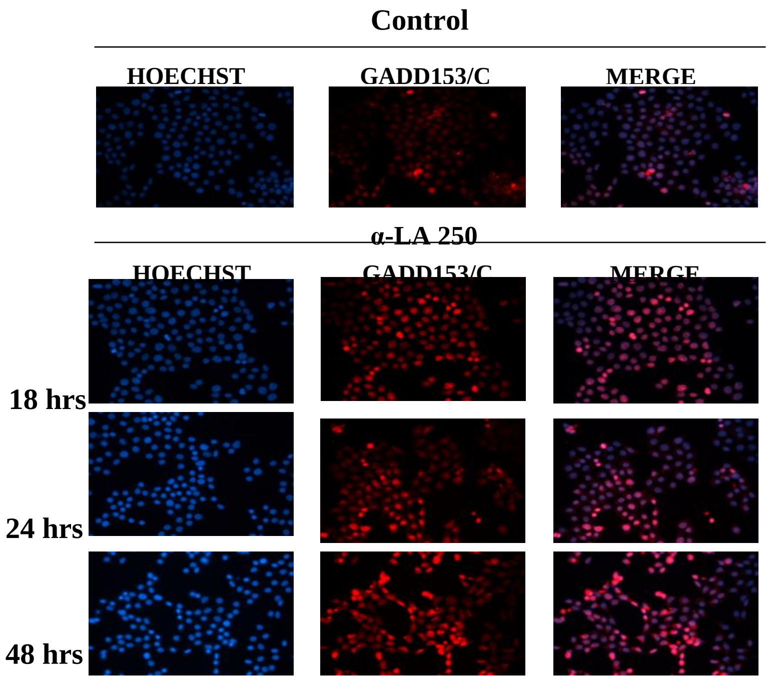

Supplementary Figure Legends

Figure 1

Gene expression profiling in α-LA -treated FaO cells (A-B) UPR and ER stress pathways after 48 hrs α-LA-treatment. Genes were coloured based on their expression (Red for up-regulated genes; Green for downregulated genes). Data were analyzed through the use of IPA (QIAGEN Inc., https://www.qiagenbioinformatics.com/products/ingenuitypathway-analysis).

Figure 2

Immunofluorescence analysis of GRP78 in rat FaO hepatoma cells. FaO cells were subjected to Hoechst 33258 (nuclear stain) or GRP78 staining after exposure to 250 uM α-LA for 24, 48 and 72 hours. Assay was performed in quadruplicate.

Figure 3

Immunofluorescence analysis of GRP78 in rat FaO hepatoma cells. FaO cells were subjected to Hoechst 33258 nuclear stain or GRP78 staining after exposure to 100 uM α-LA for 24, 48 and 72 hours. Assay was performed in quadruplicate.

Figure 4

Immunofluorescence analysis of GADD153/CHOP in rat FaO hepatoma cells. FaO cells were subjected to Hoechst 33258 (nuclear stain) or GADD153/CHOP staining after exposure to 250 uM α-LA for 24, 48 and 72 hours. Assay was performed in quadruplicate.

Figure 5

Immunofluorescence analysis of GADD153/CHOP in rat FaO hepatoma cells. FaO cells were subjected to Hoechst 33258 (nuclear stain) or GADD153/CHOP staining after exposure to 100 uM α-LA for 24, 48 and 72 hours. Assay was performed in quadruplicate.

Supplementary information Methods

Western blot: for immunoblotting analysis, equal amounts of proteins were electrophoresed on SDS 8%, 12% or 15% polyacrilamyde gels and blotted. After gel electrotransfer onto nitrocellulose membranes, to ensure equivalent protein loading and transfer in all lanes, the membranes were stained with 0.5% (w/v) Ponceau S red (ICN Biomedicals) in 1% acetic acid for 5 min. After blocking in TBS containing 0.5% Tween 20 (Sigma) (TBS-T) and 5% non-fat dry milk for 1 hour at room temperature or overnight at 4° C, membranes were washed in TBS-T and incubated with the appropriate primary antibodies diluted in blocking buffer. Whenever possible, the same membrane was used to detect the expression of different proteins. Depending on the origin of primary antibody, filters were incubated with anti-mouse, anti-goat or anti-rabbit horseradish peroxidase-conjugated IgG (Santa Cruz Biotechnology, Santa Cruz, CA). Immunoreactive bands were identified by chemiluminescence detection system, as described by the manufacturer (Supersignal Substrate, Pierce, Rockford, IL). For immunoblotting experiments the following antibodies were used: mouse monoclonal anti-actin (clone AC-40)(Sigma), anti-ATF6 (clone 70B 1413.1) (Imgenex, San Diego, CA), anti-GADD153/CHOP (Santa Cruz Biotechnology),anti-Albumin (Bethyl Laboratories Inc., USA), rabbit polyclonal anti-SAPK/JNK (Cell Signalling), rat polyclonal anti-Phospho-SAPK/JNK (Cell Signalling), anti-GRP94 (Cell Signaling Technology, Beverly MA) and GRP78/Bip (abcam, Cambridge, UK), rabbit monoclonal anti-phospho-PERK (thr980) (Cell Signaling Technology), anti-eIF2α (phospho S51) (abcam), anti-ASK1(abcam), anti-IRE1(phosphor S724)(abcam), anti-Erp72 (Cell Signalling), anti-Erp57(Cell Signalling), anti-PDI(Cell Signalling),anti-GADD34 (SantaCruz) and anti-calnexin (Cell Signalling)were used as primary antibody. Depending on the origin of primary antibody, filters were incubated with anti-mouse, anti-goat or anti-rabbit horseradish peroxidase-conjugated IgG (Santa Cruz Biotechnology, Santa Cruz, CA). Immunoreactive bands were identified by chemiluminescence detection system (Supersignal Substrate, Pierce, Rockford, IL).

Supplementary figure 6. Uncropped blots of Figure 4D

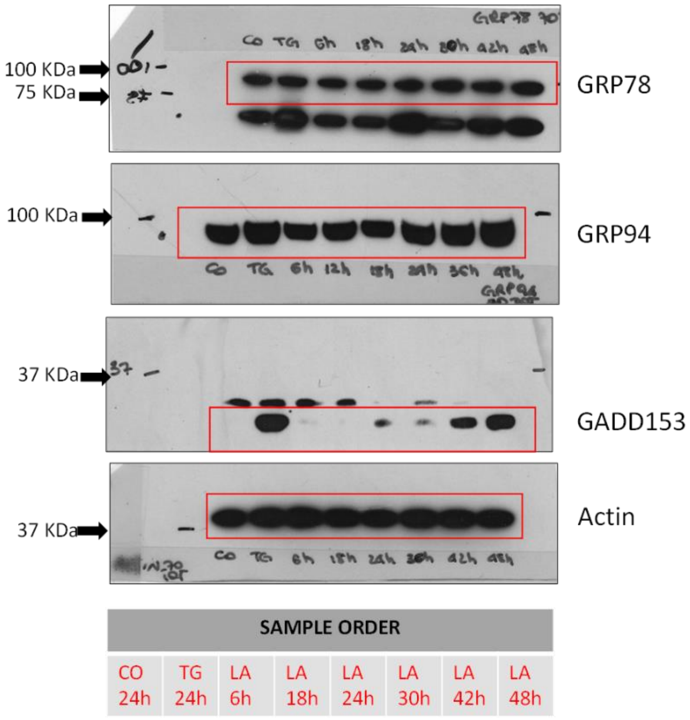

Figure 4D

Supplementary Figure 7. Uncropped Figure 7A

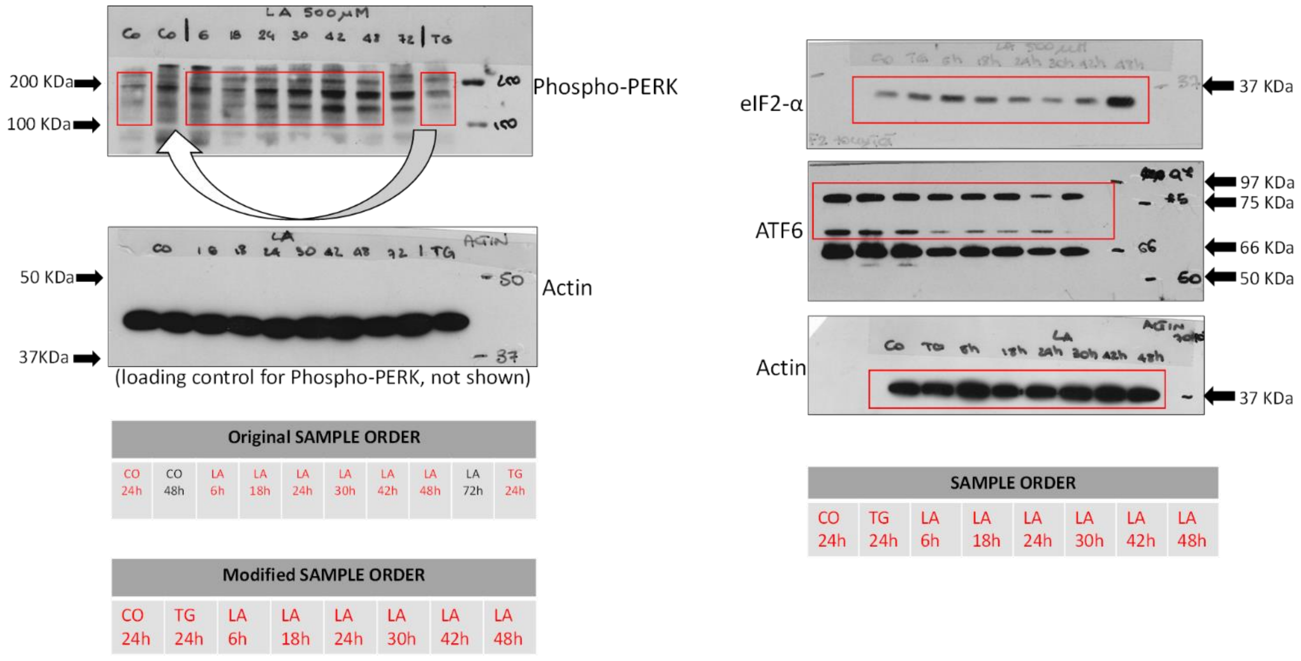

Figure 7A

Supplementary Figure 7: Uncropped figure 7B and 7C

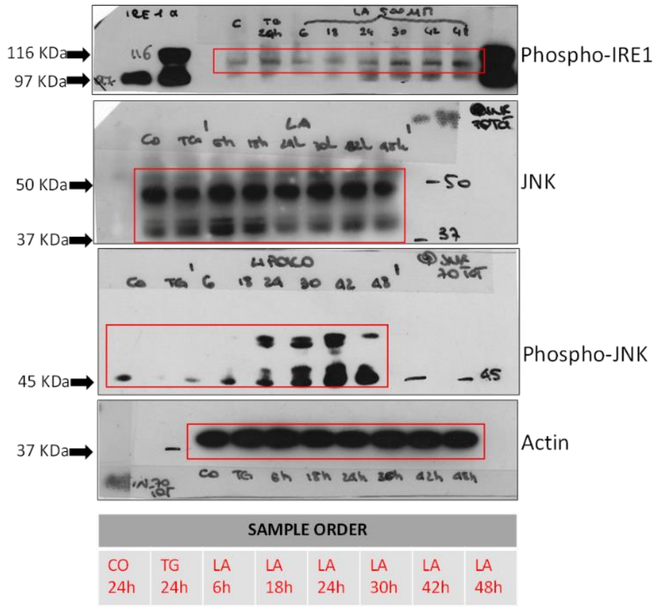

Figure 7B

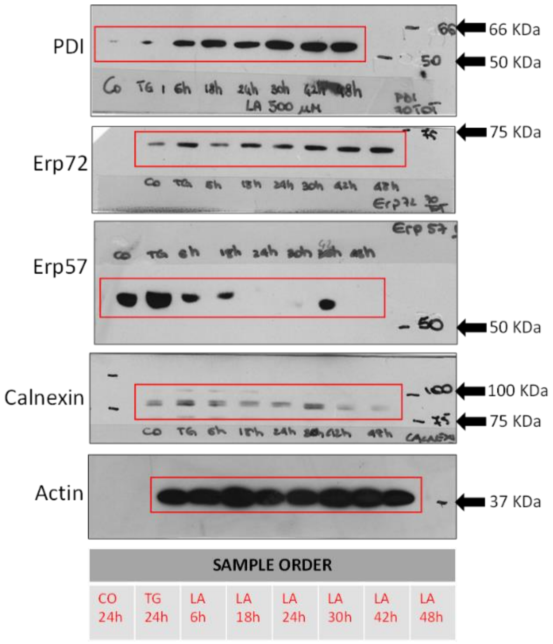

Figure 7C

Supplementary Figure 7. Uncropped Figure 7

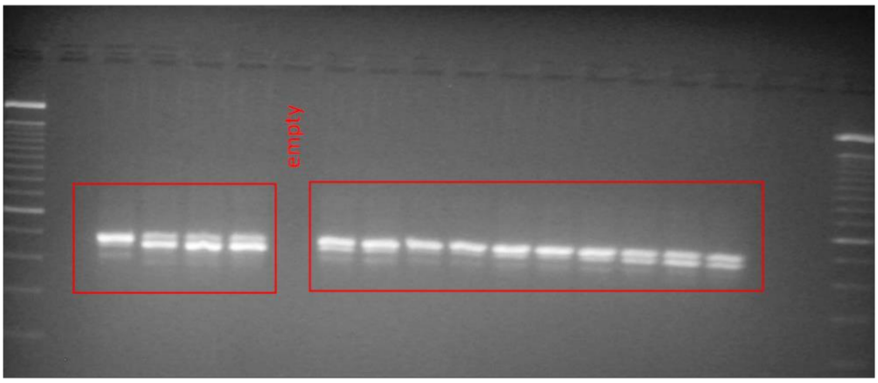

ORIGINAL SAMPLE ORDER														
CO	TG 1h	TG 3h	TG 6h	EMPTY	TG 18h	TG 24h	LA 6h	LA 12h	LA 18h	LA 24h	LA 30h	LA 36h	LA 42h	LA 48h

MODIFIED SAMPLE ORDER													
CO	TG 1h	TG 3h	TG 6h	TG 18h	TG 24h	LA 6h	LA 12h	LA 18h	LA 24h	LA 30h	LA 36h	LA 42h	LA 48h

Figure 7D

Supplementary Figure 8. Uncropped Figure 8

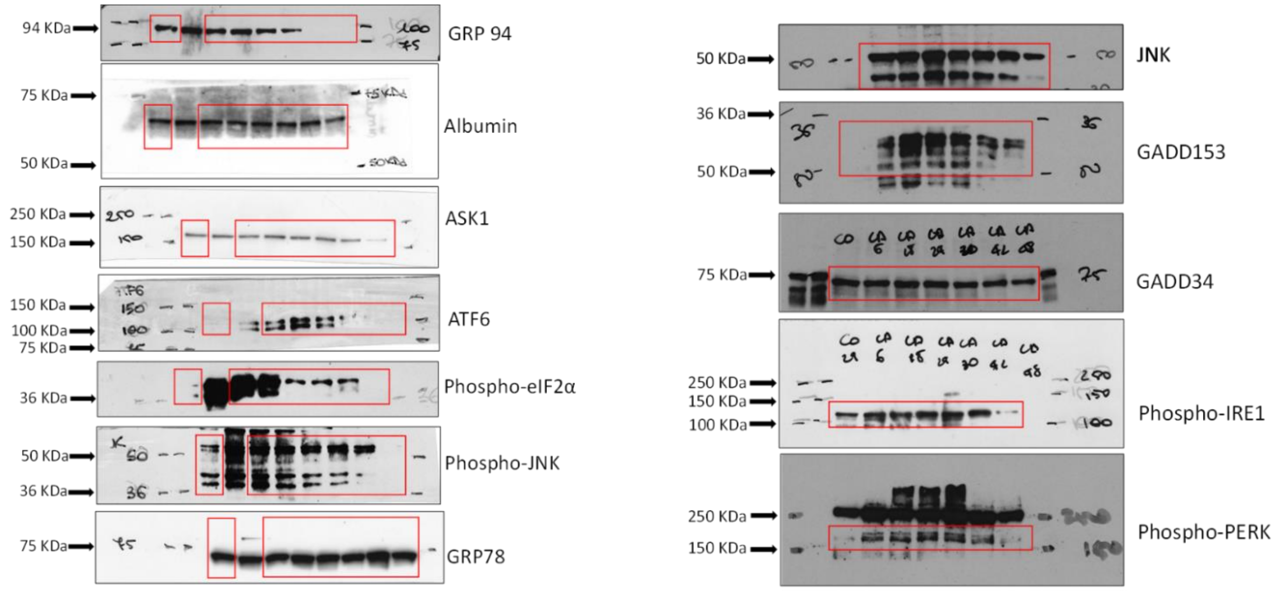

SAMPLE ORDER							
CO 24h	CO 48h	LA 6h	LA 18h	LA 24h	LA 30h	LA 42h	LA 48h

SAMPLE ORDER						
CO 24h	LA 6h	LA 18h	LA 24h	LA 30h	LA 42h	LA 48h

Figure 8A

Table I: Genes differentially expressed in each step compared to controls

UniqueID	Symbol	FC α-LA 6h	FC α-LA 18h	FC α-LA 24h	FC α-LA 30h	FC α-LA 42h	FC α-LA 48h	FC α-LA 72h
ILMN_1368089	Rrm2_mapped	-1.31	-5.56	-2.43	-3.27	-7.63	-4.16	-14.94
ILMN_1353596	Ccna2	-1.41	-4.83	-3.39	-3.54	-8.74	-6.15	-12.23
ILMN_1363828	Pck1	-2.87	-8.03	-11.73	-11.71	-9.93	-12.06	-12.07
ILMN_1375974	Cdca1_predicted	-1.27	-4.26	-3.09	-3.74	-8.2	-5.38	-11.58
ILMN_1364291	Spbc24_predicted	-1.02	-3.44	-3.2	-2.76	-5.86	-3.75	-11.07
ILMN_1359510	Ns5atp9	-1.27	-5.57	-3.94	-3.42	-6.14	-4.32	-10.74
ILMN_1371503	RGD1561694_predicted	-1.3	-5.01	-2.76	-5.22	-6.66	-4.99	-9.59
ILMN_1371599	Aldh1a4	-1.28	-7.43	-12.19	-13.46	-9.97	-13.69	-8.87
ILMN_1372675	Hmgb2	-1.21	-4.42	-3	-4.56	-6.73	-4.59	-8.81
ILMN_1358998	Aurkb	-1.46	-3.84	-2.7	-2.96	-5.64	-4.54	-8.74
ILMN_1376705	Cdca1	-1.4	-4.15	-2.93	-3.34	-6.87	-5.15	-8.63
ILMN_1359126	Prc1_predicted	-1.37	-3.64	-2.41	-2.65	-7.6	-5.12	-8.6
ILMN_1355582	Kifc1	-1.11	-3.1	-1.91	-2.58	-5.75	-4.02	-8.24
ILMN_1351744	Nusap1_predicted	-1.3	-4.94	-2.44	-4.05	-7.32	-4.95	-8.03
ILMN_1358490	Cdc2a	-1.13	-2.78	-1.64	-1.95	-6.03	-3.75	-8.01
ILMN_1351955	Hmgn2	-1.52	-5.56	-3.5	-5.48	-6.77	-6.38	-7.99
ILMN_1354271	Pole2_predicted	-1.39	-3.64	-3.48	-3.54	-6.38	-5.06	-7.89
ILMN_1366651	Fmo1	-1.56	-4.85	-5.88	-9.27	-6.38	-7.82	-7.81
ILMN_1371160	Traf4af1	-1.04	-2.16	-2.07	-2.4	-7.22	-4.72	-7.8
ILMN_1350338	Gc	-1.24	-1.8	-1.83	-2.81	-5.8	-6.45	-7.6
ILMN_1349269	Sgk	-2.2	-5.35	-4.81	-6.28	-4.75	-6.41	-7.53
ILMN_1650605	LOC497083	-1.55	-3.19	-2.69	-3	-5.04	-3.63	-7.46
ILMN_1351907	LOC498072	-1.17	-4.16	-2.77	-4.17	-5.74	-4.78	-7.45
ILMN_1355984	Tpx2_predicted	-1.42	-2.7	-2.47	-2.64	-7.39	-4.73	-7.19
ILMN_1365457	Spbc25	-1.35	-3.85	-2.39	-3.03	-5.42	-3.94	-6.97
ILMN_1366278	Ube2c_predicted	-1.44	-3.31	-2.38	-2.69	-6.83	-4.84	-6.72
ILMN_1353146	Ect2_predicted	-1.05	-2.26	-2.06	-2.22	-4.25	-3.45	-6.69
ILMN_1358066	Mcm7	-1.31	-2.64	-3.07	-3.28	-4.7	-3.2	-6.64
ILMN_1356435	Wnt4	-1.39	-3.96	-4.62	-5.52	-6.5	-6.51	-6.34
ILMN_1356153	RGD1561749_predicted	-1.24	-4.49	-2.86	-4.15	-3.66	-3.25	-6.24
ILMN_1352298	Mcm10_predicted	-1.3	-3.03	-2.95	-3.14	-4.99	-4.65	-6.19
ILMN_1373415	RGD1562246_predicted	-1.04	-3.66	-3.7	-3.59	-4.8	-3.65	-6.13
ILMN_1369124	LOC498793	-1.26	-2.61	-3.13	-4.22	-3.99	-4.49	-6.03

ILMN_1369326	Aspm_predicted	-1.28	-2.8	-2.6	-2.53	-4.88	-4.17	-6.02
ILMN_1350210	Mcm3_predicted	-1.37	-3.95	-3.85	-3.85	-4.74	-4.02	-5.94
ILMN_1358183	Kif22	-1.44	-3.87	-2.12	-3.11	-6.09	-4.58	-5.88
ILMN_1358804	Exo1_predicted	-1.14	-2.66	-2.3	-1.9	-4.89	-4.42	-5.87
ILMN_1366864	Pole_mapped	-1.17	-3.07	-3.74	-2.71	-4.4	-4.01	-5.87
ILMN_1373595	Cdc20	-1.53	-2.77	-1.82	-2	-6.19	-4.7	-5.86
ILMN_1354160	Kntc2_predicted	-1.09	-2.91	-2.14	-3.12	-5.12	-3.78	-5.86
ILMN_1363050	Bub1_predicted	1.01	-1.96	-1.69	-1.84	-4.79	-3.4	-5.84
ILMN_1353635	Aldob	-2.16	-4.88	-6.72	-10.9	-5.09	-6.89	-5.71
ILMN_1352068	Rfc5_predicted	-1.09	-2.62	-1.54	-2.38	-4.39	-3.46	-5.71
ILMN_1367740	Mt1a	1.26	-1.67	-3.61	-1.08	-7.09	-5.14	-5.69
ILMN_1365004	RGD1562047_predicted	-1.1	-2.35	-2.64	-1.96	-3.92	-2.6	-5.53
ILMN_1357068	Ube2t_predicted	-1.16	-2.39	-1.82	-1.88	-5.44	-3.69	-5.49
ILMN_1353258	Dlg7_predicted	-1.27	-2.4	-2.24	-2.68	-5.48	-3.79	-5.37
ILMN_2039661	G6pc	-1.79	-3.76	-4.55	-4.42	-4.76	-5.27	-5.34
ILMN_1353170	RGD1309107	1.06	-2.1	-1.63	-1.78	-4.97	-3.33	-5.26
ILMN_1370269	Kif20a_predicted	-1.38	-2.65	-2	-2.44	-4.93	-4.27	-5.17
ILMN_1357198	Pola2	-1.22	-3.33	-2.46	-2.45	-4.2	-3.7	-5.17
ILMN_1651164	Suv39h1_predicted	-1.14	-2.71	-1.98	-2.19	-4.98	-4.29	-5.13
ILMN_1367952	RGD1562067_predicted	-1.43	-3.8	-2.94	-3.55	-5	-3.49	-5.07
ILMN_1373537	Kntc1_predicted	-1.16	-2.72	-2.13	-1.92	-2.73	-2.55	-5.04
ILMN_1359537	Serpina6_mapped	-1.62	-1.46	-1.89	-4.14	-2.55	-4.39	-4.87
ILMN_1355685	Spag5	-1.22	-3.33	-2.78	-2.64	-4.3	-3.98	-4.84
ILMN_1354270	Racgap1_predicted	-1.21	-2.11	-1.81	-2.19	-3.71	-2.91	-4.81
ILMN_1376860	Fancd2	-1.44	-2.8	-2.83	-2.75	-4.43	-3.42	-4.79
ILMN_1356031	Mcm6	-1.2	-3.12	-2.67	-2.32	-3.47	-3.15	-4.78
ILMN_1373874	LOC682159	-1.46	-2.6	-3.31	-2.51	-3.64	-4.75	-4.77
ILMN_1374423	Ttk_predicted	-1.2	-2.24	-1.94	-2.28	-4.53	-2.91	-4.76
ILMN_1371641	LOC500916	-1.03	1.03	1.12	1.24	1.2	1.09	-4.72
ILMN_1371588	Cdca2	-1.36	-2.86	-2.06	-2.11	-4.25	-2.96	-4.71
ILMN_1361476	Kif11	-1.14	-2.59	-2.21	-2.7	-4.14	-3.36	-4.7
ILMN_1358828	Fbxo5_predicted	-1.29	-3.05	-2.17	-1.88	-3.5	-3.2	-4.69
ILMN_1363307	Ttr	-1.62	-4.32	-7.88	-7.63	-3.76	-5.8	-4.65
ILMN_1373916	Kif15	-1.11	-2.74	-2.66	-2.6	-4.15	-3.45	-4.63
ILMN_1370013	Timeless	-1.19	-2.44	-2.98	-2	-3.04	-3.44	-4.63
ILMN_1374858	Pbk_predicted	-1.09	-3.05	-1.92	-2.12	-3.43	-2.87	-4.63
ILMN_1357601	Kif4	-1.22	-2.65	-2.65	-3.21	-4.47	-3.67	-4.61
ILMN_1349419	Stmn1	-1.25	-4.32	-2.82	-3.2	-3.6	-2.94	-4.55

ILMN_2040525	Dhfr	-1.28	-3.47	-3.41	-3.64	-4.31	-4.56	-4.48
ILMN_1350922	Dnmt1	-1.25	-2.57	-2.47	-2.27	-3.52	-2.88	-4.43
ILMN_1350555	Bub1b	-1.01	-2.45	-1.58	-1.99	-4.06	-2.79	-4.43
ILMN_1357437	Kif23_predicted	-1.27	-2.61	-1.91	-1.97	-4.81	-3.11	-4.42
ILMN_1374166	Gmnn_predicted	-1.13	-2.63	-2.83	-2.35	-3.67	-3.36	-4.38
ILMN_1362603	LOC499531	-1.08	-1	1.13	1.09	1.07	1.04	-4.35
ILMN_1354888	Donson	-1.27	-2.44	-1.88	-1.81	-2.72	-2.63	-4.34
ILMN_1375658	Trip13_predicted	-1.19	-2.73	-2.08	-1.82	-3.87	-3.31	-4.32
ILMN_1367660	Rfc3	-1.11	-2.38	-2.34	-2.86	-3.74	-2.94	-4.29
ILMN_1370283	Cdca8	-1.26	-3.16	-2.12	-2.53	-4.5	-3.37	-4.27
ILMN_1366306	Rad54l_predicted	-1.05	-2.4	-2.27	-2.01	-3.3	-2.84	-4.27
ILMN_1357699	Mxd3	-1.28	-3.49	-2.36	-2.52	-3.98	-2.96	-4.26
ILMN_1371795	Mawbp	-1.7	-5.57	-5.67	-8.79	-5.13	-4.77	-4.18
ILMN_1369676	RGD1306227	-1.1	-2.95	-3.03	-3.72	-3.64	-2.85	-4.17
ILMN_1371966	Dnajc9_predicted	-1.12	-2.96	-1.83	-2.64	-3.85	-3.34	-4.16
ILMN_1358432	Ccne2_predicted	-1.03	-2.59	-2	-1.8	-3.08	-2.4	-4.16
ILMN_1359929	Rpa2	-1.05	-2.78	-2.61	-2.64	-3.44	-2.75	-4.13
ILMN_1360240	Rs21c6	1.06	-2.8	-2.91	-2.13	-4.27	-2.83	-4.12
ILMN_1367858	Tyms	-1.32	-2.94	-2.59	-2.37	-4.46	-3.09	-4.1
ILMN_1354672	RGD1561219_predicted	-1.33	-2.74	-2.49	-2.99	-4.71	-3.5	-4.08
ILMN_1362943	Pah	-1.47	-3.65	-4.28	-7.18	-3.77	-5.18	-4.06
ILMN_1355241	Hirip3	-1.2	-2.19	-1.59	-2.03	-3.93	-3.33	-4.04
ILMN_1359147	Cdca3	-1.32	-3.29	-2.68	-2.84	-4.04	-3.16	-4.03
ILMN_1650107	LOC499564	-1.21	-1.09	1.1	1.2	1.21	1.04	-4.03
ILMN_1374557	RGD1560913_predicted	1.2	-2.66	-1.92	-2.12	-3.03	-2.92	-4.02
ILMN_1353164	Fen1	-1.37	-2.65	-1.83	-2.21	-2.46	-2.2	-4.02
ILMN_1367207	LOC303471	-1.08	-3.68	-2.87	-3.43	-3.87	-2.7	-4.01
ILMN_1357646	LOC687334	-1.19	-2.15	-2.06	-2.11	-3.44	-2.23	-4
ILMN_1351339	Nup107	-1.07	-2.93	-2.17	-2.25	-4.24	-3.75	-3.96
ILMN_1368725	Anln_predicted	-1.05	-2.59	-2.12	-2.37	-3.94	-2.64	-3.94
ILMN_1370245	Solt_predicted	-1.04	-2.46	-1.91	-2.36	-2.41	-1.81	-3.93
ILMN_1364508	Cdkn3_predicted	-1.28	-2.47	-1.95	-2.64	-3.52	-3.11	-3.86
ILMN_1366392	LOC360888	-1.41	-2.21	-2.24	-2.36	-3.49	-3.02	-3.85
ILMN_1365376	Orc1l	-1.06	-2.43	-2.1	-1.63	-3.39	-3.25	-3.83
ILMN_1366131	Smc2l1_predicted	-1.13	-2.65	-2.41	-2.2	-3.31	-2.75	-3.8
ILMN_1357317	Mcm2_predicted	-1.32	-3.09	-2.41	-2.47	-2.65	-2.61	-3.78
ILMN_1359908	LOC498669	-1.11	1.02	-1.02	1.28	1.18	1.03	-3.78
ILMN_1370544	Tubb5	1.07	-2.4	-2.78	-2.05	-2.45	-2.45	-3.75

ILMN_1354129	Rfc4_predicted	-1.1	-2.01	-1.67	-1.77	-2.64	-2.39	-3.73
ILMN_1355586	Lig1	-1.31	-2.3	-2.76	-2.46	-3.16	-2.89	-3.72
ILMN_1371803	Mphosph1_predicted	-1.16	-2.33	-2.01	-2.03	-3.51	-3.17	-3.68
ILMN_1354227	Plg	-1.26	-1.85	-3.28	-3.81	-3.73	-4.41	-3.67
ILMN_1363940	Plek2_predicted	-1.17	-2.34	-2.11	-2.09	-3.96	-3.41	-3.67
ILMN_1361625	LOC361885	-1.29	-1.02	1.13	1.15	1.12	1	-3.66
ILMN_1367985	Nrp1	-1.23	-3.85	-5.01	-2.9	-3.76	-3.87	-3.64
ILMN_1364997	Pcnt1	-1.17	-2.31	-1.81	-1.91	-3.34	-2.84	-3.64
ILMN_1373022	Orc6l	-1.17	-2.42	-1.97	-1.81	-2.72	-2.57	-3.62
ILMN_1356318	Cdc42ep5_predicted	-1.26	-2.86	-4.48	-3.13	-4.52	-6.1	-3.57
ILMN_1369432	Plk1	-1.19	-2.04	-1.65	-1.7	-2.81	-2.88	-3.56
ILMN_1349449	Htra1	-1.06	-2.11	-2.3	-2.9	-2.15	-2.15	-3.56
ILMN_1364679	RGD1310778_predicted	-1.09	-1.94	-1.84	-1.46	-3.46	-2.74	-3.55
ILMN_1360655	Asf1b_predicted	-1.34	-2.7	-2.37	-2.3	-3.38	-2.77	-3.53
ILMN_1364203	Pcna	-1.26	-2.65	-1.95	-2.2	-2.61	-2.19	-3.52
ILMN_1370146	RGD1561555_predicted	-1.22	-2.61	-2.16	-1.9	-3.21	-2.44	-3.51
ILMN_1359661	Nasp	-1.18	-2.13	-1.62	-2.04	-3.29	-2.65	-3.5
ILMN_1650982	LOC501232	-1.03	-4.41	-5.33	-6.92	-4.11	-5.14	-3.48
ILMN_1353298	Sesn1_predicted	-1.45	-4.04	-4.15	-4.53	-3	-3.42	-3.48
ILMN_1371550	Tmpo	-1.25	-1.9	-1.81	-1.74	-3.32	-3.1	-3.45
ILMN_1350866	Hmmr	1.21	-1.91	-1.16	-1.58	-3.64	-2.91	-3.44
ILMN_1350177	RGD1561797_predicted	1.04	-1.62	-1.69	-1.66	-1.77	-1.63	-3.44
ILMN_1361716	Lgals2	-1.21	-2.28	-3.48	-3.9	-3.15	-3.87	-3.42
ILMN_1349617	RGD1566119_predicted	-1.35	-2.66	-1.66	-1.89	-3.8	-3.43	-3.42
ILMN_1356320	Foxm1	-1.3	-2	-1.69	-2.03	-3.06	-2.6	-3.4
ILMN_1352964	RGD1562596_predicted	-1.31	-2.18	-2.29	-1.94	-2.99	-2.72	-3.37
ILMN_1351035	H2afz	-1.11	-2.1	-1.86	-2.11	-2.73	-2.2	-3.37
ILMN_1649783	LOC680847	-1.08	-1.91	-1.83	-1.83	-2.8	-2.29	-3.36
ILMN_1357012	LOC680847	-1.08	-1.91	-1.83	-1.83	-2.8	-2.29	-3.36
ILMN_1358965	Rfc2	-1.28	-3.06	-1.99	-2.34	-3.58	-2.82	-3.33
ILMN_1363365	Mad2l1_predicted	-1.1	-2.57	-2.09	-2.06	-2.56	-2.2	-3.32
ILMN_1348835	RGD1563603_predicted	-1.36	-2.55	-1.81	-2.29	-3.01	-2.88	-3.29
ILMN_1367676	RGD1307397	1.12	-2.82	-2.42	-2.65	-3.11	-2.5	-3.26
ILMN_1350397	RGD1304693_predicted	-1.01	-2.25	-2.21	-1.87	-2.72	-2.05	-3.26
ILMN_1363607	Pask	-1.22	-2.5	-2.31	-2.26	-3	-2.73	-3.24
ILMN_1362561	LOC498378	-1.01	1.01	-1.07	1.05	1.32	1	-3.24
ILMN_1368379	Pold1	-1.13	-1.69	-2.08	-1.58	-3.37	-2.79	-3.22
ILMN_1365871	Id1	1.53	-1.04	1.14	-1.13	-1.06	-1.15	-3.19

ILMN_1351436	LOC501231	-1.07	-2.8	-3.35	-4.21	-3.97	-4.43	-3.18
ILMN_1354409	Ngef_predicted	-1.57	-2.38	-3.44	-3.71	-3.69	-4.11	-3.17
ILMN_1360929	LOC299625	-1.27	-2.01	-3.39	-1.99	-3.24	-3.28	-3.16
ILMN_1367978	Cdc6_predicted	1.03	-2.02	-1.81	-1.8	-2.73	-2.33	-3.15
ILMN_1374606	LOC497682	1.05	-1.42	-1.56	-1.21	-2.92	-2.29	-3.14
ILMN_1360693	Serpina1	-1.13	-1.85	-2.02	-2.53	-2.46	-2.9	-3.12
ILMN_1356468	LOC685909	-1.23	-2.88	-3.85	-4.16	-2.73	-2.57	-3.11
ILMN_1365229	Siva_predicted	-1.03	-2.76	-2.69	-2.13	-1.94	-1.91	-3.11
ILMN_1371469	LOC501179	-1.61	-2.03	-2.12	-2.12	-3.81	-3.23	-3.1
ILMN_1371176	Prkcn	-1.29	-2.09	-1.17	-2.64	-2.96	-2.89	-3.1
ILMN_1356833	LOC499501	-1.1	-2.32	-1.94	-1.92	-2.66	-2.1	-3.09
ILMN_1353918	Lgr5	1.68	-1.58	-1.78	-2.15	-2.95	-3.37	-3.07
ILMN_1374411	RGD1563786_predicted	-1.55	-3.54	-2.27	-2.51	-4.57	-2.87	-3.06
ILMN_1365707	LOC498799	1.01	1.04	1.08	1.19	-1.01	-1.04	-3.06
ILMN_1359384	RGD1562949_predicted	-1.26	-1.99	-2.22	-1.74	-2.36	-2.1	-3.04
ILMN_1349902	LOC312299	-1.1	-1.83	-1.54	-1.62	-2.15	-1.72	-3.04
ILMN_1374754	Tubg1	-1.03	-1.79	-1.25	-1.7	-2.18	-2.09	-3.02
ILMN_1364755	Snrpa	-1.01	-1.24	-1.06	-1.15	-1.68	-1.81	-3.02
ILMN_1350896	Gstm2	1.05	-1.99	-1.73	-2.25	-2.55	-3.81	-3.01
ILMN_1359053	LOC498212	-1.34	-2.48	-2.2	-2.32	-2.67	-2.66	-3.01
ILMN_1350958	LOC498644	-1.17	1.06	1.71	1.22	1.32	-1.58	-3
ILMN_1361017	LOC500398	-1.11	1.21	2.01	1.5	1.12	-1.27	-3
ILMN_1370740	E2f8	-1.35	-2.17	-2.25	-2.06	-2.72	-2.18	-2.98
ILMN_1357590	Ascl2	-1.55	-3.02	-3.25	-3.22	-3.43	-3.81	-2.97
ILMN_1365898	Traip_predicted	-1.1	-2.2	-1.84	-1.69	-3.3	-2.66	-2.96
ILMN_1365716	Gstm1	-1.15	-3.09	-2.46	-3.54	-2.97	-2.93	-2.95
ILMN_1358106	Pxmp2	-1.7	-3.28	-3.73	-3.23	-3.29	-3.59	-2.93
ILMN_1370632	RGD1562646_predicted	-1.05	-1.66	-1.9	-1.94	-2.64	-2.58	-2.93
ILMN_1352772	Tubb2c	-1.23	-2.81	-2.29	-2.23	-3.27	-3.15	-2.91
ILMN_1363543	Stk6	-1.45	-2.23	-1.36	-1.74	-3.44	-2.45	-2.91
ILMN_1357840	Hat1	-1.19	-2.46	-2.06	-2.05	-2.79	-2.75	-2.9
ILMN_1361765	Iqgap3_predicted	-1.16	-2.38	-2.09	-2.02	-2.69	-2.61	-2.9
ILMN_1366671	RGD1307465	-1.08	-2.75	-1.83	-2.48	-2.7	-2.46	-2.9
ILMN_1373469	RGD1565583_predicted	-1.37	-2.57	-1.93	-2.33	-2.58	-2.5	-2.89
ILMN_1371709	LOC681932	-1.27	-2.07	-1.47	-1.57	-3.15	-2.81	-2.87
ILMN_1364275	Mcm8_predicted	1.07	-1.53	-1.44	-1.52	-2.34	-2.01	-2.86
ILMN_1364165	Incenp_predicted	-1.33	-2.3	-2.17	-1.77	-2.53	-2.09	-2.82
ILMN_1374242	Pzp	-1.2	-1.42	-2.23	-2.25	-3.63	-5.6	-2.81

ILMN_1376370	Kif2c	-1.32	-2.58	-1.82	-2.16	-2.94	-2.86	-2.79
ILMN_1370970	Cep55	-1.02	-1.97	-1.49	-1.72	-2.9	-2.55	-2.78
ILMN_1376349	Vrk1	1.01	-1.91	-1.38	-1.91	-2.61	-2.1	-2.76
ILMN_1376839	Obfc1	-1.18	-1.97	-1.7	-2.25	-2.22	-2.22	-2.74
ILMN_1350755	Dio1	-1.03	-1.98	-2.36	-2.72	-2.96	-3.07	-2.72
ILMN_1369773	LOC499880	-1.61	-2.39	-2.99	-3.75	-2.72	-2.76	-2.72
ILMN_1352509	RGD1305288	-1.27	-2.31	-1.59	-2.51	-2.25	-2.7	-2.72
ILMN_1357973	LOC500829	-1.06	1.17	1.99	1.49	1.27	-1.19	-2.71
ILMN_1352762	Ifitm3	-1.32	-1.27	-2.19	-1.93	-3.47	-3.66	-2.7
ILMN_1357474	Ahcy	-1.16	-2.01	-2.41	-2.65	-2.61	-2.35	-2.69
ILMN_1352199	Hdc	-1.83	-1.01	-1.3	-1.54	-1.86	-2.35	-2.67
ILMN_1651155	LOC685579	1.17	-2.31	-1.65	-1.78	-2.48	-2.33	-2.67
ILMN_1371972	RGD1311451_predicted	1.03	-1.83	-1.57	-2.4	-2.64	-2.19	-2.67
ILMN_1352626	LOC499180	-1.18	-2.24	-1.81	-1.79	-2.03	-1.86	-2.67
ILMN_1353627	RGD1564956_predicted	-1.48	-4.08	-3.15	-4.13	-2.61	-2.71	-2.66
ILMN_1356467	Mki67_predicted	-1.18	-2.02	-1.65	-1.54	-2.22	-2.47	-2.66
ILMN_1372884	Rgn	-1.46	-2.95	-3.52	-3.7	-2.66	-2.51	-2.64
ILMN_1371788	Tcf19	-1.24	-2.42	-2.15	-2.13	-2.22	-2.05	-2.64
ILMN_1372669	Nucks	-1.35	-2.06	-1.72	-1.89	-2.45	-2.31	-2.63
ILMN_1365245	RGD1560584_predicted	-1.13	-2.07	-2.04	-2.39	-2.34	-2.06	-2.63
ILMN_1366055	LOC287111	-1.1	-2.04	-1.72	-1.7	-2.65	-2.26	-2.62
ILMN_1349244	Ptma	-1.27	-2.62	-1.63	-2.31	-2.94	-2.62	-2.61
ILMN_1373878	Lsm3_predicted	-1	-1.96	-2.39	-1.91	-2.55	-2.21	-2.6
ILMN_1359099	Mastl_predicted	-1.1	-2.13	-2.03	-2.36	-2.28	-2	-2.6
ILMN_2038974	Hao1_mapped	-1.73	-2.93	-2.58	-2.9	-2.62	-2.96	-2.59
ILMN_1361161	Rnaseh2a	-1.01	-1.63	-1.45	-1.45	-1.92	-2.11	-2.59
ILMN_1371361	RGD1306721	-1.2	-1.46	-1.15	-1.3	-2.15	-1.79	-2.59
ILMN_1366742	Tm4sf4	-1.04	-2.56	-2.34	-4.01	-2.02	-2.21	-2.58
ILMN_1349333	Nsun6_predicted	-1.12	-2.12	-2.09	-2.41	-2.67	-1.84	-2.58
ILMN_1354912	Grb7	1.01	-2.09	-2.46	-3.03	-2.04	-1.76	-2.58
ILMN_1354651	Serpina10	-1.43	-1.96	-2.36	-3.02	-2.99	-3.58	-2.57
ILMN_1353096	Troap_predicted	-1.33	-1.74	-1.65	-1.75	-3.05	-2.6	-2.57
ILMN_1370037	Troap_predicted	-1.33	-1.74	-1.65	-1.75	-3.05	-2.6	-2.57
ILMN_1650104	Gulo	-1.29	-2.41	-2.27	-3.75	-2.15	-2.3	-2.57
ILMN_1364622	Cyp2b3	-1.43	-1.96	-2.25	-3.32	-2.76	-4.55	-2.56
ILMN_1364259	Anxa13_predicted	-1.18	-1.12	-1.71	-2.6	-1.73	-2.66	-2.56
ILMN_1371171	RGD1564921_predicted	1.01	-2.25	-2.02	-1.64	-2.38	-2.25	-2.56
ILMN_1358531	Tubb6	-1	-2.14	-1.55	-1.36	-1.95	-2.05	-2.56

ILMN_1362345	Smc4l1	-1.1	-1.61	-1.43	-1.34	-2.28	-2.28	-2.55
ILMN_1372915	RGD1305854_predicted	1.1	-1.32	-1.02	-1.24	-2.08	-1.97	-2.55
ILMN_1362428	MGC114417	-1.28	-2.23	-1.8	-2.31	-2.3	-2.1	-2.54
ILMN_1353343	Pkmyt1_predicted	-1.26	-1.68	-1.67	-1.51	-1.94	-1.81	-2.54
ILMN_1364541	LOC363333	-1.32	-1.84	-1.82	-1.62	-2.41	-2.08	-2.53
ILMN_1357050	RGD1559962_predicted	-1.34	-2.11	-1.9	-2.53	-2.7	-1.92	-2.49
ILMN_1363662	Plk4_predicted	1.13	-1.56	-1.57	-1.24	-1.99	-1.9	-2.48
ILMN_1370114	Chek2	-1.12	-1.66	-1.57	-1.91	-2.19	-1.89	-2.48
ILMN_1354821	RGD1307201_predicted	1.02	-1.78	-1.3	-1.7	-2.09	-1.77	-2.48
ILMN_1650433	LOC360690	1.03	-1.29	1.05	1.08	1.09	-1.19	-2.48
ILMN_1366289	Chp	-1.53	-5.03	-3.14	-4	-2.46	-2.69	-2.47
ILMN_1357712	Spr	-1.35	-2.73	-2.65	-2.14	-2.92	-3.01	-2.45
ILMN_1350574	Rad18_predicted	-1.02	-1.93	-1.69	-1.75	-1.72	-1.39	-2.45
ILMN_1350621	RGD1564930_predicted	-1.95	-2.24	-2.86	-2.09	-2.41	-2.86	-2.44
ILMN_1353990	Bok	1.1	-1.23	-1.32	-1.65	-2.11	-2.02	-2.44
ILMN_1366882	LOC360258	-1.1	-1.96	-1.41	-1.89	-2.16	-1.85	-2.44
ILMN_1373748	Lama2_predicted	-1.01	-2.04	-2.66	-2.73	-2.32	-2.47	-2.43
ILMN_1355020	Prim2	-1.16	-2.43	-1.99	-1.94	-2.28	-2.06	-2.43
ILMN_1365869	Msh2	-1.08	-2	-2.04	-2.38	-2.24	-2.32	-2.42
ILMN_1356008	RGD1309522	-1.19	-1.77	-1.45	-1.89	-2.45	-2.18	-2.41
ILMN_1650492	LOC499798	-1.27	-2.16	-1.97	-2.1	-1.95	-1.84	-2.41
ILMN_1356013	RGD1565672_predicted	-1.22	-1.79	-1.52	-1.81	-1.94	-1.76	-2.41
ILMN_1353345	Prim1	-1.08	-1.91	-1.32	-1.31	-1.93	-1.65	-2.41
ILMN_1376442	Nedd9	-2.21	-3.11	-3.17	-3.57	-2.59	-3.18	-2.38
ILMN_1361497	Blm_predicted	-1	-1.57	-1.76	-1.54	-2.5	-1.84	-2.38
ILMN_1367184	Lipc	-1.15	-1.21	-1.57	-1.81	-1.54	-2.88	-2.36
ILMN_1365220	LOC498265	-1.07	-1.96	-1.69	-1.76	-2.27	-2.06	-2.36
ILMN_1371797	Itih1_predicted	-1.33	-1.85	-2.45	-2.15	-2.71	-2.57	-2.35
ILMN_1374247	Luzp5_predicted	1.1	-2.12	-1.81	-2.29	-2.17	-1.94	-2.35
ILMN_1356336	RGD1306908	-1.17	-1.7	-1.67	-1.82	-2.3	-1.9	-2.35
ILMN_1364301	LOC500495	-1.26	1.03	1.16	1.3	1.2	-1.14	-2.35
ILMN_2038852	Mcm5_predicted	-1.26	-2.01	-1.72	-1.9	-1.96	-2.35	-2.34
ILMN_2040160	Mcm5_predicted	-1.26	-2.01	-1.72	-1.9	-1.96	-2.35	-2.34
ILMN_2039440	LOC367214	-1.17	-2.51	-2.06	-2.36	-2.36	-2.31	-2.34
ILMN_1352254	LOC499854	1.17	-1.07	-1.05	1.25	1.1	-1.14	-2.34
ILMN_1350540	LOC499372	-1.07	-1.08	-1.11	1.06	1.13	-1.11	-2.34
ILMN_1356523	Nt5e	1.08	-2.71	-2.99	-3.34	-2.44	-2.89	-2.32
ILMN_1373539	Slc35d2_predicted	-1.75	-2.47	-2.9	-3.61	-2.97	-2.77	-2.32

ILMN_1350030	Cideb_predicted	-1.13	-2.56	-2.63	-2.82	-1.77	-2.52	-2.3
ILMN_1369507	RGD1562563_predicted	-1.38	-1.6	-1.71	-1.94	-1.98	-2.01	-2.3
ILMN_1362286	Itpr1	-1.59	-2.03	-2.37	-2.73	-2.95	-3.01	-2.29
ILMN_1360105	Hspa14	-1.09	-1.72	-1.44	-1.62	-2.1	-2.26	-2.29
ILMN_1369064	Uhrf1_mapped	-1.23	-1.73	-1.59	-1.55	-2.12	-2.1	-2.29
ILMN_1360983	RGD1563164_predicted	-1.45	-2.39	-1.73	-1.79	-1.77	-1.14	-2.29
ILMN_1348866	Qdpr	-1.13	-2.61	-1.34	-2.02	-2.22	-2.13	-2.28
ILMN_1650758	Ugt2b4	-1.06	-2.47	-3	-3.83	-4.29	-4.03	-2.27
ILMN_1348787	Ugt2b4	-1.06	-2.47	-3	-3.83	-4.29	-4.03	-2.27
ILMN_1354853	RGD1306568	-1.76	-3.04	-2.77	-4.45	-2.74	-2.75	-2.27
ILMN_1369063	LOC497722	-1.13	-1.66	-1.28	-1.59	-2.23	-2.04	-2.27
ILMN_1369717	LOC682323	-1.19	-2	-1.38	-2.2	-1.62	-1.56	-2.27
ILMN_1364944	LOC498745	1.13	-1.07	-1	1.21	1.14	1.03	-2.27
ILMN_1650907	LOC680876	-1.05	-1.52	-1.2	-1.39	-2.04	-1.92	-2.26
ILMN_1357320	Aldh7a1	-1.11	-1.69	-1.29	-1.77	-1.78	-1.89	-2.26
ILMN_1357559	Tube1_predicted	-1.24	-1.76	-1.63	-1.74	-2.04	-1.83	-2.26
ILMN_1365344	LOC498889	-1.11	-1.71	-1.87	-2	-2.32	-2.04	-2.25
ILMN_1357931	Pbx3_predicted	-1.27	-1.45	-1.33	-1.55	-1.64	-1.73	-2.25
ILMN_1373855	Zfp289	-1.3	-1.72	-2.12	-2.4	-2.48	-2.6	-2.24
ILMN_1357611	Cutc_predicted	1.02	-2.45	-2.22	-2.73	-2.59	-2.56	-2.24
ILMN_1352992	Smc1l1	-1.12	-1.95	-2.11	-2.21	-2.27	-2.27	-2.24
ILMN_1374127	RGD1563348_predicted	1.68	-1.15	-1.51	-1.31	-1.55	-1.81	-2.24
ILMN_1650657	LOC500788	1.06	-1.19	-1.12	1.06	-1.07	-1.36	-2.24
ILMN_1360341	LOC298442	-1.25	-2.01	-1.91	-1.94	-1.57	-2.28	-2.23
ILMN_1352528	RGD1309007_predicted	-1.08	-1.67	-1.46	-1.8	-2.44	-2.22	-2.23
ILMN_1355010	Gemin6	-1.04	-1.8	-2.23	-1.46	-2.3	-2.2	-2.23
ILMN_1361143	Suv420h2_predicted	-1.25	-1.39	-1.08	-1.52	-2.52	-2.11	-2.23
ILMN_1368648	Cbx5_predicted	-1.08	-2.19	-2.03	-2.08	-2.24	-2.28	-2.22
ILMN_1375320	Rbp1	-1.12	-1.45	-2.05	-1.68	-1.51	-2.05	-2.21
ILMN_1374210	RGD1311361	-1.06	-1.46	-1.48	-1.29	-1.6	-1.51	-2.21
ILMN_1352659	Fadd	-1.15	-1.46	-1.41	-1.72	-2.15	-2.26	-2.2
ILMN_1362292	RGD1311747_predicted	-1.03	-1.88	-1.71	-1.58	-1.87	-1.99	-2.2
ILMN_1370132	Ppil1	-1.15	-1.89	-1.43	-1.65	-2.01	-1.89	-2.2
ILMN_1373168	LOC501553	1.04	1.05	1.61	1.13	1.08	-1.24	-2.19
ILMN_1369723	RGD1359349	1.09	-1.67	-1.98	-2.83	-1.77	-2.27	-2.18
ILMN_1650532	Mycn	-1.31	-1.78	-1.94	-2.02	-1.82	-2.23	-2.18
ILMN_1349466	Plp2_mapped	-1	-1.56	-1.47	-1.42	-1.92	-1.9	-2.18
ILMN_1349515	Nkd1_predicted	1.16	-2.3	-2.14	-2.14	-2.13	-2.73	-2.17

ILMN_1376623	Cyp2c23	-1.14	-1.65	-1.45	-2.4	-1.82	-2	-2.17
ILMN_1354125	RGD1565588_predicted	-1.45	-3.6	-2.38	-3.15	-2.37	-2.15	-2.16
ILMN_1365885	Mmp14	-1.36	-1.15	-1.46	-1.28	-1.93	-1.99	-2.16
ILMN_1365198	Bard1	-1.28	-1.92	-1.9	-1.73	-2.03	-1.87	-2.16
ILMN_1368471	RT1-N3	1.08	-1.71	-1.15	-1.79	-1.68	-1.78	-2.16
ILMN_1358384	Nxt1_predicted	-1.02	-1.19	-1.47	-1.05	-1.81	-1.61	-2.16
ILMN_1365602	Ing1l_predicted	-1.17	-1.41	-1.34	-1.16	-2.04	-1.89	-2.15
ILMN_1356089	LOC499933	-1.17	-1.77	-1.4	-1.47	-1.79	-1.79	-2.15
ILMN_1650876	LOC363174	1.13	-1.59	-1.17	-1.6	-1.99	-1.71	-2.15
ILMN_1358017	Ugt2b3	-1.06	-1.77	-2.27	-2.96	-2.97	-3.33	-2.14
ILMN_1371262	LOC296724	-1.09	-2.23	-1.23	-2.08	-2.71	-2.2	-2.14
ILMN_1360217	RGD1564866_predicted	-1.15	-2.25	-2.02	-2.44	-2.43	-1.74	-2.14
ILMN_1363859	Hpx	-1.12	-1.06	-1.29	-1.46	-1.21	-1.55	-2.14
ILMN_1375002	LOC313840	-1.17	-2.21	-2	-2.35	-2.76	-2.95	-2.13
ILMN_1650395	Usp1	1.04	-1.71	-1.55	-1.58	-1.85	-1.55	-2.13
ILMN_1356247	RGD1564894_predicted	-1.34	-2.56	-2.71	-3.37	-2.65	-2.91	-2.12
ILMN_1355709	Rcbtb2	-1.11	-1.52	-1.7	-2.29	-2.16	-2.02	-2.12
ILMN_1650132	RGD1563943_predicted	-1.11	-1.77	-1.55	-1.47	-1.83	-1.75	-2.12
ILMN_1650329	Mbd6_predicted	-1.39	-1.26	-1.62	-1.62	-1.94	-1.7	-2.12
ILMN_1376245	Ell3	1.16	1.25	-1.15	1.25	-1.54	-1.58	-2.12
ILMN_1374674	LOC362882	-1.2	-1.36	-1.13	-1.8	-2.09	-2.33	-2.11
ILMN_1362189	Klf10	-1.28	-1.81	-1.65	-1.54	-2.12	-2.27	-2.11
ILMN_1362185	Hsd17b13	-1.12	-1.35	-1.65	-2.87	-1.74	-2.2	-2.11
ILMN_1351586	Nup205_predicted	1.08	-1.27	-1.38	-1.09	-2.11	-1.97	-2.11
ILMN_1357608	RGD1311868	-1.07	-1.82	-1.57	-1.78	-1.85	-1.62	-2.11
ILMN_1365562	LOC500974	-1.45	-1.34	-1.32	-1.56	-1.93	-1.44	-2.11
ILMN_1360845	Lsm4_predicted	-1.04	-2.27	-2.08	-1.77	-2.31	-1.9	-2.1
ILMN_1349445	Exosc2_predicted	-1.16	-1.52	1.15	-1.11	-2.07	-1.89	-2.1
ILMN_1374586	RGD1560967_predicted	-1.08	-1.31	-1.19	-1.29	-1.88	-1.81	-2.09
ILMN_1368325	Aaas_predicted	1.03	-1.59	-1.34	-1.66	-2	-2.14	-2.07
ILMN_1368548	Exosc8_predicted	-1.1	-1.58	-1.39	-1.47	-2.09	-1.87	-2.07
ILMN_1354033	Exosc8_predicted	-1.1	-1.58	-1.39	-1.47	-2.09	-1.87	-2.07
ILMN_1367133	Ppif	1.12	-1.73	-1.2	-1.5	-2.12	-1.8	-2.07
ILMN_1373246	MGC116373	-1.19	-1.74	-1.7	-1.84	-1.72	-1.79	-2.07
ILMN_1371186	Pfn1	-1.07	-1.58	-1.29	-1.3	-1.56	-1.64	-2.07
ILMN_1350758	Slc25a21	-1.18	-1.73	-1.9	-2.22	-1.9	-2.19	-2.06
ILMN_1366898	Irf2bp1_predicted	-1.34	1.03	-1.27	1.09	-1.99	-2.06	-2.06
ILMN_1349312	LOC499481	-1.02	-1.01	1	-1.01	-1.08	-1.28	-2.06

ILMN_1360864	Parp1	-1.16	-1.41	-1.21	-1.33	-2.15	-2.14	-2.05
ILMN_1373131	Nup35	-1.27	-1.78	-1.54	-1.54	-1.46	-1.53	-2.05
ILMN_1363701	Espl1_predicted	-1.33	-1.24	-1.36	-1.16	-1.63	-1.4	-2.05
ILMN_1355207	Lztr2	-1.73	-2.27	-2.5	-2.93	-2.08	-2.09	-2.04
ILMN_1361008	LOC687799	-1.14	-2.29	-2.46	-2.92	-2.01	-1.97	-2.04
ILMN_1369196	RGD1566094_predicted	1.06	-1.36	-1.22	-1.81	-1.58	-1.82	-2.04
ILMN_1355778	LOC366505	1.01	-1.7	-1.64	-1.68	-2.02	-1.76	-2.04
ILMN_1361140	RGD1310784_predicted	1.08	-1.34	-1.08	-1.28	-1.94	-1.58	-2.04
ILMN_1367544	Ard1_predicted	-1.03	-1.47	-1.45	-1.58	-2.77	-3.01	-2.03
ILMN_2039387	Mcm4	1.03	-2.02	-1.64	-1.72	-1.8	-1.9	-2.03
ILMN_1350185	Raph1_predicted	1.04	-1.36	-1.29	-1.51	-1.39	-1.85	-2.03
ILMN_1364841	Mthfd1l_predicted	1	-1.18	-1.45	-1.13	-1.62	-1.66	-2.03
ILMN_1363141	RGD1305497_predicted	1.02	-1.85	-1.78	-1.74	-2	-1.55	-2.03
ILMN_1374623	Mbl2	-1.05	-2.69	-2.03	-2.62	-2.08	-2.29	-2.02
ILMN_1349646	RGD1311783_predicted	-1.39	-2.56	-1.95	-2.28	-2.2	-2.23	-2.02
ILMN_1360606	LOC498765	-1.19	-1.87	-1.5	-1.67	-1.92	-1.9	-2.02
ILMN_1364749	LOC501746	-1.13	-1.63	-1.95	-2.08	-1.9	-1.87	-2.02
ILMN_2040825	Pms2_predicted	-1.11	-1.49	-1.55	-1.36	-1.95	-1.83	-2.02
ILMN_1360916	Pms2_predicted	-1.11	-1.49	-1.55	-1.36	-1.95	-1.83	-2.02
ILMN_1367665	Zfyve21_predicted	-1.4	-2.07	-1.98	-1.63	-1.71	-1.81	-2.02
ILMN_1358205	RGD1560975_predicted	1.11	-2.4	-1.86	-1.47	-2.06	-2.1	-2.01
ILMN_1364553	Melk_predicted	1	-1.84	-1.67	-1.63	-2.01	-1.9	-2.01
ILMN_1369426	Car8	-1.29	-1.1	-1.28	-1.78	-1.8	-1.89	-2.01
ILMN_1355147	Fanca_predicted	-1.23	-1.52	-1.56	-1.5	-1.93	-1.86	-2.01
ILMN_1362748	RGD1305243_predicted	-1.18	-1.73	-1.76	-1.75	-1.88	-1.76	-2.01
ILMN_1355617	Zfp692_predicted	-1.15	-1.39	-1.21	-1.18	-1.96	-1.62	-2.01
ILMN_1367817	Phf5a	-1.11	-1.67	-1.56	-1.49	-1.4	-1.37	-2.01
ILMN_1649965	Car14_predicted	-1.05	-1.26	-1.04	-1.5	-2	-2	-2
ILMN_1356521	Tesk2	-1.27	-1.54	-1.44	-1.52	-1.76	-1.69	-2
ILMN_1365830	Gamt	-1.18	-1.54	-1.92	-1.49	-2.47	-2.46	-1.99
ILMN_1363657	RGD1560583_predicted	-1.06	-2.42	-1.87	-2.16	-2.25	-2.2	-1.99
ILMN_1374500	RGD1311980	-1.31	-1.37	-1.16	-1.25	-1.91	-2.14	-1.99
ILMN_1354580	Stard4_predicted	-1.17	-2.23	-2.45	-2.58	-2.29	-1.98	-1.99
ILMN_1364575	Pfkfb1	-1.09	-1.7	-1.34	-1.61	-1.67	-1.91	-1.99
ILMN_1374643	Ube2s_predicted	-1.01	-1.62	-1.08	-1.55	-1.81	-1.73	-1.99
ILMN_1357757	Bdh1	-1.1	-1.74	-1.7	-2.15	-1.67	-1.6	-1.99
ILMN_1362987	Hgfac	-1.3	-1.46	-1.69	-1.87	-2.76	-3.39	-1.98
ILMN_1361447	Tle2	-1.92	-1.06	-1.31	-1.54	-2.3	-2.55	-1.98

ILMN_1361266	Ppap2b	-1.34	-1.61	-1.95	-1.83	-1.83	-1.86	-1.98
ILMN_1364235	Fh1	-1.13	-1.37	-1.16	-1.78	-1.81	-1.84	-1.98
ILMN_1366540	LOC500721	-1.35	-1.17	-1.65	-1.71	-1.78	-1.82	-1.98
ILMN_1372735	Cyp4f2	-1.45	-1.32	-1.23	-1.39	-1.39	-1.69	-1.98
ILMN_1358568	Slc7a7	1.07	1.09	1.05	-1.25	-1.2	-1.48	-1.98
ILMN_1352854	Ivd	-1.36	-1.96	-1.63	-2.72	-2.21	-2.58	-1.97
ILMN_1369554	Gltp_predicted	-1.14	-2.33	-1.65	-1.82	-1.74	-1.8	-1.97
ILMN_1368742	LOC679295	-1.1	-1.32	-1.72	-1.64	-1.78	-1.5	-1.97
ILMN_1369211	LOC500949	-1.06	-1.19	1	1.15	1.01	-1.22	-1.97
ILMN_1376454	Tardbp	-1.24	-1.23	-1.25	-1.14	-1.77	-1.72	-1.96
ILMN_1351334	Rad1_predicted	1.08	-1.5	-1.13	-1.1	-1.71	-1.61	-1.96
ILMN_1349535	Rbm10	-1.1	-1.45	-1.19	-2	-1.68	-1.6	-1.96
ILMN_1651057	St3gal6	-1.18	-1.06	-1.45	-1.69	-1.47	-1.59	-1.96
ILMN_1370719	Fbln1_predicted	1.21	1.12	-1.13	1.34	-1.5	-1.02	-1.96
ILMN_1363220	LOC499770	-1.14	-1.39	-1.02	-1.39	-2	-2.12	-1.95
ILMN_1371494	Amacr	-1.33	-1.69	-2.34	-3.79	-1.93	-1.82	-1.95
ILMN_1352522	LOC361346	-1.06	-1.49	-1.39	-1.28	-1.85	-1.81	-1.95
ILMN_1376357	Acy1	1.06	-1.11	1.38	-1.23	-1.66	-1.8	-1.95
ILMN_1351772	Pold2	1.13	-1.31	-1.18	-1.16	-1.88	-1.69	-1.95
ILMN_1358252	Psph	-1.06	-1.2	-1.25	-1.23	-1.29	-1.55	-1.95
ILMN_1361110	Ass	-1.29	-1.22	-1.09	-1.5	-2.44	-3.03	-1.94
ILMN_1353921	Mttp	-1.11	-1.06	-1.41	-1.71	-1.77	-2.27	-1.94
ILMN_1349106	Wdr23	-1.38	-1.59	-1.83	-1.97	-1.91	-1.8	-1.94
ILMN_1360123	RGD1562119_predicted	-1.16	-1.72	-1.4	-1.59	-1.75	-1.65	-1.94
ILMN_1353537	RGD1310495_predicted	-1.19	-1.29	-1.68	-1.88	-1.9	-1.98	-1.93
ILMN_1366787	Cbx3	-1.25	-1.52	-1.26	-1.57	-1.95	-1.86	-1.93
ILMN_1353127	Tbc1d12_predicted	1.07	-1.35	-1.21	1.05	-2.37	-1.85	-1.93
ILMN_1350577	Zcchc8_predicted	-1.24	-1.26	-1.36	-1.36	-1.81	-1.79	-1.93
ILMN_1361131	Mutyh	-1.12	-1.28	-1.49	-1.3	-1.96	-1.77	-1.93
ILMN_1370617	LOC678896	-1.21	-1.56	-1.38	-1.69	-2.08	-1.6	-1.93
ILMN_1368909	RGD1309095_predicted	-1.01	-1.28	-1.21	-1.11	-1.52	-1.58	-1.93
ILMN_1356020	Sf3a3	-1.06	-1.38	-1.17	-1.26	-1.58	-1.55	-1.93
ILMN_1365511	LOC499980	-1.16	-1.07	1.01	-1.33	-1.6	-1.44	-1.93
ILMN_2039973	Bhmt	-1.12	-1.24	-1.26	-1.77	-1.87	-2.26	-1.92
ILMN_1355343	Timm17b_predicted	-1.04	-1.88	-1.8	-1.95	-2.11	-2.04	-1.92
ILMN_1368486	Pc	-1.06	-1.4	-1.19	-1.85	-1.86	-1.78	-1.92
ILMN_1350418	LOC498736	1.13	-1.98	-1.8	-1.73	-1.43	-1.6	-1.92
ILMN_1352264	Pkn3_predicted	-1.33	-1.28	-1.32	-1.34	-1.57	-1.49	-1.92

ILMN_1373971	Aurkc_predicted	-1.31	-1.95	-1.67	-1.98	-1.97	-2.08	-1.91
ILMN_1353057	Eppb9_predicted	-1.12	-1.87	-2.01	-1.67	-1.68	-2.07	-1.91
ILMN_1375121	Tcf2	-1.57	-1.48	-1.63	-1.59	-1.83	-1.99	-1.91
ILMN_1362683	Agxt2	-1.35	-1.02	1.24	-1.5	-1.33	-1.72	-1.91
ILMN_1376480	Hmgcs1	-1.44	-3	-2.67	-2.84	-1.46	-1.57	-1.91
ILMN_1374749	Emg1_predicted	1.03	-1.5	-1.38	-1.16	-2.42	-2.62	-1.9
ILMN_1349705	Anxa4	-1.25	-1.87	-2	-1.88	-1.73	-2	-1.9
ILMN_1351353	Chaf1b	-1.25	-1.66	-1.42	-1.69	-1.51	-1.75	-1.9
ILMN_1366143	Sfrs7_predicted	-1.34	-1.46	-1.19	-1.38	-1.92	-1.74	-1.9
ILMN_1359078	Slc37a4	-1.21	-1.31	-1.13	-1.47	-1.67	-1.69	-1.9
ILMN_1360877	E2f1	-1.03	-1.6	-1.69	-1.75	-1.7	-1.66	-1.9
ILMN_1369927	Tmco3_predicted	-1.66	-1.81	-1.73	-2.13	-1.88	-1.6	-1.9
ILMN_1351027	Csrp2	-1.15	-1.54	-1.62	-1.58	-1.16	-1.21	-1.9
ILMN_1352121	Rnase1	-1.61	-2.02	-2.22	-2.67	-2.21	-2.63	-1.89
ILMN_1372487	LOC296599	1.01	-1.16	-1.13	-1.29	-1.72	-1.91	-1.89
ILMN_1371671	Prpf4_predicted	-1.09	-1.39	-1.22	-1.28	-1.96	-1.83	-1.89
ILMN_1356188	RGD1562954_predicted	1.03	-2.61	-2.38	-3.36	-2.16	-1.81	-1.89
ILMN_1352790	Sip1	1.08	-1.35	-1.5	-1.61	-1.79	-1.55	-1.89
ILMN_1366126	RGD1564040_predicted	1.16	1.19	1.07	1.14	-1.07	-1.19	-1.89
ILMN_1649797	LOC499560	1.03	1.08	1.39	1.29	1.24	-1.11	-1.89
ILMN_1365302	Snf1lk	-1.22	-1.98	-2.08	-1.79	-1.85	-2.17	-1.88
ILMN_1356152	Dnajc14	-1.25	-1.51	-1.58	-1.65	-1.81	-2.03	-1.88
ILMN_1365397	RGD1311634_predicted	-1.22	-1.73	-1.87	-1.93	-1.59	-1.78	-1.88
ILMN_1351188	RGD1305475_predicted	-1.1	-1.59	-1.66	-1.61	-1.72	-1.73	-1.88
ILMN_1371743	Nup133_predicted	-1.13	-1.69	-1.66	-1.48	-1.94	-1.7	-1.88
ILMN_1650297	RGD1308828	-1.18	-1.86	-1.34	-1.63	-1.71	-1.57	-1.88
ILMN_1361515	Mrip	-1.05	-1.38	-1.45	-1.42	-1.56	-1.31	-1.88
ILMN_1366454	RGD1560183_predicted	-1.05	-1.85	-2.66	-1.98	-1.91	-2.29	-1.87
ILMN_1362581	Itpka	-1.25	-1.34	-1.19	1.01	-2.09	-2.14	-1.87
ILMN_1352974	LOC498973	1.03	1.01	1.06	-1.08	1.09	1.13	-1.87
ILMN_1356482	Acadvl	-1.1	-1.8	-2	-2.57	-2.32	-2.88	-1.86
ILMN_1368529	L3mbtl2	-1.21	-1.4	-1.65	-1.62	-2.14	-1.98	-1.86
ILMN_1357045	RGD1310710_predicted	1.01	-1.59	-1.27	-1.32	-1.93	-1.83	-1.86
ILMN_1364865	LOC499206	-1.21	1.05	-1.1	-1.01	-1	-1.16	-1.86
ILMN_1365433	LOC498368	-1.1	-1.31	-1.92	-1.78	-2.3	-3.08	-1.85
ILMN_1365523	Mthfd1	-1.15	-1.33	-1.38	-1.35	-2.24	-2.47	-1.85
ILMN_1374372	Ptplb_predicted	-1	-1.42	-1.14	-1.48	-2.15	-2.21	-1.85
ILMN_1374339	Alg8	-1.14	-1.8	-1.51	-1.73	-1.95	-2.14	-1.85

ILMN_1358679	Ralgps2	-1.14	-1.65	-1.78	-1.77	-2.06	-1.99	-1.85
ILMN_1357709	Nola2_predicted	1.03	-1.18	-1.65	-1.29	-2.01	-1.79	-1.85
ILMN_1349604	LOC315508	-1.09	-1.56	-1.77	-1.63	-1.78	-1.66	-1.85
ILMN_1363488	Tsc22d3	-1.07	-1.42	-1.63	-1.56	-1.6	-1.57	-1.85
ILMN_1362770	LOC499582	-1.11	-1.02	-1.02	1.08	-1.04	-1.04	-1.85
ILMN_1375191	LOC316373	-1.24	-2.34	-1.87	-2.02	-2.91	-2.35	-1.84
ILMN_1367779	RGD1311155	-1.31	-1.51	-1.23	-2.13	-1.91	-1.99	-1.84
ILMN_1367312	Pck2_predicted	-1.02	-1.07	1.13	-1.31	-1.62	-1.77	-1.84
ILMN_1352641	LOC363416	1.06	-1.43	-1.43	-1.13	-1.85	-1.75	-1.84
ILMN_1371636	RGD1308089_predicted	1.01	-1.31	-1.64	-1.28	-1.93	-1.65	-1.84
ILMN_1351053	Cenpf	-1.15	-1.34	-1.32	-1.57	-1.88	-1.57	-1.84
ILMN_1361057	Ung	-1.15	-1.49	-1.55	-1.38	-1.27	-1.36	-1.84
ILMN_1366749	Zfp207_predicted	-1.3	-1.83	-1.72	-1.84	-1.29	-1.31	-1.84
ILMN_1530377	Tmem97	1.06	-1.73	-1.2	-1.32	-1.92	-2.1	-1.83
ILMN_1360876	Tmem97	1.06	-1.73	-1.2	-1.32	-1.92	-2.1	-1.83
ILMN_1363856	Slc25a5	-1.06	-1.47	-1.03	-1.34	-2.18	-2.06	-1.83
ILMN_1373516	RGD1560896_predicted	-1.14	-1.54	-1.02	-1.39	-2.26	-2.04	-1.83
ILMN_1352512	RGD1560896_predicted	-1.14	-1.54	-1.02	-1.39	-2.26	-2.04	-1.83
ILMN_1373050	Hnrpa1	-1.2	-2.21	-1.72	-2.63	-2.38	-1.9	-1.83
ILMN_1359244	Atp5a1	-1.19	-1.72	-1.27	-1.81	-1.9	-1.88	-1.83
ILMN_1369636	Cdk2ap1_predicted	-1.06	-1.92	-2.09	-1.74	-2.02	-1.82	-1.83
ILMN_1651182	Ankrd15	-1.15	-1.63	-2.13	-2.46	-1.68	-1.7	-1.83
ILMN_1368771	Ddb2_predicted	-1.21	-1.42	-1.4	-1.64	-1.43	-1.42	-1.83
ILMN_1360164	Hnrpdl_predicted	-1.05	-1.06	-1.03	-1.27	-1.74	-1.36	-1.83
ILMN_1370211	Lrrc50	-1.02	-1.87	-1.77	-1.46	-1.89	-1.91	-1.82
ILMN_1373203	Kif12	-1.34	-1.21	-1.21	-1.32	-1.62	-1.79	-1.82
ILMN_1364585	LOC301126	-1.26	-1.72	-1.2	-1.76	-2.54	-1.78	-1.82
ILMN_1354034	Plxnb2	-1.13	-1.2	-1.07	-1.38	-1.72	-1.66	-1.82
ILMN_1354069	Gpr19	1.01	-1.64	-1.56	-1.85	-1.72	-1.64	-1.82
ILMN_1372000	Sfrs10	-1.3	-1.46	-1.27	-1.71	-1.19	-1.28	-1.82
ILMN_1362061	LOC500507	1.02	-1.15	1.01	-1	1.01	-1.18	-1.82
ILMN_1367115	Ugt2b	-1.07	-1.62	-2.22	-2.89	-2.96	-2.82	-1.81
ILMN_1368887	Ebp	-1.17	-2	-2.34	-2.41	-2.21	-2.4	-1.81
ILMN_1364217	RGD1307688	-1.2	-1.29	-1.19	-1.36	-1.75	-1.66	-1.81
ILMN_1360908	Tfrc	1.28	-1.34	1	-1.14	-1.53	-1.31	-1.81
ILMN_1651040	C9	-1.05	-1.99	-3.19	-3.17	-2.3	-2.8	-1.8
ILMN_1369207	Cdca7	1.13	1.02	1.32	1.29	-1.91	-1.86	-1.8
ILMN_1530501	Cdca7	1.13	1.02	1.32	1.29	-1.91	-1.86	-1.8

ILMN_1352005	Mig12	-1.33	-1.72	-1.48	-1.59	-1.78	-1.71	-1.8
ILMN_1361575	Adm2	-1.1	1.08	-1.06	-1.19	-1.45	-1.67	-1.8
ILMN_1370362	Myef2	1.01	-1.21	-1	-1.26	-1.61	-1.65	-1.8
ILMN_1352949	Arid1a_predicted	-1.11	-1.39	-1.38	-1.8	-1.76	-1.63	-1.8
ILMN_1361041	Irf3	-1.11	1.14	1.21	1.13	-1.47	-1.57	-1.8
ILMN_1376492	Zfp367	-1.18	-1.27	-1.35	-1.48	-1.39	-1.47	-1.8
ILMN_1371231	Sfpq	-1.18	-1.71	-1.9	-2.23	-1.6	-1.42	-1.8
ILMN_1363007	RGD1560795_predicted	-1.34	-2.57	-2.57	-1.99	-1.89	-2.41	-1.79
ILMN_1367643	Olfml3_predicted	-1.02	-1.85	-1.51	-2.18	-2.12	-1.76	-1.79
ILMN_1350522	Nfix	-1.16	-1.27	-1.76	-1.55	-2.02	-1.76	-1.79
ILMN_1374336	RGD1307084	-1.11	-1.32	-1.26	-1.34	-1.69	-1.59	-1.79
ILMN_1361248	LOC500428	-1.18	-1.03	-1.1	1.02	-1.15	-1.22	-1.79
ILMN_1360426	RGD1308123	-1.18	-1.39	-1.23	-1.57	-1.85	-1.69	-1.78
ILMN_1376025	Obfc1_predicted	1.17	-1.84	1.01	-1.74	-1.44	-1.46	-1.78
ILMN_1362599	RGD1309020	-1.19	-1.26	-1.52	-1.08	-1.49	-1.38	-1.78
ILMN_1359795	LOC499555	-1.04	1.09	-1.07	1.08	1.05	-1.35	-1.78
ILMN_1355941	Usp52	-1.18	-1.29	-1.2	-1.04	-1.2	-1.08	-1.78
ILMN_1362081	LOC361117	-1.02	-1.01	-1.26	1.05	1.04	1.07	-1.78
ILMN_1354128	LOC497867	-1.34	-1.71	-1.37	-1.44	-1.81	-2.01	-1.77
ILMN_1357346	Mospd3	-1.35	-2.43	-2.14	-2.33	-1.93	-1.81	-1.77
ILMN_1361957	Rab3ip	-1.09	-1.74	-1.48	-1.8	-1.85	-1.73	-1.77
ILMN_1373286	LOC498696	-1.64	-1.71	-1.48	-1.99	-1.5	-1.61	-1.77
ILMN_1361680	Trim28	-1.12	-1.02	-1.19	-1.02	-1.47	-1.46	-1.77
ILMN_1373506	Map2k3	1.1	-1.27	-1.63	-1.15	-1.24	-1.37	-1.77
ILMN_1361255	RGD1564512_predicted	-1.08	-1.42	1.04	-1.27	-2.25	-1.8	-1.76
ILMN_1360593	LOC314859	-1.09	-1.81	-1.69	-1.9	-1.82	-1.7	-1.76
ILMN_1361584	RGD1565889_predicted	-1.05	-1.5	-1.18	-1.36	-1.55	-1.69	-1.76
ILMN_1365332	St6gal1	-1.13	-1.76	-2.57	-2.58	-1.68	-1.63	-1.76
ILMN_1355370	RGD1562476_predicted	-1.17	-1.88	-2.44	-2.21	-2.08	-2.29	-1.75
ILMN_1371196	Spp2	-1.21	-1.77	-2.01	-2.03	-1.66	-2.28	-1.75
ILMN_1368764	LOC499417	-1.14	-1.56	-1.21	-1.64	-1.85	-1.9	-1.75
ILMN_1370484	Alms1_predicted	-1.17	-1.73	-2.1	-1.94	-1.83	-1.64	-1.75
ILMN_1357610	RGD1359144	-1.11	-1.49	-1.35	-1.09	-1.58	-1.56	-1.75
ILMN_1352015	RGD1308584_predicted	-1	-1.5	-1.66	-1.7	-1.49	-1.5	-1.75
ILMN_1350919	Clcc1	-1.25	-1.31	1.59	-1.57	-1.6	-1.34	-1.75
ILMN_1359052	Rhbg	-1.08	1.03	-1	-1.45	-1.9	-2.48	-1.74
ILMN_1350718	Cpox	1.06	-1.73	-1.51	-1.68	-2.22	-2.13	-1.74
ILMN_1370199	Stard7_predicted	-1.16	-1.49	-1.54	-1.65	-2.24	-2.06	-1.74

ILMN_1366266	Rtkn	-1.06	-1.05	-1.38	-1.13	-1.89	-1.84	-1.74
ILMN_1375024	Slc23a1	-1.26	-1.34	-1.51	-1.48	-1.81	-1.84	-1.74
ILMN_1374598	Tm4sf12	-1.17	-1.8	-1.69	-2.3	-1.66	-1.74	-1.74
ILMN_1371223	Fbl	-1.04	-1.31	-1.26	-1.22	-1.89	-1.65	-1.74
ILMN_1372087	Anapc5_predicted	-1.19	-1.93	-1.04	-1.75	-1.91	-1.54	-1.74
ILMN_1369290	RGD1559935_predicted	1.68	-1.12	-1.18	-1.1	-1.28	-1.21	-1.74
ILMN_1357604	Hspb6	-1.08	-1.67	-1.26	-1.66	-1.75	-1.97	-1.73
ILMN_1370802	Taf13_predicted	-1.22	-2.57	-2.14	-2.26	-1.91	-1.89	-1.73
ILMN_1359684	Wdhd1_predicted	-1.16	-1.58	-1.55	-1.15	-1.82	-1.86	-1.73
ILMN_1374757	Ndufs8_predicted	-1.2	-1.81	-1.92	-1.96	-2	-1.85	-1.73
ILMN_1360118	MGC112830	-1.06	-1.17	1.02	1.08	-1.56	-1.47	-1.73
ILMN_1649986	RGD1562162_predicted	-1.15	-1.88	-2.14	-2.11	-1.76	-1.41	-1.73
ILMN_1367786	MGC73003	1.02	-1.12	1.01	-1.21	-1.09	-1.21	-1.73
ILMN_1363017	Mybl1_predicted	-1.06	-1.39	-1.3	-1.22	-1.72	-1.15	-1.73
ILMN_1359044	Es2	1.15	-2.62	-3.58	-4.28	-2.84	-2.81	-1.72
ILMN_1353589	Sult1b1	-1.22	-2.04	-2.09	-2.76	-2.29	-2.24	-1.72
ILMN_1375780	Maf	1.14	-1.83	-2.1	-1.85	-2.28	-2.23	-1.72
ILMN_1353122	Maged1	-1.42	-2.07	-1.86	-2.24	-2.15	-2.12	-1.72
ILMN_1370405	Calm2	-1.26	-1.81	-1.51	-1.79	-1.81	-1.87	-1.72
ILMN_1352866	Adam8_predicted	-1.09	-1.63	-1.77	-1.83	-1.86	-1.76	-1.72
ILMN_1353973	Tpcn2_predicted	-1.28	-1.17	-1.28	-1.16	-1.94	-1.73	-1.72
ILMN_1352346	Ccnf	-1.42	-1.5	-1.59	-1.37	-1.74	-1.73	-1.72
ILMN_1374454	LOC309557	-1.2	-1.16	-1.19	-1.07	-1.6	-1.67	-1.72
ILMN_1349806	Whsc2	-1.21	-1.45	-1.26	-1.32	-1.35	-1.25	-1.72
ILMN_1371682	Nme3	-1.2	-1.43	-2.35	-1.79	-2.43	-2.9	-1.71
ILMN_1360844	Mrs2l	-1.19	-1.49	-1.46	-1.53	-2.18	-2.18	-1.71
ILMN_1369859	Xpnpep2	-1.06	-1.75	-2.02	-2.01	-2.04	-2.16	-1.71
ILMN_1367822	Pigr	-1	-1.64	-1.83	-1.86	-1.39	-2.07	-1.71
ILMN_1349888	Il1rl1l	-1.13	-1.63	-1.62	-1.46	-2.77	-2.05	-1.71
ILMN_1352437	Gtf2h4	-1.27	-1.76	-1.61	-1.81	-2.13	-1.86	-1.71
ILMN_1362592	LOC299002	-1.2	-1.34	1.04	-1.24	-1.89	-1.69	-1.71
ILMN_1370138	Hnrph1	-1.15	-1.29	-1.4	-1.57	-1.98	-1.64	-1.71
ILMN_1650160	LOC307347	-1.1	-1.43	-1.36	-1.4	-1.69	-1.49	-1.71
ILMN_1372216	Toe1_predicted	-1.23	-1.75	-1.87	-1.74	-1.82	-1.41	-1.71
ILMN_1355705	Slc3a1	-1.28	-1.68	1.09	-1.72	-1.07	-1.37	-1.71
ILMN_1365953	Silv_predicted	-1.01	-1.15	-1.18	-1.23	-1.4	-1.32	-1.71
ILMN_1364065	LOC497692	1.1	-1.43	-1.26	-1.16	-1.56	-1.26	-1.71
ILMN_1373251	LOC500285	-1	-1.01	-1.3	1.1	1.26	1.15	-1.71

ILMN_1368395	Ddx23_predicted	-1.1	-1.36	-1.08	-1.28	-1.84	-1.62	-1.7
ILMN_1363457	Sf3b2_predicted	-1.12	-1.36	-1.51	-1.55	-1.78	-1.51	-1.7
ILMN_1349125	RGD1307935	-1.3	-1.49	-1.17	-1.16	-1.14	-1.41	-1.7
ILMN_1365399	LOC499213	-1.26	-1.73	-1.91	-1.6	-1.42	-1.38	-1.7
ILMN_1355274	LOC310612	-1.1	-1.32	-1.06	-1.2	-1.35	-1.3	-1.7
ILMN_1650298	LOC498076	-1.03	1.07	-1.31	1.16	1.12	1.11	-1.7
ILMN_1365849	RGD1565561_predicted	-1.21	-2.26	-2	-1.65	-1.94	-1.89	-1.69
ILMN_1356593	Spsb3_predicted	-1.09	-1.42	-1.38	-1.61	-1.79	-1.64	-1.69
ILMN_1354341	MGC112883	-1.1	-1.67	-1.44	-1.72	-1.53	-1.59	-1.69
ILMN_1364015	LOC687570	1.08	-1.12	-1.1	-1.13	-1.64	-1.46	-1.69
ILMN_1369706	Dnajb1_predicted	-1.5	-1.48	-1.2	-1.23	-1.53	-1.42	-1.69
ILMN_1352021	Tmx2	1.04	-1.38	1.19	-1.35	-1.22	-1.32	-1.69
ILMN_1374082	Slc25a11	-1.08	-2.03	-1.18	-2.46	-2.16	-2.49	-1.68
ILMN_1362640	Psmc3ip	-1.16	-1.66	-1.73	-1.58	-2.04	-2.41	-1.68
ILMN_1367582	Mif4gd	-1.23	-1.71	-1.43	-1.6	-2.26	-2.1	-1.68
ILMN_1350333	Zfp637	-1.26	-1.8	-1.69	-2.04	-1.93	-1.94	-1.68
ILMN_1362432	Alg6	1.04	-1.47	-1.26	-1.46	-1.76	-1.82	-1.68
ILMN_1358688	Sdhd	-1.18	-1.83	-1.67	-1.91	-1.86	-1.77	-1.68
ILMN_1369643	Mrpl37	-1.16	-1.4	1.16	-1.64	-1.84	-1.75	-1.68
ILMN_1651163	LOC500077	-1.15	-1.35	-1.27	-1.25	-1.86	-1.73	-1.68
ILMN_1376647	LOC304396	-1.11	1.02	-1.17	-1.1	-1.56	-1.61	-1.68
ILMN_1351140	RGD1306274	-1.16	-1.44	-1.82	-1.75	-1.73	-1.6	-1.68
ILMN_1650466	LOC684771	-1.13	-1.53	-1.12	-1.48	-1.63	-1.58	-1.68
ILMN_1372829	RGD1310671_predicted	-1.17	-1.59	-1.32	-1.49	-1.69	-1.51	-1.68
ILMN_1357966	Phgdh	-1.16	-1.16	-1.05	-1.19	-1.28	-1.3	-1.68
ILMN_1365322	RGD1560358_predicted	-1.14	-1.38	-1.39	-1.28	-1.24	-1.26	-1.68
ILMN_1376515	Orc2l	1.15	-1.16	1.13	-1.12	-1.05	-1.09	-1.68
ILMN_1361315	Cep152_predicted	-1.15	-1.32	-1.05	1.08	-1.08	1.09	-1.68
ILMN_1369708	LOC500586	-1.06	-1.12	-1.15	1.18	1.32	1.14	-1.68
ILMN_1363497	Onecut1	-1.01	-1.41	-1.5	-1.69	-2.61	-2.67	-1.67
ILMN_1372174	Pycard	-1.32	-1.45	-1.84	-1.81	-1.98	-2.52	-1.67
ILMN_1360629	Snrpa1_predicted	1.12	-1.37	-1.01	-1.31	-2.04	-2.35	-1.67
ILMN_1360394	Rbl2	-1.26	-2.17	-2.36	-3.08	-2	-2.2	-1.67
ILMN_1364858	Taf10_predicted	-1.03	-1.91	-2.24	-1.78	-1.74	-2.09	-1.67
ILMN_1353248	LOC361596	-1.07	-1.55	-1.4	-2.06	-1.88	-2.04	-1.67
ILMN_1651016	Paqr4	-1.26	-1.72	-1.76	-1.86	-1.73	-1.96	-1.67
ILMN_1361733	Wnk4	-1.28	1.11	-1.38	-1.28	-1.85	-1.9	-1.67
ILMN_1352828	MGC94282	-1.17	-1.45	-1.27	-1.47	-1.91	-1.67	-1.67

ILMN_1650164	RGD1310674	-1.09	-1.57	-1.51	-1.53	-1.68	-1.67	-1.67
ILMN_1372845	RGD1308317_predicted	-1.05	-1.85	-1.37	-1.54	-1.58	-1.57	-1.67
ILMN_1363250	Mbtps1	-1.17	-1.6	-1.9	-2.05	-1.73	-1.53	-1.67
ILMN_1360839	LOC501652	-1.21	-1.08	1.34	-1.03	1.04	-1.43	-1.67
ILMN_1357954	Anapc1_predicted	1.2	1.16	-1.22	-1.11	-1.52	-1.41	-1.67
ILMN_1372095	Irf1	-1.02	-1.57	-1.36	-1.6	-1.5	-1.27	-1.67
ILMN_1356194	Mkks	1.01	-2.03	-1.82	-1.77	-1.17	-1.12	-1.67
ILMN_1358378	Ugt2b5	-1.19	-1.81	-2.01	-2.4	-2.24	-2.27	-1.66
ILMN_1372708	Gbl	-1.14	-1.4	-1.33	-1.79	-2.22	-2.16	-1.66
ILMN_1372643	RGD1563634_predicted	-1.28	-1.17	1.04	-1.08	-2.17	-2.1	-1.66
ILMN_1350539	Chd1l_predicted	-1.16	-1.46	-1.66	-1.83	-1.86	-1.82	-1.66
ILMN_1376407	Tm9sf1	-1.14	-1.71	-1.56	-1.98	-2.06	-1.8	-1.66
ILMN_1370966	Xrcc1	-1.19	-1.41	-1.94	-1.71	-1.72	-1.73	-1.66
ILMN_1363414	LOC365025	-1.01	-1.78	-1.42	-1.37	-1.65	-1.72	-1.66
ILMN_1376600	Tpst1	1.02	-1.46	-1.05	-1.2	-1.7	-1.69	-1.66
ILMN_1353884	Sfmbt1	-1.07	-1.1	-1.17	-1.19	-1.83	-1.49	-1.66
ILMN_1367890	RGD1311822_predicted	1.02	-1.58	-1.24	-1.36	-1.61	-1.43	-1.66
ILMN_1354759	RGD1565754_predicted	-1.13	-1.37	-1.58	-1.2	-1.66	-1.41	-1.66
ILMN_1651054	LOC684490	1.21	1.11	1.32	-1.04	-1.2	-1.27	-1.66
ILMN_1363480	Srebf1	-1.29	-1.69	-1.27	-1.77	-1.54	-1.9	-1.65
ILMN_1359119	RGD1564611_predicted	-1.24	-1.3	-1.41	-1.47	-1.53	-1.77	-1.65
ILMN_1365019	LOC299949	1.11	-1.46	-1.55	-1.59	-1.84	-1.67	-1.65
ILMN_1365020	RGD1560681_predicted	1.07	-1.31	-1.04	-1.38	-1.66	-1.62	-1.65
ILMN_1359725	Brd9_predicted	-1.35	-1.35	-1.46	-1.65	-1.89	-1.61	-1.65
ILMN_1349254	Cntf	-1.02	-1.28	-1.31	-1.11	-1.07	-1.31	-1.65
ILMN_1530528		-1.01	-1.67	-2.25	-1.51	-1.31	-1.45	-1.64
ILMN_1350834	Phyh	-1.37	-1.33	-1.76	-2.25	-2.04	-2.66	-1.64
ILMN_1351331	MGC94780	-1.38	-2	-1.6	-1.71	-2.04	-2.09	-1.64
ILMN_1360418	RGD1302996	-1.37	-1.3	-1.84	-1.22	-1.89	-1.93	-1.64
ILMN_1357330	Cpb2	-1.05	-1.26	-1.46	-1.96	-1.38	-1.91	-1.64
ILMN_1358087	Synj2	-1.32	-1.43	-1.73	-1.49	-1.74	-1.76	-1.64
ILMN_1363601	RGD1560020_predicted	-1.13	-1.38	-1.5	1.14	-1.61	-1.7	-1.64
ILMN_1362035	Ifrd2_predicted	1.2	1.14	1.02	1.11	-1.8	-1.58	-1.64
ILMN_1371715	Wbp11	-1.22	-1.37	-2.2	-1.51	-1.6	-1.56	-1.64
ILMN_1359111	Ccdc21	-1.07	-1.05	1.1	1.07	-1.36	-1.25	-1.64
ILMN_1372167	Rap1ga1	-1.4	-1.5	-1.44	-1.89	-1.78	-2.07	-1.63
ILMN_1350003	Pde4d	-1.03	-1.58	-1.81	-1.41	-1.59	-1.91	-1.63
ILMN_1361928	Zdhhc2	-1.11	-1.57	-1.54	-1.61	-1.58	-1.59	-1.63

ILMN_1365269	Pop4	-1.17	-1.72	-1.63	-1.59	-1.79	-1.53	-1.63
ILMN_1367568	Pdgfc	-1.06	-1.37	-1.31	-1.43	-1.46	-1.52	-1.63
ILMN_1361185	LOC686577	1.3	-1.28	-1.08	-1.11	-1.26	-1.49	-1.63
ILMN_1368539	Cxxc1	-1.24	-1.17	-1.44	-1.26	-1.59	-1.46	-1.63
ILMN_1365570	Ubadc1	-1.12	-1.74	-1.53	-1.4	-1.84	-1.45	-1.63
ILMN_1369009	Mybl2_predicted	-1.02	-1.35	-1.34	-1.26	-1.39	-1.45	-1.63
ILMN_1362443	LOC497733	-1.16	-1.1	-1.52	-1.31	-1.45	-1.41	-1.63
ILMN_1352908	RGD1565591_predicted	-1.06	-1.37	-1.42	-1.33	-1.54	-1.3	-1.63
ILMN_1359113	Kifc3	-1.11	-1.19	-1.11	-1.35	-2.13	-1.85	-1.62
ILMN_1359213	Pklr	-1.22	-1.35	-1.36	-1.53	-1.67	-1.75	-1.62
ILMN_1373241	Exosc5_predicted	1.02	-1.53	-1.6	-1.89	-2.19	-1.64	-1.62
ILMN_1362886	Hp1bp3	-1.27	-1.42	-1.36	-1.62	-2.07	-1.64	-1.62
ILMN_1373264	Parp2_predicted	-1.07	-1.42	-1.33	-1.51	-1.46	-1.47	-1.62
ILMN_1371600	Cdk5rap2	-1.03	-1.46	-1.58	-1.65	-1.87	-1.46	-1.62
ILMN_1372348	LOC499636	-1.17	-1.41	-1.28	-1.42	-1.66	-1.44	-1.62
ILMN_1350995	Tmem17	-1.12	-1.3	-1.29	-1.46	-1.63	-1.44	-1.62
ILMN_1373152	B4galnt1	-1.25	-1	-1.11	-1.05	-1.32	-1.4	-1.62
ILMN_1374912	Mcrs1	-1.38	-1.48	-1.65	-1.61	-1.5	-1.35	-1.62
ILMN_1359299	Cabc1	-1.09	-2.15	-2.45	-3.15	-2.08	-2.04	-1.61
ILMN_1352928	Acpl2	-1.01	-1.43	-1.27	-1.46	-1.73	-1.65	-1.61
ILMN_1350324	Atp5b	-1.18	-1.5	-1.35	-1.74	-1.71	-1.6	-1.61
ILMN_1363645	RGD1306433_predicted	-1.14	-1.39	-1.37	-1.32	-1.53	-1.57	-1.61
ILMN_1359316	Nipa2_predicted	-1	-1.35	-1.08	-1.23	-1.62	-1.54	-1.61
ILMN_1364045	LOC499767	-1.15	-1.54	-1.22	-1.42	-1.64	-1.49	-1.61
ILMN_1373448	Wbp1	-1.15	-1.55	-1.54	-1.52	-1.42	-1.42	-1.61
ILMN_1366252	Ccne1	-1.35	-1.47	-1.3	-1.17	-1.44	-1.4	-1.61
ILMN_1353346	Ltbp4	-1.15	-1.17	-1.41	-1.5	-1.34	-1.37	-1.61
ILMN_1350108	Safb	-1.19	-1.33	-2.18	-1.48	-1.47	-1.35	-1.61
ILMN_1352980	Amd1	-1.08	-1.13	1.27	1.07	-1.08	-1.29	-1.61
ILMN_1371910	Ube2e3_predicted	-1.06	-1.3	-1.46	-1.43	-1.35	-1.24	-1.61
ILMN_1372150	Lsm8_predicted	-1.02	-1.64	-1.95	-1.71	-1.21	-1.11	-1.61
ILMN_1361132	Map2k6	-1.33	-1.88	-2.4	-2.32	-1.81	-2.1	-1.6
ILMN_1363338	Gata6	-1.07	-1.43	-1.61	-1.65	-1.87	-1.9	-1.6
ILMN_1362572	Gramd1b_predicted	-1.06	-1.13	1.04	1.03	-1.94	-1.82	-1.6
ILMN_1371455	RGD1307897	-1.12	-1.36	-1.46	-1.4	-1.92	-1.71	-1.6
ILMN_1361482	Wee1	-1.12	-1.11	-1.09	1.17	-1.83	-1.68	-1.6
ILMN_1365456	Ppp1ca	-1.17	-1.84	-1.53	-1.77	-1.67	-1.67	-1.6
ILMN_1376552	Tia1	1.01	-1.5	-1.06	-1.34	-1.26	-1.52	-1.6

ILMN_1370286	Ctcf	-1.2	-1.59	-1.68	-1.57	-1.67	-1.49	-1.6
ILMN_1376601	Cenpc1	-1.18	-1.38	-1.34	-1.39	-1.68	-1.45	-1.6
ILMN_1372557	H2afy	-1.14	-1.29	-1.26	-1.26	-1.59	-1.39	-1.6
ILMN_1355521	RGD1560103_predicted	-1	-1.28	-1.27	-1.91	-1.59	-1.38	-1.6
ILMN_1650113	RGD1308009	-1.07	-1.34	-1.58	-1.44	-1.41	-1.25	-1.6
ILMN_1357732	Armc5	-1.23	-1.36	-1.31	-1.48	-1.27	-1.22	-1.6
ILMN_1355961	Cdca4	-1	-1.11	-1.43	-1	-1.25	-1.14	-1.6
ILMN_2038793	Alb	-1.2	-1.6	-1.57	-2.26	-2.02	-2.79	-1.59
ILMN_2038792	Alb	-1.2	-1.6	-1.57	-2.26	-2.02	-2.79	-1.59
ILMN_1355605	Vkorc1	-1.04	-1.32	-1.8	-2.22	-2.38	-2.46	-1.59
ILMN_1354910	Akr1b8	-1.05	-1.7	-1.41	-1.94	-2.1	-2.02	-1.59
ILMN_1350892	Afp	-1.09	-1.48	-1.68	-1.91	-1.63	-1.83	-1.59
ILMN_1364341	Fgfr4	-1.39	-1.68	-1.69	-1.83	-1.61	-1.59	-1.59
ILMN_1376519	Nubp2	-1.37	-1.67	-1.5	-1.65	-1.51	-1.55	-1.59
ILMN_1355484	Rom1	-1.25	-1.6	-1.52	-1.63	-1.65	-1.53	-1.59
ILMN_1354339	Depdc1b_predicted	-1.05	-1.48	-1.12	-1.52	-1.39	-1.53	-1.59
ILMN_1349281	RGD1309922_predicted	-1.24	-1.15	-1.57	-1.35	-1.36	-1.41	-1.59
ILMN_1365614	Eif5a	1.04	-1.63	1.08	-1.39	-1.51	-1.35	-1.59
ILMN_1649865	Tial1_mapped	-1.08	1.03	-1.21	1.05	-1.4	-1.31	-1.59
ILMN_1650793	Cdc26	-1.01	-1.44	-1.52	-1.43	-1.46	-1.3	-1.59
ILMN_1362283	Yy1	-1.12	-1.09	-1.37	-1.3	-1.25	-1.2	-1.59
ILMN_1352752	Ctnnb1	-1.24	-1.3	-1.16	-1.41	-1.23	-1.15	-1.59
ILMN_1349285	Dtymk_predicted	-1.11	-1.07	-1.04	-1.05	-1.03	-1.1	-1.59
ILMN_1650984	LOC498404	-1.03	-1.08	-1.22	-1.14	-1.17	-1.04	-1.59
ILMN_1364348	Ca2	-1.17	-4.49	-3.86	-3.99	-3.51	-3.56	-1.58
ILMN_1358888	Psmb10	1.01	-1.57	-2.88	-1.38	-1.56	-1.82	-1.58
ILMN_2040046	Psmb10	1.01	-1.57	-2.88	-1.38	-1.56	-1.82	-1.58
ILMN_1374420	Msh6_predicted	-1.25	-1.16	-1.39	1.08	-1.69	-1.66	-1.58
ILMN_1358867	Cd320	-1.05	-1.11	-1.09	-1.01	-1.74	-1.64	-1.58
ILMN_1359302	Amt	-1.02	-1.32	-1.21	-1.15	-1.42	-1.58	-1.58
ILMN_1351182	Aplp2	1.05	-1.4	-1.25	-1.69	-1.49	-1.57	-1.58
ILMN_1359709	RGD1562178_predicted	1.08	-1.27	-1.06	-1.06	-1.37	-1.47	-1.58
ILMN_1353637	Csad	1.11	-1.2	-1.32	-1.36	-1.13	-1.43	-1.58
ILMN_1350521	RGD1565687_predicted	-1.29	-1.07	-1.33	-1.23	-1.46	-1.36	-1.58
ILMN_1352595	L2hgdh_predicted	-1.23	-1.2	1.02	-1.24	-1.76	-1.32	-1.58
ILMN_1360778	Dhx15_predicted	-1.02	-1.05	1	1.04	-1.04	-1.08	-1.58
ILMN_1353071	Rnpep	-1.04	-1.31	-1.09	-1.44	-1.81	-1.78	-1.57
ILMN_1376785	Metrn	-1.2	-1.7	1.34	-1.34	-1.42	-1.56	-1.57

ILMN_1357878	Lsm5_predicted	1.02	-1.14	-1.92	-1.19	-1.31	-1.33	-1.57
ILMN_1369588	Klc4	-1.17	-1.54	-1.47	-2.68	-1.36	-1.28	-1.57
ILMN_1372821	Fbxo33_predicted	-1.15	-1.37	-1.11	-1.37	1.42	1.38	-1.57
ILMN_1350310	Mecr	-1.14	-1.61	-1.66	-1.53	-1.59	-1.8	-1.56
ILMN_1364448	LOC301067	-1.45	-1.19	-1.13	-1.26	-1.34	-1.61	-1.56
ILMN_1367484	Mrps7	-1.11	-1.22	1	-1.16	-1.57	-1.6	-1.56
ILMN_1373365	RGD1311907_predicted	-1.02	-1.25	-1.42	-1.46	-1.52	-1.55	-1.56
ILMN_1350685	UST4r	-1.21	-1.41	-1.27	-1.33	-1.61	-1.52	-1.56
ILMN_1349443	Magi3	-1	-1.33	-1.39	-1.14	-1.57	-1.52	-1.56
ILMN_1376918	LOC304000	-1.36	-1.76	-1.75	-2.34	-1.29	-1.51	-1.56
ILMN_1349558	Pls1_predicted	1.02	-1.49	-1.7	-2.13	-1.82	-1.42	-1.56
ILMN_1349383	RGD1562582_predicted	-1.14	-1.41	-1.26	-1.34	-1.76	-1.4	-1.56
ILMN_1353499	Lars2_predicted	1.02	-1.28	-1.27	-1.26	-1.66	-1.36	-1.56
ILMN_1353054	Nfatc3_predicted	1.02	-1.32	-1.12	1.11	-1.26	-1.32	-1.56
ILMN_1376386	Pycrl	1.01	-1.19	-1.23	-1.42	-1.54	-1.68	-1.55
ILMN_1366848	Gpsn2	-1.17	-1.83	-1.09	-2.04	-1.73	-1.58	-1.55
ILMN_1349120	Hnrpm	-1.1	-1.51	-1.59	-1.69	-1.5	-1.51	-1.55
ILMN_1361237	RGD1562306_predicted	-1.11	-1.87	-1.62	-1.36	-1.81	-1.5	-1.55
ILMN_1358392	Slc25a3	-1.12	-1.19	1.12	-1.31	-1.7	-1.49	-1.55
ILMN_1355194	Actl6a	-1.21	-1.69	-1.44	-1.6	-1.62	-1.49	-1.55
ILMN_1366291	Cenpj_predicted	-1.38	-1.39	-1.53	-1.45	-1.57	-1.48	-1.55
ILMN_1362562	Sin3a_predicted	-1.2	-1.5	-1.59	-1.52	-1.47	-1.4	-1.55
ILMN_1349756	RGD1310571	1.02	-2.18	-1.69	-2.04	-1.35	-1.38	-1.55
ILMN_1376456	Trip13	1.05	-1.57	-1.22	-1.28	-1.77	-1.35	-1.55
ILMN_1375051	Ssrp1	-1.12	-1.39	-1.27	-1.23	-1.55	-1.35	-1.55
ILMN_1370371	Tm4sf2_mapped	-1.07	-1.47	1.07	-1.35	-1.15	-1.33	-1.55
ILMN_1376291	Recql	-1	-1.22	-1.09	-1.23	-1.36	-1.31	-1.55
ILMN_1360423	RGD1307481	-1.25	-1.75	-1.71	-1.32	-1.31	-1.3	-1.55
ILMN_1349048	Ucp2	1.16	-1.62	-1.12	-1.43	-1.82	-2.13	-1.54
ILMN_1354187	Lancl1	-1.21	-1.54	-1.89	-2.72	-2.01	-2.04	-1.54
ILMN_1358631	Abcg5	1.18	-1.25	-1.38	-1.95	-1.34	-1.61	-1.54
ILMN_1359180	Mrpl18_predicted	-1.19	-1.7	-1.96	-1.76	-1.56	-1.6	-1.54
ILMN_1369339	Rnaseh1	-1.16	-1.48	-1.48	-1.34	-1.52	-1.51	-1.54
ILMN_1358393	RGD1565734_predicted	-1.07	-1.35	-1.36	-1.17	-1.57	-1.47	-1.54
ILMN_1362970	LOC679731	-1.12	-1.45	-1.15	-1.21	-1.63	-1.45	-1.54
ILMN_1351516	Srpk1	1.11	1.13	1.29	1.15	-1.49	-1.36	-1.54
ILMN_1352627	LOC367191	-1.07	-1.07	-1.27	-1.11	-1.48	-1.28	-1.54
ILMN_1370670	Lancl2_predicted	-1.2	-1.57	-1.22	-1.74	-1.31	-1.27	-1.54

ILMN_1355225	Rac1	-1.08	-1.08	-1.01	-1.14	-1.39	-1.24	-1.54
ILMN_1364885	Zdhhc6	1.11	-1.37	1.27	-1.16	-1.34	-1.16	-1.54
ILMN_1349040	Oaz1	-1.02	-1.79	-1.42	-1.88	-2.15	-1.93	-1.53
ILMN_1348834	Tnfsf13	-1.02	-1.12	-1.24	-1.49	-1.52	-1.86	-1.53
ILMN_1650547	Rpo1-1	-1.04	1.07	1.02	-1.05	-1.85	-1.73	-1.53
ILMN_1366889	RGD1307279	1.06	-1.82	-1.52	-1.66	-1.63	-1.57	-1.53
ILMN_1650940	LOC296126	-1.31	-1.34	-1.42	-1.35	-1.56	-1.5	-1.53
ILMN_2040505	LOC685393	-1.11	-1.22	-1.24	-1.32	-1.56	-1.48	-1.53
ILMN_1368239	LOC500251	-1.46	-1.22	-1.29	-1.13	-1.48	-1.44	-1.53
ILMN_1349001	LOC688269	1.15	1.22	1.09	1.82	-1.14	-1.31	-1.53
ILMN_1363754	Maf1	-1.15	-1.53	-2.32	-2.34	-1.38	-1.29	-1.53
ILMN_1351732	LOC688528	1.02	-1.54	-1.07	-1.37	-1.12	-1.14	-1.53
ILMN_1373540	Arf2	-1.06	-1.35	-1.24	-1.45	-1.1	-1.08	-1.53
ILMN_1365599	Bphl	-1.09	-1.27	-1.47	-1.86	-1.97	-2.68	-1.52
ILMN_1361208	Psme1	1.06	-1.33	-1.66	-1.48	-1.96	-2.16	-1.52
ILMN_1649944	Mrps26_predicted	-1.04	-1.44	-1.72	-1.32	-1.59	-2	-1.52
ILMN_1352040	Slc22a3	-1.15	-1.93	-2.16	-1.99	-2.09	-1.73	-1.52
ILMN_1365487	LOC501282	-1.11	-1.55	-1.33	-1.71	-1.94	-1.72	-1.52
ILMN_1352036	RGD1359682	-1.47	-1.5	-1.58	-1.73	-1.54	-1.5	-1.52
ILMN_1353630	RGD1565495_predicted	-1.14	-1.69	-1.23	-1.35	-1.64	-1.46	-1.52
ILMN_1375844	Tia1_predicted	-1.04	-1.57	-1.17	-1.26	-1.65	-1.45	-1.52
ILMN_1358290	Rsafd1_predicted	-1.05	-1.5	-1.42	-1.98	-1.53	-1.45	-1.52
ILMN_1351093	Foxp1_predicted	-1.18	-1.11	-1.38	-1.63	-1.36	-1.42	-1.52
ILMN_1650206	Sox9	-1.23	-1.34	-1.44	-1.42	-1.41	-1.4	-1.52
ILMN_1369534	RGD1307071_predicted	1.18	-1.27	-1.74	-1.93	-1.73	-1.33	-1.52
ILMN_1365715	Frg1_predicted	-1.16	-1.76	-1.78	-2.14	-1.29	-1.22	-1.52
ILMN_1650736	RGD1565368_predicted	-1.09	-1.61	-1.26	-1.55	-1.16	-1.22	-1.52
ILMN_1369651	Pcgf4_predicted	1.1	-1.44	1.02	-1.46	-1.52	-1.2	-1.52
ILMN_1367571	RGD1564688_predicted	-1.06	-1.62	-1.24	-1.32	-1.19	-1.2	-1.52
ILMN_1366398	Pogz_predicted	-1.16	1.06	-1.14	-1.27	-1.08	-1.2	-1.52
ILMN_1362666	LOC497739	-1.44	-1.15	-1.04	-1.13	-1.39	-1.16	-1.52
ILMN_1651004	RGD1562189_predicted	-1.12	-1.11	-1.02	-1.1	-1.35	-1.1	-1.52
ILMN_1363145	Zcwcc1_predicted	1.14	1.08	1.1	-1.28	-1.05	1.02	-1.52
ILMN_1373381	LOC498105	-1.18	-1.24	-1.33	-1.08	1.09	1.2	-1.52
ILMN_1369005	Egr1	-1.58	2.7	1.45	1.08	1.56	1.21	-1.52
ILMN_1364790	RGD1308064_predicted	-1.2	-2.06	-2.15	-2.37	-1.96	-1.9	-1.51
ILMN_1649852	Echdc2_predicted	-1.18	-1.33	-1.62	-1.81	-1.67	-1.84	-1.51
ILMN_1365968	Proc	-1.16	-1.44	-1.55	-1.65	-1.73	-1.79	-1.51

ILMN_1371595	RGD1563945_predicted	-1.2	-1.32	-1.42	-1.24	-1.67	-1.69	-1.51
ILMN_1366876	C8g_predicted	-1.13	1.03	1.07	-1.11	-1.41	-1.65	-1.51
ILMN_1349197	Cspg6	-1.07	-1.39	-1.64	-1.45	-1.71	-1.58	-1.51
ILMN_1367299	Adora1	1.1	-1.02	1.1	-1	-1.48	-1.57	-1.51
ILMN_1352860	Psip1	-1.1	-1.29	-1.49	-1.46	-1.51	-1.51	-1.51
ILMN_1354462	RGD1562550_predicted	-1.03	-1.53	-1.36	-1.74	-1.38	-1.5	-1.51
ILMN_1370664	Sh3bgrl_predicted	-1.13	-1.96	-1.4	-1.73	-1.63	-1.47	-1.51
ILMN_1357459	RGD1565523_predicted	1.06	-1.02	-1.13	1.14	-1.43	-1.3	-1.51
ILMN_1369752	Nr2f6	-1.14	1.37	1.09	1.26	-1.19	-1.27	-1.51
ILMN_1366231	Ugt2b10_predicted	1.05	-1.99	-1.4	-2.52	-1.79	-2.17	-1.5
ILMN_1358318	Pon2	-1.29	-2.59	-1.71	-2.77	-1.71	-2.1	-1.5
ILMN_1530349	Pon2	-1.29	-2.59	-1.71	-2.77	-1.71	-2.1	-1.5
ILMN_1364055	C1r	1.04	-1.45	-1.63	-1.94	-1.7	-2.01	-1.5
ILMN_1376257	Hnrpu	-1.14	-1.57	-1.53	-1.52	-1.97	-1.95	-1.5
ILMN_1367034	Prkag1	-1.27	-1.57	-1.49	-1.9	-1.93	-1.77	-1.5
ILMN_1361081	Lgr4	1.03	-1.22	-1.37	-1.36	-1.59	-1.66	-1.5
ILMN_1358787	Mss4	-1.25	-1.5	-1.66	-1.32	-1.54	-1.58	-1.5
ILMN_1368871	Nde1	-1.07	-1.24	-1.13	-1.36	-1.59	-1.57	-1.5
ILMN_1369151	RGD1310270_predicted	-1.25	1.03	-1.01	-1.22	-1.6	-1.56	-1.5
ILMN_1352534	Mta3_predicted	-1.03	-1.27	1.03	-1.41	-1.53	-1.56	-1.5
ILMN_1369650	Atp1b3	-1.02	-1.16	-1.14	-1.27	-1.64	-1.47	-1.5
ILMN_1363870	Rad51_predicted	-1.03	-1.32	-1.26	-1.23	-1.65	-1.41	-1.5
ILMN_1366297	Rpa3_predicted	1.03	-1.83	-2.15	-2.03	-1.38	-1.34	-1.5
ILMN_1360058	LOC293679	-1.09	-1.59	-1.53	-1.64	-1.55	-1.33	-1.5
ILMN_1369046	RGD1309370_predicted	-1.06	1.28	-1.3	1.25	-1.18	-1.33	-1.5
ILMN_1349379	Tbp	-1.04	-1.03	1.08	1.03	-1.25	-1.21	-1.5
ILMN_1366695	LOC678741	-1.01	-1.22	-1.44	-1.6	-1.32	-1.11	-1.5
ILMN_1363966	Carhsp1	-1.3	-1.68	-1.93	-1.73	-1.16	-1.07	-1.5
ILMN_1357697	LOC498611	-1.11	-1.06	-1.11	1	1.1	1.05	-1.5
ILMN_1361558	Pspla1	1.2	1.4	1.52	-1.03	-1.35	-1.75	-1.49
ILMN_1372689	RGD1566063_predicted	-1.01	-1.49	-1.43	-1.79	-1.58	-1.74	-1.49
ILMN_1376481	Btd	-1.26	-1.84	-2	-2.16	-1.41	-1.71	-1.49
ILMN_1357206	Cyc1_predicted	-1.13	-1.13	-1.02	-1.13	-1.57	-1.67	-1.49
ILMN_1349862	Bnip2_predicted	-1.15	-1.65	-1.62	-1.64	-1.83	-1.63	-1.49
ILMN_1354780	LOC360975	-1.1	-1.62	-1.72	-1.71	-1.54	-1.63	-1.49
ILMN_1358963	Ddx59	-1.23	-1.54	-1.48	-1.81	-1.51	-1.61	-1.49
ILMN_1355291	Bat5	-1.27	-1.43	-1.75	-1.85	-1.6	-1.59	-1.49
ILMN_1359470	Las1l_predicted	-1.3	-1.74	-1.32	-1.45	-1.61	-1.54	-1.49

ILMN_1358763	RGD1305689	-1.48	-2.28	-1.62	-2.19	-1.53	-1.41	-1.49
ILMN_1373766	RGD1307594	1.25	-1.16	-1.44	-2.32	-1.36	-1.38	-1.49
ILMN_1349008	H3f3b	-1.31	-1.44	-1.12	-1.73	-1.4	-1.32	-1.49
ILMN_1368551	Ccng2_predicted	1.21	-1.8	-1.4	-1.92	-1.45	-1.26	-1.49
ILMN_1358023	Smtn_predicted	-1.09	-1.24	-1.39	-1.08	-1.45	-1.26	-1.49
ILMN_1374269	LOC688785	-1.15	1.08	-1.16	-1.17	-1.18	-1.26	-1.49
ILMN_1363468	Oxnad1_predicted	1.01	-1.27	-1.18	-1.15	-1.09	-1.12	-1.49
ILMN_1650325	Cep76	1.44	1	-1.1	-1.05	-1.11	-1.05	-1.49
ILMN_1349146	Elovl5	1.16	-1.22	1.06	-1.01	-1.02	1.03	-1.49
ILMN_1355792	Abcb6	-1.11	-1.22	-1.4	-1.6	-1.94	-2.04	-1.48
ILMN_1371516	Akr7a3	1.3	-2.53	-2	-1.97	-2.17	-1.99	-1.48
ILMN_1352469	Irf7	-1.38	-1.73	-1.75	-2.08	-2	-1.95	-1.48
ILMN_1371187	Gpaa1	-1.16	-1.53	-1.01	-1.44	-1.58	-1.87	-1.48
ILMN_1358322	Adk	-1.1	-2.09	-1.74	-2.36	-1.78	-1.79	-1.48
ILMN_1354266	Thra	-1.2	-1.61	-1.64	-1.81	-1.64	-1.61	-1.48
ILMN_1350184	mrpl9	1.14	1.08	-1.17	-1.16	-1.45	-1.53	-1.48
ILMN_1353867	Mrps12_predicted	-1.1	-2	-2.33	-1.85	-1.77	-1.51	-1.48
ILMN_1356620	Smarcal1_predicted	-1.33	-1.6	-1.49	-1.35	-1.67	-1.48	-1.48
ILMN_1373492	Kpnb1	1.15	-1.25	-1.15	-1.09	-1.36	-1.44	-1.48
ILMN_1373313	Tnfrsf1a	-1.03	-1.02	-1.4	-1.1	-1.3	-1.4	-1.48
ILMN_1373342	Wrn_predicted	-1.15	-1.16	-1.35	-1.32	-1.47	-1.39	-1.48
ILMN_1352052	RGD1560666_predicted	1.08	-1.49	-1.22	-1.65	-1.53	-1.36	-1.48
ILMN_1355583	Bclaf1	-1.27	-1.25	-1.23	-1.83	-1.3	-1.36	-1.48
ILMN_1373128	Rcc2_predicted	1.13	-1.4	-1.07	-1.74	-1.37	-1.35	-1.48
ILMN_1363743	LOC501562	1.03	1.19	1.37	1.45	1.13	-1.22	-1.48
ILMN_1371026	Rab2l	-1.23	1.02	1.14	-1.04	-1.23	-1.21	-1.48
ILMN_1357358	Med4	-1.18	-1.55	-1.02	-1.38	-1.28	-1.17	-1.48
ILMN_1376942	Zfp313	-1.06	-1.1	-1.06	-1.24	-1.19	-1.16	-1.48
ILMN_1368793	Cdr2	-1.17	-1.43	-1.07	-1.17	-1.05	1.09	-1.48
ILMN_1376395	Mar-02	-1.35	-2.41	-2.02	-2.87	-1.54	-1.57	-1.47
ILMN_1358460	LOC287148	-1.14	-1.57	-1.2	-1.42	-1.68	-1.93	-1.47
ILMN_1361016	Agt	-1.3	-1.2	-1.23	-2.04	-1.71	-1.84	-1.47
ILMN_1355916	Cse1l_predicted	-1.08	-1.42	-1.32	-1.06	-1.8	-1.71	-1.47
ILMN_1360618	LOC682714	1.01	-1.63	-1.28	-1.33	-1.36	-1.71	-1.47
ILMN_1349140	RGD1308302	1.03	-1.07	-1.18	1.1	-1.43	-1.7	-1.47
ILMN_1373114	Cdk4	-1.18	-1.5	-1.2	-1.46	-1.69	-1.67	-1.47
ILMN_1353161	Atp5g2	-1.05	-1.17	-1.75	-1.54	-1.78	-1.65	-1.47
ILMN_1376607	Slc16a6	1.14	-1.14	1.09	-1.25	-1.42	-1.6	-1.47

ILMN_1650380	Foxa3	-1.2	-1.07	1.05	1.02	-1.31	-1.6	-1.47
ILMN_1360490	Myst2	-1	-1.31	-1.51	-1.64	-1.52	-1.55	-1.47
ILMN_1352586	Siahbp1	-1.15	-1.21	-1.52	-1.27	-1.64	-1.44	-1.47
ILMN_1369760	Cntrob_predicted	-1.1	-1.12	-1.14	-1.02	-1.17	-1.36	-1.47
ILMN_1373985	Itgb3bp	-1.18	-1.53	-1.71	-1.57	-1.3	-1.33	-1.47
ILMN_1359659	Mrpl50_predicted	-1.06	-1.3	-1.29	-1.27	-1.43	-1.32	-1.47
ILMN_1364469	Ctdspl_predicted	-1.14	-1.16	-1.71	-1.23	-1.51	-1.31	-1.47
ILMN_1361282	Luc7l2_predicted	1.07	-1.38	1.03	-1.21	-1.32	-1.28	-1.47
ILMN_1352900	Abtb2	-1.26	-1.34	-1.26	-1.02	1.02	-1.25	-1.47
ILMN_1351912	RGD1304868_predicted	-1.21	-1.38	-1.26	-1.23	-1.24	-1.22	-1.47
ILMN_1364020	RGD1306730	1.03	-1.28	-1.13	1.06	-1.4	-1.21	-1.47
ILMN_1365503	RGD1311362	-1.02	-1.02	1.03	1.07	-1.18	-1.2	-1.47
ILMN_1359824	Wiz_predicted	1.01	1.01	-1.16	1.21	1.01	1.12	-1.47
ILMN_1363648	Qprt	-1.07	1.05	-1.14	-1.26	-1.63	-2	-1.46
ILMN_1530394	Qprt	-1.07	1.05	-1.14	-1.26	-1.63	-2	-1.46
ILMN_1368406	RGD1310450	-1.04	-1.31	-1.29	-1.62	-1.61	-1.68	-1.46
ILMN_1650305	Pop7_predicted	-1.21	-1.29	-1.91	-1.44	-1.53	-1.63	-1.46
ILMN_1360769	Zyg11bl	-1.31	-1.56	-1.66	-1.88	-1.61	-1.61	-1.46
ILMN_1366264	Ctbp1	-1.21	-1.67	-1.4	-1.55	-1.64	-1.57	-1.46
ILMN_1376770	Fbxo9	-1.05	-1.37	-1.38	-1.36	-1.39	-1.49	-1.46
ILMN_1358795	LOC290396	1.09	-1.33	-1.32	-1.35	-1.64	-1.46	-1.46
ILMN_1356584	Pdhb	-1.16	-1.78	-1.64	-2.23	-1.71	-1.44	-1.46
ILMN_1357740	RGD1560636_predicted	-1.37	-1.42	-1.45	-1.49	-1.44	-1.43	-1.46
ILMN_1353641	Xpo1	1.19	-1.29	-1.1	1.02	-1.34	-1.38	-1.46
ILMN_1376338	Nup93	1.01	-1.29	-1.23	1.08	-1.25	-1.3	-1.46
ILMN_1356841	Atf1	1.14	-1.01	1.31	-1.02	-1.38	-1.1	-1.46
ILMN_1361614	Kcnj14	-1.19	-1.14	-1.16	-1.15	-1.33	-1.09	-1.46
ILMN_1367310	LOC500389	1.02	-1.09	-1.2	1.15	1.12	-1.08	-1.46
ILMN_1354482	Fxc1	1.11	-1.38	-1.65	-1.24	-1.44	-1.73	-1.45
ILMN_1373706	Fabp2	-1.12	-1.69	-1.82	-2.08	-1.31	-1.62	-1.45
ILMN_1376640	Cab39l	-1.17	-1.16	1.18	-1.36	-1.24	-1.51	-1.45
ILMN_1356512	Ap1g2_predicted	-1.33	-1.1	-1.25	-1.43	-1.58	-1.46	-1.45
ILMN_1357199	Nup155	1.02	-1.49	-1.33	-1.1	-1.57	-1.42	-1.45
ILMN_1371560	RGD1560544_predicted	-1.06	1.13	-1.24	-1.33	-1.84	-1.4	-1.45
ILMN_1370291	Ppa2_predicted	-1.03	-1.34	-1.57	-1.87	-1.39	-1.39	-1.45
ILMN_1367946	Baz1a_predicted	1.09	1.09	-1.14	1.24	-1.35	-1.39	-1.45
ILMN_1355472	Idh3g	-1.14	-1.15	-1.13	-1.28	-1.38	-1.38	-1.45
ILMN_1371143	Rab8a	-1.24	-1.34	-1.27	-1.64	-1.5	-1.37	-1.45

ILMN_1353517	Hagh	-1.1	-1.64	-1.56	-2.07	-1.18	-1.35	-1.45
ILMN_1367467	Per2	-1.06	1.69	-1.04	1.61	-1.15	-1.32	-1.45
ILMN_1365695	Dnaja1	-1.43	-1.17	-1.13	1.06	-1.3	-1.31	-1.45
ILMN_1353851	Hhex	-1.01	1.14	1.04	-1.05	-1.27	-1.28	-1.45
ILMN_1651010	Hspd1	-1.1	-1.3	-1.02	-1.22	-1.31	-1.24	-1.45
ILMN_1359063	Taf6l_predicted	1.09	-1.19	-1.15	1.08	-1.45	-1.23	-1.45
ILMN_1362743	RGD1309350_predicted	-1.42	-1.83	-2.62	-2.06	-1.61	-2.02	-1.44
ILMN_1369569	MGC108882	-1.23	-1.57	-1.32	-1.69	-1.79	-1.9	-1.44
ILMN_1352477	Lrrk1_predicted	-1.09	-1.29	-1.39	-1.84	-1.71	-1.85	-1.44
ILMN_1365702	Pank2_predicted	-1.08	-1.21	-1.19	-1.11	-1.53	-1.52	-1.44
ILMN_1374052	Pkp4_predicted	1.24	-1.2	-1.14	1	-1.44	-1.47	-1.44
ILMN_1350598	Ddx47	1.06	-1.16	-1.06	-1.24	-1.48	-1.45	-1.44
ILMN_1362836	Mk1	-1.13	-1.41	-1.57	-1.12	-1.29	-1.42	-1.44
ILMN_1363128	RGD1311624	-1.34	-1.14	-1.12	-1.09	-1.61	-1.36	-1.44
ILMN_1649758	RGD1304762	-1.23	-1.2	-1.38	-1.17	-1.51	-1.35	-1.44
ILMN_1362866	Svil_predicted	-1.11	-1.08	-1.35	-1.1	-1.36	-1.34	-1.44
ILMN_1349190	Deaf1	-1.08	-1.54	-1.36	-1.15	-1.57	-1.31	-1.44
ILMN_1356222	LOC500438	-1.05	-1.05	1.54	1.08	1.03	-1.31	-1.44
ILMN_1348850	Hnrpa2b1_predicted	-1.05	-1.72	1.05	-1.29	-1.31	-1.27	-1.44
ILMN_2040898	Aqp9	1.03	-1.09	-1.16	-1.68	-1.15	-1.27	-1.44
ILMN_1357560	RGD1308696	1.08	-1.32	-1.18	-1.31	-1.4	-1.25	-1.44
ILMN_1357820	Uhrf2_predicted	-1.05	-1.13	-1.29	-1.27	-1.44	-1.24	-1.44
ILMN_1367183	RGD1562567_predicted	-1.09	-1.19	1.02	1.09	-1.23	-1.18	-1.44
ILMN_1351960	LOC500506	-1.01	-1.62	-1.05	-1.39	-1.22	-1.17	-1.44
ILMN_1363458	Fmr1	-1.13	-1.31	-1.1	-1.34	-1.21	-1.15	-1.44
ILMN_1364100	Ap2m1	1.01	-1.45	-1.42	-1.36	-1.23	-1.14	-1.44
ILMN_1353144	RGD1562474_predicted	1.17	1.07	-1.03	1.14	-1.18	-1.1	-1.44
ILMN_1356146	Zfp384	-1.29	1.05	-1.1	1	-1.15	-1.06	-1.44
ILMN_1359479	Rbbp5_predicted	-1.04	-1.26	-1.2	1.02	1.16	1.09	-1.44
ILMN_1356381	RGD1305631_predicted	-1.22	-1.63	-2.01	-2.1	-1.72	-1.9	-1.43
ILMN_1350917	Cops3	-1.12	-1.23	-1.35	-1.42	-1.25	-1.77	-1.43
ILMN_1359567	RGD1310507	-1.09	-1.31	1.05	-1.58	-1.7	-1.66	-1.43
ILMN_1372211	Ela1	1.09	-1.58	-2.45	-1.69	-1.54	-1.64	-1.43
ILMN_1368767	Polg	-1.24	-1.18	-1.1	-1.15	-1.51	-1.63	-1.43
ILMN_1650923	Cyb5r1	1.04	-1.42	-1.29	-2.03	-1.59	-1.61	-1.43
ILMN_1354667	LOC497769	-1.16	-1.7	-2.02	-2.46	-1.6	-1.55	-1.43
ILMN_1349513	Xrcc6	1.01	-1.42	-1.37	-1.41	-1.31	-1.5	-1.43
ILMN_1352320	Msrb2	-1.02	-1.18	-1.19	-1.42	-1.28	-1.47	-1.43

ILMN_1360066	RGD1559901_predicted	-1.1	-1.59	-1.78	-1.53	-1.37	-1.42	-1.43
ILMN_1376451	Mapkapk2	1.06	-1.51	-1.98	-1.71	-1.3	-1.41	-1.43
ILMN_1352246	Lgals3bp	-1.28	-1.13	-1.25	-1.12	-1.34	-1.36	-1.43
ILMN_1354625	Rnf167	-1.39	-1.77	-1.27	-1.4	-1.55	-1.35	-1.43
ILMN_2038799	Actb	-1.06	-1.26	-1.04	-1.32	-1.49	-1.34	-1.43
ILMN_1355039	Actb	-1.06	-1.26	-1.04	-1.32	-1.49	-1.34	-1.43
ILMN_2038798	Actb	-1.06	-1.26	-1.04	-1.32	-1.49	-1.34	-1.43
ILMN_1364251	LOC366669	-1.06	-1.18	-1.15	-1.51	-1.34	-1.32	-1.43
ILMN_1365880	RGD1310712_predicted	-1.1	-1.3	-1.21	-1.41	-1.29	-1.3	-1.43
ILMN_1650663	Aim1l_predicted	-1.1	-1.09	-1.4	-1.27	-1.33	-1.29	-1.43
ILMN_1350267	Hcfc1_predicted	-1.05	-1.33	-1.37	-1.29	-1.31	-1.29	-1.43
ILMN_1376585	Rnps1	-1.07	-1.06	-1	1.07	-1.29	-1.29	-1.43
ILMN_1358552	Pafah1b2	-1.21	-1.22	1.04	-1.24	-1.47	-1.28	-1.43
ILMN_1374123	MGC108896	-1.1	-1.23	1	-1.29	-1.23	-1.28	-1.43
ILMN_1366467	Dlx1	-1.02	1.05	-1.09	-1	-1.43	-1.18	-1.43
ILMN_1352176	Cggbp1_predicted	1.02	-1.16	-1.48	-1.19	-1.26	-1.18	-1.43
ILMN_1364558	Tle3	-1.12	1.25	1.1	-1.14	-1.08	-1.12	-1.43
ILMN_1649922	LOC684872	1.17	-1.23	-1.34	-1.28	-1.44	1	-1.43
ILMN_1650174	RGD1311298_predicted	-1.11	-1.52	1.15	-1.93	-1.15	1.01	-1.43
ILMN_1350345	LOC499638	1.01	-1.12	-1.47	-1.07	1.1	1.09	-1.43
ILMN_1364802	RGD1559459_predicted	1.07	-1.97	-1.49	-2.2	-1.94	-2.3	-1.42
ILMN_1358639	Kptn_predicted	-1.32	-1.47	-1.41	-1.61	-1.85	-2.03	-1.42
ILMN_1348859	Gle1l	-1.3	-1.37	-1.31	-2.07	-1.96	-1.87	-1.42
ILMN_1355842	Pdk2	-1.2	-1.69	-1.59	-1.89	-1.83	-1.81	-1.42
ILMN_1359720	RGD1307700	-1.19	-1.47	-1.66	-1.41	-1.66	-1.63	-1.42
ILMN_1350232	Phyh2	-1.37	-1.71	-1.91	-2.77	-1.57	-1.63	-1.42
ILMN_1367491	Nupl1	1.05	-1.16	-1.07	-1.07	-1.49	-1.56	-1.42
ILMN_1361874	Rabep2	-1.32	-1.45	-1.54	-1.5	-1.47	-1.52	-1.42
ILMN_1370978	Hnrpl	-1.06	-1.63	-1.34	-1.74	-1.77	-1.5	-1.42
ILMN_1362327	Tgm2	-1.11	-1.21	-1.38	-1.14	-1.14	-1.5	-1.42
ILMN_1350501	RGD1566014_predicted	-1.29	-1.29	-1.37	-1.38	-1.95	-1.49	-1.42
ILMN_1371638	Lmo4	-1.46	-1.17	-1.12	-1.35	-1.31	-1.44	-1.42
ILMN_1369962	RGD1311892_predicted	-1.01	-1.06	-1.07	-1.21	-1.2	-1.4	-1.42
ILMN_1358174	Mrps5_predicted	-1.1	-1.34	-1.13	-1.38	-1.42	-1.39	-1.42
ILMN_1360147	Ncl	-1.02	1.04	1.26	1.16	-1.59	-1.37	-1.42
ILMN_1349478	Slc39a3	-1.1	-1.19	-1.08	-1.63	-1.54	-1.36	-1.42
ILMN_1373747	Myo1b	-1.04	-1.08	1.07	-1.12	-1.3	-1.31	-1.42
ILMN_1349585	RGD1305094_predicted	1.02	1.09	-1	1.13	-1.33	-1.29	-1.42

ILMN_1373780	Aven_predicted	-1	1.01	-1.22	-1.25	-1.51	-1.23	-1.42
ILMN_1371331	Fnbp4	-1.52	-1.08	1.05	-1.08	-1.26	-1.22	-1.42
ILMN_1349906	Casp2	-1.08	-1.35	-1.31	-1.49	-1.66	-1.18	-1.42
ILMN_1350151	Slc30a2	1.07	-1.12	-1.13	-1.03	-1.35	-1.18	-1.42
ILMN_1352197	RGD1306487_predicted	-1.31	-1.63	-1.17	-1.84	-1.31	-1.16	-1.42
ILMN_1352780	LOC317418	1.07	1	1.48	-1.01	-1.14	-1.16	-1.42
ILMN_1363052	Pcgf6	-1.07	1.2	1.08	1.17	-1.25	-1.11	-1.42
ILMN_1365113	RGD1564649_predicted	-1.06	-1.05	-1.1	1.09	-1.01	-1.09	-1.42
ILMN_1369171	RGD1562337_predicted	1.07	-1.21	1.41	-1.28	-1.15	-1.08	-1.42
ILMN_1359570	Slc35c1_predicted	-1.03	-1.08	-1.35	1.15	-1.05	-1.07	-1.42
ILMN_1355237	Cxcl1	-1.9	-1.32	-1.7	-1.53	1.64	1.69	-1.42
ILMN_1363985	Bat1a	-1.23	-1.14	-1.05	-1.16	-1.43	-1.63	-1.41
ILMN_1369659	LOC317380	-1.25	-1.3	-1.47	-1.63	-1.69	-1.49	-1.41
ILMN_1373194	Mtr	-1.01	-1.2	-1.69	-1.33	-1.49	-1.47	-1.41
ILMN_1364344	Saps3_predicted	-1.03	-1.36	-1.49	-1.57	-1.43	-1.45	-1.41
ILMN_1650701	Abca1	-1.23	-1.27	-1.68	-1.21	-1.39	-1.42	-1.41
ILMN_1373561	Ercc3	-1.29	-1.46	-1.47	-1.34	-1.55	-1.37	-1.41
ILMN_1366421	Brd8	-1.2	-1.36	-1.44	-1.76	-1.56	-1.36	-1.41
ILMN_1373466	Map3k6_predicted	-1.02	-1.09	-1.04	-1.08	-1.18	-1.36	-1.41
ILMN_1374063	Plxnb1_predicted	-1.11	-1.02	1.02	-1.12	-1.3	-1.34	-1.41
ILMN_1363064	Phc1_predicted	-1.19	-1.32	1.02	-1.11	-1.1	-1.32	-1.41
ILMN_1355259	LOC362732	-1.04	-1.28	-1.23	-1.43	-1.12	-1.29	-1.41
ILMN_1354025	Acp6	-1.03	-1.18	-1.2	-1.27	-1.29	-1.28	-1.41
ILMN_1362247	Bpgm	-1.12	-1.27	-1.18	-1.36	-1.3	-1.25	-1.41
ILMN_1361231	Lepre1	-1.13	-1.47	1.09	-1.52	-1.29	-1.23	-1.41
ILMN_1355011	Magoh_predicted	-1.06	-1.32	-1.77	-1.29	-1.15	-1.21	-1.41
ILMN_1376700	Elf2	-1.1	-1.12	-1.26	-1.26	-1.26	-1.17	-1.41
ILMN_1375290	Elf2	-1.1	-1.12	-1.26	-1.26	-1.26	-1.17	-1.41
ILMN_1351618	Rpp21	1.07	-1.15	-2.17	-1	-1.28	-1.16	-1.41
ILMN_1374190	LOC499733	-1.02	1.03	1.42	1.27	1.17	-1.1	-1.41
ILMN_1352290	Pold3	-1.01	-1	1.03	-1.06	-1.17	-1.09	-1.41
ILMN_1349390	Rbm4_predicted	-1.19	-1.19	-1.27	-1.17	-1.16	-1.03	-1.41
ILMN_1366485	RGD1359529	-1.32	1.02	1.14	-1.06	-1.22	-1	-1.41
ILMN_1356637	Rhebl1	1.4	1.18	1.34	1.07	-1.39	1.01	-1.41
ILMN_1366203	Selenbp1	-1.27	-1.8	-2.99	-3.47	-2.87	-2.95	-1.4
ILMN_1373267	Echs1	-1.26	-2.01	-1.6	-2.41	-2.29	-2.35	-1.4
ILMN_1530387	Nipsnap3a	1.01	-1.29	-1.5	-1.54	-1.64	-1.75	-1.4
ILMN_1368304	Nipsnap3a	1.01	-1.29	-1.5	-1.54	-1.64	-1.75	-1.4

ILMN_1360071	RGD1307673_predicted	-1.08	-1.33	-1.54	-1.71	-1.53	-1.64	-1.4
ILMN_1361854	Serbp1	-1.11	-1.19	-1.06	-1.23	-1.54	-1.46	-1.4
ILMN_1371248	LOC362414	-1.24	-1.3	-1.2	-1.54	-1.48	-1.46	-1.4
ILMN_1366832	Slc17a2_predicted	1.02	-1.2	-1.38	-1.61	-1.93	-1.45	-1.4
ILMN_2040226	Anp32e	-1.05	-1.4	-1.08	-1.25	-1.79	-1.44	-1.4
ILMN_1363755	Anp32b	-1.2	-1.27	-1.14	-1.17	-1.46	-1.44	-1.4
ILMN_1366762	Slc30a6_predicted	-1.04	-1.58	-1.21	-1.39	-1.25	-1.42	-1.4
ILMN_1367031	LOC290341	-1.15	-1.38	1.31	-1.2	-1.19	-1.42	-1.4
ILMN_1376310	Rangap1	-1.07	-1.48	1.13	-1.15	-1.6	-1.41	-1.4
ILMN_1358624	Papss2_predicted	1.11	-1.33	-1.1	-1.29	-1.31	-1.38	-1.4
ILMN_1369091	RGD1564876_predicted	1.04	-1.43	-1.21	-1.15	-1.33	-1.31	-1.4
ILMN_1649991	Akap9	-1.18	-1.14	-1.26	-1.28	-1.31	-1.31	-1.4
ILMN_1351553	LOC682593	-1.23	-1.55	-1.62	-2.1	-1.29	-1.27	-1.4
ILMN_1373756	LOC682593	-1.23	-1.55	-1.62	-2.1	-1.29	-1.27	-1.4
ILMN_1374759	LOC360807	-1.19	-1.16	-1.05	-1.18	-1.06	-1.16	-1.4
ILMN_1363012	Rdm1_predicted	1.13	1.25	1.36	1.15	-1.37	-1.15	-1.4
ILMN_1362140	Ap4m1	-1.28	1.27	1.04	-1.01	-1.3	-1.15	-1.4
ILMN_1362242	LOC501280	-1	-1.46	1.26	-1.13	-1.26	-1.15	-1.4
ILMN_1359055	LOC501280	-1	-1.46	1.26	-1.13	-1.26	-1.15	-1.4
ILMN_1376465	Naga	-1.17	-1.15	-1.28	-1.33	1.14	-1.09	-1.4
ILMN_1358745	Cbx1_predicted	-1.08	-1.09	-1.07	1.07	-1.04	-1.08	-1.4
ILMN_1350510	Eed_predicted	1.05	-1.05	1.01	1.22	-1.11	-1.05	-1.4
ILMN_1369034	Crkl	-1.12	-1.2	-1.09	-1.15	1.19	1.12	-1.4
ILMN_1349469	LOC494499	1.08	-1.51	-2.35	-2.06	-2.34	-2.44	-1.39
ILMN_1365225	Ap1m1	-1.11	-1.34	-1.43	-1.53	-2.06	-1.93	-1.39
ILMN_1366364	Thpo	-1.47	-1.2	-1.34	-1.34	-1.77	-1.82	-1.39
ILMN_1365255	Enpp1	-1.05	1.08	1.02	-1.47	-1.76	-1.73	-1.39
ILMN_1649963	Tpm3	-1.09	-1.7	-1.34	-1.44	-1.71	-1.73	-1.39
ILMN_1363774	Tpm3	-1.09	-1.7	-1.34	-1.44	-1.71	-1.73	-1.39
ILMN_1355457	Mucdhl	1.09	-1.55	-1.8	-1.8	-1.56	-1.72	-1.39
ILMN_1649996	Pmpca	-1.08	-1.3	1.02	-1.17	-1.64	-1.69	-1.39
ILMN_1349693	LOC288514	-1.1	-1.36	-1.33	-1.3	-1.76	-1.53	-1.39
ILMN_1349629	Ctnnal1_predicted	-1.04	-1.46	-1.44	-1.39	-1.62	-1.51	-1.39
ILMN_1363496	Tspan8	-1.14	-1.75	-1.56	-1.98	-1.31	-1.51	-1.39
ILMN_1359731	Vps52	-1.19	-1.22	1.29	-1.26	-1.81	-1.5	-1.39
ILMN_1374681	Zfp499_predicted	-1.12	-1.1	-1.42	-1.27	-1.35	-1.49	-1.39
ILMN_1367500	Sass6_predicted	1.02	-1.36	-1.17	-1.55	-1.81	-1.42	-1.39
ILMN_1359900	RGD1311900_predicted	-1.12	-1.23	-1.08	1.08	-1.43	-1.4	-1.39

ILMN_1349057	Cdc25a	1.05	-1.07	-1.04	1.23	-1.25	-1.3	-1.39
ILMN_1374453	Srrm1_predicted	-1.39	-1.19	-1.24	-1.26	-1.24	-1.3	-1.39
ILMN_1371149	RGD1562456_predicted	1.01	-1.17	1.12	-1.18	1.03	-1.3	-1.39
ILMN_1369571	Pole4_predicted	-1.15	-1.26	-1.08	-1.33	-1.35	-1.28	-1.39
ILMN_1351557	Tmem9_predicted	-1.04	-1.08	-1.21	-1.04	-1.19	-1.27	-1.39
ILMN_1357028	Eftud2	-1.03	-1.35	1.04	-1.22	-1.37	-1.26	-1.39
ILMN_1355481	Daxx	1.08	-1.3	-1.32	-1.19	-1.16	-1.25	-1.39
ILMN_1376773	Slc19a1	1.05	1.41	1.62	1.76	-1.23	-1.16	-1.39
ILMN_1361348	Slc39a5_predicted	-1.17	-1.1	1.08	-1.05	-1.07	-1.07	-1.39
ILMN_1369123	Ubqln4_predicted	1	-1.14	-1.19	-1.15	-1.16	-1.05	-1.39
ILMN_1362533	Cdkn1b	-1.22	-1.11	-1.46	-1.42	1.09	-1.05	-1.39
ILMN_1360390	Cdc14a_predicted	1.06	-1.19	-1.08	-1.25	-1.07	-1.04	-1.39
ILMN_2039861	LOC498245	-1.04	-1.02	-1.22	1.07	1.23	1.23	-1.39
ILMN_1360676	RGD1565542_predicted	-1.15	-1.44	-3.61	-1.71	-1.5	-2.28	-1.38
ILMN_1370877	RGD1306762_predicted	-1.06	-1.05	-1.48	-1.47	-1.46	-1.75	-1.38
ILMN_1360448	RGD1309198_predicted	-1.08	-1.64	-1.36	-1.32	-1.32	-1.66	-1.38
ILMN_1356909	Fgg	-1.08	-1.66	-1.5	-1.82	-1.53	-1.64	-1.38
ILMN_2040131	Tst	-1.08	-1.02	-1.11	-1.28	-1.5	-1.62	-1.38
ILMN_1372903	Mppe1_predicted	-1.31	-1.55	-1.38	-1.7	-1.75	-1.6	-1.38
ILMN_1369194	LOC498122	-1.12	-1.25	-1.51	-1.13	-1.5	-1.6	-1.38
ILMN_1365843	LOC687055	-1.16	-1.61	-1.13	-1.78	-1.74	-1.54	-1.38
ILMN_1369924	Smarcb1	-1.07	-1.02	-1.07	1.01	-1.46	-1.45	-1.38
ILMN_1362800	Pola1	1.15	-1.3	-1.42	-1.29	-1.41	-1.41	-1.38
ILMN_1359444	RGD1561639_predicted	-1.22	1.31	1.11	1.17	-1.36	-1.39	-1.38
ILMN_1356820	RGD1308048_predicted	1.1	-1.39	-1.05	-1.3	-1.49	-1.37	-1.38
ILMN_1376323	Ang1	-1.4	-1.61	-1.57	-1.94	-1.25	-1.36	-1.38
ILMN_1362413	RGD1309019_predicted	-1.28	-1.42	-1.34	-1.62	-1.42	-1.34	-1.38
ILMN_1359089	Coq3	1.06	-1.19	-1.09	-1.11	-1.16	-1.32	-1.38
ILMN_1375082	Sirt4_predicted	1.23	-1.12	1.03	-1.26	-1.44	-1.25	-1.38
ILMN_1374605	RGD1563167_predicted	1.19	-1.43	-1.6	-1.65	-1.05	-1.2	-1.38
ILMN_1361253	Ndufb11_predicted	1.06	-1.2	-1.7	-1.23	-1.22	-1.19	-1.38
ILMN_1372721	Eif4b	1.05	1.19	-1.8	-1.39	-1.4	-1.14	-1.38
ILMN_1359735	Snrp70_predicted	-1.04	1.07	-1.13	1.1	-1.18	-1.13	-1.38
ILMN_1348994	Ppp1r8_predicted	1.11	-1.02	1.19	1.02	-1.03	-1.13	-1.38
ILMN_1370050	Csrp1	1.01	-1.4	-1.21	-1.36	-1.23	-1.09	-1.38
ILMN_1369946	Zfp238	1.13	1.33	1.47	1.56	-1.04	-1.04	-1.38
ILMN_1375729	Mar-02	-1.36	-2.68	-2.11	-3.17	-1.60	-1.55	-1.38
ILMN_1376249	Tf	-1.16	-1.46	-1.37	-2.03	-1.61	-1.94	-1.37

ILMN_1370368	Pom210	1.03	-1.1	-1.07	1.05	-1.99	-1.62	-1.37
ILMN_1650719	Tmem106a	1	-1.05	-1.09	-1.06	-1.12	-1.56	-1.37
ILMN_1351005	Slc7a2	-1.15	-1.1	-1.56	-1.74	-1.92	-1.51	-1.37
ILMN_1367706	RGD1563715_predicted	-1.15	-1.18	-1.25	-1.18	-1.49	-1.5	-1.37
ILMN_1374246	LOC500614	1.06	-1.49	-1.28	-1.42	-1.48	-1.43	-1.37
ILMN_1374733	Ckap5	-1.02	1.04	-1.38	1.03	-1.36	-1.42	-1.37
ILMN_1374549	Rfx1_predicted	-1.12	1.12	-1.44	-1.13	-1.7	-1.39	-1.37
ILMN_1369424	Napg	-1.06	-1.16	-1.22	-1.28	-1.5	-1.39	-1.37
ILMN_1371522	LOC498918	-1.29	-1.14	-1.44	-1.22	-1.54	-1.36	-1.37
ILMN_1364330	Rad23a	1.03	-1.3	-1.02	-1.62	-1.39	-1.36	-1.37
ILMN_1354721	LOC503000	-1.3	-1.21	-1.12	-1.18	-1.34	-1.34	-1.37
ILMN_1351305	Ndufa11	-1.01	-1.42	-2.29	-1.41	-1.3	-1.34	-1.37
ILMN_1368469	Ddx20	1	1.11	-1.04	1.28	-1.24	-1.28	-1.37
ILMN_1351052	Ppm1g	-1.03	-1.36	1.19	-1.2	-1.3	-1.26	-1.37
ILMN_1357266	Mesdc2	-1.04	-1.6	-1.44	-1.87	-1.5	-1.25	-1.37
ILMN_1359938	RGD1308759_predicted	-1.03	-1.25	-1.42	-1.39	-1.42	-1.25	-1.37
ILMN_1357714	Ikbkb	-1.17	-1.37	-1.41	-1.47	-1.2	-1.2	-1.37
ILMN_1356570	Rqcd1	-1.02	-1.26	-1.22	-1.13	-1.15	-1.14	-1.37
ILMN_1373588	LOC499427	-1.01	-1.25	-1.06	-1.31	-1.16	-1.13	-1.37
ILMN_1375168	Mdfic_predicted	-1.04	-1.09	-1.13	-1.19	-1.22	-1.1	-1.37
ILMN_1355902	Dolpp1_predicted	1	-1.17	-1.02	-1.1	-1.07	-1.06	-1.37
ILMN_1364680	Mapre1	-1.03	-1.17	1.6	1.04	-1.14	-1.02	-1.37
ILMN_1362474	LOC501281	-1.12	1.02	-1.1	1.03	-1.15	1.01	-1.37
ILMN_1351362	Mrpl34	-1.11	-1.41	-1.86	-1.34	-1.75	-1.92	-1.36
ILMN_1353442	Sema4g_predicted	-1.45	-1.3	-1.59	-1.62	-1.57	-1.92	-1.36
ILMN_1372024	Htatsf1_predicted	-1.46	-1.72	-1.76	-1.66	-1.97	-1.76	-1.36
ILMN_1361035	Pdxk	1.03	-1.25	-1.26	-1.23	-1.48	-1.65	-1.36
ILMN_1362897	Acadm	-1.26	-1.59	-1.79	-2.29	-1.45	-1.64	-1.36
ILMN_1368463	Np_mapped	-1.14	-1.99	-1.68	-2.74	-1.51	-1.62	-1.36
ILMN_1365662	Mccc1	-1.23	-1.19	-1.28	-1.54	-1.3	-1.57	-1.36
ILMN_1361783	LOC362855	-1.14	-1.21	-1.07	-1.31	-1.46	-1.49	-1.36
ILMN_1358668	Ddt	-1.04	1.18	-1.26	-1.04	-1.27	-1.46	-1.36
ILMN_1357942	RGD1565934_predicted	-1.11	-1.37	-1.37	-1.12	-1.65	-1.45	-1.36
ILMN_1349982	RGD1306947	1.08	1.16	1.2	1.17	-1.36	-1.38	-1.36
ILMN_1359586	Scd2	-1.11	-1.62	-1.68	-1.84	-1.32	-1.33	-1.36
ILMN_1370863	LOC497876	-1.07	-1.55	-1.1	-1.13	-1.31	-1.33	-1.36
ILMN_1349816	RGD1559600_predicted	-1.04	-1.12	-1.27	-1.47	-1.09	-1.31	-1.36
ILMN_1356929	RGD1304881_predicted	-1.17	-1.21	-1.25	-1.07	-1.53	-1.3	-1.36

ILMN_1364151	Srd5a1	-1.31	-1.25	-1.26	-1.25	-1.32	-1.3	-1.36
ILMN_1350274	Efna4_predicted	-1.1	1.07	-1.2	-1.22	-1.15	-1.29	-1.36
ILMN_1357257	Ccdc52	-1.09	-1.18	-1.56	-1.29	-1.49	-1.27	-1.36
ILMN_1367476	LOC501326	-1.02	-1.3	1.42	-1.12	-1.42	-1.26	-1.36
ILMN_1364653	Banf1	-1.01	-1.25	-1.43	-1.2	-1.31	-1.26	-1.36
ILMN_1368735	RGD1311103_predicted	-1.16	-1.28	-1.43	-1.57	-1.34	-1.22	-1.36
ILMN_1360749	LOC500247	-1.4	-1.46	-1.63	-1.84	-1.39	-1.21	-1.36
ILMN_1376701	Cbx6	-1.05	-1.04	-1.18	-1.12	-1.16	-1.18	-1.36
ILMN_1348930	RGD1563437_predicted	-1.1	-1.32	-1.55	-1.71	-1.25	-1.14	-1.36
ILMN_1366851	Ifngr	-1.23	-1.4	-1.18	-1.57	-1.15	-1.1	-1.36
ILMN_1364125	Dnmt3b	1.17	1.43	1.54	1.98	-1.25	-1.09	-1.36
ILMN_1356257	Rchy1	-1.21	-1.13	-1.17	-1.37	-1.1	-1.04	-1.36
ILMN_1352355	LOC497920	1.12	-1.1	-2	-1.39	1	-1.01	-1.36
ILMN_1359349	Hrmt1l2	-1.09	-1.19	1.03	1.02	-1.55	-1.54	-1.35
ILMN_1367329	Ppp1r16a_predicted	-1.32	-1.15	-1.32	-1.1	-1.34	-1.53	-1.35
ILMN_1357397	Tsn	-1.09	-1.41	-1.56	-1.32	-1.21	-1.52	-1.35
ILMN_1349832	Arhgap18_predicted	-1	-1.44	-1.66	-2.26	-1.45	-1.47	-1.35
ILMN_1359148	Ogg1	-1.06	-1.22	-1.3	-1.06	-1.42	-1.46	-1.35
ILMN_1352417	Brp16	1.09	1.13	1.33	1.2	-1.69	-1.45	-1.35
ILMN_1355138	Cln6_predicted	1.19	1.21	-1.13	-1.47	-1.28	-1.44	-1.35
ILMN_1356233	Hnrpd	-1.09	-1.3	-1.08	-1.44	-1.4	-1.43	-1.35
ILMN_1365112	Ruvbl1	-1.02	-1.1	1.02	1.05	-1.59	-1.34	-1.35
ILMN_1364265	Pole3	-1.17	-1.59	-1.42	-1.26	-1.43	-1.33	-1.35
ILMN_1351196	Rabl4_predicted	-1.13	-1.67	-1.76	-1.67	-1.38	-1.33	-1.35
ILMN_1352511	RGD1562033_predicted	-1.04	-1.32	-1.42	-1.22	-1.25	-1.29	-1.35
ILMN_1355763	Eif2b4	-1.01	-1.06	1.05	1.03	-1.46	-1.27	-1.35
ILMN_1361265	Xrcc5	-1.01	-1.15	1.04	-1.13	-1.26	-1.27	-1.35
ILMN_1349219	Prkaca	-1.03	-1.53	-1.5	-1.41	-1.11	-1.26	-1.35
ILMN_1361777	Man2b1	-1.2	-1.02	-1.08	-1.01	-1.18	-1.24	-1.35
ILMN_1361212	Itgb1bp2_predicted	-1.03	-1.11	1.02	-1	-1.16	-1.21	-1.35
ILMN_1374693	LOC362264	-1.2	-1.14	1.11	-1.23	-1.19	-1.2	-1.35
ILMN_1356804	Sf3a1_predicted	-1.22	-1.11	-1.54	-1.07	-1.24	-1.19	-1.35
ILMN_1358630	Nfyc	1.05	-1.11	-1.08	-1.04	-1.23	-1.16	-1.35
ILMN_1350240	RGD1311358	-1.07	-1.29	-1.05	-1.32	-1.56	-1.15	-1.35
ILMN_1359331	LOC687248	1.01	-1.37	-1.06	-1.25	-1.26	-1.12	-1.35
ILMN_1350977	Ppfibp1_predicted	1.06	-1.04	1.35	-1.06	-1.1	-1.1	-1.35
ILMN_1366358	Nup54	1.1	1.26	1.41	1.4	1.08	1.23	-1.35
ILMN_1367573	Slc25a25	-1.2	1.6	1	1.31	1.79	1.53	-1.35

ILMN_1367159	RGD1561422_predicted	-1.19	-1.43	-1.2	-1.46	-1.61	-1.73	-1.34
ILMN_1361915	Pde10a	-1.01	-1.09	-1.22	-1.29	-1.56	-1.61	-1.34
ILMN_1368811	RGD1560924_predicted	-1.06	-1.26	-1.18	1.02	-1.69	-1.6	-1.34
ILMN_2039260	RGD1565407_predicted	-1.16	-1.48	-1.37	-1.33	-1.49	-1.56	-1.34
ILMN_2039010	RGD1565407_predicted	-1.16	-1.48	-1.37	-1.33	-1.49	-1.56	-1.34
ILMN_1355396	Txnl5_predicted	-1.17	-1.4	-2	-1.54	-1.39	-1.56	-1.34
ILMN_1356229	Ndufa12_predicted	-1.03	-1.73	-2.15	-1.72	-1.42	-1.53	-1.34
ILMN_1373568	LOC368001	1.05	-1.38	1.16	-1.23	-1.24	-1.49	-1.34
ILMN_1371435	Casp9	-1.2	-1.6	-1.52	-1.26	-1.37	-1.47	-1.34
ILMN_1359879	Thrb	-1.13	-1.28	-1.11	-1.25	-1.42	-1.42	-1.34
ILMN_1362515	RGD1309892	-1.14	-1.14	-1.35	-1.45	-1.5	-1.41	-1.34
ILMN_1367199	LOC687796	-1.19	-1.27	-1.33	-1.59	-1.46	-1.38	-1.34
ILMN_1351135	RGD1309929	1.03	1.05	1.14	1.02	-1.26	-1.38	-1.34
ILMN_1367145	Aprin_predicted	-1.06	-1.05	-1.09	-1.14	-1.38	-1.34	-1.34
ILMN_1374002	Txnrd2	-1.08	-1.33	-1.43	-1.62	-1.41	-1.3	-1.34
ILMN_1356258	Myo9b	-1.28	-1.21	-1.28	-1.06	-1.32	-1.3	-1.34
ILMN_1370649	Osbpl6_predicted	1.08	1.15	-1.11	-1.09	-1.24	-1.28	-1.34
ILMN_1365454	LOC680831	-1.08	-1.45	1.49	-1.44	-1.46	-1.26	-1.34
ILMN_1367191	Cic_predicted	-1.28	-1.19	-1.78	-1.36	-1.16	-1.25	-1.34
ILMN_1363010	Tubgcp3	-1.09	-1.21	-1.14	-1.14	-1.27	-1.24	-1.34
ILMN_1373913	Ywhaz	-1.2	-1.15	-1.07	-1.34	-1.31	-1.21	-1.34
ILMN_1352258	Os-9	-1.2	-1.22	-1.51	-1.47	-1.1	-1.21	-1.34
ILMN_1374766	Eif3s8	1.02	-1.03	1.04	1.06	-1.02	-1.09	-1.34
ILMN_1375869	Fbxo8_predicted	1.12	-1.6	-1.6	-2.05	1.08	-1.04	-1.34
ILMN_1366960	LOC500397	1.02	-1	-1.04	1.1	1.07	1.02	-1.34
ILMN_1352994	RGD1565988_predicted	-1.06	-1.21	1.03	-1.2	-1.58	-1.79	-1.33
ILMN_1351709	Ndufs7	-1.03	-1.28	-1.35	-1.28	-1.52	-1.6	-1.33
ILMN_1364053	Slc27a1	-1.4	-1.41	-1.43	-1.72	-1.41	-1.53	-1.33
ILMN_1358895	Nup88	-1.15	-1.25	-1.27	-1.25	-1.29	-1.39	-1.33
ILMN_2040754	Ran	-1.04	-1.23	-1.07	-1.17	-1.35	-1.36	-1.33
ILMN_1357005	Cbfb	-1.24	-1.4	-1.27	-1.34	-1.27	-1.36	-1.33
ILMN_1650496	RGD1305441	-1.11	-1.25	-1.37	1.03	-1.36	-1.35	-1.33
ILMN_1374940	RGD1307390_predicted	-1.29	-1.38	-1.17	-1.21	-1.27	-1.35	-1.33
ILMN_1357666	Xpo7	1.02	-1.06	-1.18	-1.17	-1.42	-1.34	-1.33
ILMN_1370036	Gripap1	-1.43	-1.22	-1.19	-1.07	-1.49	-1.32	-1.33
ILMN_1355251	Ccni_predicted	-1.21	-1.33	-1.22	-1.58	-1.43	-1.32	-1.33
ILMN_1650272	Scrn3_predicted	-1.08	-1.48	-1.2	-1.52	-1.28	-1.32	-1.33
ILMN_1354867	Nt5c3_predicted	-1.17	-1.16	-1.09	-1.24	-1.37	-1.31	-1.33

ILMN_1369541	Tcf4	-1.2	1.22	-1.15	-1.31	-1.23	-1.31	-1.33
ILMN_1366120	Ewsr1	-1.18	-1.17	-1.17	-1.31	-1.37	-1.29	-1.33
ILMN_1354229	RGD1305453	-1.14	-1.13	-1.04	-1.26	-1.29	-1.29	-1.33
ILMN_1366549	Gpd2	-1.16	-1.27	-1.04	-1.12	-1.39	-1.28	-1.33
ILMN_1355224	Stk35_predicted	-1.05	1.05	-1.14	1.01	-1.32	-1.28	-1.33
ILMN_1349267	RGD1562206_predicted	-1.24	-1.4	-1.47	-1.43	-1.24	-1.28	-1.33
ILMN_1354490	LOC246263	1.01	-1.44	-1.24	-1.37	-1.24	-1.25	-1.33
ILMN_1352632	Phc2	-1.24	-1.1	-1.44	-1.28	-1.22	-1.21	-1.33
ILMN_1351609	Atf7ip_predicted	-1.18	-1.15	-2.02	-1.54	-1.28	-1.2	-1.33
ILMN_1355189	Nr0b2	-1.15	-1.26	-1.45	-1.51	-1.36	-1.19	-1.33
ILMN_1364814	LOC361841	1.02	-1.91	1.04	-1.28	-1.03	-1.19	-1.33
ILMN_1370471	Cdc91l1	-1.01	-1.36	-1.38	-1.38	-1.31	-1.18	-1.33
ILMN_1365940	Spnb2	-1.09	-1.06	1.13	-1.22	-1.14	-1.17	-1.33
ILMN_1650052	Pcbp2	-1	-1.02	-1.24	-1.07	-1.3	-1.12	-1.33
ILMN_1364510	Eef1d	-1.05	1.01	1.26	1.01	-1.24	-1.12	-1.33
ILMN_1365857	Suv420h1_predicted	-1.14	1.12	-1.03	-1.31	-1.09	-1.12	-1.33
ILMN_1349900	RGD1565310_predicted	-1.09	-1.14	-1.03	-1.16	-1.17	-1.11	-1.33
ILMN_1372992	Sp3	1.18	1.01	1.01	-1.08	1.01	-1.09	-1.33
ILMN_1374735	RGD1311499_predicted	-1.02	-1.17	-1.01	-1.03	-1.03	-1.05	-1.33
ILMN_1364535	Rnpc2	-1.14	-1.13	-1.18	-1.21	-1.01	1.05	-1.33
ILMN_1650979	Phf13_predicted	-1.26	-1.2	-1.17	-1.12	1.24	1.16	-1.33
ILMN_1364831	Limd1_predicted	-1.02	1.39	1.33	1.3	1.17	1.2	-1.33
ILMN_1361581	Sod3	-1.26	-1.84	-1.58	-2	-2.08	-2.04	-1.32
ILMN_1370821	RGD1565002_predicted	-1.35	-1.57	-1.84	-2.3	-1.67	-1.88	-1.32
ILMN_1358222	LOC360800	-1.15	-1.24	-1.16	-1.53	-1.63	-1.73	-1.32
ILMN_1357122	Ddah1	1.06	-1.21	-1.25	-1.17	-1.42	-1.6	-1.32
ILMN_1357489	LOC499625	1.23	-1.35	-1.01	-1.31	-1.35	-1.58	-1.32
ILMN_1373279	RGD1305133	-1.06	-1.26	-1.2	-1.07	-1.65	-1.57	-1.32
ILMN_1354106	Dbi	1.03	-1.62	-2.07	-1.55	-1.31	-1.55	-1.32
ILMN_1365363	RGD1303003	-1.12	-1.03	1.06	-1.18	-1.65	-1.54	-1.32
ILMN_1374808	Mrpl4_predicted	-1.03	-1.14	-1.26	-1.35	-1.38	-1.54	-1.32
ILMN_1374452	Hnrpul1_predicted	-1.11	-1.32	-1.65	-1.42	-1.35	-1.46	-1.32
ILMN_1358372	Myh10	-1.01	-1.06	-1.04	1.13	-1.24	-1.42	-1.32
ILMN_1376327	Lnk	-1.11	1.32	-1.03	1.31	-1.16	-1.41	-1.32
ILMN_1368424	RGD1310868_predicted	1.01	-1.24	-1.41	-1.31	-1.46	-1.4	-1.32
ILMN_1373871	C20orf165	1.13	-1.15	1.07	1.06	-1.49	-1.39	-1.32
ILMN_1349751	Tysnd1_predicted	-1.07	-1.32	-1.01	-1.16	-1.15	-1.39	-1.32
ILMN_1376319	Fus	1.07	-1.06	-1.63	-1.49	-1.4	-1.36	-1.32

ILMN_1355830	Cbx8	-1.21	-1.24	-1.34	-1.61	-1.58	-1.35	-1.32
ILMN_1376358	Gtf2i	-1.06	-1.1	-1.11	-1.2	-1.51	-1.33	-1.32
ILMN_1369497	Pitrm1_predicted	-1.02	-1.35	-1.57	-1.42	-1.21	-1.26	-1.32
ILMN_1361060	Prr3	-1.16	-1.08	1.23	1.06	-1.17	-1.26	-1.32
ILMN_1362728	Psmb8	1.04	1	1.12	-1.01	-1.16	-1.23	-1.32
ILMN_1360936	Morc3_predicted	-1.05	-1.12	-1.16	-1.11	-1.38	-1.19	-1.32
ILMN_1367896	LOC685491	-1.14	-1.46	-1.14	-1.38	-1.33	-1.16	-1.32
ILMN_1373959	Map3k3_predicted	-1.04	-1.33	-1.35	-1.2	-1.32	-1.16	-1.32
ILMN_1370137	Trex1	-1.06	-1.24	-1.07	-1.1	-1.19	-1.14	-1.32
ILMN_1370301	Polr2f	-1.09	-1.17	1.01	1.01	-1.16	-1.09	-1.32
ILMN_1360824	Dctn5	-1.22	-1.42	1.05	-1.6	-1.17	-1.07	-1.32
ILMN_1352517	MGC109149	-1.23	-1.28	-1.41	-1.76	-1.12	-1.02	-1.32
ILMN_1350103	RGD1304593_predicted	-1.15	-1.22	-1.65	-1.24	1.26	1.23	-1.32
ILMN_1361326	Dhrs4	1	-1.31	1.3	-1.51	-1.53	-1.73	-1.31
ILMN_1361728	Mns1	-1.06	-1.24	-1.31	-1.41	-1.54	-1.65	-1.31
ILMN_1366522	Elmo3	-1.42	-1.13	-1.09	-1.47	-1.38	-1.59	-1.31
ILMN_1352913	Rbm35b_predicted	-1.16	-1.32	-1.17	-1.21	-1.48	-1.55	-1.31
ILMN_1369876	Tprkb	-1.11	-1.01	-1.06	-1.13	-1.37	-1.53	-1.31
ILMN_1365295	Fancl_predicted	-1.21	-1.25	-1.22	-1.57	-1.64	-1.49	-1.31
ILMN_1366111	Ptov1	-1.23	-1.24	-1.86	-1.47	-1.62	-1.48	-1.31
ILMN_1363364	Rab11b	-1.18	-1.71	-1.56	-1.29	-1.45	-1.48	-1.31
ILMN_1358225	RGD1306284	-1.01	-1.09	-1.54	-2	-1.3	-1.46	-1.31
ILMN_1350608	RGD1309207	-1.02	-1.37	-1.41	-1.68	-1.66	-1.44	-1.31
ILMN_1373101	Rnf34	-1.03	-1	-1.03	-1.06	-1.27	-1.43	-1.31
ILMN_1368874	Ttc23	-1.07	-1.41	-1.43	-1.83	-1.28	-1.4	-1.31
ILMN_1649947	Sephs1	-1.25	-1.71	-1.39	-1.54	-1.65	-1.39	-1.31
ILMN_1370150	Fbxw9	-1.06	-1.21	-1.12	-1.04	-1.29	-1.38	-1.31
ILMN_1359395	Apoa5	-1.12	-1.39	-1.31	-1.69	-1.2	-1.38	-1.31
ILMN_1368718	Chac1_predicted	-1.08	-1.15	1	-1.2	-1.13	-1.36	-1.31
ILMN_1353713	Ipo9_predicted	-1.05	-1.37	-1.19	-1.1	-1.49	-1.34	-1.31
ILMN_1358109	Tomm22	-1.08	-1.33	1.13	-1.17	-1.42	-1.3	-1.31
ILMN_1364837	Fbxl3	-1.03	-1.26	-1.33	-1.68	-1.34	-1.29	-1.31
ILMN_1376635	Cyp1b1	1.06	-1.11	-1.46	-1.31	-1.38	-1.26	-1.31
ILMN_1375706	Rxrb	1.12	1.26	1.12	-1.12	-1.47	-1.2	-1.31
ILMN_1364536	Dlat	1.16	1	1.03	1.1	-1.13	-1.14	-1.31
ILMN_1354003	Nmnat1	-1.09	-1.03	1.14	-1.14	-1.2	-1.13	-1.31
ILMN_1364160	Abl1_mapped	-1.07	1.17	-1.01	1.09	-1.12	-1.13	-1.31
ILMN_1360523	RGD1308066_predicted	-1.14	-1.04	-1.15	1.22	-1.03	-1.12	-1.31

ILMN_1351440	Rpn2	1.08	-1.44	1	-1.29	-1	-1.12	-1.31
ILMN_1376282	Mad2l2	-1.02	-1.29	-1.02	-1.13	-1.51	-1.11	-1.31
ILMN_1369542	Gcn5l2_predicted	1.01	1.39	-1.1	1.2	-1.26	-1.1	-1.31
ILMN_1355799	RGD1306107_predicted	-1.26	-1.13	-1.25	-1.01	-1.19	-1.1	-1.31
ILMN_1355269	Lztfl1	1	-1.45	-1.57	-1.82	-1.4	-1.02	-1.31
ILMN_1369110	Snx1	-1.21	-1.26	-1.03	-1.05	-1.05	1	-1.31
ILMN_1363922	Dgcr8_predicted	-1.13	-1.17	1.07	-1.04	1.01	1.07	-1.31
ILMN_1351199	Syvn1	-1.33	-1.31	-1.24	-1.28	1.25	1.28	-1.31
ILMN_1350544	Gusb	-1.02	-1.61	-1.67	-1.51	-2.24	-1.78	-1.3
ILMN_1374314	Serping1	-1.26	-1.37	-1.27	-1.87	-1.5	-1.71	-1.3
ILMN_1357075	C12orf10	-1.12	-1.02	1.04	-1.04	-1.6	-1.67	-1.3
ILMN_1363056	Camta2_predicted	-1.22	1.03	-1.11	-1.03	-1.74	-1.66	-1.3
ILMN_1358139	Pcbd1	-1.15	-1.77	-2.02	-2.09	-1.46	-1.65	-1.3
ILMN_1357109	F2	-1.31	-1.09	1.12	-1.32	-1.35	-1.64	-1.3
ILMN_1374140	Ptms	1.22	-1.49	-1.76	-1.52	-1.54	-1.59	-1.3
ILMN_1353797	Bag1_predicted	-1.11	-1.15	-1.33	-1.26	-1.52	-1.52	-1.3
ILMN_1362818	LOC361727	-1.04	-1.41	-1.32	-1.38	-1.47	-1.51	-1.3
ILMN_1364399	B3gnt6_predicted	1.01	-1.07	1.01	1.19	-1.62	-1.5	-1.3
ILMN_1369415	Podxl	1.3	1.1	1.29	1.41	-1.99	-1.45	-1.3
ILMN_1369051	Nme1	1.16	-1.38	-1.58	-1.23	-1.35	-1.44	-1.3
ILMN_1351252	RGD1562760_predicted	1	-1.23	1.05	-1.08	-1.49	-1.43	-1.3
ILMN_1376732	Scrn2	-1.16	-1.12	-1.15	-1.31	-1.32	-1.43	-1.3
ILMN_1369871	Znf324_predicted	-1.07	-1.1	-1.17	-1.15	-1.25	-1.43	-1.3
ILMN_1352703	Tmem63a_predicted	-1.1	-1.11	-1.19	-1.28	-1.34	-1.38	-1.3
ILMN_1370607	Nubp1	-1.21	-1.3	-1.4	-1.49	-1.37	-1.37	-1.3
ILMN_1353631	Cox6a1	1.03	-1.37	-1.75	-1.36	-1.37	-1.37	-1.3
ILMN_1356675	Pank1_predicted	-1.14	-1.39	-1.6	-1.5	-1.31	-1.36	-1.3
ILMN_1352591	Prpf38a_predicted	-1.43	-1.47	-1.41	-1.41	-1.08	-1.36	-1.3
ILMN_1360896	Tcfe2a	-1.22	1.01	-1.06	1.08	-1.25	-1.33	-1.3
ILMN_1370903	LOC683694	-1.16	-1.37	-1.42	-1.55	-1.42	-1.32	-1.3
ILMN_1355048	Zfp668_predicted	-1.25	-1.29	-1.08	-1.05	-1.29	-1.32	-1.3
ILMN_1356083	Slc29a2	-1.09	-1.12	1	1.35	-1.29	-1.32	-1.3
ILMN_1357975	Dnajb5_predicted	-1.21	-1.06	-1.17	-1.09	-1.31	-1.31	-1.3
ILMN_1361395	Frap1	-1.06	-1.2	-1.54	-1.22	-1.32	-1.3	-1.3
ILMN_1367182	Wdr75	-1	-1.01	1.16	1.07	-1.2	-1.29	-1.3
ILMN_1362862	RGD1307832_predicted	-1.12	-1.07	-1.07	-1.07	-1.25	-1.28	-1.3
ILMN_1366935	Spnb3	-1.01	1.05	-1.28	1.01	-1.55	-1.27	-1.3
ILMN_1650975	Wbscr21	1.03	-1.3	1.03	-1.11	-1.21	-1.26	-1.3

ILMN_1361479	RGD1309888	-1.01	-1.05	1.02	-1.05	-1.12	-1.2	-1.3
ILMN_1370224	Rcbtb1	-1.09	-1.13	-1.34	-1.22	-1.1	-1.2	-1.3
ILMN_1357874	RGD1564042_predicted	1.1	-1.42	-1.48	-1.57	-1.53	-1.18	-1.3
ILMN_1650840	Ccnl2_predicted	-1.24	1.06	1.07	-1.07	-1.42	-1.17	-1.3
ILMN_1369837	Raf1	-1.32	-1.28	-1.27	-1.29	-1.23	-1.15	-1.3
ILMN_1357015	Slc6a9	1.55	1.34	1.38	1.03	1.09	1	-1.3
ILMN_1360328	Pgrmc2	-1.06	-1.19	1.14	-1.2	-1.14	1.03	-1.3
ILMN_1376385	Nap1l4	1	-1.14	1.02	-1.19	-1.12	1.07	-1.3
ILMN_1369596	Bcl2l13_predicted	1	1.13	1.09	1.09	1.02	1.09	-1.3
ILMN_1364804	RGD1308279_predicted	-1.08	-1.17	-1.37	-1.15	1	1.1	-1.3
ILMN_1649787	RGD1561590_predicted	-1.17	-1.24	-1.34	-1.26	1.12	1.26	-1.3
ILMN_1368228	LOC365842	-1.19	-1.39	-1.24	-1.12	-1.64	-1.64	-1.29
ILMN_1374634	Zc3h7b_predicted	-1.02	-1.26	-1.39	-1.24	-1.42	-1.57	-1.29
ILMN_1365207	Vars2l	-1.12	-1.19	-1.28	-1.07	-1.56	-1.55	-1.29
ILMN_1370898	RGD1308872_predicted	1.14	-1.53	-1.62	-1.27	-1.25	-1.53	-1.29
ILMN_2040836	Sumo2	-1.21	-1.45	-1.37	-1.4	-1.49	-1.47	-1.29
ILMN_1363427	LOC684624	-1	-1.4	-2.33	-1.67	-1.42	-1.44	-1.29
ILMN_1350394	Sel1h	-1.28	-1.55	-1.59	-1.56	-1.28	-1.41	-1.29
ILMN_1366479	Ezh1_predicted	-1.15	-1.07	-1.15	-1.15	-1.27	-1.41	-1.29
ILMN_1373473	Acat1	-1.02	-1.31	-1.14	-1.53	-1.28	-1.4	-1.29
ILMN_1371753	F5_mapped	-1.03	1.05	-1.37	-1.23	-1.28	-1.4	-1.29
ILMN_1356901	Cog1_predicted	-1.07	-1.29	-1.08	-1.29	-1.64	-1.39	-1.29
ILMN_1373233	Mto1_predicted	-1.15	-1.27	-1.12	-1.22	-1.59	-1.37	-1.29
ILMN_1351644	LOC501311	-1.11	-1.25	1.3	-1.28	-1.49	-1.29	-1.29
ILMN_1369087	RGD1304567	-1.08	-1.09	-1.23	-1.01	-1.19	-1.29	-1.29
ILMN_1365821	RGD1566093_predicted	-1.05	-1.09	-1.39	-1.41	-1.28	-1.26	-1.29
ILMN_1361125	Ash2l_predicted	-1.08	-1.23	1.01	-1.23	-1.42	-1.24	-1.29
ILMN_1362149	Mrpl46	-1.08	-1.07	-1.08	1.02	-1.36	-1.24	-1.29
ILMN_1372842	Cars_predicted	-1.15	-1.3	-1.37	-1.05	-1.02	-1.24	-1.29
ILMN_1357988	RGD1560498_predicted	-1.11	-1.11	-1.02	-1.04	-1.18	-1.23	-1.29
ILMN_1355680	Polr3d	1.02	-1.14	1.09	-1.04	-1.2	-1.22	-1.29
ILMN_1351388	Smc6l1_predicted	1.06	-1.14	-1.12	1.12	-1.3	-1.19	-1.29
ILMN_1365030	Grinl1a	-1.12	-1.3	1.22	-1.17	-1.36	-1.15	-1.29
ILMN_1370640	LOC498909	-1.03	-1.39	-1.1	-1.35	-1.3	-1.14	-1.29
ILMN_1368866	Exosc6_predicted	-1.05	-1.16	-1.01	-1.04	-1.1	-1.13	-1.29
ILMN_1353072	Ing3	-1.07	-1.36	1.02	-1.23	-1.42	-1.12	-1.29
ILMN_1362703	Stip1	-1.06	-1.23	1.07	1.2	-1.1	-1.1	-1.29
ILMN_1376824	Dnmt3a	-1.11	1.22	-1.09	1.41	1.07	-1.07	-1.29

ILMN_1373095	Wbscr1	-1.13	-1.18	-1.19	-1.24	-1.09	-1.06	-1.29
ILMN_1373805	Pols_predicted	-1.06	1.33	1.22	1.43	-1.09	-1.04	-1.29
ILMN_1354004	Stag1_predicted	1.23	-1.21	-1.28	-1.09	-1.31	-1.01	-1.29
ILMN_1362634	RGD1303232	-1.28	-1.34	1.06	-1.93	-1.82	-1.8	-1.28
ILMN_1354307	Sdf4	-1.17	-1.83	-1.48	-1.63	-1.74	-1.78	-1.28
ILMN_1374963	Acp2	-1.13	-1.12	-1.12	-1.28	-1.42	-1.62	-1.28
ILMN_1361029	RGD1308959	-1.07	-1.38	-1.5	-1.58	-1.62	-1.53	-1.28
ILMN_1352857	Mum1_predicted	1.04	-1.09	-1.07	-1.04	-1.55	-1.53	-1.28
ILMN_1364635	Rhbdl7_predicted	1.04	-1.25	1.1	-1.19	-1.78	-1.49	-1.28
ILMN_1372701	Nmb_predicted	1	-1.2	-1.19	-1.2	-1.43	-1.48	-1.28
ILMN_1365082	Ndufv1	-1.08	-1.23	-1.23	-1.39	-1.34	-1.45	-1.28
ILMN_1367341	Tbc1d7_predicted	-1.07	-1.36	-1.14	-1.27	-1.33	-1.43	-1.28
ILMN_1650896	Ostf1	-1.17	-2.44	-2.05	-2.59	-1.49	-1.36	-1.28
ILMN_1349407	Usp5_predicted	-1.1	-1.38	-1.45	-1.18	-1.4	-1.36	-1.28
ILMN_1376351	Tada2l	-1.04	1	1.04	1.11	-1.17	-1.36	-1.28
ILMN_1650976	Dsm-1	-1.1	-1.03	-1.01	1.11	-1.19	-1.34	-1.28
ILMN_1372734	Ppia	-1.01	-1.44	-1.39	-1.37	-1.4	-1.33	-1.28
ILMN_1376534	Cdadc1	-1.24	-1.68	-1.45	-1.82	-1.21	-1.32	-1.28
ILMN_2038910	Trfr2_predicted	-1.29	-1	1.14	-1.11	-1.09	-1.31	-1.28
ILMN_1362476	Mapk7	-1.27	-1.02	1.08	-1.1	-1.14	-1.28	-1.28
ILMN_1370992	RGD1307161	-1.05	-1.05	1.05	-1.25	-1.16	-1.25	-1.28
ILMN_1366282	Gtf2b	-1.16	-1.49	-1.59	-1.76	-1.06	-1.24	-1.28
ILMN_1354877	LOC362587	-1.13	-1.27	-1.32	-1.29	-1.35	-1.23	-1.28
ILMN_1362810	Ulk1_mapped	-1.23	-1.39	-1.66	-1.92	-1.29	-1.22	-1.28
ILMN_1358978	RGD1306126	-1.16	-1.23	-1.16	-1.06	-1.16	-1.22	-1.28
ILMN_1361432	LOC288654	-1.08	-1.07	-1.12	-1.1	-1.46	-1.19	-1.28
ILMN_1353663	Apobec3	-1.12	-1.09	1.07	-1.06	-1.27	-1.18	-1.28
ILMN_1375687	RGD1559843_predicted	-1.13	-1.24	-1.3	-1.06	-1.29	-1.16	-1.28
ILMN_1366034	Dcbld1_predicted	-1.06	-1.12	-1.06	-1.09	-1.09	-1.16	-1.28
ILMN_1357638	Sucla2_predicted	-1.01	-1.33	-1.11	-1.39	-1.16	-1.12	-1.28
ILMN_1349509	Clasp2	1.23	1.24	-1.02	1.24	-1	-1.1	-1.28
ILMN_1371451	Tpcn1	-1.1	1.06	-1.18	1.2	-1.07	-1.08	-1.28
ILMN_1376723	Rnf10	-1.08	-1.23	-1.37	-1.23	-1.14	-1.07	-1.28
ILMN_1354675	Chrac1_predicted	-1.08	-1.21	-1.38	-1.23	-1.1	-1.05	-1.28
ILMN_1352844	Bivm_predicted	1.06	-1.09	1.06	-1.14	-1.27	-1.04	-1.28
ILMN_1376805	Serpinf2	-1.27	1.21	-1.13	-1.03	1.14	-1.03	-1.28
ILMN_1368786	LOC500248	1.14	-1.04	-1.33	-1.19	1.11	-1.02	-1.28
ILMN_1348971	RGD1310683_predicted	1.1	-1.04	1.11	1.02	1.07	1.01	-1.28

ILMN_1357093	Tpm4	1.24	-1.22	1.24	1.09	-1.03	1.03	-1.28
ILMN_1370599	RGD1307526	1.11	-1.12	-1.05	-1.06	1.15	1.13	-1.28
ILMN_1359196	RGD1307526	1.11	-1.12	-1.05	-1.06	1.15	1.13	-1.28
ILMN_1362410	Rnase4	-1.07	-1.12	-1.13	-2.49	1.13	1.21	-1.28
ILMN_1530353		1.04	-1.19	-1.25	-1.15	-1.63	-1.65	-1.27
ILMN_1356825	Hrsp12	-1.06	-1.47	-1.52	-1.87	-1.44	-1.71	-1.27
ILMN_1358349	Mrpl49	-1.11	-1.52	-1.34	-1.37	-1.67	-1.68	-1.27
ILMN_1355168	RGD1562228_predicted	-1.06	-1.35	-1.44	1	-1.74	-1.65	-1.27
ILMN_1367459	Erp29	-1.03	-1.23	-1.22	-1.13	-1.54	-1.62	-1.27
ILMN_1367386	Bles03	-1.22	-1.41	-1.49	-1.44	-1.64	-1.61	-1.27
ILMN_1350701	Nadsyn1	-1.27	-1.49	-1.68	-1.52	-1.37	-1.57	-1.27
ILMN_1359133	Zmym3	-1.16	-1.35	-1.05	-1.37	-1.56	-1.48	-1.27
ILMN_1359427	Ep400	-1.16	-1.14	-1.52	-1.17	-1.52	-1.48	-1.27
ILMN_1351956	Pcnxl3	-1.18	1.12	-1.31	1.05	-1.47	-1.48	-1.27
ILMN_1365165	Brp44l	-1.26	-1.77	-1.77	-1.93	-1.27	-1.47	-1.27
ILMN_1363598	RGD1306844	-1.12	-1.39	-1.44	-1.55	-1.65	-1.45	-1.27
ILMN_1353074	Snx25	-1.16	-1.04	-1.06	1.05	-1.41	-1.45	-1.27
ILMN_1356626	Nup160_predicted	1.14	-1.16	-1.25	1.12	-1.39	-1.41	-1.27
ILMN_1369137	Atp5i	1.08	-1.24	-3.33	-1.31	-1.43	-1.38	-1.27
ILMN_1352592	Smagp	-1.09	-1.49	-1.3	-1.42	-1.35	-1.36	-1.27
ILMN_1372541	Nphp1_predicted	-1.24	-1.06	1.24	-1.22	-1.21	-1.36	-1.27
ILMN_1362331	LOC687237	-1.04	-1.77	-3.15	-2.24	-1.24	-1.34	-1.27
ILMN_1366239	Setdb1_predicted	-1.04	1.24	-1.01	1.01	-1.3	-1.32	-1.27
ILMN_1355855	LOC298643	-1.25	-1.17	-1.2	-1.13	1.02	-1.32	-1.27
ILMN_1372465	LOC367170	-1.07	-1.22	1	-1.08	-1.19	-1.29	-1.27
ILMN_1361612	LOC362774	-1.05	-1.17	-1.13	-1.32	-1.28	-1.28	-1.27
ILMN_1372924	Cpn1	1	-1.82	-2	-2.9	-1.4	-1.27	-1.27
ILMN_1376881	Dap3	-1.06	-1.05	1.05	-1.2	-1.26	-1.27	-1.27
ILMN_1350057	Smap1l	-1.22	-1.17	1.04	-1.03	-1.21	-1.27	-1.27
ILMN_1367212	Cbx7	1.04	-1.11	-1.08	-1.01	-1.3	-1.26	-1.27
ILMN_1351765	MGC72581	-1.25	-1.17	1.11	-1.14	-1.14	-1.24	-1.27
ILMN_1372508	RGD1308082	-1.09	-1.88	-1.18	-1.64	-1.18	-1.22	-1.27
ILMN_1373654	Mre11a	1.07	-1.04	-1.28	-1.17	-1.43	-1.21	-1.27
ILMN_1359314	Senp1_predicted	1.1	-1.19	-1.2	-1.19	-1.23	-1.2	-1.27
ILMN_1349327	LOC368070	-1.34	-1.43	-1.21	-1.3	-1.18	-1.2	-1.27
ILMN_1355431	Epc1_predicted	-1.27	1.04	-1.15	-1.31	-1.16	-1.2	-1.27
ILMN_1363067	Slc9a1	-1.13	-1.26	-1.18	-1.08	-1.1	-1.2	-1.27
ILMN_1373327	LOC300963	-1.02	-1.19	-1.04	-1.1	-1.21	-1.19	-1.27

ILMN_1355977	Glo1	1.02	1.06	1.03	-1.01	-1.2	-1.15	-1.27
ILMN_1359509	LOC498749	-1.04	-1.32	-1.21	-1.34	-1.4	-1.14	-1.27
ILMN_1374439	RGD1308076	-1.13	-1.07	-1.25	-1.18	-1.24	-1.14	-1.27
ILMN_1376987	Actn4	-1.03	-1.1	-1.09	1.15	1.11	-1.14	-1.27
ILMN_1360977	Adnp	1.08	1.04	1.02	-1.11	-1.23	-1.11	-1.27
ILMN_1361506	Acp1	-1.09	-1.14	1.07	1.03	-1.08	-1.11	-1.27
ILMN_1370316	Atp1b1	-1.82	1.48	1.3	1.57	-1.15	-1.09	-1.27
ILMN_1362729	MGC112727	1.08	1.28	-1.03	-1.03	-1.05	-1.08	-1.27
ILMN_1352155	Oat	-1.36	-1.06	1.04	-1.58	-1.01	-1.06	-1.27
ILMN_1352296	LOC500840	-1.04	-1.29	-1.89	-1.62	1.07	-1.05	-1.27
ILMN_1363777	Scap2	-1.2	-1.56	-1.41	-1.8	-1.16	-1.03	-1.27
ILMN_1376637	Lyar	-1.01	1.27	1.35	1.46	-1.14	-1.02	-1.27
ILMN_1361642	RGD1307365	-1.04	-1.15	-1.24	-1.2	-1.22	1.03	-1.27
ILMN_1373417	Sp1	1.02	-1.1	-1.11	-1.25	-1.07	1.05	-1.27
ILMN_1359685	Wipi2	-1.01	-1.19	-1.24	-1.24	-1.07	1.05	-1.27
ILMN_1360362	Vgll4	-1.21	1	-1.18	-1.22	1.09	1.11	-1.27
ILMN_1650864	Taf9_predicted	-1.08	-1.26	-1.05	-1.24	1.19	1.13	-1.27
ILMN_1357443	Atp2c1	-1.13	-2.11	-2.51	-2.23	-1.94	-2.21	-1.26
ILMN_1367859	Gcdh_predicted	-1.29	-1.46	-1.65	-1.7	-1.89	-1.94	-1.26
ILMN_1355402	Abr_predicted	-1.06	1.19	-1.02	1.28	-1.41	-1.59	-1.26
ILMN_1369386	Blvra	-1.27	-1.71	-1.54	-1.77	-1.55	-1.54	-1.26
ILMN_1361484	Nnt_mapped	-1.1	1.12	-1.11	-1.4	-1.33	-1.54	-1.26
ILMN_1350945	Nup37_predicted	1.04	-1.04	1.08	-1.12	-1.49	-1.53	-1.26
ILMN_1650362	Atp5o	-1.13	-1.26	-1.49	-1.47	-1.43	-1.52	-1.26
ILMN_1349297	Brf1_predicted	-1.22	-1.22	-1.17	-1.13	-1.41	-1.51	-1.26
ILMN_1355654	Agrn	-1.19	1.08	-1.14	1.3	-1.43	-1.48	-1.26
ILMN_1358945	Ankrd47_predicted	-1.05	1.11	1.22	-1.05	-1.28	-1.44	-1.26
ILMN_1363899	Pitpnm1	-1.24	-1.05	-1.23	1.01	-1.34	-1.42	-1.26
ILMN_1650035	Tbc1d10b_predicted	1.03	1.1	-1.07	1.09	-1.13	-1.4	-1.26
ILMN_1364389	Rab5b_predicted	-1.18	-1.41	-1.32	-1.48	-1.25	-1.39	-1.26
ILMN_1349971	Dsp	-1.2	-1.3	-1.21	-1.28	-1.68	-1.37	-1.26
ILMN_1371691	Bckdha	-1.19	-1.52	-1.25	-1.46	-1.31	-1.37	-1.26
ILMN_1361251	LOC497811	-1.06	-1.62	-1.72	-1.88	-1.57	-1.35	-1.26
ILMN_1367028	Psmb5	-1.01	-1.16	-1.29	-1.28	-1.44	-1.35	-1.26
ILMN_1369483	Habp2	-1.09	1.2	1.17	-1.17	-1.17	-1.35	-1.26
ILMN_1373237	Dars2	-1.07	-1.2	-1.45	-1.54	-1.49	-1.32	-1.26
ILMN_1370154	Alcam	-1.12	-1.6	-1.72	-2.07	-1.47	-1.29	-1.26
ILMN_1354399	Cox7b	-1.02	-1.3	-1.79	-1.17	-1.21	-1.27	-1.26

ILMN_1359465	Pak4_predicted	-1.05	-1.28	-1.24	-1.09	-1.08	-1.27	-1.26
ILMN_1366414	Akap1	-1.08	1.05	1.25	-1.11	-1.25	-1.26	-1.26
ILMN_1363137	Coil	-1.08	-1.04	-1.17	1	-1.22	-1.25	-1.26
ILMN_1376777	Mpst	-1.09	1.02	1.09	-1.29	-1.17	-1.25	-1.26
ILMN_1354434	Ppfia1_predicted	-1.12	-1.1	1.08	-1.03	-1.13	-1.24	-1.26
ILMN_1372058	Cnot8	-1.09	-1.37	-1.3	-1.53	-1.25	-1.23	-1.26
ILMN_1374289	Leo1	-1.14	-1.16	-1.07	-1.22	-1.21	-1.23	-1.26
ILMN_1372140	Tsga14	-1.05	-1.48	-1.05	-1.39	-1.17	-1.23	-1.26
ILMN_1353153	RGD1306410	-1.23	-1.22	-1.11	-1.41	-1.18	-1.21	-1.26
ILMN_1366267	Rbmxrt_predicted	-1.16	-1.22	-1.04	-1.15	-1.16	-1.18	-1.26
ILMN_1371704	RGD1309219	1.12	-1.07	-1.02	-1.12	-1.12	-1.18	-1.26
ILMN_1364745	Slc9a3r1	-1.49	-1.41	-1.75	-1.71	-1.06	-1.18	-1.26
ILMN_1650485	RGD1563106_predicted	-1.2	-1.13	-1.22	-1.34	-1.27	-1.17	-1.26
ILMN_1369486	Ppcdc_predicted	-1.04	-1.15	-1.21	-1.26	-1.21	-1.15	-1.26
ILMN_1650615	RGD1307981_predicted	-1.1	1.14	-1.06	1.2	-1.17	-1.12	-1.26
ILMN_1362976	Cad_mapped	-1.02	-1.05	-1.18	1	-1.33	-1.11	-1.26
ILMN_1374222	Impdh2	-1.02	1.15	1.43	1.11	-1.29	-1.1	-1.26
ILMN_1362980	Bms1l	-1.05	1.06	1.11	1.21	-1.29	-1.1	-1.26
ILMN_1352344	MGC94954	-1.15	1.17	1.15	1.11	-1.2	-1.07	-1.26
ILMN_1364181	RGD621352	-1.14	-1.14	-1.07	-1.14	-1.08	-1.06	-1.26
ILMN_1372226	Usp42_predicted	-1.09	-1.08	-1.09	1.01	1.02	-1.05	-1.26
ILMN_1369875	LOC498027	-1.24	1.27	1.19	1.25	1.12	-1.04	-1.26
ILMN_1651070	Nfyb	1.01	-1.05	1.26	1.19	-1.11	-1.03	-1.26
ILMN_1366699	Ankrd10	-1.24	-1.15	-1.33	-1.14	-1.03	-1.02	-1.26
ILMN_1357214	RGD1560880_predicted	-1.09	-1.1	1.03	-1.14	-1.23	1.01	-1.26
ILMN_1360120	Ankrd11_predicted	1.03	1.06	1.16	1.03	-1	1.13	-1.26
ILMN_1368936	Prpsap1	1.03	1.07	-1.03	-1.16	1.04	1.18	-1.26
ILMN_1354296	Cyp2r1_predicted	-1.02	-1.33	-1.18	-1.36	-1.38	-1.57	-1.25
ILMN_1350655	Cyp2t1	1.01	-1.24	-1.17	-1.45	-1.48	-1.53	-1.25
ILMN_1349280	Gcs1	1.03	-1.1	-1.16	-1.06	-1.47	-1.46	-1.25
ILMN_1357393	Rps29	1.15	-1.16	-2.38	-1.3	-1.32	-1.4	-1.25
ILMN_1351435	Anapc4	-1.12	-1.08	-1.13	-1.31	-1.44	-1.36	-1.25
ILMN_1376555	Arpc1a	-1.22	-1.55	-1.28	-1.49	-1.22	-1.35	-1.25
ILMN_1367593	LOC306324	1.05	1.04	1.13	-1.02	-1.27	-1.33	-1.25
ILMN_1364057	RGD1566420_predicted	1.04	-1.19	-1.18	-1.14	-1.52	-1.32	-1.25
ILMN_1349135	RGD1565465_predicted	-1.1	1.02	1.07	-1.16	-1.38	-1.32	-1.25
ILMN_1372029	LOC497766	1.04	-1.22	-1.33	-1.35	-1.28	-1.32	-1.25
ILMN_1370276	Mak10	-1.06	-1.24	-1.37	-1.27	-1.29	-1.31	-1.25

ILMN_1363443	RGD1564087_predicted	-1.06	-1.62	-1.09	-1.53	-1.12	-1.31	-1.25
ILMN_1348979	MGC93707	-1.33	-1.36	-1.56	-1.39	-1.28	-1.3	-1.25
ILMN_1353417	LOC499785	-1.12	-1.13	-1.28	-1.08	-1.25	-1.29	-1.25
ILMN_1364722	Rpo1-4	1	1.1	-1.12	1.11	-1.2	-1.29	-1.25
ILMN_1350489	Hyal2	-1.28	-1	-1.14	-1.09	-1.31	-1.28	-1.25
ILMN_1373041	Vamp3	-1.28	-1.34	1	-1.22	-1.29	-1.28	-1.25
ILMN_1360881	MGC112899	1.17	-1.2	-2.46	-1.4	-1.35	-1.27	-1.25
ILMN_1366643	Aip1	1.13	-1.1	-1.53	-1.17	-1.31	-1.27	-1.25
ILMN_1359297	Mbd3_predicted	-1.06	-1.46	-1.22	-1.32	-1.48	-1.26	-1.25
ILMN_1350743	Prmt5_predicted	1.01	1.19	1.02	1.19	-1.34	-1.26	-1.25
ILMN_1375089	Mtch2_predicted	-1.1	-1.66	-1.28	-1.7	-1.09	-1.25	-1.25
ILMN_1376893	Foxa2	1.03	-1.15	-1.38	-1.26	-1.28	-1.24	-1.25
ILMN_1366542	LOC685745	-1.1	-1.27	1.45	-1.31	-1.3	-1.22	-1.25
ILMN_1360742	Trrap_predicted	-1.24	-1.3	-1.23	-1.19	-1.37	-1.21	-1.25
ILMN_1367144	Fusip1	-1.01	-1.17	-1.08	-1.07	-1.24	-1.2	-1.25
ILMN_1369085	Cugbp1	1.08	-1.05	1.1	1.08	-1.15	-1.2	-1.25
ILMN_1366409	Txndc12	1.1	-1.09	1.22	-1.06	-1.23	-1.19	-1.25
ILMN_1370658	Ing5_predicted	1.09	-1.1	-1.39	-1.05	-1.22	-1.18	-1.25
ILMN_1361551	Ehmt1_predicted	-1.01	1.22	-1.28	1.07	-1.19	-1.17	-1.25
ILMN_1356425	Ptbp1	1.11	-1.01	1.05	1.03	-1.21	-1.16	-1.25
ILMN_1365389	RGD1305481	-1.25	-1.36	-1.26	-1.5	-1.15	-1.08	-1.25
ILMN_1374158	RGD1311135_predicted	-1.31	-1.11	-1.18	-1.14	-1.42	-1.05	-1.25
ILMN_1370049	Zfp180	1.01	1.01	-1.03	1.09	-1.16	-1.03	-1.25
ILMN_1349043	MGC94464	1.04	-1.46	-1.49	-1.52	1.13	-1.02	-1.25
ILMN_1366064	G10	-1.13	-1.24	-1.27	-1.42	-1.03	-1.01	-1.25
ILMN_1375142	Atp2a2	-1.28	-1.54	-1.26	-1.66	1.13	-1	-1.25
ILMN_1371482	RGD1562294_predicted	-1.02	1.18	1.08	1.08	-1.12	1.02	-1.25
ILMN_1364029	Ugcgl1	-1.15	-1.18	-1.3	-1.25	-1.09	1.04	-1.25
ILMN_1374474	Tjap1_predicted	-1.17	1.13	-1.2	1.06	1.07	1.11	-1.25
ILMN_1349946	Eif1b_predicted	-1.25	-1.22	-1.19	-1.25	1.22	1.11	-1.25
ILMN_1366726	RGD1303127	1.01	1.03	1.13	-1.02	-1.08	1.13	-1.25
ILMN_1350423	RGD1305797_predicted	1.25	1.03	1.07	-1.15	-1.07	1.18	-1.25
ILMN_1364250	Tuba4	1	-1.61	-1.16	1.3	-1.48	-1.92	-1.24
ILMN_1352808	Lamb1_predicted	-1.28	1.16	-1.32	-1.01	-1.5	-1.5	-1.24
ILMN_1354653	Crot	-1.24	-1.36	-1.33	-1.55	-1.48	-1.48	-1.24
ILMN_2039669	Eif2b5	-1.05	-1.07	-1.04	-1.07	-1.58	-1.44	-1.24
ILMN_1354857	Gba2	-1.1	-1.11	-1.37	-1.07	-1.43	-1.44	-1.24
ILMN_1358646	Fbp1	-1.06	-1.06	-1.22	-1.38	-1.29	-1.42	-1.24

ILMN_1361743	Pla2g1b	-1.21	3.44	2.09	1.83	-1.16	-1.4	-1.24
ILMN_1374769	RGD1304822_predicted	-1.09	-1.04	-1.15	-1.23	-1.49	-1.38	-1.24
ILMN_1349823	Slc35b4_predicted	-1.05	-1.1	-1.01	-1.37	-1.45	-1.38	-1.24
ILMN_1354050	LOC310946	-1.08	-1.27	-1.1	-1.15	-1.35	-1.38	-1.24
ILMN_1357630	RGD1306809_predicted	1.13	-1.39	-1.36	-1.75	-1.36	-1.36	-1.24
ILMN_1358247	Slc25a4	-1.04	-1.09	1.02	-1.17	-1.26	-1.35	-1.24
ILMN_2038940	Snx6_predicted	1.02	-1.3	1.21	-1.06	-1.39	-1.34	-1.24
ILMN_1361918	Ncstn	-1.2	-1.25	1.02	-1.24	-1.29	-1.33	-1.24
ILMN_1365577	Pqbp1	1.02	-1.48	-1.36	-1.39	-1.84	-1.3	-1.24
ILMN_1365923	RGD1310794	-1.03	-1.33	-1.17	-1.38	-1.46	-1.28	-1.24
ILMN_1362420	Ssfa2_predicted	-1.14	-1.09	-1.01	1.13	-1.28	-1.27	-1.24
ILMN_1361070	Amid_predicted	-1.23	-1.2	-1.09	-1.14	-1.19	-1.25	-1.24
ILMN_1351692	Dopey2_predicted	-1.17	-1.31	-1.31	-1.3	-1.37	-1.24	-1.24
ILMN_1354101	Dazap1_predicted	-1.07	-1.19	-1.29	1.05	-1.42	-1.23	-1.24
ILMN_1369798	Hnrpf	-1.14	-1.13	1.1	-1	-1.12	-1.23	-1.24
ILMN_1371665	RGD1560493_predicted	1.09	-1.02	-1.23	-1.05	-1.46	-1.2	-1.24
ILMN_1355094	RGD1309710_predicted	1.11	-1.06	-1.58	-1.25	-1.26	-1.2	-1.24
ILMN_1357044	Foxo1a	-1.08	-1.22	-1.27	-1.07	-1.23	-1.16	-1.24
ILMN_1357279	LOC287274	-1.13	-1.62	-2.15	-2.09	-1.2	-1.16	-1.24
ILMN_1363349	Hs2st1	-1.09	-1.18	-1.17	-1.08	-1.01	-1.15	-1.24
ILMN_1361799	RGD1561792_predicted	-1.1	-1.37	-1.68	-1.23	-1.2	-1.14	-1.24
ILMN_1350942	Muted_predicted	-1.05	-1.27	1	-1.14	-1.14	-1.13	-1.24
ILMN_1366977	Siae_predicted	-1.04	-1.44	-1.31	-1.45	-1.32	-1.12	-1.24
ILMN_1363689	Nhn1	-1.15	-1.11	-1.33	-1.2	-1.07	-1.12	-1.24
ILMN_1358940	Rab43	-1.12	-1.2	-1.33	-1.25	-1.14	-1.1	-1.24
ILMN_1374816	RGD1563028_predicted	1.08	1.1	1.01	-1.19	-1.1	-1.1	-1.24
ILMN_1358681	RGD1561319_predicted	-1.18	-1.11	-1.13	-1.12	-1.24	-1.09	-1.24
ILMN_1353785	LOC497941	-1.1	-1.11	-1.09	-1.05	-1.17	-1.09	-1.24
ILMN_1351949	Cabin1	-1.02	-1.06	1.08	1.08	-1.45	-1.07	-1.24
ILMN_1364888	LOC497673	-1.04	-1.17	-1.21	-1.4	-1.31	-1.06	-1.24
ILMN_1357553	Saps1_predicted	-1.02	1.04	-1.38	1.1	-1.09	-1.05	-1.24
ILMN_1350909	RGD1564036_predicted	1.1	-1.13	-1.04	1.07	-1.17	-1.04	-1.24
ILMN_1374155	Nfkb2	-1.28	1.08	-1.39	1.03	1.15	-1.04	-1.24
ILMN_1363761	Mcm3ap_predicted	-1.14	1.01	-1.34	-1.13	-1.09	-1.01	-1.24
ILMN_1650237	Ap1g1	-1.08	-1.23	-1.22	-1.25	-1.08	-1.01	-1.24
ILMN_1367532	LOC502635	1.21	1.09	1.32	1.42	-1.25	1.03	-1.24
ILMN_1360127	Insr	1.17	1.03	1.37	1	-1.01	1.03	-1.24
ILMN_1362042	LOC497768	-1.03	1	-1.04	-1.28	-1.03	1.05	-1.24

ILMN_1363135	Clic4	1.26	-1.16	-1.22	1.04	1.01	1.1	-1.24
ILMN_1366212	Errfi1	-1.58	1.02	1.06	-1.1	1.37	1.1	-1.24
ILMN_1360665	Pdcd6ip	-1.04	-1.14	-1.09	1.06	1.1	1.12	-1.24
ILMN_1356422	Timm22	1.01	-1.45	-1.42	-1.69	-1.88	-2.01	-1.23
ILMN_1355827	RGD1306959_predicted	-1.02	-1.57	-1.66	-1.59	-1.53	-1.61	-1.23
ILMN_1349703	Upf3b_predicted	-1.12	-1.27	-1.27	-1.25	-1.65	-1.54	-1.23
ILMN_1352204	LOC682869	-1.23	1.02	1.18	-1.37	-1.25	-1.52	-1.23
ILMN_1650314	RGD1306928_predicted	1.1	1.04	-1.12	1.08	-1.21	-1.49	-1.23
ILMN_1350372	Ccnd1	-1.01	-1.31	-1.2	-1.2	-1.58	-1.47	-1.23
ILMN_1358159	Wbscr16_predicted	-1.07	-1.14	-1.15	-1.32	-1.42	-1.41	-1.23
ILMN_1362585	Apeh	-1.14	-1.19	1.27	1.03	-1.27	-1.37	-1.23
ILMN_1373380	Mrpl19	-1.04	-1.16	-1.1	1.07	-1.46	-1.36	-1.23
ILMN_1356946	Nat2	-1.19	-1.37	-1.29	-1.5	-1.18	-1.36	-1.23
ILMN_1367061	RGD1561141_predicted	1.22	1.16	1.03	1.02	-1.11	-1.36	-1.23
ILMN_1370292	RGD1561141_predicted	1.22	1.16	1.03	1.02	-1.11	-1.36	-1.23
ILMN_1352839	LOC682893	-1.13	-1.19	1.07	-1.17	-1.26	-1.34	-1.23
ILMN_1365031	RGD1304696	-1.18	-1.15	-1.13	-1.37	-1.33	-1.33	-1.23
ILMN_1376660	Lig3	1.07	-1.13	-1.28	-1.09	-1.43	-1.32	-1.23
ILMN_1367882	Jtb	-1	-1.18	-1.1	-1.18	-1.16	-1.32	-1.23
ILMN_1359514	LOC687849	-1.11	-1.34	-1.22	-1.09	-1.64	-1.29	-1.23
ILMN_1367569	RGD1305633_predicted	1.05	1.08	1.24	1.35	-1.45	-1.29	-1.23
ILMN_1359719	Tcp1	-1.04	-1.09	1.12	1	-1.24	-1.29	-1.23
ILMN_1360294	LOC685878	1.01	-1.32	-1.03	-1.14	-1.43	-1.28	-1.23
ILMN_1371836	Stat5b	-1	1.26	1.36	1.01	-1.29	-1.28	-1.23
ILMN_1374047	Yeats4_predicted	1.01	1.03	1.07	-1.27	-1.26	-1.28	-1.23
ILMN_1349516	St3gal2	-1.02	-1.2	-1.37	-1.23	-1.47	-1.27	-1.23
ILMN_1649873	Rpusd3_predicted	1.14	-1.15	-1.03	-1.09	-1.12	-1.25	-1.23
ILMN_1350011	Rere	-1.13	1.12	-1.23	-1.22	-1.32	-1.24	-1.23
ILMN_1352855	Ywhae	-1.12	-1.33	-1.2	-1.26	-1.31	-1.23	-1.23
ILMN_1354205	Pebp1	-1.02	-1.39	-1.13	-1.26	-1.21	-1.22	-1.23
ILMN_1369917	RGD1311595	-1.02	-1.13	-1.53	-1.46	-1.3	-1.2	-1.23
ILMN_1349593	Srrm2_predicted	-1.1	-1.06	-2.59	-1.28	-1.16	-1.18	-1.23
ILMN_1362935	MGC94555	1.08	-1.15	-1.11	-1.12	-1.24	-1.17	-1.23
ILMN_1363742	Mrpl53_predicted	1.04	-1.04	-1.44	-1.14	-1.15	-1.17	-1.23
ILMN_2040532	LOC317464	-1.09	-1.24	-1.25	-1.32	-1.21	-1.16	-1.23
ILMN_1351139	LOC498279	-1.14	1.02	-1.14	-1.06	-1.04	-1.16	-1.23
ILMN_1353148	Glrx5_predicted	1	-1.26	-1.32	-1.11	-1.08	-1.14	-1.23
ILMN_1357981	Tmem7_predicted	-1.07	-1.22	-1.49	-1.69	-1.17	-1.13	-1.23

ILMN_1358138	Hdgf	1.08	1.1	-1.02	1.11	-1.24	-1.12	-1.23
ILMN_1369217	Thumpd1	-1.08	1.01	1.02	-1.01	-1.16	-1.12	-1.23
ILMN_1350899	Elovl7_predicted	1.09	-1.56	-1.07	-1.45	-1.1	-1.09	-1.23
ILMN_1368978	RGD1311045_predicted	1.08	-1.18	1.03	-1.24	-1.22	-1.06	-1.23
ILMN_1352893	Banp_predicted	1.06	-1.34	-1.19	1.39	-1.11	-1.04	-1.23
ILMN_1354076	RGD1564153_predicted	1.32	1.23	1.18	1.21	-1.04	-1.03	-1.23
ILMN_1353743	Hn1	-1.28	-1.31	-1.29	-1.22	-1.02	-1.03	-1.23
ILMN_1365669	Mark2	-1.15	1.21	1.06	1.1	-1.04	-1	-1.23
ILMN_1363830	RGD1309656_predicted	-1.04	-1.09	1.2	1.01	-1	1	-1.23
ILMN_1358388	Vezf1_predicted	-1.09	-1.12	-1.5	-1.61	-1.12	1.03	-1.23
ILMN_1360955	LOC366994	1.38	-1.08	-1.3	-1.13	-1.13	1.06	-1.23
ILMN_1353927	Dpf2_predicted	-1.23	1.02	-1.19	-1.03	-1.05	1.08	-1.23
ILMN_1363652	Zfp410_predicted	-1.15	1.03	1.02	1.01	1.21	1.11	-1.23
ILMN_1352529	Ier2	-1.07	1.38	-1.28	1.24	1.28	1.17	-1.23
ILMN_1364356	LOC688266	-1.56	-1.7	-1.5	-1.65	-1.64	-2.09	-1.22
ILMN_1352599	Nr1h3	-1.21	-1.13	-1.11	-1.57	-1.81	-1.8	-1.22
ILMN_1372031	Arfrp1	-1.02	-1.08	-1.21	-1.08	-1.45	-1.73	-1.22
ILMN_1366331	Habp4_predicted	-1.19	-1.61	-1.16	-1.18	-1.63	-1.64	-1.22
ILMN_1362642	Cd1d1	-1.28	-1.6	-1.51	-2.09	-1.46	-1.51	-1.22
ILMN_1367457	C1s	-1.03	-1.27	-1.43	-1.87	-1.34	-1.49	-1.22
ILMN_1366449	Bcl7c_predicted	-1.01	-1.64	-1.41	-1.32	-1.52	-1.46	-1.22
ILMN_1367523	Lgals1	1.15	1.02	-1.6	-1.07	-1.05	-1.45	-1.22
ILMN_1356307	Tmlhe	-1.08	-1.09	-1	-1.12	-1.45	-1.41	-1.22
ILMN_1355144	Agtpbp1_predicted	1.04	-1.16	-1.29	-1.02	-1.16	-1.4	-1.22
ILMN_1374604	Pts	1.03	-1.31	-1.13	-1.59	-1.21	-1.39	-1.22
ILMN_1364384	RGD1308331_predicted	-1.1	1.04	-1.2	1.09	-1.21	-1.39	-1.22
ILMN_1355497	Rnasen	-1.25	-1.33	-1.5	-1.43	-1.51	-1.37	-1.22
ILMN_1371688	Idh3B	-1.27	-1.07	-1.14	-1.18	-1.24	-1.36	-1.22
ILMN_1350848	Ndufa7_predicted	-1.22	-1.29	-1.15	-1.44	-1.19	-1.36	-1.22
ILMN_1372171	RGD1307583_predicted	1.1	1.06	-1.8	1.3	-1.31	-1.34	-1.22
ILMN_1361776	MGC72584	1.04	-1.08	1.04	1.01	-1.22	-1.31	-1.22
ILMN_1357518	Ppap2c	-1.09	1.01	1.04	-1.11	-1.37	-1.29	-1.22
ILMN_1376393	Clpb	1.07	1.15	-1.06	-1.16	-1.52	-1.25	-1.22
ILMN_1351197	Col11a2_mapped	-1.11	1.05	1.17	1.14	-1.07	-1.25	-1.22
ILMN_1360866	RGD1308143_predicted	1.07	-1	-1.09	1.01	-1.28	-1.22	-1.22
ILMN_1372159	Stat1	-1.09	-1.36	-1.31	-1.7	-1.06	-1.22	-1.22
ILMN_1353544	Sod1	-1.02	-1.32	-1.54	-1.45	-1.32	-1.21	-1.22
ILMN_1358314	Luzp1	-1.07	1.09	-1.03	1.23	-1.11	-1.19	-1.22

ILMN_1359632	LOC295452	1.09	-1.53	-1.1	-1.21	-1.13	-1.18	-1.22
ILMN_1350640	Rev1l_predicted	1.08	1.19	-1.03	1.19	-1.28	-1.15	-1.22
ILMN_2039717	Rev1l_predicted	1.08	1.19	-1.03	1.19	-1.28	-1.15	-1.22
ILMN_1359769	RGD1566122_predicted	-1.34	-1.14	-1.26	-1.21	-1.22	-1.13	-1.22
ILMN_1349844	Maea	-1.21	-1.13	-1.22	-1.14	-1.02	-1.13	-1.22
ILMN_1354765	Sirt3_predicted	-1.01	1.24	1.35	-1.03	-1.01	-1.13	-1.22
ILMN_1353143	LOC363188	-1.22	-1.35	-1.39	-1.24	-1.31	-1.12	-1.22
ILMN_1351776	Arntl	1.1	1.08	1.07	-1.04	-1.01	-1.12	-1.22
ILMN_1362691	Odf2	1.03	1.01	1.07	1.2	-1.29	-1.11	-1.22
ILMN_1362859	Mrpl22_predicted	-1.04	-1.07	-1.41	-1.1	-1.14	-1.11	-1.22
ILMN_1367804	Tmem18	-1.07	-1.17	1.05	-1.12	-1.09	-1.11	-1.22
ILMN_1357556	Palm	-1.12	-1.01	-1.03	-1.18	-1.08	-1.11	-1.22
ILMN_1363638	Zbed4_predicted	-1.07	1.03	1.03	1.12	-1.13	-1.1	-1.22
ILMN_1650537	Gpc4	-1.05	1.07	1.04	1.16	-1.16	-1.09	-1.22
ILMN_1351710	Fam3c	-1.08	-1.23	1.05	-1.13	-1.07	-1.08	-1.22
ILMN_1351366	Usp48	-1.2	-1.51	-1.26	-1.29	-1.26	-1.07	-1.22
ILMN_2039502	Tmem77	-1.08	-1.22	-1.16	-1.27	-1.21	-1.06	-1.22
ILMN_1358713	Usf1	-1.1	-1.2	-1.36	-1.43	-1.17	-1.06	-1.22
ILMN_1355272	Akap8	-1.15	1.04	1.04	1.09	-1.28	-1.05	-1.22
ILMN_1357624	Dlgh3	1	1.03	1.02	1.05	-1.12	-1.05	-1.22
ILMN_1367035	Lcat	-1.3	-1.11	-1.03	-1.02	1.05	-1.02	-1.22
ILMN_1359840	Ddx5	-1.3	-1.11	1.01	-1.18	-1.08	-1.01	-1.22
ILMN_1364072	Copz1_predicted	-1.01	-1.38	1.49	-1.26	1.12	1.05	-1.22
ILMN_1372510	Gpx1	2.23	1.14	1.07	1.24	1.31	1.06	-1.22
ILMN_1359177	Pdpk1	-1.01	-1.04	1.05	-1.1	-1	1.13	-1.22
ILMN_1357596	Fbxo34_predicted	1	1.05	1.11	-1.29	1.03	1.15	-1.22
ILMN_1354372	LOC498346	-1.09	1.05	1.06	1.02	1.2	1.15	-1.22
ILMN_1367112	Eps8l2_predicted	1.05	-1.64	-1.81	-1.59	-1.87	-1.92	-1.21
ILMN_1362837	Galk1	1.02	1.23	1.51	1.15	-1.26	-1.73	-1.21
ILMN_1374461	Mpp6_predicted	-1.04	1.18	1.06	-1.1	-1.54	-1.65	-1.21
ILMN_1372684	LOC501194	-1.1	-1.21	-1.23	-1.32	-1.66	-1.63	-1.21
ILMN_1373042	Sharpin	-1.3	-1.37	-1.38	-1.67	-1.58	-1.57	-1.21
ILMN_1354256	Tm2d2	-1.13	-1.53	-1.45	-1.56	-1.37	-1.53	-1.21
ILMN_1375793	LOC684448	-1.14	-1.33	1.01	-1.6	-1.37	-1.5	-1.21
ILMN_1368384	A3galt2	1.11	-1.29	-1.58	-1.65	-1.32	-1.48	-1.21
ILMN_1369440	Tinf2	-1.09	-1.42	-1.17	-1.45	-1.56	-1.46	-1.21
ILMN_1366518	Apoa1bp_predicted	-1.29	-1.28	-1.11	-1.36	-1.31	-1.45	-1.21
ILMN_1371255	Rarsl_predicted	-1	-1.36	1.08	-1.25	-1.34	-1.43	-1.21

ILMN_1353102	Akt1	-1.08	-1.36	-1.24	-1.49	-1.4	-1.41	-1.21
ILMN_1355874	RGD1565180_predicted	-1	-1.53	-1.42	-1.63	-1.39	-1.41	-1.21
ILMN_1359654	Dok4_predicted	-1.05	1.08	1.06	1.15	-1.37	-1.37	-1.21
ILMN_1366860	Dok4_predicted	-1.05	1.08	1.06	1.15	-1.37	-1.37	-1.21
ILMN_1365212	Mrpl54_predicted	-1.09	-1.65	-2.15	-1.6	-1.54	-1.36	-1.21
ILMN_1359027	RGD1563482_predicted	1.02	-1.15	-1.23	-1.35	-1.11	-1.36	-1.21
ILMN_1363445	Ctbs	-1.21	-1.09	-1.1	-1.31	-1.26	-1.34	-1.21
ILMN_1349212	RGD1309896_predicted	-1.19	-1.2	-1.1	1.1	-1.07	-1.33	-1.21
ILMN_1366178	RGD1562657_predicted	-1.05	-1.09	-1.03	-1.25	-1.12	-1.31	-1.21
ILMN_1650152	RGD1560961_predicted	1.11	-1.5	-1.32	-1.53	-1.46	-1.3	-1.21
ILMN_1372622	Lrrc8	-1.16	-1.31	-1.49	-1.28	-1.14	-1.3	-1.21
ILMN_1650806	RGD1307357_predicted	-1.07	1.26	1.37	1.38	-1.09	-1.29	-1.21
ILMN_1358569	LOC309362	-1.07	-1.33	-1.69	-1.55	-1.38	-1.28	-1.21
ILMN_1365426	Ap1gbp1	-1.24	-1.18	-1.2	-1.18	-1.21	-1.26	-1.21
ILMN_1373613	Lims1_predicted	1.05	-1.07	1.11	-1.24	-1.13	-1.26	-1.21
ILMN_1371280	Gstt2	-1.27	-1.56	-1.52	-1.66	-1.33	-1.25	-1.21
ILMN_1370051	Rad51l3_predicted	-1.04	1.05	1.07	-1.09	-1.32	-1.25	-1.21
ILMN_1375104	RGD1562335_predicted	-1.28	-1.14	1.05	-1.04	-1.3	-1.25	-1.21
ILMN_1359538	Pcaf	1.1	-1.23	-1.05	-1.47	-1.23	-1.25	-1.21
ILMN_1352457	RGD735029	1.13	-1.09	-1.04	-1.25	-1.27	-1.22	-1.21
ILMN_1349578	RGD1309720	1.24	-1.12	-1.1	-1.04	-1.14	-1.21	-1.21
ILMN_1357145	LOC308954	-1.03	1.06	1.1	-1.09	-1.21	-1.2	-1.21
ILMN_1350647	LOC498118	1.08	1.01	-1.09	1.07	-1.16	-1.2	-1.21
ILMN_1354659	Mmachc_predicted	-1.06	1.02	1.14	-1.11	-1.29	-1.19	-1.21
ILMN_1354746	Uqcrfs1	-1.14	-1.02	-1.05	-1.15	-1.17	-1.19	-1.21
ILMN_1376455	Mdm4	-1.08	1.83	1.23	1.24	-1.35	-1.18	-1.21
ILMN_1355313	Nid67	1.05	-1.18	-1.11	-1.16	-1.08	-1.18	-1.21
ILMN_1650575	RGD1310774_predicted	1.03	-1.3	1.02	-1.32	-1.1	-1.16	-1.21
ILMN_1366431	Nr2f2	1.09	-1.2	-1.41	-1.34	-1.42	-1.15	-1.21
ILMN_1349657	RGD1309765_predicted	-1.06	-1.03	-1.23	-1.27	-1.33	-1.15	-1.21
ILMN_1376771	Trub1	-1.04	1.18	1.37	1.15	-1.25	-1.14	-1.21
ILMN_1650198	RGD1562046_predicted	1	-1.16	1.26	1.02	-1.23	-1.14	-1.21
ILMN_1367435	Pdzd7_predicted	1.02	1.26	-1	1.29	-1.13	-1.14	-1.21
ILMN_1370461	RGD1308535_predicted	-1.15	-1.11	-1.54	-1.15	-1.1	-1.14	-1.21
ILMN_1349123	Top1	-1.11	-1.37	1.08	-1.06	-1.04	-1.14	-1.21
ILMN_1363930	Nsf	-1.01	-1.22	-1.19	-1.47	-1.72	-1.13	-1.21
ILMN_1367136	Eif2s2	1.02	1.01	1.12	1.02	-1.02	-1.12	-1.21
ILMN_1351843	Thap11_predicted	-1.06	1.03	-1.15	1.1	-1.18	-1.1	-1.21

ILMN_1355068	Tnpo3	-1.04	-1.15	-1.02	1.01	-1.1	-1.1	-1.21
ILMN_1355554	RGD1310609_predicted	1.02	1.02	1.08	1.33	-1.05	-1.09	-1.21
ILMN_1368601	Mtvr2	-1.03	-1.08	-1.13	-1.13	-1.04	-1.09	-1.21
ILMN_1365482	Zfp422_predicted	-1.13	-1.07	-1.09	-1.17	-1.25	-1.08	-1.21
ILMN_1355153	Gabpa_predicted	-1.11	-1.16	-1.28	-1.39	-1.19	-1.08	-1.21
ILMN_1367069	Mars_predicted	1.02	1.25	1.03	1.04	-1.1	-1.07	-1.21
ILMN_1349293	RGD1310835_predicted	1.14	1.03	-1.05	1.05	-1.06	-1.07	-1.21
ILMN_1352872	RGD1310835_predicted	1.14	1.03	-1.05	1.05	-1.06	-1.07	-1.21
ILMN_1371610	LOC245925	-1.11	1.11	-1.32	1.07	-1.08	-1.05	-1.21
ILMN_1650028	Recc1	-1.04	-1.25	-1.26	-1.21	-1.3	-1.03	-1.21
ILMN_1356046	Tdg	1	1.05	1.12	1.08	-1.2	-1.03	-1.21
ILMN_1351771	Cct3	1.05	1.15	1.29	1.17	-1.15	-1.03	-1.21
ILMN_1364154	LOC686275	1.05	-1.41	-1.1	-1.15	-1.04	-1.01	-1.21
ILMN_1352243	Zc3hc1_predicted	-1.25	-1.02	-1.11	-1.32	1.01	-1.01	-1.21
ILMN_1357106	Casp6	-1.17	-1.09	1.07	-1.1	-1.1	1.02	-1.21
ILMN_1369383	LOC305453	1.21	1.07	1.14	1	1.01	1.12	-1.21
ILMN_1366640	Exosc3_predicted	1.06	1.01	-1.02	1.16	1.15	1.13	-1.21
ILMN_1374460	Yars	1.09	1.25	1.55	1.47	1.28	1.19	-1.21
ILMN_1353094	Akr7a2	-1.19	-1.6	-1.23	-1.69	-1.67	-1.8	-1.2
ILMN_1349959	Pik3c2g	1.09	-1.13	-1.54	-1.47	-1.23	-1.58	-1.2
ILMN_1374479	Gpx4	1.14	-1.18	-1.42	-1.28	-1.42	-1.57	-1.2
ILMN_1359040	RGD1561110_predicted	-1.39	-1.47	-1.38	-1.56	-1.87	-1.56	-1.2
ILMN_1354588	Trappc1	-1.05	-1.15	-1.27	-1.19	-1.18	-1.51	-1.2
ILMN_1361425	Gnpat	1.03	-1.34	-1.22	-1.41	-1.87	-1.47	-1.2
ILMN_1356838	RGD735106	-1.06	-1.52	-1.3	-1.49	-1.35	-1.45	-1.2
ILMN_1530502	LOC684536	-1.01	-1.23	-1.43	-1.18	-1.6	-1.43	-1.2
ILMN_1376856	Pctk1	-1.11	-1.17	-1.1	-1.12	-1.17	-1.41	-1.2
ILMN_1650623	Phb2	-1	-1.24	1.05	-1.18	-1.58	-1.36	-1.2
ILMN_1358075	Rbm5	-1.04	-1.32	-1.52	-1.4	-1.43	-1.35	-1.2
ILMN_1376764	Gfm	1.07	-1.29	-1.35	-1.18	-1.27	-1.35	-1.2
ILMN_1352987	Dhrs8	-1	-1.54	-1.29	-2.34	-1.6	-1.34	-1.2
ILMN_1351667	LOC686765	-1.07	-1.32	-1.36	-1.33	-1.44	-1.33	-1.2
ILMN_1367792	Rffl	1.16	-1.04	-1.04	-1.14	-1.19	-1.32	-1.2
ILMN_1362688	Zfp191	-1.08	-1.07	-1.17	-1.2	-1.12	-1.31	-1.2
ILMN_1359269	Cobl_predicted	1.07	-1.03	-1.29	-1.22	-1.26	-1.28	-1.2
ILMN_1365510	RGD1306962_predicted	1.06	-1.23	1	-1.61	-1.16	-1.27	-1.2
ILMN_1368873	RGD1309707_predicted	-1.05	1.2	-1.06	1.07	-1.15	-1.26	-1.2
ILMN_1356578	Tcof1_predicted	-1.21	-1.02	1	1.4	-1.34	-1.25	-1.2

ILMN_1371431	Casp8	-1.15	-1.13	-1.18	-1.24	-1.18	-1.24	-1.2
ILMN_1366497	Yipf6	-1.07	-1.42	-1.34	-1.51	-1.46	-1.23	-1.2
ILMN_1364537	RGD1310352	-1.04	-1.33	-1.25	-1.37	-1.2	-1.23	-1.2
ILMN_1372879	Nfe2l2	-1.42	-1.19	-1.31	-1.51	-1.34	-1.19	-1.2
ILMN_1374743	RGD1311640_predicted	-1.23	-1.12	-1.51	-1.43	-1.39	-1.17	-1.2
ILMN_1375181	Hkr3	-1.17	1.11	1.38	1.26	-1.14	-1.17	-1.2
ILMN_1649841	Eral1	-1.03	-1.27	-1.34	-1.4	-1.03	-1.17	-1.2
ILMN_1376941	rnf141	1.02	-1.05	-1.06	-1.36	-1.4	-1.15	-1.2
ILMN_1358038	LOC364773	1.03	-1.09	-2.68	-1.31	-1.13	-1.15	-1.2
ILMN_1373236	LOC364773	1.03	-1.09	-2.68	-1.31	-1.13	-1.15	-1.2
ILMN_1372444	Chkb	-1.17	1.22	1.04	1.12	-1.09	-1.15	-1.2
ILMN_1367164	Paf1	-1.33	-1.33	-1.47	-1.26	-1.34	-1.14	-1.2
ILMN_1373322	Paics	1.13	1.01	1.15	-1.03	-1.2	-1.11	-1.2
ILMN_1352695	Znf629_predicted	1.01	1.23	1.03	1.1	-1.1	-1.11	-1.2
ILMN_1374058	Kmo	1.18	1.22	1.11	-1.17	-1.03	-1.11	-1.2
ILMN_1368316	RGD1565969_predicted	-1.02	-1.39	-1.16	-1.18	-1.22	-1.1	-1.2
ILMN_1373021	Sypl	1.08	-1.16	1.01	-1.22	-1.19	-1.1	-1.2
ILMN_1354823	Anapc7_predicted	-1.03	-1.36	1.18	-1.2	-1.06	-1.1	-1.2
ILMN_1367954	Ddx39	-1.03	-1.17	-1.03	-1.01	-1.03	-1.1	-1.2
ILMN_1350584	Syncrip	-1.11	-1.07	-1	1.13	-1.32	-1.09	-1.2
ILMN_1376804	Qars	1.12	1.08	1.29	-1.06	-1.2	-1.09	-1.2
ILMN_1351965	LOC499890	-1.1	-1.1	-1.04	-1.32	-1.13	-1.09	-1.2
ILMN_1352152	RGD1562795_predicted	-1.09	-1.08	1.05	1.03	-1.1	-1.09	-1.2
ILMN_1368783	RGD1562076_predicted	-1.03	-1.1	-1.19	-1.36	-1.2	-1.07	-1.2
ILMN_1362369	RGD1308848_predicted	1.07	-1.06	-1.03	1.01	-1.29	-1.05	-1.2
ILMN_1376645	Tssc1	-1.02	-1.2	1.16	-1.32	-1.15	-1.03	-1.2
ILMN_1373118	Yes1	1.06	-1.19	1.04	-1.05	-1.03	-1.02	-1.2
ILMN_1369915	Dyt1	-1.12	-1.1	-1.15	-1.05	-1.01	-1.02	-1.2
ILMN_1374415	Gabpb1_predicted	-1.01	1.02	1.06	-1.1	1.12	1	-1.2
ILMN_1353885	Zfp219	-1.13	1.27	-1.41	-1.04	1.07	1.04	-1.2
ILMN_1350712	Usp47_predicted	1	1.13	1.18	1.07	1.2	1.07	-1.2
ILMN_1368201	LOC499897	1	-1.17	-1.07	-1.2	1.15	1.08	-1.2
ILMN_1370810	Hdlbp	1.08	1	1.03	1.07	1.27	1.12	-1.2
ILMN_1363928	Ptbp2	1.31	1.19	1.22	1.01	-1.05	1.15	-1.2
ILMN_1365223	RGD1359600	1	1.31	1.11	1.06	1.09	1.15	-1.2
ILMN_1366515	Tjp2	-1.12	-1.13	-1.1	-1.15	1.26	1.15	-1.2
ILMN_1371604	Prcc_predicted	-1.06	-1.02	-1.14	1.01	1.1	1.19	-1.2
ILMN_1367911	Abcg4	-1.1	-1.01	1.01	-1.04	-1.06	1.2	-1.2

ILMN_1362580	Sstr2	1.09	-1.03	1.08	-1.12	1.07	1.28	-1.2
ILMN_1364324	Fech_predicted	1.01	-1.75	-1.5	-1.83	-1.63	-1.75	-1.19
ILMN_2040341	Fech_predicted	1.01	-1.75	-1.5	-1.83	-1.63	-1.75	-1.19
ILMN_1369787	Mrps25	-1.01	-1.33	-1.83	-1.43	-1.79	-1.68	-1.19
ILMN_1361813	Ndufs3_predicted	-1.1	-1.38	-1.34	-1.51	-1.49	-1.54	-1.19
ILMN_1372190	Pqlc2_predicted	1.01	-1.18	-1.23	-1.01	-1.39	-1.53	-1.19
ILMN_1358621	Adprh	1.05	-1.21	-1.37	-1.1	-1.55	-1.51	-1.19
ILMN_1373337	Pik4ca	-1.07	-1.04	-1.28	-1.22	-1.27	-1.49	-1.19
ILMN_1350236	Rbm19_predicted	-1.4	-1.31	-1.29	-1.34	-1.51	-1.48	-1.19
ILMN_1350546	Gmppb_predicted	-1.2	-1.37	-1.26	-1.27	-1.1	-1.48	-1.19
ILMN_1354636	RGD620382	1.14	1.11	-1.05	1.12	-1.45	-1.44	-1.19
ILMN_2039266	Gcsh	-1.11	-1.42	-1.15	-1.27	-1.4	-1.43	-1.19
ILMN_1374160	Calm3	-1.08	-1.4	-1.42	-1.23	-1.36	-1.43	-1.19
ILMN_1356597	LOC362304	-1	-1.14	1.01	-1.46	-1.38	-1.4	-1.19
ILMN_1353956	Tapbp	-1.11	-1.13	1.09	-1.22	-1.3	-1.37	-1.19
ILMN_1367441	LOC362809	-1.05	-1.94	-1.61	-1.31	-1.28	-1.34	-1.19
ILMN_1352692	RGD1311017_predicted	1.2	-1.17	-1.09	1.15	-1.29	-1.31	-1.19
ILMN_1354138	Scamp3	1.03	-1.42	-1.3	-1.37	-1.37	-1.28	-1.19
ILMN_1359643	Ict1_predicted	1.02	-1	-1.31	-1.01	-1.25	-1.28	-1.19
ILMN_1363640	Trpt1_predicted	-1.1	1	-1.13	-1.01	-1.25	-1.27	-1.19
ILMN_1349245	RGD1306596	1.11	-1.07	-1.15	-1.17	-1.05	-1.26	-1.19
ILMN_1364932	Gsdmdc1_predicted	1	-1	-1.18	-1.3	-1.23	-1.25	-1.19
ILMN_1349029	Prkcsh_predicted	-1.03	-1.02	1.4	-1.11	-1.22	-1.25	-1.19
ILMN_1373359	U2af1l4	-1.15	-1.31	-1.45	-1.32	-1.39	-1.24	-1.19
ILMN_1357694	Dhx30	-1.11	1.26	-1.08	1.07	-1.33	-1.24	-1.19
ILMN_1373829	Fxna	1.05	-1	1.34	-1.08	-1.15	-1.24	-1.19
ILMN_1362797	RGD1310439_predicted	1.01	-1.17	-1.16	-1.09	-1.02	-1.24	-1.19
ILMN_1355841	Abhd8_predicted	-1.01	1.22	1.21	1.19	-1.13	-1.22	-1.19
ILMN_1375038	Efnb1	-1.16	-1.17	-1.03	-1.03	-1.4	-1.2	-1.19
ILMN_1354987	Stno_predicted	-1.15	-1.01	1	1.24	-1.08	-1.2	-1.19
ILMN_1363490	Npm1	1.01	-1.02	1.32	-1.19	-1.35	-1.19	-1.19
ILMN_1363486	Casp7	1.08	-1.06	1.22	-1.1	-1.18	-1.19	-1.19
ILMN_1372828	Brd4	-1.11	-1.07	-1.17	-1.14	-1.16	-1.19	-1.19
ILMN_1374756	RGD1306674_predicted	-1	1.22	1.1	1.04	-1.1	-1.18	-1.19
ILMN_1355215	Commd10	-1.12	-1.45	-1.35	-1.5	-1.24	-1.17	-1.19
ILMN_1361378	Strn4_predicted	-1.22	-1.07	-1.1	1.04	-1.21	-1.17	-1.19
ILMN_1363744	Rnf3_predicted	1.03	1.09	1.17	1.04	-1.32	-1.16	-1.19
ILMN_1355100	LOC361473	1.12	1.09	-1.17	-1.01	-1.18	-1.14	-1.19

ILMN_1362435	Ccrl2_predicted	1.02	-1.03	-1.05	-1.32	-1.2	-1.12	-1.19
ILMN_1650486	LOC313934	-1.11	-1	1.04	-1.06	-1.25	-1.1	-1.19
ILMN_1359189	Sf3b3_predicted	1.05	-1.08	-1.25	1.09	-1.23	-1.09	-1.19
ILMN_1370042	Ankrd32_predicted	1.03	-1.09	-1.16	-1.08	-1.29	-1.08	-1.19
ILMN_1362054	Cfl1	-1.02	-1.19	-1.11	1.02	-1.1	-1.07	-1.19
ILMN_1359049	Ilkap	-1.08	1.05	1.01	1.08	-1.2	-1.04	-1.19
ILMN_1352910	Trp53bp1_predicted	-1.28	1.1	-1.43	1.09	-1.07	-1.03	-1.19
ILMN_1372178	LOC363849	1.14	-1.08	1.4	1.18	-1.26	-1.02	-1.19
ILMN_1354875	LOC497991	1.01	1.12	1.08	-1.03	-1.13	-1.01	-1.19
ILMN_1367177	Ptpn18	1.14	1.09	-1.16	1.17	-1.03	-1.01	-1.19
ILMN_1371790	Tollip_predicted	-1.16	-1.19	1.05	-1.07	-1.07	1.02	-1.19
ILMN_1370879	Axin1	1	1.12	1.12	1.28	1.09	1.02	-1.19
ILMN_1374926	Acvr2b	1.1	1.23	1.26	-1.01	-1.24	1.03	-1.19
ILMN_1360989	Higd2a_predicted	1	-1.47	-1.16	-1.01	1.03	1.04	-1.19
ILMN_1371020	Dusp11_predicted	1.01	1.06	-1.03	1.16	1.12	1.05	-1.19
ILMN_1373917	Map3k12	1.02	-1.09	-1.01	1.05	1.04	1.07	-1.19
ILMN_1351717	Rev3l	-1.04	-1.14	-1.21	-1.1	-1.04	1.08	-1.19
ILMN_1348998	Akt1s1_predicted	-1.17	-1.13	1.08	-1.3	1.19	1.08	-1.19
ILMN_1359095	Mat2a	1.09	1.22	1.37	1.17	-1.11	1.11	-1.19
ILMN_1359024	Myd88	-1.12	-1.23	-1.47	-1.45	-1.01	1.12	-1.19
ILMN_1361578	Cdh17	1.04	1.31	1.45	1.48	1.21	1.12	-1.19
ILMN_1360001	Vkorc1l1	-1.02	1.13	1.05	1.13	1.04	1.16	-1.19
ILMN_1361494	LOC498901	1.02	1.06	1.17	1.02	1.15	1.16	-1.19
ILMN_1368034	Osbpl11_predicted	-1.15	1.02	1.26	1.28	1.07	1.19	-1.19
ILMN_1356538	RGD1304842_predicted	-1.1	-1.13	-1.19	-1.58	1.41	1.3	-1.19
ILMN_1355426	Ppp1r10	-1.14	-1.33	-1.2	-1.65	1.53	1.42	-1.19
ILMN_1360145	Sra1	-1.2	-1.15	-1.13	-1.28	-1.36	-1.61	-1.18
ILMN_1375183	Slc16a13	-1.07	-1.19	-1.1	-1.2	-1.53	-1.57	-1.18
ILMN_1353279	Meis2_predicted	-1.28	-1.35	-1.47	-1.61	-1.34	-1.55	-1.18
ILMN_1365772	RGD1562411_predicted	-1.13	-1.31	-1.15	-1.04	-1.29	-1.51	-1.18
ILMN_1349702	RGD1565485_predicted	1.07	-1.06	-1.28	-1.45	-1.6	-1.48	-1.18
ILMN_1365002	Abhd1	-1.18	-1.36	-1.22	-1.41	-1.45	-1.48	-1.18
ILMN_1357817	LOC502617	-1.18	-1.14	-1.34	-1.08	1	-1.46	-1.18
ILMN_1351983	RGD1564560_predicted	-1.4	-1.75	-3.49	-2.49	1.42	-1.46	-1.18
ILMN_1361471	Abcb7	-1	1.09	-1.15	-1.15	-1.5	-1.42	-1.18
ILMN_1374947	Bpnt1	1.17	-1.05	-1.65	1.06	-1.16	-1.37	-1.18
ILMN_1358441	Gpr125_predicted	-1.1	-1.03	-1.22	-1.12	-1.27	-1.35	-1.18
ILMN_1350697	Slc25a15	-1	-1.45	-1.21	-1.71	-1.1	-1.35	-1.18

ILMN_1354275	Rad50	-1.14	-1.12	-1.02	-1.08	-1.4	-1.34	-1.18
ILMN_1372100	RGD1564605_predicted	-1.25	-1.32	-1.26	-1.36	-1.31	-1.31	-1.18
ILMN_1360373	Ptpn6	-1.17	-1.17	-1.28	-1.41	-1.41	-1.3	-1.18
ILMN_1349837	Tiam1	-1.04	-1.26	-1.14	-1.28	-1.55	-1.29	-1.18
ILMN_1358048	Capn7_predicted	-1.09	-1.21	-1.33	-1.15	-1.37	-1.27	-1.18
ILMN_1371776	Haao	-1.03	-1.21	-1.05	-1.41	-1.15	-1.27	-1.18
ILMN_1369751	Bad	-1.17	-1.33	-1.54	-1.32	-1.34	-1.25	-1.18
ILMN_1353304	RGD1561287_predicted	-1.08	-1.17	-1.26	-1.36	-1.22	-1.25	-1.18
ILMN_1358838	Fut2	-1.24	-1.02	-1.09	-1.55	-1.22	-1.24	-1.18
ILMN_1366152	Cyp4f6	-1.05	1.12	-1.03	-1.09	1.01	-1.23	-1.18
ILMN_1363087	Rasl11b	-1.08	-1.51	-1.27	-1.25	-1.09	-1.22	-1.18
ILMN_1357958	Ncor1	-1.28	1.03	1.06	-1.08	-1.07	-1.22	-1.18
ILMN_1373549	Bbs5_predicted	-1.16	1.03	-1.22	-1.1	-1.3	-1.21	-1.18
ILMN_1359429	LOC301130	-1.21	-1.28	-1.36	-1.18	-1.24	-1.21	-1.18
ILMN_1362936	Cnbp1	-1.01	-1.02	1.2	-1.04	-1.16	-1.19	-1.18
ILMN_1357549	Tnip1_predicted	-1.26	-1.63	-1.82	-2.3	-1.36	-1.16	-1.18
ILMN_1370365	Elavl1_predicted	-1.09	-1.1	-1.16	-1.02	-1.28	-1.16	-1.18
ILMN_1366983	Pelp1	-1.08	-1	1.01	1.04	-1.27	-1.16	-1.18
ILMN_1369198	Prep	1.08	-1.03	1.11	-1.04	-1.28	-1.14	-1.18
ILMN_1358272	LOC497735	-1.25	-1.38	-1.33	-1.23	-1.24	-1.14	-1.18
ILMN_1376320	Psma7	1	-1.17	-1.28	-1.17	-1.13	-1.14	-1.18
ILMN_1359077	Rarb	1.03	1.06	-1.06	-1.04	-1.11	-1.14	-1.18
ILMN_1367764	LOC292811	-1.07	-1.04	-1.31	-1.06	-1.32	-1.13	-1.18
ILMN_1374031	RGD1306783	-1.1	-1.09	1.05	-1.05	-1.31	-1.13	-1.18
ILMN_1364784	Zfp111	-1.09	1.06	-1.17	1.06	-1.22	-1.13	-1.18
ILMN_1370277	LOC503172	-1.17	-1.66	-1.05	-1.89	-1.35	-1.11	-1.18
ILMN_1372183	Mtmr4_predicted	-1	-1.16	-1.25	1.02	-1.29	-1.09	-1.18
ILMN_1373107	Smarca3_predicted	1.06	-1.26	-1.57	-1.47	-1.2	-1.08	-1.18
ILMN_1357379	Smarca3_predicted	1.06	-1.26	-1.57	-1.47	-1.2	-1.08	-1.18
ILMN_1360282	Yars2	1.18	1.16	1.08	1.41	-1.15	-1.07	-1.18
ILMN_1365196	Sertad1	-1.27	-1.34	-1.16	-1.26	-1.03	-1.07	-1.18
ILMN_1374217	Syf2	-1.11	-1.61	-1.56	-2.08	-1.22	-1.06	-1.18
ILMN_1376571	Sfxn1	-1.08	-1.2	1.18	-1.24	-1.14	-1.06	-1.18
ILMN_1355243	LOC310839	-1.11	1.03	-1.07	1.09	-1.11	-1.06	-1.18
ILMN_1351925	Zfp294	1.05	1	-1.12	-1.09	-1.1	-1.05	-1.18
ILMN_1355808	LOC501706	-1.21	-1.33	-1.3	-1.4	-1.04	-1.05	-1.18
ILMN_1349827	Ppm2c	-1.06	-1.28	-1.17	-1.21	1.02	-1.04	-1.18
ILMN_1356642	Narf	-1.13	-1.13	-1.02	-1.31	1.02	-1.03	-1.18

ILMN_1650330	Hexim2_predicted	-1.27	-1.1	-1.22	-1.45	-1.19	1	-1.18
ILMN_1376830	Creb3l2	-1.12	-1.18	-1.29	-1.27	-1.23	1.03	-1.18
ILMN_1356667	Gtf2e1	1.07	-1.03	1.26	1.19	-1.1	1.03	-1.18
ILMN_1366545	Tegt	-1.54	-2.1	-1.44	-1.94	-1.03	1.04	-1.18
ILMN_1353174	Smarcad1_predicted	1.1	1.04	1.17	-1.01	-1.16	1.05	-1.18
ILMN_1360442	Yt521	-1.12	1.15	1.17	-1.21	1.09	1.09	-1.18
ILMN_1358268	Wdr33_predicted	-1.1	-1.2	-1.2	-1.41	-1.01	1.12	-1.18
ILMN_1360168	RGD1561878_predicted	-1.04	1.1	-1.02	-1.01	1.02	1.21	-1.18
ILMN_1348882	Nvl_predicted	-1.07	-1.09	-1.07	1.19	1.38	1.21	-1.18
ILMN_1357872	Pomgnt1	-1.05	1.16	1.32	1.12	-1.67	-1.87	-1.17
ILMN_1650776	Igsf11	-1.1	-1.49	-1.56	-1.86	-1.37	-1.79	-1.17
ILMN_1359985	Polr3h_predicted	-1.05	-1.42	-1.33	-1.28	-1.68	-1.78	-1.17
ILMN_1353365	Hmbs	1.05	-1.5	-1.64	-1.39	-1.52	-1.72	-1.17
ILMN_1374710	Oplah	1.06	-1.16	-1.42	-1.43	-1.35	-1.61	-1.17
ILMN_1360976	RGD1305547_predicted	-1.25	-1.34	-1.48	-1.31	-1.15	-1.43	-1.17
ILMN_1356895	Ttc13	-1.01	-1.1	1.06	1.06	-1.41	-1.4	-1.17
ILMN_1357461	LOC499094	-1.11	1.04	-1.06	-1.19	-1.46	-1.35	-1.17
ILMN_1351249	Spast_predicted	1.01	1.02	-1.25	-1.06	-1.31	-1.35	-1.17
ILMN_1357905	Extl2	1.11	-1.15	1.18	1.24	-1.21	-1.35	-1.17
ILMN_1373483	Tomm40	-1.04	-1.11	1.17	1.21	-1.23	-1.34	-1.17
ILMN_1649961	RGD1559682_predicted	-1.05	-1.59	-1.71	-1.25	-1.24	-1.32	-1.17
ILMN_1364158	Prdx2	-1.21	-1.16	-1.13	-1.14	-1.21	-1.32	-1.17
ILMN_1360128	LOC500855	-1.11	-1.58	-1.15	-1.63	-1.19	-1.32	-1.17
ILMN_1357594	RGD1560410_predicted	1.05	-1.06	1.02	-1.13	-1.22	-1.3	-1.17
ILMN_1650363	Nsun5_predicted	-1.17	1.09	1.58	1.05	-1.31	-1.26	-1.17
ILMN_1373596	Dag1	-1.23	-1.25	-1.44	-1.16	-1.25	-1.26	-1.17
ILMN_1368592	LOC362557	1.01	-1.1	-1.01	-1.05	-1.32	-1.25	-1.17
ILMN_1374434	Ddx52	-1.08	-1.09	-1.02	1.14	-1.22	-1.21	-1.17
ILMN_1650357	Rabl2a	1.06	1.01	1.01	-1.22	-1.14	-1.21	-1.17
ILMN_1364439	Zfp533_predicted	-1.05	-1.25	-1.42	-2.04	-1.12	-1.2	-1.17
ILMN_1370539	Tarbp2	-1.16	-1.11	-1.18	-1.17	-1.07	-1.2	-1.17
ILMN_1351358	RGD1308396_predicted	-1.13	1	-1.16	1.11	-1.18	-1.16	-1.17
ILMN_1368069	Ipp_predicted	1.02	-1.12	-1.06	-1.01	-1.2	-1.15	-1.17
ILMN_1350864	Tjp3_predicted	-1.23	-1.05	1.07	-1.08	-1.06	-1.15	-1.17
ILMN_1372598	RGD1565983_predicted	-1.16	-1.33	-1.61	-1.32	-1.38	-1.13	-1.17
ILMN_1357221	Taf15_predicted	1.05	-1.19	-2.67	-1.35	1.1	-1.13	-1.17
ILMN_1368504	Acsl3	1.09	-1.31	-1.31	-1.31	-1.13	-1.12	-1.17
ILMN_1367402	Impa2	-1.01	1.13	1.52	1.12	-1.08	-1.12	-1.17

ILMN_1361759	Msto1_predicted	1.04	1	1.03	1.04	-1.07	-1.12	-1.17
ILMN_1373228	Msto1_predicted	1.04	1	1.03	1.04	-1.07	-1.12	-1.17
ILMN_1350181	Set_predicted	1.09	-1.06	1.27	-1.02	-1.39	-1.11	-1.17
ILMN_1353175	Mkl1_predicted	-1.48	-1.35	-1.25	-1.29	-1.03	-1.11	-1.17
ILMN_1352978	Trak2	1.03	-1.04	-1.27	-1.16	-1	-1.11	-1.17
ILMN_1366589	Mtx2	-1.11	-1.14	1.04	-1.08	-1.12	-1.09	-1.17
ILMN_1354976	Glt25d1_predicted	1.04	1.02	-1.07	1.08	-1.11	-1.09	-1.17
ILMN_1350021	Zfp653_predicted	-1.17	1.13	-1.16	-1.02	-1.09	-1.08	-1.17
ILMN_1351670	Thtpa	-1.03	-1.03	-1.28	-1.25	-1.07	-1.08	-1.17
ILMN_1650898	Man1a2_predicted	1.14	-1.02	-1.13	-1.14	-1.29	-1.07	-1.17
ILMN_1361279	Fln29	-1.26	-1.43	-1.27	-1.67	-1.27	-1.07	-1.17
ILMN_1350028	Smad5	-1.02	1.25	-1.12	-1.04	-1.11	-1.07	-1.17
ILMN_1349840	Deadc1_predicted	1.09	1.2	1.05	1.01	-1.05	-1.07	-1.17
ILMN_1376629	Fkbp5	1.29	1.14	1.49	1.43	-1.2	-1.06	-1.17
ILMN_1350654	Tor1aip1	-1.19	-1.48	-1.32	-1.6	-1.46	-1.04	-1.17
ILMN_1366381	Armc1_predicted	-1.04	-1.38	-1.22	-1.28	1.18	-1.04	-1.17
ILMN_1351729	Mbc2	-1.09	-1.03	1.05	1.1	1.18	-1.04	-1.17
ILMN_1365464	LOC304920	-1.12	-1.02	-1.11	-1.2	-1.27	-1.03	-1.17
ILMN_1362627	Rpgrip1	-1.07	-1.07	-1.01	1.02	-1.2	-1.03	-1.17
ILMN_1368671	Shmt2	-1.04	-1.07	1.44	1.03	-1.09	-1.02	-1.17
ILMN_1354303	Tram1	-1.12	-1.54	-1.07	-1.37	1.08	-1.02	-1.17
ILMN_1360701	RGD1308847_predicted	-1.15	-1.08	-1.2	-1.02	1.14	-1.02	-1.17
ILMN_1365554	Rexo1	-1.14	1.22	-1.11	1.02	1.02	-1.01	-1.17
ILMN_1371930	Cdc42	-1.16	-1.18	-1.26	-1.18	-1.06	-1	-1.17
ILMN_1361287	Surb7_predicted	-1.01	-1.14	-1.21	1.03	1.01	1	-1.17
ILMN_1356140	LOC501170	1.38	-1.05	1.15	1.32	-1.14	1.02	-1.17
ILMN_1369835	Golga7	-1.13	-1.01	-1.03	-1.09	1.08	1.03	-1.17
ILMN_1372952	RGD1566204_predicted	-1.05	1.16	-1	1.02	-1.17	1.04	-1.17
ILMN_1362551	Tssc4	1.17	1.11	1.26	1.18	-1.04	1.04	-1.17
ILMN_1373793	Dhrs7b	1	-1.17	-1.03	-1.13	-1	1.04	-1.17
ILMN_1349638	RGD1561961_predicted	-1.08	-1.01	-1.03	-1.16	1.1	1.04	-1.17
ILMN_1370487	Za20d3	-1.25	-1.01	-1.1	-1.46	1.11	1.07	-1.17
ILMN_1375093	Zfp263_predicted	-1.23	-1.03	-1.01	1.01	1.01	1.1	-1.17
ILMN_1367448	LOC305466	1.28	1.13	1.68	1.29	1.07	1.12	-1.17
ILMN_1366672	Kpna3	1.37	1.07	1.12	1.2	-1.01	1.21	-1.17
ILMN_1376356	LOC191574	1.18	-1.31	-2.12	-2.22	-1.44	-1.88	-1.16
ILMN_2039341	Tspan4	1.28	-1.42	1.69	1.02	-2.02	-1.83	-1.16
ILMN_1357139	Ikbke_predicted	-1.22	1.08	-1.01	-1.22	-1.6	-1.64	-1.16

ILMN_1372600	Hsd17b12	-1.14	-1.25	-1.16	-1.3	-1.32	-1.55	-1.16
ILMN_1363913	Apba3	-1.08	1.15	-1.44	1.01	-1.38	-1.53	-1.16
ILMN_1369070	LOC286989	1.68	-1.19	-1.18	-1.15	-1.88	-1.49	-1.16
ILMN_1375936	Rangap1_predicted	1.08	-1.45	-1.18	-1.12	-1.25	-1.49	-1.16
ILMN_1373227	Gtf2f1	-1.36	-1.16	-1.1	-1.1	-1.36	-1.48	-1.16
ILMN_1359173	LOC502782	-1.13	-1.39	-1.12	-1.68	-1.49	-1.47	-1.16
ILMN_1356119	Prkra	-1.16	-1.12	-1.18	-1.2	-1.46	-1.45	-1.16
ILMN_1351829	Gyltl1b	-1.09	-1.07	-1.25	1.02	-1.47	-1.41	-1.16
ILMN_1353356	Pigt_predicted	-1.19	-1.53	-1.02	-1.45	-1.48	-1.4	-1.16
ILMN_1373186	LOC299828	1.02	-1.11	1.18	1.02	-1.29	-1.38	-1.16
ILMN_1358687	Pgcp	-1.07	1.23	1.23	-1.03	-1.21	-1.37	-1.16
ILMN_1372771	LOC290555	-1.05	-1.16	-1.35	-1.2	-1.33	-1.35	-1.16
ILMN_1352209	Cd14	1.03	-1.04	-1.06	-1.31	-1.15	-1.34	-1.16
ILMN_1364781	Mrpl44	-1.01	-1	-1.28	-1.4	-1.52	-1.33	-1.16
ILMN_1368606	Exosc7	1.14	-1.05	-1.04	1.14	-1.38	-1.3	-1.16
ILMN_1357400	Glt8d1	-1.21	-1.19	-1.28	-1.44	-1.48	-1.29	-1.16
ILMN_1355751	Zfp523_predicted	-1.23	-1.29	-1.06	-1.63	-1.33	-1.29	-1.16
ILMN_1369090	Tm2d3_predicted	1.32	-1.17	-1.45	-1.42	-1.24	-1.27	-1.16
ILMN_1369273	Marveld2_predicted	-1.05	-1.05	-1.1	-1.02	-1.3	-1.26	-1.16
ILMN_1372395	RGD1309038	-1.04	-1.48	-1.16	-1.23	-1.27	-1.26	-1.16
ILMN_1355840	Psmc1	-1.03	-1.26	-1.05	-1.17	-1.26	-1.26	-1.16
ILMN_1369795	Immt_predicted	1.01	-1.06	-1.25	-1.38	-1.23	-1.25	-1.16
ILMN_1354751	Leng4_predicted	1.04	-1.22	-1.06	-1.29	-1.24	-1.24	-1.16
ILMN_1364584	Bucs1_predicted	1.14	-1.2	1.47	-1.28	1.03	-1.24	-1.16
ILMN_1371461	Hnrph3_predicted	-1.18	-1.1	-1	-1.3	-1.26	-1.2	-1.16
ILMN_1356611	Zswim3_predicted	-1.07	-1.29	-1.04	-1.1	-1.26	-1.2	-1.16
ILMN_1366427	Fis1	-1.1	-1.31	-1.7	-1.36	-1.35	-1.19	-1.16
ILMN_1358019	Snx14_predicted	-1.23	-1.04	1	-1.23	-1.04	-1.18	-1.16
ILMN_1362374	Mtf2	1.16	-1.04	-1.23	1.01	-1.18	-1.17	-1.16
ILMN_1369270	Prpsap2	-1.06	1.09	1.17	-1.13	-1.16	-1.17	-1.16
ILMN_1372406	Suclg1	-1.13	-1.22	1.04	-1.31	-1.09	-1.17	-1.16
ILMN_1358251	Usp11	1.03	-1.04	-1.07	-1.35	-1.04	-1.17	-1.16
ILMN_1355882	LOC500469	-1.12	1.17	1.29	1.07	1.05	-1.17	-1.16
ILMN_1371689	RGD1310230	-1.17	-1.3	-1.33	-1.45	-1.16	-1.15	-1.16
ILMN_1367452	Mcart1	-1.1	1.01	1.38	-1.09	-1.15	-1.15	-1.16
ILMN_1355805	Inpp5e	-1.09	-1.06	1.01	1.07	-1.18	-1.14	-1.16
ILMN_1371250	Nudcd1_predicted	1.17	-1.09	1.15	-1.17	-1.12	-1.14	-1.16
ILMN_1351826	Ippk	-1.08	-1.02	1.24	1.18	-1.01	-1.12	-1.16

ILMN_1372773	Gapdh	-1.25	-1.51	-1.22	-1.2	-1.03	-1.09	-1.16
ILMN_2039222	Gapdh	-1.25	-1.51	-1.22	-1.2	-1.03	-1.09	-1.16
ILMN_1649859	Gapdh	-1.25	-1.51	-1.22	-1.2	-1.03	-1.09	-1.16
ILMN_1358989	RGD1307009	1.16	1.21	1.22	1.17	-1.23	-1.08	-1.16
ILMN_1373017	Xbp1	-1.33	1.24	1.11	-1.17	-1.17	-1.08	-1.16
ILMN_1353469	LOC363309	-1.11	-1.03	1	-1.04	-1.13	-1.08	-1.16
ILMN_1374906	RGD1562758_predicted	-1.05	-1.48	-1.31	-1.21	-1.02	-1.07	-1.16
ILMN_1365228	M6pr	-1.07	-1.18	-1.24	-1.13	-1.01	-1.07	-1.16
ILMN_1355883	Tnfaip1	1.11	1.04	1.01	-1.25	1	-1.07	-1.16
ILMN_1349345	Fbxo11	-1.15	-1.04	1.16	1.08	1.13	-1.07	-1.16
ILMN_1356142	RGD1560783_predicted	1.11	-1.19	1.04	-1.13	-1.13	-1.04	-1.16
ILMN_1376991	Dhodh	-1.01	-1.21	-1.14	1.02	1.1	-1.02	-1.16
ILMN_1368894	Ppp1cb	1.02	-1.14	1	-1.26	-1.08	-1.01	-1.16
ILMN_1360112	Nudcd2	-1.06	-1.42	1.1	-1.06	-1.15	-1	-1.16
ILMN_1363656	Znf14	1.13	1.07	1.14	-1.03	1.27	1.01	-1.16
ILMN_1362579	Sumo1	-1.02	-1.26	-1.17	-1.43	-1.05	1.02	-1.16
ILMN_1369531	Atad1	1.25	1.01	1.99	1.19	-1.13	1.03	-1.16
ILMN_1358924	LOC501116	1.14	-1.02	1.24	1.19	-1.09	1.04	-1.16
ILMN_1368987	LOC501098	1.13	-1.26	-1.06	-1.17	1.05	1.05	-1.16
ILMN_1359895	Ppp4c	-1.09	-1.03	1.05	1.07	1.05	1.05	-1.16
ILMN_1376810	Enpp2	-1.17	-1.2	-1.14	-1.42	1.47	1.06	-1.16
ILMN_1370508	RGD1564603_predicted	-1.12	-1.24	-1.33	-1.15	1.07	1.07	-1.16
ILMN_1375179	Ak3l1	-1.18	1.51	1.23	1.38	1.23	1.08	-1.16
ILMN_1356548	LOC501548	-1.06	-1.01	-1.06	-1.01	1.21	1.09	-1.16
ILMN_1365254	Gdf1_predicted	1.05	1.07	-1.2	1.09	1.13	1.1	-1.16
ILMN_1367638	Mtmr2_predicted	1.02	-1.08	1.17	-1.07	-1.1	1.11	-1.16
ILMN_1367682	Ube2z	1.1	-1.1	1.05	1.15	1.16	1.12	-1.16
ILMN_1369504	RGD1309809	-1.09	-1.28	-1.11	-1.41	1.03	1.15	-1.16
ILMN_1371632	Zfp688_predicted	-1.01	-1.05	-1.16	1.01	1.15	1.15	-1.16
ILMN_1366265	Tp53	-1.24	-1.23	-1.37	-1.04	1.1	1.33	-1.16
ILMN_1361521	LOC362015	-1.04	1.42	1.47	1.46	1.32	1.38	-1.16
ILMN_1374511	Soat2	1.06	2.21	1.7	1.83	1.58	1.53	-1.16
ILMN_1371282	RGD1304587	-1.09	-1.15	-1.33	-1.06	-1.33	-1.57	-1.15
ILMN_1360780	Ugt1a6	1.15	-1.37	1.83	-1.24	-1.81	-1.46	-1.15
ILMN_1650034	Tsen2	-1.04	1.06	-1.01	1.25	-1.42	-1.38	-1.15
ILMN_1373637	Dgkz	-1.1	-1.06	-1.41	-1.07	-1.45	-1.36	-1.15
ILMN_1350924	Ndufb6_predicted	1.02	-1.4	-1.54	-1.18	-1.28	-1.36	-1.15
ILMN_1361209	Cyb5	-1.51	-1.42	-2.14	-2.01	-1.34	-1.34	-1.15

ILMN_1354054	Smpd1	-1.2	-1.48	-1.2	-1.58	-1.36	-1.32	-1.15
ILMN_1365502	RGD1562836_predicted	-1.12	1.14	1.32	1.29	-1.25	-1.3	-1.15
ILMN_1359746	LOC499691	-1.02	-1	-1.51	-1.04	-1.24	-1.29	-1.15
ILMN_1358926	Cradd_predicted	-1.15	-1.15	-1.37	-1.21	-1.33	-1.28	-1.15
ILMN_1650462	RGD1305211_predicted	1.04	-1.36	-1.19	-1.26	-1.58	-1.27	-1.15
ILMN_1364659	Zfp574	-1.21	1.09	-1.13	1.03	-1.23	-1.27	-1.15
ILMN_1373862	Sephs2	-1.04	-1.02	1.27	-1.12	-1.18	-1.27	-1.15
ILMN_1361280	Pias3	-1.3	-1.27	-1.24	-1.28	-1.3	-1.25	-1.15
ILMN_1352931	Vps4a	-1.21	-1.3	-1.33	-1.09	-1.19	-1.24	-1.15
ILMN_1360155	LOC499677	-1.06	-1.06	-1.27	-1.14	-1.2	-1.21	-1.15
ILMN_1351158	Rasa1	-1.1	-1.01	-1.14	-1.13	-1.08	-1.21	-1.15
ILMN_1367614	Egln2	-1.18	-1.06	-1.18	-1.14	-1.34	-1.2	-1.15
ILMN_1373005	Mbip_predicted	-1.16	1.02	-1.01	-1.02	-1.16	-1.2	-1.15
ILMN_1352094	Wdr21_predicted	1.1	1.04	1.31	1.05	-1.42	-1.19	-1.15
ILMN_1354646	Tmem98	-1.05	-1.33	-1.22	-1.35	-1.1	-1.19	-1.15
ILMN_1357588	Bat2	1.02	1.07	-1.47	-1.08	1.16	-1.19	-1.15
ILMN_1363667	LOC474154	-1.03	-1.19	-1.4	-1.52	-1.2	-1.18	-1.15
ILMN_1361751	LOC293723	-1.09	1.25	1.23	1.33	-1.23	-1.17	-1.15
ILMN_1369597	Polh_predicted	-1.15	-1.23	-1.21	-1.15	-1.14	-1.17	-1.15
ILMN_1373311	Arrdc1	-1.16	-1.21	1.07	-1.02	-1.13	-1.17	-1.15
ILMN_1358564	Pabpc1	-1.38	-1.08	1.04	-1	-1.05	-1.17	-1.15
ILMN_1363528	RGD1564946_predicted	-1.12	-1.04	-1.07	-1.2	-1.46	-1.13	-1.15
ILMN_1650845	Shoc2	1.06	-1.15	-1.04	-1.21	-1.23	-1.12	-1.15
ILMN_1353220	Stoml2	-1.06	-1.06	1.11	1.03	-1.28	-1.11	-1.15
ILMN_1359392	LOC606294	-1.03	-1.23	-1.27	-1.01	-1.1	-1.11	-1.15
ILMN_1354380	Nsd1_predicted	-1.02	1.19	-1.12	1.08	-1.16	-1.09	-1.15
ILMN_1373008	RGD1560938_predicted	1.3	1.19	1.19	1.18	-1.13	-1.09	-1.15
ILMN_1374149	Surf1	-1.07	1.56	1.19	1.35	1.17	-1.09	-1.15
ILMN_1370807	Mtmr6_predicted	1.14	-1.18	-1.08	-1.23	-1.1	-1.08	-1.15
ILMN_1369058	RGD1311269_predicted	-1.03	-1.21	-1.1	1.02	-1.05	-1.08	-1.15
ILMN_1363320	Numa1	-1.14	-1.13	-1.3	-1.25	-1.21	-1.07	-1.15
ILMN_1373691	RGD1563276_predicted	-1.02	1.22	-1.03	-1	1.03	-1.07	-1.15
ILMN_1353336	Rbm3	-1.17	1.03	-1.01	1.25	1.04	-1.07	-1.15
ILMN_1360379	RGD1565137_predicted	1.09	-1.44	-1.21	-1.22	-1.09	-1.04	-1.15
ILMN_1369647	LOC499295	1.23	-1.3	-1.08	-1.27	1.05	-1.03	-1.15
ILMN_1366344	Dhx16	-1.06	1.02	-1.2	1.16	-1.18	-1.01	-1.15
ILMN_1364101	Qser1_predicted	1.18	1.2	-1.11	1.02	1.04	-1.01	-1.15
ILMN_1356752	RGD1560606_predicted	-1.07	-1.15	-1	-1.03	-1.19	-1	-1.15

ILMN_1374876	RGD1563620_predicted	-1.12	-1.42	-1.08	-1.34	-1.06	1.01	-1.15
ILMN_1349681	Rps2	1.01	-1.08	1.39	-1.02	-1.01	1.01	-1.15
ILMN_2038873	LOC498552	1	-1.08	-1.18	1.24	1.04	1.02	-1.15
ILMN_1352343	Angel2_predicted	1	-1.09	1.28	-1.03	-1.07	1.05	-1.15
ILMN_1361792	Osbp_predicted	-1.09	-1.24	-1.29	-1.34	1.07	1.05	-1.15
ILMN_1374056	LOC365954	1.05	-1.63	-1.41	-1.19	1.08	1.06	-1.15
ILMN_1367137	Tmem23	1.19	1.02	-1.1	-1.03	-1.09	1.07	-1.15
ILMN_1374147	Abcf2_predicted	1.2	1.13	1.41	1.32	-1.07	1.07	-1.15
ILMN_1362197	Asb3_predicted	1.08	-1.11	-1.1	-1.07	1.3	1.07	-1.15
ILMN_1371075	Nek9_predicted	1.07	-1.14	-1.26	-1.18	-1.07	1.1	-1.15
ILMN_1371863	Csnk1d	1.08	1.19	1.42	1.16	1.14	1.17	-1.15
ILMN_1370494	Sep-07	1.01	-1.20	1.01	-1.16	1.05	1.01	-1.15
ILMN_1364113	Ctgf	1	-3.62	-3.2	-2.66	-3.03	-2.73	-1.14
ILMN_1376671	Crry	-1.08	-1.54	-1.47	-1.83	-1.61	-1.52	-1.14
ILMN_1374396	Chchd1_predicted	-1	-1.15	-1.95	-1.12	-1.37	-1.48	-1.14
ILMN_1372790	LOC498353	-1.07	-1.35	-1.66	-2	-1.49	-1.4	-1.14
ILMN_1350709	Ndor1_predicted	-1.19	-1.2	-1.27	-1.24	-1.34	-1.4	-1.14
ILMN_1370590	LOC312667	-1.24	1.01	1.08	1.04	-1.55	-1.38	-1.14
ILMN_1376907	LOC682174	-1.15	-1.02	1.31	1.11	-1.18	-1.33	-1.14
ILMN_1363491	RGD1308992_predicted	-1.11	-1.03	-1.18	-1.24	1.04	-1.29	-1.14
ILMN_1363312	Prkcbp1	-1.01	-1.18	-1.46	-1.55	-1.26	-1.28	-1.14
ILMN_1372369	RGD1309387	1.11	1.12	1.2	1.15	-1.32	-1.24	-1.14
ILMN_1376280	Mknk2	-1.26	-1.17	-1.21	-1.15	-1.22	-1.23	-1.14
ILMN_1365219	RGD1562875_predicted	-1.11	-1.02	1.04	-1.22	-1.21	-1.22	-1.14
ILMN_1353238	LOC365555	-1.1	-1.2	-1.07	-1.75	-1.4	-1.21	-1.14
ILMN_1369379	Cox8a	1.16	-1.08	-2.09	-1.36	-1.12	-1.21	-1.14
ILMN_1366008	Tbl3	-1.1	1.08	-1.01	1.09	-1.14	-1.2	-1.14
ILMN_1358196	Bccip_predicted	-1.09	-1.15	1.06	-1.01	-1.05	-1.2	-1.14
ILMN_1363671	Bccip_predicted	-1.09	-1.15	1.06	-1.01	-1.05	-1.2	-1.14
ILMN_1366737	Ap4b1_predicted	1.1	-1.42	-1.32	-1.86	-1.28	-1.19	-1.14
ILMN_1365303	RGD1560538_predicted	1.13	1.04	-1.1	1.08	-1.18	-1.19	-1.14
ILMN_1373545	Map3k4_predicted	-1.02	1.08	-1.15	1.17	-1.22	-1.18	-1.14
ILMN_1362371	Gprk2l	-1.19	-1.18	-1.32	-1.31	-1.14	-1.18	-1.14
ILMN_1373991	Dhx35_predicted	-1.04	-1.09	-1.24	-1.11	-1.23	-1.15	-1.14
ILMN_1355271	Fbxo22	1.04	-1.04	1.02	1.04	-1.14	-1.15	-1.14
ILMN_1372078	LOC498289	-1.01	1.07	-1.01	1.16	1.04	-1.15	-1.14
ILMN_1373493	RGD735140	-1.02	-1.11	1.05	-1.18	-1.27	-1.14	-1.14
ILMN_1364751	RGD1307434_predicted	1.06	-1.08	1.19	-1.15	1.01	-1.14	-1.14

ILMN_1356783	Crsp3	-1.02	-1.32	-1.57	-1.41	-1.18	-1.13	-1.14
ILMN_1360557	RGD1306599_predicted	1.01	-1.16	-1.37	-1.2	-1.3	-1.12	-1.14
ILMN_1357889	Prkd2	-1.17	-1.2	-1.28	-1.24	-1.1	-1.12	-1.14
ILMN_1355122	RGD1559896_predicted	-1.15	-1.42	-1.3	-1.21	-1.02	-1.12	-1.14
ILMN_1353002	Mrpl48_predicted	-1.21	-1.09	-1.2	-1.09	-1.06	-1.11	-1.14
ILMN_1368544	RT1-N2	-1.04	-1.03	-1.05	-1.36	-1.3	-1.08	-1.14
ILMN_1357920	Usp3	1.15	1.35	1.08	1.19	-1.18	-1.08	-1.14
ILMN_1363053	Usp3	1.15	1.35	1.08	1.19	-1.18	-1.08	-1.14
ILMN_1352972	Btrc	1.12	-1.03	-1.21	1.01	-1.09	-1.08	-1.14
ILMN_1349824	Pik4cb	-1.21	-1.14	-1.16	-1.19	-1.08	-1.07	-1.14
ILMN_1349342	Gars	-1.04	1.04	1.13	-1.04	-1.02	-1.07	-1.14
ILMN_1357847	Peo1_predicted	1.11	1.08	1.06	-1.08	1.03	-1.06	-1.14
ILMN_1363097	RGD1311188_predicted	1.01	1.07	1.13	-1.03	1.09	-1.06	-1.14
ILMN_1361346	RGD1564940_predicted	-1.07	-1.29	-1.22	1.1	-1.23	-1.05	-1.14
ILMN_1651108	RGD1563322_predicted	1.03	-1.01	-1.29	1.03	-1.14	-1.05	-1.14
ILMN_1357010	RGD1564778_predicted	-1.18	-1.07	-1.16	1.04	-1.09	-1.05	-1.14
ILMN_1365485	Pdgfa	-1.29	1.05	-1.08	1.27	1.04	-1.05	-1.14
ILMN_1649739	Pycs_predicted	1.25	1.2	1.24	1.4	1.04	-1.04	-1.14
ILMN_1350437	Snx4_predicted	-1.1	-1.19	-1.05	-1.1	-1.12	-1.03	-1.14
ILMN_1650692	Gosr1	1.12	-1.08	1.07	-1.1	1.01	-1.03	-1.14
ILMN_1369711	Agpat6	1.06	-1.06	-1.03	1.07	1.16	-1.01	-1.14
ILMN_1350792	LOC314964	-1.08	1.14	1.33	1.18	1.17	-1.01	-1.14
ILMN_1367531	Ndufb8_predicted	-1.03	-1.32	-1.24	-1.14	-1.08	1.01	-1.14
ILMN_1356961	Azi2	-1	-1.08	-1.42	-1.3	-1.07	1.01	-1.14
ILMN_1370275	Lzic	1.08	1.04	1	-1.21	1.09	1.01	-1.14
ILMN_1368988	Csnk1g2	1.01	-1.17	1.13	-1.26	-1.16	1.02	-1.14
ILMN_1357731	Lin7c	-1.04	1.09	1.13	1.17	1.03	1.04	-1.14
ILMN_1349647	Dom3z	-1.11	-1.02	-1.18	1.15	1.1	1.06	-1.14
ILMN_1367013	Camlg	1.06	-1.11	-1.11	1.07	1.02	1.07	-1.14
ILMN_1354332	Ptpn2	-1.02	1.1	1.16	1.15	1.18	1.08	-1.14
ILMN_1351513	Asf1a_predicted	1.21	-1.02	-1.03	1.05	-1.04	1.1	-1.14
ILMN_1365088	Etv4_predicted	-1.09	1.25	1.51	1.17	-1.05	1.11	-1.14
ILMN_1356452	RGD1309059_predicted	1.17	1.04	-1.07	1.03	1.06	1.12	-1.14
ILMN_1376427	Tmem24	-1.06	1.32	1.06	1.15	-1.05	1.14	-1.14
ILMN_1368460	LOC497706	-1.14	-1.25	-1.57	-1.44	-1.07	1.18	-1.14
ILMN_1371120	LOC501637	1.03	1	-1.04	1.04	1.31	1.2	-1.14
ILMN_1363227	LOC499554	1.26	1.04	1.89	1.02	1.3	1.21	-1.14
ILMN_1362522	Dcps	-1.1	-1.47	-1.38	-1.48	-1.44	-1.67	-1.13

ILMN_1370958	Prtfdc1_predicted	-1.11	1.05	1.04	-1.06	-1.2	-1.5	-1.13
ILMN_1355649	Tnpo1	1.09	1.05	-1.31	1.07	-1.47	-1.45	-1.13
ILMN_1370450	Decr1	1.09	-1.05	-1.42	-1.8	-1.35	-1.42	-1.13
ILMN_1349155	Csnk2a2_predicted	1.18	1.03	-1.24	1.24	-1.33	-1.4	-1.13
ILMN_1371257	RGD1304924_predicted	-1.17	-1.25	-1.16	-1.28	-1.48	-1.39	-1.13
ILMN_1363330	mrpl11	-1	-1.35	-1.55	-1.34	-1.19	-1.39	-1.13
ILMN_1364067	MGC93975	-1.08	-1.4	-1.18	-1.38	-1.23	-1.38	-1.13
ILMN_1374057	LOC501075	1.26	-1.49	-1.94	-1.12	-1.41	-1.34	-1.13
ILMN_1354123	Mtif2	-1.08	-1.04	-1.22	-1.28	-1.37	-1.3	-1.13
ILMN_1353408	RGD1309562_predicted	-1.1	-1.57	-1.48	-1.74	-1.32	-1.3	-1.13
ILMN_1359476	Nbeal2_predicted	-1.08	1.01	-1.12	-1.03	-1.1	-1.3	-1.13
ILMN_1357235	LOC365436	-1.16	-1.26	1.09	-1.06	-1.24	-1.28	-1.13
ILMN_1370124	Park7	1.01	-1.44	-1.44	-1.72	-1.22	-1.28	-1.13
ILMN_1350225	Vil1_predicted	-1.14	1.4	1.07	1.77	-1.06	-1.27	-1.13
ILMN_1651180	LOC498411	1.03	-1.01	-1.05	1.03	-1.28	-1.25	-1.13
ILMN_1359676	Zdhhc12	-1.25	-1.56	-1.15	1.08	-1.24	-1.25	-1.13
ILMN_1360179	Spsb2	1	1.28	1.12	1.06	-1.2	-1.25	-1.13
ILMN_1356962	RGD1306746_predicted	-1.11	-1.28	-1.23	-1.45	-1.4	-1.23	-1.13
ILMN_1362899	Statip1	1.03	1.03	-1.07	1.01	-1.27	-1.23	-1.13
ILMN_1353828	Ppwd1_predicted	-1	-1.14	-1.23	-1.09	-1.15	-1.23	-1.13
ILMN_1350291	Tdp1	-1.28	-1.1	-1.02	-1.29	-1.22	-1.22	-1.13
ILMN_1349059	Prdx3	-1.15	1.09	1.18	1.02	-1.14	-1.22	-1.13
ILMN_1349569	Senp2	-1.01	-1.26	1.12	-1.38	1.06	-1.22	-1.13
ILMN_1366604	Rpl15	-1.02	-1.03	-1.01	-1.4	1.03	-1.2	-1.13
ILMN_1363183	Socs7_predicted	1.11	-1.03	-1.26	-1.01	-1.21	-1.19	-1.13
ILMN_1354925	LOC498418	-1.02	-1.27	-1.09	-1.37	-1.36	-1.17	-1.13
ILMN_1350244	RGD1566054_predicted	-1.12	-1.4	-1.34	-1.38	-1.25	-1.16	-1.13
ILMN_1358897	Glud1	-1	-1.02	-1.32	-1.26	-1.07	-1.16	-1.13
ILMN_1371721	RGD1309519_predicted	1.04	-1.1	1.12	-1.36	-1.28	-1.15	-1.13
ILMN_1373792	Rpl10a	1.07	-1.11	-1.19	-1.1	-1.11	-1.14	-1.13
ILMN_1360894	RGD1308637	-1.21	-1.36	-1.74	-1.9	-1.29	-1.13	-1.13
ILMN_1369744	LOC500669	-1.06	1	1.1	1	-1.14	-1.12	-1.13
ILMN_1374985	Inpp5b	1.04	1.26	-1.07	1.05	-1.1	-1.1	-1.13
ILMN_1349208	Ccdc43	-1.12	-1.26	-1.06	-1.23	-1.07	-1.1	-1.13
ILMN_1363968	Zfp148	-1.06	1.15	1.09	1.32	-1.02	-1.1	-1.13
ILMN_1356809	Xrn1_predicted	-1.05	1.01	-1.02	1.11	-1.27	-1.09	-1.13
ILMN_1354990	Spats2_predicted	1.01	1.22	-1.01	1.12	-1.18	-1.09	-1.13
ILMN_1363304	Spats2_predicted	1.01	1.22	-1.01	1.12	-1.18	-1.09	-1.13

ILMN_1364742	Fchsd2_predicted	-1.13	1.18	-1.09	1.13	-1.04	-1.08	-1.13
ILMN_1363916	Ube2f	-1.09	-1.18	1.16	-1.05	1	-1.08	-1.13
ILMN_1349559	Klf7_predicted	1.13	1.06	-1.07	-1.53	1.02	-1.08	-1.13
ILMN_1362163	Btf3	-1.12	1.02	1.01	-1.01	-1.12	-1.07	-1.13
ILMN_1366496	Slc25a32_predicted	1.06	1.12	1.49	1.42	-1.15	-1.06	-1.13
ILMN_1350897	Pfc_mapped	-1.05	-1.08	-1.32	-1.21	-1.16	-1.05	-1.13
ILMN_1364120	LOC361767	1.02	1.32	1.36	1.03	-1.14	-1.05	-1.13
ILMN_1372513	Tob1	-1.29	-1.17	-1.26	-1.37	-1.21	-1.04	-1.13
ILMN_1355536	Pias1_predicted	-1.25	-1.09	-1.17	-1.09	-1.15	-1.04	-1.13
ILMN_1368797	Klf15	1.11	1.29	1.12	1.42	1.02	-1.04	-1.13
ILMN_1354489	RGD1564433_predicted	1.08	-1.27	1.04	1.01	-1.13	-1.02	-1.13
ILMN_1349017	LOC360627	1.12	1.08	1.19	1.12	-1.04	-1	-1.13
ILMN_1374637	Ikbkap	1.03	1.13	-1.02	1.18	1.09	1.01	-1.13
ILMN_1350355	LOC360760	1.06	1.1	-1.13	-1.07	-1.17	1.02	-1.13
ILMN_1364187	Tmem111	-1.05	-1.22	1.04	-1.18	1.01	1.02	-1.13
ILMN_1376474	Strap	1.05	1.25	1.34	1.19	1.11	1.14	-1.13
ILMN_1362097	Mon2	-1.36	-1.06	-1	-1.07	1	1.15	-1.13
ILMN_1362073	RGD1305514_predicted	-1.14	1.34	1.21	1.46	1.27	1.15	-1.13
ILMN_1356401	Arpc4_predicted	-1.03	-1.05	1.09	1.15	1.06	1.16	-1.13
ILMN_1350636	LOC500067	1.02	-1.08	-1.22	-1.38	1.05	1.23	-1.13
ILMN_1369503	RGD1306346_predicted	1.52	1.11	1.3	1.05	1.23	1.39	-1.13
ILMN_1365120	Spink4	-1.27	1.23	-1.96	-1.35	-1.1	-2.25	-1.12
ILMN_1374546	Krt2-8	-1.07	-1.59	-1.47	-1.28	-1.36	-1.52	-1.12
ILMN_1355922	Dhps	1.01	-1.33	-1.18	-1.43	-1.5	-1.46	-1.12
ILMN_1355292	Rnf31_predicted	-1.04	1.38	-1.24	1.06	-1.49	-1.45	-1.12
ILMN_1369598	Dusp19_predicted	-1.05	-1.09	-1.22	-1.2	-1.46	-1.44	-1.12
ILMN_1374515	Csrp2bp_predicted	-1.07	-1.1	1.05	-1.1	-1.14	-1.41	-1.12
ILMN_1371835	RGD1562232_predicted	1.01	-1.43	-1.06	-1.35	-1.47	-1.38	-1.12
ILMN_1356277	Bckdhb	-1.18	-1.02	1.33	-1.04	1.01	-1.36	-1.12
ILMN_1369010	Pros1	-1.01	-1.15	-1.21	-1.47	-1.19	-1.34	-1.12
ILMN_1371815	Agpat2_predicted	-1.05	-1.03	1.05	-1.04	-1.26	-1.31	-1.12
ILMN_1376447	Isgf3g	-1.07	-1.19	-1.28	-1.14	-1.16	-1.31	-1.12
ILMN_1361223	Aes	-1.06	-1.78	-1.82	-1.57	-1.19	-1.3	-1.12
ILMN_1358480	LOC365566	-1.02	-1.21	-1.24	1.04	-1.27	-1.29	-1.12
ILMN_1371996	Mrpl21_predicted	-1.14	-1.3	-1.6	-1.51	-1.36	-1.27	-1.12
ILMN_1365334	Ywhaq	-1.17	-1.46	-1.23	-1.46	-1.11	-1.27	-1.12
ILMN_1352759	RGD1560373_predicted	-1.14	-1.75	-1.58	-1.85	-1.38	-1.26	-1.12
ILMN_1376345	Zipro1	-1	-1.02	-1.06	-1.09	-1.29	-1.26	-1.12

ILMN_1367710	Fibp	-1.18	-1.02	1.02	-1.26	-1.17	-1.26	-1.12
ILMN_1351167	RGD1311433_predicted	-1.23	1.06	-1.01	-1.04	-1.19	-1.25	-1.12
ILMN_1355835	Lamc1	-1.1	-1.29	-1.51	-1.44	-1.48	-1.23	-1.12
ILMN_1370141	Mfn1	1.06	-1.05	1.09	-1.01	-1.13	-1.23	-1.12
ILMN_1356137	Vdac2	1.02	1.04	-1.04	-1.28	-1.16	-1.22	-1.12
ILMN_1371277	Pigq	-1.13	-1.16	-1.15	-1.1	-1.23	-1.21	-1.12
ILMN_1359617	Ankrd49_predicted	1	-1.17	1.13	1.02	-1.22	-1.21	-1.12
ILMN_1365576	Mxd4_predicted	-1.06	-1.1	-1.31	-1.44	-1.19	-1.21	-1.12
ILMN_1366743	Hes6	-1.18	-1.03	1.1	-1.05	-1.04	-1.21	-1.12
ILMN_1350874	Aof1_predicted	-1.02	-1.08	-1.2	1.08	-1.26	-1.2	-1.12
ILMN_1353917	Spop	-1.03	-1.26	-1.56	-1.47	1.02	-1.2	-1.12
ILMN_1361015	LOC309475	-1.35	-2.1	-2.01	-2.2	-1.22	-1.19	-1.12
ILMN_1376483	Serpinc1	-1.03	1.19	1.07	-1.09	1.01	-1.19	-1.12
ILMN_1351065	LOC497834	-1.09	-1.57	-1.25	-1.21	-1.22	-1.18	-1.12
ILMN_1651082	RGD1565055_predicted	-1.09	1.2	-1.12	1.18	-1.44	-1.16	-1.12
ILMN_1359772	RGD1566386_predicted	1.12	-1.18	1.06	1.09	-1.05	-1.14	-1.12
ILMN_1368788	LOC686344	-1.03	-1.2	1.12	-1.07	1.01	-1.14	-1.12
ILMN_1357995	RGD1559841_predicted	1.1	-1.21	-1.13	1.02	-1.2	-1.12	-1.12
ILMN_1367607	RGD1559841_predicted	1.1	-1.21	-1.13	1.02	-1.2	-1.12	-1.12
ILMN_1359865	Gtl3	1	-1.17	-1.23	-1.14	-1.06	-1.11	-1.12
ILMN_1366225	Cnnm3_predicted	-1.15	-1.14	-1.36	1.06	-1.11	-1.09	-1.12
ILMN_1350644	RGD1565135_predicted	1.02	-1.12	-1.46	-1.21	-1.08	-1.08	-1.12
ILMN_1362787	Gs3	1.01	1.29	1.17	1.29	-1.15	-1.07	-1.12
ILMN_1350044	MGC94113	-1.1	1.11	1.11	1.07	-1.09	-1.07	-1.12
ILMN_1367918	Ankrd39_predicted	1.15	1.26	1.07	1.2	-1.04	-1.07	-1.12
ILMN_1365361	Epn2	1.06	1.28	1.09	1.14	1.08	-1.07	-1.12
ILMN_1349263	Ascc1	-1.09	-1.08	1.01	-1.26	-1.13	-1.03	-1.12
ILMN_1352433	RGD1305156	1.02	-1.21	-1.25	-1.2	1.01	-1.03	-1.12
ILMN_1364466	Stk25	-1.26	-1.11	-1.2	-1.24	-1.03	-1.02	-1.12
ILMN_1366666	Lman2_predicted	-1.12	-1.11	1.05	-1.13	1.06	-1.02	-1.12
ILMN_1349797	Sepw1	2	1.32	1.1	1.27	1.1	-1.02	-1.12
ILMN_1364586	RGD1308795_predicted	-1.03	1.09	-1.07	1.06	-1.13	-1.01	-1.12
ILMN_1355335	Pofut2_predicted	-1.06	-1.35	-1.13	-1.3	-1.07	-1.01	-1.12
ILMN_1357819	Stag2_predicted	1.22	1.09	1.01	-1.08	-1.24	1.02	-1.12
ILMN_1372536	Falz_predicted	-1	1.15	-1.03	-1.02	-1.21	1.02	-1.12
ILMN_1368644	Pik3cb	1.01	-1	-1	-1.28	-1.06	1.02	-1.12
ILMN_1373708	Nmt1	-1.11	-1.03	-1.2	-1.1	1.03	1.02	-1.12
ILMN_1374709	RGD1563839_predicted	-1.05	-1.05	-1.35	1.04	1.03	1.02	-1.12

ILMN_1351608	Traf4_predicted	-1.11	1.42	1.35	1.49	1.08	1.02	-1.12
ILMN_1352016	RGD1564875_predicted	-1	1.15	-1.03	1.12	-1.09	1.03	-1.12
ILMN_1372467	Rpsa	-1.04	1	1.04	-1.02	-1.04	1.03	-1.12
ILMN_1363615	Men1	-1.09	1.05	-1.15	1.08	1.06	1.03	-1.12
ILMN_1372621	Cmtm8	1.11	1.34	1.25	1.26	1.11	1.03	-1.12
ILMN_1358641	RGD1562135_predicted	1.18	-1	-1.01	1.1	-1.1	1.04	-1.12
ILMN_1367830	RGD1311265	-1.05	1.16	-1.06	-1.12	-1.13	1.05	-1.12
ILMN_1364836	Als2	-1.37	-1.08	-1.12	1.08	1.17	1.05	-1.12
ILMN_1361170	Jak2	-1.02	-1.25	-1	-1.05	1.14	1.07	-1.12
ILMN_1357783	Hbegf	1.16	1.43	1.36	1.47	-1.01	1.08	-1.12
ILMN_1352776	RGD1307636_predicted	1.06	1.07	1.09	-1.02	1.09	1.09	-1.12
ILMN_1651050	Mast2_predicted	-1.23	1.13	-1.07	1.19	1.13	1.1	-1.12
ILMN_1371010	Ube3a_predicted	-1.09	1.09	-1.03	1.26	1.04	1.11	-1.12
ILMN_1371495	LOC308775	1.03	1.06	1.15	-1.07	-1.02	1.14	-1.12
ILMN_1359231	St7l	-1.1	-1.04	-1.11	-1.26	1.04	1.14	-1.12
ILMN_1357270	LOC296582	-1.05	1	1.26	-1.1	1.06	1.14	-1.12
ILMN_1348807	RGD1307983_predicted	1.2	1.43	1.17	1.24	1.19	1.15	-1.12
ILMN_1362453	RGD1562231_predicted	1.06	-1.04	-1.08	-1.2	1.22	1.15	-1.12
ILMN_1369510	Pgam5	1.2	1.24	2.19	1.47	1.03	1.17	-1.12
ILMN_1360241	RGD1563824_predicted	-1.01	1.01	-1.05	1.17	1.13	1.19	-1.12
ILMN_1358674	Tnks2_predicted	1.2	1.16	1.1	-1.12	1.07	1.28	-1.12
ILMN_1354112	Fgb	1.03	-1.73	-2.43	-3.29	-2.33	-2.56	-1.11
ILMN_1362365	Scml4_predicted	-1.02	-1.03	-1.1	-1.09	-1.28	-1.48	-1.11
ILMN_1366070	Hadhsc	-1.21	-1.19	-1.3	-1.74	-1.3	-1.47	-1.11
ILMN_1367475	Aldh1a1	1.11	-1.83	-1.77	-2.12	-1.28	-1.46	-1.11
ILMN_1349101	As3mt	-1.47	-1.17	-1.03	-1.26	-1.43	-1.44	-1.11
ILMN_1371937	LOC501139	-1.21	-1.28	-1.78	-1.23	-1.2	-1.42	-1.11
ILMN_1373978	Pcolce	-1.17	1.04	1.06	-1.07	-1.12	-1.42	-1.11
ILMN_1355746	RGD1559617_predicted	1.08	-1.27	-1.98	-1.36	-1.5	-1.41	-1.11
ILMN_1363345	MGC94542	-1.1	-1.49	-1.14	-1.43	-1.3	-1.32	-1.11
ILMN_1372043	Nr5a2	1.1	-1.07	-1.2	-1.2	-1.13	-1.32	-1.11
ILMN_1352160	Mrps34_predicted	-1.17	1.23	1.09	1.3	-1.15	-1.3	-1.11
ILMN_1363417	RGD1309359_predicted	1.08	-1.09	-1.04	-1.02	-1.31	-1.29	-1.11
ILMN_1650091	RGD1305727_predicted	1.13	-1.06	1.21	1.2	-1.31	-1.29	-1.11
ILMN_1371986	Esd_mapped	-1.01	-1.2	-1.06	-1.23	-1.3	-1.28	-1.11
ILMN_1356454	Fgfrl1	-1.01	1.06	1.43	1.26	-1.22	-1.28	-1.11
ILMN_1351280	Dlst	1.07	-1.28	-1.2	-1.16	-1.12	-1.28	-1.11
ILMN_1369769	RGD1305007	1.03	-1.57	-1.22	-1.65	-1.38	-1.27	-1.11

ILMN_1356084	Gart	1.15	-1.03	-1.07	-1.02	-1.27	-1.27	-1.11
ILMN_1376301	Enpp3	1.31	1.11	1.09	-1.22	-1.21	-1.27	-1.11
ILMN_1363242	Crnkl1	-1.04	-1.45	-1.53	-1.54	-1.23	-1.25	-1.11
ILMN_1376352	Hip1	-1.02	1	-1.27	-1.14	-1.21	-1.25	-1.11
ILMN_1375389	Hip1	-1.02	1	-1.27	-1.14	-1.21	-1.25	-1.11
ILMN_1357450	Tnks1bp1_predicted	-1.03	-1.09	-1.35	-1.05	-1	-1.24	-1.11
ILMN_1354385	MGC114410	1.28	-1.15	1.26	1.06	-1.38	-1.21	-1.11
ILMN_1350831	LOC499357	1.03	-1.28	-1.05	-1.09	-1.18	-1.21	-1.11
ILMN_1367964	Gnai2	-1.13	-1.39	-1.19	-1.14	-1.14	-1.21	-1.11
ILMN_1367513	Slc7a6_predicted	1.11	-1.05	1.14	1.02	-1.34	-1.2	-1.11
ILMN_1364243	MGC94207	1.02	-1.03	-1.26	1.01	-1.16	-1.19	-1.11
ILMN_1368966	Mterf	1.02	-1.01	-1.03	-1.05	-1.16	-1.17	-1.11
ILMN_1362440	RGD1564209_predicted	-1.01	-1.07	1.12	-1.16	-1.22	-1.16	-1.11
ILMN_1370063	Mdh1	-1.15	-1.28	-1.17	-1.35	1.01	-1.15	-1.11
ILMN_1360443	RGD1306063_predicted	-1.32	-1.46	-1.45	-1.64	-1.15	-1.14	-1.11
ILMN_1350117	Fcgrt	-1.15	-1.09	1.29	-1.33	-1.14	-1.14	-1.11
ILMN_1359162	LOC305076	-1.04	1.15	1.07	1.16	-1.27	-1.11	-1.11
ILMN_1358985	Vdac1	-1.02	-1.07	1.13	-1.07	-1.17	-1.11	-1.11
ILMN_1355534	LOC498750	-1.15	1.01	1.02	-1.14	-1.12	-1.1	-1.11
ILMN_1369067	LOC288174	-1.2	1.05	-1.04	-1.42	-1.11	-1.1	-1.11
ILMN_1373960	Smu1	-1.06	-1.22	-1.04	-1.08	-1.08	-1.1	-1.11
ILMN_1355016	LOC498185	-1.09	1.01	-1	1.07	-1.09	-1.09	-1.11
ILMN_1364140	Cops5	-1.16	-1.18	-1.13	-1.21	-1.06	-1.09	-1.11
ILMN_1355656	Thrap3	-1.16	1.17	-1	-1.05	-1.06	-1.09	-1.11
ILMN_2040770	RGD1560826_predicted	-1.08	-1.85	-1.43	-1.08	-1.06	-1.06	-1.11
ILMN_1366658	LOC499120	1.04	-1.04	-1.05	-1.11	-1.02	-1.05	-1.11
ILMN_1352889	Tctex1	1	-1.06	-1.32	1.16	1.08	-1.05	-1.11
ILMN_1363168	Slc26a8_predicted	-1.09	1.01	1.09	-1.04	-1.36	-1.04	-1.11
ILMN_1362852	LOC679890	-1.06	-1.06	1.13	1.05	-1.13	-1.04	-1.11
ILMN_1356551	Ndrg2	-1.15	-1.47	-1.49	-1.79	-1.11	-1.04	-1.11
ILMN_1374574	RGD1307222_predicted	-1.17	-1.17	-1.15	1.01	-1.36	-1.03	-1.11
ILMN_2040065	LOC499331	1.06	-1.09	-1.01	-1.06	-1.27	-1.03	-1.11
ILMN_1373728	Traf2_predicted	1.05	1.2	1.1	1.27	1.17	-1.03	-1.11
ILMN_1650919	Terf2ip	-1.19	-1.26	-1.04	-1.28	-1.15	-1.02	-1.11
ILMN_1352413	Ing4	-1.06	-1.46	-1.38	-1.52	-1.21	-1.01	-1.11
ILMN_1364219	LOC497699	-1.03	1.23	-1.01	-1.01	-1.13	-1.01	-1.11
ILMN_1370452	Stap2	-1.26	-1.26	-1.16	-1.23	-1.08	-1.01	-1.11
ILMN_1376714	Zbtb1	-1.11	1.41	1	1.33	-1.03	-1.01	-1.11

ILMN_1368543	Mettl6	-1.22	-1.16	1.08	1.22	1.04	-1.01	-1.11
ILMN_1371540	Ndufa8	-1.12	-1.17	-1.28	-1.25	1.01	-1	-1.11
ILMN_1367829	Cept1	-1.12	-1.02	-1.1	-1.06	1.05	-1	-1.11
ILMN_1357918	Inppl1	1.01	1.41	-1.04	1.15	-1.11	1	-1.11
ILMN_1353790	Lypla2	-1.11	-1.06	-1.1	1.11	-1.05	1.01	-1.11
ILMN_1359102	Cops2	1.03	1.17	1.13	1.12	-1.04	1.01	-1.11
ILMN_1354605	Supt5h	-1.32	1.14	-1.16	-1.08	-1.02	1.01	-1.11
ILMN_1376981	Pcgf1	1.06	1.33	1.15	1.39	-1.14	1.02	-1.11
ILMN_1356036	Rps6kb1	-1.21	1.01	1.03	1.1	-1.13	1.02	-1.11
ILMN_1353982	Amfr_predicted	1.09	-1.29	1.37	-1.25	-1.1	1.02	-1.11
ILMN_1370864	Acot8	-1.19	-1.15	-1.11	-1.07	-1.04	1.02	-1.11
ILMN_1353898	Lrrc42	-1.02	1.16	1.02	1.21	1.09	1.02	-1.11
ILMN_1376384	Zfp64	-1.09	1.15	1.05	1.08	1.21	1.02	-1.11
ILMN_1650574	RGD1308513	-1.01	-1.09	1.16	-1.04	-1.13	1.03	-1.11
ILMN_2038943	RGD1306841	-1.09	-1.11	1.26	-1.05	-1.02	1.04	-1.11
ILMN_1360433	P4hb	-1.29	-1.34	1.03	-1.21	1.08	1.06	-1.11
ILMN_1369829	Prkwnk1	1.14	1.15	1.11	1.11	-1.25	1.11	-1.11
ILMN_1372402	Thrap1_predicted	-1.11	1.08	1.03	1.14	-1.09	1.11	-1.11
ILMN_1650489	RGD1309062	1.06	-1.1	1.08	-1.22	1.21	1.11	-1.11
ILMN_1356069	Clk1	-1.3	1.08	1.08	-1.02	-1	1.12	-1.11
ILMN_1362190	RGD1562028_predicted	1.12	1.08	1.15	-1.01	1.11	1.12	-1.11
ILMN_1354176	Srebf2_predicted	1.02	-1.09	1.16	-1.15	1.03	1.14	-1.11
ILMN_1356655	Il17r_predicted	-1.03	1.32	1.28	1.3	1.08	1.18	-1.11
ILMN_1355188	LOC499775	1.09	1.1	1.19	1.46	1.1	1.21	-1.11
ILMN_1357898	LOC367289	-1.07	1.09	1.22	1.19	1.28	1.21	-1.11
ILMN_1361620	LOC497884	1.01	1.02	-1.12	-1.21	1.05	1.25	-1.11
ILMN_1356369	Glrx1	-1.16	-1.91	-1.95	-2.6	-1.61	-1.83	-1.1
ILMN_1373175	RGD1562991_predicted	-1.15	-1.47	-1.07	-1.33	-1.49	-1.62	-1.1
ILMN_1359969	LOC498154	1.09	1.13	1.27	1.44	-1.32	-1.58	-1.1
ILMN_1349966	Cetn2	-1.19	-1.99	-1.85	-2.36	-1.47	-1.53	-1.1
ILMN_1364617	Mmaa_predicted	-1.05	-1.27	-1.1	-1.26	-1.54	-1.41	-1.1
ILMN_1368589	Txn2	-1.33	-1.55	-1.4	-1.5	-1.36	-1.39	-1.1
ILMN_1349288	RGD1561783_predicted	-1.16	-1.14	-1.28	-1.58	-1.42	-1.36	-1.1
ILMN_1372343	Leprotl1	1.04	-1.16	-1.43	-1.38	-1.41	-1.32	-1.1
ILMN_1360455	Tmem110	1.01	1.34	1.2	1.2	-1.28	-1.28	-1.1
ILMN_1365682	Pdk3_mapped	1.04	-1.35	-1.24	-1.32	-1.07	-1.28	-1.1
ILMN_1365251	LOC311254	-1.04	1.1	1.2	1.21	-1.21	-1.26	-1.1
ILMN_1370465	LOC501237	-1.04	-1.32	1.19	-1.16	-1.36	-1.25	-1.1

ILMN_1351337	Smyd2	1	-1.22	1.03	-1.15	-1.36	-1.25	-1.1
ILMN_1372484	Egln1	-1.24	-1.65	-1.55	-1.37	-1.2	-1.25	-1.1
ILMN_1375075	Atad3a	1.02	1.17	1.4	1.32	-1.2	-1.23	-1.1
ILMN_1357938	Tcfcp2_predicted	-1.01	-1.08	1.1	-1.18	-1.24	-1.21	-1.1
ILMN_1375140	Naca_predicted	-1.09	1.07	1.03	1.03	-1.07	-1.21	-1.1
ILMN_1367321	Pgap1	1.04	-1.02	-1.23	-1.25	-1.63	-1.19	-1.1
ILMN_1364915	LOC300760	-1.1	-1.68	-1.78	-1.81	-1.33	-1.19	-1.1
ILMN_1360143	Acin1	-1.07	1.14	-1.22	-1	-1.33	-1.16	-1.1
ILMN_1367049	Phyhd1	-1.25	1.09	1.06	-1.14	1.01	-1.16	-1.1
ILMN_1370100	RGD1304793_predicted	-1.2	1.04	1.02	-1.07	-1	-1.15	-1.1
ILMN_1365127	Noc4l	1.06	1.09	1.19	1.46	-1.16	-1.14	-1.1
ILMN_1352852	Araf	1.02	-1.12	-1.2	-1.17	-1.14	-1.13	-1.1
ILMN_1355604	Crat	-1.01	-1.08	-1.12	-1.1	-1.09	-1.13	-1.1
ILMN_1367485	LOC307798	1.04	-1.01	1.87	1.06	-1.03	-1.13	-1.1
ILMN_1371164	Pcyox1	1.03	-1.09	-1.05	-1.1	-1.06	-1.12	-1.1
ILMN_1371204	Uqcrc1	1.08	-1.04	-1	-1.01	-1.07	-1.11	-1.1
ILMN_1649834	RGD1560962_predicted	1.08	1.01	1.16	-1.16	-1.23	-1.1	-1.1
ILMN_1365126	RGD1561530_predicted	-1.03	1.39	1.48	1.3	-1.17	-1.1	-1.1
ILMN_1351395	RGD1306248	1.21	-1.05	1.25	-1.05	-1.27	-1.09	-1.1
ILMN_1365973	LOC498331	1.05	1.01	-1.01	1.02	-1.17	-1.09	-1.1
ILMN_1357839	RGD1559961_predicted	-1.04	1.07	1.02	1.28	-1.12	-1.08	-1.1
ILMN_1370360	RGD1559961_predicted	-1.04	1.07	1.02	1.28	-1.12	-1.08	-1.1
ILMN_1350429	Setdb2_predicted	-1.13	-1.03	-1.31	1.01	-1.09	-1.07	-1.1
ILMN_1369289	LOC497967	1.04	1.1	1.17	-1.02	-1.1	-1.04	-1.1
ILMN_1361530	Drap1_predicted	1.1	-1.13	-1.04	-1.3	-1.17	-1.03	-1.1
ILMN_1355392	RGD1304726	-1.26	-1.05	-1.24	-1.22	-1.16	-1.03	-1.1
ILMN_1350585	Wdr5	1.22	1.13	1.31	1.16	-1.19	-1.01	-1.1
ILMN_1376870	Hap1	1.19	1.14	1.13	1.14	-1	-1.01	-1.1
ILMN_1373773	LOC500054	-1.08	1.07	1.01	-1.04	-1.05	-1	-1.1
ILMN_1372034	Aqr_predicted	1.11	1.09	-1.14	1.09	1.02	1	-1.1
ILMN_1353216	Zfp131	1.08	1.46	1.56	1.73	1.02	1.01	-1.1
ILMN_1367124	Pprc1_predicted	1.05	1.63	1.08	1.47	1	1.02	-1.1
ILMN_1358122	Pigx	1.14	1.21	1.09	-1.11	1.05	1.02	-1.1
ILMN_1359894	MGC124888	1.09	1	-1.11	-1.07	-1.16	1.03	-1.1
ILMN_1350497	Wdr45	1.1	-1.35	-1.6	-1.84	-1.07	1.08	-1.1
ILMN_1359532	Slc4a1ap_predicted	-1.03	1.02	-1.06	1.03	-1.02	1.09	-1.1
ILMN_1373668	RGD1310553	-1.04	1.18	1.01	1.18	1.02	1.09	-1.1
ILMN_1359122	Stau1	-1.07	-1.01	1.01	-1.16	1.05	1.09	-1.1

ILMN_1351635	Sfrs2	-1.17	-1.42	1.03	-1.09	1.11	1.09	-1.1
ILMN_1353961	Prpf3_predicted	-1.15	-1.03	-1	1.18	1.07	1.1	-1.1
ILMN_1353577	Zcchc10	1.15	1.07	-1.01	-1.13	-1.08	1.11	-1.1
ILMN_1353075	Lrrc8d	1.16	-1.02	1.19	-1.2	1.11	1.11	-1.1
ILMN_1650565	Surf6_predicted	1.19	1.23	1.73	1.14	1.18	1.11	-1.1
ILMN_1372751	B4galt1_mapped	1.07	1.09	1.25	1.17	1.25	1.11	-1.1
ILMN_1354421	Phf12	-1.07	1.21	-1.19	1.07	-1.33	1.12	-1.1
ILMN_1372593	Eif3s10	-1.03	1.39	1.27	1.2	1.07	1.12	-1.1
ILMN_1362011	LOC684506	1.02	-1.13	1.1	1.06	1.22	1.13	-1.1
ILMN_1351873	Fnta	-1.17	1.16	1.08	1.2	1.19	1.14	-1.1
ILMN_1375522	Cspg5	1.05	-1	-1.22	-1.28	1.19	1.17	-1.1
ILMN_1356149	Lypla1	-1.03	-1.07	1.16	-1.17	1.22	1.19	-1.1
ILMN_1359028	RGD1559988_predicted	1.53	1.05	-1.04	-1.09	1.08	1.36	-1.1
ILMN_1357213	LOC499178	-1.12	1.24	1.09	1.18	1.15	1.41	-1.1
ILMN_1354686	Cpt2	1.02	-1.7	-1.63	-1.4	-1.52	-1.74	-1.09
ILMN_1368760	Pop5_predicted	1.21	-1.12	1.07	1.11	-1.33	-1.54	-1.09
ILMN_1354936	RGD1563202_predicted	-1.03	-1.5	-2.05	-1.73	-1.54	-1.48	-1.09
ILMN_1349348	Mipep	-1.05	1.02	-1.04	-1.08	-1.21	-1.48	-1.09
ILMN_1349253	Commd5	-1.07	-1.16	-1.28	1.03	-1.32	-1.43	-1.09
ILMN_1361660	Pqlc3	1.03	-1.2	-1.24	-1.37	-1.39	-1.42	-1.09
ILMN_1354595	Bcs1l	1.11	-1.02	1.21	1.37	-1.38	-1.4	-1.09
ILMN_1650351	Slc9a6_predicted	-1.23	-1.28	-1.59	-1.38	-1.37	-1.4	-1.09
ILMN_1366902	RGD1564947_predicted	-1.01	1.09	-1.02	-1.02	-1.2	-1.38	-1.09
ILMN_1367315	RGD1563920_predicted	-1.06	-1.28	-1.34	-1.24	-1.03	-1.36	-1.09
ILMN_1360602	Smpd3	1.05	-1.14	-1.43	-1.42	-1.17	-1.31	-1.09
ILMN_1353846	Polr3e_predicted	1.05	1.17	1.24	1.25	-1.41	-1.3	-1.09
ILMN_1368818	LOC304971	-1.13	-1.15	-1.32	-1.19	-1.31	-1.3	-1.09
ILMN_1359655	LOC683919	1.09	1.51	1.25	1.33	-1.12	-1.3	-1.09
ILMN_1349416	RGD1311612_predicted	-1.11	-1.02	-1.13	1.06	-1.18	-1.29	-1.09
ILMN_1368709	Trmu_predicted	1.03	-1.27	1.07	1.41	-1.3	-1.28	-1.09
ILMN_1649985	Rfk	1.26	-1.35	-1.35	-1.28	-1.25	-1.25	-1.09
ILMN_1356085	LOC288455	1.07	-1.7	-2.04	-1.43	-1.24	-1.25	-1.09
ILMN_1351688	RGD1311732	-1.21	-1.31	-1.29	-1.37	-1.17	-1.24	-1.09
ILMN_1367417	Polrmt_predicted	-1.02	1.58	-1	1.54	-1.25	-1.23	-1.09
ILMN_1368724	C3orf6h	-1.04	1.1	-1.03	-1.02	-1.18	-1.22	-1.09
ILMN_1356943	LOC298842	1.12	-1	1.27	-1.07	1.12	-1.22	-1.09
ILMN_1350410	Uqcrb_predicted	-1.01	-1.18	-2.1	-1.26	-1.09	-1.21	-1.09
ILMN_1362404	Peflin	-1.14	-1.19	-1.22	-1.56	-1.25	-1.2	-1.09

ILMN_1362086	RGD1304704	-1.15	-1.16	-1.17	-1.2	-1.23	-1.2	-1.09
ILMN_1360202	LOC500378	1.09	-1.13	1.38	-1.07	-1.13	-1.16	-1.09
ILMN_1364350	Dedd	-1.01	-1.18	1.29	-1.02	1.02	-1.16	-1.09
ILMN_1363982	Pdk1	1.05	1.27	1.28	1.19	1.14	-1.16	-1.09
ILMN_1349529	Rabggta	-1.03	-1.39	-1.26	-1.36	-1.26	-1.14	-1.09
ILMN_1650112	RGD1311547	1.05	1.07	1.18	1.04	1	-1.14	-1.09
ILMN_1350059	Prpf31_predicted	-1.01	-1.2	-1.33	-1.14	-1.2	-1.13	-1.09
ILMN_1371734	Tada1l	-1.08	-1.15	-1.19	-1.22	-1.12	-1.13	-1.09
ILMN_1369812	Zfp36l2	-1.13	1.09	1.13	-1.06	-1.3	-1.12	-1.09
ILMN_1364762	RGD1565023_predicted	-1.12	-1.21	1.03	-1.09	-1.28	-1.12	-1.09
ILMN_1362459	Sfrs12	1.03	1.03	-1.09	-1	-1.25	-1.11	-1.09
ILMN_1358859	Zmpste24_predicted	1.1	-1.32	1.21	-1.31	-1.1	-1.11	-1.09
ILMN_1351455	Rpl3	1.02	-1.02	1.04	-1.03	-1.07	-1.11	-1.09
ILMN_1371286	Jundp2	1.1	1.08	1.25	1.42	1.04	-1.09	-1.09
ILMN_1364233	Chfr	-1.19	-1.2	-1.1	-1.1	-1.13	-1.08	-1.09
ILMN_1349286	Pcoln3_predicted	-1.01	-1.12	1.21	-1.03	-1.1	-1.08	-1.09
ILMN_1650708	LOC501386	-1.08	-1.3	1.25	-1.14	-1.3	-1.07	-1.09
ILMN_1650398	RGD1564887_predicted	-1.14	-1.25	-1.11	-1.08	-1.28	-1.07	-1.09
ILMN_2039632	LOC685778	1.05	-1.1	-1.06	-1.24	-1.11	-1.07	-1.09
ILMN_1352402	RGD1562326_predicted	1.11	-1.13	-1.12	-1.15	-1.03	-1.07	-1.09
ILMN_1371310	RGD1305089_predicted	1.11	-1.21	-1.2	-1.15	-1.16	-1.06	-1.09
ILMN_1355787	Eif2b1	-1.21	-1.32	-1.06	-1.22	-1.03	-1.06	-1.09
ILMN_1357649	G6pc3	1.02	-1.09	1.44	-1.11	1.04	-1.05	-1.09
ILMN_1353821	Fzd7_predicted	1.08	-1.02	1.24	1.13	-1.1	-1.04	-1.09
ILMN_1369155	RGD1311703	-1.14	-1.19	-1.11	-1.22	-1.02	-1.04	-1.09
ILMN_1354897	Jak1	-1.02	-1.05	-1.09	-1.05	1.01	-1.03	-1.09
ILMN_1374988	Pdrp	-1.18	1.03	-1.14	-1.12	1.12	-1.03	-1.09
ILMN_1355409	LOC288526	-1.09	-1.01	-1.32	-1.13	1.02	-1.02	-1.09
ILMN_1350234	Fbxw4_predicted	1.05	-1.01	1.34	-1.14	1.03	-1.02	-1.09
ILMN_1356600	RGD1308087	1.03	-1.21	-1.01	1.03	-1.09	-1.01	-1.09
ILMN_1373885	Usp24_predicted	1.09	1.12	-1.08	1.07	-1.04	1	-1.09
ILMN_1650169	Znf498_predicted	-1.02	-1.04	-1.42	-1.28	-1.16	1.01	-1.09
ILMN_1359727	Ctnnd1_predicted	-1.08	-1.06	-1.63	-1.38	-1.01	1.01	-1.09
ILMN_1359081	Nob1p	1.07	1.43	1.37	1.31	1.03	1.02	-1.09
ILMN_1359798	Nck2_predicted	1.1	1.34	-1.1	1.29	1.13	1.02	-1.09
ILMN_1370636	Rab4a	-1.04	1.03	1.22	1.1	1.01	1.03	-1.09
ILMN_1349571	Mtfr1_predicted	1.25	-1.12	1.29	-1.04	1.07	1.03	-1.09
ILMN_1376954	Uchl5	1.12	-1.44	-1.05	1	1.06	1.04	-1.09

ILMN_1372206	Pak2	-1.1	1.07	1.04	1.13	-1.04	1.05	-1.09
ILMN_1364407	Rbm16	1.02	1.1	-1.12	-1.1	1	1.05	-1.09
ILMN_1363813	Celsr3	-1.24	1.26	-1.08	1.47	1.1	1.05	-1.09
ILMN_1376390	ST7	1.17	-1	1.07	-1.05	-1.06	1.07	-1.09
ILMN_1376315	Fbxo8	1.09	-1.23	-1.36	-1.71	1.09	1.08	-1.09
ILMN_1356035	Dgcr14	-1.08	-1.03	-1.12	-1.44	-1.26	1.11	-1.09
ILMN_1374732	LOC310615	-1.22	1.14	1.21	1.04	1.04	1.11	-1.09
ILMN_2038931	LOC366968	1.01	1.05	-1.08	-1.18	1.12	1.13	-1.09
ILMN_1370335	Btbd3_predicted	1.22	1.57	1.18	1.39	1.1	1.15	-1.09
ILMN_1349524	Mgea6_predicted	1.07	1.38	1.1	-1.06	1.12	1.15	-1.09
ILMN_1372256	Elf1	1.04	-1.05	1.03	-1.17	1.06	1.17	-1.09
ILMN_1365360	Pom121	-1.24	-1.03	-1.21	-1.01	1.19	1.18	-1.09
ILMN_1364290	Supt4h2_predicted	-1.05	1.01	-1.2	-1.22	1.09	1.21	-1.09
ILMN_1352070	Fntb	1.05	-1.04	-1.33	-1.22	1.55	1.23	-1.09
ILMN_1366200	RGD1562983_predicted	-1.05	-1.26	-1.22	-1.1	1.12	1.26	-1.09
ILMN_1356461	LOC246187	-1	1.14	1.03	-1.03	1.24	1.32	-1.09
ILMN_1349429	Pitpnb	1.25	1.18	1.29	1.36	1.37	1.32	-1.09
ILMN_1361919	Alad	-1.3	-1.03	-1.13	-1.03	-1.43	-1.66	-1.08
ILMN_1361374	RGD1304982_predicted	-1.1	1.14	1.05	-1.34	-1.48	-1.48	-1.08
ILMN_1651137	Bloc1s1_predicted	1.01	-1.1	-2.12	-1.35	-1.43	-1.41	-1.08
ILMN_1351410	Uxt	-1.14	-1.07	-1.45	-1.18	-1.4	-1.41	-1.08
ILMN_1354917	Slc22a18	-1.05	1.06	1.06	-1.68	-1.31	-1.39	-1.08
ILMN_1363191	Chrd	-1.32	-1.06	-1.24	-1.26	-1.45	-1.33	-1.08
ILMN_1365240	RGD1563990_predicted	-1.12	-1.19	-1.1	-1.22	-1.33	-1.33	-1.08
ILMN_1351775	Dscr2_predicted	-1.11	-1.38	-1.12	-1.44	-1.24	-1.32	-1.08
ILMN_1370087	LOC498062	1.12	-1.03	-1.76	-1.03	-1.14	-1.32	-1.08
ILMN_1366329	Tor2a	-1.07	-1.28	-1.33	-1.24	-1.24	-1.3	-1.08
ILMN_1354571	Psmd9	-1	-1.12	-1.16	-1.15	-1.29	-1.29	-1.08
ILMN_1650242	RGD1309256	-1.03	-1.06	-1.39	-1.07	-1.23	-1.29	-1.08
ILMN_1372939	Ndufb2_predicted	1.09	-1.26	-2.33	-1.28	-1.17	-1.29	-1.08
ILMN_1349061	Pigl	1.09	-1.17	-1.13	-1.22	-1.08	-1.28	-1.08
ILMN_1352453	Sav1_predicted	-1.17	-1.37	-1.15	-1.29	-1.22	-1.26	-1.08
ILMN_1368982	RGD1311709_predicted	1.19	1.2	1.22	1.49	-1.1	-1.26	-1.08
ILMN_1365458	Cldn12_predicted	1.09	-1.04	1.19	1.06	-1.36	-1.25	-1.08
ILMN_1374331	Sdhc	-1.12	-1.16	-1.11	-1.16	-1.19	-1.24	-1.08
ILMN_1373649	Spint2	-1.04	-1.11	-1.13	-1.27	-1.14	-1.23	-1.08
ILMN_1355635	LOC360354	-1.04	-1.09	-1.16	-1.36	-1.1	-1.23	-1.08
ILMN_1361276	Fubp1	-1.02	-1.31	1.11	-1.05	-1.31	-1.22	-1.08

ILMN_1356649	Rsn	1.23	1.02	1.01	-1.12	-1.13	-1.21	-1.08
ILMN_1363255	Ivns1abp_predicted	-1.03	1.22	1.27	1.02	-1.08	-1.21	-1.08
ILMN_1361724	Vps16	-1.07	-1.09	1.22	-1.08	-1.26	-1.2	-1.08
ILMN_1362070	Lrp10	-1.3	1.06	-1.27	-1.1	-1.18	-1.19	-1.08
ILMN_1368919	Terf2_predicted	-1.16	-1.12	-1.08	-1.15	-1.06	-1.18	-1.08
ILMN_1353249	LOC361571	-1.19	1.08	1.39	1.09	-1.29	-1.15	-1.08
ILMN_1358150	RGD1560953_predicted	1.05	1.06	-1.16	1.04	-1.11	-1.15	-1.08
ILMN_1359191	Tspan3	-1.04	-1.41	-1.23	-1.26	-1.15	-1.14	-1.08
ILMN_1361062	Tmem39b	1	-1.09	-1.26	-1.26	-1.21	-1.13	-1.08
ILMN_1351059	D123	-1.01	-1.27	-1.21	-1.28	-1.17	-1.13	-1.08
ILMN_1358186	Cops7b_predicted	1.16	1.05	1.31	1	-1.01	-1.13	-1.08
ILMN_1359743	Gloxd1	1.12	1.25	1.43	1.7	-1.16	-1.11	-1.08
ILMN_1351648	Mrpl13	1.04	-1.21	-1.4	-1.34	-1.11	-1.11	-1.08
ILMN_1358981	RGD1308290_predicted	-1.08	1.11	1.06	1.04	-1.02	-1.1	-1.08
ILMN_1349592	Top3a_predicted	1.02	-1.07	-1.01	1	-1.01	-1.1	-1.08
ILMN_1355190	Stim2_predicted	-1.25	-1.12	1.04	-1.16	-1.31	-1.09	-1.08
ILMN_1373335	Bmpr1a	-1.07	-1.19	-1.05	-1.23	-1.18	-1.09	-1.08
ILMN_1371818	Plcb3	1.2	1.12	-1.14	-1.01	-1.04	-1.09	-1.08
ILMN_1363150	Eif4ebp2	1.01	-1.22	1.1	-1.35	1.04	-1.09	-1.08
ILMN_2039948	Eef1a1	1.03	-1.11	1.19	-1.11	-1.09	-1.07	-1.08
ILMN_2038787	Eef1a1	1.03	-1.11	1.19	-1.11	-1.09	-1.07	-1.08
ILMN_1369684	Ric8a	1.06	1.26	-1.02	1.13	1.02	-1.07	-1.08
ILMN_1372578	Znrd1	1.09	-1.06	-1.3	1.22	-1.06	-1.06	-1.08
ILMN_1365419	Nhlrc2_predicted	1.03	1.09	-1.15	1.01	-1.24	-1.05	-1.08
ILMN_1352795	Stx12	-1.12	-1.12	1.25	-1.1	-1.06	-1.03	-1.08
ILMN_2039706	Bzw1	1.06	-1.02	1.27	1.23	-1.11	-1.02	-1.08
ILMN_1361338	Helz_predicted	-1.04	-1.21	-1.38	-1.46	-1.1	-1.01	-1.08
ILMN_1359259	Dnajc5	-1.14	1.11	-1.18	1.13	-1.05	-1.01	-1.08
ILMN_1350882	Eef1b2_predicted	-1	1.11	1.1	1.14	-1.02	-1.01	-1.08
ILMN_1361261	LOC680014	-1.07	-1.09	1.05	-1.14	1.1	-1.01	-1.08
ILMN_1376866	Zfp535	-1.23	-1.15	-1.06	-1.23	1	-1	-1.08
ILMN_1352950	LOC292116	-1.1	1.12	1.05	1.17	-1.14	1.02	-1.08
ILMN_1365189	RGD1561255_predicted	-1.08	1.23	-1.14	1.29	-1.07	1.02	-1.08
ILMN_1358344	Minpp1	-1.1	-1.06	-1.07	-1.14	-1.05	1.02	-1.08
ILMN_1355155	Aars	-1.05	1.31	1.58	1.25	1.11	1.02	-1.08
ILMN_1353820	Actr3	-1.08	1.08	1.13	1.05	-1.02	1.03	-1.08
ILMN_1358755	RGD1561067_predicted	-1.03	-1.23	-1.02	-1.13	1.16	1.03	-1.08
ILMN_1361411	Zfp95_predicted	-1.02	-1.18	-1.02	-1.12	-1.06	1.04	-1.08

ILMN_2038936	LOC687780	1.08	1.06	-1.08	1.05	1.02	1.04	-1.08
ILMN_1357252	Man2c1	-1.13	1.09	1.22	-1.02	-1.18	1.05	-1.08
ILMN_1374139	Aatf	1.06	1.3	1.78	1.56	-1.06	1.05	-1.08
ILMN_1351562	Casp12	1.33	1.08	1.08	-1.09	1.09	1.05	-1.08
ILMN_1353872	RGD1306184	-1.17	-1.14	-1.31	-1.26	1.11	1.08	-1.08
ILMN_1369074	Pgk1	1.05	-1.27	-1	-1.05	1.24	1.11	-1.08
ILMN_1372392	Zmynd10	-1.21	-1.08	-1.06	-1.19	-1.01	1.13	-1.08
ILMN_1372497	Car6	-1.02	-1.11	-1.31	-1.05	-1.01	1.13	-1.08
ILMN_1370828	Cth	1.1	1.35	1.88	1.48	1.43	1.15	-1.08
ILMN_1372523	Sdf2_predicted	-1.14	-1.06	-1.26	-1.28	-1.03	1.16	-1.08
ILMN_1350637	RGD1310211_predicted	-1.11	1.15	1.02	1.12	1.17	1.16	-1.08
ILMN_1350517	LOC502603	1	-1.02	-1.09	1.03	-1.01	1.17	-1.08
ILMN_1372737	LOC502603	1	-1.02	-1.09	1.03	-1.01	1.17	-1.08
ILMN_1357388	Zfp260	1.15	1.11	1.24	1.02	1.07	1.18	-1.08
ILMN_1650704	Birc2	1	-1.08	-1.11	-1.15	1.15	1.22	-1.08
ILMN_1367725	Zfp503_predicted	-1.01	1.98	1.23	1.97	1.58	1.35	-1.08
ILMN_1370972	Dtx2	-1.03	1.13	-1.01	1.05	1.32	1.47	-1.08
ILMN_1362541	RGD1307155	-1.01	-1.52	-1.29	-1.46	-1.13	-1.48	-1.07
ILMN_1349787	Bzrp	1.09	-1.11	-1.67	-1.43	-1.38	-1.43	-1.07
ILMN_1361139	Tmprss8	1.18	-1.22	-1.02	-1.82	-1.25	-1.41	-1.07
ILMN_1650211	RGD1307393	1.24	-1.14	1.1	1.16	-1.39	-1.35	-1.07
ILMN_1372317	LOC305375	-1.15	-1.39	-1.02	-1.02	-1.07	-1.34	-1.07
ILMN_1361657	Elac2	1.08	1.03	-1.05	-1.08	-1.45	-1.32	-1.07
ILMN_1370753	Agpat5_predicted	1.13	-1.03	1.16	1.08	-1.21	-1.32	-1.07
ILMN_1364524	Dnajc18	1.03	1.09	-1.09	1.02	-1.16	-1.32	-1.07
ILMN_1363410	Acox1	1.18	-1.1	-1.37	-1.44	-1.09	-1.31	-1.07
ILMN_1356965	Gtpbp6_predicted	-1.22	-1.03	-1.42	-1.27	1.01	-1.31	-1.07
ILMN_1364969	Hars2_predicted	-1.07	-1.25	1.46	-1.21	-1.3	-1.29	-1.07
ILMN_1368593	RGD1359593	-1.07	-1.04	-1.15	-1.28	-1.23	-1.29	-1.07
ILMN_1350622	Palmd	1	-1.3	-1.27	-1.64	-1.13	-1.29	-1.07
ILMN_1354695	Igbp1	1.05	1.12	-1.08	-1.23	-1.17	-1.26	-1.07
ILMN_1351593	Wdr48_predicted	-1.01	-1.04	1.06	-1.09	-1.29	-1.25	-1.07
ILMN_1354279	Mmp11	-1.05	-1.2	-1.21	-1.06	-1.14	-1.25	-1.07
ILMN_1374765	RGD1563940_predicted	-1.18	-1.01	-1.08	-1.11	-1.42	-1.22	-1.07
ILMN_1360572	Klhl12	-1.07	-1.13	1.1	-1.27	-1.15	-1.22	-1.07
ILMN_1352482	RGD1310857	1.03	-1.17	1.1	1.02	-1.14	-1.21	-1.07
ILMN_1375109	Cyp2d26	-1.21	1.22	1.06	-1.14	-1.27	-1.19	-1.07
ILMN_1355005	RGD1565549_predicted	1.02	-1.1	-1.26	-1.2	-1.22	-1.19	-1.07

ILMN_1353221	Alg5	1.02	-1.04	1.09	-1.07	-1.16	-1.19	-1.07
ILMN_1373132	RGD1303272	1.02	-1.17	-1.22	-1.2	-1.03	-1.19	-1.07
ILMN_1357786	Phtf1	-1.2	-1.05	-1.09	1.02	-1.07	-1.18	-1.07
ILMN_1649932	Tarsl1	1	1.09	1.16	-1.17	-1.1	-1.16	-1.07
ILMN_2039507	St5_predicted	-1.03	-1.09	-1.04	-1.07	-1.09	-1.16	-1.07
ILMN_1371126	Procr	-1.27	-1.03	1.21	-1.29	1.1	-1.16	-1.07
ILMN_2039928	Brd7_predicted	1.08	-1.1	-1.11	1.13	-1.1	-1.15	-1.07
ILMN_1370202	Pyp_mapped	1.14	-1.11	-1.01	1.05	-1.04	-1.15	-1.07
ILMN_1360420	Rnut1	-1.25	-1.15	1.07	-1.11	-1	-1.15	-1.07
ILMN_1349396	Trp53i13_predicted	-1.3	1.02	1.1	-1.05	-1.4	-1.14	-1.07
ILMN_1355574	RGD1310209_predicted	-1.09	1.04	-1.19	-1.17	-1.26	-1.14	-1.07
ILMN_1351815	Trap1	-1.06	-1.08	1.01	-1.1	-1.23	-1.13	-1.07
ILMN_1352118	LOC498525	1.26	1.14	-1.04	1.3	1.01	-1.13	-1.07
ILMN_1351536	F11r	-1.05	-1.4	-1.43	-2.06	1.07	-1.13	-1.07
ILMN_1370109	Coro1c_predicted	-1.05	1.07	1	1.23	1.05	-1.11	-1.07
ILMN_1364984	Azin1	-1.16	-1.16	1.2	1.05	1.16	-1.09	-1.07
ILMN_1359851	Papd4	-1.13	1.23	1.2	1.08	-1.2	-1.07	-1.07
ILMN_1367272	RGD1306300_predicted	1.06	1.13	1.02	1.04	-1.16	-1.07	-1.07
ILMN_1357441	RGD1564051_predicted	-1.06	-1.04	-1.02	-1.04	-1.09	-1.07	-1.07
ILMN_2039545	Coq6	-1.3	-1.24	-1.19	-1.26	-1.07	-1.07	-1.07
ILMN_1376546	Coq6	-1.3	-1.24	-1.19	-1.26	-1.07	-1.07	-1.07
ILMN_1371925	Hbld2	-1.16	-1.24	1.04	-1.05	1.04	-1.07	-1.07
ILMN_1650159	RGD1304670_predicted	-1.04	-1.32	-1.13	-1.26	-1.3	-1.06	-1.07
ILMN_1359149	Cherp_predicted	-1.05	1.2	1.01	1.25	-1.23	-1.06	-1.07
ILMN_1350370	Cherp_predicted	-1.05	1.2	1.01	1.25	-1.23	-1.06	-1.07
ILMN_1366251	RGD1311539_predicted	-1.18	-1.25	-1.05	-1.33	1.05	-1.06	-1.07
ILMN_1369190	Pdlim7	-1.06	1.73	1.62	2.24	1.12	-1.05	-1.07
ILMN_1368733	LOC682303	1.13	1.01	1.42	1.27	-1.12	-1.04	-1.07
ILMN_1359224	LOC499124	-1.1	-1.09	1.02	-1.03	-1.14	-1.03	-1.07
ILMN_1351163	LOC500199	1.08	1.26	1.33	1.3	-1	-1.03	-1.07
ILMN_1354168	LOC687582	-1.13	-1.22	-1.07	-1.34	1.06	-1.03	-1.07
ILMN_1373738	LOC683618	1.14	-1.01	-1.12	-1.11	1.01	-1.02	-1.07
ILMN_1360812	Ppp2r5e_predicted	-1.05	1.05	1.15	1.21	-1.07	-1	-1.07
ILMN_1371946	RGD1310738_predicted	1.02	1.22	1.06	1.21	-1.03	-1	-1.07
ILMN_1359142	Lrrc56	-1.18	-1.23	1.05	-1.1	-1	-1	-1.07
ILMN_1375211	Apaf1	-1.06	-1.22	-1.41	-1.17	1.08	-1	-1.07
ILMN_1371668	Apex1	1.03	-1.05	1.01	-1.49	-1.23	1	-1.07
ILMN_1368003	Cct6a_predicted	1.01	1.08	1.29	1.19	-1	1	-1.07

ILMN_1370575	RGD1561456_predicted	1.05	-1.06	1.13	1.23	1.01	1.01	-1.07
ILMN_1650200	Tspan6	-1.07	-1.35	-1.02	-1.14	1.09	1.01	-1.07
ILMN_1351401	Brwd1_predicted	-1.09	1.02	1.04	1.44	1.14	1.01	-1.07
ILMN_2040458	Prosc_predicted	1.01	1.51	1.59	1.34	1.15	1.01	-1.07
ILMN_1374973	Arhgap1_predicted	-1.03	-1.18	1.56	-1.11	-1.4	1.02	-1.07
ILMN_1351993	Cobll1_predicted	-1.15	-1.2	-1.1	-1.24	-1.12	1.03	-1.07
ILMN_1367549	RGD1304977_predicted	-1.09	-1.01	-1.14	-1.03	-1.14	1.05	-1.07
ILMN_1360062	RGD1311257	1.03	1.01	-1.04	1.26	1.26	1.05	-1.07
ILMN_1370934	Unc50	-1.12	-1.19	-1.18	-1.46	1.03	1.06	-1.07
ILMN_1372464	RGD1559643_predicted	-1.08	1.05	-1.09	-1.02	1.03	1.07	-1.07
ILMN_1363294	Zfp282_predicted	1.02	1.06	1.11	-1.06	-1.24	1.08	-1.07
ILMN_1366386	Tob2	1.03	1.26	-1.12	1.2	1.04	1.08	-1.07
ILMN_1356747	LOC363377	-1.02	1.02	1.09	1.01	1.09	1.08	-1.07
ILMN_1354974	Arid4b	-1.09	-1.03	-1.07	-1.08	1.16	1.08	-1.07
ILMN_1373866	Rps5	1.11	1.03	1.22	1.09	1.02	1.09	-1.07
ILMN_1359468	Dhcr24	1.03	-1.26	-1.23	-1.12	1.27	1.1	-1.07
ILMN_1364382	RGD1561537_predicted	1.02	1.02	-1.14	-1.07	-1.03	1.11	-1.07
ILMN_1361377	Xrn2_predicted	-1.04	1.23	1.16	1.07	1.01	1.11	-1.07
ILMN_1373826	Cdc5l	-1.06	-1.03	-1.25	-1.09	1.12	1.13	-1.07
ILMN_1366691	Vps4b	1	-1.21	1.18	-1.19	-1.04	1.15	-1.07
ILMN_1358168	LOC363897	-1.1	-1.26	1.08	-1.14	1.18	1.15	-1.07
ILMN_1367742	Ptprg	-1.08	1.36	1.13	1.04	-1.02	1.16	-1.07
ILMN_1376809	Ddost_predicted	-1.01	-1.4	1.34	-1.19	1.12	1.18	-1.07
ILMN_1368907	RGD1359460	1.14	1.56	1.33	1.76	1.05	1.2	-1.07
ILMN_1358520	LOC680460	1.07	-1.01	-1.04	-1.18	1.09	1.2	-1.07
ILMN_1650756	LOC680460	1.07	-1.01	-1.04	-1.18	1.09	1.2	-1.07
ILMN_1362636	RGD1563325_predicted	-1.44	-1.3	-1.14	-1.25	1.28	1.22	-1.07
ILMN_1371342	Zfp347	-1.25	1	-1.08	-1.16	1.08	1.23	-1.07
ILMN_1372502	Wdr45l_predicted	1.06	-1.11	1.28	1.09	1.48	1.24	-1.07
ILMN_1359975	Hps4_predicted	-1.05	1.18	1.24	1.02	1.18	1.27	-1.07
ILMN_1351296	LOC680856	1.06	1.28	1.33	1.24	1.24	1.37	-1.07
ILMN_1351141	LOC497936	-1.12	1.12	1.02	1.09	1.44	1.6	-1.07
ILMN_1368854	Gpx2	1.3	-1.76	-2.11	-1.57	-1.53	-1.77	-1.06
ILMN_1371247	Exoc7	-1.18	-1.19	-1.06	-1.22	-1.65	-1.48	-1.06
ILMN_1372302	RGD1306936_predicted	1	1.01	-1.23	1.09	-1.39	-1.48	-1.06
ILMN_1366167	Hdgfrp2	-1.2	-1.53	-1.22	-1.29	-1.19	-1.46	-1.06
ILMN_1367657	Ptpdc1_predicted	1.02	-1.02	-1.2	-1.02	-1.13	-1.45	-1.06
ILMN_1360969	Krim1	-1.06	-1.28	-1.15	-1.09	-1.71	-1.42	-1.06

ILMN_1363659	Tapbpl_predicted	1.17	-1.14	-1.28	-1.26	-1.32	-1.4	-1.06
ILMN_1349717	Spa17	-1.08	-1.61	-1.83	-1.88	-1.27	-1.4	-1.06
ILMN_1364554	Ech1	-1.1	-1.1	-1.26	-1.76	-1.29	-1.38	-1.06
ILMN_1365331	Scp2	-1.36	-1.48	-1.88	-2.03	-1.27	-1.35	-1.06
ILMN_1372217	Mosc2	1.01	-1.01	-1.16	-1.39	-1.32	-1.32	-1.06
ILMN_1371376	Stim1_predicted	1.06	1.04	1.18	1.02	-1.11	-1.32	-1.06
ILMN_1365272	Slc6a6	-1.02	-1.09	-1.49	-1.05	-1.43	-1.3	-1.06
ILMN_1364662	Zmynd19	1.18	1.28	1.38	1.71	-1.23	-1.28	-1.06
ILMN_1365992	Lamb2	-1.17	1.2	1.14	1.1	-1.34	-1.27	-1.06
ILMN_1350492	Stub1	-1.21	-1.28	1.18	-1.04	-1.25	-1.27	-1.06
ILMN_1349112	Exoc6	-1	1.11	-1.01	-1.02	-1.18	-1.27	-1.06
ILMN_2040879	LOC292072	-1.02	1.04	-1.05	1.03	-1.29	-1.25	-1.06
ILMN_1370818	Akr1c12_predicted	-1.18	-1.19	-1.27	-1.38	-1.21	-1.25	-1.06
ILMN_1367675	Pigs	-1.15	-1.44	-1.11	-1.39	-1.23	-1.22	-1.06
ILMN_1352630	Sigirr	-1.11	-1.17	-1.35	-1.22	-1.03	-1.22	-1.06
ILMN_1650742	Scoc	-1.13	-1.33	-1.19	-1.46	-1.27	-1.21	-1.06
ILMN_1374555	Gps2_predicted	1.04	1.25	-1.04	1.23	1.05	-1.21	-1.06
ILMN_1374663	RGD1306649	-1.07	-1.16	1.02	-1.1	-1.26	-1.2	-1.06
ILMN_1362726	Hmgn3	1.05	-1.11	-1.02	-1.05	-1.12	-1.2	-1.06
ILMN_1363954	Nip7	1.04	1.12	1.18	1.04	-1.13	-1.18	-1.06
ILMN_1368055	Ring1	-1.14	-1.11	-1.29	-1.28	-1.03	-1.18	-1.06
ILMN_1361681	RGD1563521_predicted	1.06	1.15	1.18	1.33	-1.31	-1.17	-1.06
ILMN_1351091	Ap2s1	-1.03	-1.4	-1.4	-1.18	-1.19	-1.17	-1.06
ILMN_1364331	Pgls_predicted	1.03	-1.12	-1.32	-1.13	-1.17	-1.16	-1.06
ILMN_1367338	Timm17a	1.01	-1.01	-1.09	1.03	-1.13	-1.16	-1.06
ILMN_1374910	Sh3bp5	1.14	1.07	-1.18	1.2	-1.04	-1.16	-1.06
ILMN_1357101	RGD1311324	-1.03	-1.18	-1.18	-1.07	1.01	-1.15	-1.06
ILMN_1373824	Cyfip1_predicted	-1.05	-1.01	-1.16	1.04	-1.25	-1.14	-1.06
ILMN_1363225	RGD1308557_predicted	-1.17	1.16	1.04	1.27	-1.01	-1.14	-1.06
ILMN_1356622	LOC309016	-1.15	-1.16	-1.18	-1.39	-1.08	-1.13	-1.06
ILMN_1369581	RGD1562618_predicted	-1.16	1.11	-1.05	1.09	-1.13	-1.12	-1.06
ILMN_1376269	Zfp513	1.08	1.17	1	1.14	-1.15	-1.11	-1.06
ILMN_1359898	Snrpe_predicted	1.17	1.16	-1.57	1.09	-1.14	-1.11	-1.06
ILMN_1374690	Snrpe_predicted	1.17	1.16	-1.57	1.09	-1.14	-1.11	-1.06
ILMN_1374578	RGD1307395	-1.21	-1.24	-1.11	-1.25	-1.1	-1.11	-1.06
ILMN_1370827	Rhod_predicted	-1.21	-1.15	-1.21	-1.1	1.11	-1.11	-1.06
ILMN_1373909	Npepl1_predicted	-1	-1.1	1.13	-1.07	-1.2	-1.1	-1.06
ILMN_1360761	RGD1561042_predicted	-1.06	-1.08	-1.11	-1.13	-1.18	-1.1	-1.06

ILMN_1376170	Rnps1_predicted	1	-1.27	1.03	-1.12	-1.14	-1.1	-1.06
ILMN_1374806	Mor1	-1	-1.19	-1.1	-1.23	-1.09	-1.1	-1.06
ILMN_1372953	RGD1304825_predicted	1.43	1.23	1.47	1.21	-1.16	-1.09	-1.06
ILMN_1359443	Pkcbpb15	-1.32	-1.04	1.11	-1.06	-1.13	-1.09	-1.06
ILMN_1375115	Rnf8	1.06	-1.18	-1.11	-1.11	-1.46	-1.08	-1.06
ILMN_1367256	Actr2	-1.16	-1.08	-1.01	-1.02	-1.04	-1.07	-1.06
ILMN_1372514	Letm1	1.14	1.35	1.35	1.54	-1	-1.07	-1.06
ILMN_1353584	RGD1310503_predicted	-1.27	-1.15	-1.15	-1.24	1.13	-1.07	-1.06
ILMN_1349311	LOC313940	-1.22	1.05	-1.07	-1.23	-1.03	-1.06	-1.06
ILMN_1373189	Ndst2_predicted	-1.07	1.01	-1.3	-1.06	-1.27	-1.05	-1.06
ILMN_1349005	Yme1l1	1.02	1.22	1.09	1.1	-1.05	-1.04	-1.06
ILMN_1355553	Ddx6	1.07	-1.17	-1.01	-1.18	1.09	-1.04	-1.06
ILMN_1353310	Sart1	-1.07	1.02	-1.18	1.07	-1.1	-1.03	-1.06
ILMN_1356190	Ahctf1_predicted	1.01	1.13	1.18	1.12	1.08	-1.03	-1.06
ILMN_1366083	RGD1565472_predicted	-1.13	-1.04	-1.05	-1.36	-1	-1.02	-1.06
ILMN_1369022	Prpf8	-1.09	-1.15	-1.12	1.02	1.13	-1.02	-1.06
ILMN_1358486	Phgdhl1	-1.16	-1.11	-1.15	-1.25	-1.01	1	-1.06
ILMN_2039175	Ctrl	-1.07	-1.15	-1.15	1.11	1	1.01	-1.06
ILMN_1366172	Mettl3	1.01	-1.1	-1.13	-1.05	-1.14	1.02	-1.06
ILMN_1366228	Ehmt2	-1.16	1.17	1.09	1.23	-1.14	1.02	-1.06
ILMN_1361504	Add1	1.32	-1.02	-1.07	-1.01	1.07	1.02	-1.06
ILMN_1371481	Psmb4	-1.01	-1.02	1.09	1.1	1.01	1.03	-1.06
ILMN_1363949	Arl6ip2	-1.07	-1.02	1.01	-1.09	-1.08	1.04	-1.06
ILMN_1357187	Wnt10b_predicted	-1.12	-1.11	-1.13	-1.01	1.11	1.04	-1.06
ILMN_1371061	Pcsk7	-1.09	-1.1	-1.14	-1.02	-1.28	1.05	-1.06
ILMN_1350242	Trpc4ap	-1.08	-1.08	1.08	-1.04	1.02	1.05	-1.06
ILMN_1376967	Ecgf1	1.05	1.14	1.56	1.15	1.06	1.05	-1.06
ILMN_1373328	Hrmt1l3	1.05	1.45	1.44	1.26	-1.02	1.06	-1.06
ILMN_1369431	Rpl8	-1.06	1.07	1.22	1.19	1.04	1.06	-1.06
ILMN_1350609	Mlf2_predicted	-1.05	-1.11	1.17	1.21	1.12	1.06	-1.06
ILMN_1353532	Pex13_predicted	-1.08	1.16	1.02	-1.03	1.15	1.07	-1.06
ILMN_1357078	Spred1	-1.07	1.09	1.19	1.06	1.11	1.08	-1.06
ILMN_1360643	Gdi1	-1.11	-1.29	1.77	-1.16	1.07	1.09	-1.06
ILMN_1370962	Kif9_predicted	1.02	1.3	-1.01	1.26	1.08	1.09	-1.06
ILMN_1367794	LOC294560	1.24	-1.1	1	-1.09	1.16	1.1	-1.06
ILMN_1356127	Ubap2_predicted	-1.09	-1.11	-1.18	-1.15	1.05	1.11	-1.06
ILMN_1350938	Dpp3	-1.11	-1.08	-1.55	-1.08	1.29	1.13	-1.06
ILMN_1372971	Dclre1a_predicted	1.07	1.26	1.02	1.15	-1.02	1.15	-1.06

ILMN_2039608	Dclre1a_predicted	1.07	1.26	1.02	1.15	-1.02	1.15	-1.06
ILMN_1651046	Irak1_predicted	1.05	1.03	1.19	-1.01	1.01	1.19	-1.06
ILMN_1358385	Ece1	-1.1	-1.28	-1.17	-1.02	-1.03	1.26	-1.06
ILMN_1371124	LOC500960	-1.02	-1.15	1.58	1.16	1.5	1.26	-1.06
ILMN_1367083	Papd1_predicted	1.03	1.37	1.59	1.6	1.27	1.27	-1.06
ILMN_1355233	LOC257650	-1.06	1.1	1.16	-1.2	1.13	1.28	-1.06
ILMN_1361423	Rkhd2_predicted	-1.19	1.23	1.21	1.14	1.39	1.36	-1.06
ILMN_1350784	Junb	-1.01	1.38	1.07	1.1	1.43	1.37	-1.06
ILMN_1356107	Atxn2l_predicted	-1.01	1.19	-1.03	1.52	1.15	1.41	-1.06
ILMN_1350965	Bcl10	-1.19	1.07	1.03	1.04	1.32	1.41	-1.06
ILMN_1348842	RGD1310724	-1.14	-1.17	-1.66	-1.52	-1.64	-1.61	-1.05
ILMN_1364069	Leng8	-1.35	-1.28	-1.54	-1.61	-1.44	-1.5	-1.05
ILMN_1356217	Slc35a1_predicted	-1.07	-1.18	-1.03	-1.18	-1.34	-1.39	-1.05
ILMN_1372363	RGD1566212_predicted	1.02	-1.04	-1.76	-1.19	-1.42	-1.38	-1.05
ILMN_1363839	Mfge8	1.02	-1.16	1.24	-1.19	-1.4	-1.34	-1.05
ILMN_1369248	RGD1564058_predicted	1.03	-1.11	-1.26	-1.23	-1.31	-1.33	-1.05
ILMN_1354950	Bmp1	-1.08	-1.11	-1.06	1.02	-1.33	-1.31	-1.05
ILMN_1372243	RGD1305347_predicted	1.1	-1.14	-1.25	-1.43	-1.21	-1.31	-1.05
ILMN_1650403	Pter	-1.13	-1.1	-1.03	-1.11	-1.27	-1.3	-1.05
ILMN_1356677	LOC679161	1.13	-1.01	1.11	1.04	-1.34	-1.29	-1.05
ILMN_1360736	Ugdh	1.24	-1.18	1.04	-1.16	-1.31	-1.26	-1.05
ILMN_1369538	Il17rc_predicted	-1.01	-1.01	-1.14	-1.06	-1.22	-1.25	-1.05
ILMN_1372479	RGD1311745	1.01	-1.28	-1.44	-1.19	-1.09	-1.25	-1.05
ILMN_1650921	Ttc5	1.01	1.07	-1.11	-1.09	-1.03	-1.25	-1.05
ILMN_1358742	MGC94326	-1.01	-1.13	-1.23	-1.1	-1.29	-1.23	-1.05
ILMN_1369425	Dusp22_predicted	1.04	-1.21	-1.1	-1.2	-1.11	-1.23	-1.05
ILMN_1374839	RGD1305356	-1.12	-1.2	-1.02	-1.05	-1.09	-1.23	-1.05
ILMN_1353957	Taf11	-1.28	-1.36	-1.38	-1.34	-1.14	-1.22	-1.05
ILMN_1359503	Ccndbp1	-1.02	-1.45	-1.28	-1.7	-1.14	-1.21	-1.05
ILMN_1375145	LOC497685	-1.03	1.09	1.28	-1.1	1.03	-1.21	-1.05
ILMN_2038784	Txn1	1.12	-1.25	-1.47	-1.17	-1.15	-1.2	-1.05
ILMN_2038783	Txn1	1.12	-1.25	-1.47	-1.17	-1.15	-1.2	-1.05
ILMN_1371958	Txn1	1.12	-1.25	-1.47	-1.17	-1.15	-1.2	-1.05
ILMN_1349881	MGC125214	-1.1	1.18	1.05	1.13	-1.21	-1.18	-1.05
ILMN_1651073	Pemt	-1.19	-1.51	-1.4	-1.49	-1.1	-1.18	-1.05
ILMN_1359178	Scarb1	1.01	1.03	-1.19	1.24	-1.13	-1.17	-1.05
ILMN_1359692	Ubtf	-1.16	-1.02	-1.08	1.03	-1.3	-1.16	-1.05
ILMN_1363654	Skp1a	-1.13	-1.32	-1.2	-1.19	-1.16	-1.16	-1.05

ILMN_1350624	Ppie	1.12	-1.16	-1.03	1	-1.17	-1.15	-1.05
ILMN_1365387	Loh12cr1_predicted	1.06	1.04	1.15	-1.08	1.02	-1.15	-1.05
ILMN_1367797	Mizf_predicted	-1.04	-1.16	-1.03	-1.17	-1.38	-1.14	-1.05
ILMN_1352073	RGD1305014	-1	1.02	1.22	1.25	-1.14	-1.14	-1.05
ILMN_1349486	Eml4_predicted	-1.05	1.04	-1.02	1.24	-1.2	-1.13	-1.05
ILMN_2039871	LOC366468	1.12	-1.01	-1.1	-1.15	-1.12	-1.13	-1.05
ILMN_1650456	LOC366468	1.12	-1.01	-1.1	-1.15	-1.12	-1.13	-1.05
ILMN_1367641	Git2	-1.19	1.01	1.1	-1.02	-1.05	-1.13	-1.05
ILMN_1355653	LOC300043	1.03	1.16	1.6	-1.01	-1.32	-1.1	-1.05
ILMN_1366244	RGD1566242_predicted	-1.06	-1.11	-1.14	-1	-1.2	-1.1	-1.05
ILMN_1355121	Rpusd4	1.14	1.07	1.12	1.03	-1.1	-1.1	-1.05
ILMN_1361937	Lta4h	-1.07	1.38	1.22	1.09	-1.08	-1.09	-1.05
ILMN_1366025	RGD1310475_predicted	-1.1	1.12	1.15	1.03	1.14	-1.09	-1.05
ILMN_1358761	LOC291914	-1.16	-1.29	-1.16	-1.1	-1.17	-1.08	-1.05
ILMN_1360192	Cxxc5	1.02	1.06	1.15	1.21	-1.06	-1.08	-1.05
ILMN_1356505	RGD1306062_predicted	1.12	1.12	1.31	1.13	-1.08	-1.07	-1.05
ILMN_1373510	Pskh1_predicted	1.15	1.03	-1.11	1.25	-1.04	-1.06	-1.05
ILMN_1354142	LOC299306	1.1	-1.11	1.16	1.19	1.01	-1.06	-1.05
ILMN_1359497	RGD1311458	-1	1.16	1.12	1.14	-1.17	-1.04	-1.05
ILMN_1355549	Cct2	-1.14	1.01	1.19	1.05	-1.06	-1.04	-1.05
ILMN_1361885	Phlda2_predicted	-1.1	1.14	1.05	1.46	-1.02	-1.04	-1.05
ILMN_1352182	Imp4	1.18	1.14	1.34	1.23	1.05	-1.04	-1.05
ILMN_2040387	Nup153	1.02	1.19	-1.14	1.28	-1.06	-1.03	-1.05
ILMN_1357965	Api5_predicted	1.14	-1.05	-1.16	1.04	-1.01	-1.03	-1.05
ILMN_2040432	Ngfrap1	1.17	-1.05	-1.17	-1.14	-1.11	-1.02	-1.05
ILMN_1367239	C8a_predicted	1.02	1.01	1.37	1.02	-1.1	-1.01	-1.05
ILMN_1349294	LOC302612	-1.14	-1.1	1.05	-1.08	1.27	-1.01	-1.05
ILMN_1362294	LOC498733	1.41	1.15	1.37	1.36	1.16	1	-1.05
ILMN_1363573	Acsl5	-1.11	1.04	1.05	-1.45	-1.13	1.01	-1.05
ILMN_1351822	Rps20	1.05	1.04	-1.11	-1.01	1.03	1.01	-1.05
ILMN_1349382	Nudt9	1.16	1.11	1.22	1.09	1.17	1.01	-1.05
ILMN_1362638	RGD1308377_predicted	-1.35	-1.46	-1.38	-1.09	-1.02	1.02	-1.05
ILMN_1352843	RGD1561940_predicted	1.03	1.22	1.14	1.14	-1.08	1.04	-1.05
ILMN_1369539	Ica1	1.03	1.11	1.35	-1.21	1.18	1.05	-1.05
ILMN_1362711	LOC499305	1.07	1.05	-1.15	1.07	1.09	1.06	-1.05
ILMN_1364811	Baz2b_predicted	-1.12	1.21	1.12	-1.23	1.16	1.06	-1.05
ILMN_1376843	Cnot2	-1.01	1.05	1.08	-1.19	-1.06	1.07	-1.05
ILMN_1371916	Rps15	1.02	1.2	-1.15	1.2	1.07	1.07	-1.05

ILMN_1356787	Gtf2a2	-1.05	-1.32	-1.51	-1.23	1.02	1.08	-1.05
ILMN_1650235	Tmem87a_predicted	1.17	-1.06	1.15	-1.14	1.02	1.08	-1.05
ILMN_1376696	Polr3c	-1.01	1.01	1.09	1.01	1.07	1.08	-1.05
ILMN_1375009	Htatip	-1.15	1.05	-1.02	-1.24	-1.02	1.09	-1.05
ILMN_1360815	Adam17	-1.01	-1.03	-1.36	-1.02	1.06	1.09	-1.05
ILMN_1349207	Id2	1.23	1.09	1.56	-1.05	1.02	1.1	-1.05
ILMN_1370412	Oxsr1_predicted	1.07	-1.07	1.08	-1.07	-1.02	1.12	-1.05
ILMN_1365998	RGD1561337_predicted	1.05	1.13	1.07	-1.1	1.05	1.12	-1.05
ILMN_1364685	Cul1_predicted	1.05	1.43	1.51	1.44	1.13	1.12	-1.05
ILMN_1371038	Mtrr	1.09	1.06	-1.03	1.04	-1.18	1.14	-1.05
ILMN_1366756	Cpsf6_predicted	1.12	1.46	1.44	1.49	-1.04	1.14	-1.05
ILMN_1367956	Zcchc11_predicted	1.1	1.28	-1.35	-1.22	1.1	1.14	-1.05
ILMN_1360101	Ube2d3	-1.11	1.01	1.12	1.15	1.15	1.14	-1.05
ILMN_1368080	Tmem43	-1.03	-1.37	-1.33	-1.33	-1.06	1.15	-1.05
ILMN_1368990	Nat5_predicted	1.08	1.16	1.04	1.16	1.3	1.17	-1.05
ILMN_1366825	Plekhm2_predicted	-1.07	1.4	-1.2	1.32	1.05	1.19	-1.05
ILMN_1360401	Mesdc1	1.17	1.43	1.01	1.44	1.16	1.19	-1.05
ILMN_1352443	Ctbp2	-1.04	1.16	1.12	1.26	1.35	1.2	-1.05
ILMN_1650897	RGD1310311	-1.1	1.14	1.13	1.3	1.14	1.22	-1.05
ILMN_1356332	RGD1565350_predicted	1.03	1.37	1.13	1.12	1.26	1.22	-1.05
ILMN_1371645	Lsr	-1.01	1.36	1.29	1.43	1.25	1.23	-1.05
ILMN_1369252	LOC316122	-1.03	1.13	1.09	-1.11	-1.07	1.25	-1.05
ILMN_1353694	Appbp2	1.05	1.22	1.09	1.34	1.19	1.32	-1.05
ILMN_1374632	Cebpa	1.1	1.49	1.43	1.3	1.3	1.33	-1.05
ILMN_1368890	Wfikkn1_predicted	-1.08	1.11	-1.01	-1	1.47	1.86	-1.05
ILMN_1371263	LOC500307	-1.03	1.94	1.63	1.29	2.18	1.9	-1.05
ILMN_1370709	Mgst2_predicted	1.02	1.15	-1.36	-1.21	-1.64	-1.72	-1.04
ILMN_1350417	RGD1565496_predicted	1.21	1.02	-1.01	-1.24	-1.34	-1.57	-1.04
ILMN_1650096	MGC116363	1.03	-1.29	-1.19	-1.32	-1.3	-1.49	-1.04
ILMN_1360926	RGD1311364	-1.05	-1.66	-1.74	-1.88	-1.15	-1.43	-1.04
ILMN_1650419	RGD1564089_predicted	-1.05	-1.02	1.08	-1.2	-1.45	-1.35	-1.04
ILMN_1352132	Fuca2	1.06	1.09	1.07	-1.14	-1.41	-1.35	-1.04
ILMN_1349186	LOC361014	-1.05	-1.53	-1.39	-1.6	-1.12	-1.32	-1.04
ILMN_1349831	RGD1561492_predicted	-1.1	-1.13	-1.22	-1.28	-1.27	-1.31	-1.04
ILMN_1370841	Cox15_predicted	1.08	-1.07	-1.13	-1.1	-1.27	-1.3	-1.04
ILMN_1352184	Smarca4	-1.03	-1.3	-1.06	-1.25	-1.35	-1.29	-1.04
ILMN_1348778	LOC300472	1.08	-1.06	-1.08	1.17	-1.04	-1.26	-1.04
ILMN_1360537	Ext2_predicted	-1.21	-1.32	-1.01	-1.5	-1.1	-1.24	-1.04

ILMN_1358618	Rsbn1l_predicted	1.06	1.03	-1.04	1.02	-1.21	-1.23	-1.04
ILMN_1350748	Abhd14b	-1.02	1.24	1.34	1.01	-1.03	-1.22	-1.04
ILMN_1359639	RGD1310313_predicted	-1.09	-1.31	-1.38	-1.94	-1.27	-1.21	-1.04
ILMN_1360919	RGD1563853_predicted	-1.06	-1.16	-1.09	-1.1	-1.22	-1.21	-1.04
ILMN_1355935	LOC503479	1.13	1.21	1.28	1.08	1.07	-1.2	-1.04
ILMN_1368917	Bcap29	-1.13	1.04	1.19	-1.09	-1.2	-1.19	-1.04
ILMN_1351519	Taf9	-1.13	1.08	1.1	-1.02	-1.18	-1.19	-1.04
ILMN_1351043	Slc35a4	-1.22	-1.19	-1.15	1.06	-1.08	-1.19	-1.04
ILMN_1354619	LOC295496	-1.07	1.06	-1.07	1.1	-1.17	-1.18	-1.04
ILMN_1361358	LOC288978	1.09	-1.14	-1.34	-1.14	-1.08	-1.18	-1.04
ILMN_1371860	Kif16b_predicted	-1.02	-1.13	-1.13	-1.18	-1.04	-1.18	-1.04
ILMN_1368366	Smarca5_predicted	-1.22	-1.09	1.02	1.01	-1	-1.18	-1.04
ILMN_1376593	B3gnt7	-1.12	-1.3	-1.15	-1.11	-1.33	-1.16	-1.04
ILMN_1374392	Nr1i2	-1.14	-1.02	1.03	-1.07	-1.12	-1.16	-1.04
ILMN_1349565	LOC313340	-1.1	-1.12	-1.02	-1.18	-1.08	-1.16	-1.04
ILMN_1354512	Ranbp1_predicted	-1.08	-1.14	-1.03	1.17	-1.12	-1.12	-1.04
ILMN_1352406	Ddx51_predicted	-1.06	-1.16	1.06	1.24	-1.03	-1.12	-1.04
ILMN_1374051	Med31_predicted	-1.05	-1.08	-1.31	-1.01	-1.45	-1.11	-1.04
ILMN_1361943	LOC362290	-1.09	1.07	1.29	-1.05	-1.1	-1.11	-1.04
ILMN_1370384	Rbm27_predicted	-1.05	1.02	-1.14	1.07	-1.15	-1.1	-1.04
ILMN_1353779	Pigw	1.08	-1.02	1.13	1.19	1.04	-1.1	-1.04
ILMN_1650920	RGD1559623_predicted	1	-1.06	-1.12	-1.15	-1.14	-1.07	-1.04
ILMN_1356654	Fkbp1a	1.03	-1.15	-1.04	-1.03	-1.03	-1.07	-1.04
ILMN_1364746	Abhd6	-1.07	1.1	1.22	1.13	1.05	-1.06	-1.04
ILMN_1365286	Axin2	1.19	1.1	1.05	1.32	1.09	-1.06	-1.04
ILMN_1368252	RGD1306811_predicted	-1.01	-1.19	-1.4	-1.54	-1.31	-1.05	-1.04
ILMN_1650535	LOC361128	1.24	1.07	1.27	1.2	1.08	-1.05	-1.04
ILMN_1368833	Nol1_predicted	-1.02	1.24	1.37	1.39	-1.14	-1.04	-1.04
ILMN_1366878	RGD1562258_predicted	1.04	1.18	-1.32	-1.19	-1.13	-1.04	-1.04
ILMN_1351423	Dtwd1	-1.06	-1.06	1.22	1.3	-1.1	-1.04	-1.04
ILMN_1367251	Pttg1ip	-1.26	-1.29	-1.16	-1.67	-1.03	-1.03	-1.04
ILMN_2040517	Ddx42_predicted	1.04	-1.17	1.17	-1.06	1.05	-1.03	-1.04
ILMN_1350658	Sacs_predicted	1.07	-1.01	1.18	1.13	-1.39	-1.02	-1.04
ILMN_1358130	Taf6	-1.06	-1.01	-1.32	-1.26	-1.17	-1	-1.04
ILMN_1351227	Pigh_predicted	-1.01	-1.09	1.4	-1.1	-1.1	-1	-1.04
ILMN_1352686	Grsf1	1.04	1.15	1.18	1.13	-1.02	1.01	-1.04
ILMN_1376287	Golph3l	-1.08	-1.96	-1.55	-2	-1.19	1.02	-1.04
ILMN_1359668	LOC499076	1.06	1.24	-1.06	1.16	-1.03	1.02	-1.04

ILMN_1359787	LOC498142	1.15	1.13	1.39	1.52	1.14	1.02	-1.04
ILMN_1353217	Srp68_predicted	-1.09	1.25	1.18	1.2	1.04	1.03	-1.04
ILMN_1360376	App	-1.04	-1.18	-1.03	-1.21	-1.12	1.04	-1.04
ILMN_1369824	Ppp4r1	1.07	1.12	-1.06	1.08	-1.03	1.04	-1.04
ILMN_1351972	RGD1562259_predicted	-1.01	1.04	-1.11	1.01	1.07	1.04	-1.04
ILMN_1351629	RGD1562259_predicted	-1.01	1.04	-1.11	1.01	1.07	1.04	-1.04
ILMN_1357031	RGD1310722_predicted	-1.1	1.1	-1.22	-1.09	1.1	1.04	-1.04
ILMN_1355973	RGD1559845_predicted	1.05	-1.03	-1.11	1.02	-1.04	1.06	-1.04
ILMN_1358443	Eif2b2	1.01	1.01	1.09	1.03	1.01	1.07	-1.04
ILMN_1361731	Pabpn1	1.15	1.1	-1.4	1.02	-1.01	1.09	-1.04
ILMN_1358719	Rps21	1.15	1.24	-1.64	1.13	1.06	1.1	-1.04
ILMN_1352668	Arfip2	-1.06	1.15	1.17	1.16	1.11	1.1	-1.04
ILMN_1353839	Per1	1	1.24	1.16	1.24	1.31	1.1	-1.04
ILMN_1376568	Sec11l1	1.1	-1.11	-1.07	-1.18	1.21	1.11	-1.04
ILMN_1349159	LOC498433	1.04	1.02	-1.16	1.04	1.09	1.14	-1.04
ILMN_1361951	Xpo6	1.06	1.14	-1.07	1.5	1.35	1.15	-1.04
ILMN_1367428	Zfp189_predicted	-1.04	1.17	1.07	1.1	1.27	1.19	-1.04
ILMN_1371994	Traf7_predicted	-1.19	-1.11	-1.2	1.09	-1.02	1.2	-1.04
ILMN_1353352	RGD1562747_predicted	-1.04	-1.22	-1.43	-1.56	1.11	1.2	-1.04
ILMN_1376905	Abi1	1	1.11	1.12	-1.03	1.26	1.2	-1.04
ILMN_1354393	RGD1560110_predicted	-1.06	1.04	1.17	-1.07	1.04	1.22	-1.04
ILMN_1348920	RGD1304592_predicted	1.11	1.07	1.39	1	1.1	1.22	-1.04
ILMN_1369680	Slc30a7	-1	-1.07	1.14	1.02	1.18	1.22	-1.04
ILMN_1355184	Nans_predicted	1.1	-1.09	1.14	1.21	1.39	1.23	-1.04
ILMN_1349081	RGD1306941_predicted	1.52	1.44	1.98	1.63	1.49	1.24	-1.04
ILMN_1370207	Crbn	-1.08	-1	-1.21	-1.57	-1.07	1.25	-1.04
ILMN_1367139	LOC503418	1.05	-1.12	-1.24	-1.27	1.2	1.25	-1.04
ILMN_1365476	Tfip11	1.03	-1.06	-1.58	-1.28	1.11	1.39	-1.04
ILMN_1362553	Hadh2	1.05	-1.3	-1.68	-1.68	-1.83	-1.58	-1.03
ILMN_1364085	LOC499716	-1.13	-1.01	-1.41	-1.04	-1.38	-1.48	-1.03
ILMN_1372959	Aldh5a1	-1.07	1.25	1.09	-1.09	-1.37	-1.42	-1.03
ILMN_1651020	RGD1307789	1.29	-1.06	1.26	-1.2	-1.19	-1.34	-1.03
ILMN_1375233	Vps24	-1.18	-1.6	-1.49	-1.5	-1.33	-1.33	-1.03
ILMN_1368944	Rad52_predicted	1.33	1.23	1.24	1.09	-1.42	-1.31	-1.03
ILMN_1350172	LOC500155	-1.09	-1.44	-2.1	-1.52	-1.33	-1.27	-1.03
ILMN_1366797	LOC501120	-1.02	-1.01	-1.35	-1.13	1.03	-1.27	-1.03
ILMN_1349654	Crebl1	1.12	1.37	1.73	1.33	-1.29	-1.24	-1.03
ILMN_1357054	Arf5	-1.08	-1.3	-1.5	-1.26	-1.13	-1.24	-1.03

ILMN_1350680	Mrpl27_predicted	-1.02	-1.03	-1.23	-1.01	-1.22	-1.23	-1.03
ILMN_1360371	RGD1564709_predicted	1.08	-1.04	1.1	-1.32	-1.21	-1.22	-1.03
ILMN_1352727	B4galt7	1.05	-1.33	-1.45	-1.33	-1.2	-1.22	-1.03
ILMN_1373830	Skiv2l	-1.15	-1.03	-1.15	1.02	-1.18	-1.2	-1.03
ILMN_1350474	RGD1559939_predicted	-1.03	1.15	-1.3	-1.02	-1.14	-1.2	-1.03
ILMN_1358167	LOC293871	1.16	1.09	1.14	1.05	-1.06	-1.2	-1.03
ILMN_1351695	Asb8_predicted	-1.37	-1.36	-1.33	-1.22	-1.29	-1.19	-1.03
ILMN_1649728	RGD1305915	1.1	-1.09	1.11	-1.13	-1.21	-1.19	-1.03
ILMN_1650833	Zbtb7b_predicted	-1.01	-1.17	-1.05	-1.16	-1.13	-1.18	-1.03
ILMN_1366369	Tomm34_predicted	-1.05	-1.01	1.47	1.05	-1.09	-1.18	-1.03
ILMN_1349239	Tceb2	1.04	-1.29	-1.95	-1.16	-1.18	-1.17	-1.03
ILMN_1375059	RGD1565289_predicted	-1.06	-1.1	1.04	-1.12	-1.01	-1.17	-1.03
ILMN_1366722	KIFC2	-1.03	1.19	-1.03	1.01	-1.39	-1.16	-1.03
ILMN_1376746	Gng10	-1.14	-1.33	1.09	-1.14	-1.16	-1.16	-1.03
ILMN_2040519	Rbx1	1.04	1.16	-1.39	1.06	-1.3	-1.14	-1.03
ILMN_1362740	RGD1565059_predicted	1.14	-1.04	1.17	1.02	-1.04	-1.14	-1.03
ILMN_1350762	Polr2h_predicted	1.12	1.2	-1.17	1.17	-1.15	-1.13	-1.03
ILMN_1368428	Polr2h_predicted	1.12	1.2	-1.17	1.17	-1.15	-1.13	-1.03
ILMN_1357565	Hdac1_predicted	1.05	-1.02	-1.11	-1.11	-1.06	-1.13	-1.03
ILMN_1373245	RGD1309605_predicted	1.05	1.07	1.33	-1.26	-1.1	-1.11	-1.03
ILMN_1354240	Ripk3	-1	-1.09	1.07	1.19	1.2	-1.11	-1.03
ILMN_1350367	RGD1307896_predicted	-1.08	-1.3	-1.12	-1.2	-1.13	-1.1	-1.03
ILMN_1354716	RGD1560258_predicted	1.31	1.26	-1.07	1.32	1.08	-1.1	-1.03
ILMN_1366288	March5_predicted	-1.02	1.11	1.08	-1.16	1.06	-1.08	-1.03
ILMN_1364409	Ndufb9_predicted	1.05	-1.17	-1.58	-1.15	1.06	-1.06	-1.03
ILMN_1649917	LOC363675	-1.23	-1.3	-1.08	-1.4	-1.1	-1.04	-1.03
ILMN_1362355	RGD1306157_predicted	1	-1.18	-1.36	-1.29	-1.1	-1.04	-1.03
ILMN_1356633	LOC499709	-1.09	1.15	-1.09	1.18	-1.09	-1.03	-1.03
ILMN_1360410	RGD1307882_predicted	-1.04	1.13	1.05	1.17	-1.03	-1.03	-1.03
ILMN_1362078	RGD1307882_predicted	-1.04	1.13	1.05	1.17	-1.03	-1.03	-1.03
ILMN_1354883	RGD1309634_predicted	-1	1.04	-1.13	-1	1.01	-1.03	-1.03
ILMN_1376263	Sp140	-1.01	-1.09	-1.26	-1.15	-1.04	-1.02	-1.03
ILMN_1370891	Pcsk9	1.05	1.05	1.64	1.19	-1.01	-1.01	-1.03
ILMN_1368744	LOC298675	-1.07	-1.3	1.08	-1.04	-1	-1.01	-1.03
ILMN_1373357	Sphk1	1.2	1.19	1.46	1.19	1.25	-1	-1.03
ILMN_1368923	Taf2	-1.04	-1.03	1.04	-1.1	1.27	-1	-1.03
ILMN_1349744	Slc29a3	1.01	-1.05	-1.07	1.04	-1.17	1	-1.03
ILMN_1352091	Tmed10	-1.11	-1.14	1.1	-1.18	-1.07	1.01	-1.03

ILMN_1364695	RGD1565131_predicted	-1.02	1.08	1.02	1.07	1.04	1.01	-1.03
ILMN_1367677	Smndc1_predicted	-1.1	-1.04	-1.09	-1.06	1.12	1.01	-1.03
ILMN_1369235	Cirbp	-1.11	-1.13	-1.13	-1.26	-1.08	1.02	-1.03
ILMN_1363355	Pggt1b	-1.09	1.08	1.27	-1	-1.06	1.02	-1.03
ILMN_1374653	Psmd7_predicted	-1.03	1	1.21	1.13	-1.03	1.02	-1.03
ILMN_1366959	Slc26a6_predicted	-1.14	-1.04	1.2	1.15	1.2	1.02	-1.03
ILMN_1355843	Mrpl32_predicted	1.11	1.05	-1.45	-1.17	1.01	1.03	-1.03
ILMN_1353814	LOC362852	-1.05	1.09	-1.51	-1.04	1.05	1.03	-1.03
ILMN_1650551	LOC362852	-1.05	1.09	-1.51	-1.04	1.05	1.03	-1.03
ILMN_1351202	Smg7_predicted	-1.14	1.2	1.12	1.2	1.13	1.03	-1.03
ILMN_1366899	Supv3l1	-1.17	-1.13	1.26	-1.02	-1.22	1.04	-1.03
ILMN_1366132	RGD1311861_predicted	1.2	-1.08	1.09	1.04	-1.06	1.04	-1.03
ILMN_1351233	Wrnip1	1.12	1.26	1.16	1.23	-1.02	1.04	-1.03
ILMN_2039012	Chuk_predicted	1.09	1.04	1.28	1.1	1.13	1.04	-1.03
ILMN_1376541	Rps25	1.17	1.1	-1.15	1.1	1.03	1.05	-1.03
ILMN_1372272	Abt1	-1.3	-1.02	-1.12	-1.06	-1.05	1.06	-1.03
ILMN_1375968	Sf3b4_predicted	-1.26	1.05	-1.25	-1.05	1.03	1.06	-1.03
ILMN_1374661	Lipa	1.16	-1.02	1.31	1.06	1.03	1.06	-1.03
ILMN_1350806	RGD1310950_predicted	1.12	1.07	-1.04	1.15	1.12	1.07	-1.03
ILMN_1357141	Rft1_predicted	1.26	-1.08	1.21	-1.09	-1.04	1.08	-1.03
ILMN_1372364	Acyp1_predicted	1.23	1.06	-1.19	1.03	1.14	1.09	-1.03
ILMN_1374807	Ddx46	1	-1.01	-1.35	-1	-1.05	1.1	-1.03
ILMN_1370151	Cdk7	-1.15	-1.06	-1.04	-1.16	1.06	1.1	-1.03
ILMN_1371678	LOC683674	-1.05	1.15	1.2	1.1	-1.04	1.11	-1.03
ILMN_1351628	Mfap3	-1.19	-1.1	1.12	1.11	1.06	1.11	-1.03
ILMN_1356186	Rtn4	-1.02	-1.12	-1.25	1.05	1.07	1.11	-1.03
ILMN_1359100	Af6	1.09	1.16	1.4	1.06	1.14	1.12	-1.03
ILMN_1361469	RGD1306820_predicted	-1.02	1.14	-1.04	1.23	1.11	1.13	-1.03
ILMN_1351924	Trip4_predicted	-1.19	1.13	1.02	1.04	1.24	1.17	-1.03
ILMN_1375928	Sfxn1_predicted	-1.06	-1.04	1.19	-1.11	1.08	1.18	-1.03
ILMN_1354798	RGD1310592_predicted	-1.07	1.06	-1.11	1.28	1.14	1.18	-1.03
ILMN_1364296	Rbm28_predicted	-1.02	-1.04	1.19	1.15	1.04	1.19	-1.03
ILMN_1350525	Xpnpep1	1.02	1.02	1.18	1.04	1.16	1.19	-1.03
ILMN_1373089	LOC292082	-1.15	-1.01	-1.1	-1.26	1.03	1.2	-1.03
ILMN_1354411	RGD1562874_predicted	-1.16	-1.13	1.17	-1.23	1.12	1.21	-1.03
ILMN_1376410	Rbms1	-1.06	1.13	1.08	-1.01	1.12	1.21	-1.03
ILMN_1368752	LOC499378	1.06	-1.04	-1.07	-1.05	1.13	1.23	-1.03
ILMN_1358192	Dctn6_predicted	1.01	1.13	-1.07	1.06	1.05	1.25	-1.03

ILMN_1368369	Fhl3_predicted	-1.12	1.62	1.49	1.34	1.5	1.44	-1.03
ILMN_1350627	MGC124555	1.13	-1.4	1.21	-1.31	1.32	1.46	-1.03
ILMN_1353341	Hddc3_predicted	1.15	1.05	-1.11	-1.13	-1.33	-1.59	-1.02
ILMN_1367913	Trmt5_predicted	-1.02	-1.08	1.15	1.1	-1.26	-1.37	-1.02
ILMN_1365606	Tmem103_predicted	-1.03	-1.42	-1.19	-1.48	-1.23	-1.34	-1.02
ILMN_1650953	Rfxank	-1.07	-1.06	-1.3	-1.16	-1.45	-1.33	-1.02
ILMN_1367466	Pex11b	-1	-1.12	1.21	1.06	-1.3	-1.33	-1.02
ILMN_1367916	Dffa	1.01	-1.06	-1.2	1.02	-1.28	-1.33	-1.02
ILMN_1365812	Kbtbd4_predicted	-1.25	-1.07	-1.41	-1.16	-1.36	-1.27	-1.02
ILMN_1362835	RGD1308492	1.08	-1.12	-1.52	-1.26	-1.08	-1.27	-1.02
ILMN_1349584	Fam51a1	-1.06	-1.28	-1.43	-1.59	-1.39	-1.26	-1.02
ILMN_1370511	Atp5g1	1.1	-1.25	-1.4	-1.13	-1.04	-1.25	-1.02
ILMN_1366259	Plekhj1	1.04	-1.09	-1.05	-1.13	-1.01	-1.25	-1.02
ILMN_1365135	Acy3	-1.31	-1.05	-1.1	-1.37	-1.31	-1.24	-1.02
ILMN_1367824	RGD1309263_predicted	1.04	-1.25	-2.41	-1.31	-1.16	-1.23	-1.02
ILMN_1354787	RGD1309263_predicted	1.04	-1.25	-2.41	-1.31	-1.16	-1.23	-1.02
ILMN_1373115	Mrps36_predicted	-1.01	-1.22	-1.81	-1.37	-1.15	-1.23	-1.02
ILMN_1367848	Rrs1_predicted	1.02	1.26	1.25	1.29	-1.06	-1.23	-1.02
ILMN_1365897	RGD1306576_predicted	1.03	1.21	-1.14	1.4	-1.13	-1.21	-1.02
ILMN_1364531	Impa1	-1.04	-1.2	1.02	-1.06	-1.11	-1.21	-1.02
ILMN_1368106	RGD1305651	-1.13	-1.08	-1.14	-1.11	-1.33	-1.2	-1.02
ILMN_1355572	RGD1305986_predicted	-1.13	-1.06	1.15	-1.19	-1.18	-1.2	-1.02
ILMN_1370153	LOC502613	-1.04	-1.31	-1.48	-1.48	-1.31	-1.19	-1.02
ILMN_1372506	Alg9_predicted	1.01	-1.19	-1.03	-1.11	-1.28	-1.19	-1.02
ILMN_1370866	Ttc8_predicted	-1.01	-1.16	-1.29	-1.11	-1.17	-1.19	-1.02
ILMN_1354916	Lztr1_predicted	-1.06	1.09	-1.05	-1.01	-1.12	-1.19	-1.02
ILMN_1363236	Ufc1	-1.19	-1.14	-1.29	-1.14	-1.19	-1.18	-1.02
ILMN_1373382	Mrps14_predicted	-1.12	-1.37	-1.23	-1.46	-1.06	-1.18	-1.02
ILMN_1365666	Nqo2	-1.18	-1.18	-1.37	-1.84	-1.51	-1.17	-1.02
ILMN_1360171	E2f4_predicted	1	-1.02	1.38	1.25	-1.05	-1.17	-1.02
ILMN_1373798	Plcb1	1.11	1.12	-1.02	-1.02	1.11	-1.17	-1.02
ILMN_1357407	RGD1566224_predicted	1.05	1.03	-1.01	1.04	-1.15	-1.16	-1.02
ILMN_1352602	RGD1306698_predicted	1.03	1.04	1.26	1.09	-1.04	-1.16	-1.02
ILMN_1372495	Polr2a_mapped	-1.23	-1.15	-1.54	-1.29	-1.42	-1.14	-1.02
ILMN_1350035	Gstt1	-1.05	1.03	-1.08	-1.26	-1.08	-1.14	-1.02
ILMN_1367698	RGD1305671_predicted	-1.14	-1.07	-1.14	1.17	-1.05	-1.13	-1.02
ILMN_1362834	Dusp6	-1.35	1.29	1.31	-1.11	-1.11	-1.12	-1.02
ILMN_1356397	Mark3	1.01	-1	1.08	-1.01	-1.07	-1.1	-1.02

ILMN_1376505	Ttll1	-1.09	-1.09	-1.02	-1.11	1.03	-1.1	-1.02
ILMN_1363054	Etnk2_predicted	1.1	1.08	1.02	-1.1	1.08	-1.1	-1.02
ILMN_1367796	RGD1310191	1.1	-1.11	-1.43	-1.13	-1.12	-1.09	-1.02
ILMN_1360747	Commd3	1.04	-1.03	-1.24	-1.46	-1.11	-1.09	-1.02
ILMN_1364641	Tcta	-1.08	-1.5	-1.09	-1.4	-1.06	-1.09	-1.02
ILMN_1355210	Atp5j	1.04	-1.19	-1.66	-1.02	-1.03	-1.09	-1.02
ILMN_1355219	Ipmk	-1.15	1.01	-1.11	-1.05	-1.17	-1.08	-1.02
ILMN_1370009	Rpl4	-1.01	1	1.21	1.06	-1.06	-1.08	-1.02
ILMN_1372330	Capza2	-1.27	-1.23	-1.04	-1.16	-1.13	-1.07	-1.02
ILMN_1370745	RGD1560212_predicted	-1.07	1.06	1.24	1.13	-1.1	-1.07	-1.02
ILMN_1366520	LOC499293	1.26	-1.16	-1.22	-1.34	-1.06	-1.07	-1.02
ILMN_1366222	LOC501416	-1.03	-1.35	1.28	-1.11	-1.27	-1.06	-1.02
ILMN_1375469	RGD1307826_predicted	-1.1	-1.01	1.04	1.28	-1.12	-1.05	-1.02
ILMN_1360796	Scap_predicted	1.01	1.11	1.09	1.01	-1.05	-1.05	-1.02
ILMN_1357504	MGC94192	-1.25	-1.01	1.02	1.13	-1.05	-1.05	-1.02
ILMN_1373185	Obfc2b	-1.07	-1.11	-1.2	1.01	-1.01	-1.05	-1.02
ILMN_1367376	Cacybp	-1.02	1.14	1.39	1.25	-1.01	-1.05	-1.02
ILMN_1369921	Usp7_predicted	1.03	1.12	-1.2	1.12	1.06	-1.05	-1.02
ILMN_1372195	Rabl3_predicted	-1.04	1.05	1.22	-1.05	1.07	-1.05	-1.02
ILMN_1650841	LOC683534	-1.02	-1.23	1.37	-1.03	-1.13	-1.03	-1.02
ILMN_1370179	Eif3s5_predicted	-1.11	1.15	1.11	-1.01	1	-1.03	-1.02
ILMN_1372262	Rutbc3	-1.21	-1	1.1	-1.1	1.09	-1.03	-1.02
ILMN_1368122	Dicer1	1.05	1.01	1.06	1.04	-1.16	-1.02	-1.02
ILMN_1373973	RGD1565047_predicted	-1.21	-1.01	1.01	1.15	-1.01	-1.02	-1.02
ILMN_1373472	Ube2l6	-1.01	-1.42	-1.55	-1.49	1.07	-1.02	-1.02
ILMN_1372040	RGD1307982	1.12	1.2	1.23	1.26	1.02	-1.01	-1.02
ILMN_2038949	Susd1_predicted	1.04	-1.1	-1.04	-1.14	-1.19	-1	-1.02
ILMN_1362217	Ppp2r2a	-1.03	1.21	1.24	1.35	-1.02	1.01	-1.02
ILMN_1650282	RGD1309472	1.19	1.14	1.26	1.04	-1.21	1.02	-1.02
ILMN_1370390	Nme2	1.09	-1.08	1.02	1.11	-1.01	1.02	-1.02
ILMN_1366271	LOC287132	-1.07	-1.5	-1.37	-1.22	1.02	1.02	-1.02
ILMN_1366092	Itgav_predicted	1.13	1.14	-1.26	1.08	-1.22	1.03	-1.02
ILMN_1356846	LOC310958	1.17	1.42	1.26	1.33	1.03	1.03	-1.02
ILMN_1370613	Zbtb7a	-1.12	1.46	1.18	1.32	1.06	1.04	-1.02
ILMN_1350499	LOC365426	-1.05	-1.08	1.18	-1.18	1.09	1.04	-1.02
ILMN_1357855	Pcgf2_predicted	1.09	1.44	-1.09	1.34	1.1	1.04	-1.02
ILMN_1349809	Mettl7a	-1.15	-1.14	-1.31	-1.94	-1.03	1.05	-1.02
ILMN_1354930	Mettl7a	-1.15	-1.14	-1.31	-1.94	-1.03	1.05	-1.02

ILMN_1374995	Srp19_predicted	-1.12	-1.27	-1.42	-1.25	1.05	1.06	-1.02
ILMN_1371107	Ncln	1.09	1.39	1.7	1.37	-1.06	1.07	-1.02
ILMN_1357071	Unc45a	-1.1	1	1.16	1.16	1.02	1.07	-1.02
ILMN_1373580	Dhx57	-1.04	1.02	1.18	1.08	-1.18	1.08	-1.02
ILMN_1353897	Pde7a	1.09	1.16	1.35	1.33	1.1	1.08	-1.02
ILMN_1360205	Pkig	-1.08	1.13	-1.01	1.03	1.21	1.08	-1.02
ILMN_1374944	Dock6_predicted	-1.11	1.27	1.13	-1.17	1.08	1.09	-1.02
ILMN_1353448	LOC362725	-1	-1.03	1.07	1.18	1.12	1.09	-1.02
ILMN_1362490	LOC499724	-1.16	-1.16	-1.14	-1.11	1.24	1.09	-1.02
ILMN_1353046	Exoc5	1.2	1.1	1.26	-1.06	-1.02	1.1	-1.02
ILMN_1357886	Mrpl16	-1.07	1	1.11	1.15	1.05	1.1	-1.02
ILMN_1367373	Dnajc10	-1.11	-1.49	-1.37	-1.4	1.36	1.11	-1.02
ILMN_1374230	Cdc42se1	-1.18	-1.16	-1.15	-1.09	-1.07	1.12	-1.02
ILMN_1352678	LOC499255	-1.04	-1.02	-1.03	1.24	-1.02	1.12	-1.02
ILMN_1361761	Cops8	1.06	1.04	1.18	1.11	-1.01	1.13	-1.02
ILMN_1376559	Snx7	-1.03	-1.23	-1.12	1.07	1.01	1.13	-1.02
ILMN_1365596	Cdc16	-1.01	1.15	1.2	1.24	1.07	1.13	-1.02
ILMN_2039356	Cdc16	-1.01	1.15	1.2	1.24	1.07	1.13	-1.02
ILMN_1650283	RGD1307047	1.08	1.33	1.27	1.28	-1.22	1.14	-1.02
ILMN_1368421	RGD1306556_predicted	1.03	-1.07	-1.37	1.05	1.04	1.15	-1.02
ILMN_1376832	LOC245960	-1.08	1.18	1.22	1.23	1.2	1.15	-1.02
ILMN_1358092	Slc5a6	1.08	1.32	1.12	1.26	1.06	1.17	-1.02
ILMN_1368937	Cops4	1.04	1.11	1.26	-1.06	1.08	1.17	-1.02
ILMN_1354105	RGD1565775	-1.26	1.03	-1.02	1.2	1.13	1.17	-1.02
ILMN_1369892	E2f6	1.01	1.01	1.13	1.17	-1.09	1.18	-1.02
ILMN_1356068	RGD1306932	1.13	1.49	1.32	1.4	1.13	1.18	-1.02
ILMN_1650863	LOC501610	1.17	-1.08	-1.15	-1.19	1.18	1.19	-1.02
ILMN_1360673	Usp16	1.01	1.06	1.1	1.09	1.25	1.19	-1.02
ILMN_1373990	Usp16	1.01	1.06	1.1	1.09	1.25	1.19	-1.02
ILMN_1357572	Sub1	1.12	-1.73	-1.28	-1.02	1.28	1.19	-1.02
ILMN_1352338	Ddr1	1.15	1.18	1.35	1.15	-1.02	1.21	-1.02
ILMN_1359849	LOC308320	-1.03	-1.08	-1.18	-1.24	1.01	1.22	-1.02
ILMN_1354703	LOC684355	1.03	1.08	1.14	1.29	1.2	1.31	-1.02
ILMN_1367327	LOC299827	-1.29	-1.02	-1.01	1.06	1.27	1.32	-1.02
ILMN_1350494	Ubc	-1.12	-1.22	1.09	-1.51	1.18	1.38	-1.02
ILMN_1359863	Ythdf1	-1.01	1.1	1.02	1.06	1.65	1.59	-1.02
ILMN_1358642	RGD1566310_predicted	-1.03	-1.03	-1.46	-1.13	-1.39	-1.69	-1.01
ILMN_1351186	Ephx1	1.15	-1.43	-1.91	-1.92	-1.84	-1.55	-1.01

ILMN_1365130	Stk16	-1.23	-1.15	-1.14	-1.28	-1.52	-1.51	-1.01
ILMN_1358065	Mrpl51_predicted	1.05	-1.35	-1.47	-1.18	-1.41	-1.45	-1.01
ILMN_1363700	Mrpl40	-1	-1.08	-1.35	1.03	-1.25	-1.35	-1.01
ILMN_1366537	Atpbd1c	1.11	-1.21	1.47	1.05	-1.41	-1.34	-1.01
ILMN_1361402	Dci	-1.2	-1.42	-2.12	-2.07	-1.04	-1.34	-1.01
ILMN_1362979	Wdr68_predicted	1.04	1.05	-1.06	-1.26	-1.04	-1.29	-1.01
ILMN_1354116	LOC301444	1.1	-1.31	-2.31	-1.28	-1.03	-1.28	-1.01
ILMN_1360753	Umps	1.09	1.19	1.71	1.29	-1.23	-1.27	-1.01
ILMN_1368846	Pacsin2	1.12	-1.06	1.37	1.02	-1.07	-1.27	-1.01
ILMN_1365704	TSEN34	-1.18	-1.46	-1.41	-1.41	-1.19	-1.25	-1.01
ILMN_1369112	RGD1563669_predicted	-1.07	-1.2	-1.14	-1.18	-1.18	-1.24	-1.01
ILMN_1368770	Hemk2_predicted	-1.08	-1.25	-1.21	-1.36	-1.19	-1.23	-1.01
ILMN_1369920	Osgep	1.09	1.06	1.03	-1.27	-1.13	-1.23	-1.01
ILMN_1353760	RGD1309585_predicted	-1.01	-1.3	-1.53	-1.09	-1.23	-1.22	-1.01
ILMN_1365239	Tmem55a	-1.1	-1.05	1.05	-1.09	-1.16	-1.22	-1.01
ILMN_1376695	Rdbp	-1.08	-1.12	-1.02	-1.06	-1.13	-1.22	-1.01
ILMN_1372450	RGD1565344_predicted	1.11	-1.39	-1.15	-1.62	-1.14	-1.21	-1.01
ILMN_1358786	Xpc_predicted	-1.11	-1.11	-1.09	-1.21	-1.3	-1.2	-1.01
ILMN_1365798	Wsb2	-1.16	-1.45	-1.1	-1.21	-1.23	-1.17	-1.01
ILMN_1650378	Rbm34	1.05	1.12	1.32	1.2	-1.18	-1.17	-1.01
ILMN_1374752	LOC305583	-1.04	1.02	1.16	-1.08	-1.15	-1.17	-1.01
ILMN_1373269	Exoc3	-1.17	-1.22	-1.15	-1.21	-1.23	-1.16	-1.01
ILMN_1366965	RGD1306819	1.15	-1.29	1.09	-1.07	-1.08	-1.16	-1.01
ILMN_1366115	Cd151	1.14	-1.19	1.06	1.11	-1.04	-1.16	-1.01
ILMN_1362759	Med6_predicted	-1.15	-1.44	-1.36	-1.35	-1.03	-1.16	-1.01
ILMN_2039276	Med6_predicted	-1.15	-1.44	-1.36	-1.35	-1.03	-1.16	-1.01
ILMN_1354317	Psme2	-1.09	-1.2	-1.23	-1.17	-1.14	-1.15	-1.01
ILMN_1354016	Psmc5	1.07	1.08	1.11	1.09	-1.12	-1.15	-1.01
ILMN_1651158	Sdccag3	-1.01	-1.13	1.33	-1.06	-1.23	-1.14	-1.01
ILMN_1363852	Atp5d	1.02	-1.19	-1.44	-1.24	-1.13	-1.14	-1.01
ILMN_1371300	Hebp2_predicted	1.05	-1.27	-1.34	-1.7	-1.09	-1.14	-1.01
ILMN_1358044	Dnajc7	1.15	-1.06	1.07	1	-1.17	-1.13	-1.01
ILMN_1376597	Fbxo7	-1.14	-1.2	1.03	-1.14	-1.08	-1.13	-1.01
ILMN_1368461	RGD1309971_predicted	1.02	1.32	1.14	1.37	-1.03	-1.13	-1.01
ILMN_1353060	RGD1359242	-1.1	-1.16	-1.14	-1.11	-1.47	-1.12	-1.01
ILMN_1352849	Hif1an_predicted	-1.01	-1.22	-1.15	-1.28	-1.2	-1.12	-1.01
ILMN_1350795	RGD1562801_predicted	-1.13	-1.08	1.1	1.07	-1.12	-1.12	-1.01
ILMN_1374017	RGD1307150_predicted	1.14	-1.04	-1.65	-1.37	-1.08	-1.12	-1.01

ILMN_1357519	Ndufs5b	1.07	-1.15	-2.52	-1.33	-1.04	-1.11	-1.01
ILMN_1374584	LOC689484	1.06	-1.72	-1.9	-2.03	-1.19	-1.1	-1.01
ILMN_1372006	Ndufa13_predicted	1.06	1.15	-1.49	1.07	-1.03	-1.1	-1.01
ILMN_1354218	RGD1305721_predicted	-1.1	1.18	-1.04	-1.09	-1.4	-1.09	-1.01
ILMN_1371035	RGD1306106	1.08	1.12	1.06	1.06	-1.12	-1.09	-1.01
ILMN_1349909	LOC501659	1.08	-1.18	1.23	1.09	-1.14	-1.08	-1.01
ILMN_1358234	RGD1562351_predicted	-1	-1.25	-1.17	-1.33	-1.09	-1.07	-1.01
ILMN_1350976	Arpc3_predicted	-1.05	-1.28	-1.55	-1.03	1.04	-1.07	-1.01
ILMN_1366365	Epb4.1l4a_predicted	1.11	-1.19	1.03	1.15	1.04	-1.07	-1.01
ILMN_1349574	Pib5pa	-1.07	1.33	1.2	-1.04	-1.1	-1.06	-1.01
ILMN_1372965	Snrpb2_predicted	-1.06	1.11	-1.04	1.03	1.05	-1.06	-1.01
ILMN_1360326	Foxp4_predicted	-1.08	1.22	-1.2	1.08	1.05	-1.06	-1.01
ILMN_1367456	C1qbp	1.1	1.24	1.36	1.13	-1.2	-1.05	-1.01
ILMN_1372466	Zcchc9	-1.06	1.17	1.15	1.19	-1.08	-1.05	-1.01
ILMN_1354954	RGD1305045_predicted	-1.03	-1.04	1.03	-1.2	-1.06	-1.05	-1.01
ILMN_1352580	Gsr	1.41	-1.33	-1.01	-1.25	-1.25	-1.03	-1.01
ILMN_1359372	Polr2b_predicted	-1.12	-1.04	-1.17	1.14	-1.08	-1.03	-1.01
ILMN_1363641	Fuk_predicted	-1.05	1.12	1.11	1.1	1	-1.03	-1.01
ILMN_1351504	Fuk_predicted	-1.05	1.12	1.11	1.1	1	-1.03	-1.01
ILMN_1365343	Baiap2	-1.12	1.47	1.42	1.54	1.05	-1.03	-1.01
ILMN_1374510	RGD1359616	1.11	-1.17	1.01	1.04	-1.05	-1.02	-1.01
ILMN_1650024	Qrsl1	1.11	1.15	1.22	1.27	1.08	-1.01	-1.01
ILMN_1650295	Pik3r2	-1.12	1.06	1.09	-1.04	1.2	-1.01	-1.01
ILMN_1369836	Dnajc2	1.2	1.31	1.65	1.5	-1.15	-1	-1.01
ILMN_1353935	Farp1_predicted	-1.06	1.07	1.04	1.13	-1	-1	-1.01
ILMN_1353108	Psmd2	1.02	-1.11	-1.17	-1.06	-1.04	1	-1.01
ILMN_1354145	Armc8_predicted	-1.07	1.08	1.12	1.16	1.04	1	-1.01
ILMN_1349054	LOC308503	1.06	1.01	-1.37	1.07	-1.1	1.01	-1.01
ILMN_1364426	Gtf2h3	-1.08	1.03	1	-1.2	1.01	1.01	-1.01
ILMN_1364598	RGD1308326_predicted	1.09	1.1	1.16	1.22	1.38	1.01	-1.01
ILMN_1363343	Hspbp1	-1.08	1.02	1.09	-1	-1.04	1.02	-1.01
ILMN_1366495	Psmb7	1.17	-1	-1.01	1.07	1.02	1.02	-1.01
ILMN_1363975	LOC690364	-1.03	-1.03	-1.06	1.04	1.03	1.03	-1.01
ILMN_1374870	Pafah1b1	-1.1	-1.24	-1.01	1.13	1.11	1.03	-1.01
ILMN_1369002	RGD1565238_predicted	-1.04	-1.27	-1.29	-1	1.25	1.03	-1.01
ILMN_1362539	Rtcd1	-1.09	1.01	1.17	1.09	1.09	1.04	-1.01
ILMN_1349802	MGC125002	1.01	-1.09	1.04	1.01	1.02	1.05	-1.01
ILMN_1359857	Abca8b_predicted	-1.03	-1	1.03	-1.06	1.2	1.05	-1.01

ILMN_1350908	RGD1305350_predicted	1.06	1.05	1.12	1.28	1.09	1.06	-1.01
ILMN_1354928	Sfrs3_predicted	-1.19	-1.15	-1.14	-1.3	1.15	1.07	-1.01
ILMN_1354677	Nt5c2_predicted	-1.06	-1.02	1.07	-1.35	1.05	1.09	-1.01
ILMN_1368183	Larp1_predicted	1.01	1.06	-1.01	1.23	-1	1.11	-1.01
ILMN_1352750	Ccpg1_predicted	-1.09	-1.03	1.02	-1.43	1.02	1.11	-1.01
ILMN_1371005	Ccpg1_predicted	-1.09	-1.03	1.02	-1.43	1.02	1.11	-1.01
ILMN_1368996	Garnl1	1.15	1.24	1.42	1.16	1.05	1.12	-1.01
ILMN_1361843	RGD1309266_predicted	1.13	1.32	-1.07	1.15	1.1	1.12	-1.01
ILMN_1363818	Ssr2_predicted	1.11	1.11	1.24	1.18	1.31	1.12	-1.01
ILMN_1356684	Dnaja3	1.11	1.17	1.59	1.17	-1.02	1.13	-1.01
ILMN_1357907	Tgoln2	1.33	1.05	1.19	1.01	-1.02	1.15	-1.01
ILMN_1363936	Fem1a	-1.07	1.17	1.1	1.12	1.08	1.15	-1.01
ILMN_1350278	RGD1560268_predicted	-1.09	1.21	-1.11	-1.14	1.05	1.16	-1.01
ILMN_1350092	Dcun1d1_predicted	1.02	1.17	1.3	1.1	1.07	1.16	-1.01
ILMN_1364363	Rrbp1_predicted	-1.12	1.23	1.05	1.24	1.27	1.16	-1.01
ILMN_1363663	Dbt	1.08	1.16	1.4	1.12	-1.06	1.18	-1.01
ILMN_1359229	Ate1_predicted	1.15	1.19	-1.1	1.27	1.06	1.18	-1.01
ILMN_1367081	Aebp2_predicted	1.01	1.16	1.04	1.39	1.06	1.18	-1.01
ILMN_1353223	Nudt19	-1.01	1.04	-1.04	1.11	1.11	1.19	-1.01
ILMN_1352048	RGD1308601_predicted	-1.02	1.11	-1.05	1.21	1.03	1.2	-1.01
ILMN_1350074	RGD1307879_predicted	1.06	-1.06	1.34	1.09	1.04	1.2	-1.01
ILMN_1368704	LOC361418	-1	1.07	1.11	1.08	-1.09	1.21	-1.01
ILMN_1376712	Cox4nb	-1.08	-1.01	1.06	1.27	1.12	1.21	-1.01
ILMN_1356575	Ptpn21	-1.09	1.13	-1.1	1.09	1.11	1.25	-1.01
ILMN_1359288	RGD1565619_predicted	-1.02	1.15	1.19	-1.11	1.2	1.25	-1.01
ILMN_1352801	LOC301119	1.01	1.6	1.42	1.73	1.11	1.26	-1.01
ILMN_1350927	RGD1307672	-1.14	1.19	1.12	1.18	-1.04	1.3	-1.01
ILMN_1355486	Rbm13	1.05	1.23	1.51	1.4	1.38	1.33	-1.01
ILMN_1350619	Pdia5	-1.05	-1.01	1.27	1.33	1.64	1.34	-1.01
ILMN_1371674	Zfp354a	1.01	1.43	1.42	1.58	1.51	1.4	-1.01
ILMN_1372729	RGD1559449_predicted	1.01	-1.35	-1.66	-1.8	1.13	1.41	-1.01
ILMN_1373397	Epha2_predicted	-1.02	1.64	1.5	1.71	1.89	1.81	-1.01
ILMN_1370678	Slc20a2	1.07	-1.34	-1.2	-1.26	-2.02	-2.2	-1
ILMN_1358068	Xpo5_predicted	1.19	1.35	1.54	1.53	-1.52	-1.55	-1
ILMN_1352737	Vrk2_predicted	1	1.13	1.17	1.17	-1.11	-1.26	-1
ILMN_1373054	Taz_mapped	-1.05	1	1.2	-1.16	-1.03	-1.19	-1
ILMN_1374853	Dhx37_predicted	1.22	1.28	1.13	1.22	-1.12	-1.18	-1
ILMN_1352847	Tufm_predicted	-1.09	-1.13	1.13	-1.04	-1.12	-1.17	-1

ILMN_1373528	Echdc1	-1.03	-1.02	1.27	1.1	-1.2	-1.16	-1
ILMN_1357511	LOC500084	1.04	1.01	-1.03	-1.1	1.03	-1.15	-1
ILMN_1366596	LOC296462	-1.12	1.01	1.24	1.04	-1.04	-1.11	-1
ILMN_1361692	Ppp1r7	-1.15	-1.25	1.01	-1.14	-1.14	-1.09	-1
ILMN_1365101	Dars	-1.02	1.05	1.11	-1.12	-1.05	-1.09	-1
ILMN_1374563	Urod	-1.01	-1.13	-1.17	-1.25	1.04	-1.09	-1
ILMN_1369991	Appbp1	1.03	1.23	1.51	1.47	-1.1	-1.07	-1
ILMN_1650371	Stk38	1.03	-1.06	1.3	-1.15	-1.24	-1.06	-1
ILMN_1372008	Wdr5b	-1.02	-1.2	1.01	-1.09	1.04	-1.06	-1
ILMN_1350981	Ripk1_predicted	-1	-1.16	-1.31	-1.07	1.13	-1.05	-1
ILMN_1374753	RGD1561254_predicted	1.19	1.2	1.58	1.27	1	-1.04	-1
ILMN_1356070	RGD1564801_predicted	-1.03	-1.01	-1.04	-1.07	1.04	-1.04	-1
ILMN_1358815	Pccb	-1.04	-1.05	-1.14	-1.28	1.05	1.01	-1
ILMN_1361656	Rpl29	1.02	1.02	-1.1	1.02	1.02	1.03	-1
ILMN_1353269	RGD1562690_predicted	-1.09	-1.2	1.11	1.07	1.09	1.03	-1
ILMN_1359633	Dctn4	-1.04	1.35	1.28	1.06	-1.05	1.05	-1
ILMN_1367422	RGD1563580_predicted	1	1.14	-1.21	-1.1	1.3	1.05	-1
ILMN_1364720	Wbp4	1.01	1.59	1.23	1.42	1.03	1.06	-1
ILMN_1363549	Stk11ip_predicted	-1.13	1.13	1.15	-1.03	1.07	1.08	-1
ILMN_1355555	LOC361420	1.03	1.64	1.73	1.75	-1.06	1.1	-1
ILMN_1373831	Cstf3_predicted	1.18	1.07	1.14	1.06	1.06	1.1	-1
ILMN_1363778	RGD1304885_predicted	1.14	1.08	1.03	-1.12	1.09	1.1	-1
ILMN_1376475	Fts	1.05	-1.04	1.13	-1.04	1.14	1.1	-1
ILMN_1650013	Tiprl_predicted	1.16	-1.05	1.09	-1.11	1.09	1.11	-1
ILMN_1372137	Dyrk1a	1.03	1.12	-1.02	1.29	1.1	1.13	-1
ILMN_1359462	RGD621098	-1.02	-1.18	-1.15	-1.24	-1.04	1.16	-1
ILMN_1362396	RGD1559516_predicted	1.11	1.05	1.3	1.23	1.11	1.17	-1
ILMN_1368070	RGD1565589_predicted	1.04	-1.01	1.07	1.17	1.3	1.23	-1
ILMN_1359545	Ubb	-1.08	-1.08	1.13	-1.26	1.17	1.3	-1
ILMN_1354280	Smad4	1.07	1.35	1.14	1.02	1.08	1.32	-1
ILMN_1371050	MGC112775	1.07	1.5	1.27	1.41	1.63	1.45	-1
ILMN_1366410	Hbp1	1.16	-1.39	-1.43	-2.53	1.08	1.47	-1
ILMN_1360838	Dmpk_predicted	-1.17	1.05	-1.05	1.08	1.41	1.49	-1
ILMN_1368999	RGD1306001_predicted	1.14	1.21	1.21	1.09	-1.36	-1.37	1
ILMN_1354616	RGD1565784_predicted	1.17	-1.17	-1.14	1.12	-1.34	-1.37	1
ILMN_1369735	Odc1	1	-1.32	1.01	-1.03	-1.32	-1.21	1
ILMN_1356126	Got1	1.17	1.29	1.29	1.16	-1.07	-1.21	1
ILMN_1358633	Atpaf2_predicted	-1.06	1.01	1.14	1.12	-1.16	-1.19	1

ILMN_1370750	Cib1	-1.18	-1.16	-1.38	-1.22	-1.07	-1.19	1
ILMN_1362559	RGD1311164_predicted	-1.16	-1.15	-1.18	-1.16	-1.48	-1.16	1
ILMN_1361160	Gna11	-1.03	-1.12	-1.17	1.05	-1.05	-1.16	1
ILMN_1365749	RGD1566149_predicted	1.02	1.08	-1.21	-1.37	-1.08	-1.14	1
ILMN_1649860	LOC303067	-1.27	-1.1	1.09	1	-1.1	-1.13	1
ILMN_1651012	LOC692000	1.11	-1.23	-2.96	-1.22	-1.11	-1.12	1
ILMN_1349530	Crsp6	-1.11	1.19	-1.02	1.08	-1.02	-1.12	1
ILMN_1374957	Mtfmt	1.07	-1.04	1.07	1.16	-1.02	-1.12	1
ILMN_1358538	Card12_predicted	1.06	1.18	-1.33	1.03	1	-1.11	1
ILMN_1376400	Htf9c	-1.12	1.09	-1.03	1.39	-1.25	-1.1	1
ILMN_1374043	Eraf_predicted	-1.13	-1.29	-1.39	-1.3	-1.19	-1.09	1
ILMN_1373476	RGD1307254	-1.08	-1.16	-1.15	-1.01	-1.08	-1.09	1
ILMN_1375291	Tbrg4_predicted	1.13	1.13	1.56	1.12	-1.21	-1.08	1
ILMN_1355482	LOC361026	-1.02	1.03	1.05	1.07	1	-1.08	1
ILMN_1376373	Plscr3	-1.02	1.04	-1.08	1.13	1.06	-1.06	1
ILMN_1361838	Pdlim1	-1	1.06	-1.18	1.23	1.02	-1.04	1
ILMN_1360689	Ube2g1	-1.08	-1.06	-1.07	-1.04	1.01	-1.02	1
ILMN_1355444	RGD1309571	1.03	-1.1	1.07	-1.11	-1.09	1.01	1
ILMN_1352149	LOC362012	-1.16	-1	-1.27	1.02	-1.05	1.02	1
ILMN_1374562	Fam38a_predicted	1.22	1.73	1.46	1.76	1.02	1.02	1
ILMN_1373904	Cox5b	1.13	-1.22	-1.78	-1.03	1.04	1.02	1
ILMN_1368903	Rpl31	1.04	-1.09	1.39	1.1	1.11	1.02	1
ILMN_1349457	LOC502770	-1.02	-1.34	-1.22	-1.09	1.15	1.02	1
ILMN_1376709	Ceecam1	1.13	1.02	1.12	-1	1.31	1.02	1
ILMN_1365807	Mrps2_predicted	1.15	1.42	-1.01	1.54	1.1	1.04	1
ILMN_1367545	Rnf19_predicted	-1.2	-1.18	-1.41	-1.32	-1.04	1.06	1
ILMN_1369663	Usp10	1.03	1.28	1.07	1.32	1.16	1.08	1
ILMN_1369686	Vps37b_predicted	1.01	1.25	-1.02	1.11	1.24	1.09	1
ILMN_1374962	Tcf8	-1.03	1.07	1.17	1.01	1.23	1.1	1
ILMN_1359869	Brd1_predicted	-1.01	1.1	1.14	-1.08	1.03	1.11	1
ILMN_1350869	Sugt1	-1.12	1.21	1.06	1.17	1.27	1.11	1
ILMN_1373698	Med8_predicted	-1.07	1.33	1.41	1.39	1.09	1.13	1
ILMN_1369543	Son	-1.13	1.09	1.01	-1.02	1.01	1.15	1
ILMN_1363341	Cbfa2t2_predicted	1.02	1.36	1.14	1.17	1.18	1.15	1
ILMN_1352610	RGD1306356	-1.18	-1.19	-1.11	-1.17	-1	1.16	1
ILMN_1361978	Crk	1.05	1.25	1.1	1.39	-1.01	1.17	1
ILMN_1650950	Josd3	1.07	1.03	1.18	1.18	1.12	1.19	1
ILMN_1375232	LOC309478	-1.07	-1.07	-1.15	-1.11	1.14	1.2	1

ILMN_1370257	Mgea5	1.19	1.11	1.22	-1.04	-1	1.27	1
ILMN_1373520	Pscd2	-1.25	1.39	1.28	1.44	1.28	1.32	1
ILMN_1361539	Rapgef2_predicted	1.08	-1.11	-1.5	-1.03	1.23	1.34	1
ILMN_1362702	Gmfg	-1.07	1.17	1.08	1.35	1.41	1.38	1
ILMN_1359893	Gsta2	1.37	-1.51	-2.15	-2.08	-1.9	-2.09	1.01
ILMN_1374974	Ndufaf1_predicted	-1.12	-1.02	-1.18	-1.04	-1.34	-1.5	1.01
ILMN_1362281	RGD1311078_predicted	1.19	-1.12	-1.4	-1.17	-1.07	-1.45	1.01
ILMN_1370214	Abcb4	-1.12	-1.02	-1.15	-1.2	-1.21	-1.35	1.01
ILMN_1353675	RGD1560015_predicted	-1.04	-1.58	-1.15	-1.44	-1.34	-1.34	1.01
ILMN_1359779	Ppp1r11	-1.07	-1.09	-1.37	-1.27	-1.18	-1.34	1.01
ILMN_1354221	RGD1305824_predicted	-1.08	-1.81	-1.85	-1.72	-1.24	-1.33	1.01
ILMN_1352426	Snapc1_predicted	-1.17	1.07	1	1.18	-1.27	-1.31	1.01
ILMN_1349855	Gm2a	-1.06	-1.19	-1.1	-1.13	-1.13	-1.3	1.01
ILMN_1350911	Cd2bp2_predicted	-1.11	-1.53	-1.49	-1.28	-1.15	-1.27	1.01
ILMN_1358213	Atp5c1	1.02	-1.07	-1.13	-1.25	-1.14	-1.26	1.01
ILMN_1376816	Nras	-1.02	-1.03	-1.17	-1.05	-1.37	-1.2	1.01
ILMN_1350280	Dnm2	-1.1	-1.22	-1.16	-1.26	-1.13	-1.2	1.01
ILMN_1367262	Fsip1	-1.17	-1.18	-1.23	-1.05	-1.12	-1.18	1.01
ILMN_1365479	Sirt7_predicted	-1.03	-1.02	1.2	-1.01	-1.16	-1.17	1.01
ILMN_1371101	RGD1307325	1.02	-1.35	-1.49	-1.91	-1.23	-1.15	1.01
ILMN_1351493	Ncoa6ip_predicted	-1.06	1.01	-1.03	1.21	-1.08	-1.15	1.01
ILMN_1364660	Ss18	-1	-1.07	1.09	-1.09	-1.15	-1.14	1.01
ILMN_1360301	LOC497693	-1.08	-1.25	-1.21	-1.4	-1.14	-1.14	1.01
ILMN_1370385	RGD1310143	-1.02	1.1	1.01	-1.09	-1.05	-1.13	1.01
ILMN_1354755	Centa2	1.24	1.19	-1.05	-1.04	1.02	-1.13	1.01
ILMN_1369256	Map2k4_predicted	-1.12	-1.08	-1	-1.04	1.02	-1.13	1.01
ILMN_1353633	Ak3	1.04	-1.2	-1.38	-1.45	-1.24	-1.12	1.01
ILMN_1372482	Bbs2	-1.03	-1.01	-1.17	-1.15	-1.18	-1.1	1.01
ILMN_1362270	LOC688869	1.01	-1.11	-2.31	-1.16	-1.14	-1.1	1.01
ILMN_1368232	Abtb1	-1.13	-1.23	-1.59	-1.83	-1.05	-1.1	1.01
ILMN_1366213	Slc25a16	-1.11	-1.15	-1.37	-1.26	-1.09	-1.09	1.01
ILMN_1359934	Armc9_predicted	-1.06	1.17	-1.05	1.17	-1.13	-1.08	1.01
ILMN_1650108	LOC500483	1.16	1.5	-1.01	1.42	-1	-1.08	1.01
ILMN_1352745	LOC682182	1.13	1.18	1.32	1.28	1.08	-1.08	1.01
ILMN_1361626	Hnrpa3	-1.06	-1.54	-1.28	-1.45	1.18	-1.08	1.01
ILMN_1361380	LOC304035	-1.04	-1.01	1.06	-1.04	-1.01	-1.07	1.01
ILMN_1350104	RGD1305274_predicted	-1.03	-1.07	-1.08	-1.01	-1.3	-1.06	1.01
ILMN_1650777	Rpl7a_predicted	-1.01	1.07	1.07	1.08	-1.09	-1.06	1.01

ILMN_1349482	LOC313067	-1.03	1.08	-1.09	-1	-1.29	-1.05	1.01
ILMN_1352563	LOC298536	-1.05	-1.19	-1.01	-1.33	-1.17	-1.04	1.01
ILMN_1370825	Eif4e2_predicted	-1.09	-1.05	1.22	1.17	-1.11	-1.04	1.01
ILMN_1376418	Ahi1	1.05	1.3	1.2	1.04	-1.07	-1.04	1.01
ILMN_1368495	Map4k3	-1.15	1.04	-1.03	1.01	-1.03	-1.04	1.01
ILMN_1357992	RGD1565159_predicted	-1.02	-1.18	-1.01	-1.05	-1	-1.03	1.01
ILMN_1358957	LOC291773	1.15	1.09	1.23	1.04	1.05	-1.03	1.01
ILMN_1358042	Rbm17	1.2	1.14	1.14	1.16	-1.12	-1.02	1.01
ILMN_1650246	RGD1561600_predicted	1.03	1.22	-1.09	1.13	1.01	-1.02	1.01
ILMN_1366901	Psma4	-1.05	-1.02	1.06	1.07	1.08	-1.02	1.01
ILMN_1355963	Ube2l3_predicted	-1.01	-1.27	-1.11	-1.14	1.1	-1.02	1.01
ILMN_1650549	Eif2s3x	1.05	-1.01	1.03	1.19	1.12	-1.02	1.01
ILMN_1360139	Ndufa5	1.09	-1.14	-2.08	-1.27	-1.02	-1.01	1.01
ILMN_2038795	Rps9	1.04	1.02	-1.19	-1.03	-1.03	1.01	1.01
ILMN_2038796	Rps9	1.04	1.02	-1.19	-1.03	-1.03	1.01	1.01
ILMN_1369884	Rpl23a	1.05	1.14	-1.07	1.11	1.06	1.01	1.01
ILMN_1368524	Glg1	-1.08	1.01	1.02	-1.08	-1.07	1.03	1.01
ILMN_1365589	Lsg1	-1.04	-1.02	1.09	1.07	-1.05	1.03	1.01
ILMN_1358726	Pik3r4_predicted	1	-1.16	-1.15	-1.35	-1.07	1.04	1.01
ILMN_1351643	Tbn_predicted	1.08	1.07	-1.05	1.3	1.06	1.06	1.01
ILMN_1349240	Tloc1_predicted	-1.13	-1.02	-1.19	-1.25	1.09	1.06	1.01
ILMN_1354511	Slc4a2	1.15	1.65	-1.2	1.55	1.24	1.06	1.01
ILMN_1370633	Itpkc	-1.12	1.12	1.09	1.09	1.31	1.06	1.01
ILMN_1370742	RGD1560629_predicted	-1.04	1.02	1.17	-1	-1.16	1.07	1.01
ILMN_1356078	RGD1559704_predicted	1.08	-1.41	1.08	-1.03	-1	1.07	1.01
ILMN_1651174	RGD1565965_predicted	-1.02	-1.21	1.21	1.06	1.09	1.07	1.01
ILMN_1349461	Abcc5	-1.17	1.28	-1.36	-1.15	1	1.08	1.01
ILMN_1375218	Cdc37	1.11	1.06	1.21	1.23	1.03	1.08	1.01
ILMN_1355197	Fbxl11_predicted	1.08	-1.03	-1.32	-1.22	1.08	1.08	1.01
ILMN_1361913	RGD1308517	1.1	-1.15	-1.13	-1.29	1.11	1.08	1.01
ILMN_1363059	RGD1560797_predicted	1.05	-1.43	-1.23	-1.06	1.03	1.1	1.01
ILMN_1371229	Atxn2_predicted	-1.09	1.26	1.12	1.16	1.05	1.1	1.01
ILMN_1360762	Arf1	-1.1	-1.38	-1.08	-1.13	1.11	1.1	1.01
ILMN_1372761	LOC291411	1.02	1.01	1.27	-1.02	-1.07	1.11	1.01
ILMN_1375508	Stxbp5	-1.01	1.17	-1.01	1.08	-1.03	1.11	1.01
ILMN_1376791	Creb1	-1.03	1.19	1.08	1.33	1.01	1.12	1.01
ILMN_1649829	Creb1	-1.03	1.19	1.08	1.33	1.01	1.12	1.01
ILMN_1361977	LOC686212	-1	1.51	1.41	1.38	1.1	1.12	1.01

ILMN_1358274	Sf3b1	-1.25	-1.24	-1.06	-1.25	1.36	1.12	1.01
ILMN_1371437	Luc7l_predicted	1.11	1.12	-1.02	1.73	-1.12	1.13	1.01
ILMN_1366845	Auh_predicted	1.01	1.02	-1.23	-1.4	-1.03	1.13	1.01
ILMN_1361750	Hes1	1	1.06	1.32	-1	1.05	1.14	1.01
ILMN_1354153	Gatad2a	1.12	1.07	-1.31	1.12	1.09	1.14	1.01
ILMN_1350033	Net1	1.07	-1.07	1.38	-1.08	1.01	1.15	1.01
ILMN_1361932	Mll5	-1.11	1.05	-1.46	-1.38	1.09	1.15	1.01
ILMN_1354827	Dcun1d5	1.05	1.22	1.29	1.39	1.16	1.17	1.01
ILMN_1355480	Usp30_predicted	-1.27	1.02	-1.31	-1.1	1.13	1.18	1.01
ILMN_1368627	Psrc2	1	-1	1.44	1.32	-1.06	1.2	1.01
ILMN_1361299	Itgb1	1.1	-1.07	1.11	1.04	1.1	1.23	1.01
ILMN_1373016	Fgfr1op2	1.1	1.3	1.17	1.3	1.41	1.24	1.01
ILMN_1360657	Tmem30a	-1.06	1.11	1.19	1	1.25	1.27	1.01
ILMN_1372711	LOC502316	1.08	1.14	1.16	1.23	1.23	1.31	1.01
ILMN_1352533	Cdc2l5	-1.02	1.27	1.12	1.21	1.33	1.36	1.01
ILMN_1370220	Fads2	-1.01	-1.4	-1.18	-1.3	-1.27	-1.4	1.02
ILMN_1368612	Pdcd8	-1.26	-1.26	-1.16	-1.2	-1.22	-1.33	1.02
ILMN_1376522	B4galt6	-1.08	-1.48	-1.23	-1.33	-1.27	-1.31	1.02
ILMN_1364419	Cnp1	-1.07	-1.1	-1.21	-1.24	-1.16	-1.3	1.02
ILMN_1366325	Raver1h	-1.08	-1.35	-1.5	-1.06	-1.42	-1.28	1.02
ILMN_1360034	LOC500419	-1.17	-1.43	-1.15	-1.49	-1.07	-1.28	1.02
ILMN_1353803	Prkar2b	1.65	-1.17	1.12	-1.04	-1.35	-1.25	1.02
ILMN_1361331	Smarcc1_predicted	-1.19	1.07	-1.29	-1.02	-1.31	-1.23	1.02
ILMN_1353508	RGD1564623_predicted	1.05	1.23	1.27	1.06	-1.27	-1.23	1.02
ILMN_1349537	Tceal8	-1.23	-1.27	-1.32	-1.44	-1.1	-1.23	1.02
ILMN_1649759	RGD1311345	-1.02	-1.1	1.1	1.1	-1.19	-1.22	1.02
ILMN_1354019	Sardh	1.19	-1.07	1.09	1	-1.05	-1.17	1.02
ILMN_1366612	Mapk3	1.11	-1.13	1.69	-1.04	-1.04	-1.15	1.02
ILMN_1374981	Cbr4	-1.09	-1.4	-1.35	-1.8	-1.13	-1.14	1.02
ILMN_1352832	Slc39a13	-1.16	-1.2	-1.31	-1.3	-1.17	-1.13	1.02
ILMN_1362964	Mapre3	-1.05	-1.26	-1.16	-1.08	-1.13	-1.13	1.02
ILMN_1355718	Pomt1	-1.07	-1.2	1.08	-1.19	-1.19	-1.12	1.02
ILMN_1353274	Nedd8	1.17	-1.13	-1.28	-1.08	-1.1	-1.12	1.02
ILMN_1362105	Dstn_predicted	1.02	-1.07	1.07	1.04	-1.05	-1.12	1.02
ILMN_1349025	Znhit2_predicted	-1.17	1.14	1.12	1.19	-1.07	-1.11	1.02
ILMN_1368848	Mon1a_predicted	-1.11	-1.11	-1.27	-1.02	1.1	-1.11	1.02
ILMN_1364292	RGD1309437	1.24	1.14	1.28	-1	-1.25	-1.1	1.02
ILMN_1359578	RGD1305574_predicted	1.02	1.25	1.08	1.59	-1.02	-1.1	1.02

ILMN_1373988	Herc1_predicted	-1.06	1.01	-1.1	-1.16	-1.07	-1.09	1.02
ILMN_1369062	Pfkl	1.01	-1.1	-1.17	-1.03	1.13	-1.07	1.02
ILMN_1649868	RGD1566137_predicted	1.07	1.04	-1.22	1.01	-1.02	-1.04	1.02
ILMN_1350071	Pias4	1.06	1.15	1.05	1.12	1.2	-1.02	1.02
ILMN_1372650	Yipf4	1.01	-1.13	-1.14	-1.51	-1.05	-1.01	1.02
ILMN_1352873	Scand1_predicted	-1	1.13	-1.62	-1.09	1.01	-1.01	1.02
ILMN_1368568	Golga2	-1.07	-1.08	-1.08	-1.34	-1.11	-1	1.02
ILMN_1351926	Ppp3cb	1.11	1.08	1.18	-1.02	1.01	-1	1.02
ILMN_1358097	Ppp2ca	1.07	-1.02	1.17	1.02	1.05	-1	1.02
ILMN_1364741	Wdr61	-1.08	-1.07	1.13	1.09	1.07	-1	1.02
ILMN_1358395	LOC502063	-1.29	-1.09	1.12	1.13	1	1	1.02
ILMN_1349337	Pum2	-1.09	1.07	-1.05	-1.12	-1.08	1.01	1.02
ILMN_1365027	RGD1305225	-1.04	1.27	1.18	1.36	-1.04	1.01	1.02
ILMN_2039950	Eef1a2l1	1.01	-1.04	1.07	-1.03	-1.03	1.01	1.02
ILMN_2039949	Eef1a2l1	1.01	-1.04	1.07	-1.03	-1.03	1.01	1.02
ILMN_1365340	Tlk1_predicted	-1.01	1.03	-1.02	-1.17	-1.02	1.01	1.02
ILMN_1371379	Psmd5_predicted	1.03	-1.11	1.1	1.11	1.13	1.01	1.02
ILMN_1352293	Bmpr2	1.09	1.17	1.13	1.29	1.12	1.02	1.02
ILMN_1368861	LOC498138	1.07	1.16	-1.02	1.15	1.15	1.02	1.02
ILMN_1351721	RGD1311605_predicted	-1.18	-1.02	1.03	-1.29	1.01	1.03	1.02
ILMN_1352699	RGD1564725_predicted	1	1.12	1.39	1.29	1.04	1.03	1.02
ILMN_1369679	LOC682072	-1	-1.03	-1.08	1.05	-1.05	1.04	1.02
ILMN_1354663	Taok1	1.15	1.17	1.28	1.06	1.12	1.06	1.02
ILMN_1353763	LOC501203	1.13	-1.04	-1	1.22	1.02	1.07	1.02
ILMN_1371874	Txndc1	1.17	-1.25	1.42	-1.07	1.08	1.08	1.02
ILMN_1368391	Med19_predicted	-1.07	-1.19	-1.95	-1.1	1.16	1.09	1.02
ILMN_1374786	Slc1a4	1.15	1.3	1.05	1.25	1.16	1.09	1.02
ILMN_1358423	Gatad2b	1.08	1.5	1.17	1.27	-1.04	1.1	1.02
ILMN_1650000	LOC681542	-1.07	-1.08	-1.1	1.21	1.02	1.1	1.02
ILMN_1376869	Usp19	-1.24	1.01	1.06	-1	1.13	1.1	1.02
ILMN_1349231	Rbm25_predicted	-1.16	-1.08	1.01	1.1	1.29	1.1	1.02
ILMN_1350706	LOC295420	1.04	-1.4	-1.23	-1.08	1.35	1.1	1.02
ILMN_1353794	Trip12	1.01	1.18	1.05	1.06	1.1	1.12	1.02
ILMN_1351180	LOC367047	-1.05	1.09	1.81	1.18	1.1	1.12	1.02
ILMN_1361740	Chmp7_predicted	1.08	1.09	1.34	-1.07	1.15	1.12	1.02
ILMN_1374441	RGD1559951_predicted	1.05	1.14	-1.16	1.13	1.12	1.14	1.02
ILMN_1361794	LOC314323	1.04	-1.05	1.09	-1.2	1.18	1.14	1.02
ILMN_1352671	Nsun4_predicted	1.02	1.27	1.18	-1.16	-1	1.15	1.02

ILMN_1364948	Unc84a	1.21	1.31	1.7	1.31	1.11	1.16	1.02
ILMN_1352530	Aco2	-1.02	-1.21	-1.28	-1.28	1.09	1.17	1.02
ILMN_1363693	RGD1565049_predicted	-1.1	-1.09	-1.12	-1.24	1.15	1.17	1.02
ILMN_1355085	LOC296207	1.21	-1.06	1.04	1.07	1.24	1.17	1.02
ILMN_1349962	Wasf1	1.16	1.42	1.09	1.32	1.13	1.18	1.02
ILMN_1371119	Alkbh5_predicted	-1.06	-1.06	-1.02	-1.03	1.2	1.18	1.02
ILMN_1351992	Cd300lf	1.11	1.25	1.78	1.2	1.07	1.19	1.02
ILMN_1354319	Wdr32_predicted	-1.03	1.26	1.11	1	1.08	1.19	1.02
ILMN_1362586	Als2cr13_predicted	1.06	1.23	1.1	1.15	-1.1	1.21	1.02
ILMN_1350121	LOC497729	-1.12	1.12	-1.11	1.02	1.18	1.22	1.02
ILMN_1374214	Thoc1_predicted	1.06	-1.2	1.11	1.07	1.28	1.22	1.02
ILMN_1353409	Dpp8_predicted	1.13	1.22	1.19	1.23	1.21	1.24	1.02
ILMN_1358047	LOC683334	-1.15	-1.01	-1.06	1.08	1.2	1.27	1.02
ILMN_1350216	LOC498930	1.16	1.02	1.18	1.18	1.19	1.32	1.02
ILMN_1354719	Dapp1_predicted	1.25	1.77	1.12	1.02	1.12	1.34	1.02
ILMN_1373964	Nol10	-1.02	1.3	1.3	1.2	1.17	1.35	1.02
ILMN_1364833	LOC361237	1.16	1.31	1.58	1.42	1.41	1.4	1.02
ILMN_1367021	Papola_predicted	1.15	1.19	1.37	1.21	1.31	1.42	1.02
ILMN_1355975	Zfp330_predicted	1.08	1.32	1.36	1.46	1.68	1.45	1.02
ILMN_1350007	Dnajb11	1.03	-1.21	-1.05	-1.15	1.59	1.46	1.02
ILMN_1373927	LOC500033	-1.03	1.01	1.1	1.07	1.33	1.59	1.02
ILMN_1376644	Crygs	-1.05	-1.24	1.12	-1.26	1.74	1.63	1.02
ILMN_1350607	Siah2	1.12	1.29	1.25	1.41	2	2.05	1.02
ILMN_1359358	Smp2a	-1.46	-1.35	-1.32	-1.41	-1.07	-1.44	1.03
ILMN_1371416	Rag1ap1_predicted	1.04	1.09	1.41	1.09	-1.04	-1.43	1.03
ILMN_1352032	Decr2	-1.23	-1.25	-1.11	-1.53	-1.47	-1.4	1.03
ILMN_1372925	LOC498208	1.03	1.09	-1.11	-1.18	-1.32	-1.39	1.03
ILMN_1373864	RGD1308113	1.14	-1.06	-1.15	-1.1	-1.17	-1.39	1.03
ILMN_1354166	LOC687840	1.14	1.15	-1.16	-1.33	-1.26	-1.38	1.03
ILMN_1362844	Map2k2	-1.1	-1.01	1.08	-1.25	-1.31	-1.32	1.03
ILMN_1369297	Mrpl41	-1.02	-1.33	-1.66	-1	-1.17	-1.3	1.03
ILMN_1350639	Ddx56	1.06	1.14	1.35	1.39	-1.47	-1.29	1.03
ILMN_1362344	Ndnl2	-1.09	-1.19	-1.2	-1.08	-1.27	-1.26	1.03
ILMN_1368299	RGD1308012_predicted	-1.09	1.22	1.17	1.14	-1.25	-1.26	1.03
ILMN_1353037	LOC293589	1.07	1.15	1.13	-1.11	-1.27	-1.23	1.03
ILMN_1352785	LOC293589	1.07	1.15	1.13	-1.11	-1.27	-1.23	1.03
ILMN_1357172	RGD1308635_predicted	-1.01	1.01	-1.35	-1.01	-1.1	-1.23	1.03
ILMN_1372253	Ufd1l	-1.06	-1.18	-1.16	1	1.09	-1.23	1.03

ILMN_1368679	RGD1563208_predicted	-1.06	-1.17	-1.22	-1.07	-1.42	-1.22	1.03
ILMN_1363130	Udpgtr2	-1.02	-1.74	-1.64	-2.1	-1.21	-1.22	1.03
ILMN_1354502	Suox	1.05	-1.06	-1.33	-1.22	-1.17	-1.2	1.03
ILMN_1373565	Aph1a	-1.01	-1.1	-1.25	1.06	-1.15	-1.2	1.03
ILMN_1365472	RGD1307703_predicted	1.2	1.23	1.79	1.25	-1.09	-1.18	1.03
ILMN_1651079	LOC500436	-1.59	-1.38	-1.08	-1.06	-1.19	-1.17	1.03
ILMN_1357678	Sdha	-1.22	-1.09	-1.24	-1.23	-1.25	-1.16	1.03
ILMN_1365157	Mrpl43_predicted	-1.01	1.14	-1.43	1	-1.15	-1.15	1.03
ILMN_1357994	RGD1309759_predicted	-1.07	-1.41	-1.2	-1.46	-1.03	-1.15	1.03
ILMN_1369321	Aptx	-1.11	-1.11	-1.01	-1.05	-1.31	-1.14	1.03
ILMN_1360269	Sfrs5	-1.31	1.01	1.25	-1.04	-1.23	-1.13	1.03
ILMN_1353996	LOC309891	1.05	-1.05	1.31	-1.29	-1.11	-1.13	1.03
ILMN_1352110	LOC363018	1.12	1.1	1.1	1.44	-1.14	-1.11	1.03
ILMN_1349851	Cmpk	-1.17	-1.24	-1.17	-1.23	-1.08	-1.11	1.03
ILMN_1374006	RGD1310199	-1.27	-1.4	-1.18	-1.31	-1.07	-1.1	1.03
ILMN_1371256	Psma2	1.01	-1.11	-1.17	-1.12	-1.03	-1.09	1.03
ILMN_1650374	LOC498362	-1.06	-1.33	1.01	-1.13	-1.21	-1.08	1.03
ILMN_1358820	Ltbp3	-1.08	1.54	1.63	1.16	-1.09	-1.08	1.03
ILMN_1375146	RGD1307055_predicted	-1.21	1.13	-1	-1.04	-1.17	-1.07	1.03
ILMN_1366394	Timm13	1.04	1.04	-1.26	1.04	-1.21	-1.06	1.03
ILMN_1359614	Herc3_predicted	-1.01	-1.09	-1.22	-1.25	-1.11	-1.06	1.03
ILMN_1368004	RGD1563476_predicted	-1.02	-1	1.17	-1	-1.08	-1.06	1.03
ILMN_1367242	RGD1563476_predicted	-1.02	-1	1.17	-1	-1.08	-1.06	1.03
ILMN_1348958	Rab14	-1.1	-1.12	1.06	-1.01	-1.03	-1.06	1.03
ILMN_1363492	Gpam	1.12	1.08	-1.04	-1	-1.01	-1.06	1.03
ILMN_1368359	Rpl6	-1.02	1.02	1.14	1.03	-1.08	-1.05	1.03
ILMN_1370241	RGD1566355_predicted	-1.2	-1.09	1.03	-1.14	-1.07	-1.04	1.03
ILMN_1371952	Pddc1_predicted	1.02	1.09	1.37	-1.06	1.09	-1.04	1.03
ILMN_1376902	Acbd4	-1.01	1.11	1.22	1.1	1.09	-1.03	1.03
ILMN_1362874	Eif4g3_predicted	1.01	1.13	1.1	1.07	1.01	-1.02	1.03
ILMN_1363036	Pnpt1	-1.02	1.38	1.27	1.59	1.08	-1.02	1.03
ILMN_1372355	LOC315547	1.16	1.01	1.33	1.08	1.14	-1.02	1.03
ILMN_1351957	Me2_predicted	1.02	1.04	1.34	1.11	-1.06	-1.01	1.03
ILMN_1349742	Papolg_predicted	-1.08	-1.02	-1.16	-1.04	1	-1.01	1.03
ILMN_1366285	Cpsf3	1.08	1.09	1.37	1.17	-1.04	-1	1.03
ILMN_1359094	Yc2	1.25	-1.81	-1.32	-1.81	1.01	-1	1.03
ILMN_1357055	H2afx	-1.02	-1.12	1.19	-1.05	-1.02	1	1.03
ILMN_1374104	Ndufc2	-1.04	1.02	-1.46	-1.12	1.08	1	1.03

ILMN_1363698	Mrps17_predicted	-1.03	-1.16	-1.37	-1.15	-1.11	1.01	1.03
ILMN_1365670	Gpi	-1.04	-1.29	-1.09	-1.16	-1.02	1.02	1.03
ILMN_1367540	RGD1309779_predicted	1.2	1.25	1	1.22	-1.01	1.02	1.03
ILMN_1369965	Myh9	-1.12	-1.15	1.27	1.08	1.02	1.02	1.03
ILMN_1350384	Sp110	-1.04	1.54	1.35	1.22	1.1	1.02	1.03
ILMN_1372435	RGD1308695_predicted	1.03	1.08	1.02	1.19	1.12	1.02	1.03
ILMN_1376485	Ubtd1	-1.06	1.14	1.12	1.24	-1.07	1.03	1.03
ILMN_1375221	RGD1305692	-1.02	1.01	1.14	1.09	-1.01	1.03	1.03
ILMN_1371197	LOC364239	-1.02	1.18	1.13	1.17	1.04	1.03	1.03
ILMN_1366532	RGD1560831_predicted	-1.02	1.01	1.11	1.04	1.07	1.03	1.03
ILMN_1369694	Chd6_predicted	-1.15	-1.01	1.05	-1.11	1.1	1.03	1.03
ILMN_1348815	RGD1560796_predicted	1.26	1.32	1.11	1.16	1.14	1.03	1.03
ILMN_1376755	Snx17	-1.15	-1.02	-1.11	-1.09	-1.11	1.04	1.03
ILMN_1364451	Arhgap21_predicted	-1	1.13	-1.02	1.05	-1.06	1.05	1.03
ILMN_1364196	RGD1561459_predicted	-1.03	-1.08	-1.26	-1.16	1.1	1.05	1.03
ILMN_1353960	Idh1	-1.07	-1.71	-1.46	-3.11	-1.03	1.06	1.03
ILMN_1373242	LOC308053	1.02	1.3	-1.09	1.04	1.02	1.06	1.03
ILMN_1372305	Cox4i1	-1.03	-1.06	-1.25	-1.08	1.06	1.06	1.03
ILMN_1376466	Cutl1	-1	1.01	1.02	1.05	1.09	1.06	1.03
ILMN_1362265	Khdrbs1	-1.04	1.28	-1.09	1.02	1.1	1.06	1.03
ILMN_1351369	Phf22	-1.01	1.06	1.01	-1.13	-1.23	1.07	1.03
ILMN_1362978	Ptpre	1.03	1.17	-1.1	1.17	1.02	1.09	1.03
ILMN_1350749	Papss1_predicted	1	-1.04	-1.18	-1.18	1.17	1.09	1.03
ILMN_1372628	Papss1_predicted	1	-1.04	-1.18	-1.18	1.17	1.09	1.03
ILMN_1361133	Tsta3_predicted	1.05	1.19	1.29	-1.01	1.05	1.11	1.03
ILMN_1358610	RGD1311526_predicted	1.41	1.09	1.04	1.19	1.1	1.11	1.03
ILMN_1360025	Ddx41_predicted	-1.07	1.39	1.36	1.51	1.17	1.11	1.03
ILMN_1357456	RGD1561984_predicted	-1.01	1.25	-1.11	1.16	1.13	1.12	1.03
ILMN_1351837	RGD1308428_predicted	-1.01	-1.2	-1.14	-1.21	-1.08	1.14	1.03
ILMN_1349782	Rpl21	-1.03	1.08	-1.01	1.14	1.05	1.14	1.03
ILMN_1371603	Rabggtb	-1.14	1.55	1.45	1.69	1.2	1.14	1.03
ILMN_1365283	Tjp1_predicted	-1.11	1.09	-1.15	-1.05	1.21	1.14	1.03
ILMN_1352183	Zfp68_predicted	1.02	-1.06	1.12	1	1.29	1.14	1.03
ILMN_1363474	RGD1307682	-1.15	-1.13	-1.27	-1.48	1.02	1.15	1.03
ILMN_1352038	Nuak2	1.13	2.61	1.87	1.77	1.05	1.15	1.03
ILMN_1376581	Eif3s6	-1.09	1.52	1.52	1.26	1.13	1.15	1.03
ILMN_1355163	Bcl2l1	1.02	1.02	1.04	1.15	1.18	1.15	1.03
ILMN_1370397	Ppib	-1.13	-1.17	1	-1.19	1.21	1.16	1.03

ILMN_1359124	Arf6	1.06	1.04	1.16	1.21	1.27	1.17	1.03
ILMN_1373735	LOC497832	-1.05	1.03	1.03	-1.16	1.1	1.18	1.03
ILMN_1376031	Cnot2_predicted	-1.14	1.01	1.02	1.07	1.12	1.18	1.03
ILMN_1372987	Aste1	-1.01	1.26	1.15	1.26	1.2	1.19	1.03
ILMN_1374374	Dctn2	-1.06	1.25	1.76	1.27	1.24	1.26	1.03
ILMN_1362167	LOC290651	-1.12	-1.1	1.1	-1	1.27	1.27	1.03
ILMN_1530347	LOC290651	-1.12	-1.1	1.1	-1	1.27	1.27	1.03
ILMN_1353080	RGD1308918_predicted	-1.1	1.15	1.06	1.02	1.64	1.32	1.03
ILMN_1369187	Tasp1_predicted	1.33	1.67	1.71	1.58	1.4	1.35	1.03
ILMN_1358358	Stx5a	1.04	-1.07	1.07	-1.16	1.22	1.38	1.03
ILMN_1348927	Tbxas1	1.07	1.5	1.33	1.29	1.34	1.39	1.03
ILMN_1354937	Rpl13	1.02	1.38	-1.03	1.38	1.35	1.41	1.03
ILMN_1357070	Rnpc1_predicted	-1.04	1.19	-1.12	1.38	1.31	1.43	1.03
ILMN_1650077	LOC361315	-1.05	-1.03	1.16	-1.14	1.58	1.52	1.03
ILMN_1356628	Nfkbia	-1.06	-1.09	-1.3	-1.15	1.45	1.69	1.03
ILMN_1365234	Slc22a7	-1.03	1.16	1	-1.18	-1.34	-1.52	1.04
ILMN_1369809	Ppil3	-1.03	-1.26	-1.3	-1.07	-1.23	-1.5	1.04
ILMN_1376827	LMO7	-1.07	-1.53	-1.36	-1.42	-1.2	-1.46	1.04
ILMN_1362764	MGC94704	1.09	1.06	-1.15	-1.06	-1.27	-1.42	1.04
ILMN_1358980	Atp6v0e2	1.11	1.02	1.78	1.15	-1.31	-1.41	1.04
ILMN_1373201	Eif4a1	-1.01	1.01	-1.07	1.04	-1.13	-1.34	1.04
ILMN_1373200	Cpsf2_predicted	1.03	1.06	1.18	1.08	-1.18	-1.33	1.04
ILMN_1349614	RGD1560917_predicted	1.07	-1.29	-1.65	-1.01	-1.12	-1.33	1.04
ILMN_1354878	Lsm7_predicted	1.26	1.18	-1.62	1.28	-1.29	-1.32	1.04
ILMN_1368154	RGD1563701_predicted	1.19	1.02	-1.03	1.12	-1.06	-1.24	1.04
ILMN_1356866	Polr2g	-1.01	1.03	-1.21	-1.04	-1.02	-1.23	1.04
ILMN_1370686	LOC499330	1.01	-1.34	-1.19	-1.37	-1.1	-1.22	1.04
ILMN_1371587	Ik	-1.09	-1.5	-1.4	-1.44	-1.25	-1.21	1.04
ILMN_1364899	Col18a1	1	1.3	-1.2	1.28	-1.05	-1.21	1.04
ILMN_1372085	Thrap6_predicted	1.06	-1.14	-1.08	-1.1	-1.18	-1.2	1.04
ILMN_1370265	Ndufb5_predicted	-1.05	1.02	-1.33	-1.08	-1.05	-1.2	1.04
ILMN_1369103	Rps26	1.17	1.21	-1.25	1.01	-1.1	-1.17	1.04
ILMN_1351818	Ndufv2	-1.02	1.05	-1.04	-1.05	-1.05	-1.17	1.04
ILMN_1372105	RGD1306209	-1.02	-1.01	1.23	1.26	1.01	-1.15	1.04
ILMN_1363937	RGD1309077_predicted	1.1	1.04	1.27	1.08	-1.03	-1.13	1.04
ILMN_1362847	Cova1_predicted	1.05	-1.17	1.01	-1.44	1.03	-1.13	1.04
ILMN_1371004	Rps16	1.15	1.19	1.13	1.07	-1.12	-1.12	1.04
ILMN_1358399	RGD1565105_predicted	1.19	1.18	-1.73	-1.12	-1.04	-1.11	1.04

ILMN_1367577	Slc4a4	-1.13	-1.23	-1.25	-1.23	-1.2	-1.1	1.04
ILMN_1363889	LOC365612	1.27	-1.08	-1.24	-1.04	-1.02	-1.09	1.04
ILMN_1352151	Mds024	1.01	1.04	1.16	1.08	-1.13	-1.08	1.04
ILMN_1650930	RGD1560519_predicted	1.05	1.01	-1.17	1.04	-1.18	-1.07	1.04
ILMN_1352134	Psmc3	-1.13	-1.35	-1.12	-1.07	-1.15	-1.07	1.04
ILMN_1366667	Prkag2	1.06	-1.1	-1.33	1.09	-1.08	-1.06	1.04
ILMN_1349352	Mrpl35_predicted	1.1	-1.15	-1.07	-1.07	-1.01	-1.06	1.04
ILMN_1362206	RGD1559639_predicted	1.09	1.09	-1.15	-1.01	-1.08	-1.05	1.04
ILMN_1357745	Smn1	1.06	1.2	1.3	1.26	-1.02	-1.05	1.04
ILMN_1359217	Mpdu1	1	1.12	1	-1.02	1.01	-1.04	1.04
ILMN_1359842	LOC498123	-1.23	-1.16	-1.36	-1.01	1.15	-1.04	1.04
ILMN_1364359	Leprot	-1.08	-1.24	-1.38	-1.74	-1.12	-1.03	1.04
ILMN_1372306	Uqcrh	1.01	1.16	-1.38	-1.11	-1.05	-1.02	1.04
ILMN_1372153	Lactb2_predicted	-1.14	-1.39	1.04	-1.2	1.23	-1.02	1.04
ILMN_1360654	Rbms2	1.2	1.41	1.22	1.14	1.08	-1.01	1.04
ILMN_1373170	Kcmf1	1.12	1.25	1.14	-1.1	1.08	-1	1.04
ILMN_1359056	Diablo	-1.1	-1.35	-1.02	-1.05	-1.08	1.02	1.04
ILMN_1351328	Dcun1d4_predicted	1.05	1.33	1.11	1.28	1.02	1.02	1.04
ILMN_1358544	Mrpl45_predicted	1.2	1.05	-1.42	1.09	1.03	1.02	1.04
ILMN_1369210	RGD1309326	1.15	1.18	1.77	1.88	1.08	1.03	1.04
ILMN_1367367	Dgcr6_predicted	1.12	1.07	-1.03	-1.03	1.13	1.03	1.04
ILMN_1368798	RGD1309576_predicted	1.04	-1.04	1.01	-1.14	1.27	1.03	1.04
ILMN_1368515	Calr	-1.28	-1.49	1.08	-1.3	1.1	1.04	1.04
ILMN_1354368	Gprasp1	1.14	1.19	1.03	1.12	1.04	1.06	1.04
ILMN_1351636	Clptm1_predicted	1.14	-1.1	-1.02	-1.07	-1.2	1.07	1.04
ILMN_1352367	Nol9	1.26	1.11	1.38	1.26	-1.04	1.07	1.04
ILMN_1361685	Dnajc11_predicted	-1.08	1.31	1.27	1.19	1.11	1.07	1.04
ILMN_1374437	LOC291905	-1.01	-1.08	-1.17	-1.32	1.13	1.08	1.04
ILMN_1362058	Scfd1	-1.35	-1.43	-1.18	-1.82	1.33	1.08	1.04
ILMN_1359594	Ranbp10_predicted	-1.12	1.05	-1.16	-1.18	1.01	1.09	1.04
ILMN_1367622	RGD1560852_predicted	1.11	1.3	1.28	1.35	1.07	1.1	1.04
ILMN_1368769	Nfib	-1.02	-1.06	-1.24	-1.32	1.03	1.11	1.04
ILMN_1362699	Ganab_predicted	-1.03	-1.08	-1.25	-1.19	1.08	1.11	1.04
ILMN_1369049	MGC114381	1.2	-1.04	1.22	1.17	1.06	1.12	1.04
ILMN_1373193	Prkce	-1.03	1.24	1.01	1.11	1.31	1.12	1.04
ILMN_1374713	Gnb2l1	-1	-1.07	1.29	1.07	1.04	1.13	1.04
ILMN_1370255	Utp14a	1.04	1.12	-1.06	1.12	1.06	1.13	1.04
ILMN_1371924	Utp14a	1.04	1.12	-1.06	1.12	1.06	1.13	1.04

ILMN_1357060	Mrpl3_predicted	-1.08	1.02	1.01	1.05	1.1	1.13	1.04
ILMN_1352177	Bcr_predicted	-1.03	1.18	-1.01	1.19	1.05	1.14	1.04
ILMN_1357049	Nosip_predicted	-1.2	1.01	-1.02	-1.07	1.09	1.14	1.04
ILMN_1350891	Gmps	-1.04	1.43	1.73	1.52	1.09	1.14	1.04
ILMN_1355676	RGD1359158	1.04	-1.07	-1.11	-1.14	-1.04	1.15	1.04
ILMN_1370758	LOC500005	-1.14	-1.08	-1.05	1.05	1.15	1.16	1.04
ILMN_1376964	RGD1311316	1	1.04	1.07	1.13	1.18	1.16	1.04
ILMN_1364828	ORF19	-1.07	1.08	1.23	-1.01	1.04	1.17	1.04
ILMN_1376209	Rnf146_predicted	1.07	1.05	1.58	-1	1.21	1.17	1.04
ILMN_1369755	Mrps18a	-1.08	1.23	-1.08	1.27	1.07	1.18	1.04
ILMN_1359759	Scnn1a	1.04	1.07	1.2	1.08	1.22	1.18	1.04
ILMN_1352362	Snrpd2_predicted	1.2	1.3	-1.13	1.22	1.01	1.19	1.04
ILMN_1353053	Snrpd2_predicted	1.2	1.3	-1.13	1.22	1.01	1.19	1.04
ILMN_1359518	Snrpd2_predicted	1.2	1.3	-1.13	1.22	1.01	1.19	1.04
ILMN_1376598	Trip10	-1.14	1.35	1.22	1.87	1.14	1.19	1.04
ILMN_1356800	Calcoco1	-1.03	-1.12	-1.22	-1.39	1.03	1.2	1.04
ILMN_1363211	Cs	1.05	1.21	1.28	1.26	1.24	1.2	1.04
ILMN_1354514	Perq1_predicted	1	1.11	-1.26	1.12	-1.02	1.21	1.04
ILMN_1365751	Kpnb3_predicted	1.22	1.15	1.05	1.34	1.28	1.21	1.04
ILMN_1650927	RGD1564081_predicted	1.22	1.18	1.25	1.11	1.35	1.21	1.04
ILMN_1351933	Rela	-1.13	-1.11	-1.29	-1.12	1.19	1.24	1.04
ILMN_1368882	RGD1305031	-1.21	1.09	1.03	-1.09	1.2	1.26	1.04
ILMN_1650933	Pphln1_predicted	1	1.02	1.01	1.44	1.34	1.26	1.04
ILMN_1366286	Ube2g2_predicted	1.23	1.07	-1.06	1.19	1.48	1.26	1.04
ILMN_1363281	LOC309035	1.03	1.22	1.3	1.3	1.24	1.31	1.04
ILMN_1366913	Sec24b_predicted	-1.01	1.38	1.26	1.46	1.3	1.33	1.04
ILMN_1352368	Atp10a	1.18	1.29	1.19	1.66	1.1	1.34	1.04
ILMN_1354135	Scyl2_predicted	1.08	1.23	1.14	1.39	1.45	1.41	1.04
ILMN_1353974	P4ha2_predicted	-1	1.13	1.1	1.11	1.16	1.44	1.04
ILMN_1359650	LOC501614	-1.04	-1.04	-1.06	-1.29	1.42	1.47	1.04
ILMN_1362588	LOC297968	-1.13	1	1.02	1.1	1.49	1.62	1.04
ILMN_1360280	Snapc4_predicted	-1.28	1.11	-1.28	1.19	-1.33	-1.35	1.05
ILMN_1361190	RGD1559917_predicted	-1.11	-1.59	-1.24	-1.17	-1.24	-1.28	1.05
ILMN_1356474	Retsat	1.07	1.21	1.1	-1.05	-1.47	-1.23	1.05
ILMN_1367596	Mocs2	-1.03	-1.27	-1.41	-1.6	-1.06	-1.2	1.05
ILMN_1357296	Gfm2	-1.12	-1.22	1.05	-1.24	-1.18	-1.19	1.05
ILMN_1368534	LOC501601	1.24	-1.25	-1.22	-1.15	-1.17	-1.18	1.05
ILMN_1366313	Ercc8_predicted	-1.08	-1.37	-1.16	-1.12	-1.05	-1.17	1.05

ILMN_1359982	Ccdc51	1.03	1.09	1.11	1.07	1.02	-1.17	1.05
ILMN_1360290	LOC684106	1.01	1.12	1.37	1.25	-1.15	-1.14	1.05
ILMN_1366780	Arsb	1.07	-1.45	-1.44	-1.35	-1.01	-1.14	1.05
ILMN_1367325	Dcakd	1.06	1.04	1.29	1.11	-1.05	-1.13	1.05
ILMN_1357910	RGD1304748	-1.04	1.08	-1.04	-1.21	-1.35	-1.12	1.05
ILMN_1358667	Cdipt	1.18	-1	1.1	1.08	-1.06	-1.11	1.05
ILMN_1365786	RGD1305145_predicted	-1.11	1.2	1.01	-1.03	-1.18	-1.1	1.05
ILMN_1353756	RGD1562094_predicted	-1.1	1.02	-1.05	-1.19	-1.13	-1.09	1.05
ILMN_1362916	Rps4x	-1.04	-1	1.06	-1	-1.03	-1.09	1.05
ILMN_1352098	LOC368190	1	-1.06	1.31	-1.01	1.01	-1.08	1.05
ILMN_1364477	RGD1309610_predicted	1.29	1.11	-1.26	-1.58	-1.11	-1.07	1.05
ILMN_1366473	Dclre1c	-1.14	1.01	-1.12	-1.23	-1.09	-1.07	1.05
ILMN_1364567	Atpbd1b_predicted	-1.08	1.09	1.01	1.26	1.01	-1.07	1.05
ILMN_1355760	Pgm1_predicted	-1.04	-1.16	1.15	-1.03	1.01	-1.06	1.05
ILMN_1363624	RGD1309148_predicted	1.11	-1.07	-1.53	-1.06	-1.02	-1.04	1.05
ILMN_1363904	Gabarapl2	1.02	-1.64	-1.41	-1.78	1.01	-1.04	1.05
ILMN_1372093	RGD1559786	-1.15	-1.02	-1.02	-1.12	-1.07	-1.03	1.05
ILMN_1367281	Rpl41	-1.04	1.02	-1.06	1.04	-1.06	-1.03	1.05
ILMN_1355128	Akr1e1	-1.06	-1.17	-1.15	-1.22	1.04	-1.02	1.05
ILMN_1366806	Mrps33_predicted	1.03	1.01	-1.32	-1.22	-1.03	-1.01	1.05
ILMN_1368986	Rara	-1.03	1.22	1.13	1.41	-1	-1.01	1.05
ILMN_1376528	Zfp276	-1.3	-1.13	1.03	1.06	-1.06	1	1.05
ILMN_1370735	Larp5_predicted	-1.06	1.07	1.02	-1.09	1.28	1.01	1.05
ILMN_1354200	LOC502655	-1.05	-1.07	-1.23	1.04	1.03	1.03	1.05
ILMN_1359715	LOC317275	1.08	1.14	1.23	1.18	1.09	1.03	1.05
ILMN_1360093	Pink1_predicted	1.1	-1.18	-1.09	-1.37	-1.12	1.05	1.05
ILMN_1368533	RGD1564423_predicted	-1.17	1.26	1.61	1.28	1.01	1.05	1.05
ILMN_1352327	Pvrl3_predicted	-1.03	1.23	1.29	1.38	1.19	1.06	1.05
ILMN_1368179	LOC689820	1.16	1.54	1.74	1.6	-1	1.08	1.05
ILMN_1351573	Ndufb4	1.01	-1.12	-1.94	-1.13	1.06	1.08	1.05
ILMN_1349564	LOC315160	1.03	1.27	1.25	1.25	1.25	1.08	1.05
ILMN_1373249	Cul3_predicted	-1.02	1.04	1.02	-1.04	1.41	1.08	1.05
ILMN_1371793	Wwp2_predicted	-1.04	1.19	1.22	1.03	-1.02	1.09	1.05
ILMN_1374358	Hdac7a	1.07	1.6	-1.03	1.2	1.08	1.09	1.05
ILMN_1369505	Gfpt1	-1.17	1.23	1.11	1.4	-1.12	1.1	1.05
ILMN_1368997	RGD1561500_predicted	1.02	1.05	-1.05	1.21	1	1.1	1.05
ILMN_1376851	Rnd3	-1.11	-1.23	-1.03	-1.15	1.17	1.11	1.05
ILMN_1359941	Cdc27	1.17	1.32	1.35	1.34	-1.01	1.12	1.05

ILMN_1352006	LOC367779	-1.01	-1.17	-1.24	-1.3	1.08	1.12	1.05
ILMN_1357484	Ncbp1	1.13	1.24	1.18	1.5	1.12	1.13	1.05
ILMN_1353191	Igf2bp3	1.02	1.08	-1.07	1.1	1.18	1.14	1.05
ILMN_1373934	Trim33_predicted	1.05	1.32	1.33	1.24	1.27	1.14	1.05
ILMN_1361239	Arl10	1.14	1.16	1.54	1.16	1.12	1.15	1.05
ILMN_1364569	Cd24	-1.18	1.11	-1.18	-1.09	1.15	1.15	1.05
ILMN_1376610	Tbce	-1.01	1.25	1.18	1.11	1.1	1.16	1.05
ILMN_1358860	RGD1306583	1.03	1.32	1.46	1.21	1.12	1.16	1.05
ILMN_1376965	Btbd1	-1.2	1.19	1.33	1.18	-1.04	1.17	1.05
ILMN_1366343	Aldh9a1	1.02	-1.08	1.57	-1.21	1.07	1.17	1.05
ILMN_1360052	LOC287622	1.28	1.46	1.35	1.83	1.24	1.19	1.05
ILMN_1376724	Map3k7ip2	1.02	1.4	1.3	1.34	1.26	1.19	1.05
ILMN_1373935	LOC305350	1.12	1.27	1.32	1.06	1.11	1.2	1.05
ILMN_1376742	Prpf4b	-1.08	1.25	-1.01	1.23	1.25	1.21	1.05
ILMN_1358798	LOC361646	-1.05	1.02	-1.12	-1.15	1.16	1.23	1.05
ILMN_1351457	Ddx1	1.01	1.33	1.55	1.49	1.25	1.23	1.05
ILMN_1349919	Ggps1	1.09	1.14	1.16	1.12	1.05	1.25	1.05
ILMN_1350381	Slc29a1	1.12	1.21	1.21	1.69	1.28	1.26	1.05
ILMN_1376359	Dr1	1.02	1.04	1.16	1.15	1.3	1.27	1.05
ILMN_1373442	Stat3	-1.03	1.19	1.17	1.13	1.33	1.27	1.05
ILMN_1368392	Tfb2m	1.13	1.19	1.11	-1.08	1.26	1.28	1.05
ILMN_1349732	RGD1310481_predicted	-1.12	-1.09	-1.09	-1.14	1.18	1.29	1.05
ILMN_1352179	Myct1_predicted	-1.01	1.04	1.07	1.14	-1.12	1.31	1.05
ILMN_1355679	Cdv1	1.26	1.34	1.69	1.39	1.27	1.31	1.05
ILMN_1349474	RGD1305036_predicted	-1.06	-1.02	-1.23	-1.08	1.19	1.34	1.05
ILMN_1376205	Lpin1_predicted	-1.1	-1.02	1.03	-1.13	1.33	1.36	1.05
ILMN_1351485	Slc40a1	1.34	-2.3	-2.02	-1.85	-1.47	-1.42	1.06
ILMN_2040544	Faim	-1.08	-1.34	-1.47	-1.24	-1.39	-1.42	1.06
ILMN_1363260	RGD1310193_predicted	1.03	1.24	1.17	1.07	-1.44	-1.4	1.06
ILMN_1359253	MGC114471	-1.1	-1	1.15	-1.02	-1.2	-1.24	1.06
ILMN_1376882	Cmtm6	1.03	-1.09	-1.07	-1.09	-1.13	-1.24	1.06
ILMN_1366907	Keap1	1.07	-1.02	1.16	-1.21	-1.38	-1.21	1.06
ILMN_1361214	Ncor2_predicted	-1.28	1.06	-1.14	1.13	-1.14	-1.2	1.06
ILMN_1352004	Lass2_predicted	1.02	1.02	1.17	1	-1.09	-1.2	1.06
ILMN_1369111	Cep57	-1.1	-1.29	-1.14	-1.08	1.02	-1.19	1.06
ILMN_1373881	Ppp2r4_predicted	1.24	-1.02	-1.19	-1.06	-1.19	-1.18	1.06
ILMN_1351838	Mrpl42_predicted	1.07	-1.16	-1.31	-1.21	-1.17	-1.18	1.06
ILMN_1370835	Ghr	-1.06	-1.19	-1.05	-1.27	-1.01	-1.16	1.06

ILMN_1369994	LOC288913	1.02	-1.12	-1.82	-1.28	-1.05	-1.12	1.06
ILMN_1349800	LOC501491	-1.07	1.05	1.16	-1.31	-1.05	-1.11	1.06
ILMN_1372678	Brf2	-1.1	-1.11	-1.08	-1.11	-1.04	-1.11	1.06
ILMN_1368896	RGD1311484	1.01	1.15	1.25	1.11	-1.05	-1.08	1.06
ILMN_1362803	Dennd2d_predicted	1.31	1.45	1.7	1.74	-1.06	-1.06	1.06
ILMN_1650728	Kdelr1	-1.2	-1.16	-1.23	-1.3	-1.02	-1.06	1.06
ILMN_1363676	RGD1305050_predicted	-1.12	-1.01	-1.06	-1.19	-1	-1.06	1.06
ILMN_1368347	Tbcc_predicted	1.03	1.14	1.21	1.24	-1.14	-1.04	1.06
ILMN_1369665	LOC296813	1.19	1.11	1.21	1.03	-1.07	-1.04	1.06
ILMN_1362014	Bcas2_predicted	-1.02	-1.12	-1.1	-1.17	-1.01	-1.04	1.06
ILMN_1650624	LOC290864	-1.16	1.09	-1.09	-1.08	1	-1.04	1.06
ILMN_1372442	Ipo4_predicted	-1.07	1.85	1.67	1.62	1	-1.03	1.06
ILMN_1358532	Atp13a1_predicted	1.06	1.29	1.24	1.21	-1.12	-1.02	1.06
ILMN_1374177	RGD1304621_predicted	-1.06	1.16	1.11	1.46	-1.09	-1.02	1.06
ILMN_1361964	Ube2i	-1.05	-1.24	-1.4	1.02	1.03	-1.02	1.06
ILMN_1372341	Dnaja2	1.03	1.22	1.45	1.32	1.04	-1.02	1.06
ILMN_1366130	Cfdp1	1.13	1.15	1.38	1.22	1.06	-1.02	1.06
ILMN_1371535	Cxcl11	1.06	-1.19	-1.14	-1.38	1.15	-1.02	1.06
ILMN_1350828	RGD1305890	1.06	1.1	-1.09	1.32	1.18	-1.02	1.06
ILMN_1351368	LOC362845	-1.01	-1.01	1.01	-1.17	-1.09	-1	1.06
ILMN_1368346	Eif2c2	1.07	-1.04	1.06	1.18	-1.06	1	1.06
ILMN_1359066	RGD1560568_predicted	-1.06	1.21	1.16	1.16	1.04	1	1.06
ILMN_1358581	Rps10	1.01	1.15	-1.16	1.02	1.1	1	1.06
ILMN_1376743	Sphk2	-1.07	-1.02	-1.08	-1.09	1.03	1.01	1.06
ILMN_1371095	Spred2_predicted	-1.04	1.05	1.29	1.1	1.05	1.01	1.06
ILMN_1359883	Phf17_predicted	1.02	1.03	1.08	1.39	1.07	1.01	1.06
ILMN_1357607	RGD1305984	1.23	1.02	1.17	-1.03	-1.01	1.02	1.06
ILMN_1364360	Fkbp3_predicted	-1.02	-1.03	1.02	1.07	-1.01	1.02	1.06
ILMN_1370695	Fbxo18_predicted	-1.05	1.1	-1.13	1.09	1.06	1.02	1.06
ILMN_1371355	RGD1309188_predicted	1.14	1.12	1.15	1.33	1.15	1.02	1.06
ILMN_1373732	Akr1a1	-1.07	-1.07	1.09	-1.14	-1.01	1.03	1.06
ILMN_1370284	Psma6	-1.13	-1.13	-1.02	1	1.05	1.03	1.06
ILMN_1360933	MGC72942	1.17	1.03	-1.89	1.06	1.07	1.03	1.06
ILMN_1371241	Wdr39	1.24	1.03	1.01	-1	1.04	1.04	1.06
ILMN_1359760	Rpl18	1.05	1.06	1.08	1.1	-1.09	1.05	1.06
ILMN_1356562	Alg1_predicted	1.09	1.18	1.24	1.24	-1.11	1.08	1.06
ILMN_1359389	LOC500277	1.21	1.34	1.51	1.53	1.02	1.08	1.06
ILMN_1353690	LOC498606	1.1	1.12	-1.14	-1.04	1.01	1.09	1.06

ILMN_1366429	Bmp2k	1.01	1.15	1.02	1.09	1.07	1.09	1.06
ILMN_1376329	Fmip	-1.01	1.17	1.05	1.13	1.11	1.09	1.06
ILMN_1364136	RGD1307679	-1.08	-1	1.04	1.13	1.17	1.09	1.06
ILMN_1355230	Psma5	1.08	-1.1	-1.05	1.13	1.23	1.09	1.06
ILMN_1358572	Tp53rk_predicted	1.03	1.41	1.41	1.26	1.12	1.1	1.06
ILMN_1367501	RGD1306323_predicted	1.09	1.36	1.19	1.37	1.12	1.1	1.06
ILMN_1370551	RGD1563888_predicted	1.21	1.41	1.23	1.09	1.16	1.1	1.06
ILMN_1371982	Cycs	1.02	1.06	1.18	1.16	1.19	1.1	1.06
ILMN_1359135	RGD1305287	1.21	1.01	1.1	1.08	-1.02	1.11	1.06
ILMN_1358242	LOC685455	1.08	1.1	1	1.07	1.07	1.11	1.06
ILMN_1372410	Edg5	1.16	1.41	1.1	1.52	1.13	1.11	1.06
ILMN_1650602	LOC499079	-1.03	-1	1.02	1.08	-1.06	1.12	1.06
ILMN_1349367	Acvr1	1.25	1.06	1.05	1.05	1.05	1.12	1.06
ILMN_1349451	Nfx1	-1.09	1.17	1.3	1.23	1.09	1.12	1.06
ILMN_1358886	LOC288165	1.01	-1.34	-1.04	-1.25	1.13	1.12	1.06
ILMN_1352867	Gga3_predicted	-1.01	1.37	1.07	1.6	1.16	1.12	1.06
ILMN_1354764	Wrb	1.18	-1.01	1.17	1.16	1.18	1.12	1.06
ILMN_1355413	Rps3	1.02	1.07	1.19	1.07	1.14	1.13	1.06
ILMN_1366066	Atg16l1_predicted	1.01	-1.02	1.34	1.19	-1.01	1.14	1.06
ILMN_1349804	Atp2b1	-1.01	1.05	1.05	1.2	1.09	1.15	1.06
ILMN_1358093	Arf4	-1.14	-1.19	1.09	-1.07	1.23	1.16	1.06
ILMN_1359440	Nbr1	-1.04	1.02	-1	-1.31	-1.07	1.18	1.06
ILMN_1351935	Mkln1	-1.03	1.12	1.06	1.21	1.19	1.18	1.06
ILMN_1369108	Dync1li1	-1.06	1.09	1.14	-1.03	1.18	1.19	1.06
ILMN_1651062	Dus4l_predicted	1.31	1.18	1.93	1.24	1.19	1.2	1.06
ILMN_1351250	Ubqln1	1.07	1.69	1.69	1.6	1.2	1.2	1.06
ILMN_1363823	Ccdc58_predicted	1.18	1.09	-1.07	1.33	1.3	1.2	1.06
ILMN_1352334	RGD1304653_predicted	1.13	1.12	1.36	1.03	1.18	1.21	1.06
ILMN_1372538	RGD1561176_predicted	1.21	1.09	1.18	1.15	1.28	1.22	1.06
ILMN_1364379	LOC316085	-1.12	1.63	1.08	1.56	1.14	1.23	1.06
ILMN_1650262	RGD1359443	-1.05	1.02	-1.18	-1.2	1.18	1.23	1.06
ILMN_1374699	RGD1559502_predicted	1.02	-1.22	-1.06	1	-1.1	1.24	1.06
ILMN_1376911	Pex6	1.02	1.15	1.04	1.05	1.06	1.24	1.06
ILMN_1361687	LOC690911	1.17	1.38	-1.11	1.09	1.22	1.25	1.06
ILMN_1372586	Ell_predicted	-1.1	-1.05	-1.25	-1.33	1.16	1.26	1.06
ILMN_1363284	Rap1a	-1.01	-1.15	1.06	-1.05	1.32	1.26	1.06
ILMN_1355156	Impad1	1.16	1.09	1.04	1.08	1.07	1.27	1.06
ILMN_1650440	Msl2l1_predicted	1.09	1.12	1.23	1.07	1.21	1.27	1.06

ILMN_1360626	LOC360618	-1.15	1.21	1.14	1.1	1.35	1.27	1.06
ILMN_1376815	Mbd1	-1.1	1.18	1.04	1.25	1.37	1.27	1.06
ILMN_1362760	RGD1559787_predicted	1.1	1.68	1.49	1.81	1.19	1.3	1.06
ILMN_1362203	MGC94288	1.03	1.53	1.33	1.66	1.11	1.31	1.06
ILMN_1367511	Lphn2	1	1.71	1.69	1.63	1.22	1.34	1.06
ILMN_1357747	Ddit4	-1.03	1.07	1.3	1.41	1.63	1.56	1.06
ILMN_1649861	LOC691918	1.02	1.48	1.94	1.38	1.58	1.63	1.06
ILMN_1650148	Golt1b_predicted	1.01	1.16	1.46	1.29	1.63	1.71	1.06
ILMN_1371027	Tmem39a	1.01	1.32	1.47	1.28	2.07	1.91	1.06
ILMN_1355675	Sep-15	-1.18	-1.29	-1.11	-1.38	1.14	1.04	1.07
ILMN_1349635	Ltb4dh	1.48	-2.11	-2.52	-2.66	-1.86	-1.59	1.07
ILMN_1374774	Psenen	-1.02	-1.45	-2.63	-2.26	-1.33	-1.31	1.07
ILMN_1530518	Pacsin3	-1.17	-1.16	-1.09	-1.04	-1.19	-1.3	1.07
ILMN_1376876	Trappc4	1.04	-1.06	-1.14	-1.02	-1.06	-1.21	1.07
ILMN_1359993	Rbbp7	-1.13	-1.45	-1.43	-1.55	-1.39	-1.19	1.07
ILMN_1368886	Hmg20b_predicted	1.02	-1.19	1.18	-1.07	-1.03	-1.18	1.07
ILMN_1372614	Ncald	-1.12	-1.72	-1.75	-2.26	-1.07	-1.17	1.07
ILMN_1357736	Bsg	1.02	-1.63	-1.31	-1.41	-1.03	-1.16	1.07
ILMN_1354609	RGD1310606	1.08	1.03	1.19	-1	-1.14	-1.15	1.07
ILMN_1355488	Nit1	-1.12	-1.11	-1.22	-1.33	-1.12	-1.14	1.07
ILMN_1376334	Arhgef1	-1.14	1.09	-1.46	-1.09	-1.1	-1.14	1.07
ILMN_1354486	Fliih	-1.08	-1.01	1.04	-1	-1.09	-1.14	1.07
ILMN_1355454	Cox6c1	1.07	-1.03	-1.46	1.12	1.03	-1.13	1.07
ILMN_1364813	RGD1564681_predicted	1.1	-1.12	-1.52	-1.19	-1.13	-1.11	1.07
ILMN_1371358	Poldip2_predicted	-1.03	1.17	1.53	1.21	-1.13	-1.1	1.07
ILMN_1357645	Ccbl1	-1.01	-1.15	-1.24	-1.28	1.02	-1.1	1.07
ILMN_1363142	Nmt2	1.01	1.13	1.09	1.23	-1.07	-1.08	1.07
ILMN_1359434	Gprk6	-1.01	-1.01	-1.09	1.1	-1.25	-1.07	1.07
ILMN_2039552	RGD1565486_predicted	-1.18	-1.28	-1.25	-1.04	1.06	-1.04	1.07
ILMN_1363644	RGD1305455	-1.13	1.1	1.11	1.25	1.15	-1.04	1.07
ILMN_1367510	Dnpep	-1.34	-1.17	1.07	-1.19	1.01	-1.03	1.07
ILMN_1355104	MGC112682	-1.08	-1.02	1.02	-1.09	1.07	-1.02	1.07
ILMN_1369013	LOC503351	1.11	1.02	1.46	1.25	1.16	-1.02	1.07
ILMN_1376262	Pigk	-1.11	-1.25	-1.11	-1.2	-1.08	-1.01	1.07
ILMN_1360993	LOC686176	1.04	1.19	-1.05	1.2	-1.08	1	1.07
ILMN_1370347	Polr2d_predicted	-1.02	-1.12	-1.09	-1.1	1.03	1	1.07
ILMN_1361889	Scarb2	1.01	1.47	1.41	1.28	-1.21	1.01	1.07
ILMN_1351403	RGD1310324_predicted	-1.07	1.32	-1.06	-1.05	1.06	1.01	1.07

ILMN_1357472	LOC362526	1.17	1.13	-1.19	1.27	1.11	1.01	1.07
ILMN_1361195	Mterfd2	-1.13	-1.1	-1.05	-1.07	-1.07	1.02	1.07
ILMN_1373087	Lrpprc	1.13	1.59	1.48	1.33	-1.01	1.02	1.07
ILMN_1375503	Zmynd11	1.08	1.11	1.14	1.1	-1	1.02	1.07
ILMN_1651198	Zmynd11	1.08	1.11	1.14	1.1	-1	1.02	1.07
ILMN_1349891	RGD1303074	-1.14	-1.36	-1.18	-1.65	1.22	1.02	1.07
ILMN_2039237	LOC288762	1.02	1.33	1.08	1.04	-1.1	1.03	1.07
ILMN_1365542	LOC360990	-1.04	1.04	-1.11	1.04	-1.09	1.03	1.07
ILMN_1361124	LOC362665	-1.12	1.41	1.08	1.37	1.2	1.03	1.07
ILMN_1370951	RGD1310899_predicted	1	1	-1.23	-1.06	-1.07	1.04	1.07
ILMN_1353322	MGC95210	-1.09	1.05	-1.19	1.04	1.03	1.04	1.07
ILMN_2039597	LOC497968	1.04	1.15	1.25	1.29	1.04	1.04	1.07
ILMN_1376689	Hgd	-1.12	2.07	1.61	1.22	1.16	1.05	1.07
ILMN_1373961	RGD1307648	-1.1	-1.14	-1.06	1.03	1.09	1.06	1.07
ILMN_1372021	LOC690428	1.11	-1.23	-1.84	-1.17	1.02	1.07	1.07
ILMN_1368776	LOC498674	1.08	-1.08	1.2	-1.24	1.13	1.07	1.07
ILMN_1360031	RGD1307615_predicted	-1.02	1.01	-1.07	-1	1.08	1.08	1.07
ILMN_1376500	Ero1l	1.07	1.23	1.2	1.44	1.38	1.08	1.07
ILMN_1368668	RGD1359634	-1.12	1.04	-1.08	-1.13	1.21	1.09	1.07
ILMN_1366979	Cnot10	-1.05	1.04	-1.11	1.02	1.01	1.11	1.07
ILMN_1359837	LOC499900	1.12	1.38	1.37	1.37	1.03	1.11	1.07
ILMN_2040414	Ilk	-1.1	-1.16	-1.06	-1.01	1.04	1.11	1.07
ILMN_2040391	Ilk	-1.1	-1.16	-1.06	-1.01	1.04	1.11	1.07
ILMN_2039085	Ilk	-1.1	-1.16	-1.06	-1.01	1.04	1.11	1.07
ILMN_1366218	Cdk9	-1.02	1.21	1.33	1.35	1.11	1.11	1.07
ILMN_1364095	LOC362703	1.4	1.54	1.56	1.76	1.01	1.12	1.07
ILMN_1373517	Slc35c2	1.16	1.27	1.28	1.12	-1.15	1.14	1.07
ILMN_1358842	RGD1562823_predicted	1.18	-1.13	-1.19	1.26	1.15	1.14	1.07
ILMN_1357522	Lrp1	1.08	1.31	-1.07	1.12	1.18	1.14	1.07
ILMN_1358542	Fdft1	1.07	-1.62	-1.17	-1.45	1.01	1.15	1.07
ILMN_1360378	LOC301448	1.06	1.27	1.39	1.35	1.25	1.16	1.07
ILMN_1375175	Ppp6c	-1.02	1.12	1.25	1.12	1.13	1.18	1.07
ILMN_1361069	RGD1311559_predicted	-1.06	1.15	-1.05	1.11	1.18	1.18	1.07
ILMN_1361946	RGD1304792_predicted	-1.27	-1.1	1.06	1.2	1.06	1.19	1.07
ILMN_1367047	Rora_predicted	1.09	1.12	-1.1	-1.24	1.38	1.2	1.07
ILMN_1372575	Kctd5_predicted	-1.03	-1.11	1.2	1.01	1.07	1.21	1.07
ILMN_1359271	NIPBL	-1.21	-1.07	-1.08	1.05	1.16	1.21	1.07
ILMN_1351349	RGD1565299_predicted	-1.15	-1.15	1.41	-1.07	1.2	1.22	1.07

ILMN_1370386	Cdc42bpb	-1.1	1.11	-1.01	1.17	-1.06	1.23	1.07
ILMN_1367094	Znf511_predicted	1.17	1.21	1.02	1.16	1.31	1.23	1.07
ILMN_1364293	Dncli2	1.03	1.31	1.28	1.22	1.15	1.26	1.07
ILMN_1361729	Necap2	-1.25	-1.2	-1.23	-1.14	1.18	1.26	1.07
ILMN_1352809	Mapk14	1	1.08	1	1.13	1.28	1.26	1.07
ILMN_1364277	Gpt1	-1.18	1.6	1.7	1.36	1.38	1.27	1.07
ILMN_1371389	Myc	1.83	2.62	2.79	2.75	1.26	1.29	1.07
ILMN_1364215	MGC72992	-1.04	-1.01	1.08	1.12	1.32	1.29	1.07
ILMN_1359896	Chd4	-1.09	1.15	1.46	1.46	1.29	1.31	1.07
ILMN_1354255	RGD1306862_predicted	-1	1.16	1.21	1.4	1.27	1.32	1.07
ILMN_1351969	LOC363266	1.08	1.47	1.35	1.53	1.29	1.32	1.07
ILMN_1371068	H13_predicted	1.04	-1.22	1.42	1.01	1.44	1.32	1.07
ILMN_1352173	Hnrpll_predicted	-1.14	1.15	1.4	1.31	1.64	1.32	1.07
ILMN_1359324	Olr1726_predicted	-1.06	1.07	1.03	-1.07	1.11	1.33	1.07
ILMN_1370716	Dscr1	-1.01	1.12	1.09	1.06	1.3	1.33	1.07
ILMN_1373010	Plekhf2_predicted	1.15	1.3	1.19	1.4	1.34	1.39	1.07
ILMN_1367148	LOC497663	-1.04	1.42	-1.04	1.11	1.45	1.41	1.07
ILMN_1375156	Erf_predicted	-1.01	1.71	1.02	1.84	1.42	1.42	1.07
ILMN_1355054	Synj1	-1.02	1.13	1.07	1.08	1.46	1.44	1.07
ILMN_1365581	Phf3_predicted	1.01	1.34	1.14	1.1	1.29	1.5	1.07
ILMN_1361990	Sctr	1.14	1.27	1.28	1.21	1.26	1.54	1.07
ILMN_1373530	Creld2	-1.08	-1.74	-1.27	-1.23	2.86	2.28	1.07
ILMN_1364162	Ggcx	1.03	-1.11	1.32	-1.38	-1.37	-1.49	1.08
ILMN_1350133	RGD1561543_predicted	1.08	-1.48	-1.77	-1.4	-1.45	-1.35	1.08
ILMN_1367610	Fbf1_predicted	-1.19	1.08	-1.11	-1.07	-1.35	-1.34	1.08
ILMN_1368699	Slc11a2	1.01	-1.28	-1.43	1.04	-1.44	-1.27	1.08
ILMN_1359521	MGC112790	1.08	-1.37	-1.48	-1.3	-1.01	-1.25	1.08
ILMN_1358479	Lass5_predicted	1.1	-1.62	-1.29	-1.24	-1.16	-1.24	1.08
ILMN_1357202	Phkb	-1.01	-1.11	-1.14	-1.3	-1.14	-1.24	1.08
ILMN_1363585	Cox6c	1.04	-1.08	-1.28	-1.17	-1.09	-1.24	1.08
ILMN_1361247	Itpa_mapped	-1.15	-1.07	1.02	-1.04	-1.07	-1.23	1.08
ILMN_1355706	Thrap5_predicted	1.34	1.04	1.05	1.56	-1.1	-1.21	1.08
ILMN_1354390	LOC498687	-1.08	-1.11	-1.39	-1.23	-1.25	-1.18	1.08
ILMN_1373039	R3hcc1_predicted	-1.11	-1.27	-1.09	-1.36	-1.17	-1.18	1.08
ILMN_1355596	RGD1307493_predicted	1.13	-1.22	1.03	1.03	-1.03	-1.17	1.08
ILMN_1352471	Ahsg	-1.11	1.18	1.23	1.09	-1.03	-1.14	1.08
ILMN_1376471	Klhl7	1.04	-1.06	-1.05	-1.06	-1.11	-1.12	1.08
ILMN_1356478	Samm50	1.06	1.15	1.23	1	-1.06	-1.11	1.08

ILMN_1650724	Pnpla2_predicted	-1.05	-1.07	-1.15	-1.22	-1.25	-1.1	1.08
ILMN_1376591	Lcmt2	-1.12	-1.01	-1.23	-1.1	-1.09	-1.09	1.08
ILMN_1357570	LOC680222	1.07	-1.25	-1.21	-1.37	-1.16	-1.07	1.08
ILMN_1360011	LOC498045	-1.7	-1.26	-1.1	1.17	-1.13	-1.07	1.08
ILMN_1362208	RGD1560334_predicted	1.06	1	-1.18	1.01	1.23	-1.06	1.08
ILMN_1350152	Gtf2h2_predicted	1.13	1.25	1.2	1.21	-1.09	-1.05	1.08
ILMN_1366779	Uck2	1.22	1.35	1.56	1.45	-1.17	-1.04	1.08
ILMN_1352800	Lkap	-1.11	-1.24	-1.62	-1.57	-1.18	-1.03	1.08
ILMN_1349287	Hiat1_predicted	-1.03	1.18	1.01	1.17	-1.07	-1.03	1.08
ILMN_1356180	RGD1310320	-1.06	-1.13	1.35	1.15	-1.03	-1.01	1.08
ILMN_1373791	Brms1	-1.07	-1.09	1.04	-1.26	-1.01	-1.01	1.08
ILMN_1376984	Nsep1	-1.02	-1.27	-1.03	1.05	-1.01	-1.01	1.08
ILMN_1373585	LOC685664	1.11	1.22	1.26	1.34	1	-1.01	1.08
ILMN_1352045	RGD1562218	-1.09	1.11	-1.17	-1.08	1.1	-1.01	1.08
ILMN_1368629	Rnf187_predicted	1.02	-1.48	-1.15	1.07	-1.11	-1	1.08
ILMN_1362466	LOC316415	1.02	-1.03	-1.36	1.06	1.28	-1	1.08
ILMN_1357776	Cald1	-1.04	-1.06	1.11	-1.39	1	1	1.08
ILMN_1367197	Tmco1	-1.03	-1.12	1.03	-1.2	1.13	1	1.08
ILMN_1357622	Capns1	-1.03	-1.41	-1.04	-1.29	-1.04	1.01	1.08
ILMN_1374493	Sdhb_predicted	1.04	1.15	1.16	1.05	1.13	1.02	1.08
ILMN_1359322	Nudc	1.1	1.05	1.25	1.12	-1.06	1.03	1.08
ILMN_1361526	Sec23a_predicted	-1.1	-1.2	-1.36	-1.27	1.16	1.03	1.08
ILMN_2040171	Nfs1	-1.04	-1.19	1.03	-1.26	-1.19	1.04	1.08
ILMN_1357378	LOC501217	-1.04	-1.06	1.34	1.02	1.01	1.04	1.08
ILMN_1361828	Pick1	1.03	1.17	1.22	1.21	1.02	1.04	1.08
ILMN_1650328	Rnu3ip2_predicted	1.34	1.65	1.85	2.08	1.03	1.04	1.08
ILMN_1375963	Calr3_predicted	-1.07	-1.09	1.12	-1.05	1.28	1.04	1.08
ILMN_1374105	Adipor1	-1.01	-1.08	-1.24	-1.34	-1.1	1.05	1.08
ILMN_1363262	Csnk2a1	1.09	1.13	1.4	1.13	1.09	1.05	1.08
ILMN_1357092	Gnb2	1.02	1.13	1.27	1.26	1.15	1.05	1.08
ILMN_1372549	Ubxd8	-1.02	-1.07	-1.01	-1.18	1	1.06	1.08
ILMN_1352498	RGD1564277_predicted	-1.07	-1.09	-1.19	1.16	1.02	1.06	1.08
ILMN_1349540	Dlgh1	1.16	1.18	1.56	1.26	1.04	1.06	1.08
ILMN_1364026	Sec61a2_predicted	1.15	1.2	1.27	1.2	1.06	1.06	1.08
ILMN_1374742	Gtf3c1	-1.1	1.5	1.01	1.16	1.02	1.07	1.08
ILMN_1361968	RGD1560523_predicted	1.18	1.35	1.77	1.35	1.04	1.07	1.08
ILMN_1364192	RGD1560523_predicted	1.18	1.35	1.77	1.35	1.04	1.07	1.08
ILMN_1651128	Trmt12	-1.21	-1.06	-1.03	1.16	1.1	1.07	1.08

ILMN_1366750	Rnf6_predicted	1.04	-1.04	-1.03	-1.04	1.01	1.08	1.08
ILMN_1650944	LOC361990	-1.07	1.02	-1.13	-1	1.03	1.09	1.08
ILMN_1362093	Emd	-1	1.19	1.21	1.17	1.08	1.1	1.08
ILMN_1365434	LOC499581	-1	1.06	1.16	1.22	1.1	1.1	1.08
ILMN_1375530	Cnnm2_predicted	-1.06	-1.08	-1.15	-1.49	1.15	1.11	1.08
ILMN_1370510	Gne	-1.22	-1.01	-1.23	-1.09	1.16	1.11	1.08
ILMN_1376576	Nfia	1.01	1.31	-1.25	1.06	1.16	1.11	1.08
ILMN_1349290	Mapk1	1.06	1.08	1.17	1.16	1.2	1.11	1.08
ILMN_1358927	Rassf3_predicted	1.14	1.05	-1.27	-1.03	1.23	1.11	1.08
ILMN_1359121	RGD1559513_predicted	-1.29	1.25	1.2	1.41	1.3	1.12	1.08
ILMN_1376913	Zc3h8	1.36	1.76	2	1.77	1.05	1.13	1.08
ILMN_1356610	Thoc1	-1.1	1.07	1.09	1.04	1.04	1.14	1.08
ILMN_1365259	MGC94941	1.08	1.03	1.08	-1.3	1.13	1.14	1.08
ILMN_2039465	Ndufv3l	-1.04	1.11	1.32	-1.03	1.01	1.15	1.08
ILMN_1356243	Psmd12	1.04	1.14	1.21	1.3	1.17	1.15	1.08
ILMN_1360400	Prkcd	-1.04	1.31	1.1	1.52	1.04	1.16	1.08
ILMN_1362176	MGC94223	-1.1	1.02	1.08	1.01	1.18	1.17	1.08
ILMN_1354758	Agmat	1.34	1.17	1.05	1.2	1.26	1.17	1.08
ILMN_1367664	Nr1h2	-1.03	1.01	-1.22	-1.2	-1.14	1.18	1.08
ILMN_1376365	Herc4	1.02	1.3	1.13	1.42	1.04	1.19	1.08
ILMN_1355538	Mrps30_predicted	-1.01	1.17	1.02	1.13	1.07	1.19	1.08
ILMN_1376341	Polb	-1.06	1.01	-1.12	-1.14	1.17	1.19	1.08
ILMN_1364313	Plod3	1.06	1.53	1.68	1.42	1.14	1.2	1.08
ILMN_1358103	Tars	1.02	1.06	-1.04	1.08	1.23	1.21	1.08
ILMN_1530369	Tars	1.02	1.06	-1.04	1.08	1.23	1.21	1.08
ILMN_1362735	Zfp482_predicted	-1.03	1.1	1.08	1.12	-1.08	1.22	1.08
ILMN_1364937	RGD1559763_predicted	-1.06	1.32	1.69	1.82	1.17	1.23	1.08
ILMN_1372967	Laptm4a	1.03	-1.17	-1.28	-1.31	1.11	1.25	1.08
ILMN_1370874	LOC686274	1.16	1.26	1.23	1.24	1.2	1.27	1.08
ILMN_1351424	RGD1310022	-1.15	1.07	-1.17	1.17	1.22	1.28	1.08
ILMN_1356552	Anapc10_predicted	-1.23	-1.24	1.14	-1.17	1.28	1.31	1.08
ILMN_1368675	Ubn1_predicted	1.06	1.19	1.05	-1.01	1.11	1.33	1.08
ILMN_1650937	LOC365601	1.19	1.16	-1.03	1.02	1.42	1.35	1.08
ILMN_1369148	Cdig2	1.04	1.38	1.32	1.2	1.35	1.36	1.08
ILMN_1361804	Lman1	1.09	1.77	1.93	1.57	1.33	1.39	1.08
ILMN_1368670	Rbm7_predicted	-1.13	1.32	1.19	1.01	1.18	1.4	1.08
ILMN_1370095	Snx16	-1.07	1.2	1.27	1.12	1.42	1.46	1.08
ILMN_1369372	Wipi1_predicted	1.09	-1.38	-1.46	-1.85	1.21	1.47	1.08

ILMN_1349967	RGD1559924_predicted	1.22	1.31	1.24	1.34	1.5	1.56	1.08
ILMN_1352934	MGC94199	1.18	1.67	1.45	1.5	1.51	1.57	1.08
ILMN_1374798	Stch	-1.12	-1.01	1.22	1.13	1.82	1.66	1.08
ILMN_1349335	Sec24d_predicted	-1.14	-1.1	-1.33	-1.2	1.8	1.72	1.08
ILMN_1370359	Sui1-rs1_predicted	1.09	1.22	1.4	1.23	1.66	1.76	1.08
ILMN_1650070	RGD1311946	-1.04	1.07	1.13	1.23	-1.05	-1.32	1.09
ILMN_1373557	Stx4a	-1.01	1.05	1.21	-1.12	-1.2	-1.29	1.09
ILMN_1353289	Ddx31_predicted	1.23	1.16	1.36	1.41	-1.12	-1.23	1.09
ILMN_1356221	RGD1304706	1.1	1.11	1.17	-1	1	-1.19	1.09
ILMN_1373502	RGD1309534	-1.1	-1.17	-1.29	-1.46	-1.23	-1.15	1.09
ILMN_1649800	RGD1307067_predicted	1.1	1.04	-1.06	-1.18	1.07	-1.14	1.09
ILMN_1356179	Lpin2_predicted	1.06	1.39	-1.03	1.21	1.21	-1.13	1.09
ILMN_1368358	RGD1311251	-1.06	1.1	1.02	-1.26	-1.02	-1.11	1.09
ILMN_1363293	RGD1310376_predicted	1.12	-1.04	1.17	-1.04	1	-1.1	1.09
ILMN_1357577	RGD1310376_predicted	1.12	-1.04	1.17	-1.04	1	-1.1	1.09
ILMN_1361547	Centd2	1.01	-1.09	1.08	-1.06	-1.23	-1.09	1.09
ILMN_1361675	Tln1	-1.12	-1.07	-1.22	-1.05	-1.03	-1.09	1.09
ILMN_1650501	Zadh1	-1.15	1.02	1.12	-1.04	1.02	-1.09	1.09
ILMN_1368862	Rage	1.07	1	-1.06	1.18	1.16	-1.09	1.09
ILMN_1364880	Aprt_predicted	1.04	1.21	1.26	1.26	-1.15	-1.07	1.09
ILMN_1362991	Vps45	-1.19	-1.14	1.03	-1.06	1.06	-1.07	1.09
ILMN_1373434	Rab28	-1.27	-1.4	-1.13	-1.26	-1.06	-1.06	1.09
ILMN_1376428	Sema4a	1	1.1	1.14	-1.06	1.2	-1.06	1.09
ILMN_1353674	Ifi35	-1.1	-1.46	-1.36	-1.61	-1.24	-1.05	1.09
ILMN_1363134	Oraov1_predicted	-1.05	1.04	-1.18	-1.05	-1.01	-1.05	1.09
ILMN_1354629	Trim37_predicted	1.03	-1.11	1.09	-1	-1.17	-1.02	1.09
ILMN_1373097	Ergic3_predicted	-1.03	-1.14	-1.07	-1.28	1.08	-1.02	1.09
ILMN_1348782	Etfdh	1.03	-1.06	-1.07	-1.11	1.1	-1.02	1.09
ILMN_1364225	Nt5dc1_predicted	1.02	-1.23	-1.02	-1.07	1.13	-1.02	1.09
ILMN_1348804	Senp3	-1.11	1.04	1.23	1.07	1.13	-1.02	1.09
ILMN_1370209	Prkrir_predicted	1.04	1.24	1.23	1.67	1.2	-1.01	1.09
ILMN_1356283	LOC501665	-1.05	-1.09	-1.01	-1.26	-1.17	-1	1.09
ILMN_1361791	Cetn3	-1.17	-1.27	-1.39	-1.21	-1.08	1.01	1.09
ILMN_1361149	Dus3l	-1.13	1.2	-1.22	1.09	-1.04	1.01	1.09
ILMN_1371867	LOC301117	1.14	1.22	1.22	1.38	1.02	1.01	1.09
ILMN_1362148	Rit1_predicted	1.06	-1.06	-1.19	-1.14	1.1	1.02	1.09
ILMN_1350670	Car9_predicted	1.01	1.11	1.17	1.16	1.28	1.02	1.09
ILMN_1375947	Herc4_predicted	-1.16	1.04	1.29	1.14	-1.05	1.03	1.09

ILMN_1365533	RGD1565385_predicted	-1.04	1.02	-1.08	1.23	1.15	1.03	1.09
ILMN_1373488	MGC105560	-1.03	1.05	-1.07	-1.09	1.02	1.04	1.09
ILMN_1366015	RGD1561676_predicted	-1.08	1.04	1.37	1.38	1.09	1.04	1.09
ILMN_1349675	Tusc4_predicted	-1.04	1.56	1.13	-1.05	1.14	1.04	1.09
ILMN_1373997	Ncoa1_predicted	-1.13	1.23	1.2	1.25	1.33	1.04	1.09
ILMN_1363732	Vars2	1.1	1.36	1.55	1.5	-1.09	1.05	1.09
ILMN_1349110	Cct5	-1.07	1.08	1.44	1.24	-1	1.05	1.09
ILMN_1369272	Nisch	-1.1	1.02	1.3	-1.02	1.01	1.05	1.09
ILMN_2040693	RGD1564943_predicted	1.03	-1.08	1.14	1.1	1.05	1.05	1.09
ILMN_1376438	Acbd6	1.08	1.24	1.06	1.24	1.1	1.05	1.09
ILMN_1353270	Psmb2	1.15	-1.02	-1.05	1.05	1.16	1.05	1.09
ILMN_1362352	Psmc4	-1.04	1.08	1.23	1.27	-1.07	1.06	1.09
ILMN_1369681	LOC362181	-1.03	1.09	1.17	1.15	1.04	1.08	1.09
ILMN_1354829	Rps3a	-1.09	1	-1	1	1.08	1.08	1.09
ILMN_1351396	Ndufab1_predicted	1.17	1.13	-1.26	1.11	1.08	1.08	1.09
ILMN_1371117	Zfp110	1.07	1.51	1.34	1.38	1.12	1.08	1.09
ILMN_1353688	Ireb2	1.05	-1.05	1.21	1.07	1.16	1.08	1.09
ILMN_1650068	RGD1310992	1.18	1.13	1.2	1.19	1.02	1.09	1.09
ILMN_2040275	Rpl19	1.09	1.06	1.11	1.07	1.07	1.1	1.09
ILMN_1371460	Rnf2	1.14	1.14	1.15	-1.02	1.17	1.1	1.09
ILMN_1352625	Ssbp1	-1.02	1.15	-1.06	-1.16	-1.04	1.11	1.09
ILMN_1365024	Cml1	1.05	-1.16	1.23	-1.08	-1.03	1.11	1.09
ILMN_1358045	Usp39_predicted	1.19	1.23	1.41	1.66	1.17	1.11	1.09
ILMN_2040250	Actr1a_predicted	-1.02	-1.08	-1.2	-1.08	1.08	1.12	1.09
ILMN_1365412	Rps27a	-1.05	1.05	1	1.05	1.08	1.12	1.09
ILMN_2040789	Slc25a27	-1.05	1.31	1.18	1.19	1.16	1.12	1.09
ILMN_1352570	Sybl1	-1.02	-1.26	1.06	-1.45	1.18	1.12	1.09
ILMN_1376415	Rpl10	1.06	1.14	1.24	1.1	1.04	1.13	1.09
ILMN_1364240	Camk2g	-1.08	1.21	-1.1	1.14	1.06	1.13	1.09
ILMN_1376833	Cyp27a1	1	1.37	1.48	-1.26	1.07	1.13	1.09
ILMN_1366513	Ahcyl1_predicted	1	1.01	1.08	1.1	-1.2	1.14	1.09
ILMN_1368474	LOC367198	1.29	1.18	1.07	1.37	-1.02	1.14	1.09
ILMN_1376961	Rdh11	-1.01	-1.02	1.21	-1.17	1.1	1.14	1.09
ILMN_1359409	Mrps27_predicted	1.06	1.02	1.09	-1.04	-1.01	1.15	1.09
ILMN_1353766	RGD1566136_predicted	1.11	1.13	-1.44	1.44	1.25	1.15	1.09
ILMN_1354707	Timm23	1.03	-1.07	1.19	-1.01	1.1	1.16	1.09
ILMN_1367008	RGD1307234_predicted	1.11	1.24	1.58	1.19	1.18	1.16	1.09
ILMN_1650682	RGD1559904_predicted	-1.1	1.04	-1.13	1.13	1.28	1.16	1.09

ILMN_1372414	Ppp3ca	1.1	1.29	-1.14	1.26	1.36	1.17	1.09
ILMN_1353815	RGD1565815_predicted	-1.01	-1.26	-1.13	1.01	1.06	1.18	1.09
ILMN_1357813	RGD1564594_predicted	1.02	1.26	1.14	1.04	1.38	1.18	1.09
ILMN_1369356	Snip1	-1.18	1.02	1.22	-1.03	1.07	1.19	1.09
ILMN_1374312	RGD1305593_predicted	1.15	1.35	-1.07	1.13	1.15	1.2	1.09
ILMN_1359487	Mapk8ip3	-1.08	1.24	1.32	1.18	1.03	1.22	1.09
ILMN_1356539	Mif	1.21	1.08	-1.3	1.11	1.19	1.22	1.09
ILMN_1361702	Pik3c2a_predicted	1.08	1.04	1.29	1.15	1.43	1.22	1.09
ILMN_1373896	Tmed2	1.03	-1.11	1.19	-1.07	1.23	1.23	1.09
ILMN_1359460	RGD1311873	-1.07	1.26	-1.04	1.57	1.18	1.27	1.09
ILMN_1349607	LOC499464	-1.08	1.17	-1.2	-1.16	1.23	1.28	1.09
ILMN_1361406	RGD1563963_predicted	-1.07	-1.16	1.61	1.19	1.25	1.29	1.09
ILMN_1359605	RGD1309199	1.03	-1.33	-1.11	-1.53	1.12	1.3	1.09
ILMN_1370921	Cd47	-1.16	-1.04	1.16	1.1	1.13	1.3	1.09
ILMN_1350717	MGC93920	1.01	1.38	1.25	1.37	1.49	1.32	1.09
ILMN_1355566	Dad1	1.06	-1.1	-1.14	-1.07	1.41	1.33	1.09
ILMN_1650001	Ociad1	-1.03	1.15	-1.9	-1.03	1.5	1.33	1.09
ILMN_1362999	Cript	1.06	-1.44	-1.25	-1.34	1.24	1.34	1.09
ILMN_1370755	Txndc5_predicted	1.09	-1.29	1.48	1.35	1.71	1.65	1.09
ILMN_1358189	Exosc4_predicted	1.11	1.02	1.12	1.36	-1.33	-1.25	1.1
ILMN_1363639	LOC500533	-1.42	-1.21	-1.27	-1.22	-1.25	-1.25	1.1
ILMN_1375198	Ugt1a7	1.01	-1.43	1.16	-1.19	-1.29	-1.23	1.1
ILMN_1349185	Mst1	-1.18	1.08	1.14	-1.16	-1.01	-1.23	1.1
ILMN_1357618	RGD1560168_predicted	-1.06	1.16	-1.1	-1.03	-1.04	-1.18	1.1
ILMN_1370775	Mettl2_predicted	-1.01	1.08	1.12	1.27	-1.09	-1.17	1.1
ILMN_1360068	Cops6_predicted	1.11	-1.03	1.04	-1.03	-1.05	-1.14	1.1
ILMN_1358746	RGD1563463_predicted	1.03	-1.42	-1.94	-1.21	-1.03	-1.14	1.1
ILMN_1352222	RGD1306193	1.11	-1.25	-1.11	-1.22	-1.07	-1.12	1.1
ILMN_1370090	Atxn10	-1.04	-1.17	1.01	-1.22	-1.03	-1.09	1.1
ILMN_1361391	LOC360370	-1.07	-1.07	-1.29	-1.04	-1.11	-1.07	1.1
ILMN_1358770	Phf1	-1.1	1.17	-1.14	1	1.13	-1.06	1.1
ILMN_1371546	Aga	-1.04	-1.17	-1	-1.35	-1.03	-1.05	1.1
ILMN_1352924	Gstm5	1	-1.06	-1.19	1.1	1.05	-1.05	1.1
ILMN_1365778	RGD1309594	-1.06	-1.1	-1.1	-1.2	-1.24	-1.04	1.1
ILMN_1370940	RGD1309621	1.06	1.23	-1	1.15	-1.22	-1.04	1.1
ILMN_1350714	Oxr1	1.18	-1.19	-1.11	-1.61	-1.16	-1.04	1.1
ILMN_1363337	RGD1564370_predicted	-1.1	1.1	1.2	1.06	-1.04	-1.04	1.1
ILMN_1359936	RGD1564370_predicted	-1.1	1.1	1.2	1.06	-1.04	-1.04	1.1

ILMN_1368465	RGD1310430_predicted	-1.12	-1.18	-1.27	-1.54	-1.12	-1.03	1.1
ILMN_1356568	LOC316632	-1.06	-1.28	-1.02	-1.33	1.06	-1.03	1.1
ILMN_1351025	Agtrap	-1.07	1.22	1.11	1.24	1.08	-1.03	1.1
ILMN_1369803	Glmn	-1.11	1.22	1.4	1.43	-1.03	-1.01	1.1
ILMN_1353644	Atox1	1.13	-1.16	-1.93	-1.02	1.08	-1.01	1.1
ILMN_1358086	LOC497764	-1.06	1.01	1.06	-1.25	1.15	-1.01	1.1
ILMN_1358074	Npdc1	-1.11	1.28	1.11	1.11	1.21	-1.01	1.1
ILMN_1357406	Psmb1	1.07	1.12	-1.07	1.03	-1.03	-1	1.1
ILMN_1363536	RGD1311126_predicted	1.07	1.31	1.29	1.18	1.18	1	1.1
ILMN_1366257	Tead3	1.03	1.27	1.23	1.16	1.04	1.01	1.1
ILMN_1366175	Nudt2	1.14	1.22	-1.27	-1.13	-1.02	1.05	1.1
ILMN_1650266	LOC501934	1.14	-1.32	-1.09	1.12	1.12	1.05	1.1
ILMN_1363590	Mtmr9	-1.08	-1.14	-1.3	1.17	1.06	1.06	1.1
ILMN_1360744	Lsm16_predicted	-1.05	1.14	1.1	1.18	1.06	1.06	1.1
ILMN_1366929	Pex7	1.05	-1.24	1.06	-1.28	1.08	1.06	1.1
ILMN_1367758	RGD1309550	1.22	1.01	1.47	1.24	1.1	1.06	1.1
ILMN_1362127	Tpr	1.07	1.24	-1.05	1.27	1.1	1.06	1.1
ILMN_1357163	Sympk	-1.01	1.16	-1.1	1.28	1.12	1.06	1.1
ILMN_1364625	LOC499523	1.03	1.29	-1.02	1.24	1.21	1.06	1.1
ILMN_1357451	RGD1308665	1.09	1.23	-1.05	1.11	1.23	1.07	1.1
ILMN_1371311	RGD1309887_predicted	-1.16	1.03	-1.25	-1.09	1.37	1.07	1.1
ILMN_1371681	Rcn3_predicted	-1.11	1.11	1.27	-1.04	-1.15	1.08	1.1
ILMN_1367032	Tex9_predicted	1.05	1.46	1.22	1.23	1.08	1.08	1.1
ILMN_1365729	Slc7a5	1.34	1.36	1.12	1.67	1.12	1.08	1.1
ILMN_1376609	Sf4	1.09	1.08	1.37	1.15	1.16	1.08	1.1
ILMN_1359370	Adrm1	1.16	1.32	-1.1	1.33	1.22	1.08	1.1
ILMN_1363589	Chd8	-1.05	-1.04	-1.06	1.11	1.09	1.09	1.1
ILMN_1354598	RGD1564938_predicted	-1.17	-1.06	-1.39	-1.09	1.41	1.09	1.1
ILMN_1349169	Pus7l_predicted	1.04	1.05	1.44	1.12	1.01	1.1	1.1
ILMN_1373299	Mphosph10_predicted	1.08	1.41	1.36	1.57	1.08	1.1	1.1
ILMN_1354766	Psmb6	1.06	1.07	-1.05	1.26	1.19	1.1	1.1
ILMN_1353004	LOC367153	-1.02	1.16	1.24	1.09	1.09	1.11	1.1
ILMN_1350964	Usp14	1.1	1.14	1.36	1.45	1.1	1.11	1.1
ILMN_1376435	Dhdds	-1.06	-1	1.05	1.02	1.13	1.11	1.1
ILMN_1365964	Dvl1	-1.11	1.61	1.49	1.9	1.27	1.12	1.1
ILMN_2040788	RGD1562725_predicted	-1	1.14	-1	1.12	1.12	1.13	1.1
ILMN_1354268	RGD1562725_predicted	-1	1.14	-1	1.12	1.12	1.13	1.1
ILMN_1357873	RGD1304737_predicted	1.05	1.22	1.11	1.15	1.17	1.13	1.1

ILMN_1349377	Pik3c3	-1.02	-1.25	-1.33	-1.41	-1.05	1.14	1.1
ILMN_1353655	RGD1308750_predicted	1.09	1.43	1.72	1.56	1	1.14	1.1
ILMN_1349036	Bmsc-UbP	-1.13	-1.1	-1.36	-1.38	1.12	1.14	1.1
ILMN_1369989	RGD1549725	-1.14	1.06	-1.06	-1.16	1.16	1.14	1.1
ILMN_1356500	RGD1561821_predicted	1	-1.27	-1.22	1.08	1.17	1.14	1.1
ILMN_1357291	Gspt1	1.05	1.23	1.21	1.5	1.1	1.16	1.1
ILMN_1354357	Jmjd1c	1.08	1.03	-1.04	-1.06	1.12	1.16	1.1
ILMN_1649751	Trappc6b_predicted	1.02	1.25	1.25	1.15	1.18	1.16	1.1
ILMN_1356980	RGD1559895_predicted	-1.2	1.07	1.45	1.14	1.33	1.16	1.1
ILMN_1368816	LOC683522	-1.02	1.05	1.08	1.22	1.1	1.18	1.1
ILMN_1376353	Zfp297b	-1.13	-1.06	-1.26	-1.21	1.06	1.2	1.1
ILMN_1650818	Tpi1	-1.04	-1.06	1.17	1.16	1.24	1.2	1.1
ILMN_1371508	Rps14	-1.01	1.12	-1.04	1.12	1.16	1.21	1.1
ILMN_1350523	RGD1561453_predicted	1.04	1.05	-1.17	1.12	1.17	1.21	1.1
ILMN_1352480	Pitpna	-1.04	1.02	-1.02	1.29	1.14	1.22	1.1
ILMN_1349974	Ltv1	1.04	1.39	1.3	1.4	1.24	1.23	1.1
ILMN_1367101	Trim23	1.24	1.11	1.25	1.13	1.21	1.25	1.1
ILMN_1367652	Bst2	1.06	1.16	1.09	1.23	-1.08	1.26	1.1
ILMN_1361908	Zfp184_predicted	-1.03	1.34	1.17	1.45	1.1	1.26	1.1
ILMN_1361941	Lig4_predicted	1.02	1.05	-1.07	1.03	1.18	1.26	1.1
ILMN_1349453	Tbrg1	-1.1	1.13	1.01	-1.07	1.17	1.27	1.1
ILMN_1368994	Bbx_predicted	1.22	1.26	1.13	1.04	1.28	1.28	1.1
ILMN_1375139	RGD1561653_predicted	-1.03	1.34	-1.07	1.3	1.18	1.3	1.1
ILMN_1349016	Rabgef1_predicted	1.06	1.12	1.16	1.24	1.25	1.3	1.1
ILMN_1374451	Hirip5_predicted	-1.05	1.09	1.16	1.1	1.34	1.3	1.1
ILMN_1651052	RGD1312006_predicted	-1	-1.02	1.23	-1.16	1.39	1.32	1.1
ILMN_1649741	Samd4_predicted	1.33	1.42	1.19	1.72	1.08	1.33	1.1
ILMN_1353239	Eif4ebp1	1.31	1.43	1.07	1.36	1.31	1.33	1.1
ILMN_1362253	Eaf1_predicted	1.18	1.23	1.05	1.15	1.34	1.37	1.1
ILMN_1349961	RGD1305492_predicted	1.23	-1.03	1.07	1.01	1.45	1.4	1.1
ILMN_1369054	RGD1305492_predicted	1.23	-1.03	1.07	1.01	1.45	1.4	1.1
ILMN_1356683	Zfp598_predicted	1.15	1.69	1.58	1.75	1.53	1.4	1.1
ILMN_1358984	Dnajc13_predicted	-1.04	1.29	1.19	1.53	1.27	1.53	1.1
ILMN_1362245	Phlpp	1.15	1.82	1.25	1.52	1.3	1.54	1.1
ILMN_1373349	RGD1562399_predicted	-1.15	1.18	1.09	1.12	-1.05	1.55	1.1
ILMN_1374581	RGD1563144_predicted	1.2	1.49	1.53	1.2	2.07	1.89	1.1
ILMN_1357226	LOC498342	1.29	-1.14	-1.12	1.01	-1.25	-1.35	1.11
ILMN_1371236	RGD1304969_predicted	1.13	-1.13	1.02	-1.02	1.03	-1.22	1.11

ILMN_1357689	Mlycd	-1.05	-1.05	-1.08	1.15	1.01	-1.2	1.11
ILMN_1352572	Sema3f_predicted	-1.04	-1.08	-1.04	1.14	-1.07	-1.13	1.11
ILMN_1650385	RGD1307381	1.28	-1.04	1.3	1.03	-1.09	-1.11	1.11
ILMN_1348790	RGD1561764_predicted	1.1	-1.08	-1.01	-1.08	-1.29	-1.1	1.11
ILMN_1362066	Ndufb7_predicted	-1.04	-1.25	-2.07	-1.15	-1.04	-1.1	1.11
ILMN_1350298	LOC308996	-1.08	1.12	-1.17	1.19	1.05	-1.09	1.11
ILMN_1372440	Psmd11_predicted	1.11	1.17	1.21	1.22	-1.06	-1.07	1.11
ILMN_1351420	RGD1305807	-1.19	1.03	1.14	-1.35	-1	-1.07	1.11
ILMN_1358140	LOC310665	1.05	-1.11	-1.21	-1.25	-1.09	-1.06	1.11
ILMN_1370088	LOC311984	-1.13	1.08	1.17	-1.04	1.1	-1.06	1.11
ILMN_1363126	Ankra2	-1.16	-1.28	1.11	-1.02	1.08	-1.05	1.11
ILMN_1376754	Abcf3	-1.03	1.04	1.06	1.19	1.11	-1.04	1.11
ILMN_1373031	Them4	1.09	1.09	1.23	1.24	1.11	-1.03	1.11
ILMN_1359524	Imp3_predicted	1.18	1.19	1.08	1.24	-1.14	-1.01	1.11
ILMN_1373775	Bid	-1.06	1.15	1.91	1.27	1.09	-1.01	1.11
ILMN_1370587	Copg	-1.16	-1.31	-1.21	-1.18	-1.03	-1	1.11
ILMN_1355072	LOC299179	-1.06	1.2	-1.22	-1.04	-1.03	-1	1.11
ILMN_1362384	Rps8	1.05	1.03	1.09	1.02	1.05	-1	1.11
ILMN_1368078	RGD1306215_predicted	-1.06	1.27	1.11	1.09	1.14	-1	1.11
ILMN_1349323	MGC116327	-1.11	1.04	1.21	1.15	-1.02	1.01	1.11
ILMN_1371166	Dock9	1.02	-1.03	1.3	-1.05	-1	1.01	1.11
ILMN_1376549	Pipox	-1.17	1.05	-1.03	-1.17	1.03	1.01	1.11
ILMN_1373543	Zcrb1	-1.01	-1.15	-1.21	-1.01	1.03	1.01	1.11
ILMN_1366355	RGD1561815_predicted	1.01	-1.02	1.1	1.02	1.11	1.01	1.11
ILMN_1369157	LOC291665	1.07	-1.09	-1.4	-1.05	1.18	1.01	1.11
ILMN_1367494	Vdac3	1	1.02	1.09	-1.03	-1.04	1.04	1.11
ILMN_1367385	RGD1305264_predicted	1.17	1.15	-1.02	-1.09	-1.02	1.04	1.11
ILMN_1355936	Adss_predicted	1.06	1.12	1.21	1.03	1.13	1.04	1.11
ILMN_1356421	Bzw2	1.26	1.32	1.14	1.59	1.17	1.04	1.11
ILMN_1356157	Fbxl4_predicted	1.18	1.34	1.25	1.22	1.26	1.05	1.11
ILMN_1355117	RGD1305283_predicted	1.06	-1.02	-1.42	-1.09	1.12	1.06	1.11
ILMN_1363791	LOC498989	-1.25	-1.33	-2.32	-1.31	1.2	1.06	1.11
ILMN_1359703	LOC685245	1.22	1.18	1.19	1.31	-1.2	1.07	1.11
ILMN_1349955	LOC685245	1.22	1.18	1.19	1.31	-1.2	1.07	1.11
ILMN_1362843	Mkrn2	1.13	1.21	1.08	1.1	1.01	1.07	1.11
ILMN_1651075	Plrg1	-1.01	-1.09	1.05	-1.06	1.17	1.07	1.11
ILMN_1351439	Tm9sf2	-1.09	-1.35	-1.18	-1.2	1.09	1.08	1.11
ILMN_1369775	Vps36_predicted	1.03	1.56	1.44	1.34	1.17	1.1	1.11

ILMN_1358928	Fkbp4	1.05	-1.04	1.09	1.16	1.1	1.11	1.11
ILMN_1352378	Tfpt	1.16	1.08	1.21	1.23	1.29	1.11	1.11
ILMN_1363581	LOC688637	1.09	1.47	1.41	1.44	1.08	1.12	1.11
ILMN_1358647	Eif4e	-1.04	-1.07	1.09	1.22	1.13	1.12	1.11
ILMN_1368116	LOC367398	1	1.15	1.16	1.14	1.14	1.12	1.11
ILMN_1373125	Acbd3	-1.03	-1.14	-1.08	-1.14	1.15	1.12	1.11
ILMN_1364406	Npepps	-1.02	1.04	1.04	1.14	1.18	1.12	1.11
ILMN_1371151	Mrps18b	1.04	1.35	1.38	1.18	1.02	1.13	1.11
ILMN_1357322	LOC502313	1.18	1.25	1.1	1.21	1.11	1.13	1.11
ILMN_1367009	LOC497813	-1	1.08	-1	1.11	1.05	1.14	1.11
ILMN_1372704	RGD1565732_predicted	1.03	1.08	-1.07	1.06	1.22	1.14	1.11
ILMN_2038971	RGD1308874	1.25	-1.23	2.04	-1.01	-1.07	1.15	1.11
ILMN_1358574	RGD1359691	1	1.22	1.3	1.38	1.07	1.15	1.11
ILMN_1373196	Nle1_predicted	1.11	1.79	1.93	1.9	1.07	1.15	1.11
ILMN_1364189	RGD1311827	1.01	-1.01	1.18	1.09	1.14	1.17	1.11
ILMN_1372345	RGD1311899	-1.03	1.05	1.17	1.28	1.17	1.18	1.11
ILMN_1370012	LOC311382	-1.2	1.03	1.03	1.09	1.09	1.19	1.11
ILMN_1361091	RGD1306064_predicted	-1.06	-1	-1.01	-1.11	1.24	1.19	1.11
ILMN_1351540	RGD1304646_predicted	1.12	1.47	1.38	1.54	1.27	1.19	1.11
ILMN_1363248	Tmem8_predicted	1.14	1.32	1.12	1.26	1.34	1.19	1.11
ILMN_1353338	Snx27	1.1	1.17	1.12	1.29	1.01	1.21	1.11
ILMN_1376279	Fastk	-1.1	1.03	1.36	1.18	1.04	1.21	1.11
ILMN_1352214	RGD1561153_predicted	1.08	2.03	1.33	1.74	1.22	1.21	1.11
ILMN_1352587	Asxl2_predicted	-1.06	-1.09	1	1.03	1.23	1.21	1.11
ILMN_1361806	Dym_predicted	1.16	1.42	1.49	1.19	1.31	1.21	1.11
ILMN_1366676	LOC499749	-1.07	1.11	-1.15	1.57	1.17	1.23	1.11
ILMN_1361939	Rgs14	1.04	1.03	1.23	1.12	1.15	1.24	1.11
ILMN_1358315	RGD1565566_predicted	-1.01	1.16	1.2	1.26	1.23	1.24	1.11
ILMN_1360138	Sept8_predicted	1.02	-1.14	1.21	1.1	1.27	1.24	1.11
ILMN_1368251	Mrps18c_predicted	1.03	1.19	-1.45	1.23	1.3	1.24	1.11
ILMN_1366328	RGD1564315_predicted	1.06	1.14	1.25	1.08	1.16	1.25	1.11
ILMN_1372608	Mpzl1	1.08	1.37	1.21	1.13	1.32	1.25	1.11
ILMN_1365429	Fdx1	1.17	1.17	1.05	1.16	1.35	1.25	1.11
ILMN_1360589	Ibrdc3_predicted	1	1.26	-1.12	1.18	1.25	1.27	1.11
ILMN_1368060	LOC307731	-1.05	-1.4	1.46	1.12	1.52	1.27	1.11
ILMN_1364132	Scamp2	-1.2	-1.08	-1.44	-1.32	1.28	1.28	1.11
ILMN_1373680	MGC116266	-1.05	1.29	1.42	1.54	1.28	1.28	1.11
ILMN_1358477	Pdcl	1.16	-1	-1.01	1.01	1.29	1.28	1.11

ILMN_1365628	Snx24	1.03	1.35	1.22	1.4	1.28	1.33	1.11
ILMN_1370483	Ssr4	1.06	1.18	-1.05	1.3	1.44	1.34	1.11
ILMN_1368269	RGD1561111_predicted	-1.2	-1.59	1.57	-1.02	1	1.35	1.11
ILMN_1371322	Tmem11_predicted	1.01	1.38	1.15	1.48	1.28	1.39	1.11
ILMN_1360368	Golgb1	-1.08	-1.29	-1.07	-1.14	1.22	1.4	1.11
ILMN_1369640	Pawr	1.1	1.38	1.33	1.5	1.58	1.5	1.11
ILMN_1355986	Rab1	1.04	-1.12	1.26	1.13	1.49	1.58	1.11
ILMN_1350442	Sirt6	-1.01	1.45	1.33	1.14	1.62	1.72	1.11
ILMN_1368674	RGD1310931_predicted	-1.04	-1.53	-1.37	-1.78	-1.45	-1.49	1.12
ILMN_1370454	Aox1	1.12	1.03	1.11	-1.61	-1.21	-1.45	1.12
ILMN_1351428	Abcc3	1.35	-1.46	-2.24	-1.4	-1.46	-1.33	1.12
ILMN_1348976	Gss	-1.11	1.14	1.35	1.2	-1.26	-1.27	1.12
ILMN_1359346	Aph1b	1.03	-1.13	-1.06	-1.34	-1.18	-1.24	1.12
ILMN_1361984	Rgs19	1.03	1.07	-1.08	1.09	-1.05	-1.17	1.12
ILMN_1376322	Dgat1	-1.09	1.15	1.17	1.09	-1.19	-1.16	1.12
ILMN_1649832	Lyplal1_predicted	1.11	-1.16	1.06	-1.6	-1.04	-1.15	1.12
ILMN_1361113	Hspe1	1.23	1.19	-1.27	1.21	-1.27	-1.14	1.12
ILMN_1360453	Vamp8	-1.06	-1.26	-1.56	-1.49	-1.08	-1.14	1.12
ILMN_1352237	LOC306137	-1.11	1.09	1.03	1.36	-1.14	-1.1	1.12
ILMN_1362981	Cpd	-1.26	1.05	-1.15	1.02	1.08	-1.09	1.12
ILMN_1348845	RGD1559821_predicted	1.08	1.2	-1.35	1.06	1.14	-1.07	1.12
ILMN_1352826	Ppcs	1.18	1.18	1.49	1.38	-1.08	-1.06	1.12
ILMN_1371155	Cast	-1.01	1.01	-1.23	1.04	-1.06	-1.06	1.12
ILMN_1367015	RGD1311021_predicted	-1.12	-1.23	-1	-1.3	1.03	-1.06	1.12
ILMN_1375217	Cand1	-1.15	-1.27	-1.17	1.18	-1.16	-1.05	1.12
ILMN_1359799	RGD1310769_predicted	-1.18	-1.49	-1.43	-1.93	-1.07	-1.05	1.12
ILMN_1369614	RGD1307100	-1.22	-1.1	-1.34	-1.2	-1.07	-1.03	1.12
ILMN_1376592	Ptprf	1.09	1.03	-1.05	1.21	-1.02	-1.03	1.12
ILMN_1360122	Ubp1_predicted	-1.01	1.1	1.44	1.04	1.11	-1.03	1.12
ILMN_1373576	Stk3	1.02	1.07	1.06	1.12	1.11	-1.03	1.12
ILMN_1363072	Eif2ak1	-1.12	-1.04	-1.1	-1.37	-1.1	-1.02	1.12
ILMN_1365613	Tnfsf5ip1_predicted	1.13	-1.14	-1.05	-1.03	-1.13	-1.01	1.12
ILMN_1376324	Zfp96	-1.08	-1.01	1.13	1.15	1.02	-1.01	1.12
ILMN_1651025	Slc25a17_predicted	-1.02	1.03	-1.03	-1.57	-1.11	1	1.12
ILMN_1373690	RGD1309410_predicted	1.09	1.16	1.11	1.35	1.05	1	1.12
ILMN_1356223	Psmc2	-1.03	1.05	1.03	-1.03	1.16	1	1.12
ILMN_1354391	Ptpmt1	1.08	-1.03	1.12	-1.1	-1.11	1.01	1.12
ILMN_1367547	RGD1311552_predicted	-1.06	1.03	-1.1	-1.23	-1.05	1.01	1.12

ILMN_1356932	Geft	-1.07	1.81	1.15	1.45	1.09	1.01	1.12
ILMN_1366571	RGD1565584_predicted	-1.17	1.2	1.05	1.18	-1.13	1.02	1.12
ILMN_1363911	MGC94339	-1.18	-1.12	-1.31	-1.25	1.22	1.02	1.12
ILMN_1364063	Bcar1	-1.05	1.09	1	1.25	1.31	1.02	1.12
ILMN_1359814	Hbxap_predicted	-1.16	1.07	-1.3	-1.04	-1.24	1.03	1.12
ILMN_1369313	LOC688606	1.1	-1.05	-1.39	-1.37	-1.05	1.03	1.12
ILMN_1352636	Rnh1	-1.15	-1.05	1.02	-1.13	1.1	1.03	1.12
ILMN_1352116	Ogt	1.02	-1.06	1.23	-1	1.03	1.04	1.12
ILMN_1354551	RGD1565033_predicted	-1.05	-1.09	-1.1	-1.24	1.2	1.04	1.12
ILMN_1360726	D1bwg1363e	-1.11	1.27	-1.04	1.28	1.02	1.05	1.12
ILMN_1356281	Cnih_predicted	1.14	1.27	1.42	-1.02	1.05	1.05	1.12
ILMN_1357173	Cox5a	1.04	1.06	-1.15	1.1	1.06	1.05	1.12
ILMN_1358826	Nudt15_predicted	1.06	-1.04	-1.33	-1.14	1.26	1.05	1.12
ILMN_1372883	RGD1564300_predicted	1	1.31	1.26	1.19	1	1.07	1.12
ILMN_1359630	LOC679663	1.09	1.03	-1.47	1.08	1.16	1.07	1.12
ILMN_1372409	Psmd3	1.14	1.16	1.49	1.32	1.09	1.09	1.12
ILMN_1363047	RGD1563757_predicted	1.07	-1	1.1	1.3	1.14	1.09	1.12
ILMN_1352305	LOC293103	1.11	1.09	1.16	1.29	1.36	1.09	1.12
ILMN_1373027	Kras	1.05	1.09	1.33	1.27	1.2	1.11	1.12
ILMN_1651074	Rpo1-2	1.39	1.84	1.56	2.16	-1.07	1.12	1.12
ILMN_1364111	RGD1564820_predicted	-1.06	-1.05	1.07	1.13	-1.05	1.12	1.12
ILMN_1374330	Xab2	1.05	-1.09	-1.22	-1	1.11	1.12	1.12
ILMN_1354375	Zfp295_predicted	-1.08	1.04	1.15	-1.02	1.17	1.12	1.12
ILMN_1371901	Clk4	-1.12	1.16	1.11	1.33	1.19	1.12	1.12
ILMN_1356064	Sall1_predicted	1.21	1.04	-1.14	-1.03	1.04	1.13	1.12
ILMN_1368026	LOC499136	1.1	1.08	1.04	1.22	1.44	1.13	1.12
ILMN_1357860	Bxdc5	1.1	1.1	1.03	1.15	1.05	1.14	1.12
ILMN_1349497	Bxdc5	1.1	1.1	1.03	1.15	1.05	1.14	1.12
ILMN_1371928	Cc2d1b	-1.03	1.16	1.29	1.22	1.25	1.14	1.12
ILMN_1370897	Hace1_predicted	1.09	1.2	1.13	1.02	1.13	1.15	1.12
ILMN_1362499	RGD1563570_predicted	1.1	1.09	-1.13	1.15	1.2	1.15	1.12
ILMN_1352034	RGD1560581_predicted	-1.03	-1.26	-1.15	1.09	1.24	1.15	1.12
ILMN_1356886	LOC500104	1.04	-1.25	-1.07	1.11	1.31	1.15	1.12
ILMN_1370272	Miz1	1.05	1.08	1.29	1.29	1.05	1.16	1.12
ILMN_1649962	Tmem93_predicted	1.16	1.31	-1.03	1.55	1.16	1.17	1.12
ILMN_1369216	RGD1565022_predicted	1	1.28	1.53	1.48	1.31	1.17	1.12
ILMN_1365881	RGD1307179_predicted	1.01	-1	-1.11	1.18	1.14	1.2	1.12
ILMN_1360804	LOC364105	1.2	1.37	-1.69	1.31	1.22	1.2	1.12

ILMN_1350253	Commd7_predicted	-1.02	1.12	1.06	1.15	1.32	1.2	1.12
ILMN_1356805	RGD1559862_predicted	-1.02	1.1	1.17	1.16	1.23	1.22	1.12
ILMN_1372030	Adprhl2_predicted	-1	1.03	-1.06	1.26	1.12	1.24	1.12
ILMN_1372974	Phc3_predicted	1.01	1.24	1.15	1.04	1.17	1.26	1.12
ILMN_1376704	Degs1	-1.08	1.14	1.41	1.27	1.28	1.29	1.12
ILMN_1360346	Smad2	1.13	1.21	1	1.42	1.25	1.3	1.12
ILMN_1370230	RGD1560513_predicted	1.09	-1.02	-1.08	1.22	1.32	1.3	1.12
ILMN_1363212	LOC498424	1.1	-1.1	1.12	-1	1.21	1.31	1.12
ILMN_1352220	Cfl2_predicted	-1.11	-1.24	1.14	-1	1.44	1.32	1.12
ILMN_1351803	Cog6	-1.09	1.09	-1.05	1.16	1.1	1.33	1.12
ILMN_1349843	LOC687406	1.18	-1.05	1.59	1.37	1.2	1.33	1.12
ILMN_1348824	LOC687406	1.18	-1.05	1.59	1.37	1.2	1.33	1.12
ILMN_1351978	Gdap2	1.17	1	1.04	1.25	1.37	1.33	1.12
ILMN_1369508	Amhr2	1.19	1.2	1.08	1.16	1.32	1.36	1.12
ILMN_1359067	Zfp265	-1.19	1.09	1.33	1.5	1.33	1.37	1.12
ILMN_1371948	Topors_predicted	-1.02	1.31	1.06	1.11	1.38	1.38	1.12
ILMN_1349400	Sec24a_predicted	1.14	1.95	1.53	2.03	1.24	1.39	1.12
ILMN_1351855	RGD1564762_predicted	1.26	1.08	1.04	1.18	1.35	1.39	1.12
ILMN_1351542	Rpl35a	1.13	1.23	-1.11	1.36	1.39	1.42	1.12
ILMN_1350093	Sec22l1	1.03	1.07	1.17	1	1.58	1.43	1.12
ILMN_1364310	RGD1308049_predicted	1.1	1.48	1.23	1.32	1.15	1.44	1.12
ILMN_1371396	Ccnc	1.16	1.33	1.78	1.49	1.42	1.44	1.12
ILMN_1352444	Tsnax	1.14	1.31	1.66	1.71	1.54	1.44	1.12
ILMN_1649737	RGD1562694_predicted	1.1	1.6	2.2	1.38	1.19	1.49	1.12
ILMN_1367011	B4galt3	1.06	1.37	1.39	1.49	1.47	1.49	1.12
ILMN_1650851	Golph3	1.23	1.15	1.42	1.03	1.4	1.61	1.12
ILMN_1363547	Ctse	1.04	-1.12	-1.09	-1.16	-1.2	-1.43	1.13
ILMN_1360786	Fkbp8	1.04	-1.03	-1.62	-1.11	-1.04	-1.27	1.13
ILMN_1362177	Stxbp3	-1.28	-1.25	-1.2	-1.41	-1.24	-1.21	1.13
ILMN_1353110	RGD1564454_predicted	1.05	-1.17	1.15	-1.14	-1.01	-1.18	1.13
ILMN_1364498	Cdk5	-1.07	1.29	1.13	1.26	-1.03	-1.17	1.13
ILMN_1368110	RGD1563216_predicted	1.07	1.01	-1.04	-1.04	1.01	-1.17	1.13
ILMN_1356689	Tnip2	1.07	1.27	1.63	1.23	-1.05	-1.14	1.13
ILMN_1360493	LOC499148	1.05	1.02	1.13	1.05	1.04	-1.12	1.13
ILMN_1356317	LOC498177	1.02	-1.07	1.1	1.33	1.07	-1.09	1.13
ILMN_1360040	Nudt14_predicted	-1.13	-1.37	-1.17	-1.55	1.07	-1.05	1.13
ILMN_1356475	Gak	1.05	1.13	1.1	1.09	1.06	-1.03	1.13
ILMN_1650859	LOC292282	-1.11	-1.02	1.02	-1.16	1.05	-1.01	1.13

ILMN_1365884	Gpr89_predicted	-1.03	-1.02	1.02	-1.01	1.07	-1.01	1.13
ILMN_1370319	RGD1310861	-1.24	-1.09	1.18	1.04	-1.05	-1	1.13
ILMN_1374365	Tmed4_predicted	1.1	1.31	1.7	1.55	1.15	-1	1.13
ILMN_1366996	Epdr2	1.19	1.28	1.26	1.12	-1.16	1.02	1.13
ILMN_1374833	Lenep	1.06	1.15	1.33	1.26	1	1.02	1.13
ILMN_1366238	Pcnx	1.02	1.12	1.29	1.09	1.04	1.02	1.13
ILMN_1366159	Psmd6	1	-1.1	1.04	1.02	1.07	1.02	1.13
ILMN_1357336	Psmb3	1.12	1.05	-1.04	1.14	-1.05	1.03	1.13
ILMN_1364412	Otud6b_predicted	1.32	1.08	1.73	1.22	1.21	1.03	1.13
ILMN_1365110	RGD1310351_predicted	-1.14	-1.1	-1.13	-1.19	-1.03	1.04	1.13
ILMN_2040706	LOC363429	1.01	1.16	-1.06	1.23	1.06	1.04	1.13
ILMN_1366022	Sec22l2	-1.08	-1.16	-1.01	-1.21	1.1	1.05	1.13
ILMN_1371618	Phlda1	-1.33	1.49	-1.2	1.41	1.24	1.05	1.13
ILMN_1370528	Nsfl1c	-1.03	1.04	1.36	1.25	-1.08	1.08	1.13
ILMN_1372480	Rpl22	1.19	1.2	-1.3	1.17	1.14	1.08	1.13
ILMN_1371108	Shkbp1_predicted	-1.2	1.11	1.42	1.24	1.07	1.09	1.13
ILMN_1373608	RGD1560934_predicted	1.09	1.29	1.37	1.24	1.13	1.09	1.13
ILMN_1367625	LOC501609	-1.02	1.02	1.03	1.05	-1.06	1.1	1.13
ILMN_1358834	Senp6_predicted	1.11	1.13	1.26	1.29	1.2	1.1	1.13
ILMN_1369433	Aadat	-1.03	1.05	1.03	-1.06	1.35	1.1	1.13
ILMN_1368520	Zbtb8os_predicted	-1.06	-1.08	-1.22	-1.13	1.01	1.11	1.13
ILMN_1358095	RGD1561181_predicted	1.1	1.09	-1.14	1.07	1.11	1.11	1.13
ILMN_1351773	RGD1561181_predicted	1.1	1.09	-1.14	1.07	1.11	1.11	1.13
ILMN_1367238	MGC94335	-1.03	1.18	1.05	1.2	1.11	1.11	1.13
ILMN_1650886	LOC499122	-1.14	1.2	1.06	1.2	1.04	1.12	1.13
ILMN_1360549	Dyrk2_predicted	1.04	1.44	1.43	1.54	-1.01	1.13	1.13
ILMN_1355712	Fbxw8_predicted	1.13	-1.04	-1.23	1.03	1.01	1.13	1.13
ILMN_1370728	Dctn1	-1.05	1.14	1.31	1.01	1.15	1.15	1.13
ILMN_1355258	Slc35b3_predicted	1.01	-1.21	1.02	-1.18	1.01	1.16	1.13
ILMN_1351184	Psen1	1.05	-1.04	-1.07	-1.04	1.14	1.16	1.13
ILMN_1374597	Yipf1	-1.11	1.31	1.47	1.08	1.16	1.16	1.13
ILMN_1365125	Csnk1g3	1.1	1.29	1.19	1.18	1.22	1.17	1.13
ILMN_1370626	Csk_predicted	-1.14	1.06	1.07	1.04	-1.04	1.18	1.13
ILMN_1352738	LOC497743	1.27	1.2	1.1	1.19	1.22	1.19	1.13
ILMN_1372724	RGD1562136_predicted	1.3	1.59	1.79	1.96	1.29	1.2	1.13
ILMN_1350631	RGD1359339	-1	1.21	1.38	1.59	1.08	1.22	1.13
ILMN_1361658	LOC679036	-1.01	1.15	1.3	1.23	1.11	1.24	1.13
ILMN_1351937	RGD1564103_predicted	-1.07	1.07	1.01	1.07	1.11	1.25	1.13

ILMN_1365801	Morf4l2	-1.22	1.02	1.07	-1	1.26	1.26	1.13
ILMN_1366711	LOC500894	1.08	1.42	1.32	1.53	1.16	1.27	1.13
ILMN_1358412	Pip5k2c	-1.07	1.15	-1.16	1.21	1.25	1.27	1.13
ILMN_1359991	Alg2	1.03	1.18	1.18	-1.05	1.32	1.3	1.13
ILMN_1363139	LOC364048	1.26	1.06	-1.12	1.32	1.37	1.3	1.13
ILMN_1351407	Atxn3	1.08	1.23	1.13	1.25	1.01	1.31	1.13
ILMN_1354226	RGD1563689_predicted	-1.07	1.15	1.25	1.15	1.2	1.31	1.13
ILMN_1361019	Nat9_predicted	1.01	1.07	1.04	-1.04	1.33	1.31	1.13
ILMN_1367378	Sgtb	1.05	1.26	1.26	1.18	1.58	1.32	1.13
ILMN_1368697	Foxk2_predicted	1.13	1.19	1.27	1.4	1.43	1.33	1.13
ILMN_1375342	Axot_predicted	1.27	1.16	1.3	1.13	1.14	1.34	1.13
ILMN_1356407	Sipa1l1	-1.02	1.15	-1.02	1.18	1.3	1.34	1.13
ILMN_1376446	Zbtb17	1.02	1.42	1.22	1.29	1.24	1.35	1.13
ILMN_1373582	Tcfe3_predicted	1.1	1.08	1.23	1.03	1.45	1.37	1.13
ILMN_1352633	Kif3b_predicted	-1.03	1.36	1.18	1.36	1.46	1.37	1.13
ILMN_1371106	Rkhd1_predicted	1.03	1.4	1.46	1.35	1.36	1.38	1.13
ILMN_1359958	RGD708449	-1.27	1.14	1.08	-1.05	1.44	1.39	1.13
ILMN_1364579	Gnai3	1.12	1.18	1.4	1.24	1.47	1.44	1.13
ILMN_1366994	Rab5a	1.08	1.02	1.28	1.03	1.66	1.54	1.13
ILMN_1354803	Arcn1	1.05	1.27	1.21	1.11	1.68	1.68	1.13
ILMN_1650644	Fbxo30	1.17	1.69	1.42	1.56	1.79	1.88	1.13
ILMN_1374544	Nars	1.09	1.14	1.42	1.26	1.81	1.93	1.13
ILMN_1367864	Ctsh	-1.2	-1.41	-1.17	-1.52	-1.25	-1.51	1.14
ILMN_1363206	Mrpl36_predicted	-1.2	-1.27	1.13	-1.05	-1.17	-1.27	1.14
ILMN_1367076	Iars2_predicted	-1.02	-1.04	-1.31	-1.1	-1.24	-1.22	1.14
ILMN_1365423	RGD1563422_predicted	-1.01	-1.07	-1.18	-1.06	1.06	-1.22	1.14
ILMN_1365131	RGD1560220_predicted	1.04	-1.23	-1.99	-1.23	-1.08	-1.2	1.14
ILMN_1354664	G3bp	1.02	1.14	-1.02	1.15	-1.19	-1.17	1.14
ILMN_1357759	Galt	-1.01	1.12	1.22	1.03	-1.23	-1.15	1.14
ILMN_1353905	RGD1307760	1.11	-1.21	-1.12	-1.06	-1.07	-1.14	1.14
ILMN_1362990	Dynll1	-1.08	-1.13	-1.44	1.07	1.01	-1.12	1.14
ILMN_1371130	Glb1_mapped	-1.06	1.14	1.33	1.17	1.01	-1.12	1.14
ILMN_1651101	Leng1_predicted	-1.21	1.13	-1.12	-1.02	-1.1	-1.11	1.14
ILMN_1375080	Chmp6_predicted	-1.12	-1.14	1.03	-1.06	1.08	-1.11	1.14
ILMN_1356536	Lactb_predicted	-1.06	-1	1.11	1.08	1.11	-1.02	1.14
ILMN_1364092	Bbs4_predicted	1.04	1.19	1.18	1.12	1.19	-1.02	1.14
ILMN_1361771	RGD1309216	1.02	1.03	1.33	1.2	-1.15	-1	1.14
ILMN_1362182	Atp5h	1.08	-1.04	-1.58	1.13	1.06	1.01	1.14

ILMN_1357395	Tspan31	-1.05	1.09	1.16	-1.03	1.23	1.01	1.14
ILMN_1352398	Hrb2_predicted	-1.05	1.01	1.21	1.2	-1.09	1.04	1.14
ILMN_1351068	RGD1309829_predicted	-1.01	1.09	-1	1.29	1.11	1.04	1.14
ILMN_1369578	LOC501832	-1.19	-1.1	1.07	1.09	1.13	1.04	1.14
ILMN_1362447	Med25_predicted	-1.09	1.12	1.31	1.38	-1.02	1.06	1.14
ILMN_1358039	Rda279	1.1	1.22	1.18	1.24	1.01	1.06	1.14
ILMN_1369279	RGD1561086_predicted	1.01	1.06	-1.02	1.16	1.08	1.08	1.14
ILMN_1363360	MGC72996	1.11	1.39	1.13	1.26	1.24	1.09	1.14
ILMN_1364060	Rhog	-1.08	1.09	-1.02	-1.15	1.07	1.1	1.14
ILMN_1369669	Mpp5_predicted	1.01	-1.14	-1.15	-1.12	1.12	1.1	1.14
ILMN_1357009	Cebpz_predicted	1.12	1.54	1.24	1.6	1.17	1.1	1.14
ILMN_1650084	RGD1561195_predicted	-1.04	1.07	-1.07	1.18	1.15	1.12	1.14
ILMN_1368759	Rexo2	1.3	1.17	1.1	1.2	1.15	1.14	1.14
ILMN_1361388	Hectd2_predicted	1.14	1.24	1.24	1.36	1.16	1.14	1.14
ILMN_1361260	LOC499699	1.1	1.01	1.26	-1.2	1.05	1.15	1.14
ILMN_1376683	Smarcd3	1.06	1.3	1.29	1.46	1.25	1.15	1.14
ILMN_1376859	Gga1	-1.08	1.36	-1.11	1.22	1.12	1.16	1.14
ILMN_1366783	Vps13d_predicted	1.29	1.1	-1.02	1.02	1.15	1.16	1.14
ILMN_1376966	Slc35a3	-1.06	-1.03	1.17	1.12	1.14	1.17	1.14
ILMN_1360590	Rplp1	1.07	1.08	-1.03	1.09	1.15	1.18	1.14
ILMN_1351207	Uba52	1.16	1.2	-1.26	1.23	1.22	1.18	1.14
ILMN_1353748	LOC360568	1.11	1.06	1.35	1.25	1.27	1.19	1.14
ILMN_1374667	LOC309854	1.18	1.16	1.24	1.05	1.09	1.2	1.14
ILMN_1354478	Csnk1a1	1.08	1.19	1.18	1.12	1.11	1.2	1.14
ILMN_1373769	Wdsof1_predicted	-1.07	1.1	1.23	1.2	1.25	1.21	1.14
ILMN_1351676	Esam	1.05	1.22	1.34	1.68	1.17	1.22	1.14
ILMN_1371403	Socs4_predicted	1.33	1.33	1.15	1.5	-1	1.26	1.14
ILMN_1350582	RGD1559672_predicted	1.07	-1.03	1.12	1.08	1.21	1.26	1.14
ILMN_1374199	Giot1	-1.31	-1.07	-1.07	-1.11	1.05	1.27	1.14
ILMN_1367121	Secisbp2	-1.14	-1	1.12	1.26	1.08	1.27	1.14
ILMN_1376655	Katna1	-1.01	1.31	1.28	1.34	1.43	1.27	1.14
ILMN_1351310	Nckap1	1.19	1.19	1.27	1.22	1.2	1.28	1.14
ILMN_1357717	Sar1b	-1.11	1.02	-1.14	-1.42	1.28	1.29	1.14
ILMN_1376540	Spata7	-1.11	1.49	1.39	1.35	1.29	1.29	1.14
ILMN_1365886	Cd164	-1.1	1.07	1.24	1.09	1.33	1.29	1.14
ILMN_1369466	Fpgt	-1	1.1	1.1	1.21	1.26	1.3	1.14
ILMN_1361811	Gdi2	-1.1	1.29	1.34	1.26	1.34	1.31	1.14
ILMN_1650556	Rbm18_predicted	1.04	1.18	1.3	1.17	1.41	1.31	1.14

ILMN_1371491	Metrnl	-1.04	1.21	-1.07	1.48	1.24	1.33	1.14
ILMN_1650392	RGD1307598	1.03	1.2	1.19	1.28	1.39	1.33	1.14
ILMN_1376487	Ubap1	1.02	1.05	1.16	1.08	1.09	1.34	1.14
ILMN_1349701	Bag5	1.07	1.41	1.46	1.49	1.29	1.34	1.14
ILMN_1354206	Tuba1	1.17	-1.33	-1.18	-2.01	1.4	1.35	1.14
ILMN_1360836	Cabp1	1.01	1.27	1.61	1.56	1.25	1.36	1.14
ILMN_1368255	Abce1	1.06	1.18	1.08	1.27	1.17	1.37	1.14
ILMN_1368188	LOC690262	1.2	1.34	1.62	1.33	1.27	1.37	1.14
ILMN_1359745	LOC498266	-1.13	-1.26	-1.24	-1.44	1.44	1.37	1.14
ILMN_1361037	Irak3_predicted	1.41	1.19	1.54	1.27	1.4	1.39	1.14
ILMN_1364421	Mcart2_predicted	1.02	1.32	1.07	1.1	1.11	1.4	1.14
ILMN_1362809	Sbds	1.02	-1.1	-1.08	1	1.14	1.42	1.14
ILMN_1649801	RGD1563050_predicted	1.07	1.21	1.24	1.19	1.59	1.42	1.14
ILMN_1366460	MGC109455	-1.04	1.2	1.18	1.15	1.39	1.44	1.14
ILMN_1371686	Vegfa	1.36	1.82	1.76	1.75	1.29	1.46	1.14
ILMN_1354548	Tex10_predicted	1.03	1.26	1.21	1.49	1.25	1.47	1.14
ILMN_1351568	Kbtbd2_predicted	1.18	1.16	1.21	-1.04	1.44	1.49	1.14
ILMN_1365079	Smc5l1_predicted	1.18	1.82	1.6	1.85	1.53	1.6	1.14
ILMN_1650033	Repin1	1.16	1.5	1.37	1.35	1.36	1.63	1.14
ILMN_1376346	Pdia6	-1.04	-1.13	1.25	-1.02	1.77	1.76	1.14
ILMN_1367529	Apoe	-1.22	-1.1	1.01	-1.19	-1.67	-1.78	1.15
ILMN_1360056	RGD1311723_predicted	1.07	-1.18	-1.71	-1.02	-1.28	-1.26	1.15
ILMN_1349238	Tgfa	1.13	1.18	1.3	1.4	-1.2	-1.2	1.15
ILMN_1370875	Card9	1.21	1.3	1.51	1.41	-1.02	-1.14	1.15
ILMN_1356507	Ibtk_predicted	1.07	-1.01	1.05	-1.06	1.07	-1.11	1.15
ILMN_1358686	Rpl5	1.31	1.28	1.63	1.42	-1.08	-1.08	1.15
ILMN_1360050	LOC500391	1.08	1.06	-1.18	1.1	1	-1.06	1.15
ILMN_1350269	Hfe	-1.05	-1.08	-1.19	-1.07	1.04	-1.06	1.15
ILMN_1363587	Slc25a19	1.29	1.39	1.24	1.58	-1.15	-1.04	1.15
ILMN_1365357	Plekha3	1.03	-1.05	-1.14	-1.06	-1.11	-1.03	1.15
ILMN_1370142	Arfgap1	1.11	1.51	1.76	1.56	-1.02	-1.02	1.15
ILMN_1372596	Prdx1	1.08	-1.28	-1.1	-1.13	1.04	-1.02	1.15
ILMN_1649855	RGD1306660_predicted	-1.03	1.03	-1.06	1.02	1.12	-1.02	1.15
ILMN_1352127	Znhit1_predicted	-1.17	-1.24	-1.47	-1.23	-1.07	-1.01	1.15
ILMN_1356731	LOC499131	1.13	-1.17	-1.93	-1.22	1.09	1.01	1.15
ILMN_1373622	RGD1305534_predicted	-1.06	1.05	-1.04	1.09	1.07	1.03	1.15
ILMN_1353188	RGD1306495	1.14	-1.24	1.18	-1.56	1.28	1.04	1.15
ILMN_1355732	Txndc9	-1.08	1.01	1.06	-1.01	-1.03	1.05	1.15

ILMN_1372041	Grtp1_predicted	1.15	1.37	1.52	1.88	1.16	1.05	1.15
ILMN_2039579	Rnf4	-1.06	-1.05	1.11	-1.07	-1.04	1.06	1.15
ILMN_1374938	Arhgef5	-1.05	1.5	1.19	1.1	1.03	1.06	1.15
ILMN_1366324	Pdrg1	1.13	1.1	1.2	1.12	1.08	1.06	1.15
ILMN_1354690	Thap4	1.06	1.3	1.05	1.35	1.12	1.07	1.15
ILMN_1353104	Pi4k2a	1.01	1.19	1.14	1.31	1.24	1.07	1.15
ILMN_1356874	Hsd17b8	1.12	1.18	1.42	1.17	1.26	1.07	1.15
ILMN_1375784	Fmo4	-1.03	-1.11	-1.1	-1.18	1.04	1.08	1.15
ILMN_1348963	RGD1305166_predicted	1.01	1.01	1.01	-1.06	1.07	1.09	1.15
ILMN_1360258	Slc39a6	1.13	1.23	1.12	1.33	-1.04	1.11	1.15
ILMN_1364500	Hspcal3_predicted	1.07	-1.16	-1.11	1.32	1.15	1.12	1.15
ILMN_1351006	Tpt1	-1.01	-1	-1.01	1.03	1.18	1.12	1.15
ILMN_1354185	Nolc1	1.05	-1.13	1.61	1.16	1.09	1.13	1.15
ILMN_1360115	MGC94142	1.03	1.15	-1.06	1.15	1.15	1.13	1.15
ILMN_1370925	RGD1309385_predicted	1.04	-1.17	-1.2	-1.17	1.13	1.14	1.15
ILMN_1349437	RGD1307603_predicted	-1.06	2.13	1.58	1.83	1.32	1.14	1.15
ILMN_1350695	Psmd13_predicted	1.09	-1.11	1.38	1.03	1.05	1.15	1.15
ILMN_1352562	LOC366910	-1.02	-1.15	1.01	1.05	1.15	1.15	1.15
ILMN_1358584	Nufip1	1.01	1.41	1.55	1.64	1.25	1.16	1.15
ILMN_1360637	Prg4_predicted	1.2	1.21	1.3	1.29	2.07	1.16	1.15
ILMN_1366169	RGD1565891_predicted	-1.05	-1.43	-1.13	1.02	1.11	1.18	1.15
ILMN_2040197	RGD1305052_predicted	1.04	1.48	1.48	1.39	1.44	1.19	1.15
ILMN_1367235	Rpl27	1.15	1.28	-1.02	1.18	1.17	1.2	1.15
ILMN_1349990	Tcf7_predicted	1.01	1.62	1.24	1.73	1.16	1.21	1.15
ILMN_1352939	Fbxl6	1.04	1.88	2.25	1.84	1.09	1.22	1.15
ILMN_1356564	Farslb	1.18	1.18	1.45	1.3	1.24	1.24	1.15
ILMN_1367418	Znf142_predicted	1.12	1.29	1.38	1.48	1.14	1.25	1.15
ILMN_1361051	LOC498815	-1.1	1.34	1.16	1.21	1.12	1.26	1.15
ILMN_1355332	Eif3s3	-1.01	1.43	1.67	1.39	1.16	1.26	1.15
ILMN_1357385	Ncdn	1.1	1.07	-1.14	1.24	1.25	1.26	1.15
ILMN_1349633	Zfp358_predicted	1	1.64	1.94	1.38	1.22	1.27	1.15
ILMN_1361841	RGD1559574_predicted	-1.05	1.29	1.55	1.24	1.27	1.28	1.15
ILMN_1355382	Isrip	1	1.3	1.65	1.39	1.27	1.29	1.15
ILMN_1364112	Ldha	-1.1	-1.15	1.3	1.25	1.51	1.29	1.15
ILMN_2040763	Sept6_predicted	1.15	1.07	1.13	1.1	1.33	1.3	1.15
ILMN_1350256	Usp36_predicted	1.11	1.43	1.58	1.45	1.42	1.31	1.15
ILMN_1374248	DERP6	1.08	1.12	1.2	1.32	1.27	1.33	1.15
ILMN_1367288	Pqlc1	1.06	1.66	1.77	2.11	1.37	1.34	1.15

ILMN_1355511	Med28_predicted	-1.12	-1.03	-1.15	-1.05	1.29	1.35	1.15
ILMN_1357411	Grn	-1.11	-1.1	-1.05	-1.06	1.21	1.4	1.15
ILMN_1365258	Trim46_predicted	1.3	1.78	1.34	1.67	1.48	1.49	1.15
ILMN_1373686	Gnrh1	-1.08	-1.1	-1.2	1.06	1.45	1.52	1.15
ILMN_1350349	Sox4_predicted	1.01	1.94	-1.24	1.46	1.77	1.63	1.15
ILMN_1356156	RGD1311955_predicted	1.07	1.38	1.27	1.34	1.72	1.83	1.15
ILMN_1371014	Itih3	-1.05	-1.79	1.02	-1.3	1.11	-1.78	1.16
ILMN_1351312	LOC366673	-1.07	-1.43	-1.11	-1.37	-1.2	-1.35	1.16
ILMN_1349873	Cd302	1	1.02	-1.2	-1.49	-1.14	-1.31	1.16
ILMN_1358975	Creld1	1.09	1.56	1.21	1.02	-1.07	-1.23	1.16
ILMN_1373278	Hla-dmb	1.02	1.19	1.05	-1.08	-1.08	-1.2	1.16
ILMN_1361546	Hsd17b7	-1.08	-1.36	-1.2	-1.12	-1.06	-1.19	1.16
ILMN_1359420	Chchd5_predicted	-1	1	-1.37	1.05	-1.15	-1.17	1.16
ILMN_1358848	Slc38a3	1.13	1.16	1.05	1.13	-1.1	-1.16	1.16
ILMN_1366390	Tmem15_predicted	-1	1.02	1.2	1.23	-1.06	-1.16	1.16
ILMN_1355578	Cltb	1.13	-1.27	-1.66	-1.17	1.05	-1.16	1.16
ILMN_1359507	Acaa1	-1.04	-1.03	1.04	-1.47	-1.04	-1.15	1.16
ILMN_1362465	LOC290964	-1.02	-1.17	-1.29	-1.37	-1.35	-1.14	1.16
ILMN_1650323	Vps33b	-1.01	1.09	1.17	1.17	-1.02	-1.13	1.16
ILMN_1349298	Mrpl52_predicted	1.05	1.07	-2	-1.11	1	-1.11	1.16
ILMN_1363223	Stx7	-1.04	-1.07	-1.16	-1.13	1.09	-1.11	1.16
ILMN_1371406	Prlph	-1	-1.07	1.01	1.1	-1.19	-1.08	1.16
ILMN_1366503	RGD1562272_predicted	1.1	-1.03	1.14	-1.11	1.08	-1.04	1.16
ILMN_1374114	Gclm	2	-1.05	-1.13	1.05	-1.18	-1.03	1.16
ILMN_1363348	Nol8_predicted	1.39	1.41	1.36	1.56	1.02	-1.03	1.16
ILMN_1364372	Rps6	-1.02	-1.04	1.08	-1.02	1.04	-1.03	1.16
ILMN_1370971	Mgst1	-1.09	-1.14	-1.11	-1.28	-1.07	-1.01	1.16
ILMN_1376774	Uros	-1.06	-1.31	-1.16	-1.47	1.02	-1	1.16
ILMN_1371898	LOC313658	1.13	-1.17	-1.2	-1.04	-1.25	1	1.16
ILMN_1650578	Rp9h_predicted	1.01	-1.01	-1.06	1.24	-1.16	1.02	1.16
ILMN_1354053	Pkd1	1.07	1.09	-1.06	1.29	1.18	1.02	1.16
ILMN_1357468	Scly	-1.01	1.1	1.27	1.14	1.2	1.02	1.16
ILMN_1363646	RGD1308371_predicted	-1.06	-1.03	-1.09	-1.05	1.15	1.04	1.16
ILMN_1356629	LOC498544	1.41	1.35	-1.03	1.3	1.13	1.06	1.16
ILMN_1370343	RGD1563601_predicted	-1.11	-1	1.21	1.16	1.29	1.07	1.16
ILMN_2040221	RGD1565170_predicted	1.08	1.36	1.04	1.31	1.22	1.09	1.16
ILMN_1357680	RGD1309016	1.02	1.55	1.48	1.84	1.1	1.1	1.16
ILMN_2040277	RGD1308430_predicted	-1.02	1.02	1.07	1.11	1.13	1.1	1.16

ILMN_1358602	RGD1307696_predicted	1.09	1.17	1.32	-1.01	1.27	1.1	1.16
ILMN_1376703	Mapkap1	1.15	1.07	1.22	1.11	1.07	1.11	1.16
ILMN_1363583	Ube2q2_predicted	1.49	1.23	1.69	1.41	1.15	1.12	1.16
ILMN_1350524	Cttn	-1.18	1.13	1.95	1.45	1.09	1.13	1.16
ILMN_1352330	LOC498557	-1	-1.06	1.1	1.04	1.11	1.16	1.16
ILMN_1351451	RGD1307343	1.07	1.1	-1.12	1.04	1.13	1.16	1.16
ILMN_1353600	Ciapin1	1.27	1.24	1.39	1.21	1.15	1.16	1.16
ILMN_1361283	Timm10	1.23	1.47	-1.02	1.74	1.26	1.16	1.16
ILMN_1375017	Gtf2e2_predicted	1.05	1.26	1.4	1.22	1.29	1.16	1.16
ILMN_1367536	Dncic2	-1.08	-1.04	-1.14	-1.07	1.14	1.18	1.16
ILMN_1354402	Rpn1	-1.06	1.02	1.29	1.07	1.24	1.19	1.16
ILMN_1352621	LOC303666	1.09	-1.03	-1.03	1.04	1.25	1.2	1.16
ILMN_1360649	Ythdf2_predicted	-1	1.37	1.15	1.24	1.32	1.2	1.16
ILMN_1367563	LOC309197	1.26	1.1	1.13	1.16	1.05	1.21	1.16
ILMN_1349533	Gmfb	1.17	1.3	1.36	1.27	1.06	1.21	1.16
ILMN_1368636	Asns	1.03	1.64	1.36	1.31	1.23	1.23	1.16
ILMN_1368002	Snag1_predicted	1.29	1.02	1.22	1.35	1.15	1.24	1.16
ILMN_1360999	Twistnb_predicted	1.29	1.49	1.13	1.61	1.29	1.24	1.16
ILMN_1365950	Ihpk1	1.03	1.15	-1.13	1.02	1.21	1.25	1.16
ILMN_1362802	Rg9mtd3	1.05	1.07	-1.01	1.28	1.32	1.25	1.16
ILMN_1374132	RGD1305020_predicted	1.13	1.44	1.26	1.3	1.33	1.25	1.16
ILMN_1370968	RGD1305486	-1.14	1.18	1.22	1.59	1.21	1.27	1.16
ILMN_1356493	RGD1311072	-1.14	-1.01	-1.26	-1.27	1.45	1.27	1.16
ILMN_1376899	Frk	-1.02	-1.03	1.12	-1.23	1.39	1.28	1.16
ILMN_1374969	Lyl1	-1.09	1.14	1.09	1.1	1.24	1.32	1.16
ILMN_1353264	Nedd4a	-1.1	1.15	1.05	1.28	1.36	1.32	1.16
ILMN_1650513	LOC502383	1.22	1.06	1.11	1.05	1.24	1.36	1.16
ILMN_1361181	Ngrn	1.18	1.13	1.26	1.24	1.37	1.37	1.16
ILMN_1373694	LOC362315	1.05	1.09	1.48	1.23	1.23	1.38	1.16
ILMN_1367096	Actr6_predicted	-1.02	1.05	1.21	1.13	1.3	1.39	1.16
ILMN_1370553	Wdr77	1.22	1.37	2.14	1.68	1.31	1.47	1.16
ILMN_1364581	RGD1563633_predicted	1.08	-1.02	1.18	1.13	1.42	1.51	1.16
ILMN_1355779	LOC497691	-1.02	1.18	1.03	1.04	1.26	1.52	1.16
ILMN_1651193	LOC498145	1.09	1.25	-1.05	1.19	1.46	1.54	1.16
ILMN_1376694	Crem	1.22	-1.01	1.23	-1.11	1.55	1.54	1.16
ILMN_1375583	Crem	1.22	-1.01	1.23	-1.11	1.55	1.54	1.16
ILMN_1375833	Crem	1.22	-1.01	1.23	-1.11	1.55	1.54	1.16
ILMN_1372951	Fblim1	1.18	2.17	2.15	2.11	2.04	1.55	1.16

ILMN_1364779	Zc3h7a_predicted	-1.05	1.15	1.04	-1.03	1.53	1.57	1.16
ILMN_1357967	RGD1310323	-1.01	1.18	-1.15	1.13	1.23	1.6	1.16
ILMN_1371772	PVR	1.03	1.44	1.75	2.02	1.86	1.61	1.16
ILMN_1649825	LOC361269	1.14	1.63	1.46	1.71	2.04	1.74	1.16
ILMN_1351488	Pnrc1	-1.21	-1.18	-1.51	-2.24	1.47	1.84	1.16
ILMN_1365557	Cyb5r3	-1.01	-1.38	-1.32	-1.64	-1.3	-1.36	1.17
ILMN_1368440	RGD1310304_predicted	-1.08	-1.23	-1.33	-1.32	1.2	-1.13	1.17
ILMN_1358743	LOC367874	1.25	1.03	-1.09	1.13	-1.06	-1.11	1.17
ILMN_1366773	Sord	1.23	1.11	1.22	-1.07	1.03	-1.09	1.17
ILMN_1375097	Rabepk	-1.09	1	-1	-1.06	1.05	-1.06	1.17
ILMN_1367711	RGD1563072_predicted	-1.08	-1.03	1.04	-1.01	-1.14	-1.04	1.17
ILMN_1360344	Snf8	1.06	-1.34	-1.03	-1.31	-1.1	-1.04	1.17
ILMN_1362745	Snx5_predicted	-1.12	-1.14	1.16	-1.1	-1.06	-1.04	1.17
ILMN_1371606	Pomc	1.01	-1.07	-1.21	-1.2	1.1	-1.04	1.17
ILMN_1365588	Plekhg5	-1.02	1.39	1.3	1.75	-1.08	-1.03	1.17
ILMN_1355261	Nol5a	1.31	1.48	2.32	2.06	1.08	-1.03	1.17
ILMN_1349217	RGD1561189_predicted	1.28	-1	-1.67	-1.07	-1.14	-1.02	1.17
ILMN_1373886	Bxdc2	1.05	-1	1.12	-1.06	1.15	-1.02	1.17
ILMN_1371755	RGD1305833	-1	1.15	1.22	1.4	-1.16	-1	1.17
ILMN_1370601	RGD1310669	-1.23	-1.15	-1.2	-1.17	-1.01	1.01	1.17
ILMN_1650490	Bet1l	-1.07	-1.16	-1.27	-1.11	1.05	1.01	1.17
ILMN_1530310	siat7D	1.27	1.3	1.27	1.56	1	1.03	1.17
ILMN_1372351	Chrnb1	-1.11	-1.08	-1.11	-1.08	1.05	1.04	1.17
ILMN_1362108	RGD1562705_predicted	1.06	1.31	1.03	1.28	1.08	1.05	1.17
ILMN_1368272	Cct7_predicted	1.06	1.11	1.47	1.23	-1.01	1.06	1.17
ILMN_1363612	Hsbp1	-1.01	-1.3	-1.06	1.03	1.04	1.06	1.17
ILMN_1365910	Etfa	-1.22	1.05	1.14	-1.1	1.26	1.06	1.17
ILMN_1371791	Dapk1_predicted	-1.04	1.22	-1.2	1.29	1.07	1.08	1.17
ILMN_1375111	Eef2	-1.06	1.22	1.27	1.22	1.13	1.08	1.17
ILMN_1367831	RGD1564029_predicted	-1.09	1.14	1.14	1.21	1.13	1.09	1.17
ILMN_1362095	LOC684302	-1.03	-1.05	1.28	-1.06	1.1	1.1	1.17
ILMN_1353861	Ralbp1	1.06	-1.15	-1.15	-1.34	1.22	1.1	1.17
ILMN_1361787	Ppp1r14b	1.1	-1.03	-1.03	1.02	1.05	1.11	1.17
ILMN_1354572	LOC500663	-1.05	1.3	1.01	1.06	1.07	1.11	1.17
ILMN_1349128	MGC95208	1.16	1.1	1.15	1.02	1.08	1.11	1.17
ILMN_1370244	Zhx1	1.03	1.2	-1.09	1.07	1.13	1.11	1.17
ILMN_1374612	Tm9sf4_predicted	-1.07	1.16	1.07	-1.08	1.16	1.11	1.17
ILMN_1650581	Msra	1.08	1.13	1.13	1.26	1.1	1.12	1.17

ILMN_1368598	RGD1564893_predicted	1.02	1.12	1.16	1.2	1.33	1.12	1.17
ILMN_1354480	LOC501052	1.06	1.05	-1.06	1.03	-1.01	1.13	1.17
ILMN_1351359	Drg1	-1.04	1.23	1.34	1.37	1.03	1.13	1.17
ILMN_1363499	Rab13	1.1	1.37	1.33	1.2	1.34	1.13	1.17
ILMN_1650868	LOC499597	1.13	1.1	-1.12	-1.15	1.23	1.14	1.17
ILMN_1650594	Rps7	1.02	1.12	1.16	1.31	1.04	1.15	1.17
ILMN_1357225	Paip2_predicted	-1.1	-1.18	-1.12	-1.27	1.08	1.15	1.17
ILMN_1351639	Ddx18	1.05	1.64	1.34	1.87	1.22	1.15	1.17
ILMN_1649813	RGD1562974_predicted	-1.25	1.31	1.12	-1.02	1.16	1.16	1.17
ILMN_1373217	Adpgk	1.23	-1.1	1.02	1.04	1.18	1.16	1.17
ILMN_1370045	RGD1306925	-1.1	1.03	1.15	1.01	1.24	1.16	1.17
ILMN_1369176	Btbd9	1.08	1.31	1.16	1.12	1.38	1.16	1.17
ILMN_1358749	Nkiras2_predicted	1.04	1.13	1.1	1.21	1.23	1.17	1.17
ILMN_1360732	LOC365059	-1.01	1.29	1.19	1.18	1.17	1.19	1.17
ILMN_1357129	Lnp_predicted	1.07	1.26	1.28	1.45	1.28	1.19	1.17
ILMN_1369351	Tekt1	-1.16	1.12	1.11	-1.09	1.29	1.19	1.17
ILMN_1359707	LOC684829	1.1	1.21	1.04	1.26	1.2	1.2	1.17
ILMN_1650835	Psmd1	1.23	1.12	-1.11	1.31	1.23	1.21	1.17
ILMN_1370341	LOC497698	1.03	-1.14	-1.27	-1.04	1.18	1.22	1.17
ILMN_1650156	Tec	1.09	1.25	1.15	1.58	1.32	1.22	1.17
ILMN_1351104	Mad2l1bp	-1.04	1.2	-1.03	-1.09	1.4	1.22	1.17
ILMN_1376462	Fxr1h	1.14	1.25	1.19	1.23	1.17	1.23	1.17
ILMN_1358080	Por	-1.04	1.01	1.01	-1.08	1.23	1.23	1.17
ILMN_1369878	LOC686415	1.08	1.2	-1.01	1.2	1.2	1.24	1.17
ILMN_1369506	RGD1560367_predicted	1.08	1.33	1.04	1.23	1.2	1.24	1.17
ILMN_1371307	Alg12_predicted	-1	1.1	1.65	1.1	1.24	1.26	1.17
ILMN_1366338	Sdfr2_predicted	1.18	1.03	1.17	-1.05	1.27	1.26	1.17
ILMN_1358227	Taf7_predicted	1.09	1.2	1.22	1.27	1.16	1.27	1.17
ILMN_1362339	RGD1304816_predicted	-1	1.19	1.09	1.06	1.24	1.28	1.17
ILMN_1355423	Prpf39_predicted	-1.15	1.49	1.17	1.23	1.25	1.28	1.17
ILMN_1363266	Rpl13a	1.1	1.36	1.3	1.35	1.25	1.29	1.17
ILMN_1372533	Ranbp6_predicted	1.12	1.23	1.12	1.23	1.22	1.31	1.17
ILMN_1370116	LOC501594	1.12	1.05	-1.11	-1.06	1.26	1.31	1.17
ILMN_2039517	Impdh1_predicted	1.19	1.37	1.54	1.51	1.39	1.31	1.17
ILMN_1365012	Impdh1_predicted	1.19	1.37	1.54	1.51	1.39	1.31	1.17
ILMN_1370628	Golga5	1.1	1.54	1.39	1.03	1.24	1.32	1.17
ILMN_1362693	Bicd2	1.01	1.46	1.27	1.52	1.35	1.32	1.17
ILMN_1356903	Got2	1.04	1.08	1.26	1.14	1.32	1.35	1.17

ILMN_1367947	Slc1a2	-1.11	1.3	1.02	1.02	1.1	1.37	1.17
ILMN_1350875	LOC365214	1.04	1.17	1.48	1.2	1.28	1.38	1.17
ILMN_1351617	RGD1308260_predicted	1.04	1.36	1.35	1.16	1.55	1.39	1.17
ILMN_1366946	LOC299262	-1.02	-1.05	-1.13	1.13	1.23	1.4	1.17
ILMN_1355095	Ddx17	1.03	1.4	-1.4	1.49	1.45	1.43	1.17
ILMN_1367580	RGD1564026_predicted	-1.05	-1.07	1.5	1.38	1.41	1.46	1.17
ILMN_1370131	RGD1564130_predicted	1.32	1.8	1.58	1.81	1.57	1.71	1.17
ILMN_1372413	Cul4b_predicted	-1.02	-1.02	1.12	1.03	1.89	1.86	1.17
ILMN_1350883	Mus81	-1.16	1.28	1.11	1.3	-1.09	-1.24	1.18
ILMN_1354174	Abca3	-1.04	1.03	-1.09	-1.1	-1.12	-1.15	1.18
ILMN_1351798	Znf500_predicted	-1.01	1.19	1.12	1.16	-1.01	-1.08	1.18
ILMN_1353761	RGD1309682	1.02	-1.04	1.04	-1.39	-1.11	-1.04	1.18
ILMN_1371961	LOC365814	-1.08	-1.22	1.31	-1.06	1.12	-1.02	1.18
ILMN_1372695	RGD1559604_predicted	1.1	-1.03	-1.08	-1.08	1.07	-1.01	1.18
ILMN_1357094	Ifi30	1.17	1.2	1.58	1.37	-1	1	1.18
ILMN_2039613	RGD1559590_predicted	-1.09	1.07	-1.08	1.16	1.01	1.01	1.18
ILMN_1359585	Fcmd_predicted	1.18	-1.08	1.27	-1.07	-1	1.03	1.18
ILMN_1355429	Opa1	1.05	-1.11	-1.02	1.2	-1.07	1.06	1.18
ILMN_1362821	Ankrd42_predicted	-1.19	1.04	-1.04	-1.24	1.03	1.06	1.18
ILMN_1361991	Thap3_predicted	1.27	1.42	1.08	1.55	1.21	1.06	1.18
ILMN_1363372	Ets2_mapped	1.19	1	1.08	1.26	1.15	1.07	1.18
ILMN_1349734	Ssx2ip	1.1	1.15	1.33	1.37	1.05	1.09	1.18
ILMN_1371349	RGD1305077_predicted	1	-1	1.01	1.01	1.16	1.09	1.18
ILMN_1355371	Jak3	-1.19	-1.03	-1.02	-1.04	-1.01	1.1	1.18
ILMN_1357682	RGD1560397_predicted	1.02	1.11	-1.04	1.1	1.15	1.1	1.18
ILMN_1373888	Hint2_predicted	-1.05	1.02	-1.19	-1.26	1.21	1.1	1.18
ILMN_1367453	Gadd45g	1.17	-1.03	1.16	2.1	1.23	1.1	1.18
ILMN_1357254	LOC364343	-1.07	1.06	1.16	1.15	1.2	1.11	1.18
ILMN_1357471	LOC499575	-1.02	1.02	-1.01	-1.2	-1.14	1.12	1.18
ILMN_1358410	Phkg2	-1.01	1.36	1.21	1.19	1.05	1.12	1.18
ILMN_1361325	Zic5_predicted	1.21	1.44	1.51	1.71	1.22	1.13	1.18
ILMN_1366295	Rock1	1.1	1.03	-1.22	1.01	1.47	1.13	1.18
ILMN_1370001	Rpl17	-1.02	1.12	1.19	1.21	1.17	1.14	1.18
ILMN_1360323	LOC367229	1.05	1.24	1.16	-1.06	1.29	1.14	1.18
ILMN_1371628	RGD1359127	-1.1	1.63	2	1.82	1.29	1.15	1.18
ILMN_1350818	Rpp40	1.03	1.17	1.03	1.02	1.01	1.16	1.18
ILMN_1352941	Dusp12	-1.01	1.02	1.28	1.31	-1.06	1.17	1.18
ILMN_1359114	Jup	-1.04	1.06	-1.66	1.04	1.12	1.17	1.18

ILMN_1364199	Rnf121_predicted	1.12	1.13	1.52	1.16	1.18	1.17	1.18
ILMN_1354892	NTF2	-1.03	1.01	1.15	1.03	1.25	1.17	1.18
ILMN_1373455	RGD1563123_predicted	-1.13	-1.11	1.02	1.05	-1.01	1.19	1.18
ILMN_1359061	Havcr2	-1.09	1.1	1.03	1.08	1.18	1.19	1.18
ILMN_1372583	Pabpc4_predicted	1.02	1.34	1.35	1.33	1.18	1.2	1.18
ILMN_1356224	Syngr1	1.07	1.08	1.17	-1.01	-1.08	1.24	1.18
ILMN_1376795	Rnf103	-1.05	1.07	1.08	1.02	1.45	1.24	1.18
ILMN_1370886	Sec31l1	-1.05	1.12	1.2	1.24	1.21	1.25	1.18
ILMN_1363542	Tpd52_predicted	1.04	1.03	1.51	-1.06	1.12	1.3	1.18
ILMN_1365634	Serinc3	-1.07	-1.19	-1.31	-1.59	1.16	1.3	1.18
ILMN_1376678	Mllt3	1.14	1.48	1.46	1.53	1.42	1.3	1.18
ILMN_1359059	RGD1564993_predicted	1.06	1.24	1.27	1.17	1.45	1.3	1.18
ILMN_1368485	RGD1566025_predicted	1.03	1.39	1.09	1.34	1.27	1.31	1.18
ILMN_1370589	Dld	1	1.14	1.38	1.21	1.35	1.32	1.18
ILMN_1368492	RGD1562639_predicted	1.15	1.4	1.35	1.52	1.22	1.33	1.18
ILMN_1365756	Dnm1l	1.18	1.37	1.62	1.53	1.18	1.36	1.18
ILMN_1355312	Gapvd1_predicted	1.03	1.04	-1.03	1.07	1.3	1.37	1.18
ILMN_1350112	Siah1a	-1.02	1.44	1.34	1.3	1.45	1.4	1.18
ILMN_1368146	Rg9mtd1	1.15	1.38	1.33	1.38	1.43	1.43	1.18
ILMN_1376332	Dirc2	1.06	-1.38	-1.04	-1.47	1.35	1.44	1.18
ILMN_1364990	RGD1563950_predicted	1.14	1.41	1.54	1.48	1.23	1.55	1.18
ILMN_1367225	Vhl	1.08	1.46	1.69	1.52	1.56	1.67	1.18
ILMN_1376383	Atf2	1.08	1.27	1.04	1.17	1.72	1.72	1.18
ILMN_1372551	Ihpk2	-1.14	1.26	1.36	1.08	1.78	1.76	1.18
ILMN_1363209	Efemp2	-1.01	1.09	1.15	-1.01	-1.15	-1.28	1.19
ILMN_1350519	RGD1561796_predicted	-1.07	-1.42	-1.32	-1.29	-1.09	-1.26	1.19
ILMN_1357178	LOC292780	-1.21	1.02	-1.04	-1.02	-1.1	-1.24	1.19
ILMN_1351753	RGD1311186_predicted	1.02	1.15	1.01	1.14	-1.01	-1.17	1.19
ILMN_1369857	Vps28_predicted	-1.05	-1.06	-1.13	-1.22	-1.02	-1.07	1.19
ILMN_1363466	RGD1560566_predicted	1.03	1.04	-1.04	-1.14	1.05	-1.05	1.19
ILMN_1372530	Crkrs	-1.04	1.16	1.1	1.19	1.06	-1.05	1.19
ILMN_1357503	Ciz1_predicted	-1.08	1.14	1.07	1.11	1.03	-1.02	1.19
ILMN_1356563	Ptges2_predicted	-1.04	1.08	1.34	1.48	-1	-1	1.19
ILMN_1362000	Lyk5	1.03	-1.22	-1.01	1.06	-1.13	1.01	1.19
ILMN_1361598	Hddc2_predicted	1.07	1.24	1.12	1.32	1.08	1.01	1.19
ILMN_1372657	Pgm1	-1.08	-1.26	1.28	-1.17	-1.07	1.02	1.19
ILMN_1369945	RGD1563464_predicted	1.01	-1.14	-1.04	-1.02	1.13	1.02	1.19
ILMN_1360299	Thyn1	1.08	-1.13	-1.22	-1.08	-1.02	1.03	1.19

ILMN_1374227	Drb1	-1.01	1.22	1.42	1.26	-1.1	1.05	1.19
ILMN_1369607	RGD1359508	-1.06	-1.04	-1.12	-1.11	1.04	1.05	1.19
ILMN_1362969	RGD1309441_predicted	1.07	1.34	-1.17	1.27	1.14	1.05	1.19
ILMN_1362620	Pdp2	-1.03	-1.17	-1.2	-1.16	-1.01	1.06	1.19
ILMN_1373849	RGD1308901_predicted	1.04	1.23	-1	1.01	-1.08	1.07	1.19
ILMN_1362225	LOC497934	1.04	1.22	1.11	1.08	1.21	1.07	1.19
ILMN_1650149	Polr2j_predicted	1.01	-1.02	-1.31	-1.01	1.06	1.08	1.19
ILMN_1368127	LOC289378	-1.09	1.13	1.04	-1.09	1.07	1.08	1.19
ILMN_1356522	LOC364514	1.08	1.28	1.24	1.12	1.19	1.08	1.19
ILMN_1352932	Ddx10_predicted	1.21	1.32	1.15	1.37	1.16	1.09	1.19
ILMN_1353070	Man1a_predicted	1.32	1	1.04	1.12	1.26	1.09	1.19
ILMN_1530273	Cd99	1.02	-1.34	-1.49	-1.2	1.06	1.11	1.19
ILMN_1354773	Cul2_predicted	1.01	1.09	1.35	1.09	1.38	1.11	1.19
ILMN_1367683	LOC304500	1.03	-1.14	-1.19	-1.32	-1.03	1.12	1.19
ILMN_1358721	Pacs1	1.08	1.49	1.38	1.47	1.24	1.12	1.19
ILMN_1367825	Arpc2_predicted	-1.01	1.1	1.3	1.19	1.24	1.13	1.19
ILMN_1349752	Sdccag10	1.1	1.32	1.33	1.41	-1	1.14	1.19
ILMN_1360488	Ndufa6_predicted	1.08	1.23	-1.32	1.06	1.22	1.15	1.19
ILMN_1372728	Smad1	-1	1.03	-1.15	-1.04	1.35	1.16	1.19
ILMN_1356873	RGD1305647_predicted	1.39	1.21	1.62	1.04	1.16	1.17	1.19
ILMN_1372240	Hps1	1.02	1.35	1.52	1.1	1.17	1.17	1.19
ILMN_1352082	Nudt3	1.24	1.13	1.12	1.16	1.11	1.18	1.19
ILMN_1365591	RGD1559546_predicted	1.03	1.04	-1.11	-1.06	-1.11	1.19	1.19
ILMN_1372726	Nub1	-1.18	-1.18	-1.09	-1.24	1.16	1.19	1.19
ILMN_1350344	Ywhab	-1.05	-1.1	-1.19	1.05	1.23	1.2	1.19
ILMN_1354673	LOC683767	1.05	-1.12	1.27	-1.16	1.62	1.2	1.19
ILMN_1357410	LOC501559	1.11	1.26	1.16	1.15	1.22	1.21	1.19
ILMN_1362302	Gata4	1.18	1.42	1.15	1.13	1.29	1.21	1.19
ILMN_1358413	Spire2_predicted	-1.04	1.11	1.13	1.28	1.07	1.24	1.19
ILMN_1374516	RGD1565431_predicted	1	1.14	-1.2	1.07	1.12	1.24	1.19
ILMN_1369116	RGD1565372_predicted	-1.17	1.09	1.38	1.47	1.17	1.24	1.19
ILMN_1375096	Tmem123	-1.04	1.01	1.2	1.12	1.32	1.24	1.19
ILMN_1365903	Ebag9	-1.14	1.1	1.01	-1.02	1.21	1.25	1.19
ILMN_1371889	LOC689581	-1.13	1.21	1.35	1.35	1.25	1.25	1.19
ILMN_1355469	LOC362154	1.02	1.32	1.26	1.35	1.28	1.25	1.19
ILMN_2040330	Tmed9	1.09	1.18	1.14	1.24	1.32	1.25	1.19
ILMN_1359730	Rnf20_predicted	-1.02	-1.11	-1.35	1.17	-1	1.26	1.19
ILMN_1371064	Rps18	1.09	1.12	-1.06	1.11	1.15	1.26	1.19

ILMN_1354494	RGD1565661_predicted	-1.06	1.07	1.2	1.23	1.09	1.27	1.19
ILMN_1353588	Cnot7_predicted	-1.26	-1.22	-1.02	-1.11	1.14	1.3	1.19
ILMN_1357884	RGD1309308_predicted	-1.09	-1.04	-1.01	1.01	1.48	1.31	1.19
ILMN_1376971	Lpin1	1.02	1.22	-1	1.04	1.43	1.32	1.19
ILMN_1366923	Acot7	1.17	1.19	1.46	1.8	1.21	1.34	1.19
ILMN_1354443	Rnf111_predicted	-1.11	1.58	1.19	1.38	1.46	1.35	1.19
ILMN_1353342	RGD1307799	-1.2	1	-1.03	-1.05	1.22	1.37	1.19
ILMN_1362107	Sfrs15	1.05	1.35	1.24	1.43	1.43	1.38	1.19
ILMN_1349847	Ube2q_predicted	-1.17	1.02	1.44	1.16	1.33	1.39	1.19
ILMN_1376543	Nap1l1	1.1	1.22	1.59	1.56	1.27	1.4	1.19
ILMN_1351226	Cblb	-1.04	1.26	-1.1	1.09	1.71	1.4	1.19
ILMN_1349218	Casp3	-1.16	-1.07	-1.01	-1.11	1.29	1.42	1.19
ILMN_1362879	Ctdp1_predicted	-1.06	1.01	-1.09	1.05	1.34	1.43	1.19
ILMN_1351550	LOC498824	1.07	1.28	1.3	1.46	1.4	1.43	1.19
ILMN_1352468	Canx	1.05	-1.07	1.95	1.29	1.51	1.48	1.19
ILMN_1358855	RGD1306582	1.12	1.21	1.83	1.7	1.49	1.62	1.19
ILMN_1366139	Crcp	1.16	1.42	1.5	-1.03	1.82	1.68	1.19
ILMN_1354100	Arl1	-1.16	-1.09	-1.05	-1.06	1.81	1.76	1.19
ILMN_1354774	Ebpl_predicted	1.11	-1.01	-1.04	-1.02	-1.04	-1.23	1.2
ILMN_1357672	Rngtt_predicted	-1.03	1.08	1.11	1.22	-1.03	-1.21	1.2
ILMN_1372638	Cwf19l2_predicted	1.15	-1.11	-1.13	-1.01	1.14	-1.21	1.2
ILMN_1362106	LOC679534	1.02	1.57	1.19	1.08	-1.05	-1.19	1.2
ILMN_1368376	RGD1308134_predicted	-1.04	-1.17	-1.43	-1.19	-1.02	-1.19	1.2
ILMN_1358081	Ldhd	-1.02	1.02	1.02	-1.11	1.09	-1.19	1.2
ILMN_1367263	Sod2	-1.04	-1.03	1.08	1.03	-1.12	-1.18	1.2
ILMN_1369371	Pctp	-1.11	-1.44	-1.14	-1.5	-1	-1.18	1.2
ILMN_1368184	Nmi	-1.06	-1.16	1.02	-1.27	-1.19	-1.17	1.2
ILMN_1362278	Slc5a3	1.09	-1.01	-1.19	1.57	-1.22	-1.12	1.2
ILMN_1376752	Nudt4	1.05	-1	1.08	1.07	-1.09	-1.11	1.2
ILMN_1369261	RGD1311565	-1.01	-1.56	-1.07	-1.01	-1.24	-1.1	1.2
ILMN_1356367	LOC313391	1.21	1.08	1.08	-1.02	1.04	-1.09	1.2
ILMN_1353251	Tep1	-1.18	1.15	1.02	-1.04	-1.13	-1.07	1.2
ILMN_1376865	Hdac1	-1.05	-1.08	1.02	1.01	1.04	-1.06	1.2
ILMN_1374611	RGD1306595	1.11	1.09	1.09	1.05	-1.22	-1.05	1.2
ILMN_1349485	LOC503197	-1.07	1.05	1.12	-1.12	-1.03	-1.05	1.2
ILMN_1366191	P2rx4	-1.02	-1.09	-1.13	-1.17	1.17	-1.01	1.2
ILMN_1354804	RGD1359592	-1.16	-1.29	-1.24	-1.36	-1.02	-1	1.2
ILMN_1366519	RGD1310686	1.05	1.42	1.3	1.28	1.06	1.01	1.2

ILMN_1351077	Ltbr	1.03	1.09	1.3	1.19	-1.1	1.02	1.2
ILMN_1376256	Fundc1	-1.01	-1.19	-1.12	-1.26	-1	1.02	1.2
ILMN_1375256	Fundc1	-1.01	-1.19	-1.12	-1.26	-1	1.02	1.2
ILMN_1374013	LOC500733	-1.13	1.01	1.01	-1.03	-1.11	1.03	1.2
ILMN_1362122	RGD1309144	-1.05	-1.17	1.39	-1.68	1.01	1.04	1.2
ILMN_1372581	Pfdn5_predicted	1.09	1.14	-1.25	1.01	1.02	1.04	1.2
ILMN_1370369	Egr2	-1.09	1	1.08	-1.08	1.06	1.04	1.2
ILMN_1351346	Acads	1.03	1.29	-1	1.12	1.39	1.05	1.2
ILMN_1371232	Btbd14a	-1.03	1.08	-1.1	1.18	1.01	1.06	1.2
ILMN_1357439	RGD1560834_predicted	-1.02	1.28	1.1	1.34	1.17	1.07	1.2
ILMN_1363721	Timm8b	1.1	1.01	-1.43	1.08	1.09	1.09	1.2
ILMN_1351495	LOC686213	1.08	-1.1	1.29	-1.01	1.14	1.09	1.2
ILMN_1355065	Ptpn1	-1.03	1.04	1.21	1.06	-1.06	1.1	1.2
ILMN_1357507	Atg4b	1.09	1.06	1.13	1.1	1.14	1.1	1.2
ILMN_1369570	Centg3_predicted	-1.07	1.13	1.54	1.42	1.2	1.1	1.2
ILMN_1359257	Ap3m1	1.04	-1.15	-1.07	1.21	1.21	1.1	1.2
ILMN_1357293	RGD1561875_predicted	-1.03	1.08	1.08	1.14	1.03	1.12	1.2
ILMN_1359785	LOC500488	1.07	1.08	1.19	1.09	1.08	1.13	1.2
ILMN_1365463	Eno3	1.11	1.02	1.3	1.04	-1.08	1.14	1.2
ILMN_1363270	Hps6	-1.05	1.16	1.49	1.37	1.03	1.14	1.2
ILMN_1368377	LOC289809	1.17	1.17	1.38	1.52	1.11	1.14	1.2
ILMN_1374195	Pdcd5_predicted	1.08	1.08	-1.2	1.24	1.13	1.14	1.2
ILMN_1365009	Cbll1_predicted	-1.01	1.1	-1.25	1.01	1.14	1.14	1.2
ILMN_1351390	Psg4	-1.03	1.38	1.17	1.1	1.17	1.14	1.2
ILMN_1356323	Cecr5_predicted	1.09	1.04	-1.04	-1.08	1.18	1.14	1.2
ILMN_1355550	LOC498351	1.01	1.02	1.31	1.14	1.15	1.16	1.2
ILMN_1367208	RGD1310061	-1.33	1.27	1.67	1.8	1.03	1.17	1.2
ILMN_1366714	Pdcd11_predicted	1.05	1.58	1.73	1.55	1.07	1.17	1.2
ILMN_1367123	Catna1	-1.01	1.11	1.08	1.07	1.18	1.17	1.2
ILMN_1650188	Uchl3	-1.13	-1.13	1.06	1.24	1.04	1.18	1.2
ILMN_1367627	Vrk3	-1.19	1.02	1.08	-1.56	1.11	1.2	1.2
ILMN_1366781	Pla2g6	1.11	-1.24	-1.37	-1.36	1.12	1.2	1.2
ILMN_1366911	Asrgl1	1.11	1.2	1.23	1.11	1.28	1.21	1.2
ILMN_1359211	Lama5	-1.02	1.39	1.29	1.41	-1.22	1.22	1.2
ILMN_1376531	Tm7sf3	1.11	1.37	2.21	1.29	1.11	1.22	1.2
ILMN_1363515	Pum1_predicted	1.04	-1.07	1.06	1.1	1.12	1.22	1.2
ILMN_1355019	Pex12	-1.09	1.11	1.12	1.03	1.37	1.22	1.2
ILMN_1360006	RGD1306343_predicted	-1.02	1.04	1.1	-1.03	1.21	1.24	1.2

ILMN_1366442	Ube2j1_predicted	1.02	1.06	1.04	1.01	1.08	1.29	1.2
ILMN_1649889	Mtpn	1.01	-1.05	1.38	1.15	1.14	1.29	1.2
ILMN_1362020	LOC499221	1.1	1.34	1.06	1.29	1.19	1.29	1.2
ILMN_1356668	RGD1306116_predicted	-1.04	1.21	1.05	1.25	1.15	1.3	1.2
ILMN_1363794	LOC684012	1.1	1.55	1.56	1.55	1.23	1.3	1.2
ILMN_1365612	Lipogenin	1.06	1.11	1.36	1.46	1.31	1.3	1.2
ILMN_1366346	Emp3	1.21	1.18	1.11	1.87	1.31	1.35	1.2
ILMN_1369641	LOC302402	1.05	1.06	1.06	1.27	1.35	1.36	1.2
ILMN_1366154	Rpl39	-1.04	1.41	-1.08	1.38	1.48	1.4	1.2
ILMN_1376363	Cnksr3	1.23	1.3	1.06	1.14	1.53	1.41	1.2
ILMN_1356795	RGD1566181_predicted	-1.01	1.33	1.54	1.1	1.3	1.43	1.2
ILMN_1357208	Zfp36	1.05	1.38	1.07	-1.1	1.54	1.44	1.2
ILMN_1356266	Hmha1_predicted	1.1	1.21	-1	1.19	1.23	1.48	1.2
ILMN_1650382	RGD1560542_predicted	1.14	-1.05	1.12	1.23	1.5	1.51	1.2
ILMN_1369960	Magee1_predicted	1.14	1.1	1.06	1.18	1.74	1.71	1.2
ILMN_1371574	Gem_predicted	1.12	1.58	1.69	1.52	2.11	1.93	1.2
ILMN_1362647	Fahd2a_predicted	1	-1.29	-1.04	-1.13	-1.06	-1.44	1.21
ILMN_1650977	Eif3s7	1.27	1.33	1.35	1.27	-1.14	-1.23	1.21
ILMN_1371533	Maob	1.09	1.05	1.09	-1.22	1.01	-1.23	1.21
ILMN_1369181	RGD1310660	-1.13	-1.23	-1.32	-1.27	1.16	-1.16	1.21
ILMN_1360126	Josd2_predicted	1.15	-1.16	-1.08	-1.05	-1.01	-1.14	1.21
ILMN_2040134	Oact5	-1.08	-1.11	1.22	-1.12	1.06	-1.12	1.21
ILMN_1376336	Oact5	-1.08	-1.11	1.22	-1.12	1.06	-1.12	1.21
ILMN_1363632	Ndufa10	1	-1.21	-1	-1.22	1.05	-1.02	1.21
ILMN_1369764	Herc2_predicted	-1.18	-1.05	-1.16	-1.11	1.01	-1.01	1.21
ILMN_1359222	RGD1564821_predicted	1.11	1.05	1.28	1.05	1.11	-1.01	1.21
ILMN_1364573	Hdac4_predicted	1.39	1.27	1.11	1.29	1.05	-1	1.21
ILMN_1358151	Mrps23_predicted	-1.06	-1.01	-1.27	-1.4	1.13	1.01	1.21
ILMN_1369664	Kif1b	1.06	1.15	-1.04	1.08	1.19	1.01	1.21
ILMN_1372429	RGD1311340_predicted	1.14	1.41	1.21	1.25	-1.11	1.02	1.21
ILMN_1351989	Coro7	-1.24	1.24	1.36	1.25	-1.1	1.03	1.21
ILMN_1353611	RGD1305276	1.28	1.4	1.81	1.38	1.53	1.03	1.21
ILMN_1374928	RGD1309388_predicted	-1.11	1.01	1.14	1.15	-1.02	1.04	1.21
ILMN_1357390	Ptdss2_predicted	-1.03	1.13	1.52	-1.11	1.09	1.04	1.21
ILMN_1368342	RGD1309698_predicted	1.14	1.1	1.14	1.09	1.15	1.04	1.21
ILMN_1353471	RGD1306954	1.05	1.39	1.36	1.25	1.17	1.04	1.21
ILMN_1363069	Ptpn11	1.19	1.02	-1.3	1.08	-1.15	1.06	1.21
ILMN_1374375	Thop1	1.09	1.45	1.93	1.99	1.01	1.06	1.21

ILMN_1373292	Ywhah	-1.09	-1.29	-1.26	-1.21	1.09	1.06	1.21
ILMN_1352831	Bcor_predicted	1.03	1.17	1.2	1.2	1.08	1.07	1.21
ILMN_1362876	RGD1305138_predicted	-1.04	1.1	1.35	1.29	1.1	1.09	1.21
ILMN_1356829	Cuta	1.08	1.09	1.06	1.04	1.12	1.1	1.21
ILMN_1356460	Gramd1a	-1.06	1.61	1.4	1.41	1.17	1.1	1.21
ILMN_1374027	Ppt1	-1.18	-1.38	-1.26	-1.48	1.11	1.11	1.21
ILMN_1353438	Mrps9	1.15	1.32	1.38	1.21	1.15	1.11	1.21
ILMN_1367557	Ndufs1	1.05	1.1	1.18	1.04	1.18	1.11	1.21
ILMN_1370040	Sfxn4_predicted	-1.03	1.02	1.03	1.06	1.03	1.13	1.21
ILMN_1357063	RGD1561880_predicted	1.09	1.13	-1.43	-1.06	1.23	1.13	1.21
ILMN_1370380	Pdzk8_predicted	1.14	1.21	1.24	1.12	1.03	1.14	1.21
ILMN_1370112	Azgp1	1.09	-1.04	-1.13	1.1	1.04	1.17	1.21
ILMN_1368494	Psen2	1.23	1.35	1.22	1.3	1.13	1.17	1.21
ILMN_1360129	LOC691947	1	1.21	1.25	1.2	1.16	1.2	1.21
ILMN_1368112	Plcg1	1.04	1.39	1.46	1.46	1.11	1.22	1.21
ILMN_1352412	Ift74	-1.1	1.27	1.14	1.03	1.19	1.22	1.21
ILMN_1376661	Lbp	-1.07	-1.21	-1	-1.22	1.11	1.24	1.21
ILMN_1371209	Sectm1	1.03	1.3	1.27	-1.08	1.17	1.24	1.21
ILMN_1359410	LOC362317	-1.12	1.27	1.15	1.25	1.28	1.24	1.21
ILMN_1358540	Bat3	-1.06	1.1	1.17	1.16	1.1	1.25	1.21
ILMN_1362537	LOC499369	-1.03	1.37	1.41	1.36	1.51	1.27	1.21
ILMN_1365164	Parp16	1.21	1.18	1.08	1.12	1.42	1.29	1.21
ILMN_1374580	LOC498555	1.19	1.1	1.07	1.17	1.22	1.3	1.21
ILMN_1650651	Hccs_predicted	-1.1	1.13	1.24	1.45	1.42	1.3	1.21
ILMN_1351243	Riok1	1.07	1.44	1.16	1.67	1.47	1.33	1.21
ILMN_1364248	Nfe2l1_predicted	1.08	1.18	1.19	1.32	1.48	1.33	1.21
ILMN_1359572	LOC498829	1.07	-1.14	-1.26	-1.12	1.32	1.35	1.21
ILMN_1353549	Rras2	-1.11	-1.01	1.01	1.09	1.37	1.35	1.21
ILMN_1373265	Apg3l	1.12	1.43	1.21	1.23	1.48	1.38	1.21
ILMN_1364339	Cdc40_predicted	1.14	1.1	1.12	-1.03	1.38	1.39	1.21
ILMN_1370477	Cdc40_predicted	1.14	1.1	1.12	-1.03	1.38	1.39	1.21
ILMN_1361207	Mterfd1	1.07	1.23	1.44	1.36	1.41	1.39	1.21
ILMN_1650543	RGD1310552_predicted	1.01	-1.11	-1.2	-1.34	1.19	1.4	1.21
ILMN_1351833	Rps27	1.11	1.21	-1.27	1.18	1.3	1.4	1.21
ILMN_1364834	Zranb1_predicted	1.06	1.25	1.05	-1.05	1.37	1.41	1.21
ILMN_1356831	Ifngr2_predicted	1.04	1.19	1.58	1.44	1.37	1.43	1.21
ILMN_1651152	Tgif	-1	1.53	1.49	1.24	1.38	1.44	1.21
ILMN_1361586	Lrp16	-1	2.44	2.75	1.98	1.84	1.44	1.21

ILMN_1372652	Rbbp6	1.08	1.44	1.21	1.4	1.37	1.47	1.21
ILMN_2040487	Gtf2ird1	1.14	1.75	1.56	1.58	1.46	1.48	1.21
ILMN_1376536	Gtf2ird1	1.14	1.75	1.56	1.58	1.46	1.48	1.21
ILMN_1367125	RGD1309085_predicted	1.3	1.89	1.6	1.59	1.44	1.51	1.21
ILMN_1360658	RGD1560157_predicted	-1.17	1.47	1.77	1.69	1.58	1.52	1.21
ILMN_1352219	Nup98	-1.03	1.38	1.06	1.33	1.7	1.52	1.21
ILMN_1367849	Map4k4_predicted	-1.02	1.22	1.36	1.49	1.4	1.54	1.21
ILMN_1352966	Cd44	1.06	1.66	1.69	1.68	1.63	1.57	1.21
ILMN_1651181	Cap1	1.06	1.11	1.48	1.48	1.4	1.61	1.21
ILMN_1370445	LOC497802	1.18	1.24	1.68	1.14	1.57	1.66	1.21
ILMN_1349037	Max	1.22	1.11	-1.12	1.22	1.45	1.67	1.21
ILMN_1363078	MGC124825	-1.22	1.15	1.02	1.26	1.79	1.77	1.21
ILMN_1364638	Ixl_predicted	1.09	-1.06	-1.2	-1.08	-1.21	-1.38	1.22
ILMN_1374409	Ppp1r14a	1.03	1.34	-1.27	1.1	1.11	-1.34	1.22
ILMN_1357787	Fvt1_predicted	1.14	-1.62	-1.77	-1.67	-1.18	-1.14	1.22
ILMN_1359864	Yif1	-1.23	-1.15	1.16	-1.07	-1.02	-1.12	1.22
ILMN_1354048	Nupr1	1.22	1.28	-1.26	-1.06	1.12	-1.05	1.22
ILMN_1375131	MGC125015	1.09	1.29	-1.09	1.54	1.24	-1.05	1.22
ILMN_1367361	Itgb1bp1_predicted	-1.05	-1.06	-1.05	-1	1.03	-1.02	1.22
ILMN_1358379	LOC315496	1.17	1.08	1.27	1.07	1.08	-1.02	1.22
ILMN_1363570	RGD1561296_predicted	-1.06	1.54	1.15	1.34	1.16	-1	1.22
ILMN_1376891	Cndp2	-1.01	1.25	1.4	1.42	1.15	1.01	1.22
ILMN_1372591	RGD1309735_predicted	1.03	1.04	-1.24	-1.05	-1.13	1.03	1.22
ILMN_1356832	LOC315910	1.35	-1.02	1.24	-1.15	-1.07	1.03	1.22
ILMN_1356646	Mybbp1a	1.15	1.71	1.03	1.78	-1.02	1.04	1.22
ILMN_1368622	Lrrc41	-1.22	1.16	1.17	1.19	1.14	1.04	1.22
ILMN_1649927	Tmem106c	1.01	1.17	1.39	1.03	1.17	1.05	1.22
ILMN_1373807	RGD1309747_predicted	1.13	1.14	1.75	1.24	1.28	1.05	1.22
ILMN_1348987	RGD1308952	1.07	1.16	-1.21	1.2	-1.03	1.06	1.22
ILMN_2039286	Xylt2	1.01	1.72	1.59	1.8	1.25	1.09	1.22
ILMN_1651045	Map1lc3a	1.07	-1.14	-1.37	-1.16	1.36	1.1	1.22
ILMN_1356404	RGD1309655	-1.05	1.08	-1.14	-1.01	1.02	1.11	1.22
ILMN_1369617	RGD1310383_predicted	1	1.12	-1.16	1.04	1.04	1.11	1.22
ILMN_1353945	Srpk2_predicted	1.04	1.45	1.24	1.41	1.09	1.11	1.22
ILMN_1368747	RGD1305327	-1.07	1.03	1.01	-1.09	1.28	1.11	1.22
ILMN_1376862	Ppan	1.18	1.64	1.42	1.5	1.11	1.12	1.22
ILMN_1349387	RGD1305235	1.01	1.22	1.34	1.34	1.25	1.13	1.22
ILMN_1361983	Scnm1_predicted	-1.05	1.19	-1.07	-1.06	1.09	1.14	1.22

ILMN_1348931	Capn2	1.01	1.33	1.06	1.22	1.12	1.14	1.22
ILMN_1362329	RGD1560433_predicted	-1	1.34	1.18	1.3	1.16	1.15	1.22
ILMN_1350248	Ryk	1.08	1.07	1.35	1.12	1.22	1.15	1.22
ILMN_1371642	Pla2g4b_predicted	1.11	1.54	1.17	1.57	1.08	1.18	1.22
ILMN_1363880	LOC295340	-1	1.19	1.07	1.16	1.09	1.18	1.22
ILMN_1365391	Trim3	1.03	1.32	1.22	1.57	1.05	1.19	1.22
ILMN_1371046	LOC688103	1.01	1.58	1.07	1.25	1.32	1.19	1.22
ILMN_1360574	RGD1308321_predicted	1.04	1.16	1.08	1.25	1.33	1.19	1.22
ILMN_1374319	RGD1310066	1.36	1.2	1.02	1.25	1.27	1.21	1.22
ILMN_1371398	Rsrc1	1.11	1.14	1.06	-1.01	-1.1	1.23	1.22
ILMN_1376753	Rnf25	-1.12	1.37	1.12	1.2	1.32	1.23	1.22
ILMN_1368872	Xpot_predicted	1.44	1.42	1.41	1.44	1.21	1.24	1.22
ILMN_1351367	Rnf7_predicted	1.04	1.15	1.14	1.19	1.4	1.24	1.22
ILMN_1360724	Dnajd1_predicted	-1.14	1.04	-1.01	1.12	1.31	1.25	1.22
ILMN_1358832	RGD1564914_predicted	1.35	1.49	1.22	1.51	1.27	1.26	1.22
ILMN_1356375	Lars	1.16	1.27	-1.02	1.27	1.27	1.28	1.22
ILMN_1374462	Chchd3_predicted	1.06	1.13	1.4	1.12	1.24	1.3	1.22
ILMN_2040058	Chchd3_predicted	1.06	1.13	1.4	1.12	1.24	1.3	1.22
ILMN_1361101	Gtpbp2	1.1	1.35	1.24	1.26	1.29	1.3	1.22
ILMN_1369867	Phax	-1.1	1.19	1.2	1.3	1.29	1.3	1.22
ILMN_1364624	LOC501605	1.02	-1.09	1.3	1.24	1.16	1.32	1.22
ILMN_1650890	Vapa	1.12	1.25	1.51	1.4	1.36	1.34	1.22
ILMN_1363208	RGD1304758	-1.1	1.06	-1.01	1.05	1.3	1.36	1.22
ILMN_1365789	Pcmt1	1.07	-1.04	1.34	-1.04	1.33	1.44	1.22
ILMN_1356300	Nrbf2	-1.11	1.25	1.42	1.42	1.38	1.44	1.22
ILMN_1650491	Arl5b	1.08	1.39	1.26	1.35	1.4	1.45	1.22
ILMN_1376812	Ddah2	-1.14	1.22	1.5	1.43	1.74	1.45	1.22
ILMN_1355080	LOC363251	-1.13	-1.13	-1.05	1.05	1.53	1.52	1.22
ILMN_1354802	LOC686548	1.25	1.14	-1.14	1.17	1.49	1.53	1.22
ILMN_1363579	Nedd4l	1.06	1.05	1.01	-1.17	1.65	1.59	1.22
ILMN_1354506	Icam1	1.13	1.94	2.21	2.29	1.77	1.77	1.22
ILMN_1359139	Slc38a2	1.26	1.52	1.36	1.23	1.65	1.82	1.22
ILMN_1362394	Sdsl_predicted	-1.12	-1.07	-1.16	-1.21	-1.01	-1.15	1.23
ILMN_1352635	Ei24	-1.08	-1.67	-1.21	-1.38	-1.15	-1.08	1.23
ILMN_1372846	Bak1	-1.07	-1.15	-1.02	1.18	-1.12	-1.08	1.23
ILMN_1364747	Jub	1.45	1.68	1.16	1.83	-1.08	-1.07	1.23
ILMN_1368678	Rexo4	-1.01	1.01	-1.01	1.32	1.03	1	1.23
ILMN_1373746	Ctnnbl1	-1.02	-1.14	1.26	-1	1.09	1.02	1.23

ILMN_1362695	Snx13_predicted	1.01	1.12	1.31	1.1	1.14	1.04	1.23
ILMN_1372405	Cyp20a1	-1.01	1.06	1.49	1.18	1.11	1.07	1.23
ILMN_2040620	Tal1_predicted	1.11	1.11	1.23	1.1	1.14	1.08	1.23
ILMN_1356219	LOC502414	-1	-1.04	1.02	1.21	1.01	1.09	1.23
ILMN_1364522	Dnajc17_predicted	1.04	-1	1.06	1.06	1.17	1.1	1.23
ILMN_1352997	LOC501242	-1.02	1.01	1.13	1.32	1.35	1.1	1.23
ILMN_1358439	Gnptg	-1.08	-1.05	1.28	1.02	1.13	1.11	1.23
ILMN_1376277	LOC688338	1.09	1.13	1.17	1.02	1.04	1.12	1.23
ILMN_1530492	Dcun1d3	1.07	1.24	1.33	1.16	1.15	1.12	1.23
ILMN_1367962	LOC310395	-1.01	1.36	1.28	1.93	1.3	1.12	1.23
ILMN_1355092	Bloc1s2	1.05	1.13	1.1	1.1	1.26	1.15	1.23
ILMN_1350898	Pdap1	1.04	1.05	-1.13	1.17	1.11	1.16	1.23
ILMN_1364114	LOC289740	1.04	1.12	1.31	1.21	1.24	1.16	1.23
ILMN_1367059	Klhl22_predicted	1.18	1.33	1.33	1.63	1.24	1.16	1.23
ILMN_1349755	RGD1560263_predicted	-1.08	1.13	1.18	1.27	1.39	1.16	1.23
ILMN_1373122	Arpc5	-1.16	1	1.07	1.06	1.11	1.17	1.23
ILMN_1365769	Daam1_predicted	1.02	1.17	1.19	1.29	1.13	1.18	1.23
ILMN_1374864	B3gnt1_predicted	1.32	1.23	1.5	1.39	1.34	1.18	1.23
ILMN_1371210	Rraga	-1.14	1.07	1.24	1.13	1.14	1.19	1.23
ILMN_1372036	RGD1565956_predicted	-1.04	1.14	1.09	1.15	1.23	1.19	1.23
ILMN_1351741	RGD1305823	1.21	-1.09	-1.03	1.02	1.16	1.2	1.23
ILMN_1354982	Klhl5	1.11	1.15	1.26	1.26	1.04	1.21	1.23
ILMN_1352365	Narg1_predicted	1.06	1.15	1.04	1.27	1.27	1.21	1.23
ILMN_1364236	Atic	-1.05	1.11	1.2	1.37	1.08	1.22	1.23
ILMN_1349830	Vegfc	1.21	1.2	-1.04	1	1.14	1.22	1.23
ILMN_2039130	Rab10	1.14	1.28	1.31	1.48	1.2	1.22	1.23
ILMN_1360016	Tcf1	1.09	1.56	-1.01	1.25	1.21	1.23	1.23
ILMN_1376599	Panx2	1.03	1.4	1.28	1.26	1.46	1.24	1.23
ILMN_1349007	Aamp_predicted	-1.16	-1.27	1.1	-1.06	1.15	1.25	1.23
ILMN_1351591	Dctn3_predicted	-1.04	1.03	-1.27	-1.03	1.24	1.26	1.23
ILMN_1371702	Hmgcl	1.22	1.39	1.49	1.23	1.42	1.26	1.23
ILMN_1353558	Pdia3	1.01	-1.6	1.44	-1.13	1.46	1.31	1.23
ILMN_1350803	LOC498623	1.05	-1.02	-1.02	1.24	1.4	1.38	1.23
ILMN_1355238	Arfip1	1.15	1.14	1.52	1.15	1.46	1.4	1.23
ILMN_1362091	RGD1309414_predicted	-1.33	1.17	1.24	1.41	1.46	1.41	1.23
ILMN_1367016	Ubl3	-1.05	-1.02	-1.08	1.02	1.28	1.42	1.23
ILMN_1350124	Trim39	-1.1	1.22	1.22	1.27	1.29	1.44	1.23
ILMN_1353929	Tenc1_predicted	1.14	1.33	-1.06	1.3	1.43	1.46	1.23

ILMN_1373979	LOC367171	1.08	1.13	-1.12	1.27	1.44	1.46	1.23
ILMN_1350470	Ugt1a1	1.08	1.14	1.35	1.39	1.28	1.47	1.23
ILMN_1374665	LOC497836	1.14	1.73	1.6	1.43	1.52	1.47	1.23
ILMN_1367190	Hcfc2	-1.12	1.3	1.35	1.39	1.71	1.49	1.23
ILMN_1364180	RGD1564883_predicted	1	1.41	1.54	1.41	1.41	1.53	1.23
ILMN_1355191	Slc16a1	1.24	1.32	1.44	1.67	1.52	1.64	1.23
ILMN_1356828	Arg1	-1	-1.44	-1.79	-2.38	-1.36	-1.8	1.24
ILMN_1373630	Aqp11	-1.11	-1.1	-1.29	-1.28	-1.38	-1.65	1.24
ILMN_1374107	Acaca	1.02	-1.27	-1.01	-1.02	-1.11	-1.25	1.24
ILMN_1650267	RGD1307010	-1.04	-1.17	1.24	-1.03	1.08	-1.08	1.24
ILMN_1360087	Pde6d_predicted	1.05	-1.19	1.07	1.01	-1.05	-1.07	1.24
ILMN_1360519	Polg2_predicted	-1	1.24	1.3	1.23	-1	-1.04	1.24
ILMN_1361404	Adck2_predicted	-1.07	1.1	1.04	1.12	-1.12	-1.03	1.24
ILMN_1357120	Lrrc16_predicted	-1.05	1.24	1.05	1.48	1.08	-1.01	1.24
ILMN_1352067	Cited2	1.16	-1.77	-2.03	-1.41	-1.02	-1	1.24
ILMN_2040701	Becn1	-1.1	-1.12	1.06	-1.07	-1.01	1.01	1.24
ILMN_2040712	Mocs3_predicted	1.03	1.01	1.12	1.01	1	1.02	1.24
ILMN_1354528	Wdr3_predicted	1.18	1.25	1.46	1.29	1.06	1.02	1.24
ILMN_1355133	RGD1565319_predicted	1.28	1.18	1.29	1.8	-1.08	1.03	1.24
ILMN_1354162	Wdr34	1.12	1.61	1.88	1.21	-1.12	1.04	1.24
ILMN_1650241	Tmem60_predicted	1.17	1.07	1.1	1.34	1.08	1.04	1.24
ILMN_1360424	Lrrc47_predicted	1.02	1.17	1.22	1.17	1.06	1.07	1.24
ILMN_1360597	LOC296884	-1.16	-1.16	1.06	-1.14	1.08	1.07	1.24
ILMN_1368625	RGD1306772_predicted	1.11	1.1	1.34	1.15	1.14	1.09	1.24
ILMN_1350984	Mtch1	-1.07	-1.31	-1.41	-1.13	1.16	1.12	1.24
ILMN_1371852	RGD1307374_predicted	-1.04	1.36	1.08	1.07	1.18	1.12	1.24
ILMN_1367449	LOC363942	-1.03	1.16	1.22	1.33	1.04	1.13	1.24
ILMN_1371146	RGD1565297_predicted	1.29	-1.26	1.15	1.59	1.07	1.17	1.24
ILMN_1351150	Capzb	1.2	1.13	1.19	1.29	1.22	1.18	1.24
ILMN_1365755	P34	1.25	1.22	1.63	1.64	1.28	1.18	1.24
ILMN_1376252	Nnp1	1.23	1.23	1.6	1.36	1.18	1.19	1.24
ILMN_1372045	RGD1306538	-1.01	-1.24	1.12	1.05	1.21	1.19	1.24
ILMN_1373308	Ppat	1.17	1.4	1.24	1.5	1.23	1.19	1.24
ILMN_1356183	LOC683302	-1.09	1.02	-1.03	1.01	1.23	1.2	1.24
ILMN_1350789	LOC685068	1.02	1.07	1.14	1.15	1.25	1.2	1.24
ILMN_1366491	LOC308650	-1.08	-1.01	1.17	-1.05	1.13	1.21	1.24
ILMN_1352823	MGC124992	1.12	1.63	1.44	1.56	1.23	1.22	1.24
ILMN_1352608	Tax1bp3	-1.16	1.12	-1.02	1.16	1.28	1.23	1.24

ILMN_1370110	Rps24	1.08	1.09	1.09	1.12	1.12	1.28	1.24
ILMN_1366151	Pxmp3	-1.04	1.01	1.14	1.01	1.28	1.28	1.24
ILMN_1359804	Cab39_predicted	-1.04	1.33	1.43	1.38	1.52	1.29	1.24
ILMN_1365074	Tfam	1.02	1.18	1.04	1.17	1.09	1.31	1.24
ILMN_1651148	Polr2i_predicted	1.4	1.17	-1.34	1.26	1.45	1.31	1.24
ILMN_1349038	Gsto1	1.01	1.39	1.39	1.34	1.43	1.35	1.24
ILMN_1364378	LOC501633	1.15	1.21	1.21	1.38	1.5	1.36	1.24
ILMN_1365752	Prcp_predicted	1.02	1.41	1.87	1.32	1.22	1.4	1.24
ILMN_1359413	Slc35b1	-1.04	-1.14	1.3	1.02	1.56	1.4	1.24
ILMN_1365486	Prei3	-1.01	-1.04	1.23	-1.01	1.46	1.41	1.24
ILMN_1354727	RGD1562236_predicted	1.12	1.32	-1	1.23	1.34	1.46	1.24
ILMN_1358316	Casc1_predicted	1.06	1.13	1.05	-1.04	1.38	1.52	1.24
ILMN_1373587	Etf1	1.22	1.44	1.53	1.75	1.43	1.56	1.24
ILMN_1372263	Sar1a	-1.01	-1.03	1.26	1.28	1.75	1.58	1.24
ILMN_1367231	RGD1310474_predicted	1.15	1.53	1.38	1.67	1.61	1.59	1.24
ILMN_1352370	Cln3	1.19	1.56	1.32	1.42	1.43	1.61	1.24
ILMN_1373383	Tiparp_predicted	1.01	1.27	1.38	1.29	1.77	1.62	1.24
ILMN_1351668	Pcf11_predicted	1.08	1.49	1.49	1.38	1.89	1.97	1.24
ILMN_1359426	RGD1305592	1	-1.05	-1.15	1.28	1.09	-1.26	1.25
ILMN_1358509	Adam15	-1.07	-1.11	1.16	-1.02	-1.05	-1.16	1.25
ILMN_1371265	Tmprss6_predicted	-1.15	1.04	1.29	1.06	1.11	-1.16	1.25
ILMN_1353786	RGD1560049_predicted	-1.09	1.02	1.15	1.12	1.18	-1.07	1.25
ILMN_1371644	Adck5	1.14	1.56	1.44	1.56	-1.06	-1.05	1.25
ILMN_2040631	LOC501965	1.05	1.01	1.23	1.08	-1.02	-1.02	1.25
ILMN_1372047	RGD1563296_predicted	1.01	1.08	1.15	-1.01	-1.03	-1	1.25
ILMN_1360481	Plec1	1.26	1.07	1.01	1.54	-1.06	1.01	1.25
ILMN_1363103	Ms4a8b_predicted	1.06	-1.17	-1.42	1.05	1.18	1.02	1.25
ILMN_1361699	Nkiras1_predicted	1.18	-1.07	1.03	1.37	1.04	1.03	1.25
ILMN_1364018	RGD1562114_predicted	1.22	1.21	-1.03	1.13	-1.1	1.04	1.25
ILMN_1362094	RGD1565210_predicted	1.19	1.09	1.02	-1.1	-1.05	1.04	1.25
ILMN_1370470	RGD1565196_predicted	1.01	-1.03	1.07	1.17	1.06	1.04	1.25
ILMN_1359504	Tcea2	1.08	1.29	1.54	1.23	1.06	1.04	1.25
ILMN_1354159	LOC500420	1.06	1.16	1.18	1.04	1.12	1.05	1.25
ILMN_1360110	Afg3l2	1.27	1.21	1.07	1.1	1.02	1.09	1.25
ILMN_1649942	Camk1	-1.08	1.3	1.55	1.11	1.14	1.09	1.25
ILMN_1372010	Dnaja4	-1.04	-1.09	1.18	-1.01	1.06	1.1	1.25
ILMN_1364280	RGD1306614	-1.07	-1.06	-1.01	-1.05	1.09	1.1	1.25
ILMN_1650187	LOC501341	1.03	1	1	1.04	-1.01	1.11	1.25

ILMN_1367572	Wdr35_predicted	-1.1	-1.03	1.04	1.14	1.19	1.11	1.25
ILMN_1357283	Metap2	1.06	1.15	1.42	1.24	1.05	1.12	1.25
ILMN_1372663	Ercc1_predicted	1.03	1.14	1.2	1.01	1.12	1.12	1.25
ILMN_1364041	Mrpl55_predicted	1.22	1.29	-1	1.28	1.16	1.12	1.25
ILMN_1366837	Tbc1d19_predicted	1	1.12	1.16	1.2	1.24	1.15	1.25
ILMN_1369677	RGD1305158_predicted	1.18	-1.07	-1.2	-1.07	1.3	1.15	1.25
ILMN_1363089	LOC298012	-1.03	-1.07	1.22	1.08	1.3	1.15	1.25
ILMN_1353282	RGD1309529_predicted	1.1	1.31	1.16	1.24	1.35	1.15	1.25
ILMN_1354535	Znf386	-1.03	-1.06	1.02	-1.1	1.06	1.17	1.25
ILMN_1369336	Freq	-1.04	1.12	1.04	-1.06	1.1	1.17	1.25
ILMN_1649981	Strn3	-1.08	1.31	1.12	1.14	1.3	1.2	1.25
ILMN_1357326	Nme7	-1.09	-1.28	-1.02	-1.14	-1.15	1.21	1.25
ILMN_1374719	Pja2	-1.14	-1.07	-1.2	-1.13	1.48	1.21	1.25
ILMN_1354796	RGD1562370_predicted	-1.01	1.03	-1.12	-1.06	-1.02	1.22	1.25
ILMN_1348906	Myo1d	-1.06	1.18	-1.05	1.17	1.29	1.23	1.25
ILMN_1650482	Ndfip1	1.06	1.02	1.13	1.12	1.12	1.26	1.25
ILMN_1356617	Zfr	1.23	1.24	1.15	1.23	1.38	1.26	1.25
ILMN_1362932	RGD1565370_predicted	1.05	1.35	1.02	1.29	1.3	1.27	1.25
ILMN_2039179	RGD1562402_predicted	1.19	1.33	-1.06	1.31	1.22	1.29	1.25
ILMN_1367901	RGD1562402_predicted	1.19	1.33	-1.06	1.31	1.22	1.29	1.25
ILMN_1371871	Arhgap5	1.19	1.38	1.19	1.29	1.48	1.29	1.25
ILMN_1352371	Mrpl47	1.02	1.29	1.08	1.45	1.31	1.32	1.25
ILMN_1349541	Neo1	1.17	1.36	1.14	1.28	1.08	1.33	1.25
ILMN_1375064	Cd82	-1.12	1.08	1.38	1.14	1.33	1.33	1.25
ILMN_1362052	LOC498256	1.03	1.16	1.26	1.36	1.25	1.34	1.25
ILMN_1350841	RGD1563764_predicted	1.14	1.33	1.21	1.27	1.37	1.34	1.25
ILMN_1373714	RGD1305754_predicted	1.02	-1.03	-1.02	1.06	1.07	1.38	1.25
ILMN_1369550	RGD1305622	1.29	1.48	1.32	1.7	1.4	1.4	1.25
ILMN_1356699	St13	1.03	1.27	1.38	1.43	1.31	1.42	1.25
ILMN_1372717	Fem1c_predicted	-1.09	1.31	1.35	1.27	1.46	1.45	1.25
ILMN_1350340	Hrmt1l1	1.1	1.39	1.08	1.33	1.53	1.49	1.25
ILMN_1355507	Brd2	-1.03	1.48	1.01	1.6	1.37	1.5	1.25
ILMN_1352273	Kns2	1.21	1.35	1.47	1.69	1.45	1.51	1.25
ILMN_1373367	RGD1311066_predicted	-1.01	1.1	1.01	1.09	1.15	1.56	1.25
ILMN_1358373	Mak3_predicted	1.38	1.86	1.58	2.29	1.39	1.6	1.25
ILMN_1368549	Sts	1	1.58	1.16	1.16	1.64	1.87	1.25
ILMN_1353445	Cttnbp2nl_predicted	-1.04	1.54	1.18	1.22	2.17	2.04	1.25
ILMN_1366162	Oma1_predicted	1.01	-1.2	1.02	-1.1	-1.44	-1.24	1.26

ILMN_1364882	Ttc14_predicted	-1.03	1.32	1.1	1.18	1.02	-1.04	1.26
ILMN_1352769	Uxs1	-1.05	1.01	1.45	1.48	1.05	-1.04	1.26
ILMN_1366290	LOC500344	-1.08	-1	1.06	-1.01	-1.04	-1.03	1.26
ILMN_1370760	Wdr1	-1.06	1	1.02	1.23	1.01	1.01	1.26
ILMN_1369522	Sgta	-1	1.06	1.18	1.11	1.01	1.04	1.26
ILMN_1360610	RGD1308324_predicted	-1.1	1.25	-1.01	1.08	1.07	1.05	1.26
ILMN_1375028	Htatip2_predicted	1.36	-1.02	-1.08	1.14	1.06	1.06	1.26
ILMN_1351069	Apln	1.13	1.28	1.22	1.96	1.25	1.06	1.26
ILMN_1349172	RGD1311925_predicted	1.02	1.25	1.15	1.11	-1.03	1.07	1.26
ILMN_1367040	Actr10	1.06	1.18	1.25	1.16	1.18	1.08	1.26
ILMN_1372599	RGD1563595_predicted	1.04	1.11	1.27	1.28	1.2	1.08	1.26
ILMN_1360607	Numbl_predicted	1.06	1.2	-1	1.15	1.18	1.11	1.26
ILMN_1360039	Pafah1b3	1.05	1.33	1.31	1.2	1.01	1.13	1.26
ILMN_1363300	Mrpl15_predicted	-1.02	1.33	1.11	1.3	1.08	1.17	1.26
ILMN_1350827	Ndufa1_predicted	1.17	-1.12	-2.57	1.01	1.33	1.21	1.26
ILMN_1369781	Pdcl3	-1.17	-1.01	1.18	1.23	1.17	1.23	1.26
ILMN_1362863	RGD1307752	1.18	1.21	1.24	1.12	1.3	1.23	1.26
ILMN_1354030	Trio	1.06	1.39	1.19	1.59	1.38	1.27	1.26
ILMN_1370493	RGD1561853_predicted	1.27	1.49	2.23	1.7	1.04	1.28	1.26
ILMN_1360364	Arvcf_predicted	1.04	1.36	1.09	1.58	1.17	1.28	1.26
ILMN_1370883	Tmed5	1.15	1.04	-1.07	1.1	1.4	1.28	1.26
ILMN_1365408	Ptk9	-1.04	1.15	1.07	1.04	1.28	1.29	1.26
ILMN_1376286	Crlz1	-1.08	1.33	1.19	1.6	1.18	1.3	1.26
ILMN_1360450	Wtip_predicted	1.06	1.57	1.4	1.86	1.32	1.32	1.26
ILMN_1360940	RGD1564247_predicted	1.07	1.5	1.38	1.69	1.49	1.33	1.26
ILMN_1355600	RGD1564247_predicted	1.07	1.5	1.38	1.69	1.49	1.33	1.26
ILMN_1363792	Ide	1.17	1.01	1.32	1.19	1.46	1.35	1.26
ILMN_1354288	Kif5b	1.15	1.25	1.06	1.53	1.44	1.37	1.26
ILMN_1351884	Pmvk	1.25	1.23	1.26	1.16	1.72	1.39	1.26
ILMN_1352496	Sgpl1	1.03	1.32	1.68	1.64	1.07	1.41	1.26
ILMN_1364303	LOC299750	1.02	1.32	1.36	1.09	1.4	1.41	1.26
ILMN_1375194	LOC500867	1.05	1.21	1.35	1.4	1.52	1.41	1.26
ILMN_1353423	LOC500058	1	1.46	1.26	1.34	1.43	1.42	1.26
ILMN_1349648	Gsk3b	1.17	1.2	1.36	1.36	1.25	1.44	1.26
ILMN_1361128	Acat2	-1.02	-2.04	-1.41	-1.5	1.3	1.45	1.26
ILMN_1371063	LOC498048	1.17	1.18	1.55	1.23	1.34	1.47	1.26
ILMN_1357903	RGD1564171_predicted	1.02	1.11	1.16	1.13	1.7	1.47	1.26
ILMN_1355694	LOC363492	1.14	1.22	1.32	1.23	1.47	1.48	1.26

ILMN_1357227	Zfp216_predicted	-1.22	1.1	1.19	-1.2	1.51	1.48	1.26
ILMN_1371446	Rnf138	1.02	1.2	1.57	1.43	1.61	1.49	1.26
ILMN_1374685	Trim27_predicted	1.07	1.56	1.34	2	1.69	1.5	1.26
ILMN_1371291	Ppp2r2d	1	1.15	1.23	1.41	1.5	1.6	1.26
ILMN_1359104	Kdelr3_predicted	1.13	1.25	1.46	1.23	1.72	1.6	1.26
ILMN_1350980	Rad17	1.13	1.39	1.39	1.3	1.67	1.62	1.26
ILMN_1651132	Klhl24	1.32	1.14	-1.21	-1.48	1.25	1.66	1.26
ILMN_1368216	Arhgap12_predicted	-1.01	1.46	1.34	1.34	1.72	1.74	1.26
ILMN_1358501	Lmbrd1	-1.09	1.16	1.07	1.03	1.8	1.78	1.26
ILMN_1351079	Rpp14_predicted	1.37	1.39	1.42	1.46	1.67	1.81	1.26
ILMN_1360470	RGD1560812_predicted	1.18	2.01	1.89	1.84	1.86	1.94	1.26
ILMN_1357457	Zcsl3_predicted	1.05	-1.33	-1.39	-1.1	-1.03	-1.19	1.27
ILMN_1364083	LOC362681	1.13	1.33	1.26	1.2	1.06	-1.13	1.27
ILMN_1351254	Tcirg1	-1.05	1.42	1.02	1.21	-1.18	-1.11	1.27
ILMN_1376460	Aldh3a2	1.05	-1.29	-1.54	-1.79	-1.11	-1.07	1.27
ILMN_1358462	Rwdd3	1.22	1.01	-1.06	1.01	-1.08	-1.07	1.27
ILMN_1355027	Fyttd1	-1.06	1.03	1.16	1.12	1.05	-1.03	1.27
ILMN_1352930	Eml2	-1.02	1.23	1.14	1.23	-1.17	1.02	1.27
ILMN_1367358	Gnl1	1.18	1.03	-1.1	1.21	-1.06	1.02	1.27
ILMN_2039107	Rps6ka1	1.01	1.13	-1.02	1.24	1.11	1.02	1.27
ILMN_1351360	LOC498329	1.21	1.38	-1.66	1.29	1.17	1.02	1.27
ILMN_1374175	RGD1565768_predicted	-1.05	1.24	1.01	1.1	1.25	1.04	1.27
ILMN_1355516	Tceb1	-1.02	-1.07	-1.19	1.04	1.21	1.07	1.27
ILMN_1355733	Acadl	1.02	1.15	1.05	-1.04	1.16	1.08	1.27
ILMN_1359536	Dok1	1.13	1.23	-1.09	1.38	1.02	1.09	1.27
ILMN_1360648	RGD1359191	1.02	1.6	1.48	1.76	1.26	1.11	1.27
ILMN_1367097	Tnfrsf11a_predicted	1.15	1.14	1.24	1.22	1.12	1.14	1.27
ILMN_1359313	RGD1562173_predicted	1.15	1.26	2.03	1.87	1.17	1.17	1.27
ILMN_1372872	Ddx55_predicted	1.17	1.39	1.36	1.53	1.31	1.18	1.27
ILMN_1355275	Ilf3	1.06	1.32	1.05	1.19	1.15	1.19	1.27
ILMN_1374445	Hdac8_predicted	1.07	1.22	1.69	1.24	1.15	1.19	1.27
ILMN_1360564	Kifap3_predicted	1.14	1.13	1.16	1.17	1.14	1.2	1.27
ILMN_1352909	Mbtps2	1.11	1.56	1.39	1.46	1.06	1.25	1.27
ILMN_1359636	Snd1	-1.16	1.15	1.13	1.23	1.44	1.27	1.27
ILMN_1375215	Nbn	1.09	-1.35	-1.13	-1.02	1.19	1.28	1.27
ILMN_1355177	Dusp5	-1.05	1.22	1.28	1.29	1.39	1.28	1.27
ILMN_1351213	E2f5	1.2	1.68	1.84	2.15	1.19	1.29	1.27
ILMN_1364476	Fubp3_predicted	-1.05	1.12	1.13	1.24	1.28	1.29	1.27

ILMN_1351431	RGD1559938_predicted	1.02	1.07	1.42	1.49	1.07	1.3	1.27
ILMN_1354491	RGD1305866_predicted	-1.14	1.15	1.24	1.24	1.41	1.3	1.27
ILMN_1353460	Rhot1_predicted	1.01	1.4	1.42	1.59	1.36	1.32	1.27
ILMN_1349521	LOC289715	1.05	1.25	-1.11	1.22	1.35	1.33	1.27
ILMN_1373167	RGD1562702_predicted	-1.07	-1.11	1.06	-1.02	1.18	1.35	1.27
ILMN_1359551	LOC500898	-1.2	1.33	1.26	1.31	1.06	1.37	1.27
ILMN_1356718	Exoc2	-1.12	1.2	1.01	-1.03	1.11	1.37	1.27
ILMN_1366574	Lcmt1	1.01	1.07	1.22	1.03	1.22	1.4	1.27
ILMN_1372699	Spata5_predicted	1.06	1.35	1.37	1.54	1.73	1.41	1.27
ILMN_1376634	Xkr8	-1.02	1.33	1.26	1.47	1.35	1.44	1.27
ILMN_1354735	RGD1563956_predicted	1.14	1.37	1.52	1.52	1.45	1.45	1.27
ILMN_1372570	LOC501207	1.02	1.26	1.1	1.19	1.21	1.47	1.27
ILMN_1355775	Rragc_predicted	-1.01	1.39	1.64	1.4	1.4	1.5	1.27
ILMN_1349724	Ipo7_predicted	1.28	1.49	1.15	1.44	1.37	1.51	1.27
ILMN_1376445	Plekhc1	1.02	1.52	1.43	1.55	1.85	1.54	1.27
ILMN_1352401	Sirt2	1.08	1.37	1.2	1.24	1.46	1.56	1.27
ILMN_1373778	LOC361750	1.03	1.08	1.19	1.45	1.38	1.57	1.27
ILMN_1370105	Commd9	-1.05	1.1	1.08	1.01	1.57	1.62	1.27
ILMN_1650633	LOC499793	-1.05	1.39	1.72	1.98	1.76	1.98	1.27
ILMN_1371314	Comtd1_predicted	1.03	1.36	1.02	1.01	1.01	-1.19	1.28
ILMN_1371283	Vps18_predicted	1.03	-1.04	1.05	-1.05	1.06	-1.06	1.28
ILMN_1368262	Fahd1	-1.08	-1.05	-1.16	-1.18	1.03	-1.05	1.28
ILMN_1368076	Cog7	-1.05	-1.13	1.16	-1.25	-1.22	-1.01	1.28
ILMN_1372897	Xpo4_predicted	-1.01	1.18	-1.23	1.33	-1.03	1.01	1.28
ILMN_1651151	LOC499009	-1.12	1.26	1.11	1.1	-1.07	1.02	1.28
ILMN_1359493	RGD1564804_predicted	1.02	-1.11	-1.08	-1.09	1.33	1.02	1.28
ILMN_2040592	Cib2	-1	1.05	-1.05	1.03	1.09	1.03	1.28
ILMN_1363578	Zgpat	-1.19	1.24	1.01	-1.01	-1.05	1.04	1.28
ILMN_1351578	Pus1	1.05	1.39	1.22	1.94	1.07	1.04	1.28
ILMN_1353625	LOC292069	1.01	1.15	1.25	1.78	1.11	1.06	1.28
ILMN_1370039	Usp32_predicted	1	-1.09	1.06	-1.12	-1.05	1.09	1.28
ILMN_1374848	Hps3_predicted	1.14	1.08	1.05	1.12	1.15	1.09	1.28
ILMN_1373531	Cyb561d2	-1.17	-1.01	-1.13	-1.11	1.18	1.13	1.28
ILMN_1354870	Vamp4_predicted	-1.01	1.13	1.24	1.16	1.33	1.14	1.28
ILMN_1351559	Lrba_predicted	-1.04	1.04	-1.09	-1.03	-1.05	1.16	1.28
ILMN_1372152	Ddef1_predicted	1.12	1.16	-1.02	1.06	1.1	1.19	1.28
ILMN_1366118	Ckap1_predicted	-1.01	-1.01	-1.04	1.22	1.41	1.2	1.28
ILMN_1353210	Nipsnap1	1.05	1.99	1.87	1.64	1.24	1.21	1.28

ILMN_1361775	Rpl26	1.04	1.18	1.08	1.21	1.12	1.24	1.28
ILMN_1360033	Rpl26	1.04	1.18	1.08	1.21	1.12	1.24	1.28
ILMN_1359160	Ccnt2_predicted	-1.01	1.26	1.38	1.43	1.46	1.25	1.28
ILMN_1373412	Rps11	-1.04	1.22	-1.02	1.31	1.23	1.26	1.28
ILMN_1368590	Cldnd1	-1.1	-1.28	-1.07	-1.07	1.14	1.27	1.28
ILMN_1350095	Mrps35_predicted	1.12	1.62	1.38	1.54	1.21	1.27	1.28
ILMN_1371992	Arv1_predicted	1.07	1.52	1.46	1.68	1.25	1.29	1.28
ILMN_1358845	Rpl35	1.16	1.21	-1.19	1.23	1.26	1.3	1.28
ILMN_1353818	Pex3	-1.01	-1.04	1.09	-1.17	1.33	1.31	1.28
ILMN_1369730	Sidt2_predicted	-1.02	1.16	-1.41	-1.06	1.35	1.31	1.28
ILMN_1350175	Pfdn1_predicted	-1.04	1.2	-1.01	1.39	1.39	1.31	1.28
ILMN_1371194	LOC302497	1.32	1.06	-1.19	1.34	1.17	1.32	1.28
ILMN_1361603	Vil2	1.2	1.51	1.46	1.73	1.36	1.32	1.28
ILMN_1358613	Rwdd1	1.09	1.25	1.4	1.58	1.26	1.35	1.28
ILMN_1356599	Isg20l2	1.07	1.51	1.61	1.79	1.27	1.35	1.28
ILMN_1349748	Zfml_predicted	1.03	1	-1.03	-1.02	1.35	1.36	1.28
ILMN_1349760	Pygl	-1.11	1.12	1.2	1.37	1.46	1.4	1.28
ILMN_1365091	Blcap	-1.02	1.3	1.46	1.47	1.39	1.42	1.28
ILMN_1374343	Ghitm	1.1	1.26	1.64	1.22	1.27	1.44	1.28
ILMN_1369093	RGD1561808_predicted	1	1.22	1.19	1.28	1.33	1.44	1.28
ILMN_1354141	Lrrfip2	1.21	1.33	1.33	1.51	1.51	1.44	1.28
ILMN_1352431	Itsn1	-1.03	1.48	1.31	1.39	1.4	1.45	1.28
ILMN_1361004	Yipf3	1.1	1.12	-1.02	1.12	1.1	1.49	1.28
ILMN_1365663	Bcl6_predicted	1.14	1.01	-1.03	1.07	1.7	1.66	1.28
ILMN_1368809	Btg2	-1.11	1.57	1.14	-1.11	2.23	2.47	1.28
ILMN_1355223	RGD1306839_predicted	-1.1	-1.15	-1.12	-1.23	-1.09	-1.22	1.29
ILMN_1368610	Gchfr	1.13	1.09	-1.37	-1.27	1.01	-1.11	1.29
ILMN_1356289	Bcap31	-1.04	-1.45	1.17	-1.12	-1.11	-1.06	1.29
ILMN_1362604	Vnn1	1.17	-1.08	-1.14	-1.02	-1.09	-1.05	1.29
ILMN_1370995	Thnsl1	1.24	1.22	1.21	1.1	1.11	1.03	1.29
ILMN_2039586	LOC366554	-1.1	-1.03	-1.02	1.07	1.09	1.05	1.29
ILMN_1370796	RGD1565064_predicted	1.06	1.48	1.47	1.79	1.16	1.07	1.29
ILMN_1356049	Usp8_predicted	-1.04	-1.21	-1.02	-1.3	-1.05	1.09	1.29
ILMN_1358144	Hspca	-1.52	-1.39	-1.33	1.35	1.05	1.11	1.29
ILMN_1369783	Adsl_predicted	1.16	1.3	1.68	1.32	1.11	1.16	1.29
ILMN_1349198	Itm2c	1.09	1.08	1.09	-1.02	1.16	1.16	1.29
ILMN_1373950	RGD1311424_predicted	1.26	1.68	1.47	1.8	1.22	1.16	1.29
ILMN_1650168	LOC679898	-1.05	-1.38	1.08	-1.05	1.08	1.19	1.29

ILMN_1649891	RGD1563544_predicted	1.04	1.47	1.11	1.39	1.65	1.22	1.29
ILMN_1359238	Rock2	1.41	1.29	1.09	1.15	1.24	1.23	1.29
ILMN_1363289	Rdx	1.26	1.18	1.08	1.19	1.28	1.23	1.29
ILMN_1365029	Smpd2	-1.02	1.2	1.26	1.17	1.34	1.23	1.29
ILMN_1358514	Eif2s1	-1.1	1.14	1.36	1.3	1.21	1.24	1.29
ILMN_1359854	Ptk2	1.06	1.06	1.15	1.29	1.11	1.25	1.29
ILMN_1372411	Mpi_mapped	1.08	1.21	1.59	1.25	1.34	1.27	1.29
ILMN_1376313	Txndc4	-1.01	1.13	1.29	1.21	1.29	1.3	1.29
ILMN_1358278	Tyro3	1.01	1.89	1.78	1.74	1.2	1.31	1.29
ILMN_1349150	RGD1565099_predicted	1.05	1.17	1.16	1.26	1.23	1.31	1.29
ILMN_1358938	RGD1562923_predicted	1.12	1.45	-1.02	1.48	1.21	1.34	1.29
ILMN_1372455	RGD1359108	1.01	1.64	1.56	1.75	1.54	1.35	1.29
ILMN_1369699	LOC499830	-1	1.26	1.14	1.19	1.01	1.37	1.29
ILMN_1349378	Tsg101	1.08	1.34	1.52	1.28	1.4	1.38	1.29
ILMN_1372919	Cyr61	1.26	1.02	-1.09	-1.06	1.09	1.39	1.29
ILMN_1368869	RGD1565346_predicted	1.17	1.31	1.42	1.67	1.17	1.44	1.29
ILMN_1358419	MGC94190	1.03	1.29	1.34	1.14	1.61	1.46	1.29
ILMN_1358626	RGD1562502_predicted	-1.04	1.12	1	1.38	1.41	1.47	1.29
ILMN_1356291	RGD1305179_predicted	1.04	1.34	1.17	1.29	1.53	1.48	1.29
ILMN_1356102	Tanc1	1.13	1.78	1.5	1.49	1.32	1.5	1.29
ILMN_1362110	RGD1561102_predicted	1.1	1.09	-1.04	1.44	1.76	1.53	1.29
ILMN_1358771	Reps1_predicted	1.19	2.07	2.24	1.96	1.54	1.63	1.29
ILMN_1350277	Rhpn1_predicted	-1.01	1.09	1.16	1.13	-1.18	-1.4	1.3
ILMN_1354201	Fah	-1.17	1.06	-1.13	-1.45	-1.03	-1.32	1.3
ILMN_1349187	RGD1560656_predicted	1.03	1.02	1.35	1.11	-1.19	-1.15	1.3
ILMN_1370689	Peci	-1.15	-1.43	-1.13	-1.19	-1.15	-1.15	1.3
ILMN_1362437	Ramp2	1.18	1.51	1.64	1.79	1.14	-1.01	1.3
ILMN_1356042	RGD1307947	1.06	1.11	1.03	1.11	1.03	1.03	1.3
ILMN_1651153	LOC680519	1.2	1.17	1.16	1.2	1.16	1.03	1.3
ILMN_1373160	Nif3l1_predicted	1.02	1.19	1.56	1.48	1.05	1.07	1.3
ILMN_1363295	Tceal1	-1.04	-1.02	-1.06	-1.41	1.07	1.08	1.3
ILMN_1349142	RGD1562501_predicted	1.12	1.02	-1.39	-1	1.22	1.08	1.3
ILMN_1366921	RGD1308723_predicted	1.22	1.41	1.64	1.61	1.09	1.1	1.3
ILMN_1351877	Rab2b	-1.12	1.07	-1.03	1.05	1.14	1.11	1.3
ILMN_1373066	Tfpi	1.06	1.1	1.3	1.02	-1.01	1.12	1.3
ILMN_1355648	Xpa_predicted	1.17	1.53	1.18	1.24	1.11	1.15	1.3
ILMN_1366987	Psmc6	-1.15	-1.03	1.05	-1.02	1.22	1.15	1.3
ILMN_1364926	RGD1560646_predicted	1.11	1.3	1.24	-1.02	1.28	1.17	1.3

ILMN_1369547	Nudt5	1.08	1.14	1.03	1.11	1.01	1.19	1.3
ILMN_2040576	LOC683907	-1.01	-1.02	1.1	1.14	1.21	1.19	1.3
ILMN_1362290	Rcn2	-1.01	1.19	1.35	1.27	1.18	1.21	1.3
ILMN_1372922	Gpr21_predicted	1.14	1.08	1.16	1.1	1.35	1.23	1.3
ILMN_1375069	Gnb1	1.02	-1.3	-1.2	-1.22	1.29	1.24	1.3
ILMN_1363224	Mrpl1_predicted	-1.05	1.15	-1.13	1.04	1.19	1.25	1.3
ILMN_1369995	Bcat2	1.07	1.24	1.32	1.23	1.41	1.25	1.3
ILMN_1361629	Fbxw5	1.12	-1.05	1.22	-1.04	-1.06	1.26	1.3
ILMN_1350246	Rab24	1.06	1.44	1.54	1.4	1.29	1.27	1.3
ILMN_1359090	LOC305913	1.15	1.55	1.34	1.58	1.44	1.28	1.3
ILMN_1356041	Nsun2_predicted	1.22	1.53	1.71	1.71	1.15	1.31	1.3
ILMN_1373638	Slc38a6	1.11	1.2	1.54	1.19	1.22	1.32	1.3
ILMN_1368412	Jmjd3_predicted	1.07	1.51	1.09	1.35	1.42	1.32	1.3
ILMN_1374646	Socs6_predicted	-1.04	1.36	1.45	1.34	1.3	1.33	1.3
ILMN_1368020	Paip1_predicted	1.12	1.14	1.45	1.16	1.09	1.34	1.3
ILMN_1355376	Ercc5_mapped	-1.01	1.19	1.01	-1.13	1.14	1.34	1.3
ILMN_1368423	Fbxl5_predicted	1.29	1.92	1.45	1.82	1.32	1.34	1.3
ILMN_1363673	RGD1563124_predicted	1.06	1.46	-1.17	1.44	1.34	1.34	1.3
ILMN_1363514	RGD1563124_predicted	1.06	1.46	-1.17	1.44	1.34	1.34	1.3
ILMN_1352680	RGD1308470	-1.09	1.16	1.26	1.27	1.39	1.39	1.3
ILMN_1362591	RGD1564308_predicted	-1.03	-1.01	1.26	1.29	1.26	1.41	1.3
ILMN_1354400	Spag9_predicted	1.03	1.55	1.11	1.43	1.11	1.43	1.3
ILMN_1374519	Picalm	1.22	1.13	1.58	1.58	1.35	1.43	1.3
ILMN_1361040	RGD1560708_predicted	1.04	1.12	1.2	1.06	1.62	1.46	1.3
ILMN_1363655	RGD1309906	1.01	1.08	1.05	1.08	1.71	1.46	1.3
ILMN_1371140	Cope_predicted	-1.07	-1.22	-1.03	1.16	1.31	1.47	1.3
ILMN_2038894	Eprs	1.09	1.25	-1.02	1.25	1.47	1.47	1.3
ILMN_1530409	Ier5	-1.06	1.11	1.34	1.54	1.28	1.53	1.3
ILMN_1369883	RGD1564781_predicted	-1.02	-1.01	1.41	1.32	1.23	1.58	1.3
ILMN_1367801	Sec61a1	1.11	1.13	1.38	1.31	1.67	1.61	1.3
ILMN_1361722	Rbm8_predicted	-1.11	-1.06	1.05	-1.12	1.51	1.68	1.3
ILMN_1365404	Swap70_predicted	1.36	1.87	1.86	1.93	1.66	1.71	1.3
ILMN_1354983	LOC497846	1.15	-1.04	1.42	1.1	1.75	1.71	1.3
ILMN_1366775	Dusp8_predicted	-1.05	1.31	1.2	1.33	1.96	1.78	1.3
ILMN_1376396	Tfg	-1.15	1.26	1.21	1.38	2.23	2.1	1.3
ILMN_1369277	Isg20	1	1.07	1.25	1.04	2.32	2.78	1.3
ILMN_1369191	Thoc2_predicted	-1.12	1.17	-1.1	-1.03	-1.12	-1.14	1.31
ILMN_1376284	Calm1	-1.15	-1.47	-1.37	-1.28	1.17	-1.05	1.31

ILMN_1376547	Scyl1	-1.18	1.05	1.19	1.17	1.2	-1.05	1.31
ILMN_1369572	Stard3nl	1.02	-1.06	-1.03	-1.05	1.02	1.01	1.31
ILMN_1354640	RGD1309730_predicted	-1.09	-1.16	-1.13	-1.76	1.05	1.03	1.31
ILMN_1354052	Hist2h4_predicted	1.11	-1.26	-1.45	-1.05	-1.22	1.04	1.31
ILMN_1364242	Nanp	1.06	1.11	1.28	1.36	1.02	1.05	1.31
ILMN_1366841	Ddx49_predicted	1.04	1.2	1.13	1.33	1.12	1.06	1.31
ILMN_1350797	LOC300731	1.22	1.3	-1.04	1.32	1.03	1.07	1.31
ILMN_1368615	LOC683686	1.04	1.22	1.31	1.18	1.18	1.08	1.31
ILMN_1374521	RGD1308261_predicted	1.07	1.07	1.13	1.23	1.15	1.09	1.31
ILMN_1349491	Sfrs9	-1.02	1.3	1.28	1.43	1.16	1.09	1.31
ILMN_1350668	RGD1359380	1.01	1.21	1.18	1.03	1.13	1.1	1.31
ILMN_1353529	Ccdc23	1.03	-1.01	-1.17	1.26	1.09	1.12	1.31
ILMN_2040930	LOC679081	1.06	1.12	1.32	1.21	1.17	1.14	1.31
ILMN_1366608	Stambp	-1.41	-1.01	1.06	-1.12	1.26	1.14	1.31
ILMN_1357104	LOC498786	1.09	1.27	1.37	1.49	1.14	1.15	1.31
ILMN_1351266	Ddx24	-1.07	1.3	1.34	1.2	1.17	1.18	1.31
ILMN_1353292	RGD1565095_predicted	-1	1.12	-1.1	1.11	1.25	1.19	1.31
ILMN_1358223	RGD1565358_predicted	1.19	1.18	-1.44	1.17	1.15	1.22	1.31
ILMN_1372609	Bcl2l2	-1.01	1.4	1.14	1.41	1.2	1.24	1.31
ILMN_1374672	Nol5	1.2	1.5	1.57	1.57	1.46	1.24	1.31
ILMN_1349418	RGD1562579_predicted	-1.07	1.17	1.56	1.38	1.22	1.25	1.31
ILMN_1361472	LOC500959	1.06	-1.05	1.95	1.25	1.36	1.29	1.31
ILMN_1355142	LOC500959	1.06	-1.05	1.95	1.25	1.36	1.29	1.31
ILMN_1376783	Samd8	1.06	1.4	1.43	1.41	-1.02	1.3	1.31
ILMN_1371000	Dnajb4	1.18	-1.21	1.08	-1.33	1.09	1.3	1.31
ILMN_1373092	Tsc2	-1.03	1.24	1.33	1.35	1.09	1.31	1.31
ILMN_1354762	LOC306766	-1.01	1.22	1.04	1.07	1.43	1.31	1.31
ILMN_1369753	RGD1566326_predicted	1.09	1.28	1.02	1.43	1.31	1.33	1.31
ILMN_1354728	RGD1564698_predicted	1.1	1.32	1.04	1.38	1.37	1.34	1.31
ILMN_1366557	RGD1564698_predicted	1.1	1.32	1.04	1.38	1.37	1.34	1.31
ILMN_1376672	Dync1h1	1.15	1.28	1.17	1.28	1.27	1.37	1.31
ILMN_1365742	Npuk68	1.05	1.76	1.53	1.5	1.35	1.37	1.31
ILMN_1352574	Commd4_predicted	-1.14	1.45	1.21	1.16	1.3	1.38	1.31
ILMN_1364052	Arhgdia	-1.07	1.03	1.03	1.35	1.33	1.38	1.31
ILMN_1376472	Irgm	1.08	1.18	1.19	1.32	1.33	1.4	1.31
ILMN_1361781	Srpr	1	1.23	1.03	-1.03	1.6	1.5	1.31
ILMN_1373266	Itm2b	-1.09	-1.32	1.19	-1.58	1.53	1.56	1.31
ILMN_1350087	Ns5atp4	1.05	1.23	1.56	1.09	1.24	1.57	1.31

ILMN_1352891	RGD1309102_predicted	1.09	1.62	1.23	1.39	1.64	1.62	1.31
ILMN_1359903	LOC498453	1.2	1.53	2.2	1.64	1.69	1.63	1.31
ILMN_1371058	LOC498453	1.2	1.53	2.2	1.64	1.69	1.63	1.31
ILMN_1350560	Clcn3	1.14	1.32	1.4	1.41	2.15	2.11	1.31
ILMN_1363621	MGC94010	-1.06	-1.34	-1.3	-1.16	-1.23	-1.31	1.32
ILMN_1358198	Edf1_predicted	1.07	-1.15	-1.19	-1.03	-1.16	-1.17	1.32
ILMN_1650288	LOC682248	1.15	1.99	1.63	2.11	1	-1.14	1.32
ILMN_1363448	Slc39a4_predicted	1.03	-1.61	-1.41	-2.21	1.08	-1.12	1.32
ILMN_1375152	Sh3bgrl3_predicted	-1.18	-1.23	-1.38	-1.18	-1.08	-1.1	1.32
ILMN_1358976	Taldo1	1.22	-1.23	-1.12	-1.14	-1.15	-1.05	1.32
ILMN_1349833	Nudt7_predicted	1.05	1.11	-1.24	-1.01	-1.09	-1.05	1.32
ILMN_1651186	Glrx2	1.13	1.08	-1.28	-1.2	1.07	-1.04	1.32
ILMN_1354855	Gas2l1_predicted	-1	1.57	1.1	1.57	1.07	1.07	1.32
ILMN_1368464	LOC497720	-1.17	-1.11	1.06	1.18	-1.21	1.08	1.32
ILMN_1357021	LOC363332	-1.12	1.06	-1.12	-1.19	-1.03	1.09	1.32
ILMN_1373564	RGD1305687_predicted	-1.05	-1.06	-1.69	-1.14	1.3	1.09	1.32
ILMN_1356807	RGD1563812_predicted	-1	1.25	1.13	1.09	1.09	1.13	1.32
ILMN_1362723	Flnb_predicted	1.08	1.24	-1.04	1.3	1.32	1.15	1.32
ILMN_1363531	LOC498427	1.16	1.14	-1.13	1.02	1.08	1.16	1.32
ILMN_1370986	RGD1304687_predicted	1.18	1.2	1.28	1.3	1.13	1.16	1.32
ILMN_1649846	LOC364236	1.03	1.11	-1.08	1.1	1.2	1.16	1.32
ILMN_1366686	Clns1a	1.11	-1.03	1.2	1.12	1.05	1.19	1.32
ILMN_1374079	RGD1308284_predicted	1.15	1.15	1.19	1.13	1.16	1.2	1.32
ILMN_1370488	Inpp4a	-1.14	1.01	-1.39	1.11	1.01	1.24	1.32
ILMN_1353724	RGD1304927_predicted	1.05	1.57	1.33	1.65	1.24	1.24	1.32
ILMN_1370020	Acn9	1.23	1.28	-1.16	1.18	1.41	1.25	1.32
ILMN_1350119	RGD1565165_predicted	1.05	1.13	1.35	1.23	1.09	1.27	1.32
ILMN_1350303	RGD1565165_predicted	1.05	1.13	1.35	1.23	1.09	1.27	1.32
ILMN_1361285	LOC361048	-1.01	-1.01	1.04	1.04	1.17	1.27	1.32
ILMN_1353378	RGD1560376_predicted	1.29	1.15	1.02	1.15	1.4	1.27	1.32
ILMN_1375214	Rpia_predicted	1.19	1.27	1.83	1.58	1.2	1.3	1.32
ILMN_1375149	Suhw3_predicted	1.26	1.95	1.9	1.81	1.14	1.31	1.32
ILMN_1349318	Tmem49	1.08	1.94	1.94	1.51	1.26	1.31	1.32
ILMN_1357417	Scye1	-1.06	1.15	1.27	1.24	1.28	1.31	1.32
ILMN_1373785	Pcsk4	1.02	1.61	1.21	1.79	1.36	1.31	1.32
ILMN_1369141	RGD1304686	1.06	-1.01	1.05	1.34	1.48	1.32	1.32
ILMN_1376831	Ubxd2	1.18	1.25	1.49	1.36	1.3	1.33	1.32
ILMN_1358594	LOC498979	1.17	1.01	1.05	1.08	1.21	1.34	1.32

ILMN_1369893	Wdr20_predicted	1.03	1.25	1.04	1.23	1.39	1.35	1.32
ILMN_1351563	RGD1560911_predicted	-1.1	1.35	1.32	1.2	1.36	1.36	1.32
ILMN_1363201	RGD1563867_predicted	1.04	1.41	-1.28	1.24	1.41	1.37	1.32
ILMN_1356037	LOC501413	-1.02	-1.16	-1.15	-1.01	1.2	1.4	1.32
ILMN_1373475	Ripk5	-1.01	1.23	1.25	1.17	1.27	1.41	1.32
ILMN_1366443	LOC363418	-1.16	1.57	1.43	1.44	1.51	1.42	1.32
ILMN_1371388	LOC361309	-1.01	-1.16	-1.04	-1.39	1.13	1.44	1.32
ILMN_1361860	Tbc1d23_predicted	1	-1.06	-1.26	-1.19	1.64	1.47	1.32
ILMN_1349996	RGD1311463	1.14	1.92	1.82	1.51	1.3	1.48	1.32
ILMN_1353734	Orc4l	-1.01	1.47	1.64	1.34	1.37	1.48	1.32
ILMN_1360454	Wdr12	1.32	1.59	1.91	1.66	1.36	1.51	1.32
ILMN_1351218	RGD1308706_predicted	1.04	2.12	2.84	2.05	1.84	1.55	1.32
ILMN_1350131	Zfp57	1.11	1.1	1.01	1.04	1.82	1.63	1.32
ILMN_1360053	Slc27a4	1.03	1.47	1.31	1.67	1.81	1.84	1.32
ILMN_1530238	LOC684681	1.29	1.07	1.25	1.31	1.47	1.93	1.32
ILMN_1350174	Rab3d	1.01	1.02	1.11	1.02	1.09	-1.37	1.33
ILMN_1351313	Mgst3_predicted	1.21	-1.09	-1.27	-1.26	1.06	-1.11	1.33
ILMN_1371474	Tmem106b	1.06	-1.1	1.05	-1.21	-1.07	-1.08	1.33
ILMN_1362377	Dap	1.14	1.52	1.3	-1.02	1.16	-1.04	1.33
ILMN_1369107	Pkp2	1.02	1.44	1.06	1.4	1.23	1.03	1.33
ILMN_1362671	Zc3h6_predicted	1.04	1.07	1.13	1.07	-1.03	1.05	1.33
ILMN_1352882	Aurkaip1	1.08	1.06	1.09	1.16	1.08	1.05	1.33
ILMN_1376530	RT1-A3	1.07	-1.12	1.2	1.07	1.17	1.06	1.33
ILMN_1369971	Pllp	1.21	1.12	1.13	1.26	1.13	1.07	1.33
ILMN_1366361	Fez2	1.24	-1	1.33	-1.02	1.16	1.08	1.33
ILMN_1365731	Pus7_predicted	1.29	1.5	1.61	2.21	1.04	1.1	1.33
ILMN_1371591	Eno1	-1.26	-1.73	1.13	-1.08	1.05	1.13	1.33
ILMN_1366973	LOC364139	1.13	1.3	1.06	1.47	1.3	1.13	1.33
ILMN_1357880	Sncg	1.13	1.05	-1.17	-1.03	1.52	1.13	1.33
ILMN_1356386	Pon3	1.01	1.32	1.04	1.1	1.73	1.13	1.33
ILMN_1357309	Cfi	1.12	1.57	1.56	1.08	1.12	1.14	1.33
ILMN_1348870	Flot1	-1.03	1.66	1.38	1.36	1.35	1.15	1.33
ILMN_1351947	Rab5c_predicted	1.01	-1.31	-1.22	-1.01	1.09	1.16	1.33
ILMN_1351951	RGD1310147_predicted	-1.02	1.69	1.71	1.53	1.2	1.16	1.33
ILMN_1351247	Ube2d1_predicted	1.14	1.1	-1.21	1.18	1.16	1.18	1.33
ILMN_1364820	MGC108776	-1.03	1.16	1.2	1.06	1.17	1.18	1.33
ILMN_1350308	Pgpep1	1.09	1.18	1.18	1.17	1.41	1.19	1.33
ILMN_1349109	RGD1306332_predicted	1.24	1.28	1.61	1.41	1.29	1.2	1.33

ILMN_1369478	XRG9	1.03	1.36	1.14	1.09	1.43	1.24	1.33
ILMN_1369703	RGD1564228_predicted	1.02	1.34	2.12	1.51	1.24	1.28	1.33
ILMN_1349645	LOC499569	1.01	-1.1	1.07	-1.03	1.47	1.3	1.33
ILMN_1364097	Cerk_predicted	1.38	1.8	1.78	1.84	1.53	1.35	1.33
ILMN_1361833	Gnpda2_predicted	1.18	1.04	-1.05	-1.07	1.43	1.38	1.33
ILMN_1363944	Sms	1.14	1.17	1.48	1.5	1.22	1.39	1.33
ILMN_1364467	RGD1561886_predicted	1.13	1.27	1.06	1.39	1.34	1.39	1.33
ILMN_1367022	LOC501648	1.11	1.07	1.21	-1.04	1.57	1.39	1.33
ILMN_1349699	Ggnbp1	-1.05	1.56	1.39	1.5	1.52	1.41	1.33
ILMN_1350840	Myo5a	1.06	1.39	1.53	1.56	1.56	1.42	1.33
ILMN_1350876	RGD1563395_predicted	1.05	1.32	1.5	1.66	1.59	1.44	1.33
ILMN_1351237	Txnrd1	1.8	1.16	1.23	1.6	1.46	1.51	1.33
ILMN_1354991	LOC499837	1.22	1.48	1.15	1.31	1.53	1.53	1.33
ILMN_1363155	Plekhb2_predicted	-1.06	1.03	1.03	1.16	1.61	1.54	1.33
ILMN_1355940	Fip1l1	1.11	1.1	1.7	1.47	1.45	1.56	1.33
ILMN_1376558	Bbs7	1.14	1.18	1.21	1.55	1.62	1.6	1.33
ILMN_1363637	Vcp	-1.06	1.04	1.08	1.15	1.65	1.61	1.33
ILMN_1356848	Btg1	-1.09	1.6	1.57	1.07	1.89	2.15	1.33
ILMN_1372185	RGD1566118_predicted	1.35	2.59	1.79	2.53	2.65	2.56	1.33
ILMN_1352039	Gsta4	-1.08	-1.08	-1.36	-1.42	-1.28	-1.36	1.34
ILMN_1351415	Ndufb3_predicted	1.03	-1.12	-1.55	-1.13	1.02	-1.01	1.34
ILMN_1359734	LOC295419	-1.03	1.03	1.03	1.26	1.17	1.01	1.34
ILMN_1358458	RGD1565691_predicted	1.17	1.46	1.25	1.06	1.02	1.03	1.34
ILMN_1352178	Zdhhc13	1.26	1.23	1.62	1.68	1.09	1.04	1.34
ILMN_1356915	Slc9a3r2	-1.05	1.33	1.14	1.09	-1.01	1.05	1.34
ILMN_1362282	Epas1	1.13	1.27	1.24	1.29	1.19	1.08	1.34
ILMN_1372579	Slc22a5	1.08	-1.08	-1.02	1.06	1.3	1.08	1.34
ILMN_1364444	Cenpb_predicted	1.11	1.64	1.3	1.48	1.19	1.12	1.34
ILMN_1368071	Ube1c	-1.11	1.02	1.22	-1.07	1.26	1.12	1.34
ILMN_1367659	Tat	1.05	1.31	1.08	1.33	1.43	1.13	1.34
ILMN_1355205	Kars	-1.02	1.21	1.2	1.24	1.3	1.14	1.34
ILMN_1354734	Rnf12	-1.14	1.4	1.62	1.52	1.14	1.16	1.34
ILMN_1365694	Mfn2	1.18	1.05	1.01	1.37	1.25	1.17	1.34
ILMN_1348830	Cyb5r4	-1.05	-1.03	1.13	1.04	1.28	1.19	1.34
ILMN_1364188	Dnalc4	-1.05	1.11	-1.16	1.22	1.22	1.2	1.34
ILMN_1368345	LOC290999	1.2	-1.01	-1.36	1.16	1.23	1.22	1.34
ILMN_2039346	Hla-dma	1.07	1.1	1.21	1.27	1.24	1.23	1.34
ILMN_1350533	RGD1563551_predicted	1.13	1.27	1.03	1.26	1.18	1.26	1.34

ILMN_1364753	Asah3l_predicted	1.07	1.2	1.18	1.56	1.23	1.27	1.34
ILMN_1349047	LOC681314	1.12	1.02	3.01	1.29	-1.03	1.28	1.34
ILMN_1650140	RGD1562652_predicted	-1.01	1.28	1.61	1.38	1.19	1.28	1.34
ILMN_1369149	RGD1564784_predicted	1	1.26	1.19	1.17	1.29	1.28	1.34
ILMN_1373086	LOC361639	1.17	1.33	1.71	1.68	1.41	1.3	1.34
ILMN_1358666	Rab3gap2	1	1.45	1.23	1.59	1.55	1.3	1.34
ILMN_1362448	LOC499779	1.12	1.21	-1.38	1.16	1.33	1.31	1.34
ILMN_1367003	Cox7a2l_predicted	1.08	1.34	1.53	1.47	1.41	1.32	1.34
ILMN_1352175	Bnip1	1.27	1.38	1.36	1.28	1.36	1.33	1.34
ILMN_1354654	Zfp592_predicted	1.08	1.22	1.13	1.21	1.18	1.34	1.34
ILMN_1376697	RT1-Ke4	1.19	1.66	1.74	1.49	1.35	1.34	1.34
ILMN_1362113	Pak1	1.05	1.41	1.58	1.47	1.5	1.34	1.34
ILMN_1365837	RGD1306101_predicted	-1.09	1.19	1.07	1.37	1.67	1.35	1.34
ILMN_1368229	LOC498425	-1.02	1.18	1.2	1.14	-1.01	1.36	1.34
ILMN_1351625	Zpbp2	-1	1.06	1.27	-1.03	1.47	1.36	1.34
ILMN_1353774	Rpl24	1.3	1.76	1.17	1.71	1.44	1.43	1.34
ILMN_1353840	Mrrf	1.23	1.3	1.02	1.07	1.49	1.44	1.34
ILMN_1361708	Fbln5	-1.42	1.75	1.7	1.57	1.17	1.45	1.34
ILMN_1649928	Gorasp2	-1.05	1.03	1.18	1.07	1.45	1.47	1.34
ILMN_1365777	Gorasp2	-1.05	1.03	1.18	1.07	1.45	1.47	1.34
ILMN_1358269	LOC305633	1.03	1.42	1.41	1.45	1.37	1.48	1.34
ILMN_1364855	LOC498131	1.23	1.39	1.24	1.43	1.29	1.5	1.34
ILMN_1363333	LOC498171	1.02	1.5	1.39	1.43	1.47	1.51	1.34
ILMN_1369015	Mnat1	1.12	1.57	1.62	1.7	1.66	1.51	1.34
ILMN_1371918	Aff4_predicted	1.1	1.42	1.22	1.31	1.67	1.55	1.34
ILMN_1376606	Anxa6	-1.1	1.09	-1.06	1.27	1.67	1.59	1.34
ILMN_1368841	Lrrc59	1.23	1.33	1.87	1.66	1.77	1.64	1.34
ILMN_1355152	Ddx50	1.14	1.87	1.8	1.81	1.86	1.73	1.34
ILMN_1358748	RGD1307394_predicted	-1.05	1.13	1.42	1.36	1.56	1.76	1.34
ILMN_1351816	Rab6ip1_predicted	1.01	1.34	1.18	1.49	1.78	1.77	1.34
ILMN_1376738	Wars	1.13	1.32	2.34	2.01	2.11	2.06	1.34
ILMN_1370868	Herpud1	1.18	1.64	1.14	1.04	3.01	3.15	1.34
ILMN_1367433	RGD1566239_predicted	-1.07	1.07	-1.37	-1.05	-1.01	-1.24	1.35
ILMN_1371515	Gadd45gip1	-1.1	-1.06	-1.2	1.1	-1.18	-1.18	1.35
ILMN_1351082	Znf593_predicted	1.18	1.62	-1.15	1.59	-1.21	-1.07	1.35
ILMN_1376421	Mrps10	1.14	-1.13	1.2	-1	-1.01	-1.03	1.35
ILMN_1361954	Pkn1	-1.07	1.93	1.33	1.51	1.06	1.03	1.35
ILMN_1650956	Hig1	-1.18	1.23	-1.13	1.27	1.31	1.03	1.35

ILMN_1366963	LOC292074	1.14	-1.03	-1.08	-1.03	1.14	1.04	1.35
ILMN_1369382	Ldlr	1.01	-1.06	1.02	1.11	1.16	1.07	1.35
ILMN_1376847	Zfp297	-1.09	1.44	-1.03	1.07	-1.01	1.08	1.35
ILMN_1362248	Trappc3	-1.01	1	-1.05	1.01	1.06	1.08	1.35
ILMN_1360197	RGD1565117_predicted	1.1	1.65	-1.61	1.51	1.29	1.08	1.35
ILMN_1350912	Chchd4	1.22	1.24	1.41	1.63	1.13	1.12	1.35
ILMN_1348916	LOC361786	1.05	1.23	1.07	1.17	1.27	1.13	1.35
ILMN_1367951	RGD1308697	1.09	1.47	1.48	1.37	1.37	1.13	1.35
ILMN_1376814	RGD1311196	-1	1.26	1.28	1.06	1	1.15	1.35
ILMN_1365519	Pmpcb	1.03	-1.02	1.11	-1.1	1.03	1.15	1.35
ILMN_1359878	Gca_predicted	1.01	1.11	1.04	-1.03	1.34	1.15	1.35
ILMN_1355708	Pftk1_predicted	1.47	1.14	1.27	1.27	1.33	1.16	1.35
ILMN_1365988	RGD1309624_predicted	1.06	1.17	1.03	-1.13	1.1	1.17	1.35
ILMN_1369671	LOC367088	-1.07	-1.08	-1.18	-1.08	1.06	1.18	1.35
ILMN_1374916	Rpl23	1.24	1.49	1.04	1.37	1.27	1.21	1.35
ILMN_1375856	Epb4.1l1	1.04	1.11	1.14	1.11	1.18	1.22	1.35
ILMN_1372512	RGD1559877_predicted	1.17	1.39	-1.13	1.23	1.1	1.24	1.35
ILMN_1353413	RGD1308106_predicted	1.06	1.27	1.17	1.13	1.36	1.24	1.35
ILMN_1356171	Gyg1	1.15	1.35	1.27	1.45	1.28	1.25	1.35
ILMN_1352269	RT1-149	-1.03	1.18	1.07	1.22	1.35	1.25	1.35
ILMN_1348879	Slc35e4	1.05	1.17	1.14	1.4	1.19	1.29	1.35
ILMN_1363891	Timm8a	1.36	1.32	1.6	1.44	1.22	1.29	1.35
ILMN_1354236	Faf1	1.18	1.49	1.69	1.51	1.41	1.29	1.35
ILMN_1372468	Fads6_predicted	1.25	2.23	1.99	1.61	1.21	1.34	1.35
ILMN_1360136	Dhx36_predicted	1.03	1.8	1.48	1.64	1.32	1.34	1.35
ILMN_1348961	Ube4a	-1.03	-1.03	1	1.02	1.34	1.35	1.35
ILMN_1350731	Cct4	1.18	1.55	1.67	1.42	1.26	1.38	1.35
ILMN_1370774	RGD1563503_predicted	1.08	1.17	1.3	1.19	1.17	1.42	1.35
ILMN_1363971	RGD1307929	1.01	1.12	1.11	1.37	1.25	1.43	1.35
ILMN_1369845	Stx6	-1.01	1.54	1.17	1.43	1.52	1.47	1.35
ILMN_1369444	LOC361942	1.12	1.18	1.59	1.32	1.63	1.47	1.35
ILMN_1356810	Irf6_predicted	1.07	-1.14	-1.29	1.15	1.74	1.5	1.35
ILMN_2040780	Dd5	1.12	1.55	1.32	1.63	1.71	1.62	1.35
ILMN_1360660	Pim3	1.42	2.49	1.86	2.6	1.53	1.66	1.35
ILMN_1364012	RGD1311593_predicted	-1.04	1.22	1.3	1.26	1.79	1.69	1.35
ILMN_1363677	Copb1	1.01	1.38	1.12	1.27	1.91	1.77	1.35
ILMN_2039866	Copb1	1.01	1.38	1.12	1.27	1.91	1.77	1.35
ILMN_1368822	Dnajc3	1.06	1.6	1.72	1.77	1.91	1.79	1.35

ILMN_1368381	P4ha1	-1.1	1.19	1.79	1.61	1.94	1.88	1.35
ILMN_1368053	LOC361923	-1.1	1.07	-1.12	1.2	-1.06	-1.16	1.36
ILMN_1362960	RGD1310224	1.04	1.07	1.17	-1.14	1.11	1.01	1.36
ILMN_1350564	Abca2	1.13	1.42	1.13	1.3	-1.08	1.06	1.36
ILMN_1362103	LOC361288	1.06	1.08	1.35	1.07	1.24	1.07	1.36
ILMN_1374736	RGD1310925_predicted	1.08	1.11	-1.07	1.16	1.16	1.09	1.36
ILMN_1366903	Rps6ka4_predicted	-1.01	1.23	1	1.48	1.07	1.13	1.36
ILMN_1365552	RGD1561113_predicted	-1.07	-1.02	-1.51	1.11	1.31	1.13	1.36
ILMN_1360059	Sumf1_predicted	-1.23	-1.15	-1.39	-1.33	1.13	1.15	1.36
ILMN_1352707	Gna12	1.03	1.93	1.57	1.23	1.31	1.15	1.36
ILMN_1357165	Gata2	1.05	1.27	1.17	1.16	1.08	1.17	1.36
ILMN_1358877	RGD1311648	1.08	-1.05	1	1.18	1.32	1.18	1.36
ILMN_1375155	Wwp1	1.12	1.43	1.33	1.29	1.2	1.19	1.36
ILMN_1364889	Gls2	-1.02	1.3	1.3	1.39	1.38	1.2	1.36
ILMN_1371239	Tomm20	1.03	1.14	1.13	1.26	1.19	1.22	1.36
ILMN_1371306	Pigv	1	1.26	1.3	1.57	1.11	1.24	1.36
ILMN_1368324	Gpatc1_predicted	1.08	-1.04	-1.05	1.09	1.14	1.25	1.36
ILMN_1357175	RGD1563705_predicted	1.17	1.19	1.01	1.12	1.15	1.26	1.36
ILMN_1356377	RGD1310681_predicted	1	1.52	1.24	1.31	1.18	1.27	1.36
ILMN_1361493	Plekhf1	-1.09	1.3	1.29	1.2	1.29	1.27	1.36
ILMN_1376892	Zfp403	-1	1.69	1.33	1.48	1.37	1.31	1.36
ILMN_1352667	Nab1	1.19	1.3	1.18	1.26	1.35	1.33	1.36
ILMN_1370671	Usp15	1.16	1.34	1.42	1.63	1.16	1.34	1.36
ILMN_1650414	Fkbp7_predicted	1.08	1.3	1.13	1.39	1.31	1.34	1.36
ILMN_1651034	Papd5_predicted	1.15	1.39	1.31	1.4	1.53	1.35	1.36
ILMN_1365467	Rpl36a	1.17	1.61	1.1	1.61	1.35	1.38	1.36
ILMN_1651135	PNAS-4	1.2	1.03	1.11	1.19	1.52	1.38	1.36
ILMN_1364723	Coq10a_predicted	1.12	1.4	1.25	1.33	1.28	1.39	1.36
ILMN_1372644	RT1-M10-1	1.01	1.07	1.13	-1.01	1.37	1.4	1.36
ILMN_1368838	Srp72_predicted	1.11	1.42	1.35	1.34	1.38	1.4	1.36
ILMN_1370730	Srp72_predicted	1.11	1.42	1.35	1.34	1.38	1.4	1.36
ILMN_1365617	Samd4b	-1.15	1.21	-1.05	1.44	1.51	1.42	1.36
ILMN_1366897	Klf9	1.48	1.55	1.35	1.46	1.56	1.43	1.36
ILMN_1367313	Nr1h4	1.19	1.26	1.18	1.14	1.2	1.47	1.36
ILMN_1376715	Lypla3	1.11	1.34	1.72	1.11	1.38	1.47	1.36
ILMN_1368431	Ube4b_predicted	1.25	1.1	-1.02	-1.02	1.5	1.52	1.36
ILMN_1366384	Cbara1	1.12	1.22	1.58	1.22	1.42	1.53	1.36
ILMN_1369219	Trim41_predicted	1.04	1.17	1.19	1.21	1.48	1.63	1.36

ILMN_1360324	Kpna4	1.32	1.41	1.58	1.72	1.71	1.65	1.36
ILMN_1365236	Rnmt	1.18	1.11	1.25	1.08	1.65	1.73	1.36
ILMN_1351948	Pthr1	1.06	1.61	1.84	1.42	2.14	1.94	1.36
ILMN_1373577	Tpp2	1.05	1.68	1.45	1.65	2.35	2.15	1.36
ILMN_1364670	Mdm2_predicted	1.09	1.24	1.34	1.26	1.69	2.27	1.36
ILMN_1355217	Tmem66	-1.17	1.02	1.21	1.04	2.95	2.63	1.36
ILMN_1373599	RGD1560991_predicted	1.04	-1.06	-1.41	-1.01	1.01	-1.05	1.37
ILMN_1352381	Frag1	-1.03	1.26	1.07	1.1	1.05	1.02	1.37
ILMN_1356121	Dus2l_predicted	1.12	1.46	1.33	1.45	1.3	1.1	1.37
ILMN_1355193	LOC303057	-1	1.01	-1.12	-1.39	1.16	1.15	1.37
ILMN_1358238	MGC94881	-1.08	1.12	1.01	1.09	1.21	1.16	1.37
ILMN_1352105	Cry1	1.39	2.34	2.16	2.51	1.36	1.18	1.37
ILMN_1351859	LOC365960	1.26	1.05	1.17	1.12	1.19	1.2	1.37
ILMN_1650706	RGD1563508_predicted	1.07	1.18	1.11	1.19	1.3	1.2	1.37
ILMN_1365578	RGD1561055_predicted	1.23	1.34	1.63	1.16	1.15	1.21	1.37
ILMN_1356444	RGD1307907_predicted	-1.13	1.09	1.17	1	1.26	1.25	1.37
ILMN_1374368	RGD1566099_predicted	1.08	-1.22	-1.48	-1.23	1.18	1.28	1.37
ILMN_1349202	Dhcr7	1.23	-1.19	-1.11	-1.19	1.3	1.28	1.37
ILMN_1355470	Vcpip1	1.03	1.12	1.23	1.2	1.26	1.29	1.37
ILMN_1367537	Sgpp1	1.29	1.4	1.47	1.28	1.31	1.29	1.37
ILMN_1361267	LOC499941	1.16	1.53	1.22	1.47	1.19	1.31	1.37
ILMN_1358502	RGD1307883	1.11	1.45	1.27	1.59	1.28	1.31	1.37
ILMN_1363841	Mrpl23	-1	1.33	-1.08	1.4	1.39	1.31	1.37
ILMN_1366698	Vapb	1.01	1.11	1.92	1.52	1.26	1.33	1.37
ILMN_1358016	Kidins220	1.02	1.28	1.06	1.17	1.44	1.33	1.37
ILMN_1360708	Ppme1	1	1.01	1.13	1.29	1.45	1.34	1.37
ILMN_1367688	Tmbim4	1.08	1.06	-1.04	1.12	1.36	1.35	1.37
ILMN_1358946	Cotl1_predicted	1.08	1.52	1.33	1.75	1.77	1.35	1.37
ILMN_1650508	LOC503110	1.05	1.29	1.02	1.33	1.41	1.38	1.37
ILMN_1354729	LOC312654	1.11	1.24	1.32	1.33	1.53	1.38	1.37
ILMN_1370606	Lrp3	1.19	1.91	1.32	1.74	1.4	1.39	1.37
ILMN_1361818	Adprtl1	-1.11	1.12	1.11	-1.01	1.49	1.39	1.37
ILMN_1349770	Dyrk3	1.21	1.34	1.16	1.42	1.26	1.42	1.37
ILMN_1374964	Atp6v1e1	-1.03	1.29	1.53	1.27	1.46	1.44	1.37
ILMN_1358231	Plod1	1.09	1.4	1.24	1.42	1.5	1.47	1.37
ILMN_1359287	LOC310086	-1.16	1.27	1.38	1.47	1.6	1.52	1.37
ILMN_1352230	LOC362543	1.19	1.26	1.44	1.37	1.4	1.53	1.37
ILMN_1365946	Me1	1.49	1.14	1.2	1.32	1.56	1.56	1.37

ILMN_1363077	Tbc1d15	1.1	1.08	1.02	-1.11	1.33	1.6	1.37
ILMN_1367645	Adipor2_predicted	1.18	1.56	1.02	1.38	1.51	1.61	1.37
ILMN_1371856	Rlf_predicted	1.02	1.62	1.22	1.55	1.67	1.64	1.37
ILMN_1375128	Bnip3l	1.02	-1.25	-1.3	-1.69	1.76	1.64	1.37
ILMN_1376943	Hbs1l	1.07	1.32	1.16	1.32	1.66	1.65	1.37
ILMN_1366334	Iars_predicted	1.1	1.59	1.68	1.58	1.64	1.75	1.37
ILMN_1369410	Sec23b_predicted	1.08	1.25	1.16	1.34	1.89	1.79	1.37
ILMN_1369742	Kiaa0415	1.08	1.43	1.28	1.22	1.62	1.8	1.37
ILMN_1372254	LOC304923	-1.03	1.21	1.22	1.08	1.47	1.83	1.37
ILMN_1374121	Eif2ak3	1.03	-1.02	-1.13	-1.04	2.2	2.21	1.37
ILMN_1376625	Tra1_predicted	-1.04	-1.03	1.27	1.24	2.54	2.21	1.37
ILMN_1376484	Sep-09	1.11	1.32	1.14	1.67	1.41	1.31	1.37
ILMN_1363976	Ak2	1.01	-1.08	-1.29	-1.08	-1.23	-1.18	1.38
ILMN_1366102	Azi1_predicted	-1.02	1.2	1.03	-1	1.08	-1.13	1.38
ILMN_1355934	Mina	1.31	1.4	1.31	1.64	-1.01	-1.02	1.38
ILMN_1365436	C2	1.12	1.29	1.59	1.2	1.02	1.03	1.38
ILMN_1356759	RGD1566282_predicted	-1.03	1.04	1.07	1.15	1.34	1.03	1.38
ILMN_1371685	Eps8l3_predicted	1.06	1.61	1.37	1.44	1.37	1.08	1.38
ILMN_2038882	LOC498469	1.01	-1.15	1.06	1.01	1.12	1.09	1.38
ILMN_1365170	LOC689253	1.21	1.06	1.01	1.07	1.28	1.12	1.38
ILMN_1376652	Sh3glb1	1.05	1.13	1.08	-1.05	1.13	1.14	1.38
ILMN_1355172	RGD1305240_predicted	1.14	1.26	1.6	1.34	1.03	1.15	1.38
ILMN_2039472	LOC364582	1.02	1.15	1.24	1.26	1.13	1.18	1.38
ILMN_1358656	Hrpt2_predicted	1.01	1.12	-1.1	1.1	1.23	1.18	1.38
ILMN_1366690	Eef1e1_predicted	1.23	1.27	1.37	1.55	1.2	1.19	1.38
ILMN_1352965	Ublcp1	-1.07	1.07	1.29	1.16	1.26	1.2	1.38
ILMN_1372311	RGD1311005_predicted	1.3	1.41	1.18	1.1	1.15	1.22	1.38
ILMN_1357524	RGD1304719	1.11	-1.04	-1.28	-1.47	1.26	1.22	1.38
ILMN_1649757	Ppp2r5b	1.12	1.46	1.56	1.41	1.32	1.22	1.38
ILMN_1360625	Nrd1	-1.08	1.31	1.16	1.11	1.35	1.22	1.38
ILMN_1365373	LOC366784	1	1.17	1.27	1.26	1.25	1.23	1.38
ILMN_1363567	Dhrsx_predicted	1.19	1.44	1.35	1.31	1.17	1.24	1.38
ILMN_1366753	RGD1563048_predicted	1.08	1.19	1.18	1.22	1.37	1.25	1.38
ILMN_1351300	Srfbp1	1.07	1.57	1.5	1.86	1.37	1.25	1.38
ILMN_1359820	Ap3b1_predicted	-1.07	1.29	1.15	1.31	1.22	1.26	1.38
ILMN_1364489	LOC361157	-1.1	-1.01	1.08	1.19	1.11	1.28	1.38
ILMN_1350986	RGD1559475_predicted	1.03	1.02	-1.12	1.03	1.2	1.28	1.38
ILMN_1350199	RGD1559475_predicted	1.03	1.02	-1.12	1.03	1.2	1.28	1.38

ILMN_1376054	Slc39a8_predicted	1.11	-1.08	1.3	1.15	1.3	1.3	1.38
ILMN_1370543	RGD1309307_predicted	-1.13	1.16	1.11	1.06	1.13	1.33	1.38
ILMN_1530383	Tpst2	-1.03	1.27	1.24	1.28	1.65	1.33	1.38
ILMN_1362370	Tpst2	-1.03	1.27	1.24	1.28	1.65	1.33	1.38
ILMN_1358649	Zfyve9_predicted	1.41	1.52	-1.57	1.18	1.46	1.34	1.38
ILMN_1362318	LOC682762	-1.01	1.18	-1.01	1.21	1.26	1.35	1.38
ILMN_1369097	LOC303456	1.18	1.36	1.41	1.4	1.4	1.35	1.38
ILMN_1364272	RGD1561297_predicted	1.02	1.24	1.12	1.28	1.32	1.37	1.38
ILMN_1351703	Tnk2	1.21	2.5	2.24	2.65	1.38	1.37	1.38
ILMN_1352631	RGD1562140_predicted	1.04	1.13	1.3	1.33	1.37	1.44	1.38
ILMN_1356754	RGD1562140_predicted	1.04	1.13	1.3	1.33	1.37	1.44	1.38
ILMN_1361249	RGD1311824_predicted	-1.03	1.09	1.26	1.05	1.2	1.47	1.38
ILMN_1365097	LOC497818	1.06	-1.06	1.01	-1.02	1.2	1.5	1.38
ILMN_1363025	RGD1561817_predicted	-1.02	1.32	1.34	1.57	1.65	1.52	1.38
ILMN_1365390	Pigm	1.3	1.51	1.8	1.71	1.46	1.67	1.38
ILMN_1360014	Txnl1	1.27	1.06	1.2	1.25	1.56	1.73	1.38
ILMN_1357677	Fem1b_predicted	1.29	1.95	1.85	2.24	2.23	2.63	1.38
ILMN_1366631	Mvp	-1.02	-1.02	1.16	-1.04	1.14	-1.04	1.39
ILMN_1371074	Nme6	1.32	1.33	1.33	1.34	1.1	-1.03	1.39
ILMN_1650908	RGD1311086	1.2	1.89	1.56	1.73	-1.05	-1.02	1.39
ILMN_1365422	Alkbh2_predicted	-1.05	-1.08	1.05	-1.05	1.14	1.07	1.39
ILMN_1362871	LOC501506	-1.12	1.06	1.27	1.19	1.18	1.13	1.39
ILMN_1355110	Fbxl20	1.18	-1.24	1.04	-1.25	1.09	1.18	1.39
ILMN_1370521	Vasp_predicted	-1.05	1.26	1.02	1.34	1.26	1.19	1.39
ILMN_1364527	RGD1562407_predicted	1.37	1.11	1.09	1.05	1.32	1.21	1.39
ILMN_1367708	Hprt	-1.01	-1.01	-1.03	-1	1.19	1.22	1.39
ILMN_1361618	RGD1307778	-1.08	1.14	1.08	-1.05	1.32	1.23	1.39
ILMN_1369102	Prkacb_predicted	1.01	1.19	-1.09	-1.05	1.28	1.29	1.39
ILMN_1373944	RGD1559740_predicted	1.31	1.24	1.03	1.36	1.37	1.3	1.39
ILMN_1353777	Rae1	1.16	1.28	1.88	1.89	1.31	1.31	1.39
ILMN_1360885	Rps17	1.09	1.26	-1.07	1.27	1.3	1.33	1.39
ILMN_1367799	RGD1561264_predicted	1.02	1.45	1.08	1.35	1.38	1.34	1.39
ILMN_1374659	Rab18	-1.04	-1.08	1.04	-1.19	1.32	1.39	1.39
ILMN_1371927	Srp54	-1.07	1.16	1.22	1.28	1.42	1.39	1.39
ILMN_1354975	RGD1563311_predicted	-1.12	1.35	1.34	1.41	1.44	1.4	1.39
ILMN_1358580	LOC299740	1.2	1.44	1.05	1.41	1.45	1.42	1.39
ILMN_1358108	Srm	1.25	1.52	1.94	1.69	1.68	1.42	1.39
ILMN_1349268	Rhoa	1.25	-1.04	-3.14	-1.02	1.67	1.46	1.39

ILMN_1356458	Clic1	-1.01	-1.06	1.18	1.32	1.37	1.48	1.39
ILMN_1370582	LOC298977	1.21	1.39	1.36	1.33	1.48	1.48	1.39
ILMN_1373136	Tmem68_predicted	1.24	1.54	1.9	1.42	1.53	1.48	1.39
ILMN_1374856	RGD1305613	-1.07	1.21	1.56	1.18	1.39	1.49	1.39
ILMN_1353436	LOC497719	-1.02	1.33	1.7	1.55	1.56	1.5	1.39
ILMN_1363939	Ccnl1	-1.12	1.95	1.79	1.3	1.4	1.53	1.39
ILMN_1361127	Pdcd6_predicted	1.04	1.32	1.38	1.35	1.7	1.55	1.39
ILMN_1374078	Rab7	-1.04	-1.12	1.32	1.12	1.59	1.66	1.39
ILMN_1349615	Gbe1	1.15	1.32	1.37	1.66	1.59	1.71	1.39
ILMN_1369147	Tcea1	1.06	1.45	1.88	1.75	1.73	1.88	1.39
ILMN_1372932	LOC500504	1.02	1.43	1.51	1.38	1.45	2.22	1.39
ILMN_1357950	LOC362121	1.11	-1.05	2.21	1.27	1.24	-1.14	1.4
ILMN_1361387	Trim45_predicted	-1.02	-1.03	-1.03	1.05	-1.07	-1.12	1.4
ILMN_1350790	Tdrd7	1.01	1.21	1.02	1.02	1.14	1.02	1.4
ILMN_1357363	Nubpl_predicted	1.05	-1.02	-1.01	-1.35	1.16	1.02	1.4
ILMN_1369982	RGD1562835_predicted	1.21	1.37	-1.71	1.58	1.23	1.06	1.4
ILMN_1353732	Znf291	-1.02	1.21	1.26	1.05	1.23	1.08	1.4
ILMN_1353746	LOC679430	1.03	1.17	1.01	1.22	1.18	1.11	1.4
ILMN_1351195	RGD1565122_predicted	1.1	1.09	1.11	1.22	1.16	1.12	1.4
ILMN_1354299	Ktn1_predicted	1.03	1.57	1.31	1.32	1.24	1.19	1.4
ILMN_1366075	Ktn1_predicted	1.03	1.57	1.31	1.32	1.24	1.19	1.4
ILMN_1362179	Pygb	1.18	1.18	1.01	1.16	1.25	1.19	1.4
ILMN_1371849	Ccdc59_predicted	-1.09	-1.04	1.14	1.16	1.13	1.2	1.4
ILMN_1361715	RGD1311283_predicted	1.12	1.05	1.15	1.18	1.15	1.21	1.4
ILMN_1353224	Creb3	-1.03	1.13	1.27	1.34	1.41	1.24	1.4
ILMN_1372187	LOC499708	1.21	1.43	1.89	1.65	1.14	1.25	1.4
ILMN_1369400	Cul5	1.08	1.24	1.15	1.39	1.3	1.26	1.4
ILMN_1374824	LOC309309	1.25	1.34	1.53	1.61	1.15	1.28	1.4
ILMN_1357501	Pcm1	-1.08	1.13	1.09	1.14	1.17	1.3	1.4
ILMN_1363169	Atp6v0c	1.03	1.19	1.56	1.25	1.24	1.31	1.4
ILMN_1354493	Ninj1	1.07	1.35	1.44	1.38	1.46	1.33	1.4
ILMN_1367362	LOC361635	1.02	1.16	1.23	1.26	1.27	1.36	1.4
ILMN_1358239	Braf	1.17	1.48	1.62	1.43	1.73	1.41	1.4
ILMN_1370524	Dhx9_predicted	1.15	1.07	-1.78	1.24	1.39	1.43	1.4
ILMN_1365844	Nucb1	1.05	1.14	1.58	1.39	1.44	1.45	1.4
ILMN_1349854	Kpna1	1.1	1.55	1.96	1.66	1.74	1.79	1.4
ILMN_1372296	RGD1561833_predicted	-1	1.65	1.55	1.5	1.85	2.03	1.4
ILMN_1376840	Sh2d4a	1.14	-1.06	-1.01	-1.24	2.07	2.2	1.4

ILMN_1352309	Reep5_predicted	-1	-1.07	-1.32	-1.05	1.22	-1.1	1.41
ILMN_1357253	RGD1562987_predicted	1.12	1.3	1.41	1.52	1.2	-1	1.41
ILMN_1368234	Psap	1.16	1.03	1.07	-1.1	1.18	1.01	1.41
ILMN_1357541	Dnase1l1	-1.07	1.17	1.5	1.19	1.2	1.09	1.41
ILMN_1376331	Hadha	-1.18	-1.2	1.17	-1.02	1.23	1.09	1.41
ILMN_1362725	Exosc9	-1.05	1.51	1.24	1.47	1.2	1.1	1.41
ILMN_1350154	Them2_predicted	1.22	-1.09	-1.2	-1.13	1.13	1.11	1.41
ILMN_1352255	RGD1308210	1.14	1.33	1.6	1.41	1.1	1.12	1.41
ILMN_1373198	Ppp1r15b_predicted	-1.04	1.1	-1.05	1.21	1.22	1.12	1.41
ILMN_1368182	Sdad1	1.04	1.78	1.77	1.9	-1.11	1.15	1.41
ILMN_1354272	LOC310926	1.29	1.16	-2.9	1.2	1.33	1.18	1.41
ILMN_1371384	RGD1563195_predicted	1.11	1.79	1.82	1.8	1.31	1.21	1.41
ILMN_1349858	RGD1562884_predicted	1.09	1.44	1.44	1.41	1.12	1.24	1.41
ILMN_1370118	Rpl7	1.17	1.19	1.35	1.27	1.17	1.24	1.41
ILMN_1351217	Stard6	1.06	1.44	1.35	1.45	1.37	1.25	1.41
ILMN_1360823	Hsd17b4	1.12	1.16	1.16	-1.11	1.13	1.27	1.41
ILMN_1353991	Unc93b1	1.01	1.55	1.2	1.37	1.36	1.29	1.41
ILMN_1359825	LOC688018	1.12	1.26	-1.15	1.37	1.61	1.29	1.41
ILMN_1365515	Ehd4	-1.08	1.16	1.48	1.33	1.46	1.31	1.41
ILMN_1376829	Raly	-1.03	-1.17	1.82	1.33	1.42	1.34	1.41
ILMN_1367460	RGD1565744_predicted	1.04	1.26	1.17	1.26	1.48	1.36	1.41
ILMN_1369573	LOC688712	1.02	1.76	1.28	1.43	1.45	1.38	1.41
ILMN_1374370	Ke2	1.4	1.37	1.41	1.55	1.37	1.39	1.41
ILMN_1351080	RGD1559963_predicted	1.19	1.47	1.16	1.33	1.5	1.42	1.41
ILMN_1356007	Tubgcp5_predicted	1.2	1.38	1.63	1.44	1.58	1.44	1.41
ILMN_1368621	Rassf5	1.48	2.01	1.8	2.31	1.24	1.45	1.41
ILMN_1376379	Gkap1	1.08	1.52	1.4	1.49	1.41	1.45	1.41
ILMN_1358732	Rxra	1.19	1.78	1.09	1.5	1.35	1.47	1.41
ILMN_1376784	Phf7	1.13	1.18	1.26	1.03	1.32	1.48	1.41
ILMN_1355719	Nyw1	1.12	1.22	-1.03	1.23	1.44	1.48	1.41
ILMN_1351996	Hadhb	-1.07	1.24	1.71	1.32	1.67	1.48	1.41
ILMN_1361301	Slc12a4	1.22	1.38	1.4	1.58	1.42	1.5	1.41
ILMN_1360478	R3hdm1	1.04	1.34	1.23	1.37	1.55	1.5	1.41
ILMN_1367730	Mtdh	1.27	1.82	1.87	1.78	1.81	1.53	1.41
ILMN_1361236	Trmt1	1.12	1.92	1.69	1.83	1.18	1.56	1.41
ILMN_1356094	Chmp5	1.06	1.16	1.19	1.02	1.55	1.56	1.41
ILMN_1370927	MGC72957	1.13	1.39	1.33	1.43	1.46	1.58	1.41
ILMN_1370936	Slc31a1	1.1	1.32	1.36	1.24	1.87	1.59	1.41

ILMN_1366528	Jmjd2c_predicted	1.07	1.36	1.23	1.57	1.57	1.61	1.41
ILMN_1375203	Ptp4a1	1.15	1.11	1.32	1.33	1.55	1.66	1.41
ILMN_1355879	RGD1310128_predicted	-1.02	1.07	1.09	1.4	1.63	1.68	1.41
ILMN_1351408	Slc7a1	1.33	1.34	1.09	1.29	1.77	1.68	1.41
ILMN_1373509	Sec61b_predicted	-1.05	1.1	-1.16	1.12	2.2	1.82	1.41
ILMN_1362346	Otud5	1.02	1.34	1.16	1.2	1.57	1.92	1.41
ILMN_1376646	Bag3	-1.1	1.09	1.02	-1.1	1.96	1.93	1.41
ILMN_1650170	RGD1311037	1.33	1.69	2.12	1.54	1.99	2	1.41
ILMN_1350852	LOC246120	-1.03	-1.06	1.23	-1.27	-1.3	-1.3	1.42
ILMN_1358377	Mthfs	-1.04	-1.16	-1.12	-1.59	-1.19	-1.1	1.42
ILMN_1374322	RGD1561381_predicted	-1.06	1	-1.45	-1.18	1.16	-1.09	1.42
ILMN_1353778	LOC498914	1.02	1.03	-1.22	1.06	1.21	1.03	1.42
ILMN_1349826	Ccs	-1.14	1.07	1.07	-1.08	1.11	1.12	1.42
ILMN_1376719	Cd81	-1.12	-1.31	-1.08	-1.13	1.03	1.14	1.42
ILMN_1355149	Manba	1.06	1.21	1.27	1.22	1.24	1.15	1.42
ILMN_1360281	RGD1308923_predicted	1.09	1.27	1.53	1.14	1.21	1.17	1.42
ILMN_1361779	Tcea3	1.12	2.39	1.8	1.46	1.16	1.19	1.42
ILMN_1361950	Sdc2	1.09	1.51	1.47	1.27	1.45	1.28	1.42
ILMN_1365054	LOC289401	1.02	1.45	-1.15	1.49	1.3	1.29	1.42
ILMN_1359325	Mta1	1.22	1.96	1.89	1.66	1.2	1.31	1.42
ILMN_1351142	LOC502302	1.06	1.32	-1.13	1.28	1.35	1.39	1.42
ILMN_1372060	RGD1305605_predicted	-1.02	1.41	1.1	1.47	1.33	1.43	1.42
ILMN_1372542	Ppp2cb	1.06	-1.19	-1.03	-1.13	1.45	1.44	1.42
ILMN_1365490	RGD1565809_predicted	1.05	1.34	1.13	1.43	1.46	1.46	1.42
ILMN_1349413	Igf2r	-1.04	1.4	1.38	1.38	1.53	1.48	1.42
ILMN_1360571	Ap3d1	-1.05	1.21	1.1	1.09	1.5	1.49	1.42
ILMN_1351089	Cpt1a	1.22	1.22	-1.02	-1.09	1.33	1.53	1.42
ILMN_1354463	LOC498192	1.12	1.25	1.38	1.44	1.75	1.55	1.42
ILMN_1348811	RGD1308813	1.24	1.65	2.25	1.68	1.86	1.58	1.42
ILMN_1368291	RGD1565286_predicted	-1.05	1.5	1.32	1.75	1.52	1.62	1.42
ILMN_1374305	Cltc	1.03	1.53	1.33	1.63	1.62	1.62	1.42
ILMN_1376796	Rnf166	1.05	1.2	1.45	1.53	1.44	1.63	1.42
ILMN_1368948	Abcb1	1.09	-1.16	-1.17	-1.16	1.58	1.66	1.42
ILMN_1370974	Dnajb6	1.13	1.34	1.42	1.2	1.65	1.76	1.42
ILMN_1376551	Zfp143	-1.12	1.19	1.18	1.42	1.89	1.84	1.42
ILMN_1354525	RGD1310875_predicted	1.14	1.62	1.96	1.97	2.02	1.91	1.42
ILMN_1356063	Tlk2_predicted	1.07	1.85	1.68	1.89	2.08	1.93	1.42
ILMN_1376312	Mar-07	1.27	1.52	1.92	1.02	1.93	1.70	1.42

ILMN_1374895	Blvrb_predicted	1.13	1.09	-1	1.15	-1.11	-1.35	1.43
ILMN_1367162	Gpm6a	-1.03	-1.04	1.1	-1.07	1.23	-1.08	1.43
ILMN_1370698	RGD1563584_predicted	-1.11	-1.08	-1.3	1.32	1.11	-1.04	1.43
ILMN_1356509	Anp32a	1.13	-1.05	1.18	1.27	1.07	-1.03	1.43
ILMN_1350032	Hmgn1	1.02	-1.82	-1.23	-1.28	-1.07	1.01	1.43
ILMN_1364936	Syap1	-1.05	-1.07	1.03	1.01	1.09	1.12	1.43
ILMN_1356778	RGD1310427_predicted	-1.05	1.35	1.48	1.37	1.19	1.15	1.43
ILMN_1373719	Zdhhc7	1.18	1.33	1.38	1.68	1.28	1.17	1.43
ILMN_1353482	RGD1565975_predicted	1.14	1.38	1.19	1.24	-1.02	1.18	1.43
ILMN_1358257	Pold4	-1.19	-1.05	-1.29	-1.65	1.27	1.18	1.43
ILMN_1650963	LOC685888	1.13	1.18	1.03	1.21	1.43	1.18	1.43
ILMN_1374045	Gltscr2	-1.03	1.34	1.34	1.21	1.18	1.2	1.43
ILMN_1357766	Klhl9_predicted	-1.04	-1	1.1	1.01	1.28	1.2	1.43
ILMN_1368639	Dhx29_predicted	1.15	1.06	1.14	1.14	1.28	1.2	1.43
ILMN_1349689	Psma1	-1.13	-1.14	-1.33	1.19	-1.01	1.24	1.43
ILMN_1371829	Slc37a3_predicted	1.22	1	1.56	1.05	1.08	1.26	1.43
ILMN_1354658	Ribc1	1.21	1.43	1.54	-1.18	1.41	1.27	1.43
ILMN_1356998	Krtcap2_predicted	1.11	1.47	1.08	1.42	1.51	1.29	1.43
ILMN_1366807	Hnrpk	1.01	1.08	1.16	1.03	1.4	1.31	1.43
ILMN_1353983	Psmd4	1.07	1.31	1.32	1.26	1.38	1.34	1.43
ILMN_1371170	RGD1305457	1.07	1.39	1.28	1.48	1.38	1.34	1.43
ILMN_1369389	Rpp38	1.07	1.61	1.34	1.69	1.4	1.34	1.43
ILMN_1368953	Sec3l1_predicted	1.1	1.05	1.17	1.29	1.4	1.35	1.43
ILMN_2040891	Psd	1.1	1.39	1.32	1.37	1.24	1.36	1.43
ILMN_1350600	LOC301124	1.01	1.09	-1.04	1.18	1.55	1.46	1.43
ILMN_1373618	Ccnh	1.13	1.5	1.46	1.47	1.5	1.47	1.43
ILMN_1352194	Ubxd4_predicted	1.21	1.47	1.3	1.62	1.39	1.49	1.43
ILMN_1373191	LOC499794	1.16	1.81	1.5	1.6	1.28	1.53	1.43
ILMN_1348951	Zfp622	1.07	1.2	1.26	1.38	1.61	1.64	1.43
ILMN_1360891	Spin	1.05	1.64	1.31	1.69	1.28	1.76	1.43
ILMN_1374986	Cldn2_predicted	-1.07	1.3	1.25	1.17	1.15	1.02	1.44
ILMN_1367541	S100a13_predicted	1.12	1.41	-1.39	1.25	1.22	1.04	1.44
ILMN_1373574	Hdac3	-1.08	-1.02	-1.02	1.04	1.07	1.07	1.44
ILMN_1359425	RGD1305975_predicted	1.06	1.18	-1.04	1.35	1.21	1.09	1.44
ILMN_1364273	Aadacl1_predicted	1.11	1.14	1.48	1.26	1.24	1.16	1.44
ILMN_1364845	RGD1305147_predicted	1.18	1.34	1.15	1.26	1.25	1.23	1.44
ILMN_1364146	RGD1305147_predicted	1.18	1.34	1.15	1.26	1.25	1.23	1.44
ILMN_1373210	Entpd5	1.5	1.05	-1.24	-1.12	1.06	1.25	1.44

ILMN_1360491	RGD1311752_predicted	1.26	1.97	1.89	2.25	1.26	1.26	1.44
ILMN_1372547	Fbxo4_predicted	1.08	1.15	1.06	1.16	1.47	1.31	1.44
ILMN_1364021	Grpel1	-1.12	1.37	1.48	1.35	1.57	1.35	1.44
ILMN_1376241	Rab34	-1.04	1.59	1.38	1.54	1.51	1.36	1.44
ILMN_1375166	Kctd9_predicted	1.15	-1.13	-1.06	-1.01	1.13	1.42	1.44
ILMN_1368274	RGD1309784	1.14	1.27	1.61	1.4	1.47	1.42	1.44
ILMN_1650862	LOC497716	1.11	1.34	1.46	1.36	1.36	1.45	1.44
ILMN_1371270	Gpr56	1.46	1.72	1.32	1.71	1.76	1.49	1.44
ILMN_1351165	Cln8	1.22	1.07	1.09	1.05	1.29	1.51	1.44
ILMN_1355576	RGD1561768_predicted	1	1.57	1.4	1.48	1.37	1.51	1.44
ILMN_1355249	Slc30a5_predicted	1.14	1.39	1.35	1.48	1.68	1.52	1.44
ILMN_1358918	Serinc1	1.07	-1.09	-1.02	-1.18	1.57	1.55	1.44
ILMN_1349720	Arfgef1_predicted	1.28	1.72	1.35	1.68	1.65	1.55	1.44
ILMN_1356542	LOC498176	1.26	1.85	1.68	2.29	1.58	1.57	1.44
ILMN_1364910	RGD1561967_predicted	1.03	1.28	1.4	1.49	1.88	1.57	1.44
ILMN_1357626	RGD1559909_predicted	1.13	1.06	1.08	1.22	1.74	1.61	1.44
ILMN_1362402	Alkbh_predicted	1.11	1.3	1.62	1.58	1.53	1.63	1.44
ILMN_1372654	Map1lc3b	1.06	-1.02	1.3	-1.29	1.68	1.73	1.44
ILMN_1358079	Rab21	-1.02	1.43	1.56	1.59	1.68	1.75	1.44
ILMN_1352448	RGD1565054_predicted	1.17	1.46	1.06	1.43	1.62	1.8	1.44
ILMN_1362767	Clu	-1.08	-1.17	-1.28	-1.45	-1.05	-1.16	1.45
ILMN_1365417	Rala	1.05	1.12	-1.14	1.25	1.18	1.09	1.45
ILMN_1372230	Rnf149	1.11	1.16	1.43	1.29	1.08	1.11	1.45
ILMN_1371378	RGD1311849_predicted	1.15	1.38	1.09	1.17	1.18	1.13	1.45
ILMN_1352307	Pepd_mapped	1.09	1.06	1.31	1.12	1.27	1.13	1.45
ILMN_1351567	LOC360728	1.29	1.13	1.1	1.3	1.48	1.15	1.45
ILMN_1370444	RGD708545	1.07	1.64	1.51	1.48	1.38	1.16	1.45
ILMN_1354684	Tmem33	1.15	1.19	1.1	1.46	1.56	1.16	1.45
ILMN_1650080	LOC360910	1.19	1.15	-1.06	1.4	1.64	1.18	1.45
ILMN_1370808	RGD1307235_predicted	-1.1	1.03	1.11	-1.07	1.35	1.19	1.45
ILMN_1355235	Fads1	1.02	-1.18	1.2	-1.07	1.29	1.23	1.45
ILMN_1358902	Eps15	1.07	1.47	1.24	1.34	1.2	1.24	1.45
ILMN_1368402	Uap1_predicted	-1.17	1.03	1.03	1.05	1.48	1.27	1.45
ILMN_1361617	Eftud1_predicted	1.23	1.21	1.13	1.28	1.52	1.29	1.45
ILMN_1351737	Kctd13	1.41	1.75	2.54	2.49	1.49	1.34	1.45
ILMN_1359184	Stxbp1	1.12	1.27	1.1	1.25	1.27	1.35	1.45
ILMN_1367784	RGD1562073_predicted	1.17	1.3	-1.12	1.44	1.41	1.35	1.45
ILMN_1351535	LOC310360	-1.1	1.27	1.51	1.53	1.38	1.36	1.45

ILMN_1355990	Ube2m_predicted	1.09	1.17	1.3	1.33	1.36	1.41	1.45
ILMN_1372861	RGD1305755	1.14	1.28	1.29	1.32	1.52	1.41	1.45
ILMN_1365061	Ywhag	1.01	1.17	1.19	1.28	1.44	1.43	1.45
ILMN_1366226	RGD1359713	1.01	1.29	-1.06	1.32	1.62	1.43	1.45
ILMN_1353280	LOC363861	1.34	1.26	-1.07	1.52	1.43	1.44	1.45
ILMN_1354859	MGC105508	-1.06	1.29	1.23	1.4	1.56	1.5	1.45
ILMN_1359345	RGD1307915_predicted	1.09	1.18	1.35	1.12	1.49	1.51	1.45
ILMN_1349174	RGD1559923_predicted	1.07	1.87	1.76	1.86	1.57	1.61	1.45
ILMN_1356048	Snapc3	1.12	1.76	1.73	1.68	1.49	1.62	1.45
ILMN_1360573	RGD1563861_predicted	1.02	1.63	1.39	1.75	1.44	1.64	1.45
ILMN_1365384	RGD1563861_predicted	1.02	1.63	1.39	1.75	1.44	1.64	1.45
ILMN_1352550	Ppm1a	1.37	1.65	1.39	1.38	1.75	1.64	1.45
ILMN_1368688	Atg12	1.12	1.25	1.21	1.16	1.85	1.87	1.45
ILMN_1351318	Arl4a	1.11	2.4	1.63	1.54	1.85	2.02	1.45
ILMN_1348799	RGD1306192_predicted	1	-1.03	1.05	-1.07	1.02	-1.03	1.46
ILMN_1370782	Dnase2	-1.02	1.12	1.13	1.05	1.09	1.07	1.46
ILMN_1375330	Pex16_predicted	1.06	1.1	1.55	1.14	1.24	1.12	1.46
ILMN_1370814	Fkbpl	1.12	1.27	1.1	1.46	1.25	1.12	1.46
ILMN_1360596	Ssb	-1.12	1.01	1.16	1.3	1.06	1.15	1.46
ILMN_1366813	Ppt2	1.01	1.27	1.52	1.46	1.25	1.2	1.46
ILMN_1363415	Pcbp4_predicted	1.11	1.45	1.1	1.47	1.35	1.2	1.46
ILMN_1376793	Anxa11	1.03	-1.02	1.07	-1.02	1.25	1.24	1.46
ILMN_1365396	Anpep	-1.11	1.08	-1.02	1.22	1.17	1.26	1.46
ILMN_1651136	Chchd6_predicted	-1.03	1.16	1.52	1.47	1.35	1.27	1.46
ILMN_1366627	LOC501156	1.05	1.05	1.07	1.02	1.29	1.28	1.46
ILMN_1363352	Tbc1d20	-1.1	1.05	1.1	1.26	1.36	1.29	1.46
ILMN_1354938	RGD1561942_predicted	1.17	1.71	1.48	1.63	1.58	1.33	1.46
ILMN_1365924	RGD1310905_predicted	1.01	1.11	1.03	1.17	1.28	1.38	1.46
ILMN_1355226	LOC500380	-1.09	-1.17	-1.18	1.03	1.23	1.39	1.46
ILMN_1356726	Tmem34	1.12	1.18	1.22	-1.07	1.5	1.4	1.46
ILMN_1349358	RGD1560069_predicted	1.17	1.39	1.05	1.36	1.41	1.41	1.46
ILMN_2040496	RGD1565602_predicted	1.03	1.29	1.23	1.11	1.42	1.42	1.46
ILMN_1369180	Rab2	1.16	1.19	1.15	1.23	1.46	1.56	1.46
ILMN_1376437	Zfand3	1.09	1.42	1.03	1.53	1.68	1.57	1.46
ILMN_1362582	RGD1564290_predicted	1.18	1.34	-1.02	1.34	1.79	1.58	1.46
ILMN_1348826	LOC367857	1.11	1.18	1.4	1.28	1.46	1.61	1.46
ILMN_1355725	Clta	1.09	1.56	1.28	1.51	1.54	1.65	1.46
ILMN_1365095	Dhx40	1.22	1.2	-1	-1.18	1.59	1.73	1.46

ILMN_1358788	Hspa9a_predicted	1.16	1.61	1.92	1.63	1.88	1.82	1.46
ILMN_1353117	Pecr	-1.15	1.64	1.38	1.31	1.03	-1.28	1.47
ILMN_1650290	Hist2h3c2_predicted	1.03	-1.25	-1.16	-1.04	-1.11	1.08	1.47
ILMN_1351340	LOC500950	1.11	1.11	1.12	1.13	1.45	1.1	1.47
ILMN_1374435	C1galt1c1	-1.11	-1.14	-1.01	-1.22	1.31	1.13	1.47
ILMN_1364391	RGD1311091_predicted	1.02	1.6	1.55	2.35	1.14	1.16	1.47
ILMN_1650114	Cdc37l1	1.09	1.23	1.16	1.36	1.3	1.19	1.47
ILMN_1375135	Ctps_predicted	1.38	1.9	1.79	2.14	1.21	1.24	1.47
ILMN_1376674	Lap3	-1.19	-1.21	1.02	1.06	1.31	1.27	1.47
ILMN_1355612	Usp45_predicted	1.29	1.24	1.14	1.4	1.22	1.32	1.47
ILMN_1353807	RGD1563531_predicted	1.22	1.7	1.68	1.92	1.29	1.35	1.47
ILMN_1353772	LOC365800	1.05	1.16	-1.03	1.39	1.28	1.42	1.47
ILMN_1359576	Tmem63b_predicted	1.32	1.66	1.59	1.7	1.52	1.42	1.47
ILMN_1353829	Mnab_predicted	1.21	1.4	1.05	1.35	1.54	1.42	1.47
ILMN_1370684	Mnt_predicted	1.38	1.26	1.01	-1.02	1.19	1.43	1.47
ILMN_1357698	RGD1305500_predicted	1.11	1.71	1.73	1.52	1.48	1.43	1.47
ILMN_1371316	Tyki_predicted	1.1	1.31	1.7	1.46	1.23	1.44	1.47
ILMN_1374244	Coro1b	1.04	1.39	1.53	1.31	1.38	1.46	1.47
ILMN_1359658	Gla_mapped	1.01	1	1.25	1.05	1.53	1.46	1.47
ILMN_1357564	Ythdf3_predicted	-1.09	1.17	1.29	1.19	1.7	1.46	1.47
ILMN_1355401	RGD1563543_predicted	1.04	1.42	1.06	1.54	1.43	1.49	1.47
ILMN_1372322	Scmh1_predicted	-1.03	1.33	1.25	1.45	1.17	1.59	1.47
ILMN_1350468	Map2k1ip1	1	1.41	1.72	1.35	1.8	1.63	1.47
ILMN_1361290	RGD1359310	1.14	1.36	1.45	1.36	1.72	1.83	1.47
ILMN_1374102	Snx2_predicted	1.09	1.74	1.75	1.58	1.93	1.98	1.47
ILMN_1360978	Ratsg2	-1.21	1.59	1.57	1.61	2.54	2.06	1.47
ILMN_1350438	Nfkbib	-1.03	1.52	1.36	1.38	2.42	2.21	1.47
ILMN_1369337	Lysmd3	-1.18	1.21	1.04	1.01	2.65	2.42	1.47
ILMN_1364519	Sdccag1	-1.15	1.11	-1.32	1.05	1.15	1.17	1.48
ILMN_1349012	Parg	1.07	1.12	1.1	1.14	1.23	1.18	1.48
ILMN_1649875	RGD1307129	1.11	1.09	1.06	1.04	1.22	1.21	1.48
ILMN_1351172	RGD1559149_predicted	1.09	1.21	1.08	1.38	1.49	1.24	1.48
ILMN_1359932	LOC497701	1.09	1.13	1.16	1.06	1.16	1.25	1.48
ILMN_1362982	C1galt1	1.06	1.5	1.23	1.35	1.15	1.34	1.48
ILMN_1369696	LOC499853	1.07	1.28	1.31	1.49	1.55	1.34	1.48
ILMN_1361096	RGD1566229_predicted	-1.14	1.22	1.17	1.34	1.23	1.4	1.48
ILMN_1352722	LOC316550	1.04	-1.19	-1.01	-1.07	1.47	1.4	1.48
ILMN_1365758	Atp5s	-1.02	1.01	1.13	1.1	1.44	1.41	1.48

ILMN_1359928	RGD1561198_predicted	1.12	1.33	1.21	1.74	1.45	1.42	1.48
ILMN_1355933	RGD1311444_predicted	-1.13	1.03	1.08	-1.08	1.6	1.43	1.48
ILMN_1362057	Spire1_predicted	1.09	1.5	1.45	1.66	1.38	1.44	1.48
ILMN_1352654	RGD1309602_predicted	1.11	1.78	2.14	1.6	1.3	1.45	1.48
ILMN_1376936	Tdrd3	1.07	1.9	1.66	1.79	1.35	1.46	1.48
ILMN_1352331	Tex264	1.18	1.5	1.64	1.34	1.45	1.46	1.48
ILMN_1375676	Neurl2_predicted	1.17	1	1.35	-1	1.18	1.47	1.48
ILMN_1363374	RGD1566189_predicted	1.3	1.69	2.11	1.41	1.36	1.48	1.48
ILMN_1370963	Anxa5	1.04	1.13	1.44	1.19	1.4	1.5	1.48
ILMN_2039248	Rars_predicted	1.14	1.21	1.17	1.31	1.56	1.5	1.48
ILMN_1354929	RGD1306697_predicted	1.21	2.16	1.6	1.85	1.38	1.54	1.48
ILMN_1359251	Sec23ip	1.02	1.4	1.11	1.08	1.59	1.56	1.48
ILMN_1368523	LOC502636	-1.05	1.2	1.27	1.44	1.54	1.59	1.48
ILMN_1374120	RGD1566317_predicted	1.02	1.72	-1.13	1.3	1.65	1.59	1.48
ILMN_1370334	Kctd6_predicted	1.28	1.92	1.62	2.23	1.61	1.69	1.48
ILMN_1372574	RGD1564163_predicted	-1.01	-1.03	1.09	1.21	2.14	1.84	1.48
ILMN_1376727	Gsto2	1.03	1.2	1.77	1.34	3.49	3.72	1.48
ILMN_1363156	LOC684318	1.02	1.13	1.15	1.26	1.14	1.17	1.49
ILMN_1355282	Fuca	-1.03	1.12	1.3	1.06	1.16	1.17	1.49
ILMN_1349757	Sirt5	-1.02	1.89	1.61	1.49	1.31	1.18	1.49
ILMN_1351569	Atp1a1	1.12	1.58	1.8	1.83	1.16	1.19	1.49
ILMN_1356553	Ormdl2_predicted	-1.03	-1.07	-1.02	1.03	1.35	1.21	1.49
ILMN_1363605	LOC503176	1.14	1.33	1.27	1.48	1.12	1.27	1.49
ILMN_1371440	Asmtl_predicted	-1.07	1	-1.11	-1.01	1.46	1.29	1.49
ILMN_1365109	LOC500343	1.04	1.18	1.03	1.07	1.24	1.3	1.49
ILMN_1376473	Tesk1	-1.05	1.59	1.79	1.75	1.22	1.33	1.49
ILMN_1357483	Creb3l1	1.07	1.41	1.74	1.89	1.44	1.37	1.49
ILMN_1651060	RGD1565900_predicted	1.32	1.63	1.15	1.79	1.29	1.39	1.49
ILMN_1353028	LOC497732	1.24	-1.12	1.01	1.2	1.27	1.42	1.49
ILMN_1362269	Actg_predicted	1.09	2.63	2.52	1.93	1.29	1.44	1.49
ILMN_1353162	Tmem50a_predicted	1.07	1.33	1.24	1.25	1.47	1.45	1.49
ILMN_1365309	RGD1560936_predicted	-1	-1.02	1.09	1.37	1.29	1.51	1.49
ILMN_1361370	Rybp_predicted	1.3	1.59	1.37	1.53	1.49	1.55	1.49
ILMN_1374817	Rp1h	1.01	-1.03	-1.14	-1.06	1.67	1.55	1.49
ILMN_1651184	Hp	1.04	1.26	1.59	1.22	1.45	1.66	1.49
ILMN_1351619	Tmtc3_predicted	1.34	1.05	-1.32	1.11	1.13	1.07	1.5
ILMN_1355570	Pscd3	1.25	1.02	1.25	1.02	1.25	1.09	1.5
ILMN_1360473	Jund	1.01	1.17	1.93	-1.09	1.15	1.11	1.5

ILMN_1365800	Rac2	1.05	1.11	1.1	1.12	1.26	1.16	1.5
ILMN_1359210	Vezt	1.26	1.1	1.22	1.5	1.44	1.31	1.5
ILMN_1360321	Cd276	1.03	1.15	1.03	1.04	1.49	1.34	1.5
ILMN_1363239	RGD1305072	1.15	1.21	1.6	1.34	1.49	1.36	1.5
ILMN_1650870	Ua20	-1.18	-1.04	-1.18	1.05	1.22	1.38	1.5
ILMN_1368007	Tcf20_mapped	1.11	1.51	1.41	1.54	1.43	1.38	1.5
ILMN_1367213	Cgrrf1	1.14	1.12	1.11	1.03	1.19	1.39	1.5
ILMN_1364547	LOC299907	1.03	-1.2	-1.02	1.11	1.66	1.41	1.5
ILMN_1357991	LOC500124	1.01	1.76	1.25	1.56	1.48	1.44	1.5
ILMN_1352231	LOC686087	1.14	-1.07	1.27	-1.12	1.34	1.45	1.5
ILMN_1351690	LOC500687	-1.06	1.06	-1.07	-1.02	1.42	1.46	1.5
ILMN_1363695	Gosr2	1.01	1.2	1.47	-1.05	1.8	1.53	1.5
ILMN_1376554	Mat1a	1.13	1.31	1.7	1.31	1.97	1.59	1.5
ILMN_1352794	RGD1566264_predicted	-1.03	1.55	-1.01	1.56	1.55	1.61	1.5
ILMN_1365883	Ibrdc1_predicted	1.27	1.73	1.51	1.72	1.5	1.63	1.5
ILMN_1366950	Vps29_predicted	1.06	1.16	1.33	1.46	1.67	1.92	1.5
ILMN_1367306	RGD1311920_predicted	1.14	1.4	1.3	1.32	1.97	1.99	1.5
ILMN_1370817	Stk17b	1.24	1.35	1.46	1.34	1.93	2.05	1.5
ILMN_1370430	LOC302328	1.16	1.18	-1.29	-1.02	1.24	-1.1	1.51
ILMN_1370882	LOC362129	1.07	-1.06	1.11	-1.13	1.14	-1.04	1.51
ILMN_1354146	RGD1564337_predicted	1.24	1.25	1.12	1.25	1.04	1.14	1.51
ILMN_1363917	Pbef1	1.18	1.17	1.09	1.16	1.42	1.23	1.51
ILMN_1349295	Lgtn_predicted	-1.01	1.14	1.08	1.06	1.36	1.3	1.51
ILMN_1371479	Pnkp	-1.1	1.43	1.71	1.67	1.32	1.32	1.51
ILMN_1355968	Bxdc1_predicted	1.01	1.95	2.03	2.05	1.42	1.36	1.51
ILMN_1349811	Rab11a	-1.22	-1.31	-1.07	-1.14	1.69	1.38	1.51
ILMN_1362068	RGD1564227_predicted	1.07	1.38	1.2	1.37	1.51	1.42	1.51
ILMN_1350462	Slc41a2_predicted	1.21	1.49	1.39	2.1	1.66	1.42	1.51
ILMN_1367105	Cbr1	1.4	1.12	-1	1.31	1.47	1.46	1.51
ILMN_1349761	Cirh1a	1.32	2.36	2.13	2.57	1.61	1.51	1.51
ILMN_1349276	RGD1564677_predicted	1.31	2.25	2.56	2.41	1.62	1.52	1.51
ILMN_1372910	Gmppa	-1.05	1.25	1.26	1.01	1.55	1.54	1.51
ILMN_1369998	Rps23	1.15	1.43	1.15	1.4	1.47	1.58	1.51
ILMN_1351306	RGD1561789_predicted	1.05	1.58	-1.09	1.42	1.63	1.58	1.51
ILMN_1352342	Eif5	1.21	1.52	1.44	1.52	1.62	1.7	1.51
ILMN_1651172	Wdr22	1.08	2.13	1.62	2.03	1.86	1.7	1.51
ILMN_1368191	RGD1309708	1.13	1.26	1.18	1.29	1.57	1.73	1.51
ILMN_1352959	RGD1307475	1.08	1.39	1.46	1.18	1.63	1.75	1.51

ILMN_1351154	RGD1560076_predicted	1.01	1.28	1.26	1.61	1.3	1.82	1.51
ILMN_1358841	LOC302913	1.02	1.62	1.43	1.24	2.04	1.89	1.51
ILMN_1364929	Bambi	1.44	2.07	2.32	2.61	2.08	2.03	1.51
ILMN_1371021	RGD1303142	-1.01	1.2	1.12	1.19	2.08	2.49	1.51
ILMN_1351240	Hspa5	-1.13	1.29	1.73	1.3	2.96	2.61	1.51
ILMN_1374412	RGD1563547_predicted	1.02	1.27	-2.01	1.24	1.22	-1.16	1.52
ILMN_1357729	Abcd3	1.1	1.01	-1.15	-1.16	1.17	1.07	1.52
ILMN_1650838	Usp33	1.01	1.12	1.27	1.29	1.26	1.1	1.52
ILMN_1376945	RGD1309313	1.14	1.33	1.14	1.2	1.45	1.14	1.52
ILMN_1351083	Tmem14a_predicted	1.05	-1.09	1.29	-1.21	1.27	1.16	1.52
ILMN_1371362	RGD1563438_predicted	1.23	1.14	-1.53	1.06	1.5	1.17	1.52
ILMN_1351282	Hmgcr	1.23	-1.49	-1.07	-1.25	1.22	1.29	1.52
ILMN_1376433	Pex16	-1.02	1.15	1.19	1.29	1.25	1.29	1.52
ILMN_1350444	Psmb9	1.13	-1.02	-1.02	1.27	1.02	1.3	1.52
ILMN_1361028	RGD1562489_predicted	1.06	1.63	1.12	1.56	1.3	1.32	1.52
ILMN_1372463	Nagk_predicted	-1.07	1.18	1.31	1.3	1.27	1.35	1.52
ILMN_1362324	LOC501503	1.08	-1	-1	1.14	1.31	1.35	1.52
ILMN_1650350	Tmem9b_predicted	-1.05	-1.07	1	-1.05	1.41	1.36	1.52
ILMN_1359446	RGD1562091_predicted	1.37	1.75	1.34	1.44	1.4	1.41	1.52
ILMN_1359254	RGD1564586_predicted	-1.09	1.2	-1.09	1.32	1.41	1.42	1.52
ILMN_1371338	Ttc1	1.02	1.08	1.16	-1.03	1.35	1.43	1.52
ILMN_1362445	Tomm70a	1.12	1.44	1.51	1.61	1.48	1.49	1.52
ILMN_1364005	Rnd1	1.14	1.26	1.24	1.23	1.6	1.49	1.52
ILMN_1367875	Chordc1_predicted	-1.07	1.38	1.35	1.77	1.46	1.51	1.52
ILMN_1373552	LOC500909	1.07	1.18	1.43	1.29	1.82	1.55	1.52
ILMN_1350785	Rbm24_predicted	-1	1.2	1.19	1.21	1.53	1.62	1.52
ILMN_2039333	Dgka	-1.03	1.82	2.3	1.74	1.77	1.64	1.52
ILMN_1351387	RGD1565798_predicted	-1.03	1.29	1.26	1.29	1.67	1.72	1.52
ILMN_1350067	RGD1565798_predicted	-1.03	1.29	1.26	1.29	1.67	1.72	1.52
ILMN_1361429	Arih1	1.22	2.52	2.01	2.56	1.58	1.75	1.52
ILMN_1375100	Atf4	1.09	1.66	1.7	1.49	1.85	1.76	1.52
ILMN_1363481	Ube1dc1	-1	1.45	1.71	1.39	1.73	1.92	1.52
ILMN_1375568	Txndc7	-1.05	-1.3	-1	-1.05	2.89	2.61	1.52
ILMN_1365681	Snapap	-1.07	1.13	1.36	1.01	1.23	-1.03	1.53
ILMN_1356091	Ebna1bp2	-1.05	1.04	1.11	1.22	1.08	1.07	1.53
ILMN_1357552	Apip_predicted	1	1.01	1.04	-1.1	1.26	1.2	1.53
ILMN_1370437	Gpnmb	-1.09	1.31	1.51	1.6	1.52	1.29	1.53
ILMN_1372201	LOC313245	1.15	1.11	1.1	1.67	1.12	1.3	1.53

ILMN_1363332	LOC499675	1.07	1.12	-1.58	1.34	1.18	1.3	1.53
ILMN_1354584	LOC287558	1.15	1.25	-1.11	1.39	1.7	1.34	1.53
ILMN_1371920	Cyb5d2	1.12	-1.02	1.25	-1.04	1.43	1.37	1.53
ILMN_1360968	Arl5a	-1.06	1.23	1.23	1.2	1.35	1.38	1.53
ILMN_1354252	RGD1311805	-1.19	1.37	1.33	1.4	1.46	1.44	1.53
ILMN_2039023	RGD1560328_predicted	1.16	1.33	-1.39	1.17	1.46	1.5	1.53
ILMN_2039030	LOC499129	1.46	2.1	2.51	2.61	1.49	1.52	1.53
ILMN_1364918	Cyp51	-1.04	1.01	1.03	1.34	1.5	1.55	1.53
ILMN_1365182	Hsf2	1.23	1.54	1.33	1.53	1.54	1.58	1.53
ILMN_1359058	RGD1309995_predicted	1.28	1.68	1.61	1.78	2.11	1.81	1.53
ILMN_1351285	Rhob	1.12	1.69	1.19	1.14	2.28	2.24	1.53
ILMN_1374308	RGD1308127	1.14	1.32	1.45	1.5	2.05	2.25	1.53
ILMN_2039396	Nqo1	1.59	-1.92	-1.25	-1.33	-1.42	-1.27	1.54
ILMN_1650346	RGD1560732_predicted	1.35	-1.01	-1.15	1.17	1.07	-1.01	1.54
ILMN_1365622	Pcyt2	-1.06	1.07	1.41	-1.05	1.3	1.12	1.54
ILMN_1365521	Unr	1.05	1.01	1.1	1.17	1.4	1.19	1.54
ILMN_1373883	S100a11	1.41	-1.15	-1.63	1.11	1.22	1.22	1.54
ILMN_1352000	RGD1560496_predicted	-1.07	1.17	1.17	1.28	1.3	1.22	1.54
ILMN_1376289	Yif1b	1.18	1.18	1.18	-1.05	1.2	1.23	1.54
ILMN_1375843	Yif1b	1.18	1.18	1.18	-1.05	1.2	1.23	1.54
ILMN_1370811	Tm2d1_predicted	-1.02	1.34	1.37	1.22	1.49	1.31	1.54
ILMN_1349195	RGD1559610_predicted	1.23	1.32	1.44	1.45	1.39	1.32	1.54
ILMN_1356595	Churc1_predicted	1.06	1.4	-1.35	1.13	1.45	1.34	1.54
ILMN_1371537	Irak1bp1_predicted	1.25	1.49	1.49	1.36	1.39	1.39	1.54
ILMN_1372113	Alpl	1.23	1.55	2.24	1.79	1.45	1.39	1.54
ILMN_1365959	Cryl1	1.48	1.39	1.56	1.08	1.35	1.4	1.54
ILMN_1367781	RGD1561771_predicted	1.04	1.52	1.57	1.62	1.41	1.44	1.54
ILMN_1363661	Galm	1.1	1.23	1.31	-1.03	1.47	1.46	1.54
ILMN_1354634	Wdfy1	1.22	1.66	1.51	1.79	1.41	1.49	1.54
ILMN_1530375	Wdfy1	1.22	1.66	1.51	1.79	1.41	1.49	1.54
ILMN_1364794	LOC498407	1.02	1.18	1.51	1.57	1.4	1.5	1.54
ILMN_1372778	Limk1	1.15	1.72	1.26	1.94	1.77	1.5	1.54
ILMN_1373230	Slc17a3	1.39	1.35	1.22	1.44	1.65	1.52	1.54
ILMN_1360106	Commd8_predicted	-1.03	1.12	1.19	1.05	1.56	1.54	1.54
ILMN_1350699	RGD1562404_predicted	1.19	1.48	1.11	1.48	1.63	1.61	1.54
ILMN_1349995	Tap2	1.04	1.39	1.33	1.65	1.69	1.67	1.54
ILMN_1357312	Mcl1	1.15	1.54	1.27	1.23	1.7	1.73	1.54
ILMN_1376512	Sacm1l	-1.06	1.43	1.72	1.41	2.45	2.14	1.54

ILMN_1358691	Myadm	1.15	-1.29	-1.72	1.15	1.43	1.31	1.55
ILMN_1370134	RGD1563586_predicted	1.06	1.33	1.48	1.29	1.34	1.36	1.55
ILMN_1360892	Napa	1.02	1.03	1.45	-1.05	1.43	1.44	1.55
ILMN_1361601	LOC497793	-1.15	1.34	1.09	1.25	1.36	1.47	1.55
ILMN_2040297	Ndrg1	-1.13	-1.24	-1.2	1.09	1.51	1.48	1.55
ILMN_1366945	Cklf	1.29	1.35	-1.17	1.26	1.64	1.49	1.55
ILMN_1361194	Rab30	-1.02	1.23	1.32	1.5	1.64	1.53	1.55
ILMN_1356788	RGD1559865_predicted	1.06	1.14	-1.09	1.3	1.42	1.56	1.55
ILMN_1351640	Hspbap1	1.51	2.31	2.3	2.85	1.57	1.57	1.55
ILMN_1375110	Tmbim1	1.34	1.62	1.37	1.42	1.55	1.61	1.55
ILMN_1363106	Ndel1	1.13	1.49	1.52	1.35	1.73	1.61	1.55
ILMN_1369131	Rsbn1_predicted	1.09	1.67	1.77	1.65	1.84	1.74	1.55
ILMN_1354445	Rassf1	1.02	1.44	1.57	1.44	1.65	1.79	1.55
ILMN_1650031	RGD1306781_predicted	1.08	1.25	1.48	1.17	1.95	1.79	1.55
ILMN_1370554	Dhh	1.03	1.38	1.52	1.32	1.59	1.89	1.55
ILMN_1357381	Bet1	1.01	-1.38	1.05	-1.34	2.43	2.05	1.55
ILMN_1356719	Chka	1.28	2.33	2.09	1.55	2.42	2.13	1.55
ILMN_1370585	Armet_predicted	-1.07	1.25	1.46	1.44	2.77	2.59	1.55
ILMN_1372716	Ankrd24_predicted	1.19	1.21	1.19	1.23	-1.02	1.02	1.56
ILMN_1360584	Nckipsd_predicted	1.04	1.4	1.94	1.83	1.46	1.03	1.56
ILMN_1356793	F3	1.21	1.3	1.45	1.48	1.29	1.09	1.56
ILMN_1354150	Spg20	1.15	1.02	1.15	1.22	1.02	1.13	1.56
ILMN_1370683	Nfatc4	1.06	1.07	1.14	1.06	1.21	1.14	1.56
ILMN_1364860	Bop1	1.36	1.56	1.35	1.87	1.36	1.17	1.56
ILMN_1359642	Galnt11	1.05	1.35	1.22	1.33	1.23	1.2	1.56
ILMN_1371292	Wdr46	1.13	1.16	1.21	1.61	1.14	1.23	1.56
ILMN_1376721	Vti1a	1.1	1.42	1.32	1.46	1.14	1.24	1.56
ILMN_1349209	Lrp5_predicted	1.07	1.68	-1.23	1.41	1.27	1.24	1.56
ILMN_1351487	RT1-A1	-1.12	1.16	1.56	1.51	1.15	1.25	1.56
ILMN_1364550	Sv2b	-1.05	1.23	-1.08	1.39	1.53	1.3	1.56
ILMN_1372866	Slc25a20	1.05	1.13	1.34	1.09	1.58	1.34	1.56
ILMN_1354381	LOC367835	-1.03	1.5	1.51	1.54	1.39	1.37	1.56
ILMN_1360900	Wbp5_predicted	1.2	-1.24	-1.07	-1.23	1.41	1.38	1.56
ILMN_1361272	RGD1559972_predicted	1.13	1.42	1.1	1.53	1.48	1.38	1.56
ILMN_1356211	LOC367250	1.04	1.37	1.19	1.42	1.25	1.44	1.56
ILMN_1369298	Arhgap17	-1.12	1.18	1.19	1.2	1.44	1.48	1.56
ILMN_1348986	Arl6ip5	1.16	-1.01	1.12	1.07	1.6	1.48	1.56
ILMN_1368895	RGD1561599_predicted	1.2	1.74	1.4	1.43	1.44	1.64	1.56

ILMN_1374731	LOC497754	-1.01	1.06	1.35	1.18	1.22	1.68	1.56
ILMN_1354939	Btbd10	-1.05	1.58	1.44	1.5	1.81	1.77	1.56
ILMN_1370530	Ssr3	-1.09	1.13	1.23	1.09	1.98	1.79	1.56
ILMN_1375855	Dmtf1	1.18	1.98	1.37	1.9	1.85	1.83	1.56
ILMN_1376429	Dmtf1	1.18	1.98	1.37	1.9	1.85	1.83	1.56
ILMN_1375047	LOC501086	1.14	1.42	1.78	1.38	1.77	1.86	1.56
ILMN_1350576	LOC290704	-1.02	1.58	1.44	1.15	2.86	2.24	1.56
ILMN_1372949	RGD1562052_predicted	-1.14	1	1.02	1.03	2.2	2.25	1.56
ILMN_1353230	LOC497821	1.56	1.91	1.87	1.93	2.31	2.35	1.56
ILMN_1371299	RGD1308329_predicted	1.12	1.41	-1.02	1.57	1.19	1.09	1.57
ILMN_1356361	Ggh	-1.05	-1.02	1.27	1.12	1.27	1.11	1.57
ILMN_1359694	Lgals8	1.3	1.11	1.19	1.11	1.06	1.15	1.57
ILMN_1361109	RGD735065	1.2	1.21	1.16	-1.15	1.28	1.24	1.57
ILMN_1373727	LOC362803	1.13	-1	-1.07	1.07	1.31	1.27	1.57
ILMN_1365626	Oprs1	1.22	1.22	1.53	1.45	1.37	1.32	1.57
ILMN_1364195	LOC500437	1.37	1.25	1.43	1.47	1.18	1.34	1.57
ILMN_1360773	RT1-M3	1.08	1.19	1.28	1.28	1.6	1.37	1.57
ILMN_1369944	Rufy1	1.02	1.07	-1.09	1.3	1.28	1.41	1.57
ILMN_2039665	Timm9	1.22	1.24	1.47	1.57	1.5	1.44	1.57
ILMN_1351051	Tom1	-1.01	1.28	1.26	1.22	1.29	1.5	1.57
ILMN_1355722	RGD1310937_predicted	-1.04	1.28	1.14	-1.04	1.56	1.5	1.57
ILMN_1358698	RGD1565767_predicted	1.24	1.76	1.23	1.97	1.62	1.5	1.57
ILMN_1363878	LOC500621	1.11	-1	1.4	1.05	1.65	1.51	1.57
ILMN_1359871	Vac14	1.14	1.31	1.2	1.56	1.54	1.52	1.57
ILMN_1354260	Atp6v0e1	1.16	1.14	1.03	1.23	1.53	1.53	1.57
ILMN_1376806	Dcbld2	-1.05	1.09	1.2	1.43	1.36	1.58	1.57
ILMN_1358792	Atp6v0a1	1.09	1.12	-1.1	1.11	1.82	1.61	1.57
ILMN_1349328	RGD1564400_predicted	1.32	1.56	1.36	1.63	1.5	1.62	1.57
ILMN_1359096	Dph5	1.19	1.44	1.72	1.5	1.61	1.66	1.57
ILMN_1362012	Fndc3b_predicted	-1.02	1.49	1.23	1.6	1.9	1.91	1.57
ILMN_1375567	Kng1	-1.01	-1.53	-1.46	-1.4	1.01	-1.53	1.58
ILMN_1353995	Mcee_predicted	-1.14	-1.24	-1.83	-1.58	1.22	-1.06	1.58
ILMN_1376411	Ppp1r3c	1.26	1.71	1.91	1.84	1.54	1.18	1.58
ILMN_1375212	RGD1307772	-1.03	1.76	1.64	1.64	1.28	1.2	1.58
ILMN_1371817	LOC499902	-1.08	1.12	1.01	1.12	1.33	1.21	1.58
ILMN_1359711	LOC500652	-1.06	1.22	1.17	1.56	1.19	1.31	1.58
ILMN_1354291	Col4a3bp_predicted	1.11	1.07	-1.27	1.07	1.21	1.31	1.58
ILMN_1364059	RGD1309341	1.04	1.09	1.2	-1.06	1.22	1.35	1.58

ILMN_1370964	RGD1309701_predicted	1.02	-1.09	-1.03	1.03	1.5	1.35	1.58
ILMN_1369724	RGD1306148_predicted	1.31	1.44	1.32	1.45	1.44	1.42	1.58
ILMN_1370250	Tmem5	1.16	1.78	1.74	1.73	1.51	1.45	1.58
ILMN_1358146	Gtpbp8	1.05	1.71	1.68	1.61	1.62	1.49	1.58
ILMN_1368620	Txnl2	-1	1.16	1.23	1.23	1.45	1.67	1.58
ILMN_1366543	RGD1560119_predicted	1.06	1.93	1.71	1.8	1.47	1.72	1.58
ILMN_1349052	RGD1565940_predicted	1.2	1.79	1.51	1.6	2.21	1.79	1.58
ILMN_1368807	Zfyve27	1.14	1.63	1.22	1.41	2.16	1.96	1.58
ILMN_1367087	Fndc3a_predicted	-1.06	1.15	1.16	-1.03	2.49	2.23	1.58
ILMN_1364980	RGD1562691_predicted	-1.05	1.06	1.32	1.09	1	1.1	1.59
ILMN_1353297	LOC502599	1	1.39	1.11	1.87	1.37	1.18	1.59
ILMN_1362261	Nsdhl	-1.08	-1.31	1.07	-1.29	1.36	1.3	1.59
ILMN_1356706	Trit1_predicted	1.09	1.71	1.55	1.59	1.26	1.34	1.59
ILMN_1364806	Nostrin	1.01	1.92	1.84	1.51	1.75	1.4	1.59
ILMN_1650834	RGD1560073_predicted	1.09	1.31	1.04	1.7	1.82	1.43	1.59
ILMN_1370030	LOC498078	1.04	1.3	1.17	1.6	1.51	1.47	1.59
ILMN_1366910	Acly	-1.26	-1.04	1.05	1.25	1.62	1.51	1.59
ILMN_1368758	RGD1564005_predicted	1.24	2.31	2.58	2.25	1.61	1.58	1.59
ILMN_1371486	RGD1565301_predicted	1.15	1.26	1.02	1.47	1.78	1.65	1.59
ILMN_1374350	Inpp5a_predicted	1.14	1.57	1.81	1.79	1.89	1.93	1.59
ILMN_1351094	Sars1	1.11	1.79	1.73	1.95	1.9	1.97	1.59
ILMN_1363604	Slc15a4	1.37	2.45	1.93	2.72	1.98	2.42	1.59
ILMN_1352802	Cxadr	1.25	1.67	1.66	1.77	2.4	2.55	1.59
ILMN_1349130	Spcs2_predicted	1.01	-1.21	1.41	1.02	1.41	1.23	1.6
ILMN_1366171	RGD1304694	1.2	1.29	1.52	1.55	1.12	1.29	1.6
ILMN_1372238	Dncl2a	1.05	1.12	1.02	1.12	1.34	1.32	1.6
ILMN_1364084	Lgals4	1.06	2.57	1.72	1.58	1.83	1.34	1.6
ILMN_1367519	Idi1	1.02	-1.45	-1.36	-1.25	1.18	1.37	1.6
ILMN_1649782	RGD1309948	1.11	1.4	1.45	1.42	1.58	1.38	1.6
ILMN_1358890	Laptm4b	1.21	1.19	1.41	1.33	1.44	1.4	1.6
ILMN_1359801	RGD1566265_predicted	1	1.22	1.17	1.11	1.42	1.42	1.6
ILMN_1360333	RGD1562397_predicted	1.1	1.26	-1.26	1.51	1.38	1.47	1.6
ILMN_1349836	Spg21	1.01	1.54	1.63	1.35	1.47	1.48	1.6
ILMN_1369851	RGD1304624_predicted	1.05	1.55	1.71	1.59	1.95	1.48	1.6
ILMN_1372795	Arpp19	1.2	1.4	-1.05	1.52	1.39	1.51	1.6
ILMN_1369868	Ipo13	1.23	1.83	1.37	2.42	1.55	1.57	1.6
ILMN_2040743	Lias_predicted	1.35	1.6	2.02	1.54	1.49	1.63	1.6
ILMN_1362258	Vps11_predicted	-1.1	1.69	1.64	1.55	1.89	2.22	1.6

ILMN_1372631	Slc25a1	1.18	1.26	1.79	1.43	1.39	1.23	1.61
ILMN_1366033	LOC361163	1.05	-1.16	-1.13	1.06	1.12	1.29	1.61
ILMN_1369072	Pir	1.46	1.16	1.36	1.22	1.55	1.3	1.61
ILMN_1363683	RGD1565806_predicted	-1.02	1.56	-1.24	1.47	1.3	1.31	1.61
ILMN_1363325	Prdx5	1.11	1.29	1.25	1.2	1.48	1.51	1.61
ILMN_1363245	Csf1	-1.04	1.52	1.28	1.77	1.57	1.74	1.61
ILMN_1360723	Copa_predicted	-1.06	1.19	-1.13	1.2	1.73	1.77	1.61
ILMN_1368761	RGD1559720_predicted	1.16	1.34	1.09	1.61	1.99	1.77	1.61
ILMN_1363970	RGD1309228	-1.03	1.53	1.34	1.27	2.09	1.9	1.61
ILMN_1350707	LOC290549	1.01	1.08	1.01	1.02	1.89	1.92	1.61
ILMN_1364637	Mafg	1.41	1.95	1.58	1.65	1.78	1.98	1.61
ILMN_1357690	Pdia4	-1.06	1.27	1.46	1.15	2.41	2.25	1.61
ILMN_1365949	Hcfc1r1	1.01	1.41	-1.15	1.17	1.44	1.21	1.62
ILMN_1364145	Abhd3_predicted	-1.02	1.34	1.27	1.31	1.51	1.36	1.62
ILMN_1366294	Gstp2	-1.04	2.32	1.89	2.07	1.41	1.45	1.62
ILMN_1359516	Gclc	1.55	1.9	1.85	1.94	1.43	1.46	1.62
ILMN_1374010	RGD1561310_predicted	1.09	1.49	-1.59	1.45	1.39	1.51	1.62
ILMN_1365762	Pi4k2b	1.41	1.35	1.43	1.15	1.41	1.58	1.62
ILMN_1354661	Rcn1_predicted	1.04	1.43	1.13	1.45	1.66	1.58	1.62
ILMN_1356589	RGD1565520_predicted	1.05	1.39	1.08	1.13	1.28	1.63	1.62
ILMN_1369576	LOC680294	1.03	1.49	-1.2	1.46	1.41	1.67	1.62
ILMN_1650881	RGD1304876_predicted	1.08	1.37	1.64	1.44	1.75	1.68	1.62
ILMN_1368630	RGD1309660_predicted	1.15	1.89	1.98	1.88	1.75	1.78	1.62
ILMN_1370076	Zfand2b	1.15	2.29	1.74	2.19	2.39	2.05	1.62
ILMN_1372285	RGD1305466	-1.02	1.25	1.45	1.19	2.08	2.09	1.62
ILMN_1364809	Crip2	1.09	2.11	2.03	3.07	2.15	2.12	1.62
ILMN_1372213	LOC360527	1.17	1.42	1.3	1.57	1.98	2.14	1.62
ILMN_1372377	Slc3a2	1.42	2.29	2.04	1.87	1.91	2.16	1.62
ILMN_1361158	Slc33a1	1.34	1.71	1.71	1.65	2.33	2.5	1.62
ILMN_1376509	Mrlcb	1.1	1.03	-1.33	-1.27	1.04	-1.1	1.63
ILMN_1650504	RGD1561507_predicted	1.03	1.3	-1.07	1.19	1.28	1	1.63
ILMN_1359704	LOC307332	1.24	1.16	1.27	1.33	1.13	1.13	1.63
ILMN_1356431	RGD1560158_predicted	1.07	1.18	-1.09	1.36	1.27	1.18	1.63
ILMN_1357072	LOC500668	1	1.05	1.1	1.04	1.19	1.19	1.63
ILMN_1376258	Ddit4l	1.49	-1.13	-1.02	1.2	1.05	1.27	1.63
ILMN_1530410	Tm7sf2	-1.05	-1	1.35	1.11	1.62	1.31	1.63
ILMN_1358232	Tm7sf2	-1.05	-1	1.35	1.11	1.62	1.31	1.63
ILMN_1351663	Tspyl	-1.04	1.12	1.1	-1.03	1.77	1.62	1.63

ILMN_1349049	LOC501441	1.03	-1.13	-1.15	-1.04	1.68	1.67	1.63
ILMN_1360461	LOC313450	1.26	1.62	1.52	2.34	1.76	1.67	1.63
ILMN_1373551	RGD1564744_predicted	-1.03	1.13	-1.05	1.38	1.62	1.77	1.63
ILMN_1361375	Prr7	1.46	3.88	2.52	3.57	2.55	2.58	1.63
ILMN_1372755	Eif4a2	-1.08	1.28	1.37	1.57	1.23	1.29	1.64
ILMN_1369810	LOC297591	1.04	-1.01	-1.36	-1.03	1.33	1.3	1.64
ILMN_1372394	Unc119	1.02	1.62	1.89	1.85	1.44	1.39	1.64
ILMN_1361243	Ptpn23	-1.11	1.23	-1.01	1.37	1.61	1.4	1.64
ILMN_1370572	RGD1563264_predicted	1.12	1.44	1.27	1.76	1.29	1.52	1.64
ILMN_1367692	Rds	1.45	1.66	1.5	1.5	1.27	1.63	1.64
ILMN_1353590	Gadd45b	1.07	1.79	1.26	1.43	1.45	1.68	1.64
ILMN_1354575	Ppid	-1.1	1.51	1.27	1.97	1.48	1.68	1.64
ILMN_1361286	Ppid	-1.1	1.51	1.27	1.97	1.48	1.68	1.64
ILMN_1356345	RGD1562381_predicted	1.1	1.2	-1.03	1.49	1.69	1.71	1.64
ILMN_1371855	Vps35_mapped	1.06	1.61	1.63	1.74	1.8	1.72	1.64
ILMN_1374863	RGD1564795_predicted	1.14	1.17	1.29	1.47	1.62	1.73	1.64
ILMN_1359050	Fbxo6b	1.04	1.54	1.48	1.29	1.86	1.84	1.64
ILMN_1355873	RGD1308251_predicted	1.22	1.59	1.57	1.62	1.12	-1.05	1.65
ILMN_1376355	Slc39a8	1.11	1.32	1.09	1.17	1.37	1.4	1.65
ILMN_1359736	Atf5	1.13	1.62	1.79	1.59	1.28	1.41	1.65
ILMN_1353559	Ubxd5	-1.09	1.49	1.35	1.3	1.54	1.42	1.65
ILMN_1365151	RGD1309400_predicted	-1.01	1.28	-1	1.33	1.81	1.59	1.65
ILMN_1351124	Sec13l1	1.03	1	1.39	1.39	1.37	1.61	1.65
ILMN_1367271	Tax1bp1	1.01	1.3	1.21	1.13	1.48	1.63	1.65
ILMN_1376621	Adck4	1.22	1.58	1.85	1.4	1.43	1.66	1.65
ILMN_1369633	Map3k1	1.05	1.85	1.2	1.63	1.49	1.72	1.65
ILMN_1356958	RGD1560979_predicted	-1.17	1.23	-1.19	1.35	1.96	1.76	1.65
ILMN_1376740	Glipr1	1.48	2.02	1.83	2.49	1.93	1.79	1.65
ILMN_1366649	LOC501087	1.27	1.12	1.65	1.43	1.73	1.87	1.65
ILMN_1369675	RGD1310433_predicted	1.59	1.85	1.09	1.88	2.26	1.96	1.65
ILMN_1360262	RGD1306067	-1.05	1.22	1.29	1.49	1.32	-1.04	1.66
ILMN_1374866	Hibch	1.16	1.45	1.41	-1.01	1.41	1.29	1.66
ILMN_1353563	Aacs	1.15	1.36	1.56	1.63	1.59	1.31	1.66
ILMN_1355517	Ankrd46	1.26	1.49	1.78	2.07	1.3	1.36	1.66
ILMN_1365962	Bre	1.02	1.17	1.07	-1.12	1.38	1.46	1.66
ILMN_1361079	LOC497745	-1.03	1.13	1.23	1.04	1.46	1.57	1.66
ILMN_1350504	Hint3	1.01	1.17	1.33	-1.07	1.55	1.59	1.66
ILMN_1356116	Pdcd2	1.29	2.55	2.23	2.41	2.09	2.03	1.66

ILMN_1350404	Ypel5	-1.02	-1.05	1.52	1.13	2.41	2.17	1.66
ILMN_1650784	Gadd45a	1.27	-1.03	1.17	1.2	3.37	2.49	1.66
ILMN_1356200	Dnajc1_predicted	1.21	1.43	1.45	1.39	1.49	1.29	1.67
ILMN_1369814	Ldhb	-1.04	2.13	2.42	1.87	1.42	1.3	1.67
ILMN_1374977	RGD1562953_predicted	1.18	1.44	-1.04	1.53	1.61	1.35	1.67
ILMN_1366138	LOC306428	-1.95	-1.43	-1.34	1.39	1.08	1.41	1.67
ILMN_1353992	RGD1309051	1.23	1.52	1.6	1.37	1.82	1.43	1.67
ILMN_1361911	RGD1305976_predicted	1.07	1.36	1.21	1.24	1.53	1.5	1.67
ILMN_1368173	Ap3s1_predicted	-1.25	-1.07	1.07	1.14	1.77	1.7	1.67
ILMN_1353889	MGC94720	-1.09	1.6	1.73	1.69	1.64	1.71	1.67
ILMN_1364648	Rb1cc1_predicted	1.12	1.47	1.19	1.32	1.88	2.12	1.67
ILMN_1372527	Ap1s2_predicted	-1.05	1.23	1.38	1.57	1.99	2.34	1.67
ILMN_1361975	Map2k1	1.07	1.49	1.73	1.92	2.64	2.37	1.67
ILMN_1530391	MGC95092	-1.15	1	1.14	-1.56	1.21	1.05	1.68
ILMN_1357051	MGC95092	-1.15	1	1.14	-1.56	1.21	1.05	1.68
ILMN_1364143	Vps53_predicted	-1.03	1.16	1.35	1.2	1.33	1.32	1.68
ILMN_1354287	Metapl1_predicted	1.05	2.13	1.84	1.49	1.55	1.35	1.68
ILMN_1373043	S100b	1.57	1.2	1.15	1.09	1.25	1.39	1.68
ILMN_1530372	Reep6	1.06	1.57	2.44	1.79	1.59	1.49	1.68
ILMN_1371225	Reep6	1.06	1.57	2.44	1.79	1.59	1.49	1.68
ILMN_1369452	LOC300350	1.19	1.7	1.46	1.75	1.79	1.58	1.68
ILMN_1349707	Katnal1	1.14	2.12	1.92	1.7	1.43	1.63	1.68
ILMN_1366634	LOC690038	1.72	1.4	1.7	1.89	1.45	1.63	1.68
ILMN_1650062	RGD1563903_predicted	1.1	1.54	1.19	1.65	1.5	1.67	1.68
ILMN_1351503	LOC684988	1.11	1.54	-1.07	1.6	1.68	1.77	1.68
ILMN_1363095	RGD1563135_predicted	-1.01	1.62	-1.38	1.68	1.95	1.81	1.68
ILMN_1355642	Mrpl17	1.11	1.44	1.09	1.6	1.07	-1.13	1.69
ILMN_1357481	Apoc2_predicted	1.09	1.27	1.16	1.26	1.14	1.19	1.69
ILMN_1374638	RGD1559846_predicted	1.02	1.42	1.08	1.36	1.23	1.34	1.69
ILMN_1354927	Ethe1_predicted	1.04	1.38	1.36	1.4	1.39	1.43	1.69
ILMN_1373500	Sdfr1	1.04	1.01	1	1.07	1.65	1.51	1.69
ILMN_1363845	Mfsd1_predicted	1.17	1.28	1.48	1.27	1.38	1.61	1.69
ILMN_1352918	Harpb64	1.08	2.13	2.04	1.9	1.83	1.73	1.69
ILMN_1374838	Zfp364_predicted	1.06	1.31	1.37	1.52	1.58	1.83	1.69
ILMN_1358659	LOC298495	1.23	1.49	-1.05	1.91	1.87	1.9	1.69
ILMN_1372236	Zfp36l1	-1.12	2.22	1.45	1.17	1.86	1.91	1.69
ILMN_1353564	Plaa	1.38	1.96	1.58	1.91	2.2	1.92	1.69
ILMN_1363363	RGD735175	1.23	1.33	-1.14	-1.04	1.48	2.09	1.69

ILMN_1375808	Atp6v1c1_predicted	1.32	1.59	2.59	1.8	1.93	2.14	1.69
ILMN_1349454	Hist1h2bp_predicted	-1.03	1.33	1.89	1.46	1.53	2.48	1.69
ILMN_1371344	Cebpb	1.87	2.77	2.18	2.33	2.34	2.63	1.69
ILMN_1358352	Vdp	1.07	1.69	2	1.56	3.45	2.86	1.69
ILMN_1650195	LOC502332	1.12	1.24	-1.07	1.17	1.24	1.24	1.7
ILMN_1361542	Srp14_predicted	1.07	1.26	-1	1.26	1.47	1.27	1.7
ILMN_1353519	Mcts1	1.03	1.08	-1.14	1.17	1.2	1.37	1.7
ILMN_1371695	LOC366258	-1.05	1.49	1.4	1.39	1.22	1.39	1.7
ILMN_1355756	Csda	1.12	1.3	1.34	1.3	1.52	1.41	1.7
ILMN_1364947	Scamp4	-1.03	1.31	1.22	1.43	1.47	1.44	1.7
ILMN_1366721	Cxcl16	1.27	1.11	1.31	1.32	1.51	1.45	1.7
ILMN_1362088	Hnrpab	1.04	1.89	2.23	2.15	2.3	1.52	1.7
ILMN_1370353	LOC499978	1.02	1.31	1.27	1.15	1.68	1.53	1.7
ILMN_1366665	LOC498095	1.13	2.05	1.79	1.75	1.57	1.55	1.7
ILMN_1366124	RGD1559986_predicted	1.17	1.81	1.66	1.88	1.62	1.62	1.7
ILMN_1361561	Pten	1.13	1.23	1.23	1.29	1.51	1.68	1.7
ILMN_1370451	Cebpg	1.31	1.89	1.44	1.98	1.8	1.68	1.7
ILMN_1649993	LOC499474	1.03	1.28	1.52	1.31	1.73	1.7	1.7
ILMN_1373745	Bri3	1.17	1.55	1.75	1.63	1.63	1.75	1.7
ILMN_1367254	RGD1565793_predicted	-1.01	1.62	1.87	1.74	1.9	2	1.7
ILMN_1372068	Hdac5	1.12	2.29	1.56	1.63	2.44	2.5	1.7
ILMN_1366377	Mal2	1.3	1.53	1.44	1.6	1.17	-1.02	1.71
ILMN_1649818	S100a16_predicted	-1.03	1.1	1.03	1.3	1.16	1.11	1.71
ILMN_1372532	RGD1565840_predicted	-1.06	1.37	1.54	1.35	1.25	1.26	1.71
ILMN_1366970	Ifi44	1.07	1.11	1.02	-1.02	1.3	1.29	1.71
ILMN_1372241	Asl	-1.03	1.56	1.91	1.9	1.89	1.43	1.71
ILMN_1356210	LOC498350	1.14	1.1	1.25	-1.07	1.81	1.49	1.71
ILMN_1369771	Chpt1	-1.03	1.28	1.2	1.49	1.62	1.53	1.71
ILMN_1650694	Atp6v1g1_predicted	1.13	1.51	1.29	1.65	1.66	1.66	1.71
ILMN_1370862	Upp1	1.27	1.31	1.49	1.57	1.7	1.79	1.71
ILMN_1367300	Phr1_predicted	1.12	1.96	1.96	2.25	1.82	1.84	1.71
ILMN_1372419	LOC316326	-1.06	2.04	1.59	1.29	3.27	3.37	1.71
ILMN_1368814	Adi1	1.12	1.07	1.83	1.28	1.4	1.2	1.72
ILMN_1651177	Ddb1	1.16	1.18	1.35	1.17	1.38	1.26	1.72
ILMN_1365278	Sdccag8	1.26	1.38	1.41	1.2	1.84	1.46	1.72
ILMN_1364046	LOC361172	1.1	1.26	1.41	1.28	1.77	1.49	1.72
ILMN_1369530	Cat	1.44	1.04	1.44	-1.01	1.33	1.52	1.72
ILMN_1362194	RGD1565306_predicted	1.18	1.56	1.21	1.62	1.46	1.72	1.72

ILMN_1350073	Lrp12_predicted	1.24	1.82	1.93	1.81	1.82	1.82	1.72
ILMN_1651100	RGD1304601	-1.2	1.38	1.6	1.22	1.35	1.09	1.73
ILMN_1351098	RGD1564580_predicted	1.08	1.41	-1.07	1.53	1.47	1.27	1.73
ILMN_1355860	Gstk1	1.18	1.86	1.33	1.99	1.37	1.33	1.73
ILMN_1374954	Ap2b1	1.05	1.01	1.85	1.33	1.53	1.42	1.73
ILMN_1354887	RGD1565411_predicted	1.14	2.25	1.83	1.95	1.58	1.51	1.73
ILMN_1373933	mrpl24	1.13	1.13	1.32	1.43	1.64	1.61	1.73
ILMN_1366981	Ftl1	1.45	1.82	2.43	1.66	1.48	1.66	1.73
ILMN_1376270	RT1-CE7	1.13	1.1	1.13	1.08	2.03	1.87	1.73
ILMN_1351679	LOC500040	1.26	1.53	1.63	1.39	3.24	3.04	1.73
ILMN_1363259	LOC687579	1.25	1.13	-1.46	1.11	1.42	1.18	1.74
ILMN_1363545	LOC500536	-1.09	1.63	1.59	1.27	1.61	1.22	1.74
ILMN_1359207	RGD1311578	1.12	1.33	1.47	1.52	1.43	1.23	1.74
ILMN_1352524	Usmg5	1.04	-1.14	-1.9	1.2	1.18	1.26	1.74
ILMN_1376434	Pgrmc1	1.36	-1.11	1.01	-1.02	1.26	1.28	1.74
ILMN_1351040	RGD1560903_predicted	1	1.28	1.09	1.71	1.4	1.39	1.74
ILMN_1359298	RGD1311558_predicted	1.23	1.38	1.48	1.61	1.59	1.43	1.74
ILMN_1363371	Nmd3_predicted	1.29	1.99	1.75	1.75	1.56	1.45	1.74
ILMN_1362335	LOC316130	1.01	1.75	1.64	1.72	1.67	1.54	1.74
ILMN_1363003	Lrp8_predicted	1.5	1.91	1.91	3.08	1.5	1.58	1.74
ILMN_1369568	RGD1310879_predicted	1.68	1.32	1.34	1.39	2.4	1.99	1.74
ILMN_2039008	Cdk105	1.11	1.56	-1.1	1.48	1.26	1.19	1.75
ILMN_1375078	RGD1561154_predicted	-2.26	-1.41	-1.04	1.4	-1.28	1.35	1.75
ILMN_1353729	Pgd	1.73	-1.39	1.71	1.02	1.36	1.41	1.75
ILMN_1367581	RGD1560687_predicted	1.38	1.88	2.47	1.62	1.6	1.53	1.75
ILMN_1366337	RGD1560687_predicted	1.38	1.88	2.47	1.62	1.6	1.53	1.75
ILMN_1355813	Nrip1_predicted	1.08	2.05	1.72	1.75	1.77	1.61	1.75
ILMN_1362918	LOC361912	-1.05	1.11	1.53	1.22	1.52	1.66	1.75
ILMN_1365996	Gga2	1.06	1.31	1.68	1.56	1.91	1.71	1.75
ILMN_1361879	Cidea_predicted	1.21	1.54	1.41	1.44	2.01	1.73	1.75
ILMN_1367745	Il3ra	1.06	1.21	1.47	1.09	1.21	1.75	1.75
ILMN_1361650	RGD1563988_predicted	1.08	-1.12	-1.2	-1.28	1.3	1.24	1.76
ILMN_1374122	LOC364763	1.37	1.68	1.75	1.88	1.59	1.56	1.76
ILMN_1530495	LOC303332	1.16	2.19	2.36	2.53	1.78	1.72	1.76
ILMN_1348905	LOC303332	1.16	2.19	2.36	2.53	1.78	1.72	1.76
ILMN_1352122	Npc2	1.01	1.19	1.53	1.13	1.52	1.79	1.76
ILMN_1374780	Gpr108	-1.08	1.7	2.09	1.69	1.76	1.96	1.76
ILMN_1357658	Wsb1	-1.02	2.16	1.85	1.55	2.44	2.18	1.76

ILMN_1360950	Dscr3_predicted	1.09	1.58	1.97	1.59	2.18	2.24	1.76
ILMN_1369340	RGD1562933_predicted	1.04	1.12	1.9	1.2	1.12	1.4	1.77
ILMN_1374692	Dpm1_predicted	1.13	1.57	1.45	1.43	1.59	1.46	1.77
ILMN_1350513	Comt	1	1.37	1.22	1.3	1.74	1.53	1.77
ILMN_1360518	Rras_predicted	1.16	1.35	1.39	1.49	1.77	1.62	1.77
ILMN_1356995	Rps12	1.11	1.4	-1.02	1.5	1.68	1.65	1.77
ILMN_1371313	Pla2g12a_predicted	1.45	2.05	1.9	1.59	1.57	1.66	1.77
ILMN_1364317	RGD1307392_predicted	1.06	1.27	1.01	1.21	1.9	1.66	1.77
ILMN_1355495	Hebp1_predicted	1	1.34	1.29	1.57	1.67	1.26	1.78
ILMN_1357924	Gnl3	-1.03	1.66	1.46	1.54	1.62	1.33	1.78
ILMN_1372101	Rpl30	1.29	1.61	-1.36	1.45	1.42	1.54	1.78
ILMN_1370060	Mlx	-1.09	1.63	1.72	1.96	1.72	1.58	1.78
ILMN_1353636	Ap2a2	1.07	1.61	1.5	1.74	1.63	1.61	1.78
ILMN_1354107	Rpl37	1.24	1.57	-1.14	1.83	1.56	1.63	1.78
ILMN_1355049	Limk2	1.04	1.14	1.56	1.59	1.95	1.81	1.78
ILMN_1351385	Rabep1	1.27	2.19	2.09	2.29	2.08	1.94	1.78
ILMN_1371857	LOC291762	1.08	2.03	1.58	2.05	2.32	2.1	1.78
ILMN_1362392	RGD1565715_predicted	-1.02	-1.28	-1.38	-1.26	3.41	3.6	1.78
ILMN_1376361	Cda08	-1.07	-1.05	1.03	-1.07	1.56	1.41	1.79
ILMN_1376906	Khk	-1.01	1.75	1.94	1.34	1.63	1.45	1.79
ILMN_1363198	RGD1306682_predicted	-1.08	-1.05	-1.37	-1.08	1.85	1.63	1.79
ILMN_1363594	Zfp655	1.23	1.78	2.15	1.81	1.75	1.67	1.79
ILMN_1376917	RT1-M6-2	-1.03	1.17	1.43	1.27	1.85	1.67	1.79
ILMN_1360678	Tmem19	1.25	-1.09	1.04	-1.46	1.71	1.79	1.79
ILMN_1351336	Ift57_predicted	1.25	1.98	2.08	1.89	1.81	1.81	1.79
ILMN_1650695	Zfp99	1.88	1.3	1.42	1.12	1.75	2.17	1.79
ILMN_1371285	Maff_predicted	1.37	1.73	2.52	1.6	2.3	2.61	1.79
ILMN_1352329	Hrpap20	1.26	1.94	1.96	2.26	1.36	1.3	1.8
ILMN_1372785	MGC105647	1.27	1.88	1.55	1.76	1.68	1.72	1.8
ILMN_1367175	RGD1562265_predicted	1.19	1.41	-1.22	1.71	1.9	2.13	1.8
ILMN_1360860	Nthl1_predicted	1.21	1.31	1.45	1.27	1.3	1.44	1.81
ILMN_1369244	Mcfd2	-1.14	-1.01	1.32	1.4	1.59	1.47	1.81
ILMN_1357901	Fxr2h_predicted	1.39	1.62	1.54	2.03	1.59	1.69	1.81
ILMN_1361407	Ndfip2_predicted	1.15	1.18	1.28	1.14	1.8	1.81	1.81
ILMN_1358784	Gnl2	1.1	1.17	1.55	1.71	1.89	1.94	1.81
ILMN_1365261	Ppp3r1	1.03	1.44	3.77	2.34	2.12	2.18	1.81
ILMN_1351783	RGD1561205_predicted	1.19	1.43	1.79	1.92	1.74	1.44	1.82
ILMN_1354127	Lemd2	1.2	2.08	2.47	1.76	1.57	1.56	1.82

ILMN_1360054	RGD1560964_predicted	-1.1	1.56	1.75	1.81	1.82	1.65	1.82
ILMN_1353069	Cox17	1.17	1.85	1.11	1.75	1.69	1.68	1.82
ILMN_1369511	Bcar3_predicted	1.22	2.14	1.93	2.47	1.93	1.71	1.82
ILMN_1360580	Acss2_predicted	1.04	1.09	-1.13	-1.06	2.23	1.87	1.82
ILMN_1374022	Bdh2_predicted	1.19	2.15	2.05	1.89	1.96	2.06	1.82
ILMN_1360084	Atp6v1b2	1.13	1.36	1.81	1.65	2.16	2.14	1.82
ILMN_1369387	Parp3	1.01	1.4	1.53	1.42	1.67	1.15	1.83
ILMN_1363440	Tmem53_predicted	-1.09	1.48	1.38	1.47	1.59	1.55	1.83
ILMN_1350993	RGD1562008_predicted	1.05	1.36	1.2	1.06	1.44	1.61	1.83
ILMN_2039182	RGD1560118_predicted	1.38	1.93	2.01	1.68	1.73	1.77	1.83
ILMN_1370603	RGD1311161	1.3	1.54	1.67	1.49	2.45	2.16	1.83
ILMN_1363228	Ell2	1.31	1.53	1.63	1.9	2.66	2.4	1.83
ILMN_1358530	RGD1305178	-1.07	-1.05	1.14	1.02	1.08	1.13	1.84
ILMN_1352389	Snx8_predicted	-1.03	1.57	2.71	1.6	1.39	1.26	1.84
ILMN_1365055	Sptlc1_predicted	-1.05	-1.2	1.15	1.13	1.46	1.36	1.84
ILMN_1360063	LOC368084	1.2	1.17	2.67	1.73	1.24	1.46	1.84
ILMN_1365937	LOC498759	1.32	1.32	1.36	1.24	1.39	1.55	1.84
ILMN_1376604	S100a1	1.1	1.13	-1.08	1.49	2.02	1.98	1.84
ILMN_1357547	Centg2_predicted	1.4	1.7	1.94	1.84	1.97	2.01	1.84
ILMN_1358347	Pls3	1.53	1.11	1.45	1.33	1.85	2.03	1.84
ILMN_1359627	LOC360919	1.4	1.58	1.38	1.58	2.17	2.1	1.84
ILMN_2039303	Pelo	1.18	1.64	1.44	1.74	2.38	2.54	1.84
ILMN_1360715	LOC360303	-1.09	1.11	1.05	1.24	1.27	1.29	1.85
ILMN_1370027	LOC302640	1.16	1.33	1.35	1.36	1.8	1.43	1.85
ILMN_1359406	RGD1561926_predicted	1.22	1.36	1.7	1.2	1.81	1.7	1.85
ILMN_1374191	Scd1	1.16	1.23	1.33	1.38	2.45	2.1	1.85
ILMN_1356055	Atrn	1.36	2.39	1.79	1.97	2.8	3.09	1.85
ILMN_1363606	Lcn2	-1.08	1.4	1.79	1.96	5.26	4.21	1.85
ILMN_1376479	Gstp1	1.08	1.51	3.34	1.78	1.2	1.34	1.86
ILMN_1362913	LOC287996	1.13	1.45	-1.17	1.75	1.35	1.34	1.86
ILMN_1351805	Prdx6	1.57	1.41	1.46	1.25	1.36	1.42	1.86
ILMN_1375101	Vps26	1.1	1.56	1.76	1.6	1.67	1.5	1.86
ILMN_1368030	Stx8	1.13	1.32	1.36	1.39	1.78	1.52	1.86
ILMN_1349114	Serpinb6a	1.05	1.03	1.18	-1.08	1.88	1.6	1.86
ILMN_1351016	RGD1562153_predicted	1.5	1.64	1.2	1.62	1.59	1.71	1.86
ILMN_1356712	Ptrh1_predicted	-1.1	1.65	1.7	1.71	1.66	1.77	1.86
ILMN_1374662	RGD1565690_predicted	1.03	2.43	2.01	1.88	2.55	2.6	1.86
ILMN_1360285	Mocos_predicted	1.39	1.47	1.16	1.32	1.81	1.56	1.87

ILMN_1357318	RGD1561944_predicted	1.08	1.44	1.04	1.58	1.39	1.61	1.87
ILMN_1368656	B2m	1.15	1.68	1.54	1.68	1.86	1.84	1.87
ILMN_1360361	Scin	1.3	1.57	1.53	1.23	2.31	1.97	1.87
ILMN_1354246	RGD1308877_predicted	1.2	2.32	1.89	2.45	1.51	1.5	1.88
ILMN_1351707	RGD1560706_predicted	1.18	1.65	1.07	1.58	1.68	1.72	1.88
ILMN_1353399	Hexa	-1.08	1.2	1.07	1.01	1.82	1.83	1.88
ILMN_1371589	Scamp1	1.12	1.7	1.82	1.7	1.68	1.89	1.88
ILMN_1355120	RGD1563679_predicted	1.09	1.36	1.1	1.79	1.34	1.42	1.89
ILMN_1372525	RGD1564450_predicted	1.34	1.55	1.08	1.61	1.59	1.46	1.89
ILMN_1363881	LOC498449	1.16	1.4	1.93	1.69	1.75	1.56	1.89
ILMN_1368049	Rabac1	1.13	1.55	1.32	1.24	1.48	1.61	1.89
ILMN_1376726	Dgat2	1.33	1.55	1.92	1.88	1.8	1.68	1.89
ILMN_1364854	Sqle	1.25	-1.1	1.28	1.4	2.01	1.81	1.89
ILMN_1372022	Gch	1.3	1.63	1.58	1.75	2.43	1.82	1.89
ILMN_1361302	Klf5	1.76	1.68	1.41	1.86	1.85	1.86	1.89
ILMN_1376992	Pld3	1.11	1.75	1.79	1.63	1.69	1.45	1.9
ILMN_1372727	RGD1564862_predicted	1.28	1.61	1.56	1.37	1.54	1.68	1.9
ILMN_1361446	RGD1305222_predicted	1.25	2.01	1.87	3	1.58	1.71	1.9
ILMN_1363808	Ssu72	1.14	-1.02	1.47	1.08	2.31	2.02	1.9
ILMN_1353357	RGD1564980_predicted	1.35	1.35	1	1.57	1.51	1.45	1.91
ILMN_1355714	Tcn2	-1.03	1.6	1.49	1.45	1.71	1.76	1.91
ILMN_1351402	LOC498954	1.07	1.56	-1.17	1.66	1.88	2.05	1.91
ILMN_1370157	Cst6	1.39	2.02	1.53	2.08	2.08	2.28	1.91
ILMN_1371034	Acsl4	1.23	1.23	1.26	1.02	1.34	1.15	1.92
ILMN_1376915	LOC302898	1.02	1.43	1.19	1.42	1.59	1.47	1.92
ILMN_1354948	LOC497816	1.2	1.7	1.46	1.61	2.11	1.83	1.92
ILMN_1353531	Mapk6	1.35	2.08	1.97	2.26	1.89	1.9	1.92
ILMN_1349127	Alkbh3	-1.07	1.4	1.45	1.48	1.93	1.9	1.92
ILMN_1371962	Ier3	1.46	1.33	1.27	1.4	2.21	2.06	1.92
ILMN_1354748	Okl38	2.04	2.17	1.77	1.96	1.98	2.19	1.92
ILMN_1349555	RGD1359509	1.07	1.64	1.64	1.67	2.86	2.62	1.92
ILMN_1369315	Cyp3a13	-1.12	1.2	1.32	1.53	2.83	3.25	1.92
ILMN_1362746	Sdc1	1.22	1.42	1.52	2.16	1.87	1.45	1.93
ILMN_1363166	RGD1564055_predicted	1.06	1.38	-1.25	1.64	1.47	1.59	1.93
ILMN_1358297	RGD1562427_predicted	1.12	1.43	-1.21	1.5	1.78	1.61	1.93
ILMN_1358829	Ugp2	1.28	1.67	1.48	1.17	1.9	1.72	1.93
ILMN_1364727	LOC288515	-1.14	2.16	1.86	3.48	2.28	2.55	1.93
ILMN_1354711	LOC678833	1.36	1.61	1.48	1.52	1.76	1.29	1.94

ILMN_1367423	Atp6v1h	-1.01	1.26	1.62	1.59	2.22	1.78	1.94
ILMN_1367473	RT1-CE15	-1.02	1.09	1.24	1.35	2.09	1.79	1.94
ILMN_1374180	Bhlhb2	1.29	2.83	3.42	3.01	2.01	1.8	1.94
ILMN_1374125	LOC686771	1.11	2.22	1.71	2.5	2.27	1.84	1.94
ILMN_1376513	Ctsb	1.1	1.07	1.22	1.33	1.81	1.96	1.94
ILMN_1354484	RAMP4	1.13	1.91	2.57	2.16	3.11	2.63	1.94
ILMN_1356658	LOC363328	-1.04	2.07	1.45	2.16	2.8	2.78	1.94
ILMN_1374066	Mte1	1.19	2.45	2.79	3.14	3.74	3.86	1.94
ILMN_1355097	Hdac2	-1.05	1.07	1.21	1.31	1.41	1.28	1.95
ILMN_1365062	Abhd4_predicted	1.07	-1.24	-1.25	-1.47	1.16	1.43	1.95
ILMN_1357348	Nrg1	1.27	1.12	1.11	1.5	1.33	1.68	1.95
ILMN_1375496	Sqstm1	1.88	1.63	2.53	1.87	2.19	2.38	1.95
ILMN_1376894	Sqstm1	1.88	1.63	2.53	1.87	2.19	2.38	1.95
ILMN_1376089	Sqstm1	1.88	1.63	2.53	1.87	2.19	2.38	1.95
ILMN_1354820	Slc12a3	-1.1	1.6	-1.07	1.01	1.72	1.8	1.96
ILMN_1361470	LOC498559	-1.11	1.16	1.73	1.57	2.5	1.82	1.96
ILMN_1367248	Arl3	1.15	1.56	1.3	1.69	1.95	1.83	1.96
ILMN_1352579	MGC72560	-1.07	1.5	1.51	1.52	2.3	2.01	1.96
ILMN_2040666	RGD1563431_predicted	1.31	1.39	-1.44	1.63	1.8	2.08	1.96
ILMN_1369468	Slc35f5_predicted	1.2	1.55	1.88	1.65	1.76	2.09	1.96
ILMN_1368066	RGD1562971_predicted	1.22	1.56	1.01	1.54	1.76	1.47	1.98
ILMN_1374151	LOC294781	1.13	1.51	-1.04	1.7	1.63	1.82	1.98
ILMN_1363915	Ppp4r2_predicted	-1.1	1.06	1.18	1.12	2.69	2.34	1.98
ILMN_1372141	Sil1	-1.05	1.32	1.85	1.01	1.38	1.24	1.99
ILMN_1361562	St3gal4	1.12	1.33	1.57	1.87	1.3	1.29	1.99
ILMN_1368785	RGD1310348_predicted	1.11	1.26	1.58	1.67	1.87	1.62	1.99
ILMN_1365861	LOC687298	1.13	1.34	-1.34	1.51	1.9	1.67	1.99
ILMN_1374391	Rhoc_predicted	1.06	1.84	1.71	2.08	1.97	1.73	1.99
ILMN_1374781	Rbks_predicted	1.27	2	2.3	1.38	2.05	1.94	1.99
ILMN_1374309	RGD1562851_predicted	1.11	1.75	-1.05	1.54	1.89	1.95	1.99
ILMN_1366810	RGD1560511_predicted	1.05	1.59	1.6	1.24	2.07	1.95	1.99
ILMN_1368898	RGD1561587_predicted	1.35	1.61	-1.1	1.97	1.77	2.17	1.99
ILMN_1372999	Sdcbp	1.06	1.95	2.21	2.39	2.3	2.33	1.99
ILMN_1367396	Fbxo2	1.1	1.06	1.59	1.53	2.45	2.34	1.99
ILMN_1376787	Phlda3	1.07	1.29	1.25	1.89	2.11	2.43	1.99
ILMN_1354504	Lrp11_predicted	-1.11	1.46	1.21	1.27	1.57	1.54	2
ILMN_1357469	Myr8	1.17	1.65	1.53	1.71	1.7	1.92	2
ILMN_1364198	Sec11l3	1.09	1.22	1.24	1.24	2.35	1.95	2

ILMN_1352276	Ilvbl_predicted	1.15	1.98	2.27	1.63	1.76	2.02	2
ILMN_1376638	Atp6v0d1	-1.03	1.01	1.19	1.01	2.19	2.04	2
ILMN_1371789	LOC362919	1.12	1.63	1.49	1.63	2.22	2.11	2
ILMN_1365116	Strbp	1.18	1.83	2.72	2.35	2.39	2.22	2
ILMN_1362575	RGD1308165_predicted	1.73	2.42	2.57	2.71	2.1	2.4	2.01
ILMN_1364353	Cpeb2_predicted	-1.07	1.63	1.15	1.28	2.18	2.54	2.01
ILMN_1363160	Plac8_predicted	1.27	2.44	1.68	1.76	1.94	1.76	2.02
ILMN_1374501	Pex19	-1.08	1.39	1.44	1.18	2.12	1.76	2.02
ILMN_1370639	RGD1566373_predicted	1.24	1.61	1.06	1.66	1.76	1.83	2.02
ILMN_1362341	RGD1306404_predicted	1.03	1.8	1.84	2.14	1.85	1.83	2.02
ILMN_1356892	Bnip3	1.07	1.1	1.24	1.45	3.62	2.71	2.02
ILMN_1367486	Dusp1	1.4	1.08	1.11	-1.3	3.45	4.15	2.02
ILMN_1365447	Cryab	1.18	1.14	1.11	1.55	1.83	1.91	2.03
ILMN_1362382	RGD1559461_predicted	1.18	1.38	1.4	1.8	1.72	2.04	2.03
ILMN_1354961	RGD1311435	1.1	1.67	1.87	1.63	2.14	2.28	2.03
ILMN_1370869	RGD1308059	1.26	1.25	1.03	1.19	1.48	1.59	2.04
ILMN_1370160	LOC363459	-1	1.39	1.12	1.44	1.86	1.86	2.04
ILMN_1357530	Bid3	1.77	3.27	2.92	3.03	2.6	2.76	2.04
ILMN_1355979	Hif1a	1.45	2.44	1.87	2.61	1.91	1.75	2.05
ILMN_1356486	RGD1306952	1.18	2.01	2.17	1.64	2.29	1.88	2.05
ILMN_1357684	Gtf2h1_predicted	1.56	2.87	2.79	2.99	2.55	2.38	2.05
ILMN_1368456	RGD1563958_predicted	1.09	1.6	-1.08	1.39	1.68	1.6	2.06
ILMN_1359834	Rab9	1.29	1.39	1.81	1.52	1.68	1.74	2.06
ILMN_1369806	RGD1559795_predicted	1.09	1.24	1.12	1.38	1.53	1.87	2.06
ILMN_1357240	RGD1564549_predicted	1.24	-1.02	-1.44	1.11	1.32	1.38	2.07
ILMN_1350561	RGD1306437	1.58	1.58	1.69	1.5	1.6	1.55	2.07
ILMN_1372837	RGD1565675_predicted	1.34	1.17	1.29	1.62	1.49	1.58	2.07
ILMN_1366645	RGD1562315_predicted	1.17	1.48	-1.09	1.59	1.88	1.85	2.07
ILMN_1354527	Insig2	1.2	1.44	1.49	1.16	2.1	1.93	2.07
ILMN_1364655	Optn	1.06	1.17	1.14	1.23	1.92	1.96	2.07
ILMN_1376347	Hrasls3	-1.02	-1.05	-1.46	-1.48	1.19	-1.2	2.08
ILMN_1354915	RGD1561871_predicted	-1.04	1.47	1.04	1.68	1.65	1.8	2.08
ILMN_1364260	Anxa2	1.18	1.29	1.22	1.83	1.96	1.8	2.08
ILMN_1367895	RGD1566002_predicted	1.07	2.1	1.06	1.96	1.77	1.91	2.08
ILMN_1650254	RGD1565371_predicted	1.07	-1.02	1.22	1.49	3.66	2.62	2.08
ILMN_1376569	Abcc2	1.55	2.86	2.58	2.47	2.03	2.74	2.08
ILMN_1367604	LOC497853	1.89	1.67	1.54	2.03	1.4	1.65	2.1
ILMN_2039089	Adh1	1.72	2.31	3.18	1.76	1.67	1.77	2.1

ILMN_1371487	Sema3b_predicted	1.32	2.42	2.16	2.79	1.57	1.82	2.1
ILMN_1354330	Maoa	1.18	1.2	1.74	1.55	1.73	1.94	2.1
ILMN_1364920	Snx10	2.06	1.5	1.89	1.31	2.52	2.27	2.1
ILMN_1369816	Eif1a	1.53	1.67	2.32	2.05	2.33	2.38	2.1
ILMN_1354242	Nucb2	1.1	1.94	2.3	1.99	3.42	2.85	2.1
ILMN_1352770	LOC501172	1.2	1.36	1.3	1.5	1.24	1.44	2.11
ILMN_1361168	RGD1563952_predicted	1.1	1.12	1.3	1.33	1.52	1.75	2.11
ILMN_1374034	Atp6ap2	1.18	1.63	2.07	2.18	3	3.12	2.12
ILMN_1359568	Arl2	-1.02	1.46	1.75	1.4	1.64	1.48	2.13
ILMN_1370706	LOC367923	1.32	1.76	-1.16	1.38	1.33	1.52	2.13
ILMN_1651085	B3gat3_predicted	1.16	1.81	1.57	2.17	2.25	2.12	2.13
ILMN_1359723	Chic2_predicted	1.37	1.6	1.32	1.47	2.08	2.18	2.13
ILMN_1351456	Sc4mol	1.23	1.08	1.7	1.13	2.03	2.2	2.14
ILMN_1370324	Eif2b3	1.72	2.93	3.61	3.32	2.61	2.46	2.14
ILMN_1650442	RGD1307973_predicted	1.27	2.53	2.1	2.74	2.54	2.54	2.14
ILMN_1365705	Jun	1.04	1.65	1.39	1.19	2.83	2.74	2.14
ILMN_1354816	LOC497674	1.16	2.11	2.13	2	2.07	1.79	2.15
ILMN_1372092	RGD1562469_predicted	-1	1.47	1.1	1.87	1.83	2.11	2.15
ILMN_1369438	LOC303407	1.39	2.16	2.65	1.94	2.05	2.31	2.15
ILMN_1359644	Atp6ap1	1.26	1.41	1.98	1.6	1.72	1.66	2.16
ILMN_1361075	RGD1560915_predicted	1.17	1.52	1.74	1.6	1.98	1.71	2.16
ILMN_1367107	Aqp8	1.33	2.3	2.11	1.97	2.33	2.23	2.16
ILMN_1349205	RGD1562905_predicted	-1.05	1.73	1.55	2.1	2.06	2.06	2.17
ILMN_1363277	Sc5d	1.12	1.66	1.67	1.71	2.32	2.07	2.17
ILMN_1372752	RGD1306058_predicted	1.41	1.95	1.5	2.08	2.29	2.11	2.17
ILMN_1650223	LOC364468	1.26	1.49	1.02	1.48	1.25	1.24	2.18
ILMN_1356992	RGD1559566_predicted	1.01	1.3	1.16	1.5	1.38	1.44	2.18
ILMN_1358259	Lamp2	1.13	2.74	2.68	2.15	2.41	2.34	2.18
ILMN_1357600	Acaa2	1.08	1.24	1.44	-1.13	1.69	1.43	2.19
ILMN_1650512	RGD1566244_predicted	1.07	2.05	1.82	1.45	1.72	1.65	2.19
ILMN_1357587	Zbtb4_predicted	1.09	1.76	1.4	1.94	2.03	1.65	2.19
ILMN_2040694	Cmas	1.41	1.94	1.66	1.78	1.87	1.72	2.19
ILMN_1359195	Rnaset2_predicted	-1.01	1.54	1.61	1.54	1.73	1.73	2.19
ILMN_1361269	Atp6v1d	-1.02	1.53	1.67	1.55	2.09	2.15	2.19
ILMN_1353689	Slc22a1	1.22	2.15	1.59	1.86	2.14	2.3	2.19
ILMN_1355677	RGD1305246	1.45	1.37	1.46	1.64	2.25	2.31	2.19
ILMN_1371786	Cpeb4_predicted	-1.08	1.8	1.69	1.67	2.92	2.76	2.19
ILMN_1354070	isg12(b)	-1.07	1.08	1.07	1.23	3.33	2.89	2.19

ILMN_1359738	Ralb	1.19	3.35	2.99	3.1	3.1	3.1	2.19
ILMN_1356203	Adm	1.1	1.22	1.46	1.75	1.7	2.04	2.2
ILMN_1354552	Rnf125_predicted	1.7	3.96	3.31	2.42	2.04	2.18	2.2
ILMN_1371109	RGD1561135_predicted	1.25	1.48	-1.04	1.85	1.68	1.71	2.21
ILMN_1375690	Sep-04	1.15	2.22	1.70	1.54	1.88	1.64	2.21
ILMN_1355544	LOC498276	1.14	3.25	2.86	2.86	2.18	2.18	2.22
ILMN_1360615	LOC503409	1.87	1.69	-6.03	1.72	1.99	1.63	2.23
ILMN_1376819	Sep-04	1.12	1.90	1.69	1.47	1.71	1.31	2.23
ILMN_1367739	Dynll2	1.13	1.55	1.16	1.59	2.32	1.81	2.24
ILMN_1376575	Pltp_predicted	1	1.37	1.58	1.42	1.87	2.01	2.24
ILMN_1360686	LOC300191	1.81	1.33	2.5	1.89	1.7	1.57	2.25
ILMN_1356608	LOC499201	1.09	1.55	2.17	1.62	1.41	1.59	2.25
ILMN_1356682	RGD1565429_predicted	1.27	1.91	-1.34	1.96	2	2.25	2.25
ILMN_1357555	LOC307907	1.16	1.86	1.74	2.38	3.55	2.48	2.25
ILMN_1352241	RGD1562392_predicted	1.44	2.43	3.67	2.35	3.39	3.04	2.25
ILMN_1360188	Nfil3	1.5	4.25	3.05	3.13	4.46	4.84	2.25
ILMN_1350307	Atp6v1f	1.12	1.81	1.23	1.68	1.59	1.62	2.26
ILMN_1367240	Angel1_predicted	1.25	1.46	1.92	1.19	1.85	1.8	2.26
ILMN_1351222	RGD1562929_predicted	1.11	1.56	1.12	1.83	1.69	1.82	2.27
ILMN_1354078	Neu1	1.04	1.79	2.14	2.31	2.14	2.06	2.27
ILMN_1372202	Slc7a11_predicted	1.52	1.46	1.18	1.48	2.18	2.14	2.27
ILMN_1368863	Mvd	1.02	1.14	1.49	1.09	2.44	2.8	2.27
ILMN_1376765	Lcp1	1.53	3.78	4.06	3.77	3	2.97	2.27
ILMN_1358854	RGD1560186_predicted	1.13	1.79	-1.47	1.68	2.07	1.79	2.28
ILMN_1357137	Litaf	-1.02	1.77	1.86	1.59	2.65	2.46	2.28
ILMN_1374967	isg12(a)	1.14	1.56	1.13	1.04	1.69	1.74	2.29
ILMN_1370948	Creg_predicted	1.53	1.86	1.59	1.34	1.34	1.25	2.3
ILMN_1357538	Lamp1	1.01	1.53	1.74	1.53	1.77	2.03	2.31
ILMN_1357241	Pmm1	1.25	1.46	1.71	1.81	2	2.34	2.31
ILMN_1363999	Gys2	1.26	4.58	3.53	2.81	3.11	3.09	2.31
ILMN_1351701	LOC499056	1.28	1.62	1.16	1.73	1.81	2.11	2.34
ILMN_1350983	Fdps	1.18	1.16	1.48	1.26	2.12	2.09	2.35
ILMN_1362780	Isg20l1_predicted	1.42	2.03	2.15	2.96	2.2	2.55	2.35
ILMN_1373045	Cyp2c70	1.04	2.71	1.47	4.58	3.69	3.28	2.35
ILMN_1651063	LOC686564	1.21	1.23	-1.33	1.48	1.28	1.44	2.36
ILMN_1370842	RGD1565037_predicted	1.07	1.58	1.24	1.29	2.39	2.05	2.36
ILMN_1354198	Uap1l1_predicted	1.09	2.78	3.09	1.92	2	1.56	2.37
ILMN_1351171	RGD1303130	1.16	1.84	2.09	2.03	2.25	1.97	2.37

ILMN_1351417	RGD1566396_predicted	1.35	2.13	2.34	2.26	1.88	2.31	2.37
ILMN_1369745	Avpi1	1.24	1.17	1.71	1.85	1.42	1.41	2.38
ILMN_1650063	Tacc2	1.84	2.1	1.61	1.87	2.14	2.37	2.38
ILMN_1376798	Tacc2	1.84	2.1	1.61	1.87	2.14	2.37	2.38
ILMN_1355503	Slc17a1	1.58	1.44	1.43	1.3	1.73	1.65	2.4
ILMN_1356573	RGD1563739_predicted	1.05	1.29	1.04	1.52	1.96	2.47	2.4
ILMN_1369158	Myd116	1.31	2.82	3.22	3.52	6.18	6.38	2.41
ILMN_1362479	Hsd3b7	1.28	1.39	1.57	1.49	1.63	1.68	2.43
ILMN_1352782	Tpp1	1.32	1.96	2.02	1.65	2.01	2.31	2.43
ILMN_1376957	Atp6v1c1	1.13	1.89	2.44	2.17	2.52	2.52	2.43
ILMN_1366201	Aldoa	1.26	1.82	2.51	2.22	2.66	2.64	2.43
ILMN_1367380	Aldoa	1.26	1.82	2.51	2.22	2.66	2.64	2.43
ILMN_1650955	Ifi27l	1.18	1.89	1.44	1.17	1.77	1.96	2.44
ILMN_1356160	Rnf11_predicted	1.1	1.46	1.71	1.4	2.63	2.54	2.44
ILMN_1367585	RGD1307315	1.2	2.07	2.22	2.02	2.67	2.76	2.44
ILMN_1371513	Gaa	1.12	2.01	1.6	1.69	1.88	2.01	2.46
ILMN_1370481	Ambp	1.06	4.39	4.19	3.8	2.45	2.8	2.47
ILMN_1354441	Ppfibp2	-1.42	1.03	1.11	1.25	1.3	1.65	2.48
ILMN_1351286	LOC679886	1.17	1.09	1.19	1.42	2.72	2	2.49
ILMN_1354182	LOC499654	1.04	1.72	2.05	2.63	2.18	2.1	2.5
ILMN_1366792	Ankrd13d_predicted	1.11	2.52	2.67	2.36	1.91	2.13	2.5
ILMN_1376594	Ugcg	-1.03	1.44	1.17	1.44	2.12	1.91	2.51
ILMN_1360727	LOC292088	1.08	2.21	1.17	2.21	2.58	2.47	2.51
ILMN_1651019	Tcte1l	-1	1.23	1.44	1.23	1.66	1.61	2.52
ILMN_1370366	Dpp7	1.05	2.67	2.65	2.15	1.96	2.05	2.52
ILMN_1354313	RGD1311422_predicted	1.12	2.41	2.33	3.02	4.14	3.39	2.54
ILMN_1357299	RGD1562055_predicted	1.2	1.5	-1.11	1.67	1.99	1.83	2.55
ILMN_1651088	Anxa7	1.17	2.06	1.74	1.74	2.55	2.22	2.55
ILMN_1368144	Casp11	1.56	2.85	5.04	3.47	5.29	4.86	2.55
ILMN_1366430	LOC498363	1.31	1.73	-2	2.01	2.11	2.48	2.56
ILMN_1365021	LOC315973	1.17	1.32	1.47	-1.07	2.15	2.02	2.57
ILMN_1358078	Hbxip_predicted	1.6	2	1.84	2.11	2.54	2.73	2.59
ILMN_1373648	Mx1	1.28	1.21	2.03	1.96	2.39	2.79	2.59
ILMN_1366784	Vat1	1.12	2.25	1.73	2.65	2.47	2.57	2.61
ILMN_1363083	Atf3	1.05	1.91	1.94	1.89	5.2	5.77	2.61
ILMN_1376588	G6pdx	1.57	1.65	1.73	1.82	2.13	1.91	2.64
ILMN_1364042	Grina	1.23	1.66	1.2	1.95	2.2	2.05	2.64
ILMN_1352002	Cds1	1.15	1.49	1.27	1.35	2.13	2.06	2.64

ILMN_1370665	Zfand2a	1.41	1.92	1.91	2.44	6.63	6.31	2.64
ILMN_1373609	Tnfrsf12a	1.28	2.67	3.14	3.47	3.74	3.65	2.66
ILMN_1354864	Prpf19	1.59	1.63	1.79	1.96	2.12	2.42	2.68
ILMN_1367509	Igfbp1	3.39	4.08	3.79	1.99	3.44	3.92	2.69
ILMN_1369239	Arpc1b	1.23	1.54	2.29	2.01	2.37	2.44	2.7
ILMN_1349221	Trib3	1.76	4.42	4.61	5.08	6.3	6.03	2.71
ILMN_1350660	RGD1563250_predicted	1.15	2.08	1.6	1.45	2.22	2.24	2.72
ILMN_1356910	RGD1560729_predicted	1.22	1.48	1.06	1.76	1.69	1.88	2.74
ILMN_1350527	Flcn	1.63	2.7	2.92	2.18	2.94	3.26	2.74
ILMN_1363216	Prss15	1.39	2.39	5.09	3.42	3.38	3.29	2.75
ILMN_1356284	LOC501085	1.43	2.98	5.19	2.88	3.84	3.94	2.75
ILMN_1350180	Emp1	1.08	1.71	1.5	1.8	1.49	1.69	2.76
ILMN_2040144	Gns_predicted	1.11	1.66	1.79	1.72	2.01	2.21	2.76
ILMN_1358127	Plat	1.85	1.76	2.16	2.11	4.76	4.14	2.78
ILMN_1348991	RGD1564372_predicted	1.13	1.78	2.43	1.46	3.13	2.86	2.79
ILMN_1365569	Fstl3	1.64	3.34	3.46	3.52	2.24	2.34	2.82
ILMN_1351117	Ctsl	1.35	2.61	3.21	2.69	2.53	2.64	2.83
ILMN_1365385	Lgmn	1.36	3.38	4.03	2.38	2.51	2.81	2.83
ILMN_1353246	Pigp_predicted	1.08	1.9	1.34	1.61	1.97	2.06	2.85
ILMN_1376339	Ahr	1.35	2.04	1.49	2.25	2.46	3.28	2.85
ILMN_1369938	Slc20a1	1.9	3.56	4.56	4.12	2.03	2.36	2.86
ILMN_1363670	Ccng1	-1.13	-1.14	1.1	1.42	2.64	3.92	2.87
ILMN_1376328	Cd59	1.24	1.44	1.51	1.37	2.01	1.97	2.88
ILMN_1376748	Hexb	1.2	2.76	3.39	2.53	3.04	3.23	2.88
ILMN_1363750	Acsl1	1.26	1.91	2.2	1.51	2.59	2.3	2.91
ILMN_1650388	Tmem50b	-1.16	1.25	1.62	1.84	2.93	2.59	2.98
ILMN_2040605	RGD1560105_predicted	1.33	1.67	-1.17	1.93	2.09	2.43	2.99
ILMN_1357171	Adfp	1.3	3.58	3.27	3.68	3	2.79	3.03
ILMN_1349829	Mafk	1.61	2.39	2.48	2.49	4.48	4.67	3.03
ILMN_1355315	Dnajb9	1.35	1.75	2.28	2.01	7.13	6.86	3.06
ILMN_1355682	Cd63	1.28	2.32	3.11	2.85	2.75	2.81	3.07
ILMN_1650056	RGD1306658	1.23	1.62	2.32	2.84	2.51	2.57	3.09
ILMN_1368380	Asah1	1.06	1.67	1.83	1.98	2.29	2.21	3.1
ILMN_1368008	Mcoln1_predicted	1.13	1.77	2.36	2.22	2.86	2.88	3.1
ILMN_1363572	Mgmt	-1.03	1.87	1.66	2.12	3.05	3.27	3.11
ILMN_1365855	Atp6v1a1_predicted	1.57	1.52	2.9	2	2.77	2.76	3.17
ILMN_1370840	Lgals3	1.6	1.85	1.36	2.03	2.14	2.26	3.18
ILMN_1353139	Cstb	1.29	2.38	1.46	2.55	2.74	2.77	3.19

ILMN_1371122	Scpep1	1.39	2.59	3.13	2.19	3	3.17	3.19
ILMN_1357763	Rhoq	1.32	2.03	2.4	2.25	3.71	3.71	3.22
ILMN_1530390	MGC109491	1.48	1.11	1.3	1.31	3.26	4.14	3.23
ILMN_1372260	MGC109491	1.48	1.11	1.3	1.31	3.26	4.14	3.23
ILMN_1353138	Hspb8	1.58	1.29	1.76	1.67	4.17	4.62	3.29
ILMN_1362080	Klf6	1.5	2.22	2.07	1.57	4.5	5.25	3.32
ILMN_1361588	Slfn3	1.12	1.74	2.24	2	4.64	4.76	3.33
ILMN_1368297	RGD1305679	1.45	1.44	-1.03	1.12	2.26	1.74	3.37
ILMN_1368158	RGD1560592_predicted	1.2	2.02	3.08	1.84	3.85	3.37	3.44
ILMN_1650805	LOC364253	1.28	1.92	2.97	1.88	3.99	3.48	3.44
ILMN_1358267	Ctsd	-1.03	1.22	1.36	1.18	2.42	2.62	3.58
ILMN_1362806	Txnrd3_predicted	1.06	1.72	2.01	1.47	2.32	2.5	3.66
ILMN_1353579	RGD1309676	1.52	3.71	4.15	3.14	3.34	3.35	3.66
ILMN_1650285	Hmox1	1.57	3.28	4.45	3.81	4.24	3.76	3.68
ILMN_1367920	Eno2	1.41	4	2.88	5.1	4.6	3.49	3.71
ILMN_1650883	RGD1561956_predicted	1.23	1.93	2.75	1.9	3.87	3.24	3.76
ILMN_1650265	RGD1306873	1.34	4.57	3.83	4.03	3.11	3.01	3.78
ILMN_1355336	RGD1311122	1.25	2.32	3.04	2.12	4.5	3.6	3.87
ILMN_1368419	Sat_mapped	1.26	2.06	1.43	2.04	2.76	2.89	3.89
ILMN_1363504	Sult1c2	1.55	3.66	4.97	3.1	5.8	5.35	4.08
ILMN_1362353	LOC683385	1.92	1.78	2.6	2.68	5.09	5.61	4.1
ILMN_1358271	Gabarap	1.63	2.1	2.35	2.08	3.69	3.54	4.12
ILMN_1369241	Renbp	1.35	4.29	4.83	4.08	4	4.11	4.14
ILMN_1650700	LOC360941	-1.09	-1.08	-1.6	-1.45	-1.07	1.4	4.22
ILMN_1362645	RGD1561551_predicted	3.01	3.92	5.49	5.27	4.48	5.18	4.41
ILMN_1367559	RGD1559960_predicted	1.43	3.95	5.36	4.04	6.57	6.29	4.41
ILMN_1350480	S100a6	1.61	3.12	1.17	3.92	3.15	2.91	4.45
ILMN_1349910	Ddit3	1.99	5.61	8.08	8.45	13.49	11.54	4.51
ILMN_1357368	LOC497841	1.15	1.54	-1.06	1.81	3.1	2.21	4.56
ILMN_1376969	Creb3l3	1.5	6.32	3.42	3.33	5.42	4.3	5.21
ILMN_1376651	Arhgef2	1.62	4.56	5.56	6.56	7.65	7.57	5.21
ILMN_1350385	Tm6p1	1.17	6.42	5.93	5.67	5.79	4.37	7.01
ILMN_1351674	Gdf15	2.08	9.81	10.27	11.65	8.81	9.19	10.26
ILMN_1358849	LOC501211	1.21	2.28	2.03	4.5	7.18	12.62	11.56
ILMN_2039120	LOC502610	1.04	1.19	1.11	1.31	2.4	4.13	17.31

Table I. Gene differentially expressed in each step compared to control

	α-LA 6h	α-LA 18h	α-LA 24h	α-LA 30h	α-LA 42h	α-LA 48h	α-LA 72h
Total	13	413	459	490	639	631	594
Up	8	157	175	167	275	264	228
Down	5	256	284	323	364	367	366

Table II. Number of genes differentially expressed in each step compared to control after α-LA treatment
